# Reactivity of 1,3-enyne MIDA boronates: exploration of novel 1,2-alkyne shift *via gem*-difluorination†

**DOI:** 10.1039/d3sc06918d

**Published:** 2024-02-28

**Authors:** Samir Manna, Debasis Aich, Subrata Hazra, Shivam Khandelwal, Santanu Panda

**Affiliations:** a Department of Chemistry, Indian Institute of Technology Kharagpur Kharagpur 721302 India spanda@chem.iitkgp.ac.in

## Abstract

The discovery of a new class of heteroatom-rich boron-containing molecules (BCMs) and iterative cross-coupling (ICC) partners created a toolbox for future drug developments using organoboron compounds. Herein, we report the potential utility of 1,3-enyne MIDA boronates to access diverse *gem*-difluoro MIDA boronates *via* novel 1,2-alkyne shift. This unique reactivity of 1,3-enyne MIDA boronates offers facile access to previously challenging β-difluorinated alkyl borons. Furthermore, we demonstrated the synthesis of various novel furan-based BCMs *via* 5-*endo-dig* cyclization and iterative coupling partners *via* copper-catalyzed hydroboration and platinum-catalyzed diboration reaction.

## Abstract 2

Herein, we report the potential utility of 1,3-enyne MIDA boronates to access diverse *gem*-difluoro MIDA boronates *via* novel 1,2-alkyne shift, various novel furan-based BCMs *via* 5-*endo-dig* cyclization and iterative coupling partners *via* hydroboration and diboration reaction.

## Graphical abstract

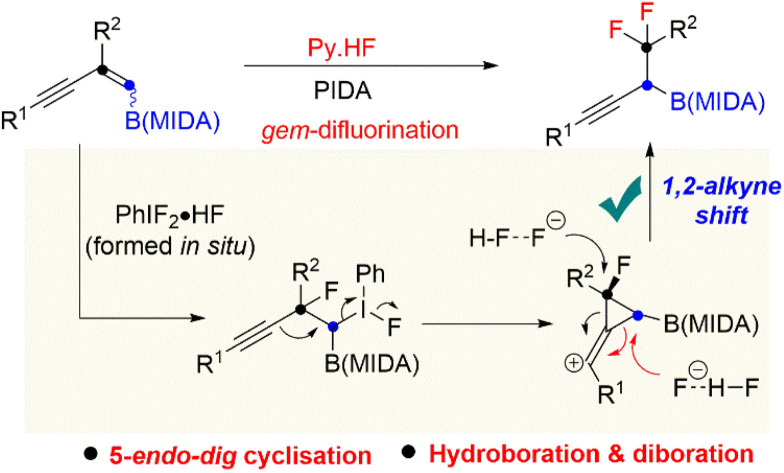

## Introduction

**Scheme 1:**
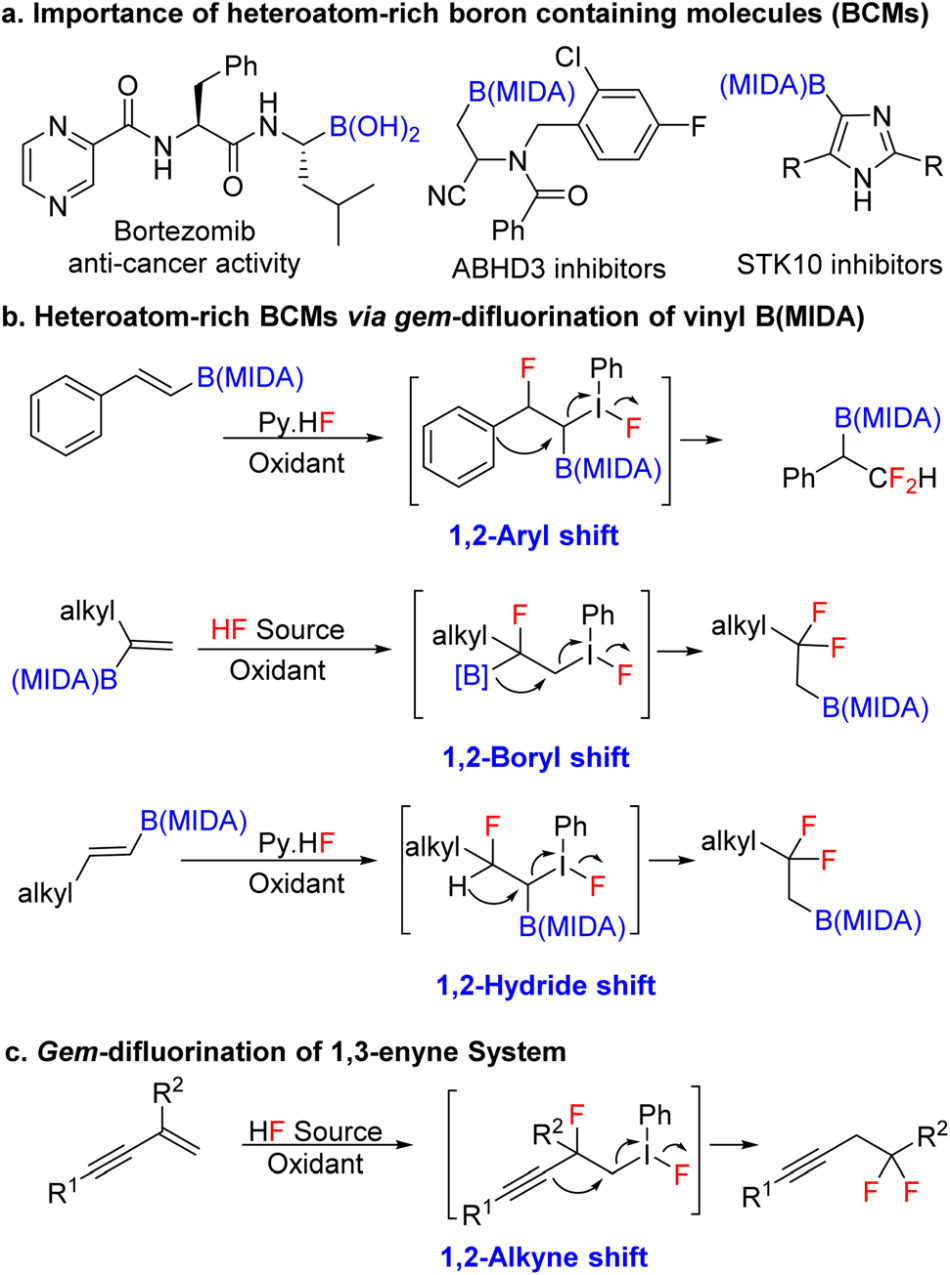
Previous work and our state of the art.

**Scheme 2:**
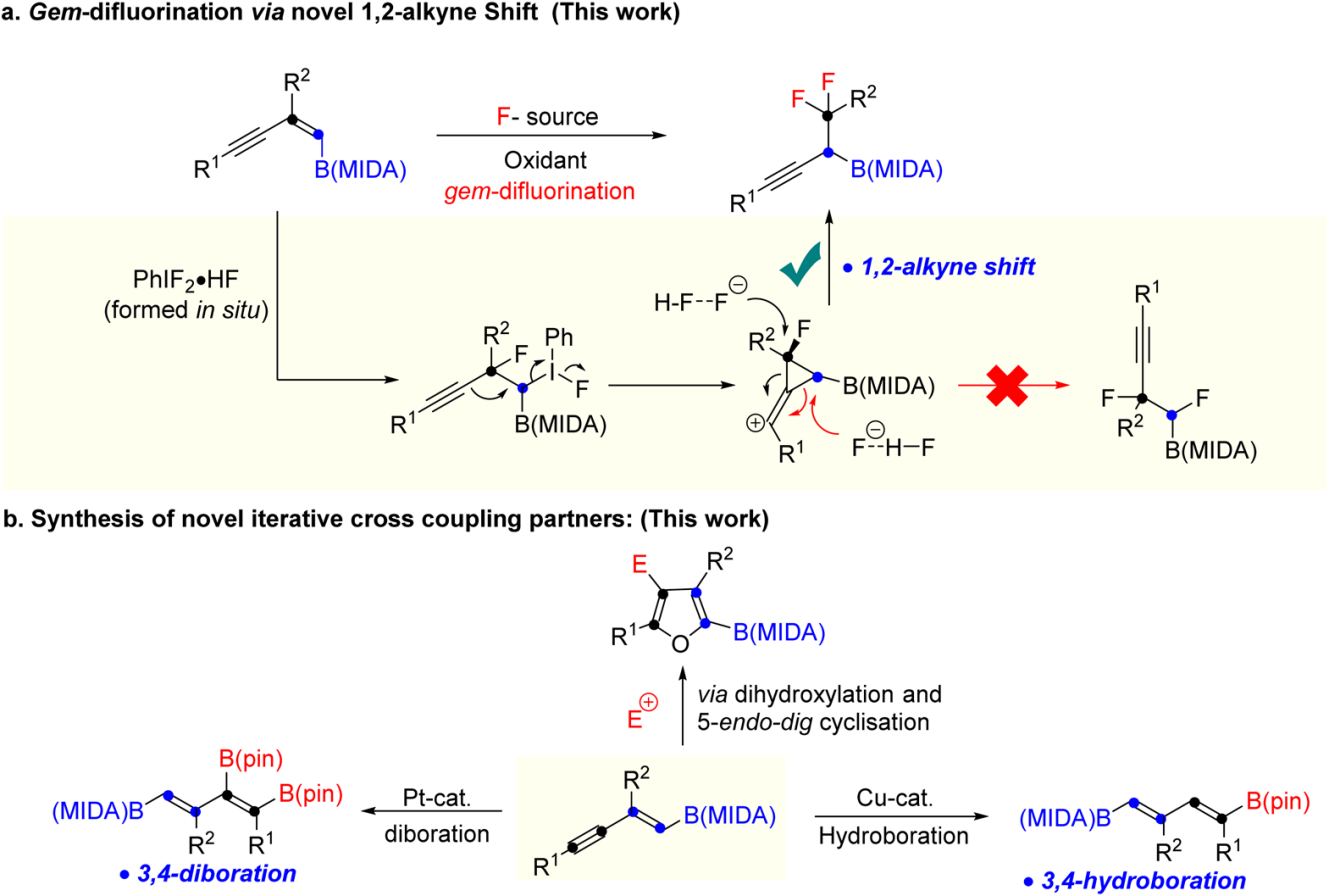
Our work.

Over the last two decades, organoboron compounds have received huge attention from medicinal chemists due to the revolutionary discovery of bortezomib, named Velcade.^1^ This ground-breaking invention has motivated synthetic chemists to develop a new path to achieve novel heteroatom-rich boron-containing molecules (BCMs) (Scheme 1a).^2^ Indeed, some of the valuable BCMs are difficult to access from the corresponding boronic acid or pinacol esters due to their instability under reaction conditions, which highlights the importance of the MIDA (*N*-methyliminodiacetic acid) as a protecting group for boron.^3^ There has been an ongoing interest in synthesizing sp^3^ and heteroatom-rich boron-containing molecules *via* the functionalization of vinyl MIDA boronates.^4^ Among the various types of heteroatom-rich BCMs, organofluorine compounds are extensively utilized in pharmaceuticals, agrochemicals, and materials science, which triggers the chemists to find a better way to synthesize a novel boron-based fluorinated architecture.^5^ After the subversive discovery of catalytic *gem*-difluorination of alkene surrogates,^6^ some interesting *gem*-difluorination reactions of vinyl MIDA boronates^7^ have been reported over the years (Scheme 1b). The electronic structures of the vinyl MIDA boronates have played an important role in the *gem*-difluorination using a hypervalent iodine platform.^7^ The Wang group stated that the 1,2-aryl^7*a*^ and 1,2-hydrogen^7*b*^ shifts have been shown to be the selective initiator for the regioselective *gem*-difluorination of aryl and aliphatic vinyl MIDA boronates (*trans* or *cis*), whereas, for the 1,1-alkyl vinyl MIDA boronates, the Szabó group pointed out that the 1,2-boryl shift^7*c*^ was predominant over the 1,2-hydrogen shift (Scheme 1b). While we were preparing the revised version of our manuscript, the Gilmour group reported *gem*-difluorination of the 1,3-enyne system by introducing novel 1,2-alkyne shift (Scheme 1c).^8^ However, a similar reactivity using 1,3-enyne MIDA boronates remains undiscovered. First of all, there will be two different pi-systems where the activation can take place. Also, there will be three possibilities for the difluorination of 1,3-enyne MIDA boronates *via* activating the vinyl boronate ester. It can go *via* 1,2-alkyne, 1,2-boryl, or 1,2-alkyl shifts when we treat with a stoichiometric amount of hypervalent iodine as an oxidant and Py·HF as a fluorine source (Scheme 2a). Gratifyingly, we are delighted to introduce the novel 1,2-alkyne shift of 1,3-enyne MIDA boronates during the *gem*-difluorination, using the stoichiometric hypervalent iodine and Py·HF (Scheme 2a).

Over the years a large number of MIDA-based skeletons have been explored by researchers across the globe for their application in the synthesis of heteroatom-rich boron-containing molecules (BCMs).^4,9^ However, the reactivity of conjugated MIDA-based boron compounds remains mostly unexplored, which hampers the utility of MIDA-based compounds in organic synthesis. Among the various conjugated MIDA boronates, 1,3-enyne MIDA boronates are one of the most diversifying boron moieties, as it has two types of π-system along with one stable C–B bond, which can be selectively activated to access previously unknown diverse fluorinated and heteroatom-rich BCMs and conjugated MIDA based iterative coupling partners, which have not been studied before.

Moreover, we have also explored a 5-*endo-dig* cyclisation of 1,2-boryl alcohol to access novel halofuran-based BCMs (Scheme 2b). Finally, a previously unknown copper catalysed hydroboration and platinum catalyzed diboration of 1,3-diene MIDA-boronate were achieved to synthesize new building blocks for iterative coupling (Scheme 2b). We have not found any previous literature, where copper catalysed hydroboration was reported using vinyl or alkynyl MIDA-boronates.

## Results and discussion

**Scheme 3:**
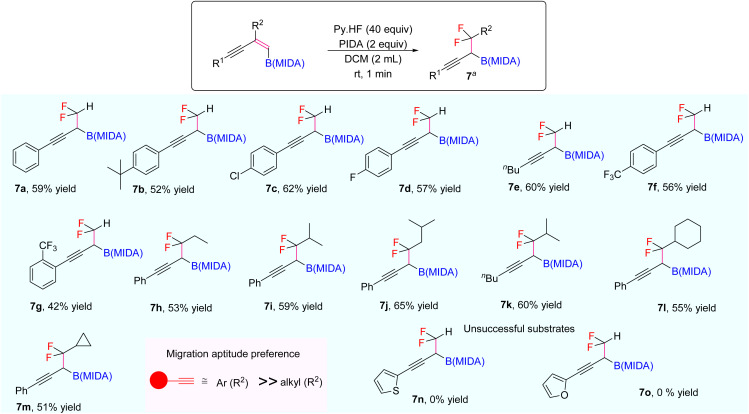
Scope of the *gem*-difluorination. ^*a*^1,3-Enyne B(MIDA) (0.2 mmol, 1 equiv.), PIDA (2 equiv.), HF·Py (40 equiv.), DCM (2 mL), rt, 1–2 min.

**Scheme 4:**
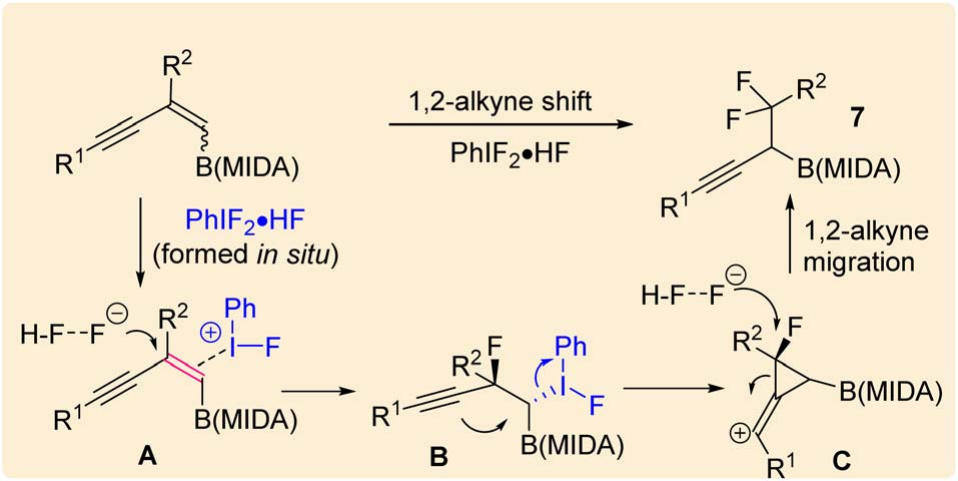
Mechanism of *gem*-difluorination.

Our investigations began with the synthesis of 1,3-enyne MIDA boronates. We hypothesized that the synthesis of 1,3-enyne MIDA boronates can be achieved *via* the boron-Wittig reaction between propargylic aldehydes or ketones with lithiated geminal boronic esters. Fortunately, we have successfully optimized and explored the stereospecific synthesis of di, tri-, and tetrasubstituted 1,3-enyne boronates using the boron-Wittig reaction followed by the transesterification strategy (detailed optimization and substrate scope of 1,3-enyne MIDA boronates *via* the boron-Wittig reaction 4a–4s, 5a–5o, and 6a–6i can be found in the ESI pages 2–4†). Now, our aim is to apply them for the stereoselective and regioselective synthesis of heteroatom-rich boron-containing molecules (BCMs) and iterative coupling partners. First, we set our focus on the synthesis of fluorine-containing BCMs *via* selective olefin activation. We are interested in determining the reactivity of our 1,3-enyne MIDA boronates in light of the recent literature on *gem*-difluorination. We hypothesized that the *gem*-difluorination on 1,3-enyne MIDA boronates might end up with three regioselective products (*via* 1,2-alkyne, 1,2-alkyl, and 1,2-boryl migrations).^7^ However, we are surprised to observe the formation of a single *gem*-difluorinated product under Wang's conditions^7*a*^ using PIDA/HF–Py. To test our hypothesis, we employed our 1,3-enyne MIDA boronates 4i using Wang's conditions^7*a*^ and observed the formation of 7i (52% yield) without any other regioisomers (Table 1, entry 1). The geometry of 7i emphasized that the product is formed by the regioselective 1,2-alkyne migration. By the systematic screening of the oxidants, the optimised yield of the 1,2-alkyne migration product 7i (59%) was examined. It was discovered that two equivalents of PIDA were required to achieve the optimal yield (Table 1, entry 5). With the optimized conditions in hand, we next explored the scope of the reaction (Scheme 3). We have screened both aliphatic and aromatic 1,3-enyne MIDA boronates with good yield and excellent regioselectivity (7a–7g). To check the electronic behaviour of the alkyne moiety, we screened both electron donation (7b) and withdrawing groups (7c, 7d, and 7f) and found that such type of 1,2-alkyne shift is more facile in the case of electron withdrawing cores. In the case of strongly electron-rich systems (like 5c and 5m, see ESI page 3†) the reactions were messy, which might be due to the strong affinity of the alkyne moiety as well as the alkene moiety towards the active catalyst. Similarly, the electronically diverse heteroaryl 1,3-enyne MIDA boronates (7n, 7m) have also not been tolerated under the optimized reaction conditions, which could be due to the same reactivity issue as strongly electron rich systems. To get a clear idea about the migration tendency of alkyne, alkyl, and boryl moieties, we screened both the di- and tri-substituted 1,3-enyne MIDA boronates and observed that the migration tendency of alkyne is higher than that of the alkyl or boryl moiety. To justify our result, we have proposed a novel vinyl carbocation C induced 1,2-alkyne migration mechanism based on the result and literature precedent (Scheme 4).^7^ First, the intermediate B is delivered by a regioselective vicinal fluoroiodination that is sparked by the reaction of the 1,3-enyne double bond and (difluoroiodo)benzene, which was generated *in situ* from PIDA and Py·HF. Then, the vinyl carbocation species C is formed as a result of the intramolecular nucleophilic attack of the alkyne moiety on the C–I bond. The second regioselective fluoride attack results in the ring-opening of C, which is accompanied by the homotopic 1,2-alkyne migration, furnishing a β-difluorinated alkyl boron compound. The higher electrophilicity of the carbon atom linked to a fluorine atom could be the cause of this selectivity. This report may be the second of its kind because the 1,2-alkyne migration is still uncommon.^10^

**Table 1:** Optimization of *gem*-difluorination

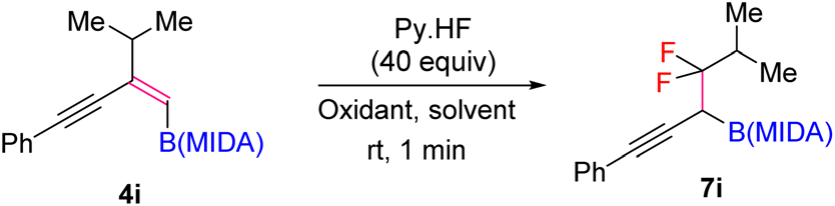			
Entry^a^	Oxidant (equiv.)	Solvent	Yield 7i (%)
1	PIDA (1.5)	DCM	52
2	PIDA (1.5)	DCE	42
3	PIDA (1.5)	Toluene	30
4	PIDA (1.5)	ACN	0
**5**	**PIDA (2)**	**DCM**	**59**
6	*m*-CPBA (2)	DCM	0
7	PIFA (2)	DCM	48

a 1,3-Enyne B(MIDA) (0.2 mmol), solvent (2 mL).

**Scheme 5:**
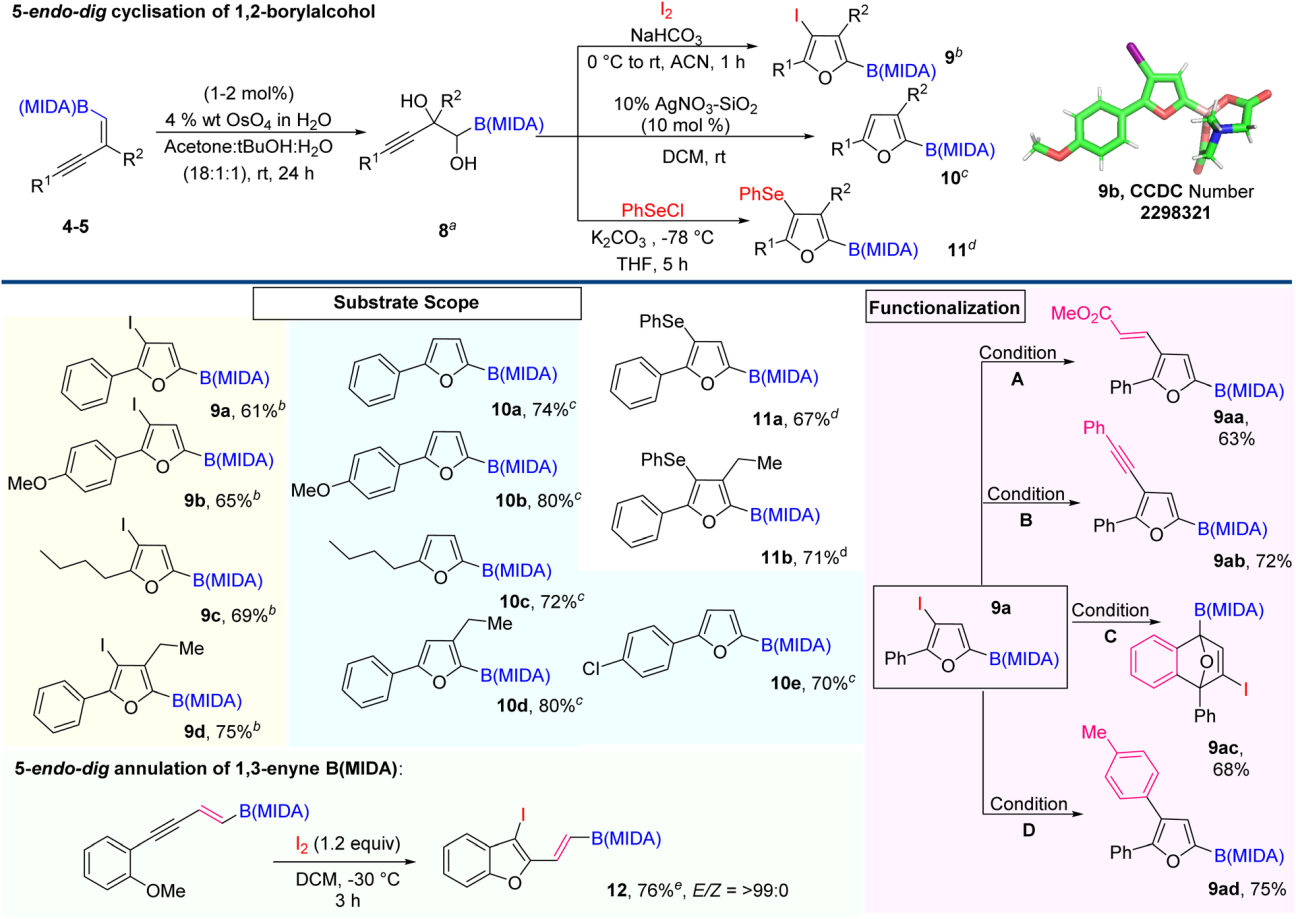
5-*endo-dig* cyclisation of 1,2-boryl alcohol. ^*a*^Dihydroxylation was achieved using 1–2 mol% of osmium tetroxide (4 wt% in H_2_O) and 1.5 equiv. of *N*-methylmorpholine *N*-oxide (NMO) in an 18 : 1 : 1 solvent ratio of acetone/*tert*-butano/water (0.05 M); ^*b*^iodocyclisation was done using I_2_ (3.3 equiv.) and NaHCO_3_ (3.3 equiv.) in ACN (0.1 M); ^*c*^AgNO_3_–SiO_2_ catalyzed cyclisation was done using 0.15 mmol scale (0.1 M) in DCM; ^*d*^selenocyclisation was done using K_2_CO_3_ (1.2 equiv.) and PhSeCl (1.1 equiv.) in THF solvent; conditions A: methyl acrylate (1.5 equiv.), Pd(OAc)_2_ (0.02 equiv.), PPh_3_ (0.15 equiv.), Et_3_N (2 equiv.), DMF (0.1 M), 90 °C, 19 h; conditions B: phenyl acetylene (1.5 equiv.), Pd(PPh_3_)_2_Cl_2_ (0.1 equiv.), Cul (0.05 equiv.), Et_3_N (3 equiv.), THF (0.1 M) RT, 4 h; conditions C: CsF (5 equiv.), 2-(trimethylsilyl)phenyl trifluoromethanesulfonate (3 equiv.), ACN (0.1 M), 60 °C, 24 h; conditions D: 4-methyl phenyl boronic acid (1.5 equiv.), Cs_2_CO_3_ (3 equiv.), Pd(OAc)_2_ (0.1 equiv.), XPhos (0.2 equiv.), THF (0.1 M), RT for 24 h. ^*e*^I_2_ (12 equiv.), DCM (0.05 M), −30 °C, 3 h.

Next, we explore the potential of 1,2-boryl alcohol (8) by introducing the 5-*endo-dig* cyclisation strategy.^11^ Here, we are very much excited to synthesize novel furan based BCMs, from the corresponding 1,2-boryl alcohol (8) by the Lewis acid-catalyzed cyclization and halocyclization (Scheme 5).^12^ To achieve our goal, we first attempted the Upjohn dihydroxylation on 1,3-enyne MIDA boronates.^1*d*,13^ The optimized conditions revealed that this dihydroxylation is fully concentration-dependent and the best yield (60%) came out using a 0.1 molar solvent system in a ratio of 18 : 1 : 1 (acetone: ^*t*^BuOH : H_2_O). Now, we turned our attention to exploring the electrophilic 5-*endo-dig* cyclization of 1,2-boryl alcohol 8 using iodine and phenyl selenium chloride as an electrophilic source.^12^ Most importantly, using this strategy we easily accessed borylated halo- and selenofurans (9 and 11), which will further participate in various types of metal-catalyzed coupling reactions. The integrity of the halofuran was confirmed by the X-ray data (9b). Next, we further carried out Lewis acid catalyzed 5-*endo-dig* cyclization using 10% w/w AgNO_3_–SiO_2_ and synthesized C-2 boryl substituted furans (10) with high yield.^12^ Importantly, this conversion was quantitative and we could not find any by-product except an equivalent amount of water. With the optimized conditions in hand, we demonstrated the scope of the AgNO_3_–SiO_2_ catalyzed 5-*endo-dig* cyclization with aromatic and aliphatic 1,3-enyne MIDA boronates, which is summarized in Scheme 5. The iodofuran product (9a) can be further derivatized to novel BCMs by employing Heck coupling (9aa), Sonaghashira coupling (9ab), Suzuki coupling (9ac), and [4 + 2] cycloaddition reaction (9ad). Interestingly, we were also able to introduce 5-*endo-dig* annulation of 1,3-enyne MIDA boronates by lowering the reaction temperature and synthesizing 2-vinyl 3-iodobenzofuran BCMs (12) with high yield and excellent stereoselectivity.^14^

**Scheme 6:**
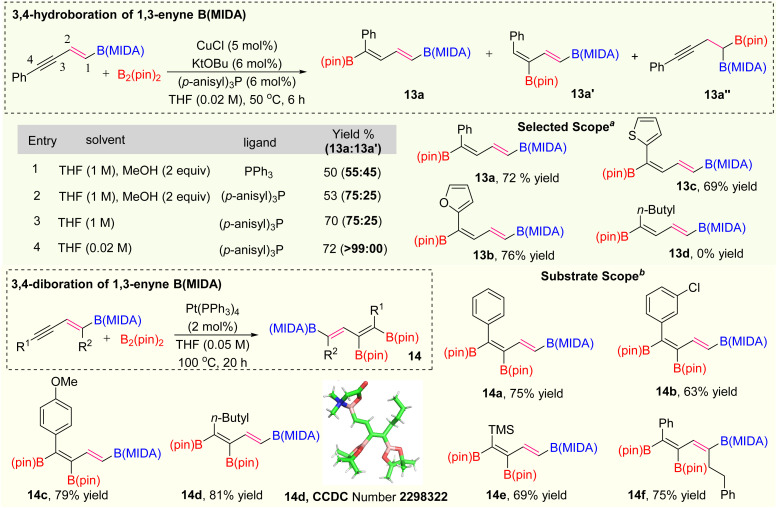
Hydroboration and diboration. ^*a*^Hydroboration using 1,3-enyne (MIDA) (1 equiv.), B_2_(pin)_2_ (1.2 equiv.); ^*b*^diboration using 1,3-enyne (MIDA) (1 equiv.), B_2_(pin)_2_ (1.2 equiv.).

After the selective activation of the alkene group, we are interested in activating the alkyne group of 1,3-enyne MIDA boronates for the synthesis of polyborylated-alkenes, which is the ultimate hope for the novel iterative cross-coupling partners.^15^ Over the last two decades several copper-catalyzed hydroboration and carboboration of 1,3-enyne systems have been known for the synthesis of diverse borylated compounds.^16^ However, we have not found any copper catalysed transformations known with either vinyl or alkynyl MIDA-boronates, which could be due to the possible elimination of the MIDA ligand. Based on the previous literature,^16*d*^ the regioisomeric preference of hydroboration of 1,3-enyne was totally controlled by the ligand catalysis. As our synthesized 1,3-enyne B(MIDA) compounds are sufficiently crowded over the double bond, we suspect that 1,3-dienylboronates 13a or 13a′ (*via* 3,4 or 4,3-hydroboration) will be our hydroboration products. After this initial understanding, we started our optimization using 5a as a model substrate with B_2_(pin)_2_ as a hydroboration coupling partner (Scheme 6). In entry 1, when we employed the Ito hydroboration^16*d*^ conditions (5 mol% CuCl, 6 mol% KO^*t*^Bu, 6 mol% PPh_3_, THF/MeOH), we ended up with the mixture of 3,4- and 4,3-hydroboration products (13a :  = 55 : 45) with poor yield. Interestingly, we have not found any 2,1-hydroboration product 13a′′. Next, we tuned the ligand environment for a better regioselective outcome of 3,4-hydroboration product 13a (for detailed optimization, see ESI pages 6 and 7†). We screened several monodentate and bidentate ligands, out of them *para*-anisyl phosphine gave the best (13a :  = 75 : 25) regioselective outcome. The lower yield in entries 1 and 2 (Scheme 6) is due to the deprotection of the MIDA protecting group in the presence of 2 equivalents of MeOH solvent.^17^ To improve the yield, we carried out the reaction using only THF solvent (1 M) and got the expected product in good yield (70%) with the same regioselectivity ratio (entry 3).^18^ We did not find any improvement in stereoselectivity by varying other solvent combinations. At this point, we decided to play with solvent concentration.^19^ The thorough optimization of the solvent concentration revealed that the 3,4-hydroboration is highly regioselective in 0.02 M solvent concentration. Having the optimized conditions in hand, we explored the scope of this hydroboration and found that aryl and heteroaryl 1,3-enyne MIDA boronates were working fine with good yield and diastereoselectivity (13a–13c). Interestingly, when we tested the scope of the aliphatic system (5h), no hydroboration product (13d) was obtained.

Next, we focused on the synthesis of tri-borylated olefin *via* selective diboration of the alkyne group of 1,3-enyne MIDA boronates (Scheme 6). We initially chose the previously unknown Pt(0)-catalyzed diboration strategy of 1,3-enyne MIDA boronates by following the standard diboration conditions of alkynes.^20^ We observed the formation of a 20% yield of the desired product using the literature conditions^20^ with some MIDA-deprotected compounds. To get the optimized yield of this diboration, we screened several polar and non-polar solvents, out of them, THF provided the best yield at 100 °C. We examined the scope of this diboration with aliphatic and aromatic 1,3-enyne MIDA boronates with high yield and diastereoselectivity (14a–14f). The structure of this tri-borylated olefin was further confirmed by the X-ray (14d). We have not found a single example, where 1,3-enyne MIDA boronate compounds were successfully employed for the Pt(0)-catalyzed diboration.^21^

**Scheme 7:**
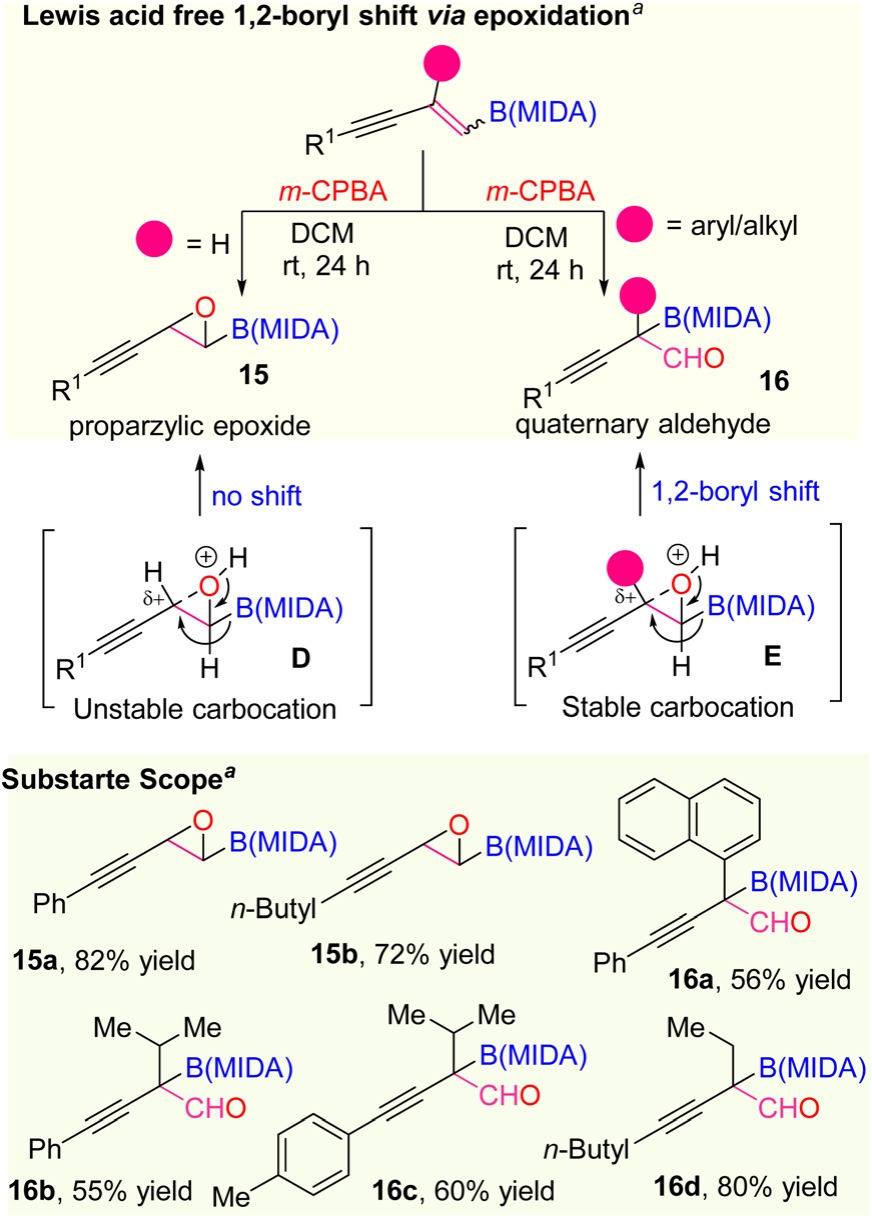
Olefin activation *via* epoxidation: ^*a*^epoxidation using *m*-CPBA (2.2 equiv.). DCM (0.05 M).

At last, we also extended the scope of the selective olefin activation of 1,3-enyne MIDA boronates by applying the epoxidation strategy using *m*-CPBA, established by the Yudin and Burke group (Scheme 7).^22^ For the disubstituted 1,3-enyne MIDA boronates, we observed only the epoxidation products (15a and 15b) but in the case of tri-substituted 1,3-enyne MIDA boronates, we ended up with amphoteric previously unknown quaternary α-boryl aldehydes (16a–16d). In general, the oxiranyl MIDA boronates need to be treated with a Lewis acid for the formation of α-boryl aldehydes.^22^ However, we observed one-step access to corresponding quaternary α-boryl aldehydes from the 1,3-enyne MIDA boronates without Lewis acid. We suspect that the difference in reactivity in the case of tri-substituted 1,3-enyne MIDA boronates *vs.* the di-substituted one is due to the stability of the quaternary propargylic carbocation (E), which can be formed in the presence of 3-chloro benzoic acid, a by-product from the *m*-CPBA reaction.

## Conclusions

In summary, we have explored the novel reactivity of 1,3-enyne MIDA boronates by applying selective olefin and alkyne activation strategies. By taking advantage of the unique 1,2-alkyne shift of 1,3-enyne MIDA boronates, a novel *gem*-difluorinated BCM has been synthesized *via* the vinyl carbocation intermediate. Notably, the success of this reaction is due to the alkene activation over the alkyne activation. Interestingly, a series of novel furan based BCMs have been synthesized by introducing the electrophilic and Lewis acidic catalyzed 5-*endo-dig* cyclization. To our delight, we found out the reason for the reactivity difference of substituted and unsubstituted propargyl oxirane MIDA boronates in the case of epoxidation. Finally, we have developed a new series of iterative cross-coupling partners by utilizing the concept of hydroboration and diboration techniques.

## Author contributions

SM carried out all the optimization, substrate scope and collected all the experimental data. DA, SH, and SK helped in the synthesis of starting materials. SP designed the project and provided direction for writing the manuscript.

## Conflicts of interest

The authors declare no competing financial interest.

## Supplementary Material

SC-015-D3SC06918D-s001

SC-015-D3SC06918D-s002
