# Supplementary material for: Reactivity of 1,3-enyne MIDA boronates: exploration of novel 1,2-alkyne shift via gem-difluorination
Source: Chem Sci. 2024 Feb 28;15(13):4989–95. doi: 10.1039/d3sc06918d (PMC10967263; doi:10.1039/d3sc06918d)
Supplement: SC-015-D3SC06918D-s001 [file SC-015-D3SC06918D-s001.pdf]

Electronic Supplementary Material (ESI) for Chemical Science.
This journal is © The Royal Society of Chemistry 2024

Reactivity of 1,3-enyne MIDA Boronates, Exploration of novel 1,2-Alkyne Shift via *gem*-Difluorination and Synthesis of diverse Iterative Coupling Partners

Samir Manna, Debasis Aich, Subrata Hazra, Shivam Khandelwal, Santanu Panda*

Supporting information

Table of Contents

General Information	S1
Synthesis of 1,3-enyne boronates	S2-S6
Optimization Table 3,4-hydroboration	S6-S7
Synthesis of Starting materials	S7-S9
General procedure	S9-S12
^{1}H-NMR & ^{13}C-NMR data for synthesized compounds	S12-S35
Crystallography data	S35-S37
Reference	S37-S38
^{1}H-NMR & ^{13}C-NMR Spectra	S38-S1578

General Information: Unless otherwise stated, reactions were performed under nitrogen using freshly purified solvents. All reactions were monitored by thin-layer chromatography with E. Merck silica gel 60 F254 pre-coated plates (0.25 mm). Flash chromatography was performed with indicated solvents using silica gel. The NMR spectra were recorded with a Bruker 400, 500 MHz NMR instrument. Data for ^{1}H NMR are reported as follows: chemical shift (ppm), integration, multiplicity (s = singlet, d = doublet, t = triplet, q = quartet, p = pentet, dd = doublet of doublets, ddd = doublet of doublets of doublets and m = multiplet), and coupling constant (Hz). Mass spectra were acquired on an Agilent technologies 1200 series LC/MS using indicated ionization methods. The known compounds were characterized by ^{1}H NMR, ^{13}C NMR, ^{11}B NMR, and ^{19}F NMR. For all the known compounds copy of ^{1}H-NMR, ^{13}C NMR and the appropriate references are given.

Materials: Chemicals were purchased from Aldrich, Alfa Aesar, Spectrochem, Avra, TCI and used without purification unless otherwise noted.

➢ <u>Synthesis of 1,3-enyne boronates</u>:

We hypothesized that if the boron-Wittig reaction of enynal with geminal B(pin) would be able to give 1,3-enyne MIDA boronates after transesterification, then our initial target of transition metal-free strategy would be conceived. However, such a strategy has to face some challenges related to the stereoselectivity issues when reacted with a ketone.[1] At the onset of our hypothesis, we began our finding with propargylic ketones. To find out our optimized reaction condition, we have done thorough optimization by varying the reaction temperature and concentration of the reaction and found that the temperature had a significant role in the progress of the reaction.

To optimized the stereoselective outcome of our designed targeted moiety, we have done through screening of all the reaction parameters. As the 1,3-enyne B(pin) (3) was highly unstable in the column chromatography, we have performed the transesterification with the crude reaction mixture. We also optimized the transesterification step and observed that the equivalences of MIDA ligand should be equal or higher than the 2.5 equiv.[2] Also the amount of proton source should be equal or higher than 4 equivalents. For the transesterification, we have optimized several temperatures out of them 100 °C is the best yielding in 12 hours. After fixed all the parameters, we have optimized the best yield 76% (Table 1, entry 7).

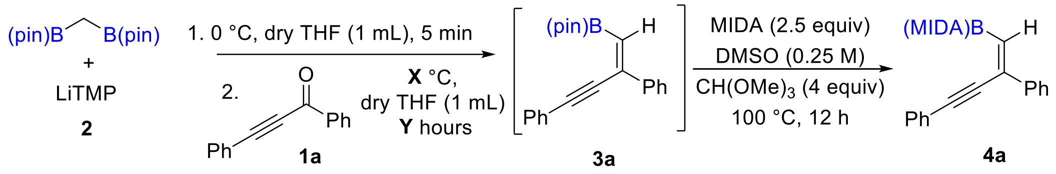

Entry[a,b]	**2** & **3** (equiv)	**1** (equiv)	Temperature (**X** °C)	Time (**Y** hours)	isolated Yield %
1	1.2	1	-78 °C	4 h	0
2	1.2	1	0 °C	2 h	50
3	1.2	1	RT	1 h	messy
4	1.2	1	0 °C	4 h	50
5	1.2	1	0 °C to RT	1 h	62
6	1.4	1	0 °C to RT	1 h	72
7	**1.5**	**1**	**0 °C to RT**	**1 h**	**76**
8	1.7	1	0 °C to RT	1 h	71

Table 1: LiTMP was made *in situ* using *n*-BuLi (1.5 equiv) and HTMP (1.5 equiv) at -78 °C, 30 min then 0 °C, 30 min; all the reactions was carried out at 0.475 mmol of **1**a.

(*Please note that the used HTMP should be highly dried, otherwise yield of this reaction was drastically diminished*.)

The optimized condition reveals that the addition of propargylic ketone at 0 °C to the α-bis(boryl)carbanions and then warming up to room temperature is the best yielding (76%) after the transesterification using MIDA in DMSO. Interestingly, the outcome of this boron-Wittig reaction with propargylic ketone gives only one isomer. The structure of all types of 1,3-enyne boronates were confirmed by the X-ray data (**4i**, **4r** and **6i**). However, the previous literature on the boron-Wittig

reaction of ketone showed poor stereoselectivity and dependency on the presence of amine additives to improve the stereoselective outcome. [1]

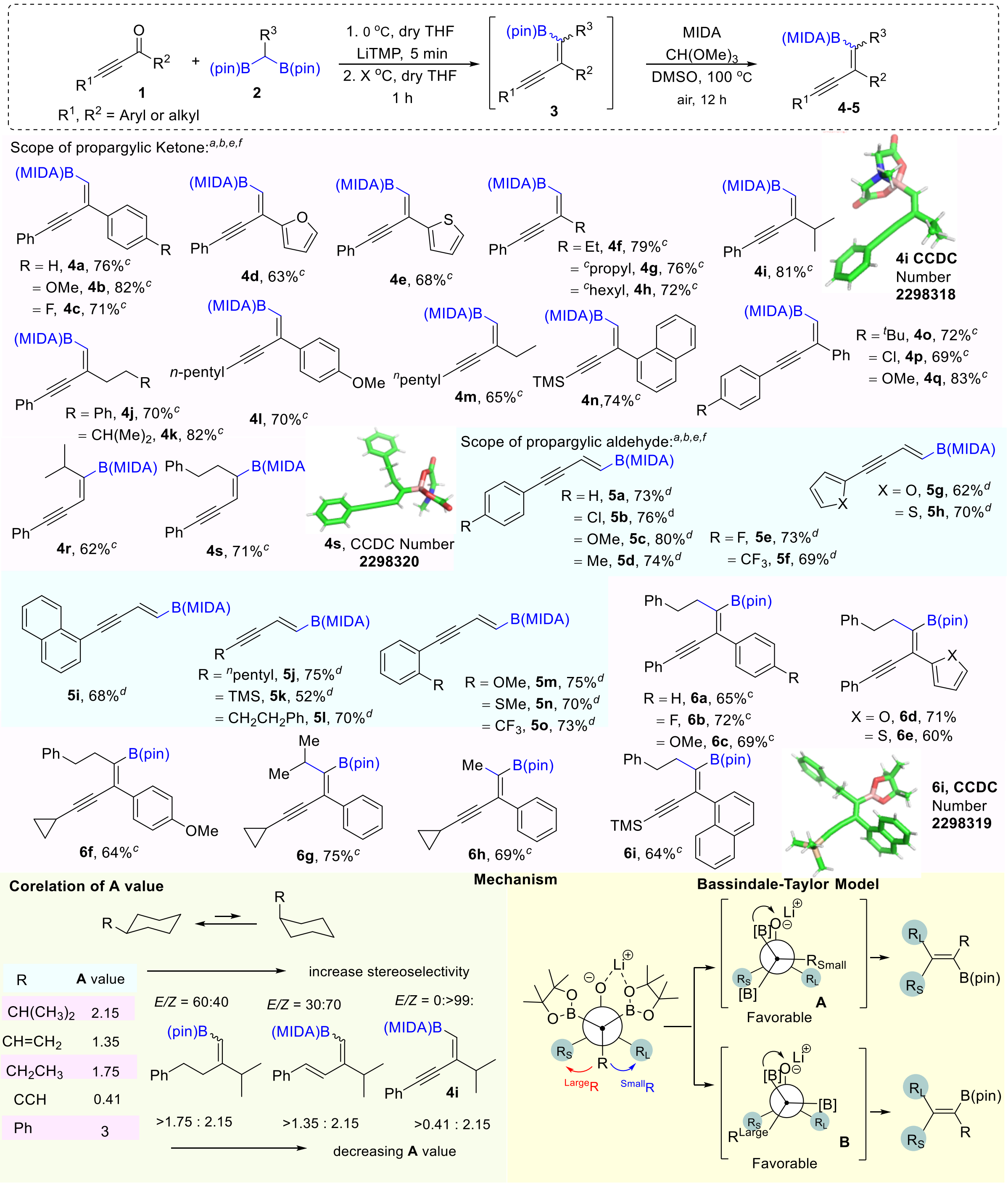

Table 2: Substarte Scope and Mechanism investigation [a] LiTMP was made *in situ* using HTMP and *n*-BuLi at -78 °C, 30 min then 0 °C, 30 min; [b] **1** (0.472 mmol), **2** (0.709 mmol), LiTMP (0.709 mmol); [c] **1** was added at 0 °C then warm up to RT for 1 h; [d] **1** was added at -78 °C and stirred for 4h; [e] all the cases crude enyne B(pin) was used for transesterification; [f] MIDA (1.18 mmol, 2.5 equiv), $CH(OMe)_3$ (1.88 mmol, 4 equiv), DMSO (2 mL)

Having optimized reaction conditions in hand, we have explored substrate scope by varying different propargylic ketones and substituted geminal B(pin). Aryl propargylic ketones with both electron-

[1] S. Namirembe, C. Gao, R. P. Wexler and J. P. Morken, *Org. Lett.*, 2019, **21**, 4392−4394

[2] E. M. Woerly, J. E. Miller and M. D. Burke, *Tetrahedron*, 2013, **69**, 7732-7740.

[3] J. R. Coombs, L. Zhang and J. P. Morken, *Org. Lett.*, 2015, **17**, 1708.

donating and electron-withdrawing group works well with high yield and diastereoselectivity (**4b** and **4c**). Most importantly, the reaction tolerated the heteroaromatic propargylic ketones efficiently with high yield and excellent diastereoselectivity (**4d** and **4e**). Further, when we employed sterically-biased aliphatic propargylic ketones, we were also able to get high diastereoselectivity and good yield (**4f-4j**). Variation of the aryl group attached to alkyne did not have any effect on the yield as well as stereoselectivity (**4n-4p**). Additionally, to make tri-substituted 1,3-enyne MIDA boronates, we examined the reactivity of substituted lithiated geminal bis(boronate)s with propargylic aldehyde and noticed the opposite selectivity (reverse with unsubstituted geminal bis(boronate) (**4q** and **4r**). We have proposed a steric model to explain the stereoselectivity outcome, which will be discussed later. Further, we explored the scope with propargylic aldehydes. Following the previous condition as reported by the Morken group,[3] we found the desired products (after the transesterification) with high yield and excellent selectivity (**5a-5n**). After successfully encountered di- and tri-substituted 1,3-enyne B(MIDA) compounds, we tuned our attention towards tetra substituted 1,3-enyne boronates. Using our optimized condition for propargyl ketone, we investigated the stereoselectivity outcome by reacting substituted geminal bis(boronate) compounds with propargylic ketones. We observed the steric-controlled Z-selective product (**6a-6i**). As such tetra substituted 1,3-enyne B(pin) are stable in column chromatography, so we are not interested in transesterification. Here, we also found the reflection of the steric model for explaining the stereoselectivity.

To get the exact reason for this excellent stereoselectivity, we have prepared three ketones with the same number of carbon as of **4i** (aliphatic, α,β-unsaturated and alkyne) and employed them in our optimized reaction condition (Table 1, entry 7). The outcome of this boron-Wittig reaction demonstrated that the stereoselectivity is increasing from aliphatic one to α,β-unsaturated to alkyne. To get a rough idea about the steric model, we have introduced the 'A' value of the R group of cyclohexane ring in the chair form (Table 2).[4] The 'A' value signifies the amount of energy cost of a 'R' group to stay at the axial position of the cyclohexane ring. As the 'A' value decreases from left (aliphatic) to right (alkyne), which correlates with the increasing stereoselectivity. This result suggested that the boron-Wittig reaction of propargylic ketone is controlled mostly by the steric model. To consolidate our hypothesis, we took the help of the well-known Bassindale and Taylor model of the addition of prochiral anion to prochiral aldehyde.[5] According to the model, the anion will approach the carbonyl group in such a way that the smallest substituent will be positioned between the two carbonyl substituents as shown in table 2. This preference will automatically position the smaller carbanion substituent next to the carbonyl compound's biggest substituent. Our result also fits with this model and signifies the dominion of a steric model.

1. <u>General procedure (**GP1**):</u> Synthesis of tri-substituted 1,3-enyne B(MIDA) (**4**):

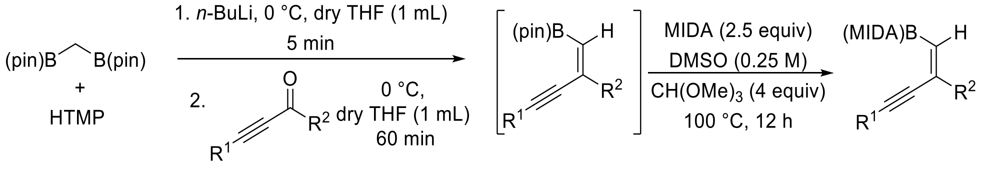

A 20 mL flame-dried reaction tube was first evacuated and backfilled with nitrogen three times then the tube was charged with a THF (1ml) solution of 2,2,6,6-tetramethylpiperidine (100 mg, 0.715 mmol, 1.5 equiv). After cooling the reaction tube at –78 °C, *n*-BuLi (1.2 M in hexanes, 0.6 mL, 0.709 mmol, 1.4 equiv) was added dropwise resulting in a light-yellow solution. The reaction mixture was stirred for

30 min[2]utes at -78 °C and then 30 minutes at 0 °C. After that, a THF (1 mL) solution of bis(4,4,5,5-tetramethyl-1,3,2-dioxaborolan-2-yl)methane (190 mg, 0.715 mmol, 1.5 equiv) was added drop wisely. The resulting mixture was stirred for 5 min at the same temperature then a solution of propargylic ketone (0.475 mmol, 1 equiv) in THF (1 mL) was added. The reaction tube was allowed to stir at room temperature for additional 1 hours. Upon completion, the reaction mixture was quenched by water and extracted with ethyl acetate. The organic layer was dried over Na_2SO_4, filtrated and concentrated under reduced pressure. The crude mixture was then undergoing a celite filtration using hexane as an eluent. The filtrate was the concentrated under reduced pressure and goes for the next step.

To an oven-dried schlenk tube equipped with a stir bar was added crude enyne boronates, anhydrous DMSO (2 mL), $CH(OMe)_3$ (4.0 equiv) and *N*-methyliminodiacetic acid (104 mg, 0.708 mmol, 2.5 equiv). The resulting mixture was stirred at 100 °C for 12 h. After cooling to room temperature, the reaction mixture was diluted with ethyl acetate (10 mL) and water (10 mL). The organic phase was seperated and the aqueous layer was extracted with ethyl acetate (10 mL) for three times. The combined organic layer was dried over anhydrous Na_2SO_4 and concentrated under reduced pressure. The resulting crude product was purified by flash chromatography on silica gel with a mixture of petroleum ether and ethyl acetate (1:3) as eluent to give 1,3-enyne MIDA boronates.

2. General procedure (**GP2**): Synthesis of di-substituted 1,3-enyne B(MIDA) (**5**):

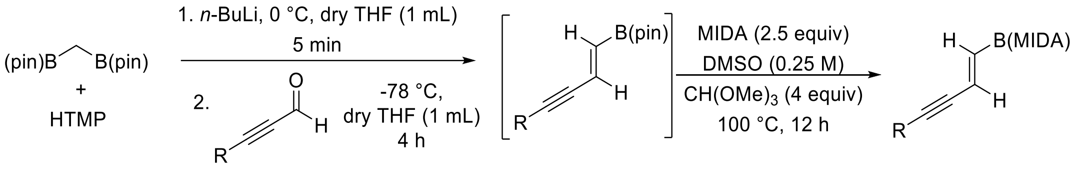

A 20 mL flame-dried reaction tube was first evacuated and backfilled with nitrogen three times then the tube was charged with a THF (1ml) solution of 2,2,6,6-tetramethylpiperidine (80 mg, 0.57 mmol, 1.2 equiv). After cooling the reaction tube at –78 °C, *n*-BuLi (1.2 M in hexanes, 0.6 mL, 0.709 mmol, 1.4 equiv) was added dropwise resulting in a light-yellow solution. The reaction mixture was stirred for 30 minutes at -78 °C and then 30 minutes at 0 °C. After that, a THF (1ml) solution of bis(4,4,5,5-tetramethyl-1,3,2-dioxaborolan-2-yl)methane (152 mg, 0.57 mmol, 1.2 equiv) was added drop wisely. The resulting mixture was stirred for 5 min at the same temperature then the reacture tube was transferred to -78 °C. After cooling at -78 °C, a solution of ynone (0.475 mmol, 1 equiv) in THF (1ml) was added. The reaction tube was allowed to stir at the same temperature for additional 4 hours. Upon completion, the reaction mixture was quenched by water and extracted with ethyl acetate. The organic layer was dried over Na_2SO_4, filtrated and concentrated under reduced pressure. The crude mixture was

[4] P. Muller, *Pure and Applied Chemistry*. 1994, **66**, 1077.

[5] A. R. Bassindale, R. J. Ellis, J. C.-Y. Lau and P. G. Taylor, *J. Chem. Soc. Chem. Commun.*, 1986, **98**.

then undergoing a celite filtration using hexane as an eluent. The filtrate was the concentrated under reduced pressure and goes for the next step.

To an oven-dried schlenk tube equipped with a stir bar was added crude enyne boronates, anhydrous DMSO (2 mL), $CH(OMe)_3$ (4.0 equiv) and *N*-methyliminodiacetic acid (104 mg, 0.708 mmol, 2.5 equiv). The resulting mixture was stirred at 100 °C for 12 h. After cooling to room temperature, the reaction mixture was diluted with ethyl acetate (10 mL) and water (10 mL). The organic phase was seperated and the aqueous layer was extracted with ethyl acetate (10 mL) for three times. The combined organic layer was dried over anhydrous Na_2SO_4 and concentrated under reduced pressure. The resulting crude product was purified by flash chromatography on silica gel with a mixture of petroleum ether and ethyl acetate (1:3) as eluent to give 1,3-enyne MIDA boronates.

3. General procedure (**GP3**): Synthesis of tetra-substituted 1,3-enyne B(pin) (**6**):

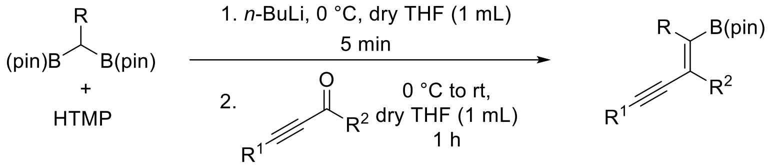

A 20 mL flame-dried reaction tube was first evacuated and backfilled with nitrogen three times then the tube was charged with a THF (1ml) solution of 2,2,6,6-tetramethylpiperidine (100 mg, 0.715 mmol, 1.5 equiv). After cooling the reaction tube at –78 °C, *n*-BuLi (1.2 M in hexanes, 0.6 mL, 0.709 mmol, 1.5 equiv) was added dropwise resulting in a light-yellow solution. The reaction mixture was stirred for 30 minutes at -78 °C and then 30 minutes at 0 °C. After that, a THF (1ml) solution of substituted germinal B(pin) (0.715 mmol, 1.5 equiv.) was added drop wisely. The resulting mixture was stirred for 5 min at the same temperature then a solution of ynone (0.472 mmol, 1 equiv) in THF (1ml) was added. The reaction tube was allowed to stir at room temperature for additional 1.5 hours. Upon completion, the reaction mixture was quenched by water and extracted with ethyl acetate. The organic layer was dried over Na_2SO_4, filtrated and concentrated under reduced pressure. The tetra substituted 1,3-enyne boronates were purified by flash chromatography on silica using a mixture of ethyl acetate and hexane as eluent.

1. Optimization table of 3,4-hydroboration of 1,3-Enyne MIDA boronates:

To optimized the stereoselective 3,4-hydroboration of 1,3-enyne B(MIDA), we have screened both the solvents and ligands, which were listed in the table 2. From this optimization table, we observed that such kind of copper catalysis was more efficient without the proton source (MeOH) and high concentration dependent. After the vigorous optimization, we found that the reaction was highly stereoselective using 0.02 M THF solvent and *p*-anisyl phosphine as a ligand at 50 °C temperature.

3,4-hydroboration of 1,3-enyne B(MIDA):

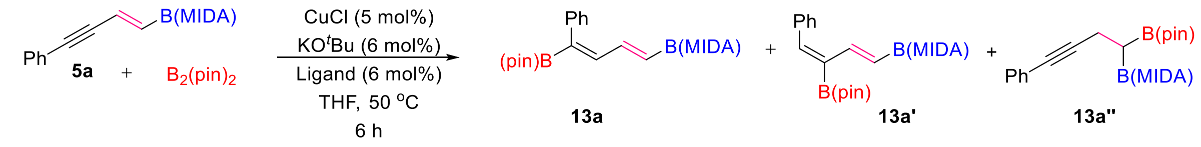

Entry	solvent	ligand	Yield % (13a:13a')
1	THF (1 M), MeOH (2 equiv)	PPh_3	40 (**55:45**)
2	THF (1 M), MeOH (2 equiv)	(p-anisyl)$_3$P	43 (**75:25**)
3	THF (1 M), MeOH (2 equiv)	(p-tolyl)$_3$P	43 (**65:35**)
4	THF (1 M), MeOH (2 equiv)	(furyl)$_3$P	43 (**60:40**)
5	THF (1 M), MeOH (2 equiv)	(Cy)$_3$P	nd
6	THF (1 M), MeOH (2 equiv)	Xantphos	nd
7	THF (1 M)	(p-anisyl)$_3$P	70 (**75:25**)
8	THF (0.5 M)	(p-anisyl)$_3$P	73 (**80:20**)
9	THF (0.02 M)	(p-anisyl)$_3$P	72 (**>99:00**)

Table 3: 5a (0.1 mmol, 1 equiv), $B_2(pin)_2$ (0.12 mmol, 1.2 equiv), nd = not detected

➢ Synthesis of Stating Materials:

1. Synthesis of geminal B(pin):

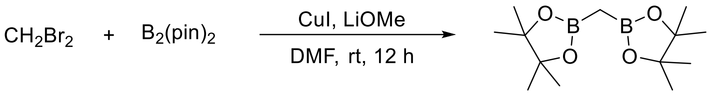

To an oven-dried 250 mL round-bottomed flask equipped with a Teflon coated magnetic stirbar, copper iodide (1.0 g, 5.5 mmol), LiO^{t}Bu (5.2 g, 137.5 mmol) and bis(pinacolato)diboron (25 g, 100 mmol) were added. The flask was evacuated and filled with argon (three cycles). DMF (150 mL) and dibromomethane (9.6 g, 55 mmol) were added *via* syringe under an argon atmosphere. The reaction mixture was stirred at 40 °C for 24 h, and then diluted with n-hexane (150 mL). The organic phase was washed with H_2O (200 mL) and the aqueous layer was extracted with n-hexane (200 mL x 3). The combined organic layers were dried over $MgSO_4$, filtered through silica gel and concentrated under reduced pressure. The crude mixture was purified by column chromatography on silica gel to give expected product (9.4 g, 70 %) as a white solid.[1]

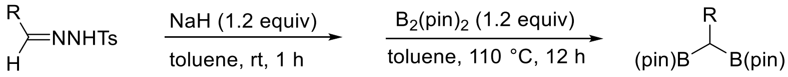

A modified Schlenk tube was charged with *N*-tosylhydrazone (1 mmol), 60% NaH (1.2 mmol, 48 mg). After degassed and filled with N_2, the tube was charged with toluene (8 mL). The mixture was stirred at room temperature for 1 h. Then a solution of B_2pin_2 (1.2 mmol, 305 mg) in toluene (2 mL) was added *via* syringe. Then the tube was sealed and heated at 110 for 12 h. After cooled to room temperature, 10 mL of Et_2O and 10 mL of H_2O were added. The mixture was stirred vigorously for 10 minutes. After separation of organic layer, the aqueous layer was extracted with Et_2O (5 mL×2). The combined organic

solution was washed with saturated brine (10 mL) and dried over anhydrous Na_2SO_4. After the solvent was evaporated, the crude product was purified by silica gel chromatography.[2]

2. Synthesis of Propargylic aldehyde and ketone:

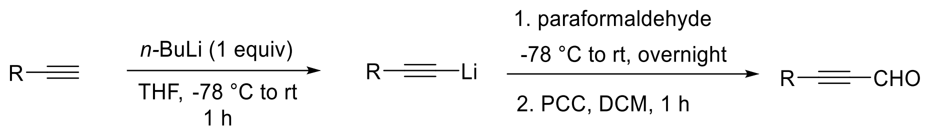

First Step: To an oven-dried 250 mL round-bottomed flask equipped with a Teflon coated magnetic stirbar and alkyne (10 mmol) in THF (25 ml) was cooled to -78 °C and charged with *n*-BuLi (1 equiv). The reaction mixture was stirred at the same temperature for 30 min then at room temperature for another 30 min. After that solid paraformaldehyde was added to the reaction mixture at -78 °C then the reaction mixture was transferred to room temperature and stirred for overnight. After the completion of the reaction, water was added and extracted the propargylic alcohol using ethyl acetate (3 times). The crude reaction mixture was dried over anhydrous Na_2SO_4 and concentrated in rotary evaporator. For the next step, the crude propargylic alcohol was used without purification.

Second Step: In a round-bottomed flask equipped with a Teflon coated magnetic stirbar and crude propargylic alcohol (1 equiv) in DCM was treated with PCC (1.5 equiv) at room temperature. After 1 h of stirring, the reaction was filtered through a silica pad using DCM solvent. Then the crude reaction mixture was concentrated in rotary evaporator and performed a flash column chromatography using 4 % ethyl acetate/hexane as eluent.

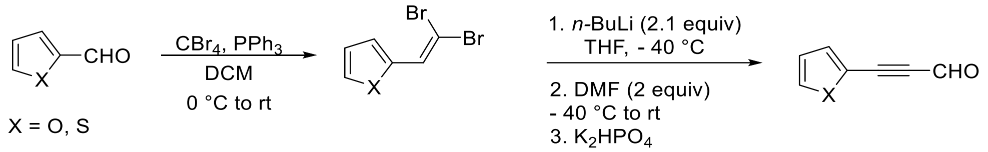

First Step: To a solution of aldehyde (1.0 equiv.) and CBr_4 (1.05 equiv.) in anhydrous DCM (0.45 M) cooled down to 0 °C was added Ph_3P (2.1 equiv.) as a solid in small portions. The resultant yellow reaction mixture was then stirred at ambient temperature for 3 h. Solvent was removed in vacuo, and the residue was dissolved in *n*-hexane. Triphenylphosphine oxide was filtered off by suction. The filtrate was concentrated under reduced pressure, and the crude gem-dibromide was purified by flash chromatography on silica gel using n-hexane/EtOAc as eluent.

Second Step: A well-stirred solution of gem-dibromide (1.0 equiv.) in anhydrous THF (0.4 M) was cooled down to -40 °C. *n*-BuLi (1.6 M solution in hexanes, 2.1 equiv.) was then added dropwise *via* syringe. The solution was stirred at -50 °C for 15 min, and anhydrous DMF (2.0 equiv.) was added in one portion. The mixture was allowed to slowly reach ambient temperature. After stirring for further 1 h, the reaction mixture was poured into a vigorously stirred mixture of KH_2PO_4 and tert-butyl methyl

ether. The layers were separated and the aqueous layer was extracted with *tert*-butyl methyl ether. The combined organic layers were washed with brine, dried over anhydrous Na_2SO_4, filtered and concentrated under reduced pressure. The crude oil was purified by flash chromatography on silica gel using *n*-hexane/EtOAc as eluent.[3]

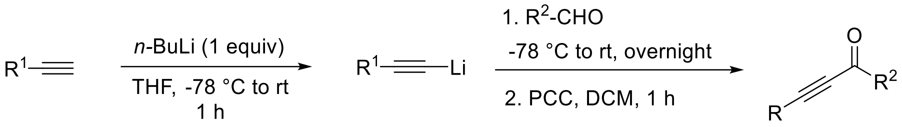

First Step: To an oven-dried 250 mL round-bottomed flask equipped with a Teflon coated magnetic stirbar and alkyne (10 mmol) in THF (25 ml) was cooled to -78 °C and charged with *n*-BuLi (1 equiv). The reaction mixture was stirred at the same temperature for 30 min then at room temperature for another 30 min. After that aldehyde was added to the reaction mixture at -78 °C then the reaction mixture was transferred to room temperature and stirred for 1.5 h. After the completion of the reaction, water was added and extracted the propargylic alcohol using ethyl acetate (3 times) as a solvent. The crude reaction mixture was dried over anhydrous Na_2SO_4 and concentrated in rotary evaporator. For the next step, the crude propargylic alcohol was used without purification.

Second Step: In a round-bottomed flask equipped with a Teflon coated magnetic stirbar and crude propargylic alcohol (1 equiv) in DCM was treated with PCC (1.5 equiv) at room temperature. After 1 h of stirring, the reaction was filtered through a silica pad using DCM solvent. Then the crude reaction mixture was concentrated in rotary evaporator and performed a flash column chromatography using 2 % ethyl acetate/hexane as eluent.

➢ General Procedure:

A. General procedure (**A**): geminal difluorination *via* 1,2-alkyne migration (**7**):

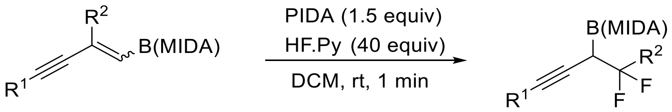

To a stirred solution of 1,3-enyne MIDA boronates (1.0 equiv), $PhI(OAc)_2$ (2 equiv) in DCM (0.1 M) in a 15 mL of Schlenk tube under ambient atmosphere was added Py.HF (40.0 equiv) in one portion. The reaction was allowed to stir at 25 °C until the complete consumption of boronates as monitored by TLC analysis (typically 1 min). The reaction mixture was directly purified by flash column chromatography on silica with an appropriate solvent to afford the pure product.

B. Synthesis of 1,2-boryl alcohol:

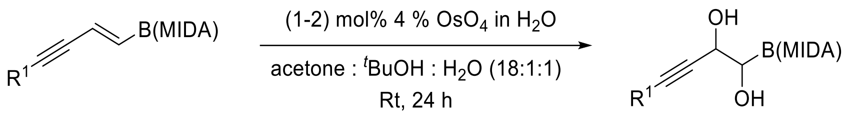

The pure 1,3-enyne MIDA boronate was dissolved in an 18:1:1 solvent mixture of acetone:*tert*-butanol:water (0.1 M) in an oven dried round bottom flask equipped with a magnetic stir bar. A solution of 4% wt of OsO_4 in H_2O (1 – 2 mol%) was added followed by the addition of *N*-methylmorpholine *N*-oxide (1.5 equiv.) at room temperature under nitrogen atmosphere. The resulting reaction mixture was allowed to stir at room temperature for 24 – 36 hours until complete starting material consumption as indicated by TLC analysis. Upon completion, most of the solvent was removed in vacuo to afford an oil residue. The crude product then undergoes a quick flash column chromatograph using 50% acetone/hexane as eluent.

C. General procedure (**B**): Synthesis of furan *via* iodocyclisation (**9**):

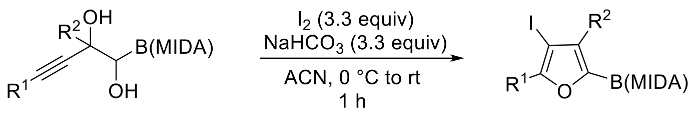

In a 10 ml dried round bottom flask containing a magnetic bar was treated with 1,2-boryl alcohol in acetonitrile solvent (0.1 M). The reaction chamber was then cooled to 0 °C and charged with $NaHCO_3$ (3.3 equiv) and stirred for 5 min at the same temperature. After that, a solid I_2 (3.3 equiv) was added into the mixture and stirred for another 5 min at the same temperature. Then the cooling bath was removed and stirring continued at room temperature until the total consumption of staring material. After that saturated thiosulphate solution was added until the color of iodine disappears. The crude reaction mixture was then concentrate under reduce pressure and extracted with ethyl acetate (3 times). The oily crude product further undergoes flash column chromatograph using 60% ethyl acetate/hexane as an eluent.

D. General procedure (**C**): Synthesis of substituted furan BCMs (**10**) using $AgNO_3$-SiO_2:

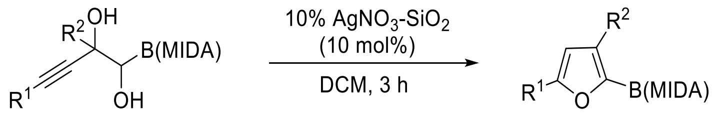

Following that, the 1,2-boryl alcohol was exposed to a 5-*endo-dig* cyclization process utilizing 10 mol% commercial 10% w/w silver(I) nitrate on silica gel in DCM (0.1 M) at room temperature for roughly 3 hours. After 3 hours of stirring at room temperature, the crude reaction mixture was run through a flash column chromatograph with an eluent of 60% ethyl acetate/hexane.

E. General procedure (**D**): Synthesis of furan *via* seleonocyclisation (**11**):

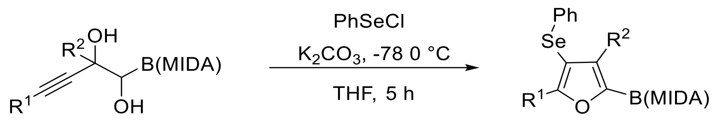

In a 10 ml dried round bottom flask containing a magnetic bar was treated with 1,2-boryl alcohol in THF solvent (0.1 M). The reaction chamber was then cooled to -78 °C and charged with K_2CO_3 (1.1 equiv) and PhSeCl (1.2 equiv). Then, the mixture was stirred for 5 h at the same temperature. After that saturated thiosulphate solution was added to quench the reaction. The crude reaction mixture was then extracted with ethyl acetate and water (3 times). The oily crude product further undergoes a flash column chromatograph using 60% ethyl acetate/hexane as an eluent.

F. General procedure (**E**): 3,4-hydroboration of 1,3-enyne MIDA boronates (**13**):

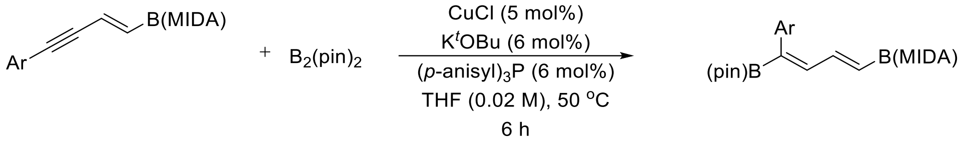

In a 10 ml reaction tube containing a stir bar was treated with CuCl (5 mol%), K^{t}OBu (6 mol%), and (*p*-anisyl)$_3$P (6 mol%). The reaction tube was degassing with nitrogen three times before adding THF (2 ml). Then the reaction mixture was stirred at room temperature for 10 min to form the black color solution. After that 1,3-enyne MIDA boronate (1 equiv, 0.1 mmol) and B_2(pin)$_2$ (1.2 equiv, 0.12 mmol) in THF (2 ml) was added in reaction mixture and stirred at 50 °C for 5-8 h. After the reaction was completed, the reaction mixture was directly subjected to flash column chromatography to give the corresponding bisborotate as a colorless oil or color oil.

G. General procedure (**F**): 3,4-diboration of 1,3-enyne MIDA boronates (**14**):

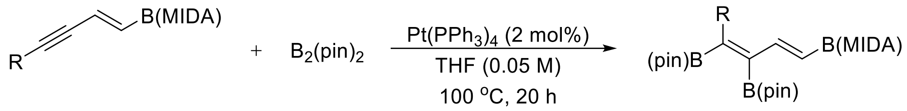

In a 10 ml reaction tube containing a stir bar was treated with 1,3-enyne MIDA boronates (1 equiv), Pt(PPh$_3$)$_4$ (2 mol%), and B_2(pin)$_2$ (1.2 equiv). The reaction tube was degassing with nitrogen three times before adding THF (0.05 M). Then the reaction mixture was stirred at 100 °C for 20 h to complete the conversion. After the reaction was completed, the reaction mixture was directly subjected to flash column chromatography to give the corresponding trisborotate as a white powder or gammy oil.

E. General procedure (**G**): epoxidation of 1,3-enyne B(MIDA):

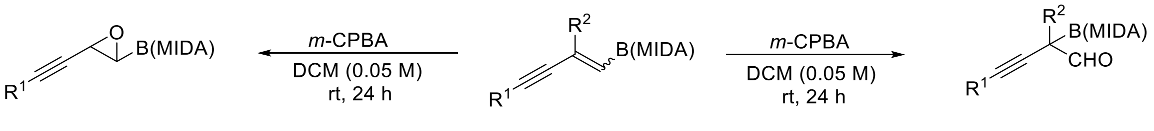

To a stirred solution of 1,3-enyne MIDA boronates in DCM (0.05M) at room temperature m-CPBA (2.2 equiv) was added and stirred for 24 h. When the reaction was completed (confirmed by TLC), the reaction mixture was quenched by aqueous $NaHCO_3$ solution and extracted using DCM (2 times). Then the crude reaction was directly subjected to flash column chromatography to give the corresponding epoxide or quaternary aldehyde.

➢ ^{1}H-NMR & ^{13}C-NMR data of Synthesized compounds:

(*Z*)-2-(2,4-diphenylbut-1-en-3-yn-1-yl)-6-methyl-1,3,6,2-dioxazaborocane-4,8-dione (**4a**):

According's to the **general procedure GP1**, white solid, 76% yield. ^{1}H NMR (500 MHz, DMSO) δ 7.79 (d, *J* = 8 Hz, 2H), 7.59-7.58 (m, 2H), 7.46-7.42 (m, 5H), 7.39-7.36 (t, *J* = 7 Hz, 1H), 6.52 (s, 1H), 4.36 (d, *J* = 17 Hz, 2H), 4.10 (d, *J* = 17 Hz, 2H), 2.92 (s, 3H). ^{13}C NMR (126 MHz, DMSO) δ 169.06, 139.18, 133.68, 131.50, 128.76, 128.49, 128.44, 128.28, 125.91, 122.34, 94.49, 88.69, 62.27, 47.18. ^{11}B NMR (160 MHz, DMSO) δ 10.06. HRMS (ESI) m/z cal. for $(M + H)^+$ 360.1402, found 360.1409.

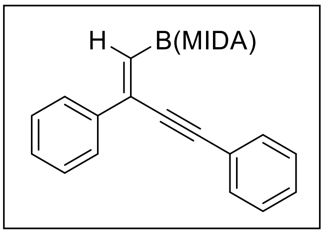

(*Z*)-2-(2-(4-methoxyphenyl)-4-phenylbut-1-en-3-yn-1-yl)-6-methyl-1,3,6,2-dioxazaborocane-4,8 dione (**4b**):

According's to the **general procedure GP1**, yellow semi solid, 82 % yield. ^{1}H NMR (500 MHz, DMSO) δ 7.72 (m, 2H), 5.56 (m, 2H), 7.41 (m, 3H), 6.99 (m, 2H),6.37 (s, 1H), 4.33 (d, *J* = 13.6 Hz, 2H), 4.07 (d, *J* = 14 Hz, 2H), 3.79 (s, 3H), 2.89 (s, 3H). ^{13}C NMR (126 MHz, DMSO) δ 169.08, 159.47, 133.08, 131.60, 131.48, 128.72, 128.49, 127.21, 122.41, 113.81, 94.27, 88.89, 62.26, 55.18, 47.19. ^{11}B NMR (160 MHz, DMSO) δ 10.31. HRMS (ESI) m/z cal. for $(M+ NH_4)^+$ 407.1773, found 407.1881.

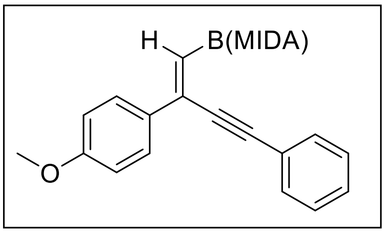

(*Z*)-2-(2-(4-fluorophenyl)-4-phenylbut-1-en-3-yn-1-yl)-6-methyl-1,3,6,2-dioxazaborocane-4,8-dione (**4c**): According's to the **general procedure GP1**, white semi solid, 71 % yield. ^{1}H NMR (500 MHz, DMSO) δ 7.83 (dd, *J* = 5.5 Hz, 2H), 7.59 (dd, *J* = 3.5 Hz, 2H), 7.43-7.42 (m, 3H), 7.27 (t, *J* = 9 Hz, 2H), 6.48 (s, 1H), 4.35 (d, *J* = 17 Hz, 2H), 4.09 (d, *J* = 17 Hz, 2H), 2.91 (s, 3H). ^{13}C NMR (126 MHz, DMSO) δ 169.04, 163.10, 161.14, 135.63 (d, *J* = 3.0 Hz), 132.45, 131.52, 128.82, 128.49, 128.02 (d, *J* = 8.3 Hz), 122.23, 115.27 (d, *J* = 21.6 Hz), 94.61, 88.47, 62.23, 47.16. ^{11}B NMR (160 MHz, DMSO) δ 10.24. HRMS (ESI) m/z cal. for $(M + H)^+$ 378.1307, found 378.1317.

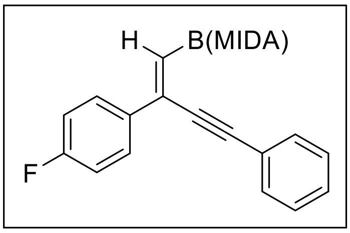

(*E*)-2-(2-(furan-2-yl)-4-phenylbut-1-en-3-yn-1-yl)-6-methyl-1,3,6,2-dioxazaborocane-4,8-dione (**4d**):

According's to the **general procedure GP1**, pale brown oil, 63 % yield. ^{1}H NMR (500 MHz, DMSO) δ 7.73 (d, *J* = 1 Hz, 1H), 7.60-7.58 (m, 2H), 7.42-7.41 (m, 3H), 6.73 (d, *J* = 3Hz, 1H), 6.58 (dd, *J* = 2 Hz, 1H), 6.38 (s, 1H), 4.32 (d, *J* = 17 Hz, 2H), 4.07 (d, *J* = 17 Hz, 2H), 2.87 (s, 3H). ^{13}C NMR (126 MHz, DMSO) δ 168.96, 152.74, 143.80, 131.59, 128.90, 128.46, 123.66, 122.01, 111.95, 108.75, 92.81, 86.22, 62.14, 47.16. ^{11}B NMR (160 MHz, DMSO) δ 10.27. HRMS (ESI) m/z cal. for $(M + H)^+$ 350.1194, found 350.1200.

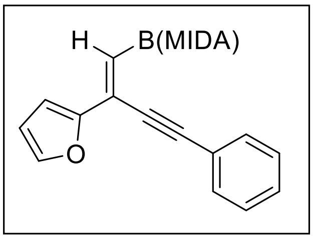

(E)-6-methyl-2-(4-phenyl-2-(thiophen-2-yl)but-1-en-3-yn-1-yl)-1,3,6,2-dioxazaborocane-4,8-dione (**4e**):

According's to the **general procedure GP1**, pale yellow semi solid, 68 % yield. ^{1}H NMR (500 MHz, DMSO) δ 7.59-7.57 (m, 2H), 7.54-7.53 (m, 1H), 7.45-7.42 (m, 4H), 7.07 (dd, *J* = 4 Hz, 1H), 6.34 (s, 1H), 4.33 (d, *J* = 17 Hz, 2H), 4.08 (d, *J* = 17 Hz, 2H), 2.89 (s, 3H). ^{13}C NMR (126 MHz, DMSO) δ 169.00, 144.66, 131.54, 128.98, 128.54, 127.97, 127.48, 126.60, 125.32, 121.98, 93.60, 87.59, 62.25, 47.25. ^{11}B NMR (160 MHz, DMSO) δ 10.06. HRMS (ESI) m/z cal. for $(M + H)^+$ 366.0966, found 366.0965.

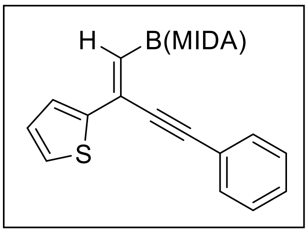

(*Z*)-2-(2-ethyl-4-phenylbut-1-en-3-yn-1-yl)-6-methyl-1,3,6,2-dioxazaborocane-4,8-dione (**4f**):

According's to the **general procedure GP1**, yellow solid, 79 % yield. ^{1}H NMR (500 MHz, DMSO) δ 7.48-7.46 (m, 2H), 7.39-7.37 (m, 3H), 5.73 (s, 1H), 4.29 (d, *J* = 17 Hz, 2H), 4.00 (d, *J* = 17 Hz, 2H), 2.84 (s, 3H), 2.34-2.30 (m, 2H), 1.14 (t, *J* = 7.5 Hz, 3H). ^{13}C NMR (126 MHz, DMSO) δ 169.00, 137.32, 131.40, 128.46, 128.42, 122.58, 93.08, 89.84, 62.15, 47.13, 33.55, 13.05. ^{11}B NMR (160 MHz, DMSO) δ 10.31. HRMS (ESI) m/z cal. for $(M + H)^+$ 312.1402, found 312.1390.

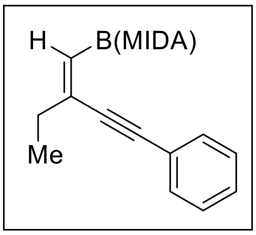

(*Z*)-2-(2-cyclopropyl-4-phenylbut-1-en-3-yn-1-yl)-6-methyl-1,3,6,2-dioxazaborocane-4,8-dione (**4g**):

According's to the **general procedure GP1**, pale yellow oil, 76 % yield. ^{1}H NMR (400 MHz, CDCl3) δ 7.46-7.45 (m, 2H), 7.35-7.33 (m, 3H), 5.90 (s, 1H), 3.87 (br. s, 4H), 2.93 (s, 3H), 1.29-1.27 (m, 1H), 0.88-0.87 (m, 2H), 0.79-0.77 (m, 2H). ^{13}C NMR (101 MHz, CDCl3) δ 167.40, 131.93, 131.86, 128.86, 128.49, 122.16, 99.99, 85.60, 62.88, 47.31, 24.93, 6.62. ^{11}B NMR (160 MHz, CDCl3) δ 10.54. HRMS (ESI) m/z cal. for $(M + H)^+$ 324.1402, found 324.1407.

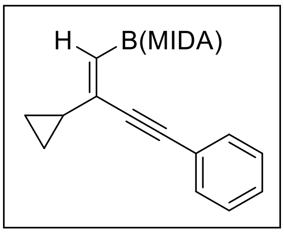

(*Z*)-2-(2-cyclohexyl-4-phenylbut-1-en-3-yn-1-yl)-6-methyl-1,3,6,2-dioxazaborocane-4,8-dione (**4h**):

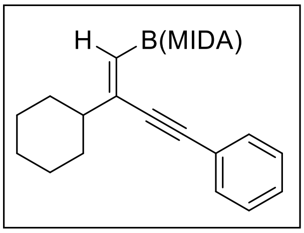

According's to the **general procedure GP1**, pale yellow oil, 72 % yield. ^{1}H NMR (400 MHz, DMSO) δ 7.46-7.38 (m, 5H), 5.71 (s, 1H), 4.28 (d, *J* = 17.2 Hz, 2H), 3.98 (d, *J* = 17.2 Hz, 2H), 2.81 (s, 3H), 2.18-2.17 (m, 1H), 1.77-1.67 (m, 5H), 1.40-1.18 (m, 5H). ^{13}C NMR (101 MHz, DMSO) δ 169.64, 131.89, 128.97, 128.96, 123.15, 123.13, 117.68, 62.72, 48.51, 47.71, 47.69, 32.21, 32.21, 32.20, 32.18, 32.17, 26.24, 26.20. ^{11}B NMR (160 MHz, DMSO) δ 10.05. HRMS (ESI) m/z cal. for $(M + NH_4)^+$ 383.2137, found 383.2154.

(*Z*)-2-(2-isopropyl-4-phenylbut-1-en-3-yn-1-yl)-6-methyl-1,3,6,2-dioxazaborocane-4,8-dione (**4i**):

According's to the **general procedure GP1**, yellow white solid, 81 % yield. ^{1}H NMR (500 MHz, DMSO) δ 7.47-7.45 (m, 2H), 7.39-7.38 (m, 3H), 5.73 (s, 1H), 4.28 (d, *J* = 17 Hz, 2H), 4.00 (d, *J* = 17 Hz, 2H), 2.82 (s, 3H), 2.56-2.52 (m, 1H), 1.13 (t, *J* = 7 Hz, 6H). ^{13}C NMR (126 MHz, DMSO) δ 169.58, 142.81, 131.93, 128.99, 123.15, 94.44, 89.16, 62.75, 47.70, 31.14, 22.19. ^{11}B NMR (160 MHz, DMSO) δ 10.51. HRMS (ESI) m/z cal. for $(M + H)^+$ 326.1558, found 326.1551.

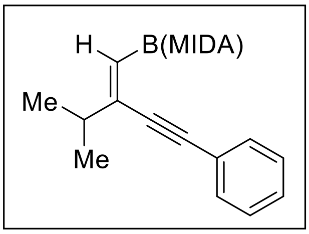

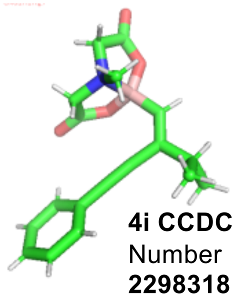

4i CCDC Number **2298318**

(*Z*)-6-methyl-2-(2-phenethyl-4-phenylbut-1-en-3-yn-1-yl)-1,3,6,2-dioxazaborocane-4,8-dione (**4j**):

According's to the **general procedure GP1**, yellow solid, 70 % yield. ^{1}H NMR (400 MHz, $CDCl_3$) δ 7.52-7.24 (m, 10H), 5.63 (s, 1H), 3.81 (d, *J* = 17.2 Hz, 2H), 3.67 (d, *J* = 17.2 Hz, 2H), 3.01 (m, 2H), 2.75-2.73 (m, 2H), 2.37 (s, 3H). ^{13}C NMR (126 MHz, $CDCl_3$) δ 167.48, 141.18, 137.42, 131.86, 128.78, 128.59, 128.50, 128.36, 125.99, 122.54, 95.27, 89.04, 62.58, 46.65, 42.59, 34.15. ^{11}B NMR (160 MHz, CDCl3) δ 10.65. HRMS (ESI) m/z cal. for $(M + NH_4)^+$ 405.1980, found 405.1984.

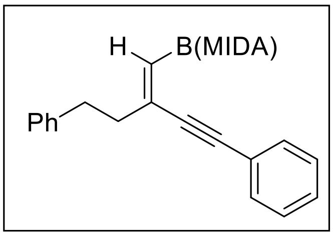

(*Z*)-6-methyl-2-(4-methyl-2-(phenylethynyl)pent-1-en-1-yl)-1,3,6,2-dioxazaborocane-4,8-dione (**4k**):

According's to the **general procedure GP1**, pale yellow oil, 82 % yield. ^{1}H NMR (400 MHz, DMSO)

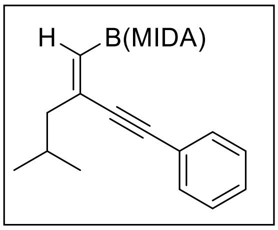

δ 7.45-7.37 (m, 5H), 5.70 (s, 1H), 4.29 (d, *J* = 18 Hz, 2H), 4.01 (*J* = 18 Hz, 2H), 2.83 (s, 3H), 2.18 (d, *J* = 6.8 Hz, 2H), 2.01-1.98 (m, 1H), 0.93 (d, *J* = 6 Hz, 6H). ^{13}C NMR (101 MHz, DMSO) δ 169.64, 135.55, 131.92, 129.02, 128.98, 123.07, 93.48, 90.48, 62.72, 50.58, 47.78, 27.25, 22.60. HRMS (ESI) m/z cal. for $(M + H)^+$ 340.1715, found 340.1745.

(*Z*)-2-(2-(4-methoxyphenyl)non-1-en-3-yn-1-yl)-6-methyl-1,3,6,2-dioxazaborocane-4,8-dione (**4l**):

According's to the **general procedure GP1**, colorless oil, 70 % yield. ^{1}H NMR (500 MHz, DMSO) δ

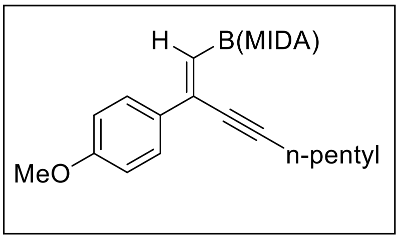

7.57 (d, *J* = 8.5 Hz, 2H), 6.89 (d, *J* = 8.5 Hz, 2H), 6.17 (s, 1H), 4.26 (d, *J* = 17 Hz, 2H), 3.93 (d, *J* = 17 Hz, 2H), 3.73 (s, 3H), 2.81 (s, 3H), 2.32 (t, *J* = 7.5 Hz, 2H), 1.52-1.47 (m, 2H), 1.35-1.24 (m, 4H), 0.86-0.84 (t, *J* = 7.5 Hz, 3H). ^{13}C NMR (126 MHz, DMSO) δ 168.99, 159.31, 133.95, 131.88, 127.02, 113.57, 96.18, 79.82, 62.55, 55.12, 47.22, 30.61, 27.54, 21.50, 18.91, 13.74. HRMS (ESI) m/z cal. for $(M + H)^+$ 384.1977, found 384.1985.

(*Z*)-2-(2-ethylnon-1-en-3-yn-1-yl)-6-methyl-1,3,6,2-dioxazaborocane-4,8-dione (**4m**):

According's to the **general procedure GP1**, colorless oil, 65 % yield. ^{1} H NMR (500 MHz, DMSO) δ

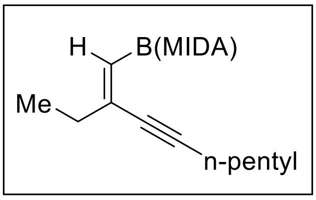

5.54 (s, 1H), 4.24 (d, *J* = 17 Hz, 2H), 3.88 (d, *J* = 17 Hz, 2H), 2.78 (s, 3H), 2.24 (t, *J* = 7 Hz, 2H), 2.15 (q, *J* = 7.5 Hz, 2H), 1.48-1.42 (m, 2H), 1.33-1.27 (m, 4H), 1.03 (t, *J* = 7.5 Hz, 3H), 0.87 (t, *J* = 7 Hz, 3H). ^{13}C NMR (126 MHz, DMSO) δ 174.23, 143.60, 99.94, 86.15, 67.67, 52.38, 38.91, 35.80, 32.85, 26.78, 24.08, 19.03, 18.23. HRMS (ESI) m/z cal. for $(M + H)^+$ 306.1871, found 306.1878.

(*Z*)-6-methyl-2-(2-(naphthalen-1-yl)-4-(trimethylsilyl)but-1-en-3-yn-1-yl)-1,3,6,2-dioxazaborocane-4,8-dione (**4n**):

According's to the **general procedure GP1**, colorless oil, 74 % yield. ^{1}H NMR (500 MHz, DMSO) δ

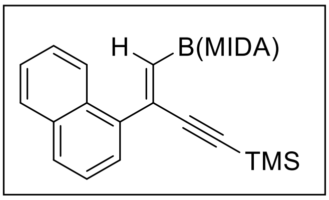

8.21 (d, *J* = 7.2 Hz, 1H), 7.95 (d, *J* = 8.4 Hz, 1H), 7.90 (d, *J* = 8.3 Hz, 1H), 7.56-7.45 (m, 4H), 6.14 (s, 1H), 4.39 (d, *J* = 17 Hz, 2H), 4.04 (d, *J* = 17 Hz, 2H), 3.04 (s, 3H), 0.11 (s, 9H). ^{13}C NMR (101 MHz, DMSO) δ 169.52, 140.21, 134.92, 133.73, 130.22, 128.80, 128.67, 126.53, 126.22, 126.08, 125.68, 105.79, 100.76, 63.15, 47.94, 0.06. HRMS (ESI) m/z cal. for $(M + H)^+$ 406.1640, found 406.1652.

(Z)-2-(4-(4-(tert-butyl)phenyl)-2-phenylbut-1-en-3-yn-1-yl)-6-methyl-1,3,6,2-dioxazaborocane-4,8-dione (**4o**):

According's to the **general procedure GP1**, white solid, 72 % yield. ^{1}H NMR (500 MHz, DMSO) δ 7.65 (d, *J* = 7.5 Hz, 2H), 7.39 (d, *J* = 8.5 Hz, 2H), 7.32-7.30 (m, 4H), 7.24 (t, *J* = 7.5 Hz, 1H), 6.36 (s, 1H), 4.23 (d, *J* = 17 Hz, 2H), 3.96 (d, *J* = 17 Hz, 2H), 2.79 (s, 3H), 1.17 (s, 9H). ^{13}C NMR (126 MHz, DMSO) δ 169.07, 151.60, 139.26, 133.88, 131.30, 128.44, 128.27, 125.90, 125.35, 119.37, 94.70, 88.15, 62.34, 47.21, 34.51, 30.83. HRMS (ESI) m/z cal. for (M + H)+ 416.2028, found 416.2045.

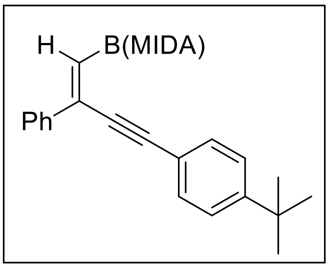

(*Z*)-2-(4-(4-chlorophenyl)-2-phenylbut-1-en-3-yn-1-yl)-6-methyl-1,3,6,2-dioxazaborocane-4,8-dione (**4p**):

According's to the **general procedure GP1**, pale yellow oil, 69 % yield. ^{1}H NMR (500 MHz, DMSO) δ 7.77 (d, *J* = 7.5 Hz, 2H), 7.60 (d, *J* = 8.5 Hz, 2H), 7.50 (d, *J* = 8.5 Hz, 2H), 7.44 (t, *J* = 7.5 Hz, 2H), 7.37 (t, *J* = 7.5 Hz, 1H), 6.54 (s, 1H), 4.35 (d, *J* = 17 Hz, 2H), 4.10 (d, *J* = 17 Hz, 2H), 2.91 (s, 3H). ^{13}C NMR (126 MHz, DMSO) δ 169.70, 139.50, 134.07, 133.92, 133.75, 129.22, 129.02, 128.89, 126.45, 121.78, 93.73, 90.21, 62.74, 47.72. HRMS (ESI) m/z cal. for $(M + H)^+$ 394.1012, found 394.1049.

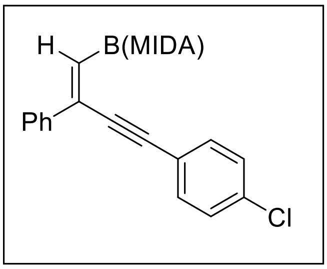

(*Z*)-2-(4-(4-methoxyphenyl)-2-phenylbut-1-en-3-yn-1-yl)-6-methyl-1,3,6,2-dioxazaborocane-4,8-dione (**4q**):

According's to the **general procedure GP1**, yellow gammy solid (*E/Z* = 05:95), 83 % yield. ^{1}H NMR (500 MHz, DMSO) δ 7.78 (d, *J* = 7.5 Hz, 2H), 7.52 (d, *J* = 8.5 Hz, 2H), 7.44 (d, *J* = 7.5 Hz, 2H), 7.36 (t, *J* = 7.5 Hz, 1H), 6.99 (d, *J* = 8.5 Hz, 2H), 6.44 (s, 1H), 4.35 (d, *J* = 17.5 Hz, 2H), 4.09 (d, *J* = 17.5 Hz, 2H), 3.80 (s, 3H), 2.91 (s, 3H). ^{13}C NMR (126 MHz, DMSO) δ 169.67, 160.17, 139.86, 134.52, 133.65, 128.96, 128.77, 126.44, 114.84, 114.74, 95.32, 88.03, 62.83, 55.77, 47.73. HRMS (ESI) m/z cal. for $(M + H)^+$ 407.1773, found 407.1780.

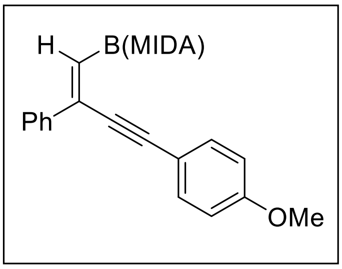

(*Z*)-6-methyl-2-(2-methyl-6-phenylhex-3-en-5-yn-3-yl)-1,3,6,2-dioxazaborocane-4,8-dione (**4r**):

According's to the **general procedure GP2**, brown oil, 62 % yield. ^{1}H NMR (400 MHz, $CDCl_3$) δ 7.43-7.39 (m, 5H), 5.91 (s, 1H), 4.28 (d, *J* = 17.6 Hz, 2H), 4.11 (d, *J* = 17.6 Hz, 2H), 2.82 (s, 3H), 2.77 (t, *J* = 6.8 Hz,1H), 1.24 (d, *J* = 6.8 Hz, 6H). ^{13}C NMR (101 MHz, $CDCl_3$) δ 170.18, 131.77, 129.79, 129.51, 123.99, 115.50, 96.87, 89.36, 63.14, 48.43, 32.23, 22.08. HRMS (ESI) m/z cal. for $(M + H)^+$ 326.1558, found 326.1550.

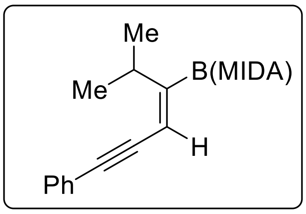

(*Z*)-2-(1,6-diphenylhex-3-en-5-yn-3-yl)-6-methyl-1,3,6,2-dioxazaborocane-4,8-dione (**4s**):

According's to the **general procedure GP2**, pale yellow, 71 % yield. ^{1}H NMR (500 MHz, $CDCl_3$) δ 7.49-7.47 (m, 2H), 7.37-7.36 (m, 2H), 7.30-7.17 (m, 6H), 6.22 (s, 1H), 3.88 (d, *J* = 16.5 Hz, 2H), 3.74 (d, *J* = 16.5 Hz, 2H), 2.91 (t, *J* = 8.5 Hz,2H), 2.81 (s, 3H), 2.61 (t, *J* = 8.5 Hz, 2H). ^{13}C NMR (101 MHz, $CDCl_3$) δ 167.49, 142.40, 131.56, 128.56, 128.47, 125.95, 123.30, 118.99, 96.60, 86.92, 62.02, 46.94, 35.84, 35.81. HRMS (ESI) m/z cal. for $(M + NH_4)^+$ 405.1980, found 405.1984.

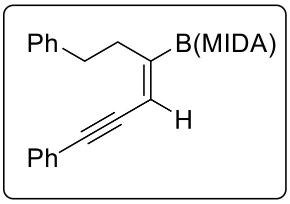

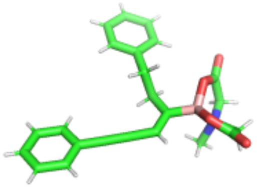

4s, CCDC Number **2298320**

(*E*)-6-methyl-2-(4-phenylbut-1-en-3-yn-1-yl)-1,3,6,2-dioxazaborocane-4,8-dione (**5a**):[4]

According's to the **general procedure GP2**, yellow solid, 73 % yield. ^{1}H NMR (500 MHz, DMSO) δ 7.46-7.45 (m, 2H), 7.40-7.39 (m, 3H), 6.28 (d, *J* = 18.5 Hz, 1H), 6.14 (d, *J* = 18.5 Hz, 1H), 4.26 (d, *J* = 17 Hz, 2H), 4.05 (d, *J* = 17 Hz, 2H), 2.82 (s, 3H). ^{13}C NMR (126 MHz, DMSO) δ 169.01, 131.27, 128.74, 128.69, 122.43, 120.70, 89.77, 61.51, 46.82. HRMS (ESI) m/z cal. for $(M + H)^+$ 284.1089, found 284.1044.

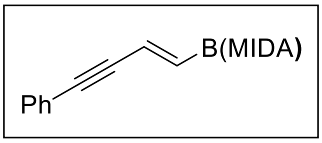

(*E*)-2-(4-(4-chlorophenyl)but-1-en-3-yn-1-yl)-6-methyl-1,3,6,2-dioxazaborocane-4,8-dione (**5b**):

According's to the **general procedure GP2**, yellow solid, 76 % yield. ^{1}H NMR (500 MHz, DMSO) δ 7.47 (m, 4H), 6.31 (d, *J* = 18.4 Hz, 1H), 6.13 (d, *J* = 18.4 Hz, 1H), 4.27 (d, *J* = 17.2 Hz, 2H), 4.05 (d, *J* = 17.2 Hz, 2H), 2.82 (s, 3H). ^{13}C NMR (126 MHz, DMSO) δ 168.86, 133.34, 133.03, 132.91, 128.81, 121.28, 120.42, 90.72, 88.48, 61.48, 46.78. HRMS (ESI) m/z cal. for $(M + H)^+$ 318.0699, found 318.0694.

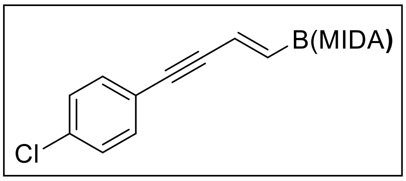

(*E*)-2-(4-(4-methoxyphenyl)but-1-en-3-yn-1-yl)-6-methyl-1,3,6,2-dioxazaborocane-4,8-dione (**5c**):

According's to the **general procedure GP2**, yellow solid, 80 % yield. ^{1}H NMR (400 MHz, DMSO) δ 7.38 (d, *J* = 6.4 Hz, 2H), 6.94 (d, *J* = 6.4 Hz, 2H), 6.16 (dt, *J* = 18, 8, 2H), 4.25 (d, *J* = 16.4 Hz, 2H), 4.04 (d, *J* = 16.8 Hz, 2H), 3.78 (s, 3H), 2.81 (s, 3H). ^{13}C NMR (126 MHz, DMSO) δ 168.87, 159.43, 132.76, 121.04, 114.39, 114.29, 89.99, 88.43, 61.45, 55.18, 46.77. HRMS (ESI) m/z cal. for $(M + H)^+$ 314.1194, found 314.1180.

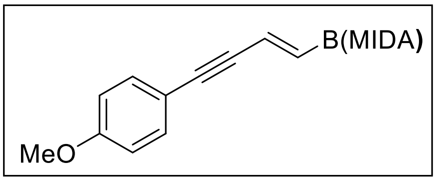

(*E*)-6-methyl-2-(4-(*p*-tolyl)but-1-en-3-yn-1-yl)-1,3,6,2-dioxazaborocane-4,8-dione (**5d**):

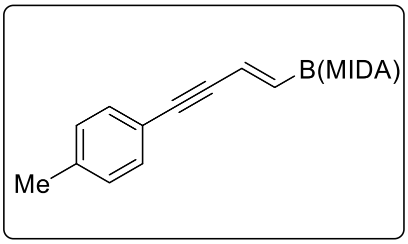

According's to the **general procedure GP2**, yellow solid, 74 % yield. ^{1}H NMR (400 MHz, DMSO) δ 7.32 (d, *J* = 8 Hz, 2H), 7.11 (d, *J* = 8 Hz, 2H), 6.33 (d, *J* = 18.4 Hz, 1H), 6.16 (d, *J* = 18.4 Hz, 1H), 4.03 (d, *J* = 17.2 Hz, 2H), 3.74 (d, *J* = 17.2 Hz, 2H), 2.87 (s, 3H), 2.33 (s, 3H). ^{13}C NMR (126 MHz, CDCl3) δ 167.76, 138.71, 131.57, 129.16, 124.17, 119.89, 91.61, 88.58, 61.65, 47.05, 21.45. HRMS (ESI) m/z cal. for $(M + NH_4)^+$ 315.1511, found 315.1520.

(*E*)-6-methyl-2-(4-(4-(trifluoromethyl)phenyl)but-1-en-3-yn-1-yl)-1,3,6,2-dioxazaborocane-4,8-dione (**5f**):

According's to the **general procedure GP2**, pale yellow oil, 69 % yield. ^{1}H NMR (400 MHz, DMSO) δ 7.75 (d, *J* = 8 Hz, 2H), 7.67 (d, *J* = 8 Hz, 2H), 6.39 (d, *J* = 18 Hz, 1H), 6.18 (d, *J* = 18 Hz, 1H), 4.28 (d, *J* = 17.2 Hz, 2H), 4.07 (d, *J* = 17.2 Hz, 2H), 2.84 (s, 3H). ^{13}C NMR (101 MHz, DMSO) δ 169.48, 132.52, 132.46, 132.43, 126.12, 126.05, 126.02, 125.97, 120.66, 120.63, 99.98, 92.64, 88.62, 62.06, 62.05, 62.00, 47.34, 47.30. ^{19}F NMR (471 MHz, DMSO) δ -61.38. ^{11}B NMR (160 MHz, DMSO) δ 10.31. HRMS (ESI) m/z cal. for $(M + H)^+$ 369.1228, found 369.1239.

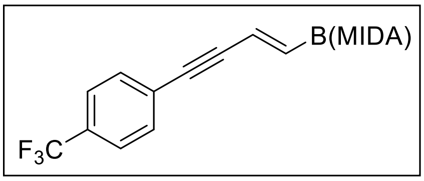

(*E*)-2-(4-(furan-2-yl)but-1-en-3-yn-1-yl)-6-methyl-1,3,6,2-dioxazaborocane-4,8-dione (**5g**):

According's to the **general procedure GP2**, brown oil, 62 % yield. ^{1}H NMR (400 MHz, $CDCl_3$) δ 6.76 (d, *J* = 18 Hz, 1H), 6.63 (d, *J* = 3.6 Hz, 1H), 6.29 (d, *J* = 3.2 Hz, 1H), 6.14 (d, *J* = 18 .4Hz, 1H), 4.02 (d, *J* = 16.8 Hz, 2H), 3.76 (d, *J* = 16.8 Hz, 2H), 3.46 (s, 1H), 2.88 (s, 3H). ^{13}C NMR (126 MHz, $CDCl_3$) δ 167.47, 154.12, 135.90, 130.68, 118.20, 110.37, 82.73, 74.14, 61.60, 46.95. HRMS (ESI) m/z cal. for $(M + H)^+$ 274.0887, found 274.0880.

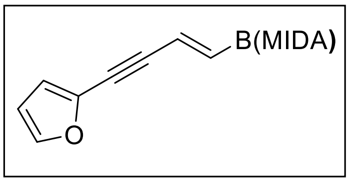

(*E*)-6-methyl-2-(4-(thiophen-2-yl)but-1-en-3-yn-1-yl)-1,3,6,2-dioxazaborocane-4,8-dione (**5h**):

According's to the **general procedure GP2**, yellow solid, 60 yield. ^{1}H NMR (500 MHz, DMSO) δ 7.27 (d, *J* = 13.5 Hz, 1H), 7.08 (d, *J* = 4 Hz, 1H), 6.93 (d, *J* = 18 Hz, 1H), 6.03 (d, *J* = 18 Hz, 1H), 4.58 (s, 1H), 4.25 (d, *J* = 17 Hz, 2H), 4.06 (d, *J* = 17 Hz, 2H), 2.81 (s, 3H). ^{13}C NMR (126 MHz, DMSO) δ 169.54, 146.23, 134.47, 133.84, 127.17, 120.53, 86.24, 77.54, 61.98, 47.28. HRMS (ESI) m/z cal. for $(M + H)^+$ 290.0653, found 290.0653.

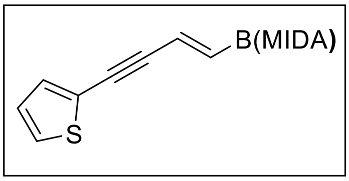

(E)-6-methyl-2-(4-(naphthalen-1-yl)but-1-en-3-yn-1-yl)-1,3,6,2-dioxazaborocane-4,8-dione (**5i**):

According's to the **general procedure GP2**, yellow solid, 68% yield. ^{1}H NMR (500 MHz, DMSO) δ 8.23 (d, *J* = 8 Hz, 1H), 7.71 (d, *J* = 8 Hz, 2H), 7.55 (d, *J* = 7 Hz, 1H), 7.42 (t, *J* = 7 Hz, 1H), 7.38 (t, *J* = 7.5 Hz, 1H), 7.18 (t, *J* = 8 Hz, 1H), 6.41 (d, *J* = 18 Hz, 1H), 6.23 (d, *J* = 18 Hz, 1H), 3.99 (d, *J* = 17 Hz, 2H), 3.68 (d, *J* = 17 Hz, 2H), 2.77 (s, 3H). ^{13}C NMR (126 MHz, CDCl3) δ 168.02, 133.16, 130.63, 129.03, 128.31, 126.95, 126.53, 126.09, 125.29, 124.03, 120.58, 94.10, 89.42, 77.29, 77.03, 76.78, 61.98, 61.74, 47.15. HRMS (ESI) m/z cal. for $(M + H)^+$ 334.1245, found 334.1246.

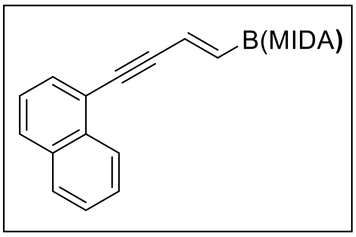

(*E*)-6-methyl-2-(non-1-en-3-yn-1-yl)-1,3,6,2-dioxazaborocane-4,8-dione (**5j**):[4]

According's to the **general procedure GP2**, yellow solid, 75 % yield. ^{1}H NMR (400 MHz, CDCl3) 6.04 (d, *J* = 18.4 Hz, 2H), 4.08 (d, *J* = 16.8 Hz, 2H), 3.74 (d, *J* = 16.8 Hz, 2H), 2.87 (s, 3H), 2.31 (t, *J* = 6 Hz, 2H), 1.56-1.52 (m, 2H), 1.36-1.33 (m, 4H), 0.93-0.90 (m, 3H). ^{13}C NMR (126 MHz, CDCl3) δ 168.34, 124.77, 93.03, 80.53, 61.69, 47.20, 31.20, 28.44, 22.26, 19.50, 14.02. HRMS (ESI) m/z cal. for $(M + H)^+$ 278.1558, found 278.1498.

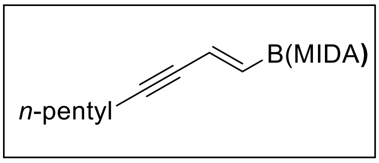

(*E*)-6-methyl-2-(4-(trimethylsilyl)but-1-en-3-yn-1-yl)-1,3,6,2-dioxazaborocane-4,8-dione (**5k**):[4]

According's to the **general procedure GP2**, yellow solid, 52 % yield. ^{1}H NMR (500 MHz, DMSO) δ 6.23 (d, *J* = 18.4 Hz, 1H), 5.91 (d, *J* = 18.4 Hz, 1H), 4.23 (d, *J* = 17.2 Hz, 2H), 4.02 (d, *J* = 17.2 Hz, 2H), 2.78 (s, 3H), 0.17 (s, 3H). ^{13}C NMR (126 MHz, CDCl3) δ 168.51, 120.46, 105.24, 94.28, 61.10, 46.39, -0.50. HRMS (ESI) m/z cal. for $(M + K)^+$ 318.0730, found 318.0869.

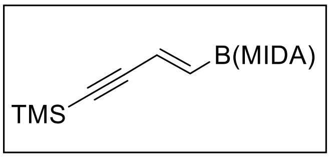

(*E*)-6-methyl-2-(6-phenylhex-1-en-3-yn-1-yl)-1,3,6,2-dioxazaborocane-4,8-dione (**5l**):

According's to the **general procedure GP2**, yellow solid, 70 % yield. ^{1}H NMR (500 MHz, DMSO) δ 7.33-7.23 (m, 5H), 6.09 (d, *J* = 18.4 Hz, 1H), 6.01 (d, *J* = 18.4 Hz, 1H), 4.06 (d, *J* = 17.2 Hz, 2H), 3.72 (d, *J* = 17.2 Hz, 2H), 2.81 (s, 3H), 2.66-2.60 (m, 2H), 1.28-1.24 (m, 2H). ^{13}C NMR (101 MHz,

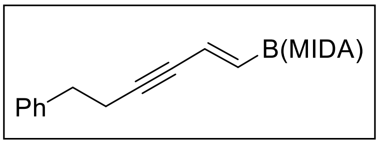

CDCl3) δ 168.39, 140.60, 128.49, 128.44, 126.37, 124.40, 91.93, 81.13, 61.59, 47.10, 34.98, 21.64. HRMS (ESI) m/z cal. for $(M + H)^+$ 312.1402, found 312.1410.

(*E*)-2-(4-(2-methoxyphenyl)but-1-en-3-yn-1-yl)-6-methyl-1,3,6,2-dioxazaborocane-4,8-dione (**5m**):[4]

According's to the **general procedure GP2**, brown solid, 75% yield. ^{1}H NMR (400 MHz, $CDCl_3$) δ

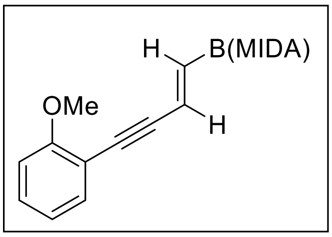

7.43 (d, *J* = 7.2 Hz, 1H), 7.34-7.29 (m, 1H), 6.95-6.89 (m, 2H), 6.42 (d, *J* = 18 .4 Hz, 1H), 6.21 (d, *J* = 18 .4 Hz, 1H), 3.97 (d, *J* = 16.8 Hz, 2H), 3.90 (s, 3H), 3.75 (d, *J* = 16.8 Hz, 2H), 2.90 (s, 3H). ^{13}C 13C NMR (126 MHz, CDCl3) δ 167.03, 159.99, 133.66, 130.05, 124.58, 120.55, 110.77, 93.05, 87.85, 61.55, 55.83, 46.83. ^{11}B NMR (160 MHz, CDCl3) δ 9.73. HRMS (ESI) m/z cal. for $(M + H)^+$ 314.1194, found 314.1180.

(*E*)-6-methyl-2-(4-(2-(methylthio)phenyl)but-1-en-3-yn-1-yl)-1,3,6,2-dioxazaborocane-4,8-dione (**5n**):

According's to the **general procedure GP2**, yellow solid, 70 % yield. ^{1}H NMR (500 MHz, $CDCl_3$) δ

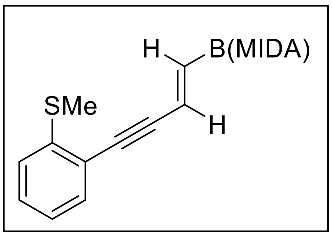

7.39 (d, *J* = 7.5 Hz, 1H), 7.30-7.28 (m, 1H), 7.14 (d, *J* = 7.5 Hz, 1H), 7.07 (t, *J* = 7.5 Hz, 1H), 6.39 (d, *J* = 18 Hz, 1H), 6.24 (d, *J* = 18 Hz, 1H), 3.99 (d, *J* = 17 Hz, 2H), 3.74 (d, *J* = 17 Hz, 2 H), 2.86 (s, 3H), 2.47 (s, 3H). ^{13}C NMR (126 MHz, $CDCl_3$) δ 167.65, 141.74, 132.51, 129.05, 124.33, 124.17, 123.88, 121.03, 95.46, 88.71, 61.66, 47.03, 15.04. ^{11}B NMR (160 MHz, CDCl3) δ 10.03. HRMS (ESI) m/z cal. for $(M + H)^+$ 347.1237, found 347.1235.

(*E*)-6-methyl-2-(4-(2-(trifluoromethyl)phenyl)but-1-en-3-yn-1-yl)-1,3,6,2-dioxazaborocane-4,8-dione (**5o**):

According's to the **general procedure GP2**, pale yellow oil, 73 % yield. ^{1}H NMR (400 MHz, DMSO)

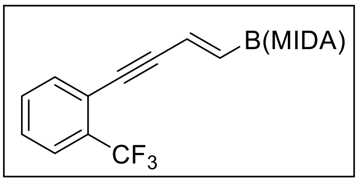

δ 7.80-7.58 (m, 4H), 6.38 (d, *J* = 18 Hz, 1H), 6.18 (d, *J* = 18 Hz, 1H), 4.28 (d, *J* = 18 Hz, 2H), 4.09 (d, *J* = 18 Hz, 2H), 2.84 (s, 3H). ^{13}C NMR (101 MHz, DMSO) δ 169.49, 134.41, 133.05, 130.32, 130.02, 129.42, 126.52, 120.68, 95.37, 85.76, 62.09, 47.32. ^{19}F NMR (471 MHz, DMSO) δ -61.01. ^{11}B NMR (160 MHz, DMSO) δ 10.06. HRMS (ESI) m/z cal. for $(M + NH_4)^+$ 369.1228, found 369.1235.

(*Z*)-4,4,5,5-tetramethyl-2-(1,4,6-triphenylhex-3-en-5-yn-3-yl)-1,3,2-dioxaborolane (**6a**):

According's to the **general procedure GP3**, colorless oil, 65 % yield. ^{1}H NMR (400 MHz, $CDCl_3$) δ 7.39 (d, *J* = 7 Hz, 2H), 7.35 (m, 2H), 7.25-7.19 (m, 10H), 7.13-7.09 (m, 1H), 2.88-2.84 (m, 2H), 2.81-2.78 (m, 2H), 1.10 (s, 12H). ^{13}C NMR (126 MHz, $CDCl_3$) δ 142.36, 140.78, 133.26, 131.60, 131.47,

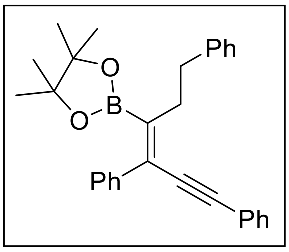

128.62, 128.57, 128.42, 128.29, 128.26, 128.17, 127.84, 127.62, 125.76, 123.65, 96.39, 88.93, 83.67, 37.64, 35.71, 24.65. ^{11}B NMR (160 MHz, CDCl3) δ 30.18. HRMS (ESI) m/z cal. for (M + H)$^+$ 435.2490, found 435.2484.

(*Z*)-2-(4-(4-fluorophenyl)-1,6-diphenylhex-3-en-5-yn-3-yl)-4,4,5,5-tetramethyl-1,3,2-dioxaborolane (**6b**):

According's to the **general procedure GP3**, colorless liquid, 72 % yield. ^{1}H NMR (500 MHz, CDCl3) δ 7.36-7.34 (m, 4H), 7.23-7.18 (m, 7H), 7.11-7.09 (m, 1H), 6.92 (t, *J* = 8.5 Hz, 2H), 2.87-2.84 (m, 2H), 2.80-2.76 (m, 2H), 1.10 (s, 12H). ^{13}C NMR (126 MHz, CDCl3) δ 162.55 (d, *J* = 246 Hz), 142.25, 136.84, 132.41, 131.59, 130.28 (d, *J* = 8 Hz), 128.62, 128.56, 128.30, 128.18, 125.80, 123.45, 114.61 (d, *J* = 21.8 Hz), 96.68, 88.77, 83.72, 37.60, 35.70, 24.65. ^{11}B NMR (160 MHz, CDCl3) δ 30.35. HRMS (ESI) m/z cal. for (M + H)$^+$ 453.2396, found 453.2400.

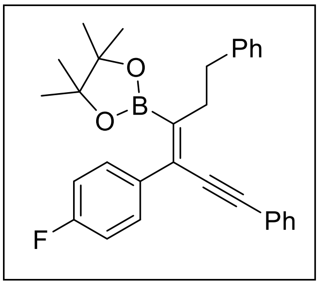

(*Z*)-2-(4-(4-methoxyphenyl)-1,6-diphenylhex-3-en-5-yn-3-yl)-4,4,5,5-tetramethyl-1,3,2-dioxaborolane (**6c**):

According's to the **general procedure GP3**, white yellow solid, 69% yield. ^{1}H NMR (400 MHz, CDCl3) δ 7.46-7.44 (m, 3H), 7.37-7.29 (m, 7H), 7.24-7.20 (m, 2H), 6.88 (d, *J* = 8.8 Hz, 2H), 3.85 (s, 3H), 2.94 (t, *J* = 4.8 Hz, 2H), 2.89 (t, *J* = 3.2 Hz, 2H), 1.23 (s, 12H). ^{13}C NMR (101 MHz, CDCl3) δ 159.36, 142.42, 133.34, 132.84, 131.60, 129.77, 128.62, 128.30, 128.29, 128.17, 125.75, 123.64, 113.24, 96.17, 89.08, 83.66, 55.36, 37.77, 35.82, 24.70. ^{11}B NMR (160 MHz, CDCl3) δ 30.20. HRMS (ESI) m/z cal. for (M + H)$^+$ 465.2596, found 465.2583.

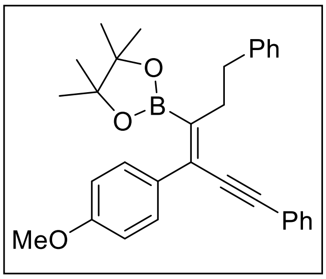

(*E*)-2-(4-(furan-2-yl)-1,6-diphenylhex-3-en-5-yn-3-yl)-4,4,5,5-tetramethyl-1,3,2-dioxaborolane (**6d**):

According's to the **general procedure GP3**, pale brown oil, 71 % yield. ^{1}H NMR (400 MHz, CDCl3) δ 7.67 (br. s, 1H), 7.52-7.51 (m, 2H), 7.44-7.43 (m, 3H), 7.29-7.17 (m, 5H), 6.59-6.55 (m, 2H), 2.78 (br. s, 4H), 1.32 (s, 12H). ^{13}C NMR (126 MHz, CDCl3) δ 152.01, 142.77, 141.24, 131.31, 128.92, 128.73, 128.26, 128.18, 125.86, 122.07, 118.54, 111.82, 108.54, 93.83, 84.97, 83.81, 36.70, 34.74, 24.61. ^{11}B NMR (160 MHz, CDCl3) δ 30.69. HRMS (ESI) m/z cal. for (M + H)$^+$ 425.2283, found 425.2290.

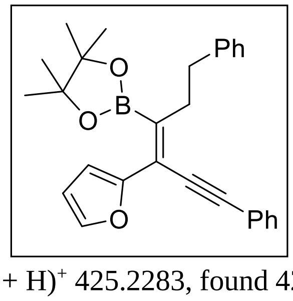

(*E*)-2-(1,6-diphenyl-4-(thiophen-2-yl)hex-3-en-5-yn-3-yl)-4,4,5,5-tetramethyl-1,3,2-dioxaborolane (**6e**):

According's to the **general procedure GP3**, pale yellow oil, 60 % yield. [1] H NMR (500 MHz, CDCl3) δ 7.37 (t, *J* = 3.5 Hz, 2H), 7.24-7.21 (m, 3H), 7.20-7.14 (m, 6H), 7.11-7.00 (m, 1H), 6.87 (t, *J* = 4 Hz, 1H), 2.84-2.82 (m, 2H), 2.79-2.77 (m, 2H), 1.17 (s, 12H). ^{13}C NMR (126 MHz, CDCl3) δ 143.52, 142.23, 131.62, 128.58, 128.36, 128.34, 128.32, 126.60, 126.52, 125.83, 125.52, 125.12, 123.37, 95.77, 88.32, 83.90, 37.80, 35.59, 24.79. ^{11}B NMR (160 MHz, CDCl3) δ 30.36. HRMS (ESI) m/z cal. for $(M + H)^+$ 441.2054, found 441.2045.

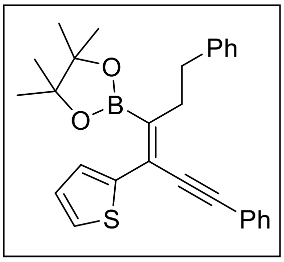

(*Z*)-2-(6-cyclopropyl-4-(4-methoxyphenyl)-1-phenylhex-3-en-5-yn-3-yl)-4,4,5,5-tetramethyl-1,3,2-dioxaborolane (**6f**):

According's to the **general procedure GP3**, colorless oil, 64 % yield. [1] H NMR (400 MHz, CDCl3) δ 7.37-7.25 (m, 6H), 7.24-7.16 (m, 1H), 6.85 (d, *J* = 8.5 Hz, 2H), 3.84 (s, 3H), 2.81 (s, 4H), 1.49-1.46 (m, 1H), 1.21 (s, 12H), 0.89-0.87 (m, 2H), 0.77-0.76 (m, 2H). ^{13}C NMR (126 MHz, CDCl3) δ 159.34, 142.77, 134.16, 133.58, 129.79, 128.64, 128.36, 125.76, 113.24, 101.10, 83.54, 55.43, 37.49, 35.83, 24.78, 9.10, 0.69. ^{11}B NMR (160 MHz, CDCl3) δ 30.35. HRMS (ESI) m/z cal. for $(M + H)^+$ 429.2596, found 429.2590.

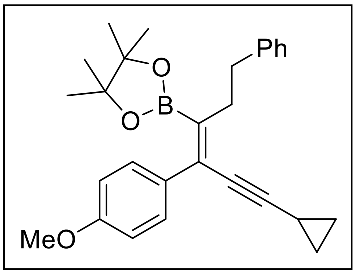

(*Z*)-2-(6-cyclopropyl-2-methyl-4-phenylhex-3-en-5-yn-3-yl)-4,4,5,5-tetramethyl-1,3,2-dioxaborolane (**6g**):

According's to the **general procedure GP3**, colorless oil, 75 % yield. [1] H NMR (400 MHz, CDCl3) δ 7.40 (d, *J* = 6.4 Hz, 2H), 7.28-7.25 (m, 3H), 3.23-3.19 (m, 1H), 1.43 (m, 1H), 1.28-1.12 (m, 18H), 0.85-.074 (m, 4H). ^{13}C NMR (126 MHz, CDCl3) δ 141.31, 128.95, 127.97, 127.24, 126.57, 99.51, 82.90, 33.77, 24.30, 21.48, 8.31, -0.00. ^{11}B NMR (160 MHz, CDCl3) δ 30.35. HRMS (ESI) m/z cal. for $(M + H)^+$ 337.2283, found 337.2279.

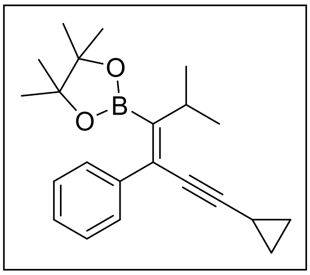

(*Z*)-2-(5-cyclopropyl-3-phenylpent-2-en-4-yn-2-yl)-4,4,5,5-tetramethyl-1,3,2-dioxaborolane (**6h**):

According's to the **general procedure GP3**, colorless oil, 69 % yield. ^{1}H NMR (400 MHz, CDCl3) δ 7.39-7.37 (m, 2H), 7.28-7.26 (m, 3H), 2.09 (s, 3H), 1.48-1.44 (m, 1H), 1.16 (s, 12H), 0.87-0.77 (m, 4H). ^{13}C NMR (126 MHz, CDCl3) δ 140.84, 132.97, 128.89, 127.88, 127.82, 127.10, 126.68, 101.24, 82.85, 23.96, 19.85, 8.48, -0.00. ^{11}B NMR (160 MHz, CDCl3) δ 30.18. HRMS (ESI) m/z cal. for $(M + Na)^+$ 331.1840, found 331.1736.

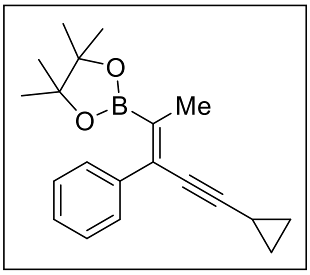

(*Z*)-trimethyl(3-(naphthalen-1-yl)-6-phenyl-4-(4,4,5,5-tetramethyl-1,3,2-dioxaborolan-2-yl)hex-3-en-1-yn-1-yl)silane (**6i**):

According's to the **general procedure GP3**, colorless oil, 64 % yield. ^{1}H NMR (500 MHz, CDCl3) δ 7.83-7.81 (m, 1H), 7.66-7.59 (m, 3H), 7.32-7.21 (m, 6H), 7.10-7.07 (m, 2H), 2.85-2.74 (m, 4H), 0.67 (s, 12H), 0.00 (s, 9H). ^{13}C NMR (126 MHz, CDCl3) δ 142.24, 138.43, 133.54, 131.99, 131.49, 128.78, 128.73, 128.31, 128.26, 127.80, 127.63, 126.61, 126.39, 125.83, 125.73, 125.56, 125.47, 125.07, 104.20, 101.96, 83.13, 36.61, 35.63, 24.26, -0.00. ^{11}B NMR (160 MHz, CDCl3) δ 30.11. HRMS (ESI) m/z cal. for $(M + K)^+$ 519.2287, found 519.2290.

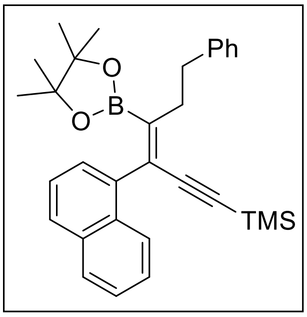

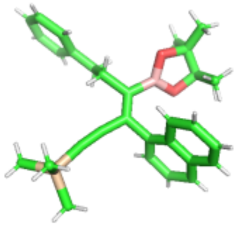

6i, CCDC Number 2298319

2-(1,1-difluoro-4-phenylbut-3-yn-2-yl)-6-methyl-1,3,6,2-dioxazaborocane-4,8-dione (**7a**):

According's to the **general procedure A**, colourless oil, 58% yield. ^{1}H NMR (500 MHz, DMSO) δ 7.36 (m, 5H), 6.12 (td, *J* = 56.5, 3 Hz, 1H), 4.45 (d, *J* = 17 Hz, 1H), 4.36 (d, *J* = 17 Hz, 1H), 4.10 (d, *J* = 17 Hz, 1H), 4.00 (d, *J* = 16 Hz, 1H), 3.11 (s, 3H), 3.06-2.98 (m, 1H). ^{13}C NMR (126 MHz, DMSO) δ 168.91, 168.65, 131.81, 129.06, 128.83, 123.07, 116.85, 84.23, 63.06, 62.71, 46.91. ^{19}F NMR (471 MHz, DMSO) δ -109.95 (d, *J* = 270 Hz), -114.46 (d, *J* = 270 Hz). ^{11}B NMR (160 MHz, DMSO) δ 10.20. HRMS (ESI) m/z cal. for $(M + NH_4)^+$ 339.1322, found 339.1332.

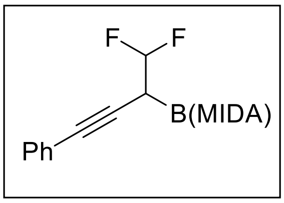

2-(4-(4-(tert-butyl)phenyl)-1,1-difluorobut-3-yn-2-yl)-6-methyl-1,3,6,2-dioxazaborocane-4,8-dione (**7b**):

According's to the **general procedure A**, colourless oil, 52% yield. ^{1}H NMR (400 MHz, DMSO) δ 7.38 (d, *J* = 7.2 Hz, 2H), 7.30 (d, *J* = 7.2 Hz, 2H), 6.12 (td, *J* = 53.6, 3 Hz, 1H), 4.46 (d, *J* = 17.2 Hz, 1H), 4.34 (d, *J* = 17.2 Hz, 1H), 4.10 (d, *J* = 17.2 Hz, 1H), 3.97 (d, *J* = 17.2 Hz, 1H), 3.10 (s, 3H), 3.04-2.95 (m, 1H), 1.27 (s, 9H). ^{13}C NMR (126 MHz, DMSO) δ 169.02, 168.68, 151.66, 131.64, 125.96, 120.19, 116.94, 84.36, 63.15, 62.78, 46.93, 35.02, 31.44. ^{19}F NMR (471 MHz, DMSO) δ -109.96 (d, *J* = 270 Hz), -114.46 (d, *J* = 270 Hz). ^{11}B NMR (160 MHz, DMSO) δ 10.24. HRMS (ESI) m/z cal. for $(M + NH_4)^+$ 395.1948, found 395.1964.

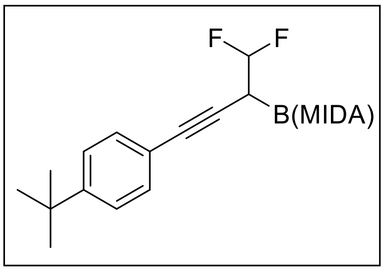

2-(4-(4-chlorophenyl)-1,1-difluorobut-3-yn-2-yl)-6-methyl-1,3,6,2-dioxazaborocane-4,8-dione (**7c**):

According's to the **general procedure A**, colourless oil, 62% yield. ^{1}H NMR (400 MHz, DMSO) δ 7.45-7.38 (m, 4H), 6.12 (td, *J* = 55.6, 2.8 Hz, 1H), 4.40 (d, *J* = 17.2 Hz, 1H), 4.35 (d, *J* = 17.2 Hz, 1H), 4.09 (d, *J* = 17.2 Hz, 1H), 4.00 (d, *J* = 17.2 Hz, 1H), 3.09 (s, 3H), 3.06-2.98 (m, 1H). ^{13}C NMR (126 MHz, DMSO) δ 173.60, 173.40, 138.31, 138.29, 133.95, 126.68, 87.88, 84.41, 84.14, 83.88, 67.78, 67.48, 51.68. ^{19}F NMR (471 MHz, DMSO) δ -109.91 (d, *J* = 270 Hz), -114.50 (d, *J* = 271 Hz). ^{11}B NMR (160 MHz, DMSO) δ 11.52. HRMS (ESI) m/z cal. for $(M + NH_4)^+$ 373.0932, found 373.0963.

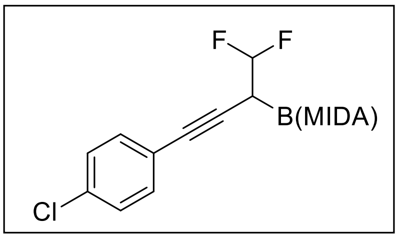

2-(4-(4-fluorophenyl)-1,1-difluorobut-3-yn-2-yl)-6-methyl-1,3,6,2-dioxazaborocane-4,8-dione (**7d**):

According's to the **general procedure A**, colourless oil, 57% yield. ^{1}H NMR (400 MHz, CDCl3) δ 7.39-7.34 (m, 2H), 7.04-7.00 (m, 2H), 6.12 (td, *J* = 56, 2.8 Hz, 1H), 4.15-3.90 (m, 4H), 3.22 (s, 3H), 2.83-2.74 (m, 1H). ^{13}C NMR (126 MHz, CDCl3) δ 166.25, 163.71, 137.49, 133.58 (d, *J* = 8.5 Hz), 130.22, 115.84 (d, *J* = 22 Hz), 100.00, 84.46, 62.93, 62.87, 46.02. ^{19}F NMR (471 MHz, CDCl3) δ -109.96 (s), 111.46 (d, *J* = 275 Hz), -113.77 (d, *J* = 195 Hz). ^{11}B NMR (160 MHz, DMSO) δ 10.26. HRMS (ESI) m/z cal. for $(M + NH_4)^+$ 357.1228, found 357.1234.

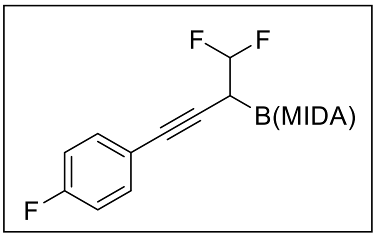

2-(1,1-difluorooct-3-yn-2-yl)-6-methyl-1,3,6,2-dioxazaborocane-4,8-dione (**7e**):

According's to the **general procedure A**, colourless oil, 60% yield. [1] H NMR (400 MHz, CDCl3) δ

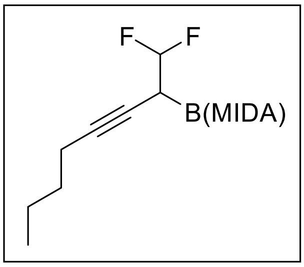

5.99 (td, J = 56, 2.8 Hz, 1H), 4.10-3.90 (m, 4H), 3.19 (s, 3H), 2.93-2.85 (m, 1H), 2.54-2.24 (m, 2H), 1.44-1.35 (m, 4H), 0.93 (t, J = 2.4 Hz, 3H). ^{13}C NMR (126 MHz, CDCl3) δ 167.44, 166.73, 116.17, 100.00, 85.87, 62.80, 62.71, 45.77, 45.76, 30.73, 21.96, 21.85, 18.39, 13.45, 13.44.^{19}F NMR (471 MHz, CDCl3) δ -111.89 (d, J = 273 Hz), -114.07 (d, J = 273 Hz). ^{11}B NMR (160 MHz, DMSO) δ 10.30. HRMS (ESI) m/z cal. for $(M + NH_4)^+$ 319.1635, found 319.1642.

2-(1,1-difluoro-4-(4-(trifluoromethyl)phenyl)but-3-yn-2-yl)-6-methyl-1,3,6,2-dioxazaborocane-4,8-dione (**7f**):

According's to the **general procedure A**, colourless oil, 56% yield. [1] H NMR (400 MHz, DMSO) δ

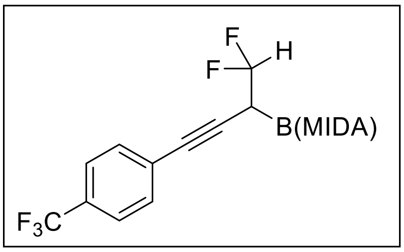

7.74 (d, J = 8.8 Hz, 2H), 7.59 (d, J = 8.8 Hz, 2H), 6.15 (td, J = 56, 3.6 Hz, 1H), 4.47-4.35 (m, 2H), 4.14-4.02 (m, 2H), 3.12 (s, 3H), 3.07-3.02 (m, 1H). ^{13}C NMR (101 MHz, DMSO) δ 168.92, 168.80, 132.59, 128.65, 127.38, 125.95, 125.91, 119.16, 116.75, 82.99, 63.00, 62.73, 46.99. ^{19}F NMR (471 MHz, DMSO) δ -61.93 (s) -111.09 (d, J = 273 Hz), -115.89 (d, J = 273 Hz). ^{11}B NMR (160 MHz, DMSO) δ 10.30. HRMS (ESI) m/z cal. for $(M + NH_4)^+$ 407.1196, found 407.1160.

2-(1,1-difluoro-4-(2-(trifluoromethyl)phenyl)but-3-yn-2-yl)-6-methyl-1,3,6,2-dioxazaborocane-4,8-dione (**7g**):

According's to the **general procedure A**, colourless oil, 42% yield. [1] H NMR (400 MHz, CDCl3) δ

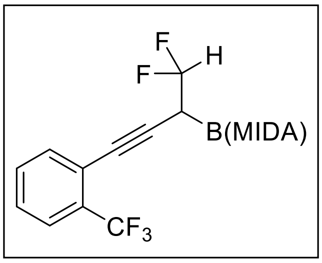

7.91-7.57 (m, 4H), 6.12 (td, J = 56, 3.2 Hz, 1H), 4.45-4.36 (m, 2H), 4.13-3.94 (m, 2H), 3.11 (s, 3H), 3.05-3.00 (m, 1H). ^{13}C NMR (101 MHz, DMSO) δ 168.76, 168.63, 134.78, 132.94, 129.07, 126.30, 80.01, 79.75, 79.43, 79.10, 79.09, 62.83, 62.64, 46.75. ^{19}F NMR (471 MHz, CDCl3) δ -60.93 (s), -110.09 (d, J = 272 Hz), -114.89 (d, J = 270 Hz). ^{11}B NMR (160 MHz, DMSO) δ 11.81. HRMS (ESI) m/z cal. for $(M + NH_4)^+$ 407.1196, found 407.1180.

2-(4,4-difluoro-1-phenylhex-1-yn-3-yl)-6-methyl-1,3,6,2-dioxazaborocane-4,8-dione (**7h**):

According's to the **general procedure A**, colourless oil, 53% yield .^{1}H NMR (400 MHz, DMSO) δ 7.37 (m, 5H), 4.44 (d, *J* = 17.2 Hz, 1H), 4.32 (d, *J* = 17.8 Hz, 1H), 4.09 (d, *J* = 17.2 Hz, 1H), 3.95 (d, *J* = 17.2 Hz, 1H), 3.12 (s, 3H), 3.01-2.98 (m, 1H), 2.20-1.99 (m, 2H), 1.02 (t, *J* = 7.2 Hz, 3H). ^{13}C NMR (101 MHz, DMSO) δ 169.16, 168.67, 131.71, 129.09, 128.86, 122.97, 83.95, 73.98, 63.05, 62.52, 46.66, 42.56, 25.41, 6.65. ^{19}F NMR (471 MHz, DMSO) δ -92.70. ^{11}B NMR (160 MHz, DMSO) δ 9.73. HRMS (ESI) m/z cal. for $(M + NH_4)^+$ 367.1635, found 367.1649.

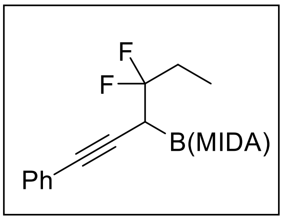

2-(4,4-difluoro-5-methyl-1-phenylhex-1-yn-3-yl)-6-methyl-1,3,6,2-dioxazaborocane-4,8-dione (**7i**): According's to the **general procedure A**, colourless oil, 59% yield .^{1}H NMR (400 MHz, DMSO) δ 7.37 (m, 5H), 4.43 (d, *J* = 17.2 Hz, 1H), 4.31 (d, *J* = 16.8 Hz, 1H), 4.09 (d, *J* = 17.2 Hz, 1H), 3.94 (d, *J* = 16.2 Hz, 1H), 3.13 (s, 3H), 3.06-2.96 (m, 1H), 2.47-2.41 (m, 1H), 1.09 (d, *J* = 6.8 Hz, 3H), 1.02 (d, *J* = 6.8 Hz, 3H). ^{13}C NMR (126 MHz, DMSO) δ 169.06, 168.61, 131.63, 129.06, 128.78, 126.55, 123.08, 87.19, 83.98, 79.42 (t, *J* = 33 Hz), 63.08, 62.59, 46.65, 34.33 (t, *J* = 25.9 Hz), 16.55 (q, *J* = 3.1 Hz), 15.94 (t, *J* = 4.9 Hz). ^{19}F NMR (471 MHz, DMSO) δ -97.69 (d, *J* = 249 Hz), -104.27 (d, *J* = 235 Hz). ^{11}B NMR (160 MHz, DMSO) δ 10.08. HRMS (ESI) m/z cal. for $(M + NH_4)^+$ 381.1792, found 381.1804.

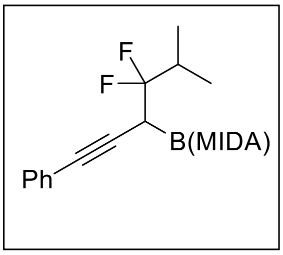

2-(4,4-difluoro-6-methyl-1-phenylhept-1-yn-3-yl)-6-methyl-1,3,6,2-dioxazaborocane-4,8-dione (**7j**):

According's to the **general procedure A**, colourless oil, 65% yield .^{1}H NMR (400 MHz, CDCl3) δ 7.39-7.29 (m, 5H), 4.01-3.88 (m, 4H), 3.19 (s, 3H), 2.87-2.80 (m, 1H), 2.12-2.08 (m, 2H), 1.30-1.26 (m, 1H), 1.02 (d, *J* = 6 Hz, 6H). ^{13}C NMR (126 MHz, CDCl3) δ 167.20, 166.49, 131.56, 130.12, 128.60, 128.55, 128.49, 128.46, 122.45, 84.97, 62.92, 62.87, 45.98, 44.35, 23.74, 23.40. ^{19}F NMR (471 MHz, CDCl3) δ -89.90 (m). ^{11}B NMR (160 MHz, DMSO) δ 10.04. HRMS (ESI) m/z cal. for $(M + NH_4)^+$ 395.1948, found 395.1961.

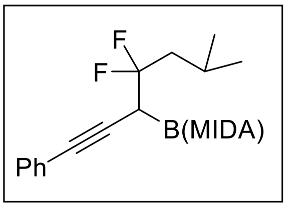

2-(3,3-difluoro-2-methyldec-5-yn-4-yl)-6-methyl-1,3,6,2-dioxazaborocane-4,8-dione (**7k**):

According's to the **general procedure A**, colourless oil, 60% yield. ^{1}H NMR (400 MHz, CDCl3) δ 4.03-3.89 (m, 4H), 3.20 (s, 3H), 3.01-2.88 (m, 1H), 2.55-2.44 (m, 1H), 2.16 (t, *J* = 6.8 Hz, 2H), 1.54-1.45 (m, 1H), 1.33-1.26 (m, 4H), 1.09-1.04 (m, 4H), 0.92-0.90 (m, 3H). ^{13}C NMR (101 MHz, CDCl3) δ 93.75, 62.85, 62.68, 45.96, 31.08, 28.35, 22.10, 18.68, 13.99, 13.99, 13.98. ^{19}F NMR (471 MHz, CDCl3) δ -98.58 (d, *J* = 240 Hz), -108.04 (d, *J* = 230 Hz). ^{11}B NMR (160 MHz, CDCl3) δ 10.08. HRMS (ESI) m/z cal. for $(M + NH_4)^+$ 361.2105, found 361.2061.

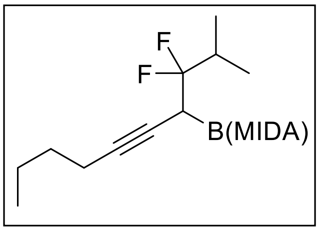

2-(1-cyclohexyl-1,1-difluoro-4-phenylbut-3-yn-2-yl)-6-methyl-1,3,6,2-dioxazaborocane-4,8-dione (**7l**):

According's to the **general procedure A**, white solid, 55% yield. ^{1}H NMR (400 MHz, DMSO) δ 7.37 (m, 5H), 4.41 (d, *J* = 17.6 Hz, 1H), 4.41 (d, *J* = 17.6 Hz, 1H), 4.04 (d, *J* = 17.6 Hz, 1H), 3.94 (d, *J* = 17.6 Hz, 1H), 3.11 (s, 3H), 3.02-2.89 (m, 1H), 2.13-1.64 (m, 6H), 1.22-1.07 (m, 5H). ^{13}C NMR (101 MHz, DMSO) δ 169.17, 168.74, 131.64, 129.16, 129.15, 129.12, 128.83, 123.04, 79.74, 79.41, 63.02, 62.50, 46.61, 25.89, 25.62, 25.60. ^{19}F NMR (471 MHz, DMSO) δ -97.16 (d, *J* = 227 Hz), -101.94 (d, *J* = 230 Hz). ^{11}B NMR (160 MHz, DMSO) δ 9.88. HRMS (ESI) m/z cal. for $(M + NH_4)^+$ 421.2105, found 421.2113.

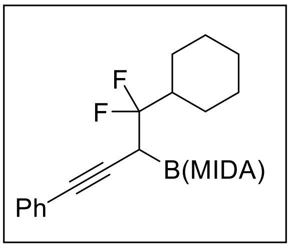

2-(1-cyclopropyl-1,1-difluoro-4-phenylbut-3-yn-2-yl)-6-methyl-1,3,6,2-dioxazaborocane-4,8-dione (**7m**):

According's to the **general procedure A**, colourless oil, 51% yield. ^{1}H NMR (400 MHz, DMSO) δ 7.31-7.19 (m, 5H), 3.97-3.79 (m, 4H), 3.13 (s, 3H), 2.87 (t, *J* = 10.2 Hz, 1H), 1.20-1.18 (m, 1H), 0.78-0.71 (m, 2H), 0.70-0.58 (m, 2H). ^{13}C NMR (101 MHz, DMSO) δ 169.23, 168.77, 131.68, 129.12, 128.83, 123.08, 63.04, 62.50, 46.64, 25.43, 2.23. ^{19}F NMR (471 MHz, CDCl3) δ -94.25 (d, *J* = 231 Hz), -96.79 (d, *J* = 233 Hz). ^{11}B NMR (160 MHz, DMSO) δ 10.20. HRMS (ESI) m/z cal. for $(M + NH_4)^+$ 379.1635, found 379.1644.

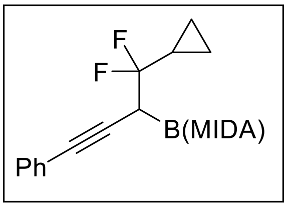

2-(4-iodo-5-phenylfuran-2-yl)-6-methyl-1,3,6,2-dioxazaboronane-4,9-dione (**9a**):

According's to the **general procedure B**, white solid, 61 % yield. ^{1}H NMR (400 MHz, DMSO) δ 7.96 (d, *J* = 8 Hz, 2H), 7.49 (t, *J* = 7.6 Hz, 2H), 7.42-7.38 (m, 1H), 6.86 (s, 1H), 4.39 (d, *J* = 17.2 Hz, 2H), 4.18 (d, *J* = 17.2 Hz, 2H), 2.74 (s, 3H). ^{13}C NMR (126 MHz, DMSO) δ 169.39, 154.15, 130.47, 129.09, 128.82, 128.68, 126.47, 63.51, 62.04, 47.81. ^{11}B NMR (160 MHz, DMSO) δ 9.66. HRMS (ESI) m/z cal. for $(M + Na)^+$ 461.9980, found 461.9989.

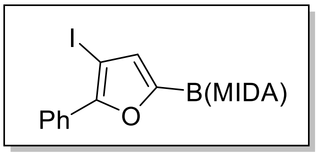

2-(4-iodo-5-(4-methoxyphenyl)furan-2-yl)-6-methyl-1,3,6,2-dioxazaborocane-4,8-dione (**9b**):

According's to the **general procedure B**, white solid, 65 % yield. ^{1}H NMR (400 MHz, DMSO) δ 7.88 (d, *J* = 8.8 Hz, 2H), 7.06 (d, *J* = 8.8 Hz, 2H), 6.82 (s, 1H), 4.39 (d, *J* = 17.2 Hz, 2H), 4.18 (d, *J* = 17.2 Hz, 2H), 3.81 (s, 3H), 2.74 (s, 3H). ^{13}C NMR (126 MHz, DMSO) δ 169.37, 159.75, 154.46, 128.42, 128.11, 123.15, 114.51, 62.02, 61.77, 55.74, 47.80. ^{11}B NMR (160 MHz, DMSO) δ 9.73. HRMS (ESI) m/z cal. for $(M + H)^+$ 456.0110, found 456.0112.

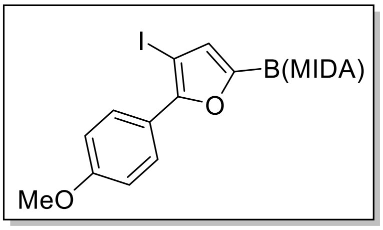

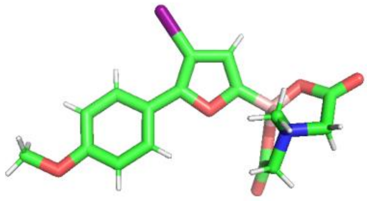

9b, CCDC Number **2298321**

2-(5-butyl-4-iodofuran-2-yl)-6-methyl-1,3,6,2-dioxazaborocane-4,8-dione (**9c**):

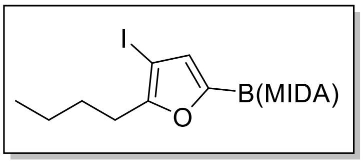

According's to the **general procedure B**, colorless oil, 69 % yield. ^{1}H NMR (400 MHz, $CDCl_3$) δ 6.77 (s, 1H), 4.06 (d, *J* = 16.4 Hz, 2H), 3.83 (t, *J* = 16.2 Hz, 2H), 6.68 (d, *J* = 3.2 Hz, 1H), 4.01 (d, *J* = 16.2 Hz, 1H), 3.92 (d, *J* = 16.2 Hz, 1H), 2.74 (s, 3H), 2.71-2.67 (m, 2H), 1.64-1.58 (m, 2H), 1.35-1.27 (m, 2H), 0.96-0.87 (m, 3H). ^{13}C NMR (101 MHz, CDCl3) δ 167.35, 160.81, 132.15, 126.60, 62.66, 61.62, 47.19, 31.21, 27.80, 22.18, 13.80. ^{11}B NMR (160 MHz, CDCl3) δ 10.24. HRMS (ESI) m/z cal. for $(M + H)^+$ 423.0583, found 423.0587.

2-(3-ethyl-4-iodo-5-phenylfuran-2-yl)-6-methyl-1,3,6,2-dioxazaborocane-4,8-dione (**9d**):

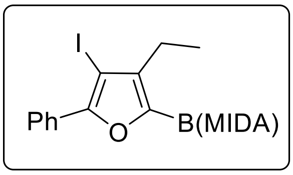

According's to the **general procedure B**, white solid, 75 % yield. ^{1}H NMR (400 MHz, $CDCl_3$) δ 7.95 (d, *J* = 6.8 Hz, 2H), 7.46-7.37 (m, 3H), 3.98 (d, *J* = 16.4 Hz, 2H), 3.88 (d, *J* = 16.4 Hz, 2H), 2.78 (s, 3H), 2.62-2.60 (m, 2H), 1.18 (t, *J* = 6.8 Hz, 3H).^{13}C NMR (101 MHz, CDCl3) δ 166.75, 154.42, 140.90, 130.34, 128.53, 128.46, 126.61, 69.47, 61.64, 46.92, 19.99, 15.77. ^{11}B NMR (160 MHz, CDCl3) δ 10.03. HRMS (ESI) m/z cal. for $(M + H)^+$ 454.0317, found 454.0316.

6-methyl-2-(5-phenylfuran-2-yl)-1,3,6,2-dioxazaboronane-4,9-dione (**10a**):[4]

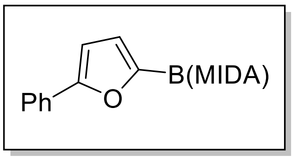

According's to the **general procedure C**, colorless oil, 74 % yield. ^{1}H NMR (400 MHz, $CDCl_3$) δ 7.69-7.67 (m, 2H), 7.42-7.38 (m, 2H), 7.31-7.28 (m, 1H), 6.88 (t, *J* = 3.2 Hz, 1H), 6.68 (d, *J* = 3.2 Hz, 1H), 4.01 (d, *J* = 16.2 Hz, 1H), 3.92 (d, *J* = 16.2 Hz, 1H), 2.79 (s, 3H). ^{13}C NMR (101 MHz, DMSO) δ 169.54, 156.45, 131.00, 129.26, 127.94, 124.20, 120.40, 106.53, 62.01, 61.94, 47.70.

2-(5-(4-methoxyphenyl)furan-2-yl)-6-methyl-1,3,6,2-dioxazaboronane-4,9-dione (**10b**):[4]

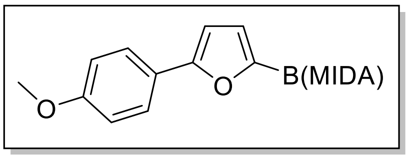

According's to the **general procedure C**, colorless oil, 80 % yield. ^{1}H NMR (400 MHz, DMSO) δ 7.66 (d, *J* = 8.8 Hz, 2H), 6.98 (d, *J* = 8.8 Hz, 2H), 6.74 (s, 1H), 6.68 (s, 1H), 4.37 (d, *J* = 17.2 Hz, 1H), 4.15 (d, *J* = 17.2 Hz, 1H), 3.77 (s, 3H), 2.68 (s, 3H). ^{13}C NMR (126 MHz, DMSO) δ 169.48, 159.27,

156.65, 125.76, 124.05, 120.36, 114.72, 104.79, 61.93, 55.67, 47.68. HRMS (ESI) m/z cal. for (M + H)$^+$ 344.1495, found 344.1462.

6-methyl-2-(5-pentylfuran-2-yl)-1,3,6,2-dioxazaboronane-4,9-dione (**10c**):[4]

According's to the **general procedure C**, colorless oil, 72 % yield. ^{1}H NMR (400 MHz, $CDCl_3$) δ 7.68 (d, *J* = 2.8 Hz, 2H), 5.99 (d, *J* = 2.8 Hz, 1H), 4.07 (d, *J* = 16.8 Hz, 2H), 3.83 (d, *J* = 16.8 Hz, 2H), 2.71 (s, 3H), 2.65-2.60 (m, 2H), 1.64-1.57 (m, 2H), 1.39-1.27 (m, 4H), 0.95-0.87 (m, 3H). ^{13}C NMR (101 MHz, $CDCl_3$) δ 167.83, 160.75, 119.96, 105.39, 61.55, 47.10, 31.40, 28.12, 27.85, 27.78, 22.38, 22.29, 13.82. HRMS (ESI) m/z cal. for (M + H)$^+$ 344.1495, found 344.1462.

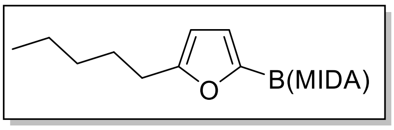

2-(3-ethyl-5-phenylfuran-2-yl)-6-methyl-1,3,6,2-dioxazaborocane-4,8-dione (**10d**):

According's to the **general procedure C**, colorless oil, 80 % yield. ^{1}H NMR (400 MHz, $CDCl_3$) δ 7.63 (d, *J* = 7.2 Hz, 2H), 7.39-7.36 (m, 2H), 7.29-7.26 (m, 1H), 6.60 (d, *J* = 2 Hz, 1H), 4.06 (d, *J* = 16.4 Hz, 2H), 3.88 (d, *J* = 16.4 Hz, 2H), 2.73 (s, 3H), 2.64-2.59 (m, 2H), 1.23-1.19 (m, 3H).^{13}C NMR (101 MHz, $CDCl_3$) δ 167.50, 156.44, 139.08, 130.70, 128.74, 127.65, 123.95, 107.23, 61.53, 46.84, 18.47, 15.69. ^{11}B NMR (160 MHz, CDCl3) δ 8.26. HRMS (ESI) m/z cal. for (M + H)$^+$ 328.1351, found 328.1259.

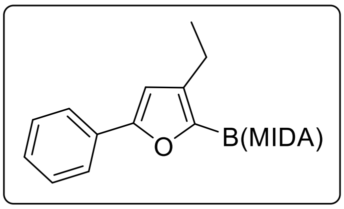

2-(5-(4-chlorophenyl)furan-2-yl)-6-methyl-1,3,6,2-dioxazaborocane-4,8-dione (**10e**):

According's to the **general procedure C**. colourless liquid, 70% yield. ^{1}H NMR (400 MHz, DMSO) δ 7.76 (d, *J* = 8.8 Hz, 2H), 7.48 (d, *J* = 8.8 Hz, 2H), 6.98-6.96 (m, 1H), 6.75-6.74 (m, 1H), 4.39 (d, *J* = 17.2 Hz, 2H), 4.17 (d, *J* = 17.2 Hz, 2H), 2.70 (s, 3H). ^{13}C NMR (101 MHz, DMSO) δ 169.51, 155.34, 132.29, 129.84, 129.30, 125.91, 124.21, 120.55, 107.31, 61.96, 47.71. HRMS (ESI) m/z cal. for (M + H)$^+$ 334.0648, found 334.0662.

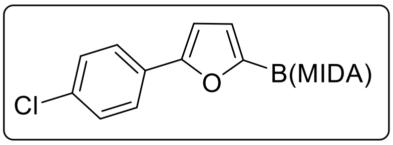

6-methyl-2-(5-phenyl-4-(phenylselanyl)furan-2-yl)-1,3,6,2-dioxazaborocane-4,8-dione (**11a**):

According's to the **general procedure D**, white solid, 67 % yield. ^{1}H NMR (400 MHz, DMSO) δ 7.92 (d, *J* = 7.6 Hz, 2H), 7.48-7.25 (m, 8H), 4.40 (d, *J* = 17.2 Hz, 2H), 4.20 (d, *J* = 17.2 Hz, 2H), 2.77 (s, 3H). ^{13}C NMR (101 MHz, DMSO) δ 169.46, 156.47, 131.66, 130.43, 130.10, 129.07, 128.85, 127.21, 126.70, 126.56, 103.57, 100.02, 62.10, 47.87. HRMS (ESI) m/z cal. for (M + H)$^+$ 356.0516, found 356.0531.

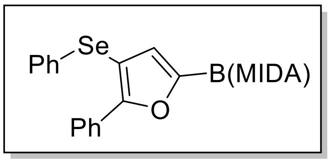

2-(3-ethyl-5-phenyl-4-(phenylselanyl)furan-2-yl)-6-methyl-1,3,6,2-dioxazaborocane-4,8-dione (**11b**):

According's to the **general procedure D**, white solid, 71 % yield. ^{1}H NMR (400 MHz, DMSO) δ 7.95 (d, *J* = 7.2 Hz, 2H), 7.42-7.17 (m, 8H), 4.43 (d, *J* = 17.2 Hz, 2H), 4.22 (d, *J* = 17.2 Hz, 2H), 2.75 (s, 3H), 2.51-2.50 (m, 2H), 0.96-0.95 (d, *J* = 7.2 Hz, 3H).^{13}C NMR (126 MHz, DMSO) δ 169.39, 157.96, 140.34, 132.75, 130.59, 130.01, 128.99, 128.94, 128.14, 126.80, 126.47, 105.07, 62.10, 47.73, 18.34, 16.60. ^{11}B NMR (160 MHz, DMSO) δ 9.58. HRMS (ESI) m/z cal. for $(M + NH_4)^+$ 501.1095, found 501.1249.

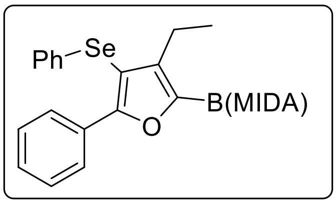

methyl(*E*)-3-(5-(6-methyl-4,8-dioxo-1,3,6,2-dioxazaborocan-2-yl)-2-phenylfuran-3-yl)acrylate (**9aa**):

Compound **9aa** was synthesized using general heck coupling procedure. **9a** (1 equiv), methyl acrylate (1.5 equiv), $Pd(OAc)_2$ (0.02 equiv), PPh_3 (0.15 equiv), Et_3N (2 equiv), DMF (0.1 M), 90 °C, 19 h. colourless oil, 63% yield. ^{1}H NMR (400 MHz, $CDCl_3$) δ 7.82 (d, *J* = 16 Hz, 1H), 7.68-7.65 (m, 2H), 7.55-7.53 (m, 1H), 7.48-7.45 (m, 2H), 7.07 (s, 1H), 6.25 (d, *J* = 15.5 Hz, 1H), 3.98 (d, *J* = 16.5 Hz, 2H), 3.86 (d, *J* = 16.5 Hz, 2H), 3.78 (s, 3H), 2.81 (s, 3H). ^{13}C NMR (101 MHz, $CDCl_3$) δ 167.55, 166.61, 135.50, 132.98, 132.16, 132.06, 131.97, 128.96, 128.59, 128.47, 127.47, 118.73, 118.49, 118.40, 61.71, 51.66, 47.19. ^{11}B NMR (160 MHz, CDCl3) δ 8.41. HRMS (ESI) m/z cal. for $(M + H)^+$ 384.1249, found 384.1255.

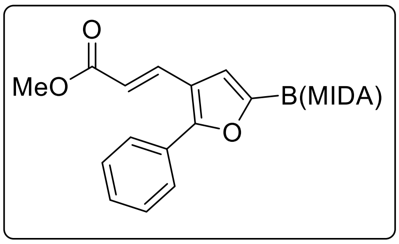

6-methyl-2-(5-phenyl-4-(phenylethynyl)furan-2-yl)-1,3,6,2-dioxazaborocane-4,8-dione (**9ab**):

Compound **9ab** was synthesized using general sonogashira coupling procedure. **9a** (1 equiv), phenyl acetylene (1.5 equiv), $Pd(PPh_3)_2Cl_2$ (0.1 equiv), CuI (0.05 equiv), Et_3N (3 equiv), THF (0.1 M), RT, 4 h. Colourless oil, 72% yield. ^{1}H NMR (400 MHz, $CDCl_3$) δ 8.11 (d, *J* = 7.5 Hz, 1H), 7.95 (d, *J* = 7.5 Hz, 1H), 7.70-7.53 (m, 2H), 7.46-7.34 (m, 5H), 7.27 (s, 1H), 6.97 (d, *J* = 3 Hz, 1H), 4.07-4.02 (m, 2H), 3.89-3.84 (m, 2H), 2.79 (s, 3H). ^{13}C NMR (126 MHz, $CDCl_3$) δ 166.83, 157.98, 155.38, 131.38, 130.24, 130.05, 129.62, 128.66, 128.60, 128.53, 128.48, 128.44, 128.34, 126.50, 125.11, 124.15, 103.63, 94.05, 82.48, 61.72, 61.69, 47.27. ^{11}B NMR (160 MHz, CDCl3) δ 8.60. HRMS (ESI) m/z cal. for $(M + NH_4)^+$ 417.1616, found 417.1619.

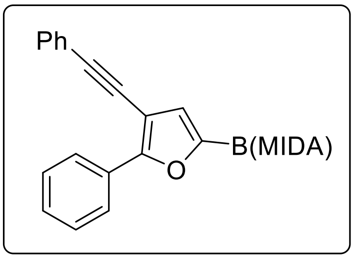

2-(3-iodo-4-phenyl-1,4-epoxynaphthalen-1(4H)-yl)-6-methyl-1,3,6,2-dioxazaborocane-4,8-dione (**9ac**):

Compound **9ac** was synthesized using general [4+2] cyclo addition procedure. **9a** (1 equiv), CsF (5 equiv), 2-(Trimethylsilyl)phenyl trifluoromethanesulfonate (3 equiv), ACN (0.1 M), 60 °C, 24 h. Colourless oil, 68% yield. ^{1}H NMR (400 MHz, $CDCl_3$) δ 7.73 (d, *J* = 7 Hz, 2H), 7.51-7.43 (m, 4H), 7.38 (d, *J* = 7 Hz, 2H), 7.04-6.97 (m, 2H), 3.92-3.83 (m, 4H), 3.02 (s, 3H). ^{13}C NMR (126 MHz, CDCl3) δ 166.75, 166.68, 153.28, 151.14, 148.56, 134.06, 128.69, 128.19, 127.82, 125.82, 124.72, 121.30, 121.13, 112.02, 96.45, 62.43, 62.38, 46.92. ^{11}B NMR (160 MHz, CDCl3) δ 8.24. HRMS (ESI) m/z cal. for $(M + H)^+$ 518.1339, found 518.1310.

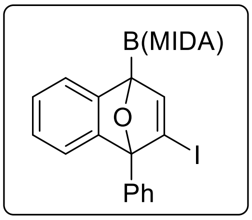

6-methyl-2-(5-phenyl-4-(p-tolyl)furan-2-yl)-1,3,6,2-dioxazaborocane-4,8-dione (**9ad**):

Compound **9ad** was synthesized using general Suzuki coupling procedure. **9a** (1 equiv), 4-methyl phenyl boronic acid (1.5 equiv), Cs_2CO_3 (3 equiv), $Pd(OAc)_2$ (0.1 equiv), XPhos (0.2 equiv), THF (0.1 M), RT for 24 h. Colourless oil, 75% yield. ^{1}H NMR (400 MHz, DMSO) δ 7.50 (d, *J* = 8.4 Hz, 2H), 7.37-7.36 (m, 2H), 7.30-7.28 (m, 3H), 7.21-7.19 (m, 2H), 6.81 (d, *J* = 3.6 Hz, 1H), 4.40 (d, *J* = 17.2 Hz, 2H), 4.19 (d, *J* = 17.2 Hz, 2H), 2.78 (s, 3H), 2.33 (s, 3H). ^{13}C NMR (126 MHz, DMSO) δ 169.43, 150.87, 136.84, 134.65, 131.52, 131.26, 129.78, 129.02, 128.54, 128.24, 126.53, 122.65, 122.35, 62.01, 47.81, 21.22. ^{11}B NMR (160MHz, DMSO) δ 8.63. HRMS (ESI) m/z cal. for $(M + H)^+$ 390.1507, found 390.1509.

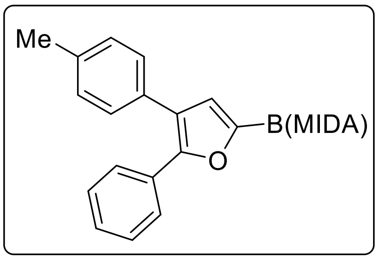

(*E*)-2-(2-(3-iodobenzofuran-2-yl)vinyl)-6-methyl-1,3,6,2-dioxazaborocane-4,8-dione (**12**):

In a reaction tube containing **5m** (1 equiv) in dry DCM (0.05 M) was cooled to -30 °C and treated with I_2 (1.2 equiv) in dry DCM (0.05). After the stirring for 3 h, the reaction mixture was quenched by using aq. $Na_2S_2O_3$ and extracted the crude mixture using DCM (2 times). Colourless oil, 76% yield. ^{1}H NMR (400 MHz, DMSO) δ 7.60-7.57 (m, 1H), 7.41-7.33 (m, 3H), 6.34 (d, *J* = 17.6 Hz, 1H), 5.99 (d, *J* = 17.6 Hz, 1H), 4.26 (d, *J* = 16.8 Hz, 2H), 4.08 (d, *J* = 16.8 Hz, 2H), 2.80 (s, 3H). ^{13}C NMR (126 MHz, CDCl3) δ 169.64, 154.38, 145.19, 144.80, 131.23, 126.67, 124.45, 121.43, 117.61, 111.67, 70.11, 62.06, 47.38. ^{11}B NMR (160 MHz, DMSO) δ 8.73. HRMS (ESI) m/z cal. for $(M + H)^+$ 535.3528, found 535.3538.

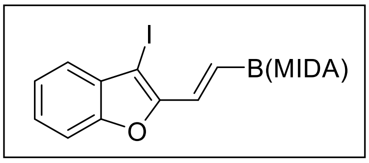

6-methyl-2-((1*E*,3*Z*)-4-phenyl-4-(4,4,5,5-tetramethyl-1,3,2-dioxaborolan-2-yl)buta-1,3-dien-1-yl)-1,3,6,2-dioxazaborocane-4,8-dione (**13a**):

According's to the **general procedure E**, colourless oil, 72% yield. ^{1}H NMR (500 MHz, DMSO) δ 7.43 (m, 2H), 7.31-7.18 (m, 4H), 6.35 (d, *J* = 18 Hz, 1H), 6.19 (d, *J* = 18 Hz, 1H), 3.96 (d, *J* = 15 Hz, 2H), 3.74 (d, *J* = 15 Hz, 1H), 2.88 (s, 3H), 1.27 (s, 12H). ^{13}C NMR (101 MHz, $CDCl_3$) δ 167.49, 131.68, 128.55, 128.40, 124.21, 122.91, 91.42, 89.02, 83.20, 61.71, 47.04, 24.57. ^{11}B NMR (160 MHz, DMSO) δ 22.53, 13.73. HRMS (ESI) m/z cal. for $(M + NH_4)^+$ 429.2363, found 429.2365.

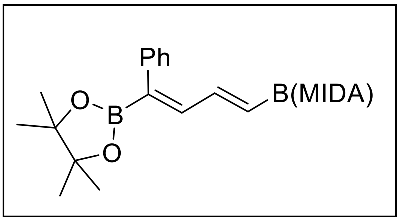

2-((1*E*,3*Z*)-4-(furan-2-yl)-4-(4,4,5,5-tetramethyl-1,3,2-dioxaborolan-2-yl)buta-1,3-dien-1-yl)-6-methyl-1,3,6,2-dioxazaborocane-4,8-dione (**13b**):

According's to the **general procedure E**, colourless oil, 76% yield. ^{1}H NMR (400 MHz, DMSO) δ 7.05 (d, *J* = 18.4 Hz, 1H), 6.69 (d, *J* = 3.6 Hz, 1H), 6.57-6.55 (m, 2H), 6.20 (d, *J* = 18.4 Hz, 1H), 6.90 (d, *J* = 18.4 Hz, 1H), 4.26 (d, *J* = 17.2 Hz, 2H), 4.06 (d, *J* = 17.2 Hz, 2H), 2.81 (s, 3H), 1.23 (s, 12H). ^{13}C NMR (126 MHz, DMSO) δ 169.54, 154.65, 152.73, 136.37, 129.23, 114.49, 111.68, 83.54, 81.83, 61.93, 47.26, 25.06, 24.95. HRMS (ESI) m/z cal. for $(M + H)^+$ 402.1890, found 402.1891. The stereochemistry of the structure has been elucidated by observing 2D NOESY NMR.

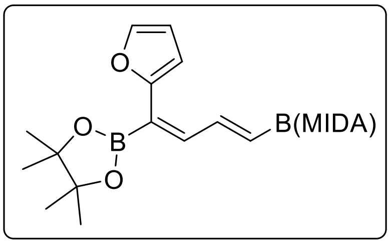

6-methyl-2-((1*E*,3*Z*)-4-(4,4,5,5-tetramethyl-1,3,2-dioxaborolan-2-yl)-4-(thiophen-2-yl)buta-1,3-dien-1-yl)-1,3,6,2-dioxazaborocane-4,8-dione (**13c**):

According's to the **general procedure E**, colourless oil, 69% yield. ^{1}H NMR (500 MHz, DMSO) δ 7.37 (d, *J* = 18 Hz, 1H), 7.21 (d, *J* = 3.2 Hz, 1H), 7.08 (d, *J* = 3.6 Hz, 1H), 6.92 (d, *J* = 18 Hz, 1H), 6.01 (d, *J* = 18 Hz, 1H), 5.71 (d, *J* = 18 Hz, 1H), 4.26 (d, *J* = 16.8 Hz, 2H), 4.06 (d, *J* = 16.8 Hz, 2H), 2.81 (s, 3H), 1.24 (s, 12H). ^{13}C NMR (101 MHz, DMSO) δ 169.58, 145.50, 142.39, 134.44, 130.48, 128.15, 83.60, 61.93, 47.24, 25.06. HRMS (ESI) m/z cal. for $(M + K)^+$ 456.1220, found 456.1363. The stereochemistry of the structure has been elucidated by observing 2D NOESY NMR.

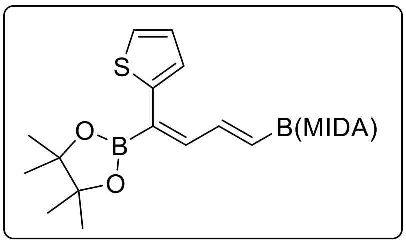

6-methyl-2-((1*E*,3*Z*)-4-phenyl-3,4-bis(4,4,5,5-tetramethyl-1,3,2-dioxaborolan-2-yl)buta-1,3-dien-1-yl)-1,3,6,2-dioxazaborocane-4,8-dione (**14a**):

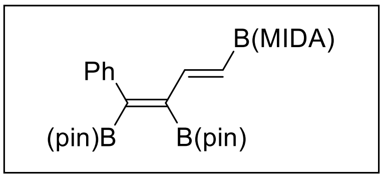

According's to the **general procedure F**, pale yellow solid, 75% yield. ^{1}H NMR (500 MHz, $CDCl_3$) δ 7.30 (t, *J* = 7.5 Hz, 2H), 7.21 (t, *J* = 7 Hz, 1H), 7.00 (d, *J* = 7 Hz, 2H), 6.53 (d, *J* = 18 Hz, 1H), 5.80 (d, *J* = 18 Hz, 1H), 4.14 (d, *J* = 17.5 Hz, 2H), 3.90 (d, *J* = 17 Hz, 2H), 2.72 (s, 3H), 1.32 (s, 8H), 1.20 (s, 8H), 1.16 (s, 4H), 1.07 (s, 4H). ^{13}C NMR (126 MHz, $CDCl_3$) δ 169.39, 142.61, 141.43, 129.31, 128.13, 126.74, 84.14, 84.04, 81.86, 74.05, 61.75, 47.14, 25.53,

25.38, 24.93, 24.87. ^{11}B NMR (160 MHz, $CDCl_3$) δ 22.23, 20.09, 11.30. HRMS (ESI) m/z cal. for $(M + H)^+$ 538.2949, found 538.2890.

2-((1*E*,3*Z*)-4-(3-chlorophenyl)-3,4-bis(4,4,5,5-tetramethyl-1,3,2-dioxaborolan-2-yl)buta-1,3-dien-1-yl)-6-methyl-1,3,6,2-dioxazaborocane-4,8-dione (**14b**):

According's to the **general procedure F**, colourless liquid, 63% yield. ^{1}H NMR (400 MHz, $CDCl_3$) δ 7.71-7.66 (m, 2H), 7.56 (d, *J* = 7.6 Hz, 1H), 7.51-7.29 (m, 1H), 6.42 (d, *J* = 18.2 Hz, 1H), 5.90 (d, *J* = 18.2 Hz, 1H), 3.87 (d, *J* = 16.4 Hz, 2H), 3.70 (d, *J* = 16.4 Hz, 2H), 2.92 (s, 3H), 1.29 (s, 12H), 1.28 (s, 12H). ^{13}C NMR (101 MHz, $CDCl_3$) δ 167.12, 132.14, 132.05, 131.98, 129.26, 129.11, 128.56, 128.48, 127.28, 126.70, 84.26, 84.21, 83.15, 61.95, 61.25, 45.68, 26.16, 25.27, 24.73, 24.57. HRMS (ESI) m/z cal. for $(M + H)^+$ 589.2835, found 589.2841.

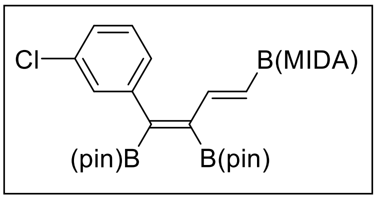

2-((1*E*,3*Z*)-4-(4-methoxyphenyl)-3,4-bis(4,4,5,5-tetramethyl-1,3,2-dioxaborolan-2-yl)buta-1,3-dien-1-yl)-6-methyl-1,3,6,2-dioxazaborocane-4,8-dione (**14c**):

According's to the **general procedure F**, colourless liquid, 79% yield. ^{1}H NMR (500 MHz, CDCl3) δ 7.04 (d, *J* = 8.6 Hz, 2H), 6.81 (d, *J* = 8.6 Hz, 2H), 6.76 (d, *J* = 18.5 Hz, 1H), 5.84 (d, *J* = 18.5 Hz, 1H), 3.88 (d, *J* = 16.5 Hz, 2H), 3.79 (s, 3H), 3.66 (d, *J* = 16.5 Hz, 2H), 2.88 (s, 3H), 1.37 (s, 9H), 1.27 (s, 9H), 1.23 (s, 6H). ^{13}C NMR (126 MHz, CDCl3) δ 167.62, 158.39, 145.08, 133.19, 132.15, 132.07, 130.47, 128.57, 128.47, 113.25, 84.03, 84.00, 83.14, 61.89, 61.21, 55.17, 46.56, 25.29, 24.78, 24.54. ^{11}B NMR (160 MHz, $CDCl_3$) δ 22.49, 12.03. HRMS (ESI) m/z cal. for $(M + H)^+$ 585.3320, found 585.3339.

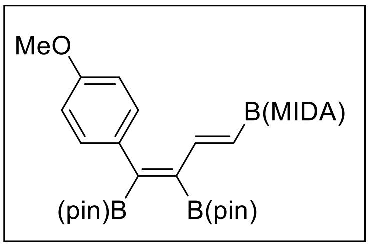

2-((1*E*,3*Z*)-3,4-bis(4,4,5,5-tetramethyl-1,3,2-dioxaborolan-2-yl)octa-1,3-dien-1-yl)-6-methyl-1,3,6,2-dioxazaborocane-4,8-dione (**14d**):

According's to the **general procedure F**, white solid, 81% yield. ^{1}H NMR (400 MHz, DMSO) δ 6.92 (d, *J* = 18 Hz, 1H), 5.72 (d, *J* = 18 Hz, 1H), 4.22 (d, *J* = 17.2 Hz, 2H), 3.98 (d, *J* = 17.2 Hz, 2H), 2.75 (s, 3H), 2.24 (br. s, 2H), 1.28 (s, 12H), 1.21 (s, 12H), 1.19-1.17 (m, 4H), 0.86 (m, 3H). ^{13}C NMR (126 MHz, DMSO) δ 169.58, 140.71, 131.99, 131.91, 129.26, 129.17, 83.74, 83.62, 61.86, 47.21, 32.30, 25.54, 25.11, 24.91, 22.47, 14.29. ^{11}B NMR (160 MHz, DMSO) δ 22.41, 9.07. HRMS (ESI) m/z cal. for $(M + H)^+$ 535.3528, found 535.3538.

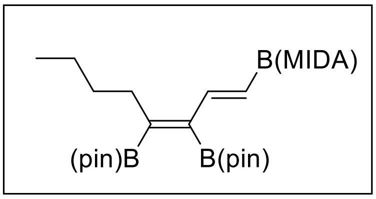

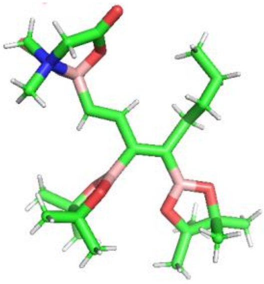

14d, **CCDC** Number **2298322**

2-((1E,3Z)-3,4-bis(4,4,5,5-tetramethyl-1,3,2-dioxaborolan-2-yl)-4-(trimethylsilyl)buta-1,3-dien-1-yl)-6-methyl-1,3,6,2-dioxazaborocane-4,8-dione (**14e**) :

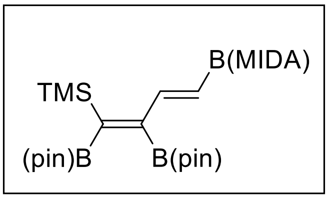

According's to the **general procedure F**, colourless oil, 69% yield. ^{1}H NMR (400 MHz, DMSO) δ 6.89 (d, *J* = 18 Hz, 1H), 5.81 (d, *J* = 18 Hz, 1H), 4.23 (d, *J* = 17.2 Hz, 2H), 3.96 (d, *J* = 17.2 Hz, 2H), 2.76 (s, 3H), 1.26 (s, 12H), 1.23 (s, 12H), 0.14 (s, 9H). ^{13}C NMR (101 MHz, DMSO) δ 169.55, 131.99, 131.89, 129.27, 129.15, 83.93, 83.60, 61.73, 47.07, 25.34, 25.25, 1.79. ^{11}B NMR (160 MHz, DMSO) δ 22.53, 13.73. HRMS (ESI) m/z cal. for $(M + H)^+$ 551.3297, found 551.3314.

2-((3Z,5Z)-1,6-diphenyl-5,6-bis(4,4,5,5-tetramethyl-1,3,2-dioxaborolan-2-yl)hexa-3,5-dien-3-yl)-6-methyl-1,3,6,2-dioxazaborocane-4,8-dione (**14f**):

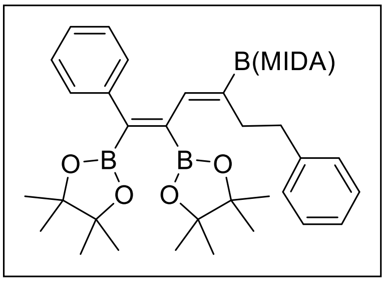

According's to the **general procedure F**, colourless oil, 75% yield. ^{1}H NMR (400 MHz, DMSO) δ 7.30-7.17 (m, 10H), 6.47 (s, 1H), 3.63 (d, *J* = 16.4 Hz, 2H), 3.44 (d, *J* = 16.4 Hz, 2H), 2.66-2.62 (m, 2H), 2.05 (s, 3H), 2.02-2.00 (m, 2H), 1.32 (s, 12H), 1.30 (s, 12H). ^{13}C NMR (126 MHz, CDCl3) δ 167.52, 143.46, 142.91, 140.89, 128.64, 128.42, 128.17, 127.88, 126.25, 125.47, 83.99, 83.92, 83.15, 61.22, 45.47, 35.39, 34.34, 24.91, 24.85, 24.56. ^{11}B NMR (160 MHz, DMSO) δ 22.53, 11.21. HRMS (ESI) m/z cal. for $(M + NH_4)^+$ 659.3841, found 659.3869.

6-methyl-2-(3-(phenylethynyl)oxiran-2-yl)-1,3,6,2-dioxazaborocane-4,8-dione (**15a**):[4]

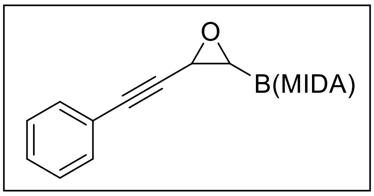

According's to the **general procedure G**, white pale white-yellow solid, 59% yield .^{1} H NMR (400 MHz, $CDCl_3$) δ 7.44 (dd, *J* = 5.4, 1.9 Hz, 2H), 7.33-7.28 (m, 3H), 4.06 (dd, *J* = 17.2, 7.2, Hz, 2H), 3.92 (d, *J* = 17.2 Hz, 1H), 3.81 (d, *J* = 17.2 Hz, 1H), 3.64 (d, *J* = 2.8 Hz, 1H), 3.15 (s, 3H), 2.64 (d, *J* = 2.8 Hz, 1H). ^{13}C NMR (126 MHz, $CDCl_3$) δ 168.10, 166.82, 131.91, 128.78, 128.33, 121.97, 86.31, 83.53, 62.17, 62.11, 46.25, 44.57.

2-(3-(hex-1-yn-1-yl)oxiran-2-yl)-6-methyl-1,3,6,2-dioxazaborocane-4,8-dione (**15b**):[4]

According's to the **general procedure G**, colourless oil, 59% yield. [1] H NMR (400 MHz, $CDCl_3$) δ

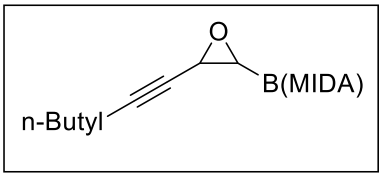

3.92 (m, 4H), 3.43 (q, *J* = 1.6 Hz, 1H), 3.15 (s, 3H), 2.48 (d, *J* = 2.8 Hz, 1H), 2.23 (t, *J* = 6.8 Hz, 2H), 1.53-1.27 (m, 4H), 0.92 (d, *J* = 7.6 Hz, 3H). ^{13}C NMR (126 MHz, $CDCl_3$) δ 167.62, 166.28, 84.87, 62.07, 61.99, 46.03, 44.74, 30.42, 21.89, 18.39, 13.52.

2-(6-methyl-4,8-dioxo-1,3,6,2-dioxazaborocan-2-yl)-2-(naphthalen-1-yl)-4-phenylbut-3-ynal (**16a**):

According's to the **general procedure G**, white solid, 56% yield. ^{1}H NMR (500 MHz, $CDCl_3$) δ 9.57

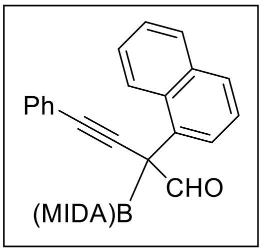

(s, 1H), 8.32 (d, *J* = 8.5 Hz, 1H), 7.92-7.75 (m, 4H), 7.52-7.40 (m, 4H), 7.37-7.32 (m, 2H), 7.32-7.23 (m, 3H), 3.95-3.77 (m, 4H), 3.07 (s, 3H). ^{13}C NMR (126 MHz, $CDCl_3$) δ 198.41, 166.71, 166.62, 134.72, 132.95, 132.11, 131.42, 130.60, 129.54, 129.32, 129.16, 128.88, 128.65, 126.08, 125.67, 125.41, 122.15, 90.39, 87.19, 63.51, 63.41, 47.22, 29.69. ^{11}B NMR (160 MHz, CDCl3) δ 10.82. HRMS (ESI) m/z cal. for $(M + NH_4)^+$ 443.1773 found 443.1791.

2-isopropyl-2-(6-methyl-4,8-dioxo-1,3,6,2-dioxazaborocan-2-yl)-4-phenylbut-3-ynal (**16b**):

According's to the **general procedure G**, white solid, 55% yield. ^{1}H NMR (400 MHz, $CDCl_3$) δ 9.72

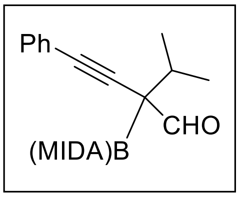

(s, 1H), 7.42-7.39 (m, 2H), 7.32-7.30 (m, 3H), 4.04 -3.95 (m, 3H), 3.79 (d, *J* = 16.9 Hz, 1H), 3.04 (s, 3H), 2.58 (m, 1H), 1.24 (d, *J* = 5.88 Hz, 3H), 1.15 (d, *J* = 6.8 Hz, 3H). ^{13}C NMR (126 MHz, $CDCl_3$) δ 203.49, 166.58, 166.31, 133.66, 131.53, 130.19, 128.55, 122.61, 88.37, 86.87, 62.95, 46.53, 33.40, 20.13, 19.94. ^{11}B NMR (160 MHz, CDCl3) δ 10.27. HRMS (ESI) m/z cal. for $(M + NH_4)^+$ 359.1773 found 359.1767.

2-isopropyl-2-(6-methyl-4,8-dioxo-1,3,6,2-dioxazaborocan-2-yl)-4-(p-tolyl)but-3-ynal (**16c**):

According's to the **general procedure G**, white solid, 60% yield. ^{1}H NMR (400 MHz, $CDCl_3$) δ 9.73

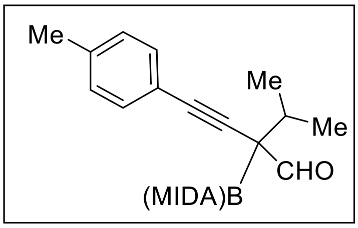

(s, 1H), 7.29 (t, *J* = 7.84 Hz, 2H), 7.14 (d, *J* = 7.8 Hz, 2H), 4.05 (d, *J* = 15.9 Hz, 1H), 3.88-3.83 (m, 2H), 3.75 (d, *J* = 16.7 Hz, 1H), 3.05 (s, 3H), 2.64-2.57 (m, 1H), 2.36 (s, 3H), 1.25 (d, *J* = 6.8 Hz, 3H), 1.19 (d, *J* = 6.8 Hz, 3H). ^{13}C NMR (101 MHz, $CDCl_3$) δ 203.60, 166.65, 166.31, 138.76, 131.43, 129.40, 129.31, 119.45, 88.42, 86.02, 62.90, 62.87, 46.47, 33.40, 21.47, 20.16, 19.97. ^{11}B NMR (160 MHz, CDCl3) δ 10.89. HRMS (ESI) m/z cal. for $(M + NH_4)^+$ 373.1929 found 373.1947.

2-ethyl-2-(6-methyl-4,8-dioxo-1,3,6,2-dioxazaborocan-2-yl)non-3-ynal (**16d**):

According's to the **general procedure G**, white solid, 80% yield. ^{1}H NMR (500 MHz, DMSO) δ 9.46 (s, 1H), 4.40 (d, J = 15 Hz, 1H), 4.29 (d, J = 15 Hz, 1H), 3.94 (d, J = 17.0 Hz, 2H), 2.99 (s, 3H), 2.22 (t, J = 14 Hz, 2H), 2.09-2.02 (m, 2H), 1.66-1.73 (m, 1H), 1.46-1.40 (m, 2H), 1.36-1.24 (m, 4H), 0.88-0.82 (m, 6H). 13C NMR (126 MHz, DMSO) δ 202.54, 168.63, 168.14, 87.53, 78.05, 63.25, 63.20, 46.70, 30.86, 28.43, 24.33, 22.02, 18.57, 14.27, 10.59. ^{11}B NMR (160 MHz, DMSO) δ 10.87. HRMS (ESI) m/z cal. for $(M + NH_4)^+$ 339.2086 found 339.2077.

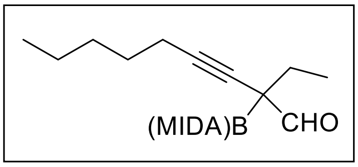

F. Crystallographic Data and Structure Refinements:

Good quality single crystals of each compound were sorted out with the help of a polarizing microscope and immersed in paratone oil, which was then mounted on the tip of glass fiber and cemented using epoxy resin. The single-crystal XRD diffraction data were collected at 298 K on a Bruker AXS (D8 Quest System) X-ray diffractometer, equipped with a PHOTON 100 CMOS detector using graphite-monochromated Mo-K_α radiation (0.71073 Å). The linear absorption coefficients, scattering factors for the atoms, and the anomalous dispersion corrections were taken from International Tables for X-ray Crystallography. Bruker Apex III software was used for data collection, unit cell measurements, absorption corrections, scaling, and integration.[5] The data were reduced and an empirical absorption correction was applied with the help of SAINTPLUS software and SADABS programs using XPREP, respectively.[6] The structures were solved by the direct method using SHELXL-2014 in the WinGx programs. The WinGx package of programs was used to carry out the full-matrix least-squares refinement against the function $|F^2|$.[7] For all the cases, non-hydrogen atoms were refined anisotropically. All other hydrogen atoms were geometrically fixed using the riding atom model and assigned fixed isotropic displacement parameters. The "ACTA" command was used to generate the Crystallographic Information File (CIF). The structural details of all the compounds are presented in **Table S2**. CCDC: 2298318, 2298319, 2298320, 2298321, 2298322 contain the crystallographic data of these compounds 4g, 6i, 4p, 7b, 21d respectively. These data are available from The Cambridge Crystallographic Data Center (CCDC) *via* www.ccdc.cam.ac.uk/data_request/cif.

Table S2: Crystal data and structure refinements for the molecules **4g**, **6i**, **4p**, **7b** and **21d**.

Entry	**4i**	**6i**	**4s**	**9b**	**14d**
Empirical formula	$C_{18} H_{20} B N O_4$	$C_{31} H_{37} B O_2 Si$	$C_{11.50} H_{11} B_{0.5} N_{0.5} O_2$	$C_{16} H_{15} B I N O_6$	$C_{25} H_{42} B_3 N O_8$
Formula weight	325.16	480.50	193.61	455.00	517.02
Temperature (K)	296(2)	296(2)	296(2)	296(2)	296(2)
Radiation	*Mo* (k_α)	*Mo* (k_α)	*Mo* (k_α)	*Mo* (k_α)	*Mo* (k_α)
Wave length (λ)	0.71073	0.71073	0.71073	0.71073	0.71073
Crystal system	orthorhombic	triclinic	orthorhombic	monoclinic	orthorhombic

Space group	*Pbcn*	$P\bar{1}$	*Ccc*2	$P2_1/c$	*Pbca*
a [Å]	10.617(1)	10.562(6)	11.125(7)	19.623(4)	13.492(5)
b [Å]	12.426(1)	12.806(9)	35.01(2)	6.695(2)	16.205(6)
c [Å]	26.89(4)	13.142(1)	10.586(7)	13.524(3)	27.144(1)
α [°]	90	61.44(3)	90	89.84(3)	90
β [°]	90	71.55(3)	90	74.91(3)	90
γ [°]	90	72.89(3)	90	89.96(3)	90
Volume [$Å^3$]	3548(1)	1459.1(1)	4123(5)	1715.4(8)	5935(4)
Z	8	2	16	4	8
Density (calculated) [$Mg\ m^{-3}$]	1.217	1.094	1.248	1.762	1.157
Absorption coefficient [mm^{-1}]	0.085	0.104	0.084	1.899	0.083
F (000)	1376	516	1632	896	2224
Refl. used [$I > 2\sigma(I)$]	7789	6456	6895	4703	19938
Independent reflections	1606	1967	2597	2169	4625
R_{int}	0.2360	0.1464	0.1563	0.0992	0.2157
Refinement method	full-matrix least squares on F^2	full-matrix least squares on F^2	full-matrix least squares on F^2	full-matrix least squares on F^2	full-matrix least squares on F^2
GOF	0.888	1.038	0.943	0.998	1.228
Final *R* indices[I>2σ(I)]	R_1= 0.0954 wR_2= 0.2111	R_1= 0.1287 wR_2= 0.3592	R_1= 0.0643 wR_2= 0.1207	R_1= 0.0496 wR_2= 0.0847	R_1= 0.1592 wR_2= 0.4519
R indices (all data)	R_1= 0.3803 wR_2= 0.3293	R_1= 0.2941 wR_2= 0.4548	R_1= 0.2312 wR_2= 0.1640	R_1= 0.1613 wR_2= 0.1078	R_1= 0.3775 wR_2= 0.5469
CCDC	**2298318**	**2298319**	**2298320**	**2298321**	**2298322**

G. Reference

1. C. Hwang, W. Jo and S. H. Cho, *Chem. Commun*. 2017, **53**, 7573-7576.
2. H. Li, X. Shangguan, Z. Zhang, S. Huang, Y. Zhang and J. Wang, *Org. Lett*. 2014, **16**, 448-451.
3. C. Liu, X. Zhang, R. Wang and W. Wang, *Org. Lett*. 2010, **12**, 4948-4951.
4. R. K. Shiroodi, O. Koleda and V. Gevorgyan, *J. Am. Chem. Soc.* 2014, **136**, 13146−13149.
5. (a) Sheldrick, G. M. Siemens Area Correction Absorption Correction Program; University of Göttingen: Göttingen, Germany, **1994**; (b) Farrugia; L. J., WinGx suite for small-molecule single crystal crystallography. *J. Appl. Crystallogr.* **1999**, *32*, 837.
6. (a) SAINT+, 6.02ed, Bruker AXS, Madison, WI, **1999**; (b) XPREP, 5.1 ed. Siemens Industrial Automation Inc., Madison, WI, **1995**,

7. (a) Sheldrick; G. M., SHELXL-97 Program for Crystal Structure Solution and Refinement; University of Göttingen: Göttingen, Germany, **1997**; (b) G. M. Sheldrick, Crystal Structure Refinement with SHELXL. *Acta Cryst C* **2015**, *71*, 3–8.

➢ ^{1}H-NMR & ^{13}C-NMR Spectra:

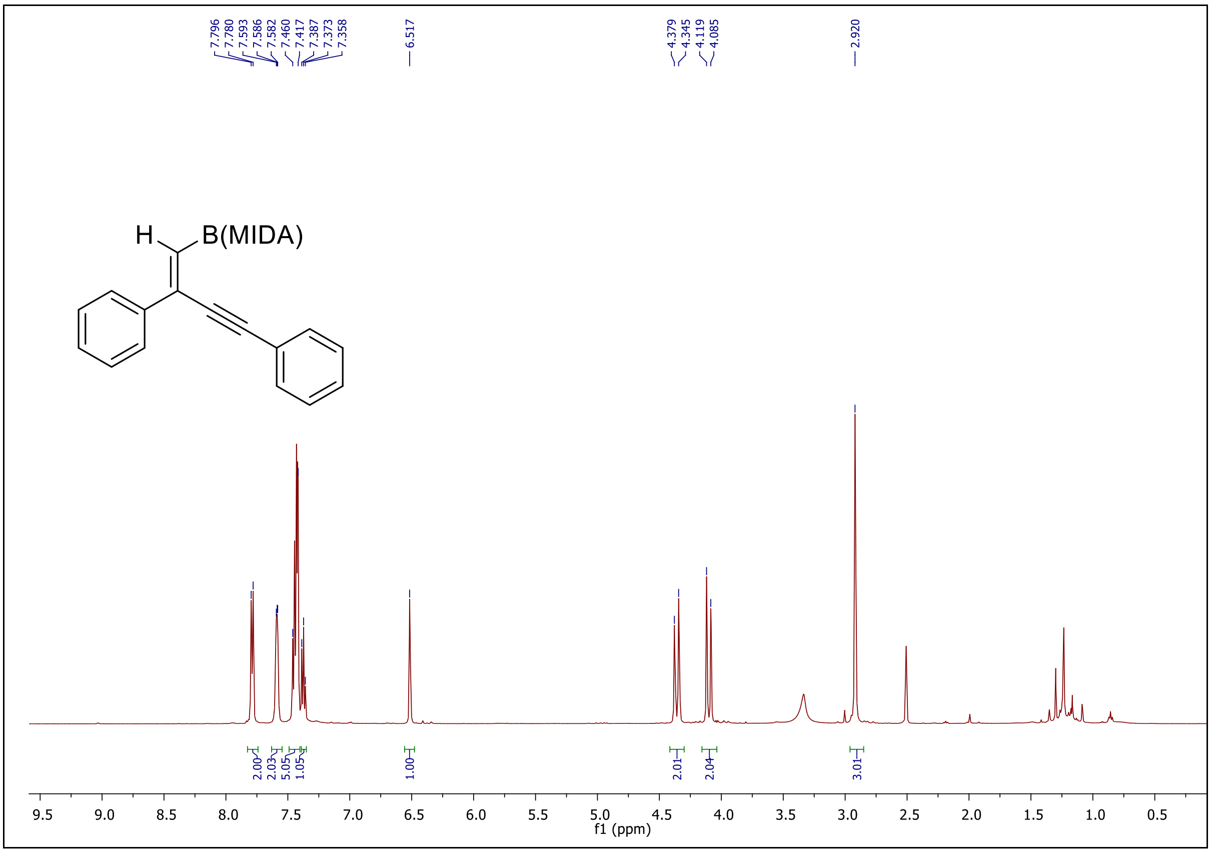

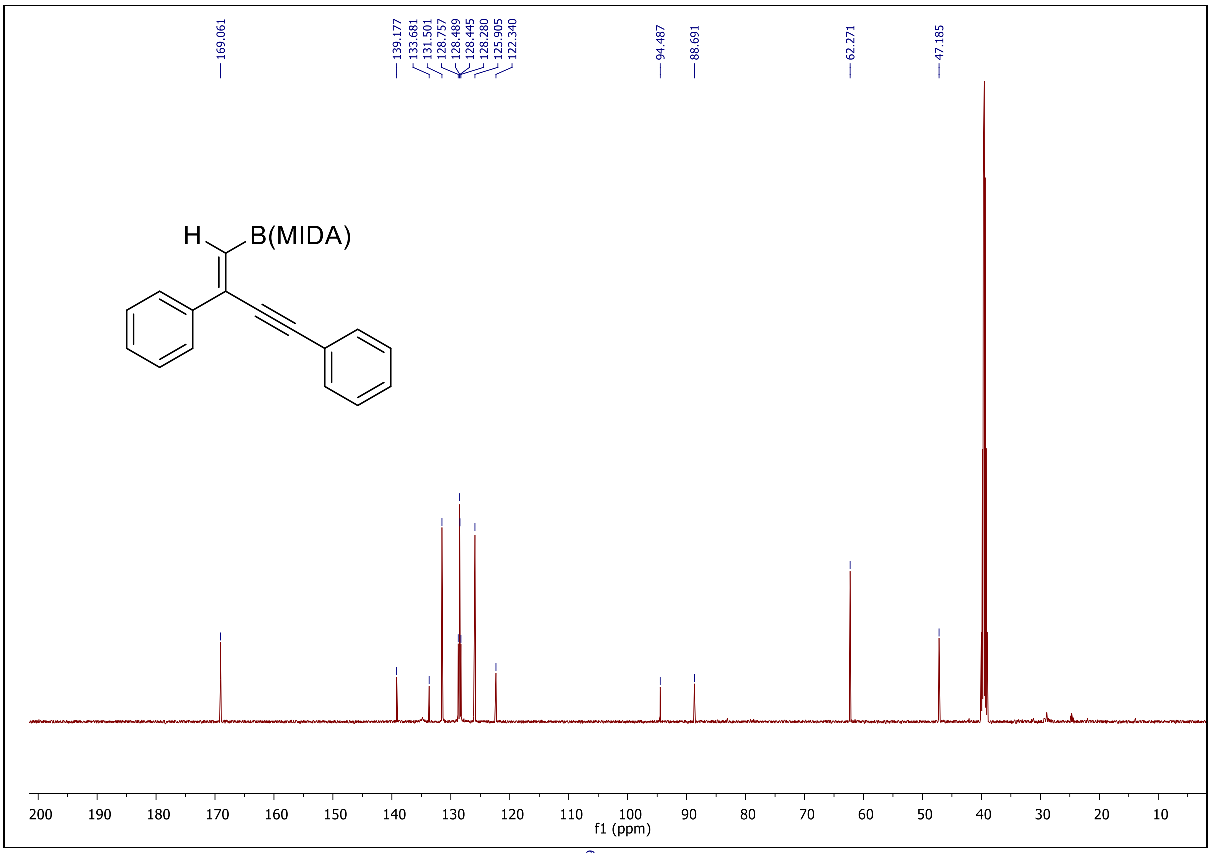
169.061
139.177
133.681
131.501
128.757
128.489
128.445
128.280
125.905
122.340
94.487
88.691
62.271
47.185
H
B(MIDA)
200 190 180 170 160 150 140 130 120 110 100 90 80 70 60 50 40 30 20 10
f1 (ppm)

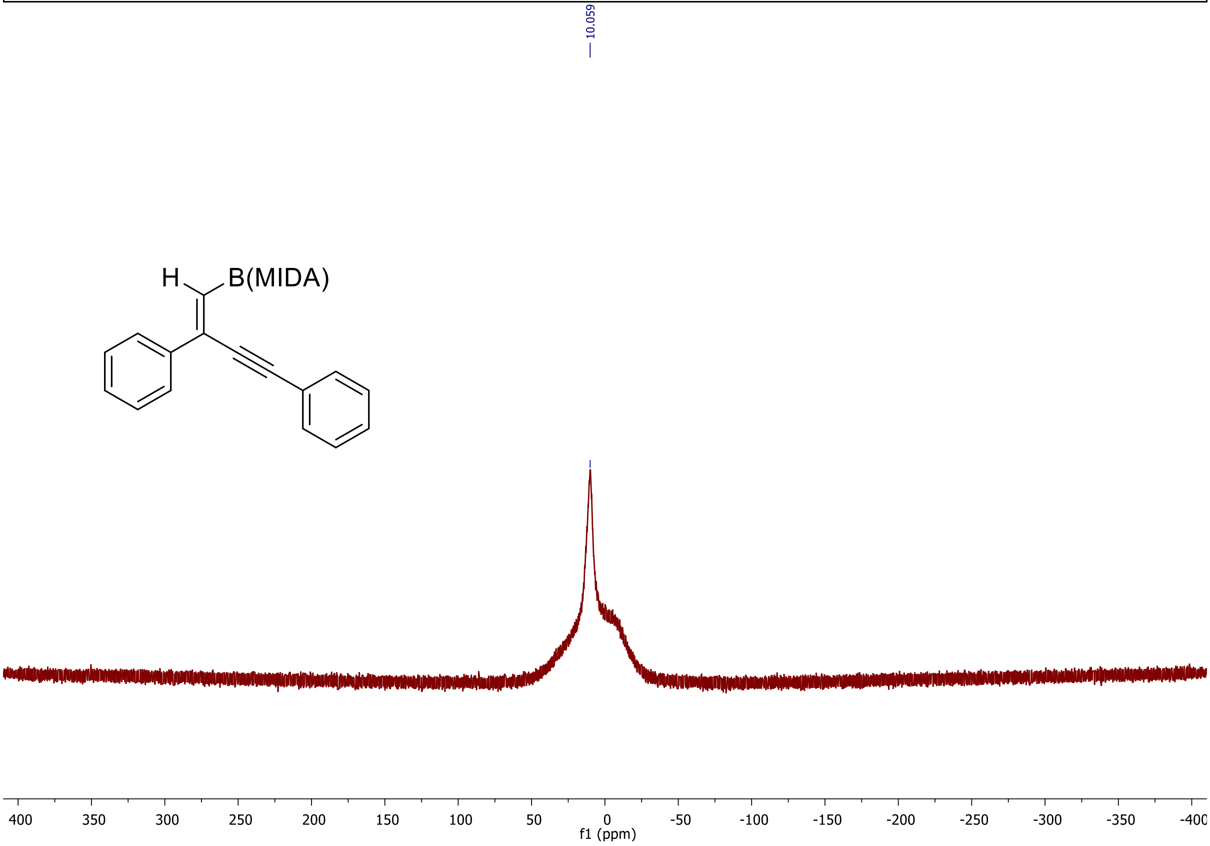
10.059
H
B(MIDA)
400 350 300 250 200 150 100 50 0 -50 -100 -150 -200 -250 -300 -350 -400
f1 (ppm)

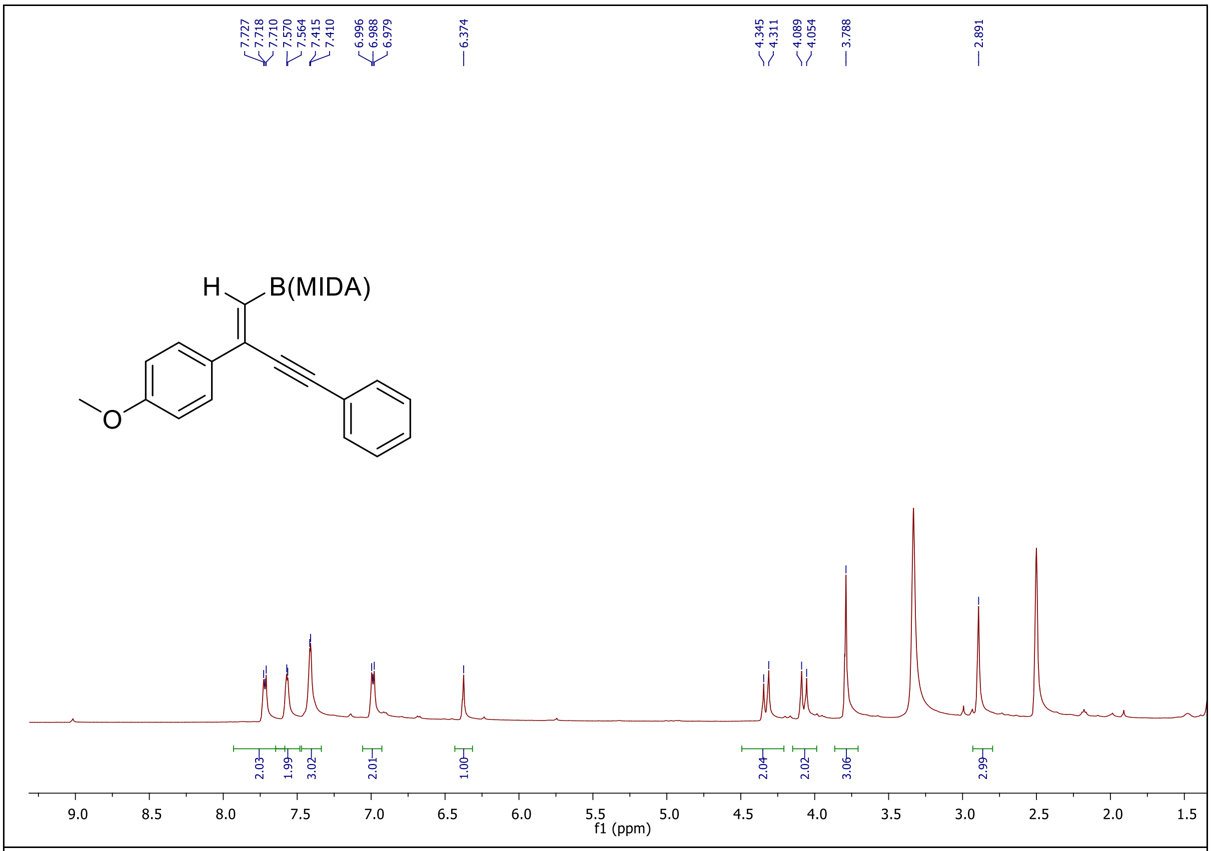

7.727
7.718
7.710
7.570
7.564
7.415
7.410
6.996
6.988
6.979
6.374
4.345
4.311
4.089
4.054
3.788
2.891
H
B(MIDA)
O
2.03
1.99
3.02
2.01
1.00
2.04
2.02
3.06
2.99
f1 (ppm)

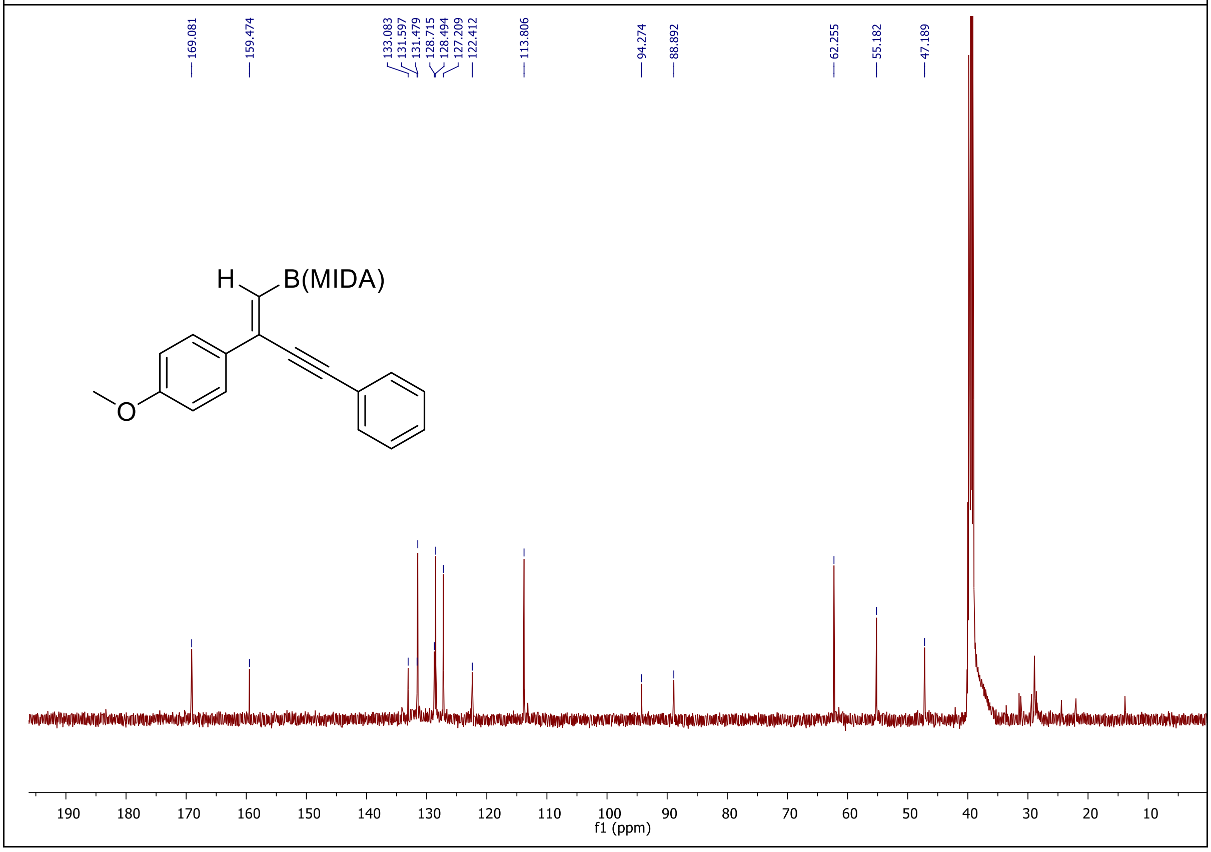

169.081
159.474
133.083
131.597
131.479
128.715
128.494
127.209
122.412
113.806
94.274
88.892
62.255
55.182
47.189
H
B(MIDA)
O
f1 (ppm)

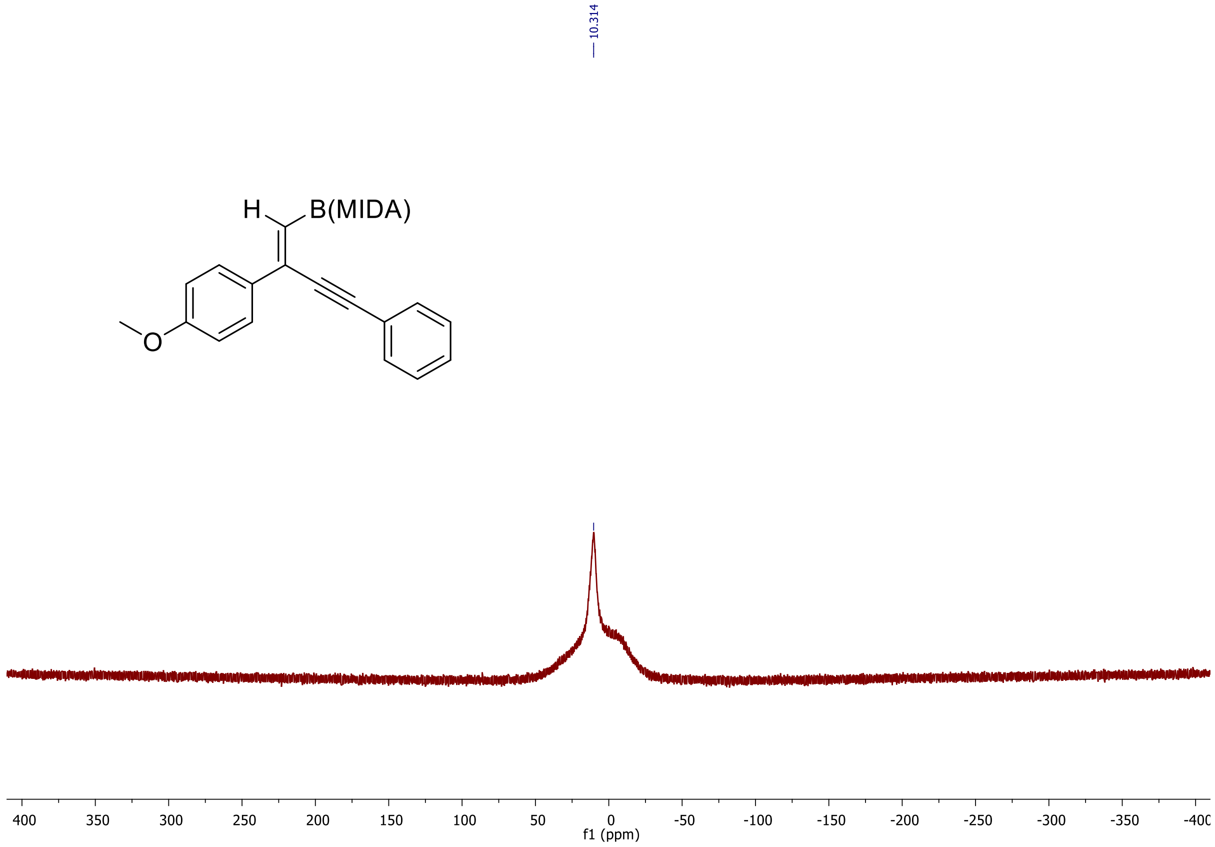
10.314
H
B(MIDA)
O
f1 (ppm)

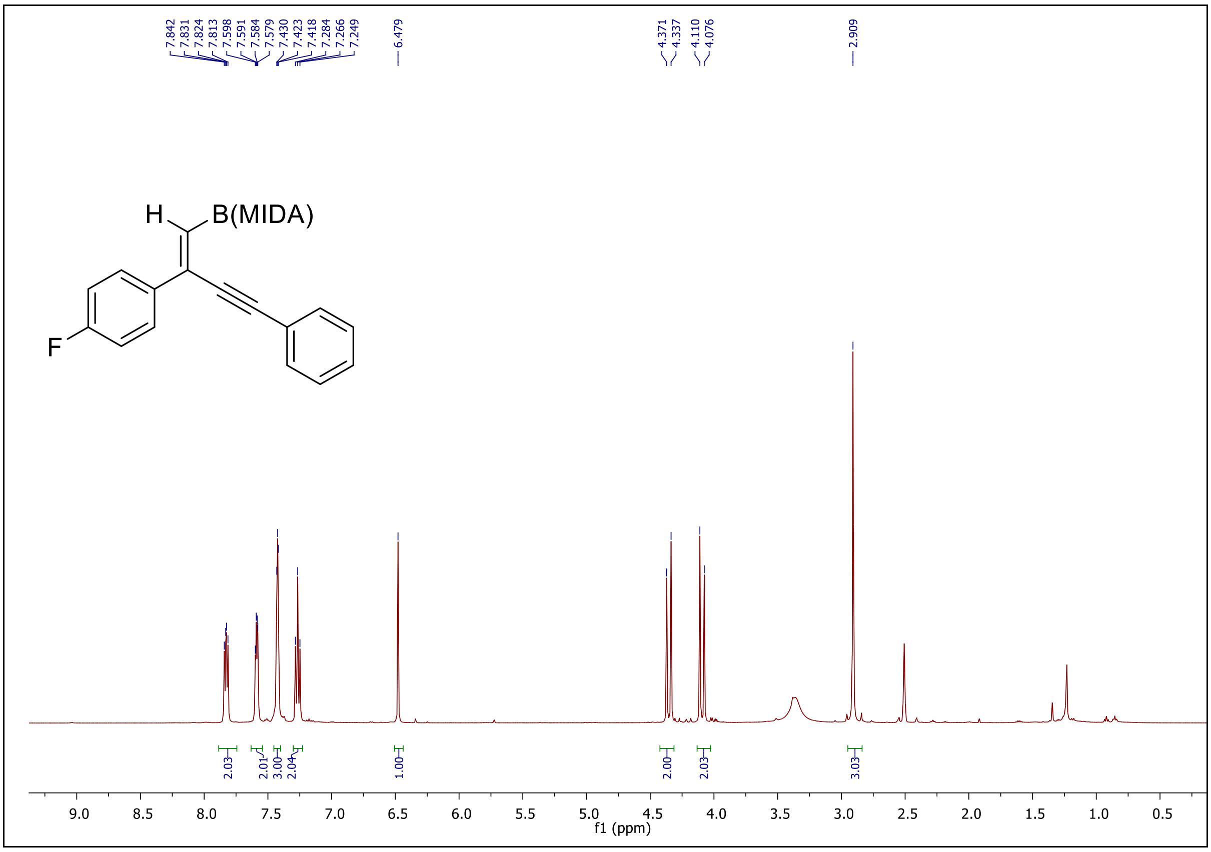
7.842
7.831
7.824
7.813
7.598
7.591
7.584
7.579
7.430
7.423
7.418
7.284
7.266
7.249
6.479
4.371
4.337
4.110
4.076
2.909
H
B(MIDA)
F
2.03
2.01
3.00
2.04
1.00
2.00
2.03
3.03
f1 (ppm)

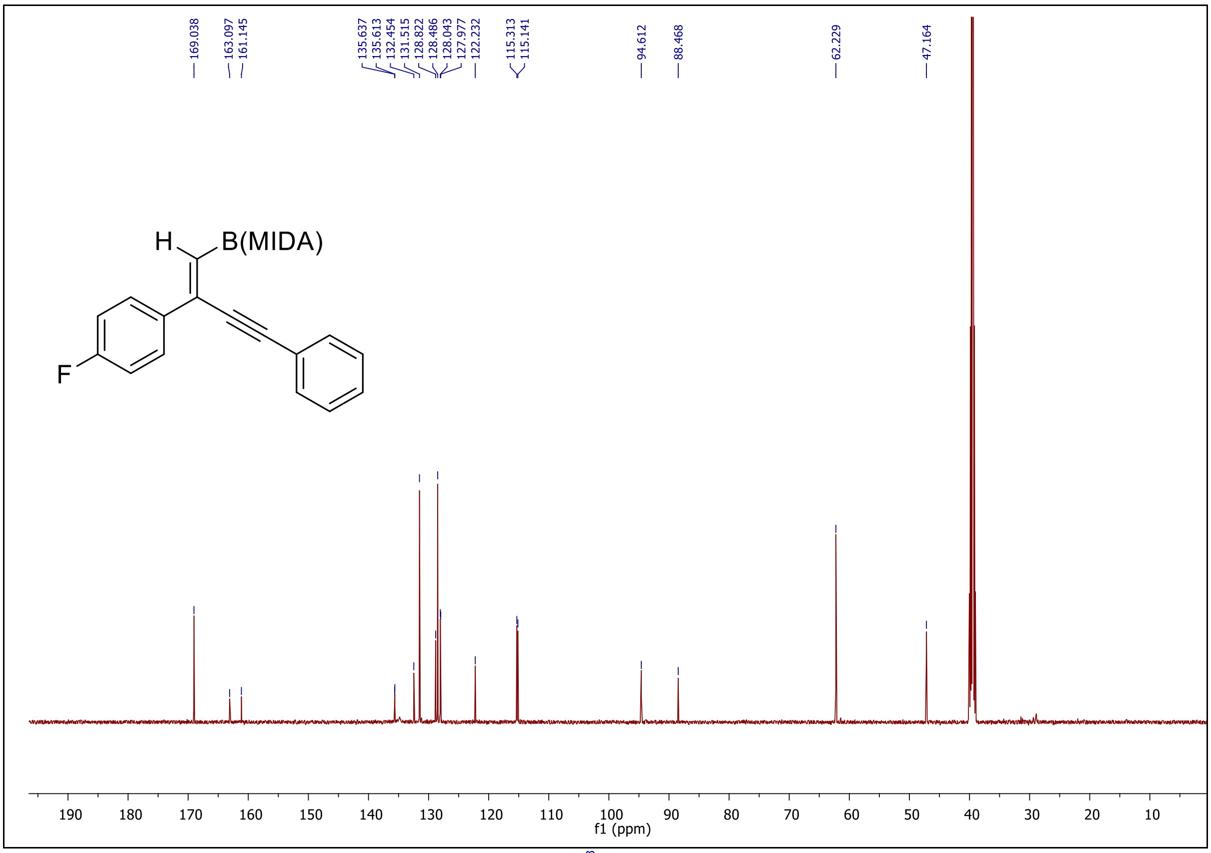
169.038
163.097
161.145
135.637
135.613
132.454
131.515
128.822
128.486
128.043
127.977
122.232
115.313
115.141
94.612
88.468
62.229
47.164
H
B(MIDA)
F
190
180
170
160
150
140
130
120
110
100
90
80
70
60
50
40
30
20
10
f1 (ppm)

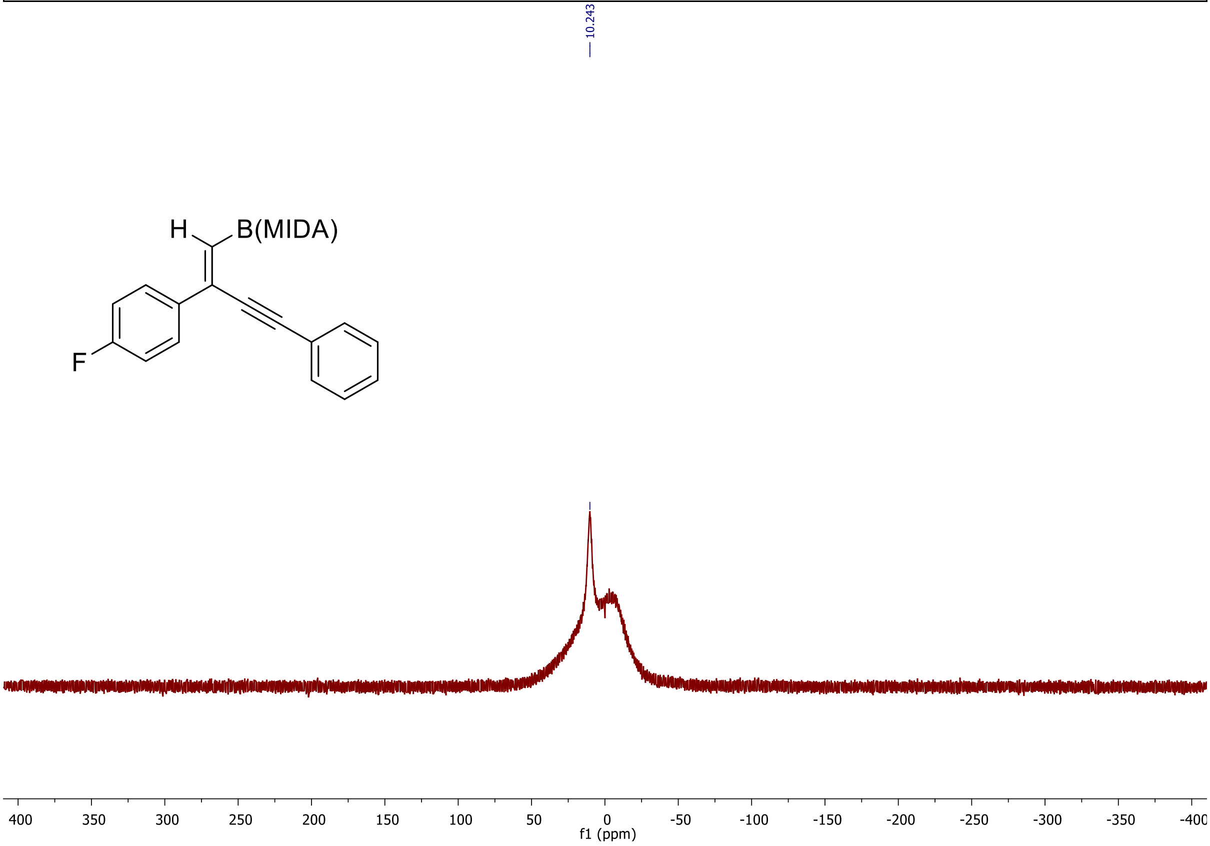
10.243
H
B(MIDA)
F
400
350
300
250
200
150
100
50
0
-50
-100
-150
-200
-250
-300
-350
-400
f1 (ppm)

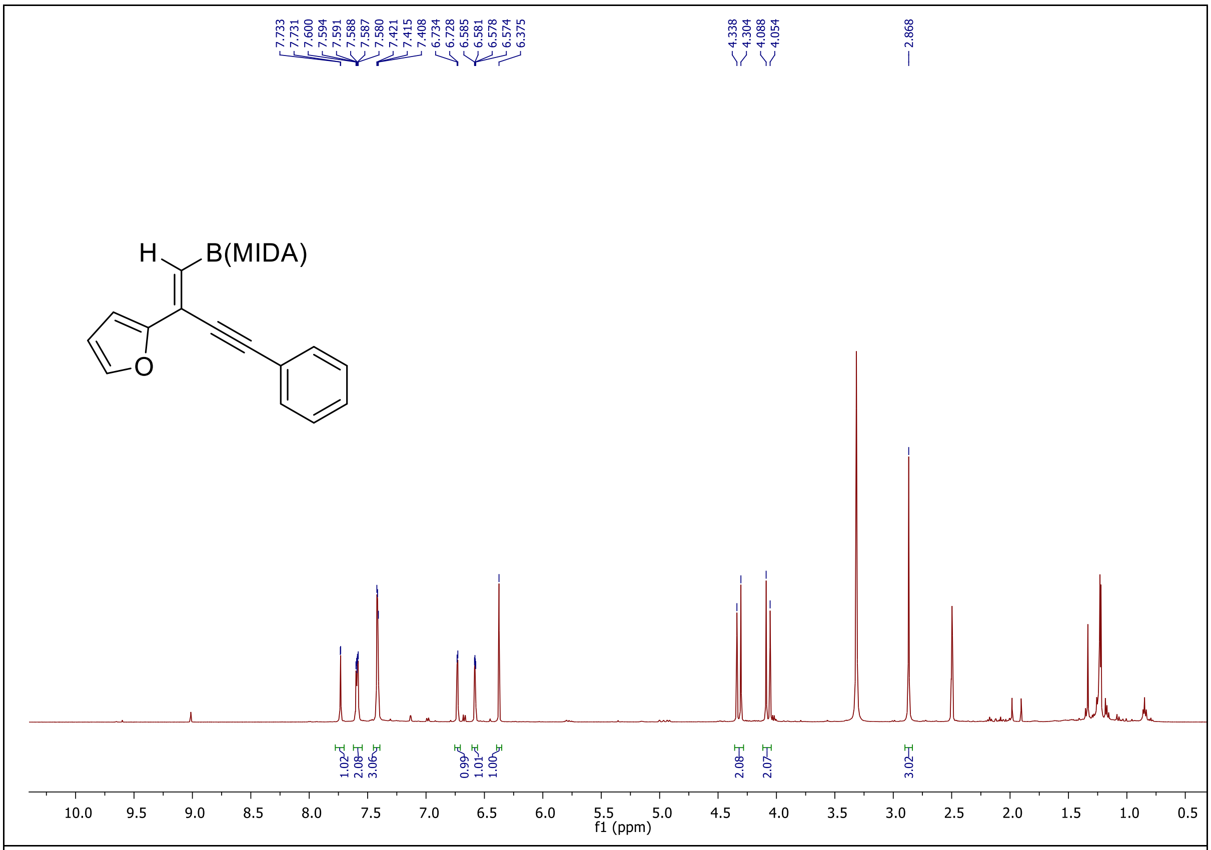

H
B(MIDA)
O
7.733
7.731
7.600
7.594
7.591
7.588
7.587
7.580
7.421
7.415
7.408
6.734
6.728
6.585
6.581
6.578
6.574
6.375
4.338
4.304
4.088
4.054
2.868
1.02
2.08
3.06
0.99
1.01
1.00
2.08
2.07
3.02
f1 (ppm)

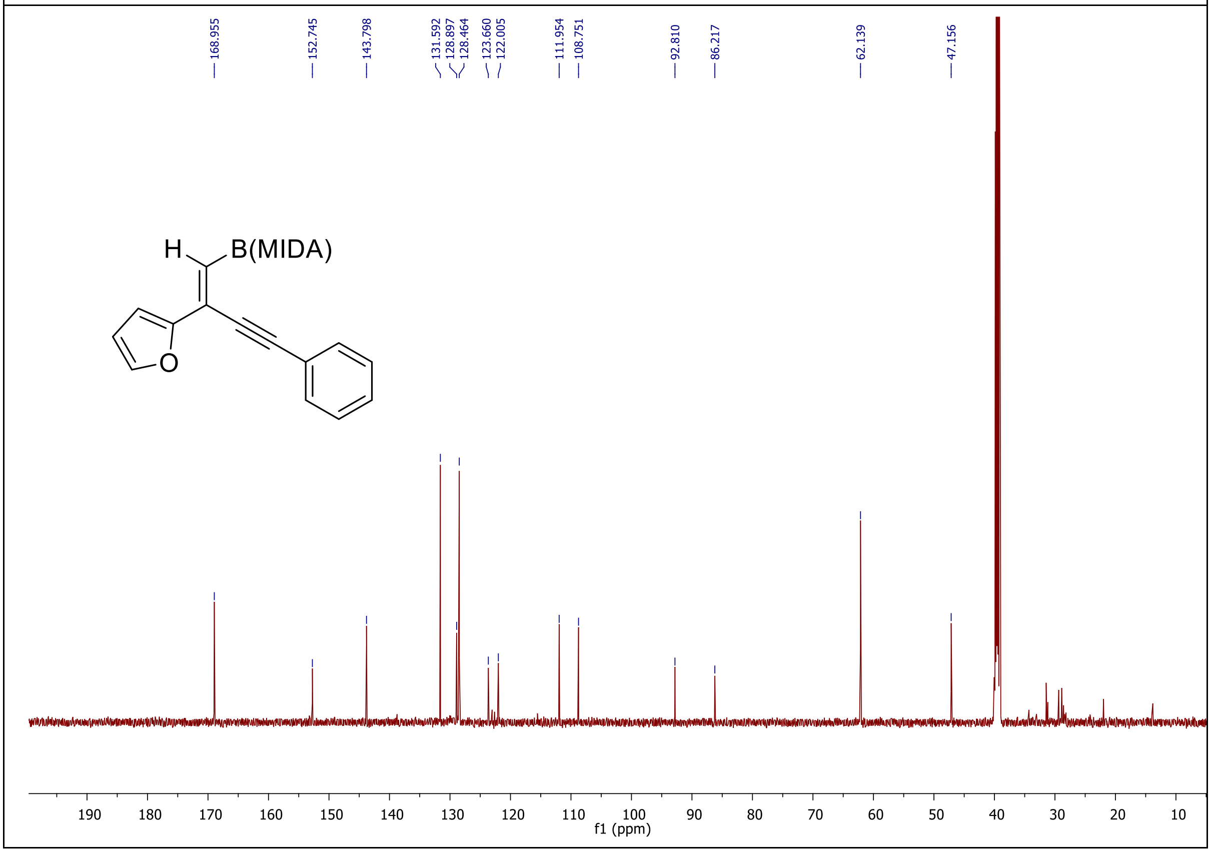

H
B(MIDA)
O
168.955
152.745
143.798
131.592
128.897
128.464
123.660
122.005
111.954
108.751
92.810
86.217
62.139
47.156
f1 (ppm)

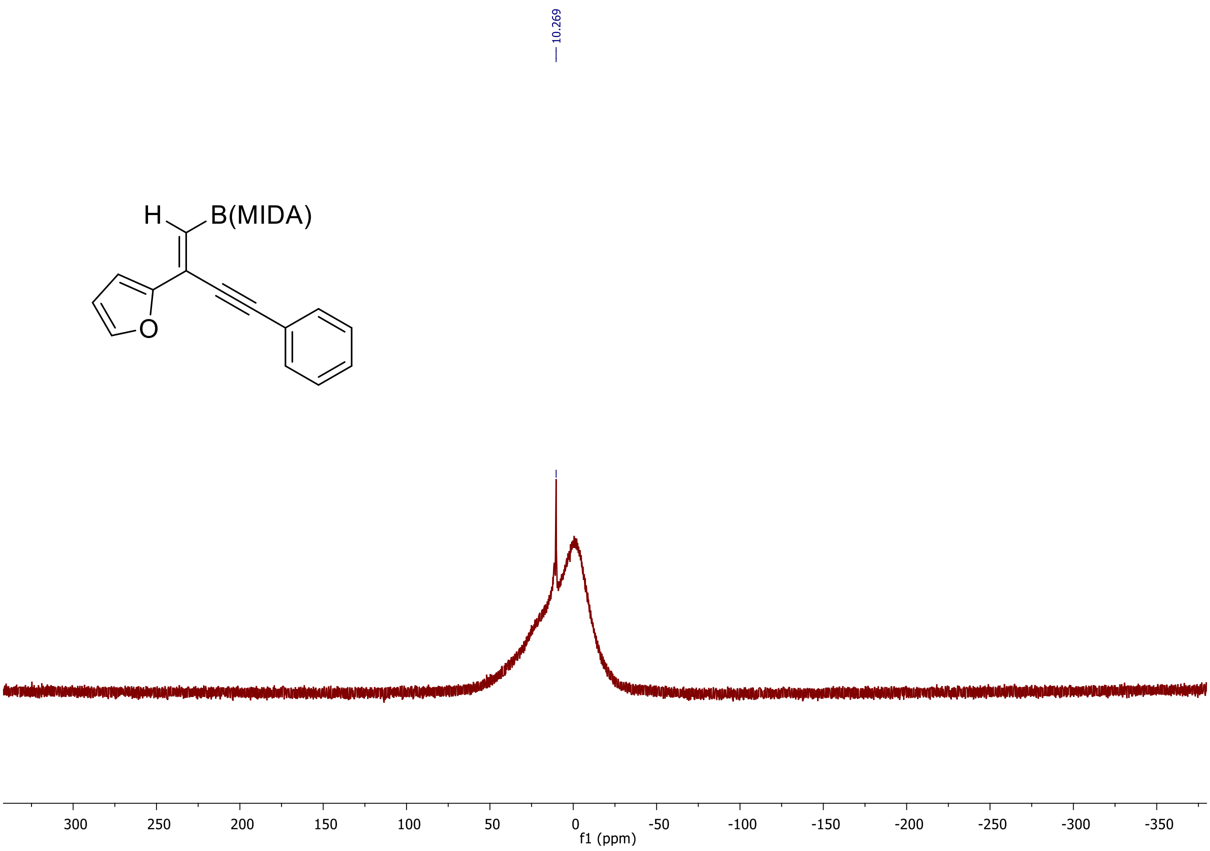
10.269
H
B(MIDA)
O
300
250
200
150
100
50
0
-50
-100
-150
-200
-250
-300
-350
f1 (ppm)

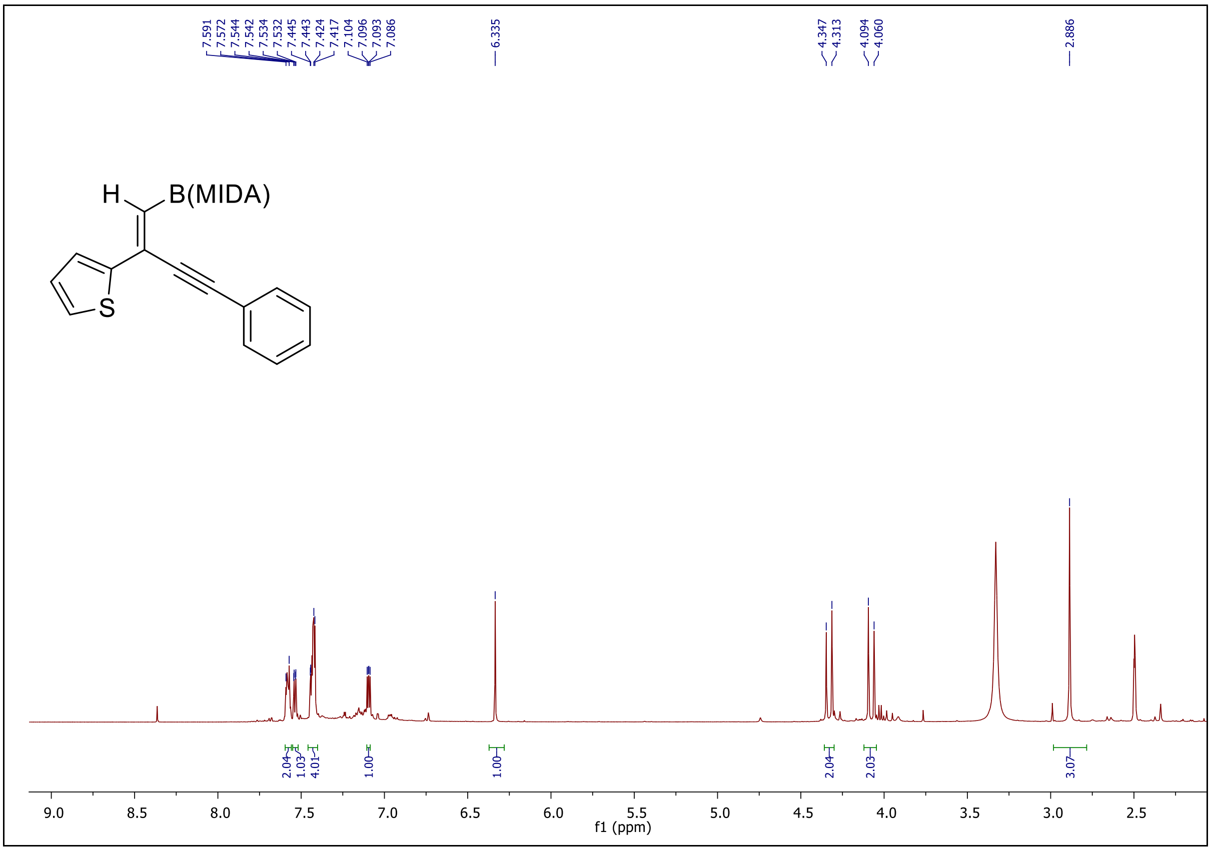
7.591
7.572
7.544
7.542
7.534
7.532
7.445
7.443
7.424
7.417
7.104
7.096
7.093
7.086
6.335
4.347
4.313
4.094
4.060
2.886
H
B(MIDA)
S
2.04
1.03
4.01
1.00
1.00
2.04
2.03
3.07
9.0
8.5
8.0
7.5
7.0
6.5
6.0
5.5
5.0
4.5
4.0
3.5
3.0
2.5
f1 (ppm)

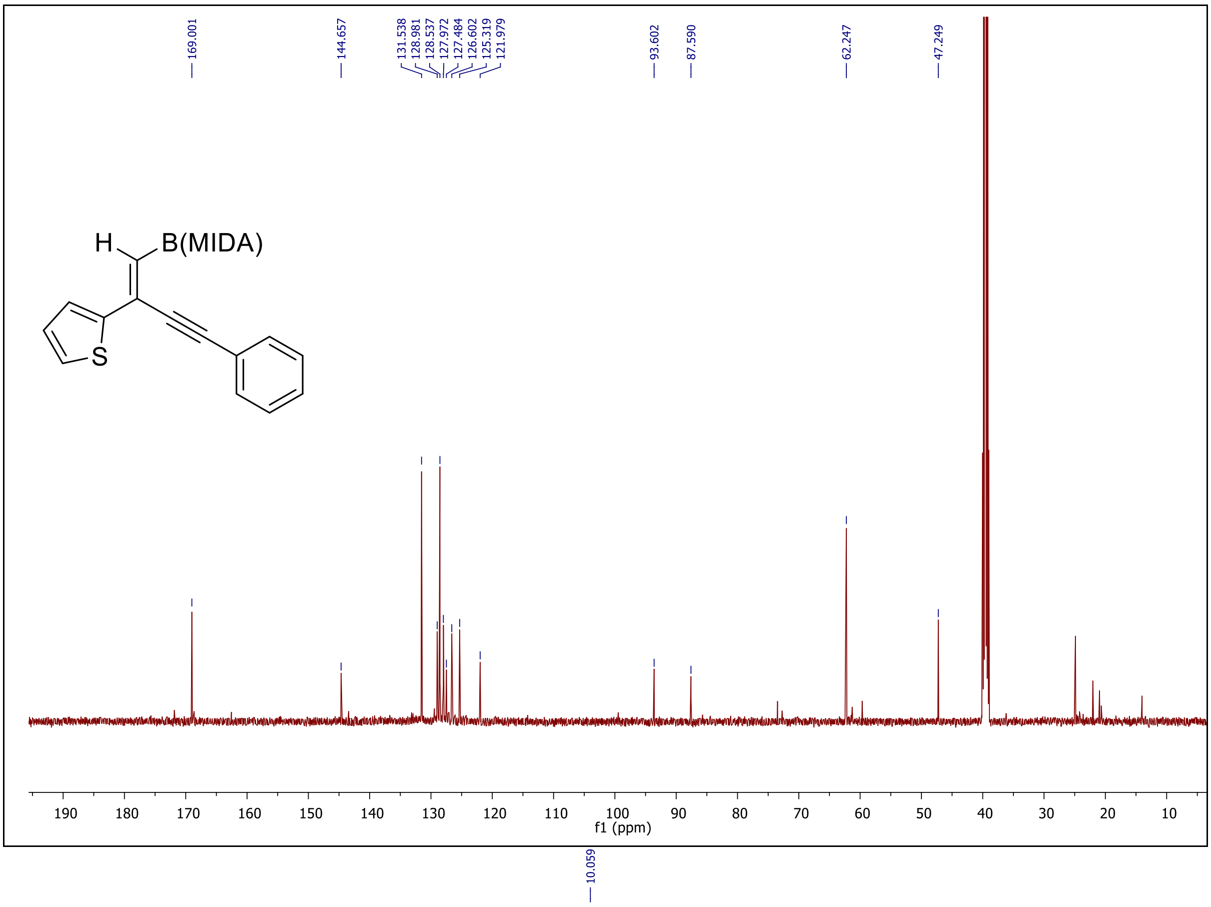
169.001
144.657
131.538
128.981
128.537
127.972
127.484
126.602
125.319
121.979
93.602
87.590
62.247
47.249
H
B(MIDA)
S
190
180
170
160
150
140
130
120
110
100
90
80
70
60
50
40
30
20
10
f1 (ppm)

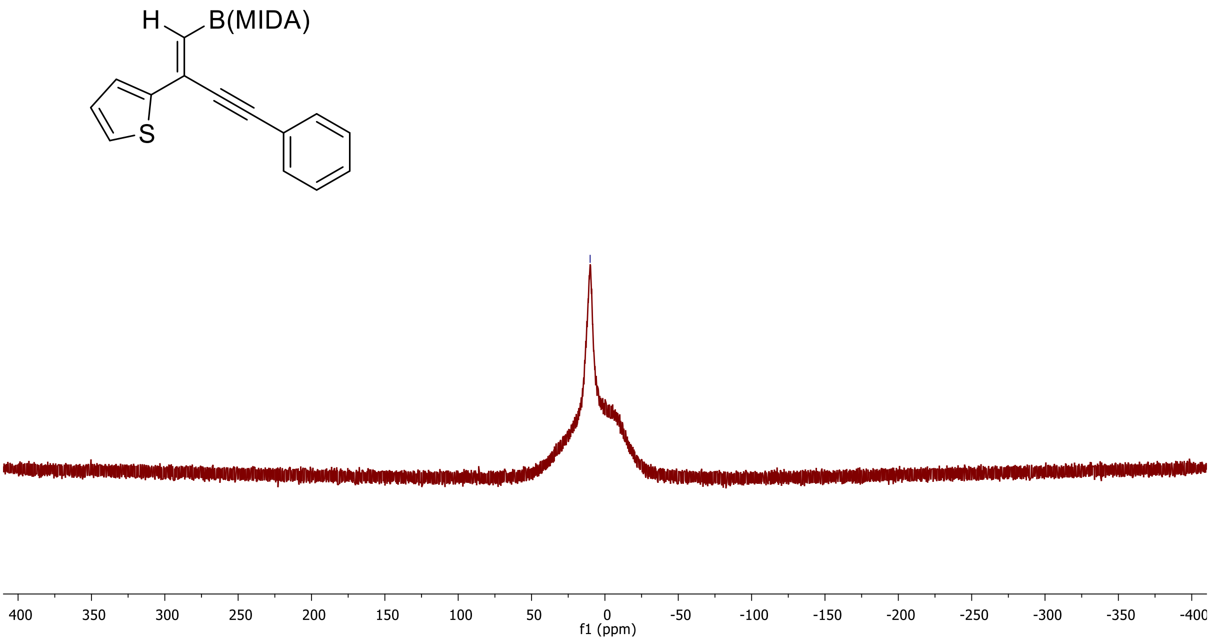
10.059
H
B(MIDA)
S
400
350
300
250
200
150
100
50
0
-50
-100
-150
-200
-250
-300
-350
-400
f1 (ppm)

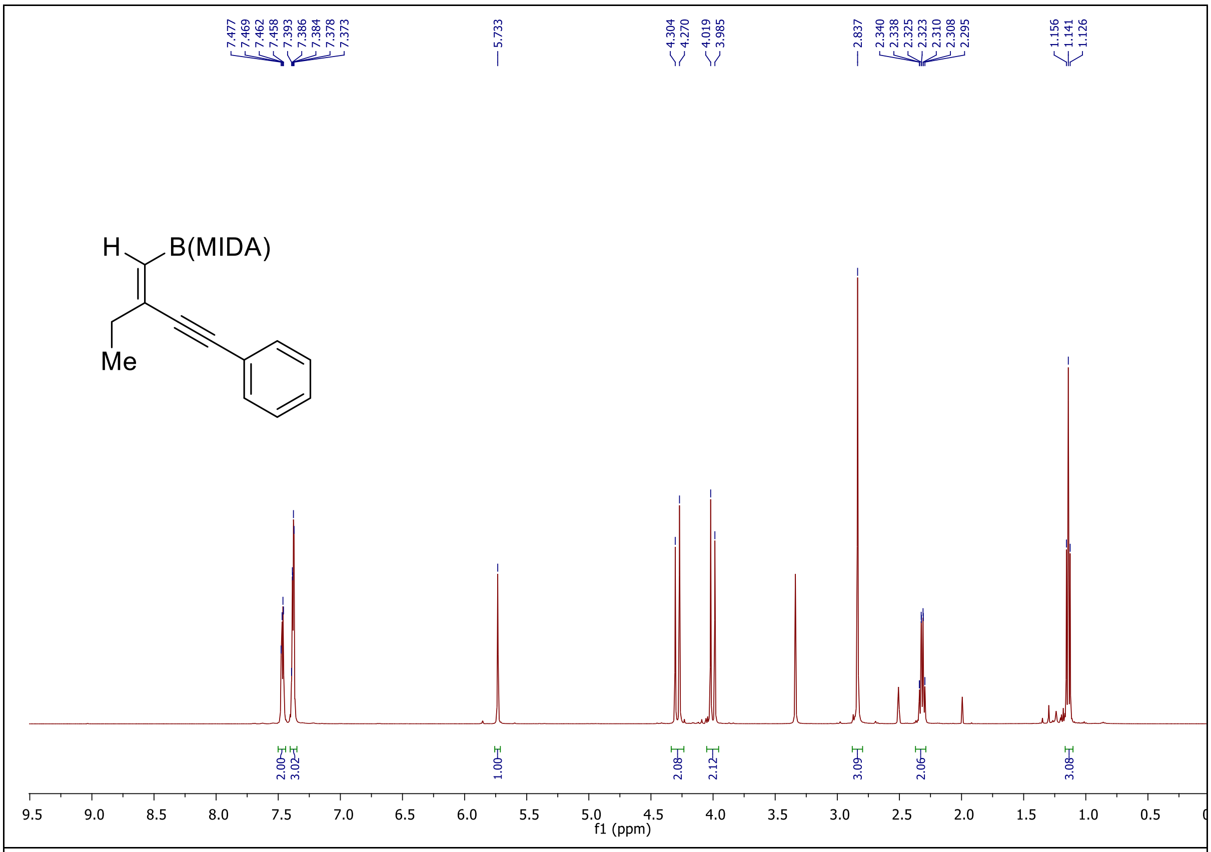
7.477
7.469
7.462
7.458
7.393
7.386
7.384
7.378
7.373
5.733
4.304
4.270
4.019
3.985
2.837
2.340
2.338
2.325
2.323
2.310
2.308
2.295
1.156
1.141
1.126
H
B(MIDA)
Me
2.00
3.02
1.00
2.08
2.12
3.09
2.06
3.08
f1 (ppm)

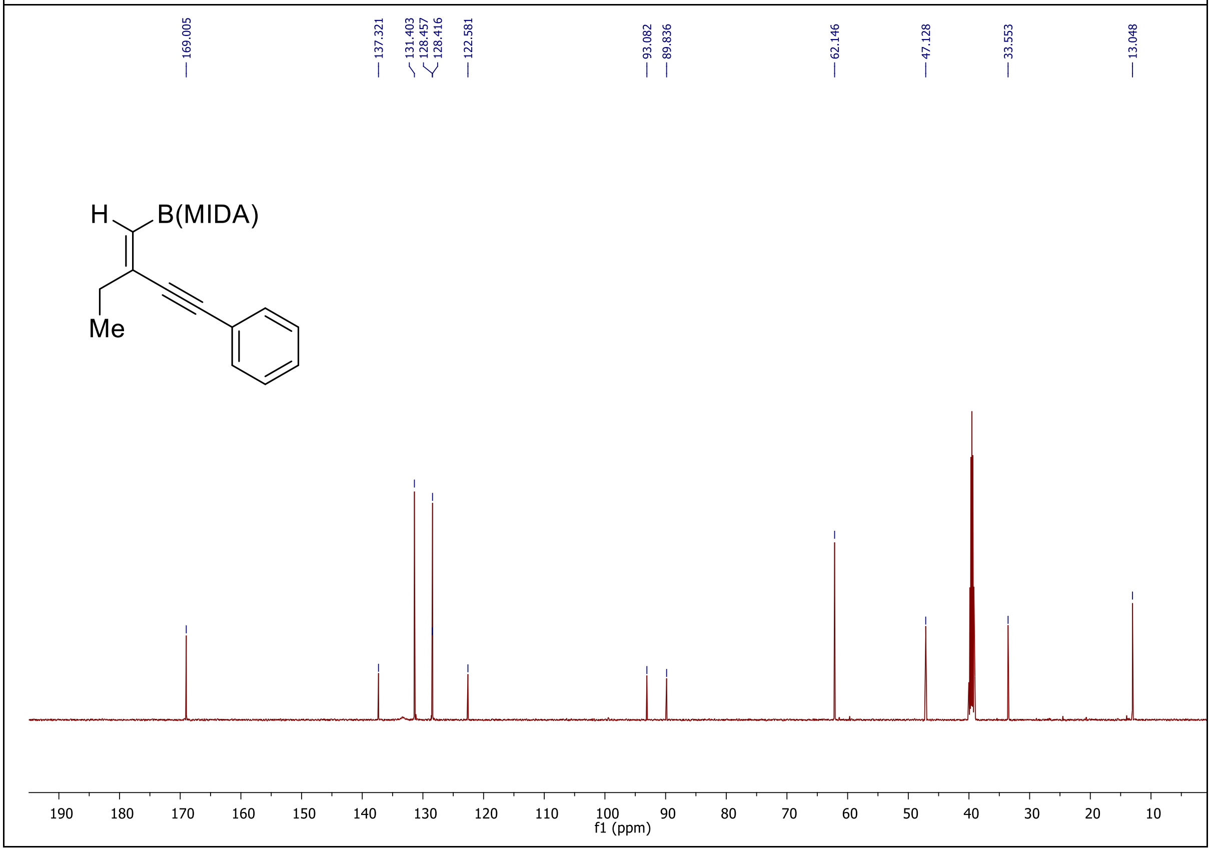
169.005
137.321
131.403
128.457
128.416
122.581
93.082
89.836
62.146
47.128
33.553
13.048
H
B(MIDA)
Me
f1 (ppm)

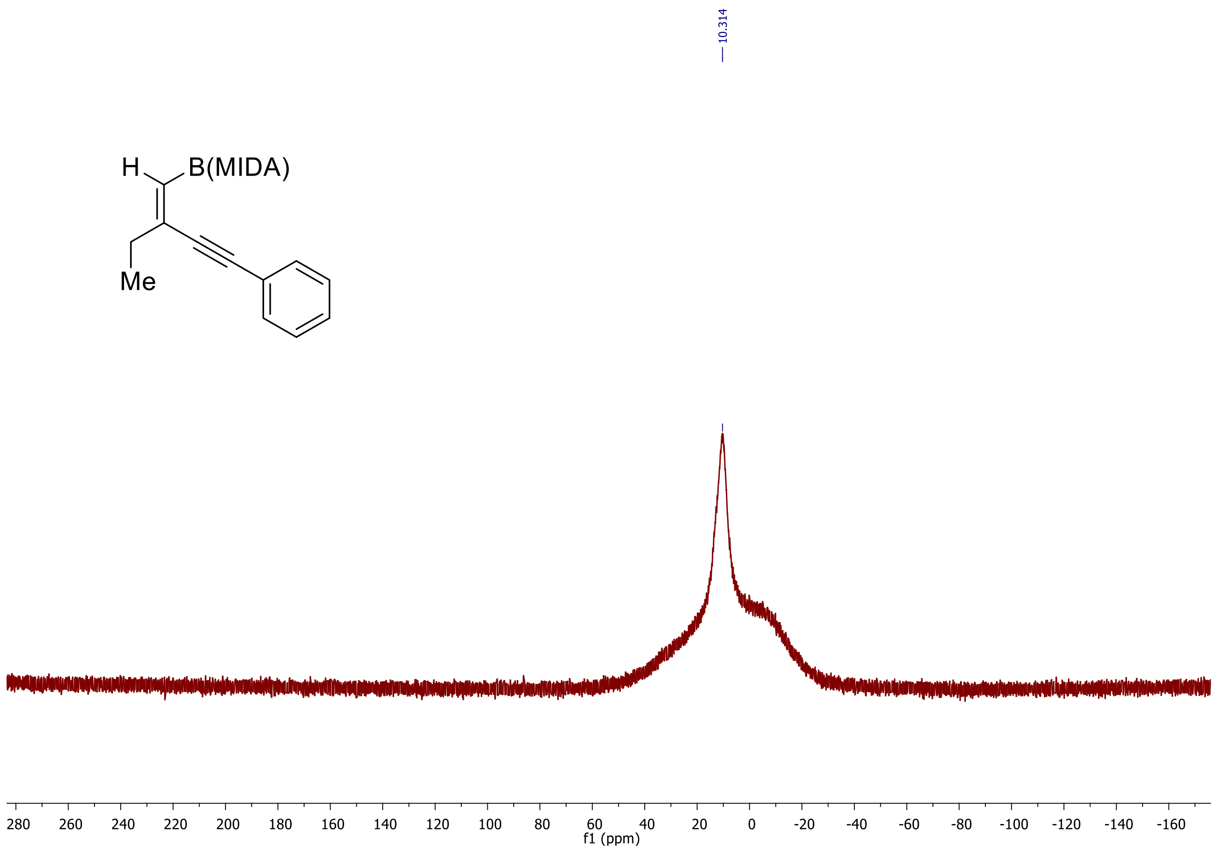
10.314
H
B(MIDA)
Me
280 260 240 220 200 180 160 140 120 100 80 60 40 20 0 -20 -40 -60 -80 -100 -120 -140 -160
f1 (ppm)

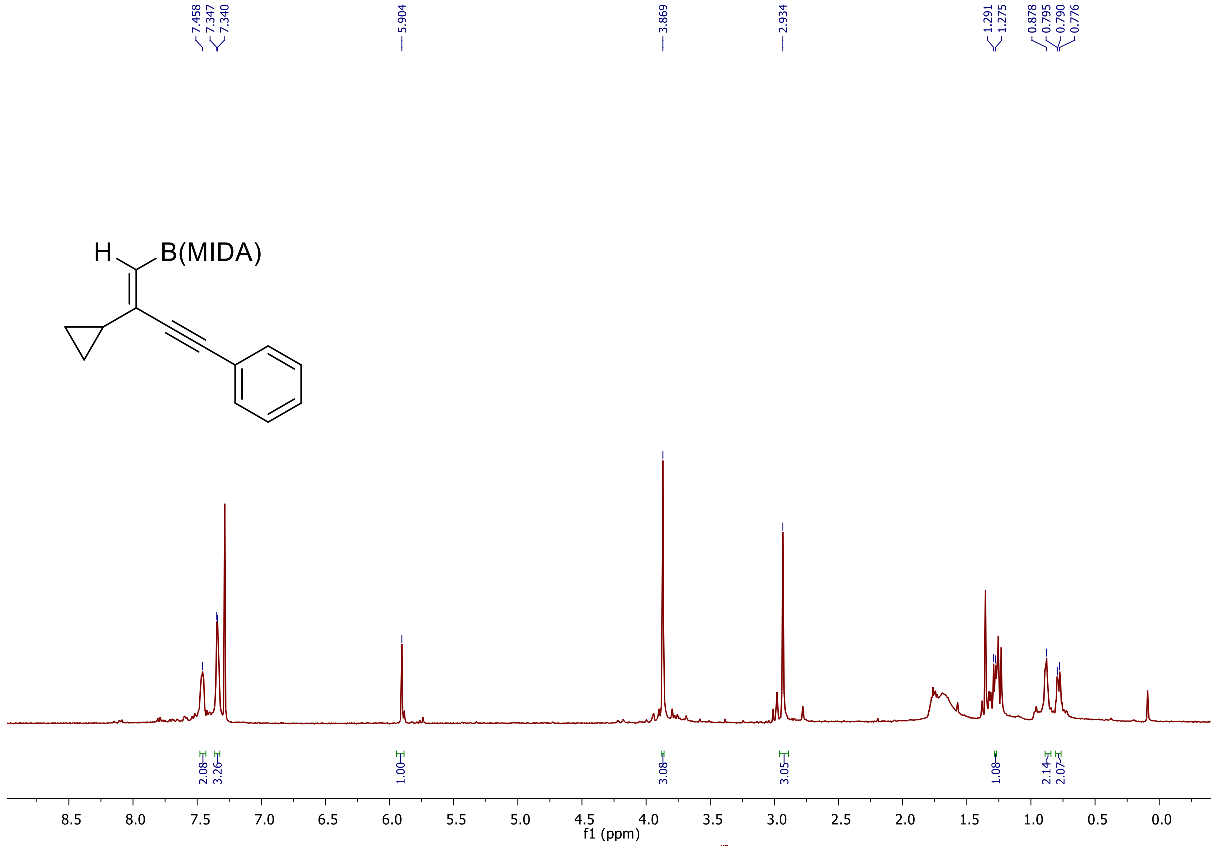

7.458
7.347
7.340
5.904
3.869
2.934
1.291
1.275
0.878
0.795
0.790
0.776
H
B(MIDA)
2.08
3.26
1.00
3.08
3.05
1.08
2.14
2.07
8.5 8.0 7.5 7.0 6.5 6.0 5.5 5.0 4.5 4.0 3.5 3.0 2.5 2.0 1.5 1.0 0.5 0.0
f1 (ppm)

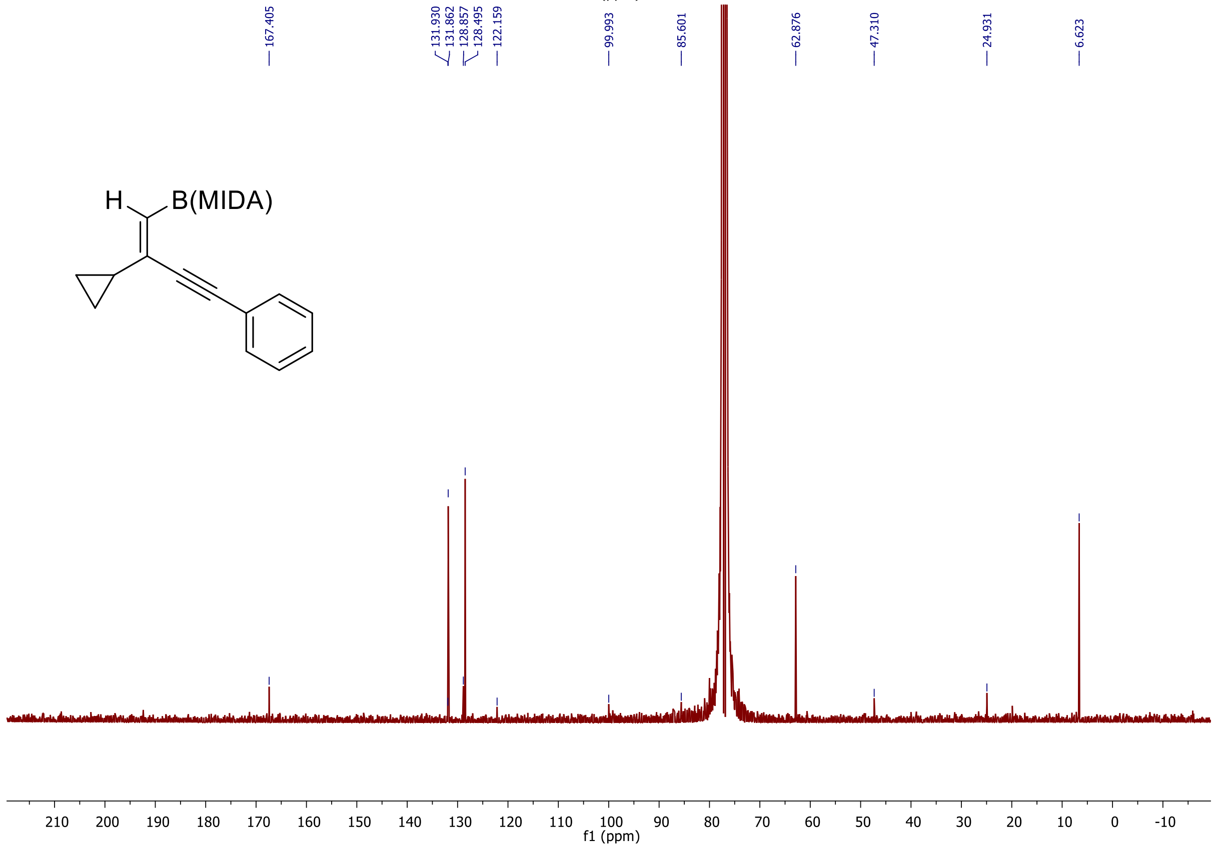

167.405
131.930
131.862
128.857
128.495
122.159
99.993
85.601
62.876
47.310
24.931
6.623
H
B(MIDA)
210 200 190 180 170 160 150 140 130 120 110 100 90 80 70 60 50 40 30 20 10 0 -10
f1 (ppm)

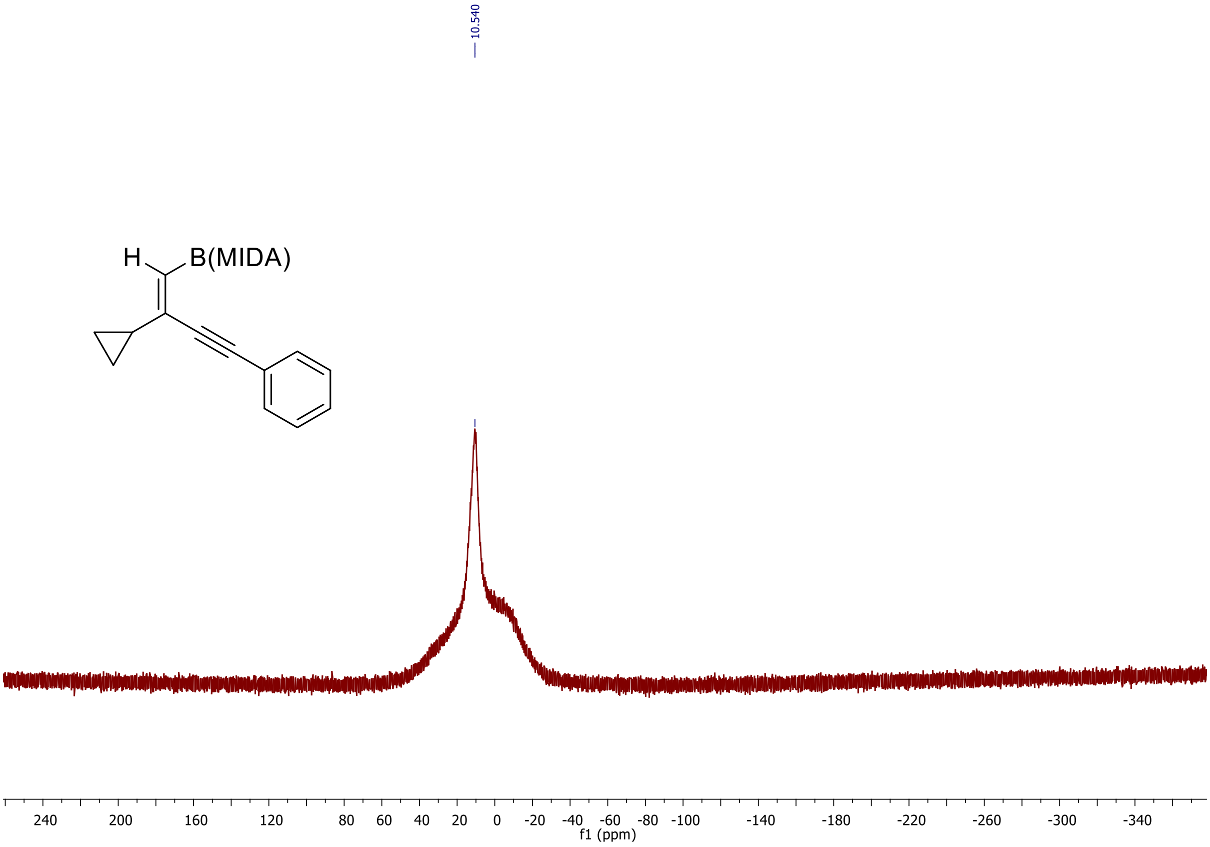

10.540
H
B(MIDA)
240
200
160
120
80
60
40
20
0
-20
-40
-60
-80
-100
-140
-180
-220
-260
-300
-340
f1 (ppm)

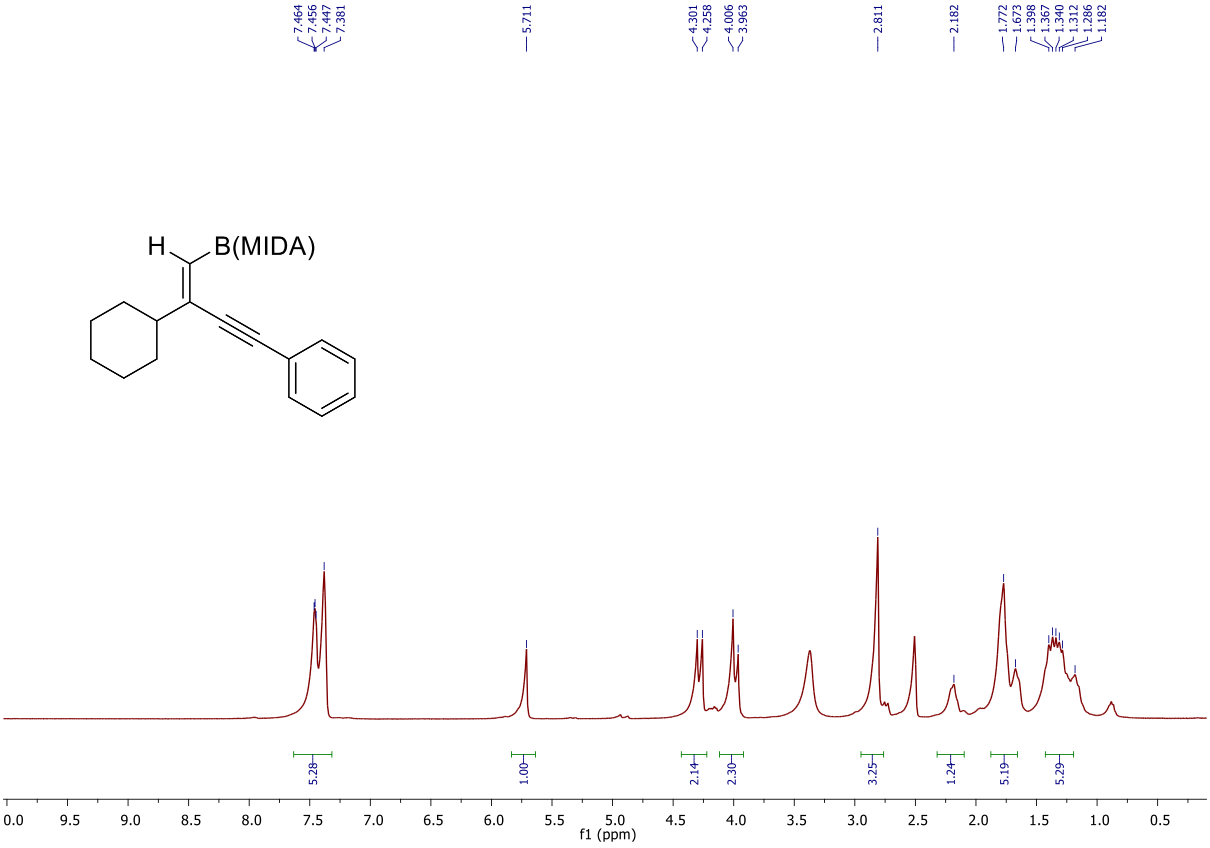

7.464
7.456
7.447
7.381
5.711
4.301
4.258
4.006
3.963
2.811
2.182
1.772
1.673
1.398
1.367
1.340
1.312
1.286
1.182
H
B(MIDA)
5.28
1.00
2.14
2.30
3.25
1.24
5.19
5.29
0.0
9.5
9.0
8.5
8.0
7.5
7.0
6.5
6.0
5.5
5.0
4.5
4.0
3.5
3.0
2.5
2.0
1.5
1.0
0.5
f1 (ppm)

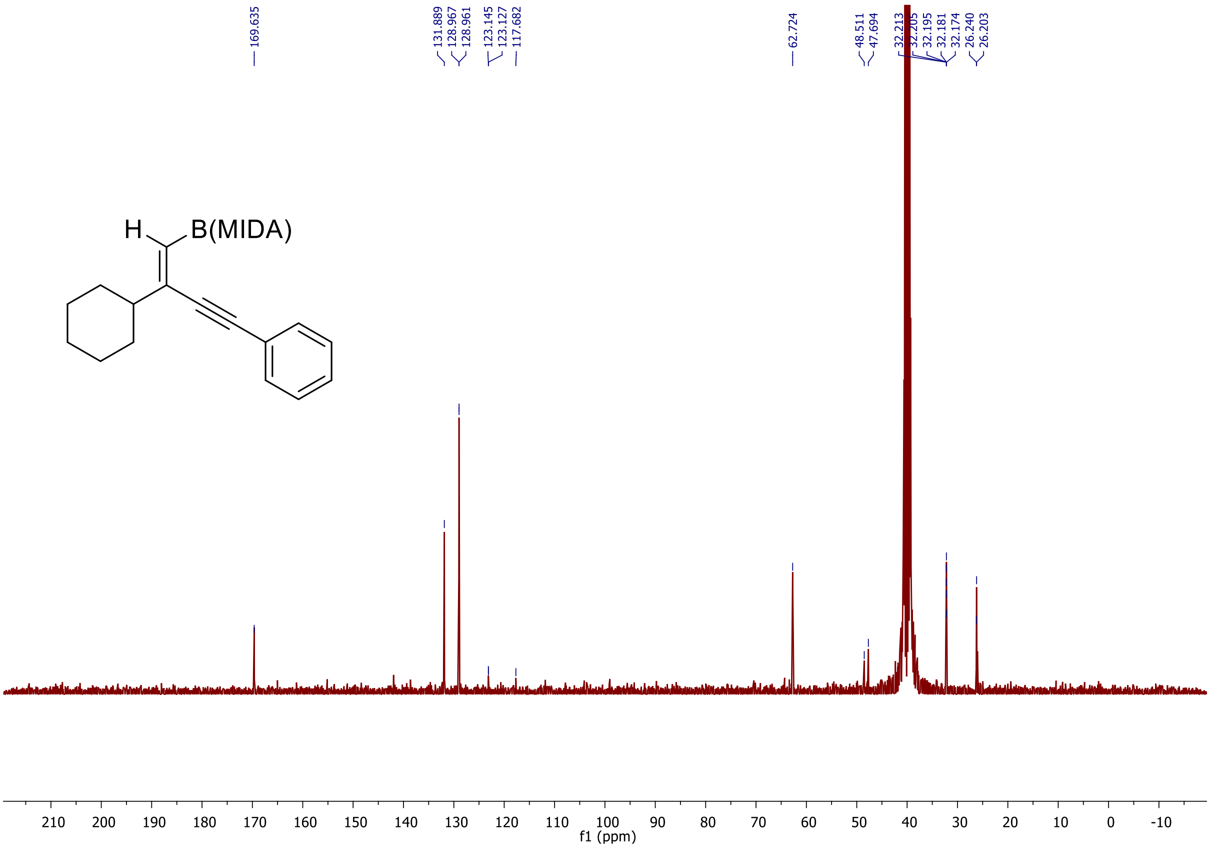
169.635
131.889
128.967
128.961
123.145
123.127
117.682
62.724
48.511
47.694
32.213
32.205
32.195
32.181
32.174
26.240
26.203
H
B(MIDA)
f1 (ppm)

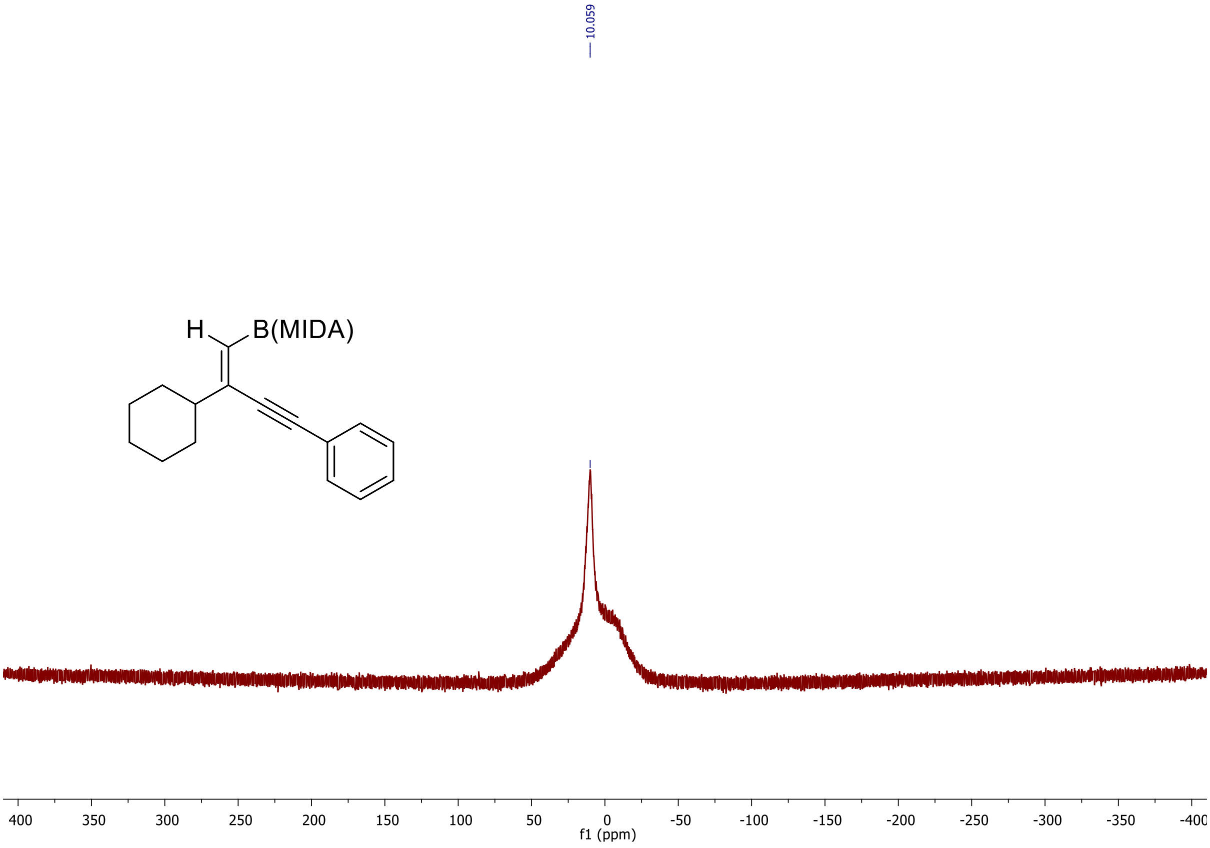
10.059
H
B(MIDA)
f1 (ppm)

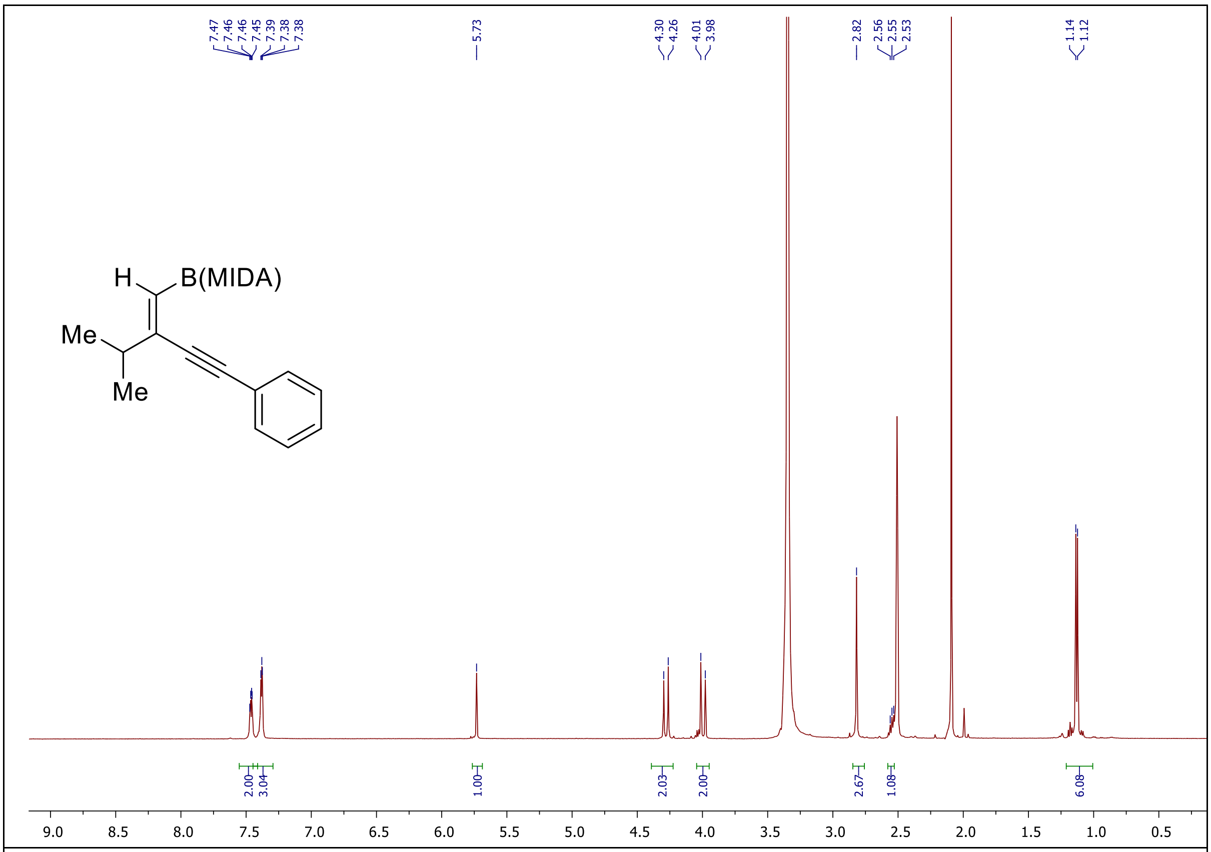
7.47
7.46
7.46
7.45
7.39
7.38
7.38
5.73
4.30
4.26
4.01
3.98
2.82
2.56
2.55
2.53
1.14
1.12
H
B(MIDA)
Me
Me
2.00
3.04
1.00
2.03
2.00
2.67
1.08
6.08
9.0 8.5 8.0 7.5 7.0 6.5 6.0 5.5 5.0 4.5 4.0 3.5 3.0 2.5 2.0 1.5 1.0 0.5

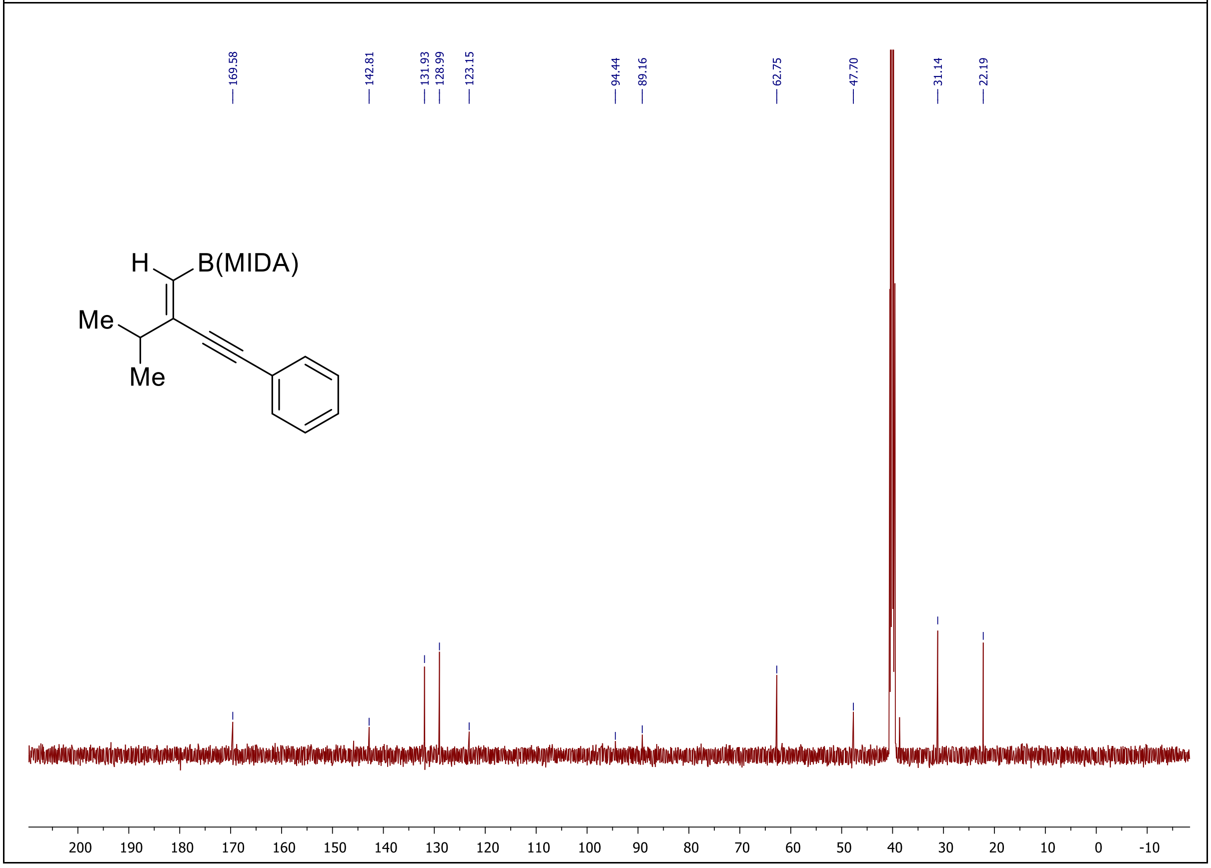
169.58
142.81
131.93
128.99
123.15
94.44
89.16
62.75
47.70
31.14
22.19
H
B(MIDA)
Me
Me
200 190 180 170 160 150 140 130 120 110 100 90 80 70 60 50 40 30 20 10 0 -10

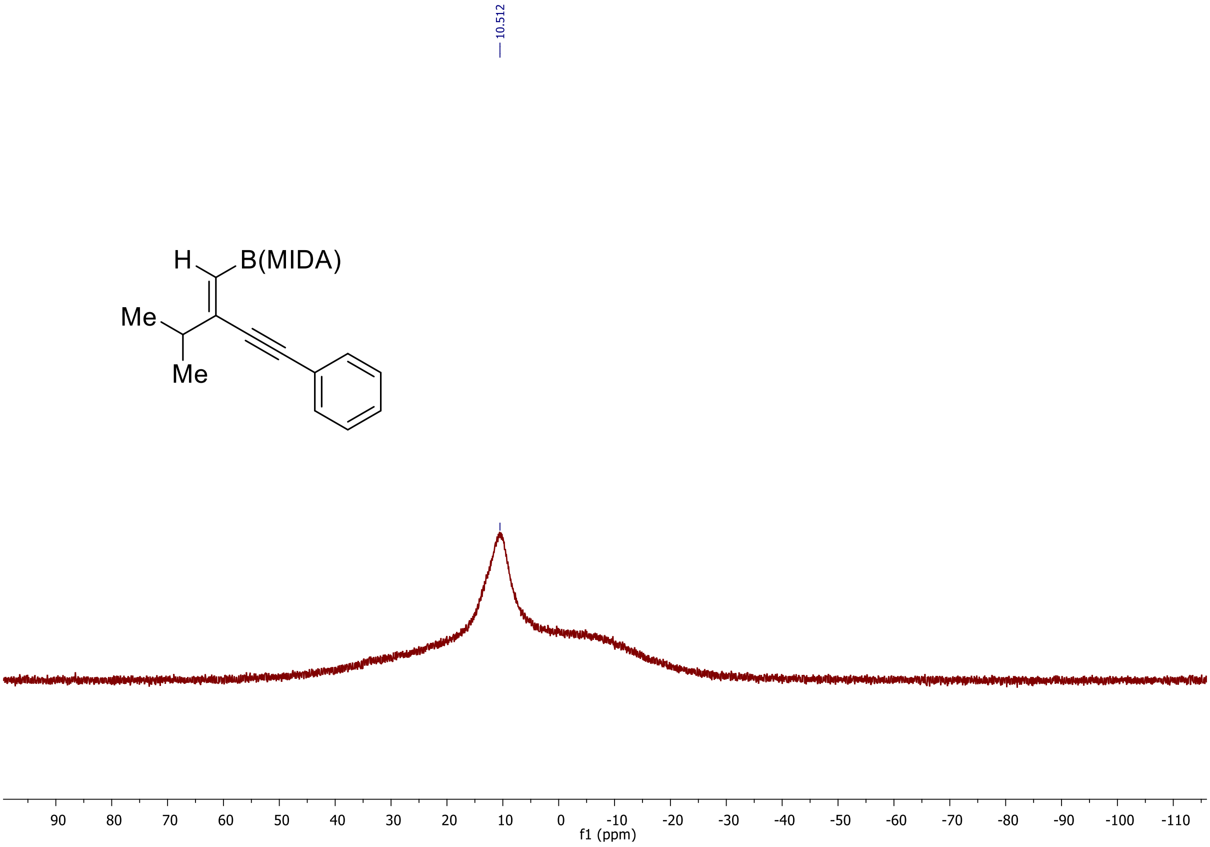
10.512
H
B(MIDA)
Me
Me
90
80
70
60
50
40
30
20
10
0
-10
-20
-30
-40
-50
-60
-70
-80
-90
-100
-110
f1 (ppm)

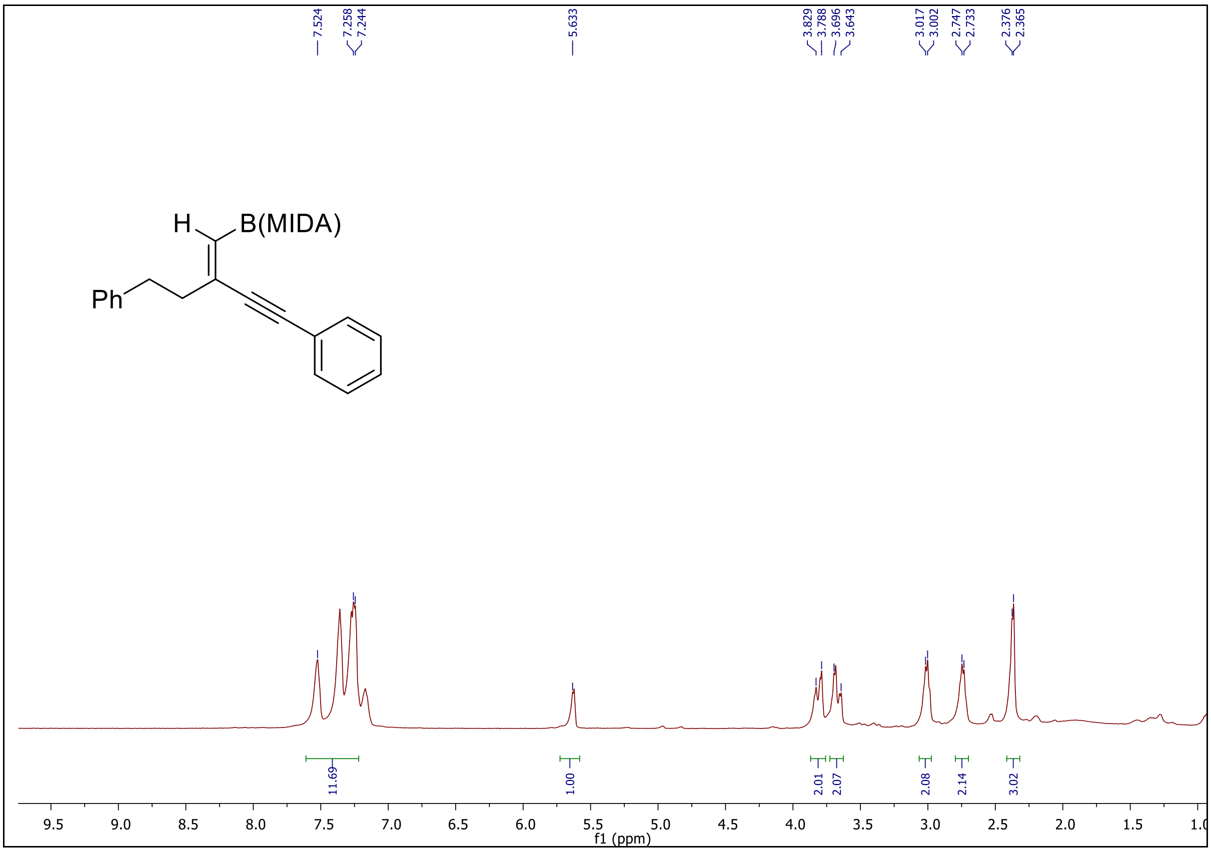
7.524
7.258
7.244
5.633
3.829
3.788
3.696
3.643
3.017
3.002
2.747
2.733
2.376
2.365
H
B(MIDA)
Ph
11.69
1.00
2.01
2.07
2.08
2.14
3.02
9.5
9.0
8.5
8.0
7.5
7.0
6.5
6.0
5.5
5.0
4.5
4.0
3.5
3.0
2.5
2.0
1.5
f1 (ppm)

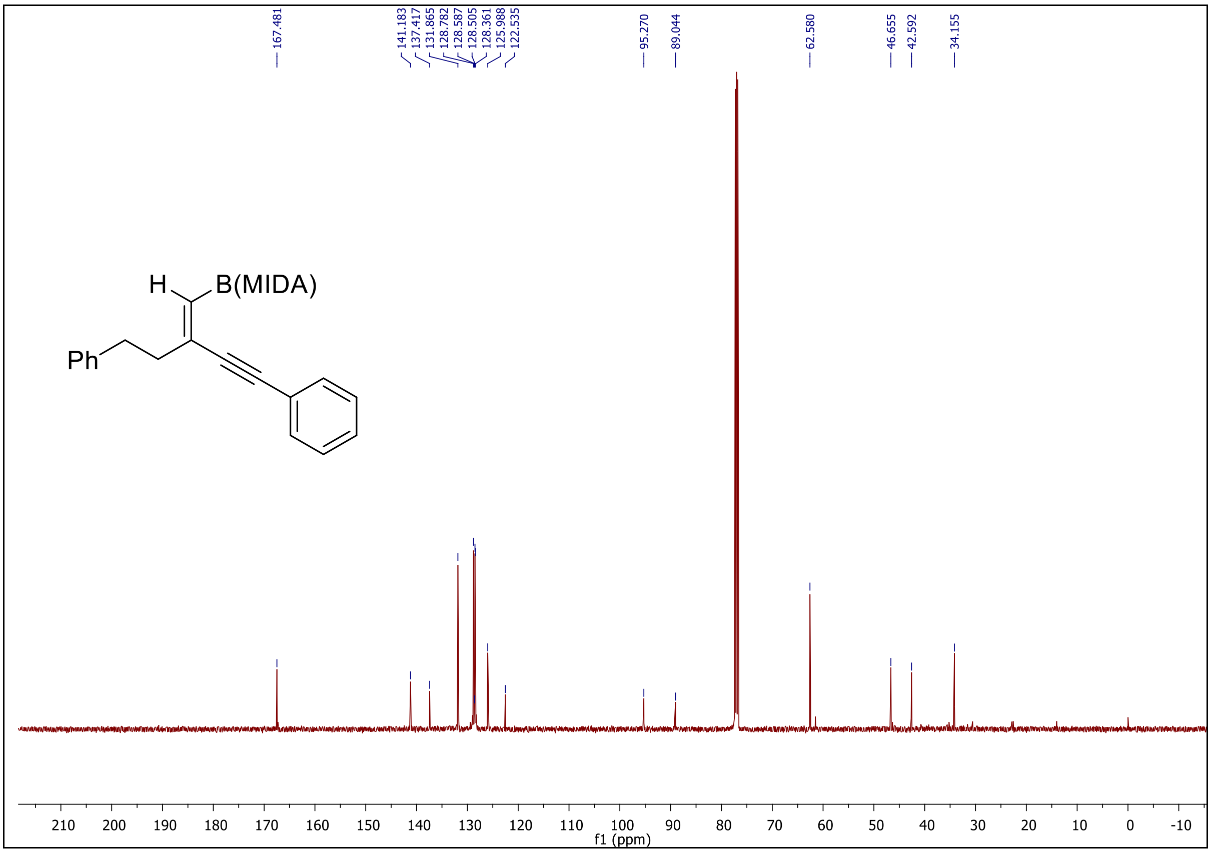
167.481
141.183
137.417
131.865
128.782
128.587
128.505
128.361
125.988
122.535
95.270
89.044
62.580
46.655
42.592
34.155
H
B(MIDA)
Ph
210 200 190 180 170 160 150 140 130 120 110 100 90 80 70 60 50 40 30 20 10 0 -10
f1 (ppm)

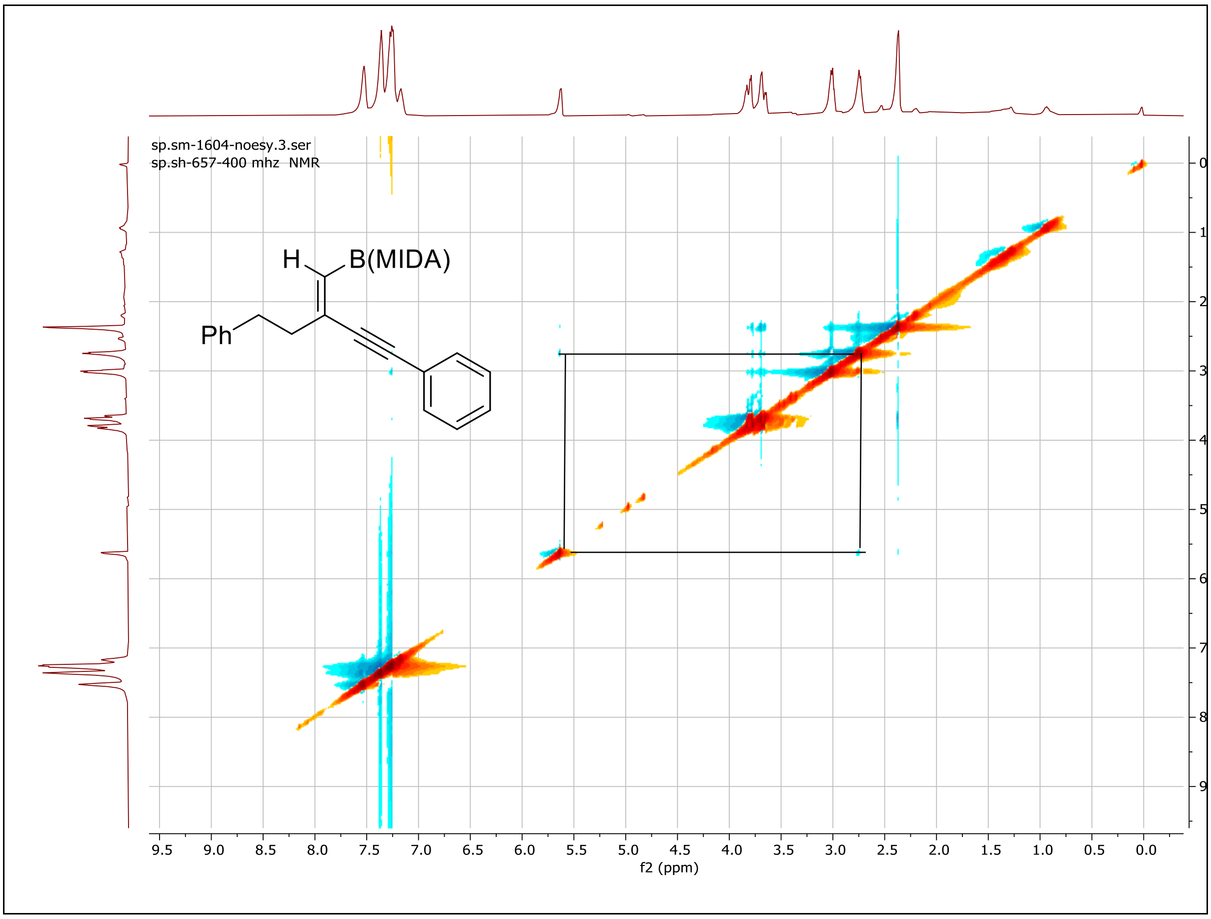
sp.sm-1604-noesy.3.ser
sp.sh-657-400 mhz NMR
H
B(MIDA)
Ph
9.5 9.0 8.5 8.0 7.5 7.0 6.5 6.0 5.5 5.0 4.5 4.0 3.5 3.0 2.5 2.0 1.5 1.0 0.5 0.0
f2 (ppm)
0 1 2 3 4 5 6 7 8 9
f1 (ppm)

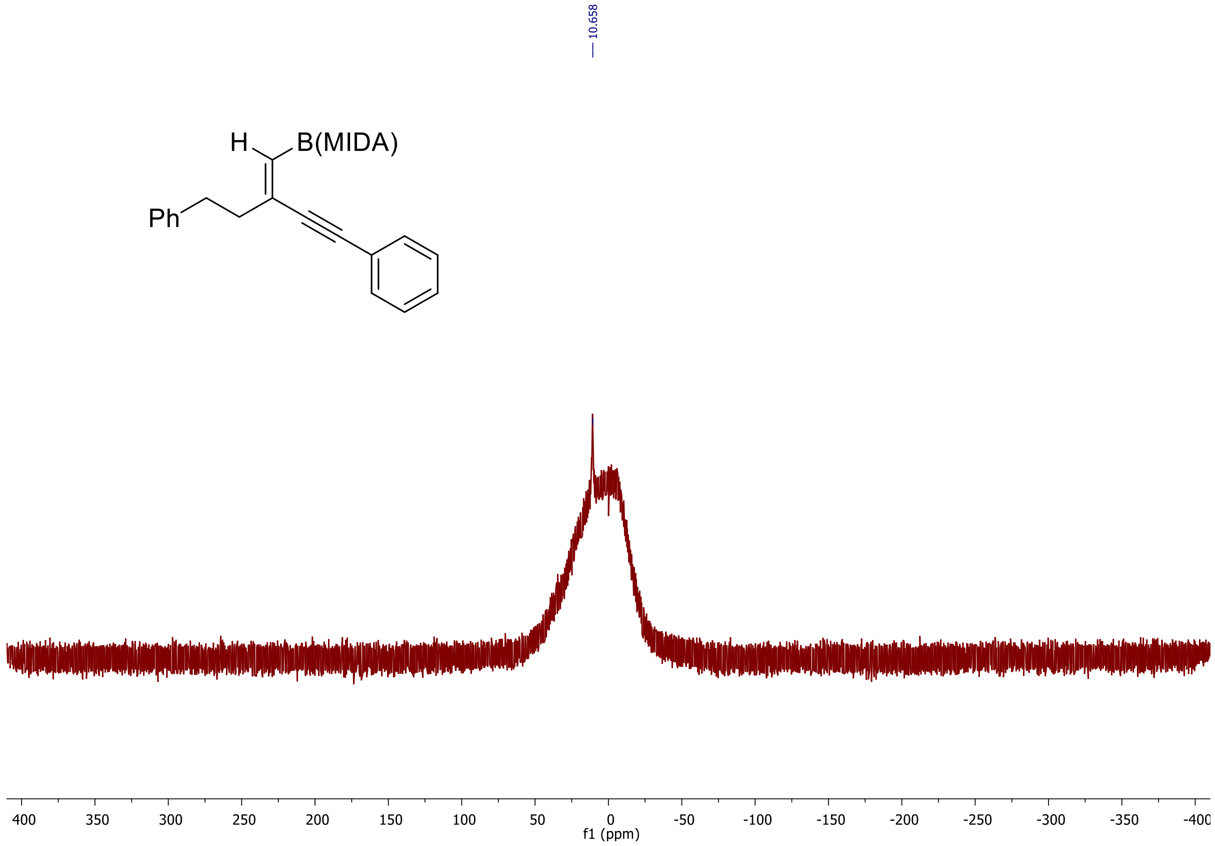

10.658
H
B(MIDA)
Ph
400
350
300
250
200
150
100
50
0
-50
-100
-150
-200
-250
-300
-350
-400
f1 (ppm)

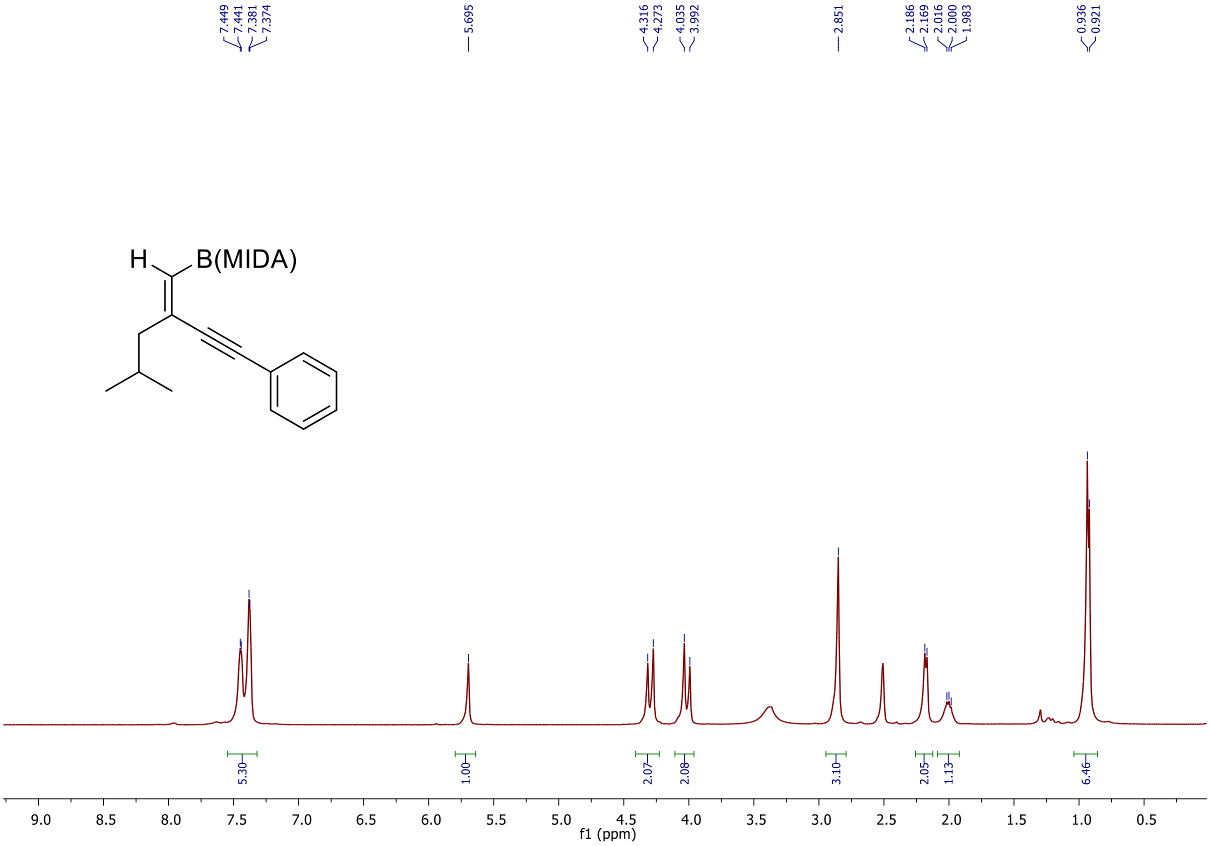
7.449
7.441
7.381
7.374
5.695
4.316
4.273
4.035
3.992
2.851
2.186
2.169
2.016
2.000
1.983
0.936
0.921
H
B(MIDA)
5.30
1.00
2.07
2.08
3.10
2.05
1.13
6.46
f1 (ppm)

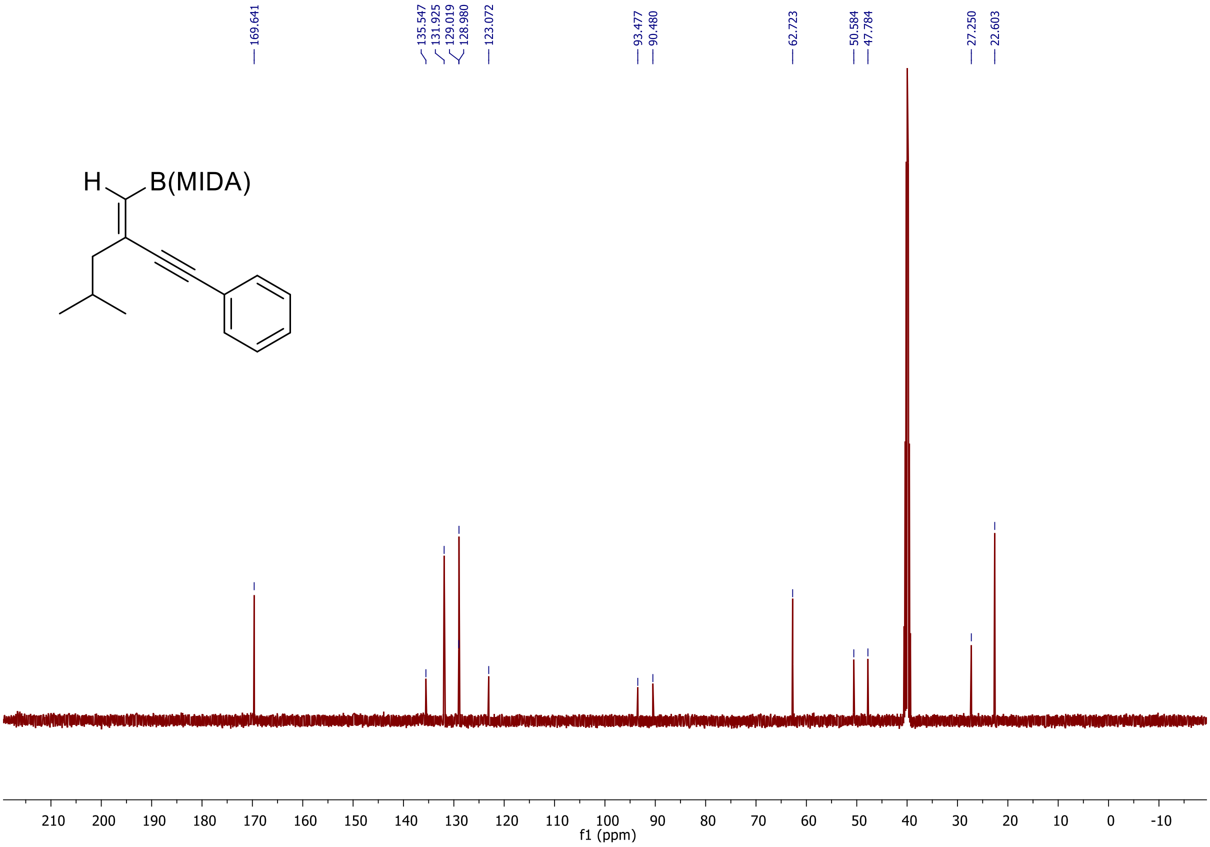
169.641
135.547
131.925
129.019
128.980
123.072
93.477
90.480
62.723
50.584
47.784
27.250
22.603
H
B(MIDA)
f1 (ppm)

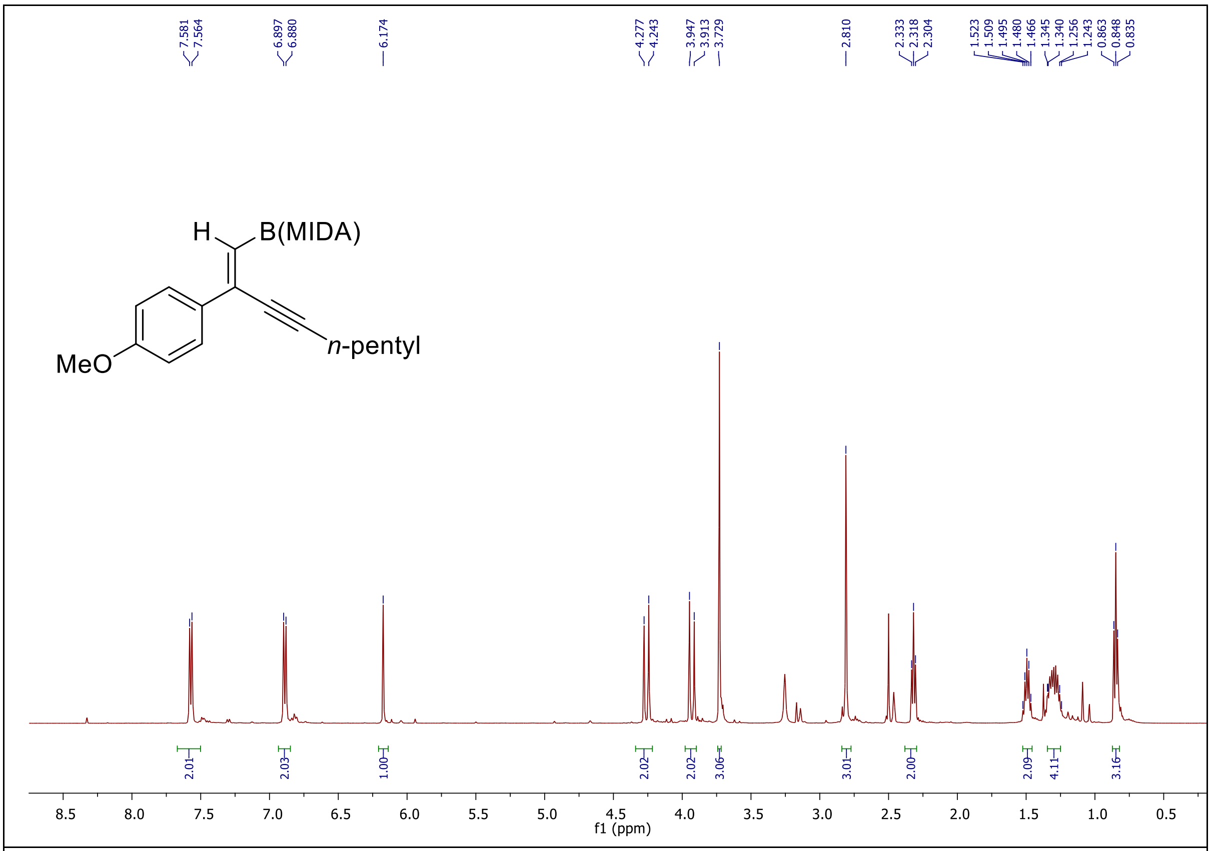
7.581
7.564
6.897
6.880
6.174
4.277
4.243
3.947
3.913
3.729
2.810
2.333
2.318
2.304
1.523
1.509
1.495
1.480
1.466
1.345
1.340
1.256
1.243
0.863
0.848
0.835
H
B(MIDA)
MeO
n-pentyl
2.01
2.03
1.00
2.02
2.02
3.06
3.01
2.00
2.09
4.11
3.16
f1 (ppm)

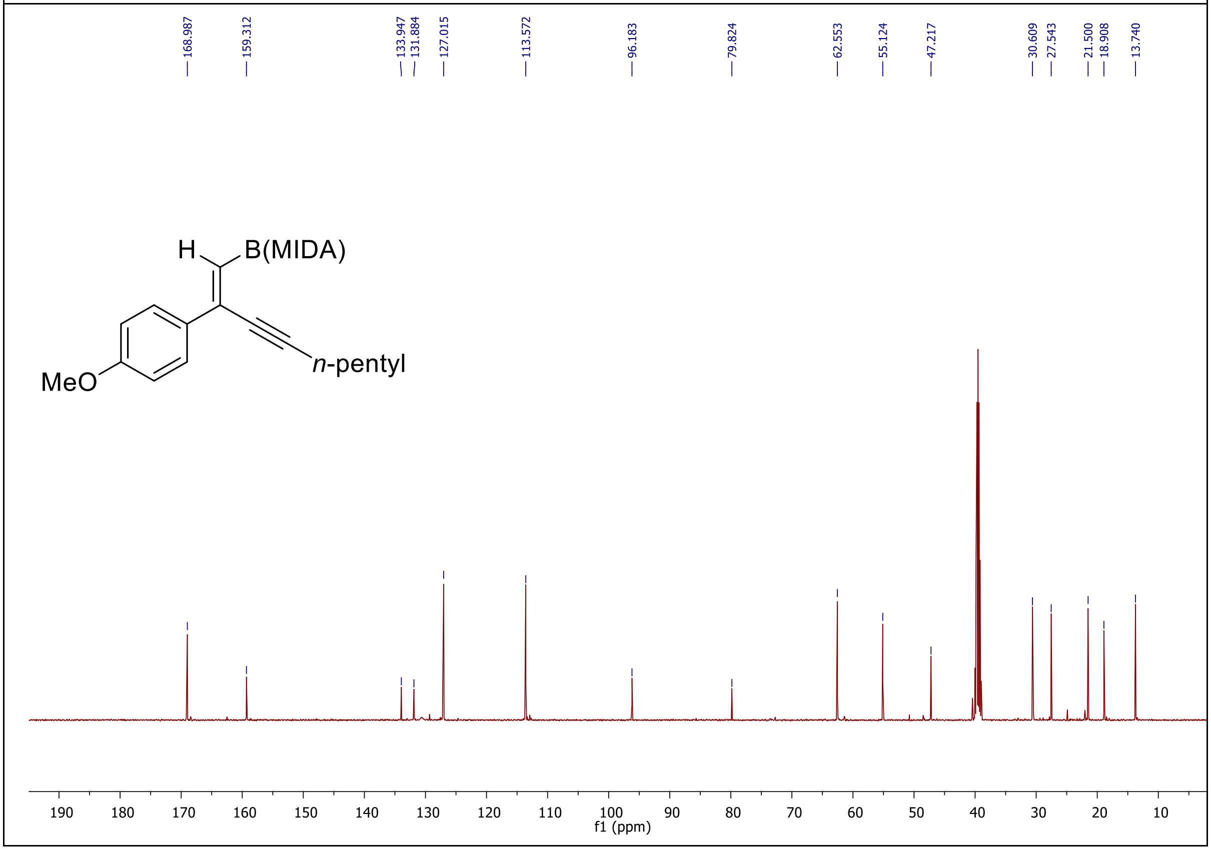
168.987
159.312
133.947
131.884
127.015
113.572
96.183
79.824
62.553
55.124
47.217
30.609
27.543
21.500
18.908
13.740
H
B(MIDA)
MeO
n-pentyl
f1 (ppm)

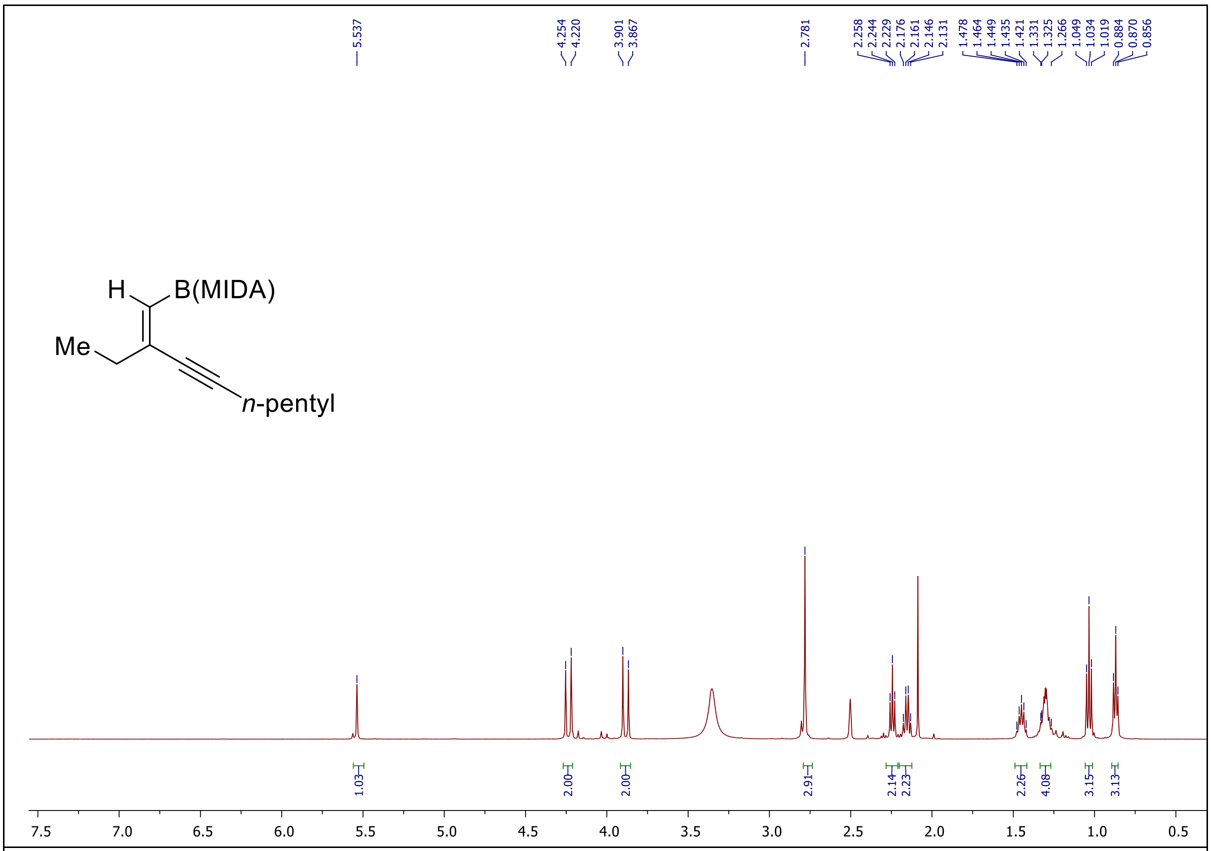
5.537
4.254
4.220
3.901
3.867
2.781
2.258
2.244
2.229
2.176
2.161
2.146
2.131
1.478
1.464
1.449
1.435
1.421
1.331
1.325
1.266
1.049
1.034
1.019
0.884
0.870
0.856
H
B(MIDA)
Me
n-pentyl
1.03
2.00
2.00
2.91
2.14
2.23
2.26
4.08
3.15
3.13
7.5
7.0
6.5
6.0
5.5
5.0
4.5
4.0
3.5
3.0
2.5
2.0
1.5
1.0
0.5

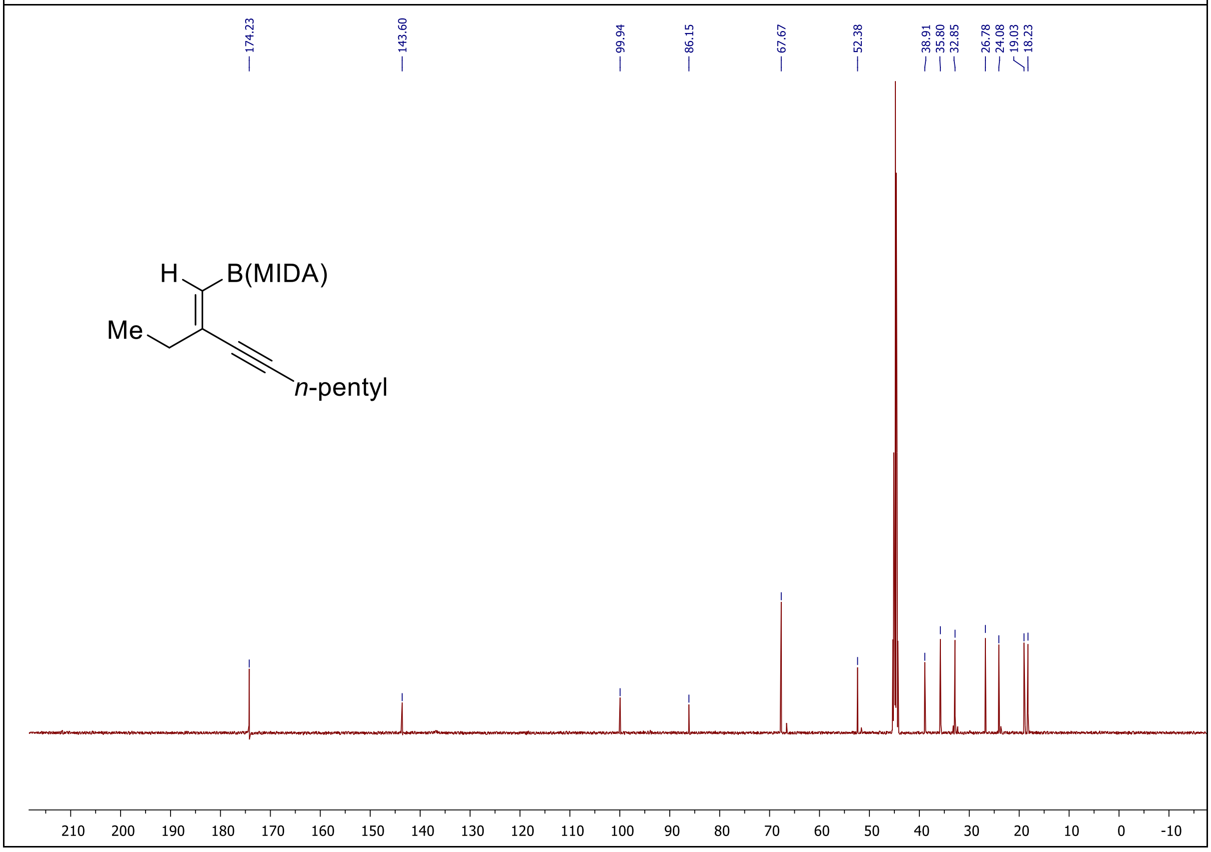
174.23
143.60
99.94
86.15
67.67
52.38
38.91
35.80
32.85
26.78
24.08
19.03
18.23
H
B(MIDA)
Me
n-pentyl
210
200
190
180
170
160
150
140
130
120
110
100
90
80
70
60
50
40
30
20
10
0
-10

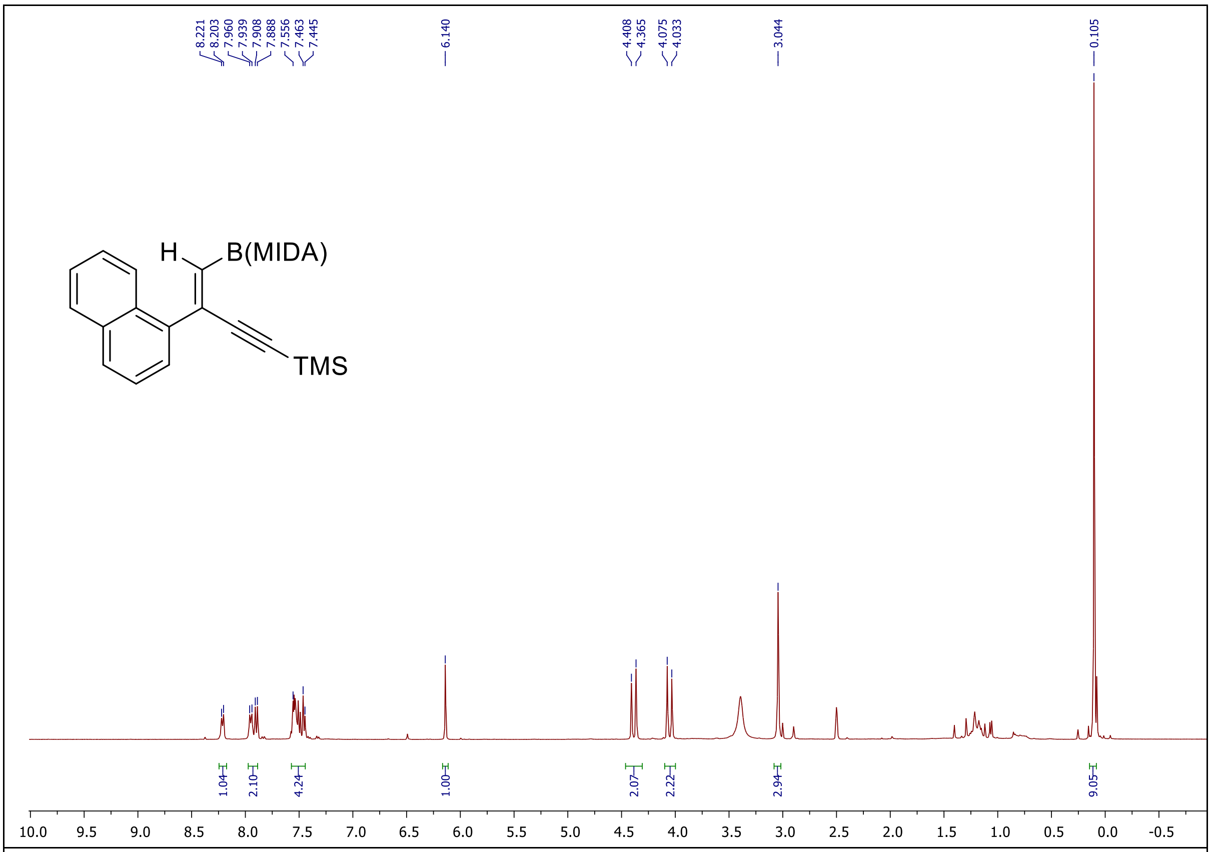
8.221
8.203
7.960
7.939
7.908
7.888
7.556
7.463
7.445
6.140
4.408
4.365
4.075
4.033
3.044
0.105
H
B(MIDA)
TMS
1.04
2.10
4.24
1.00
2.07
2.22
2.94
9.05
10.0 9.5 9.0 8.5 8.0 7.5 7.0 6.5 6.0 5.5 5.0 4.5 4.0 3.5 3.0 2.5 2.0 1.5 1.0 0.5 0.0 -0.5

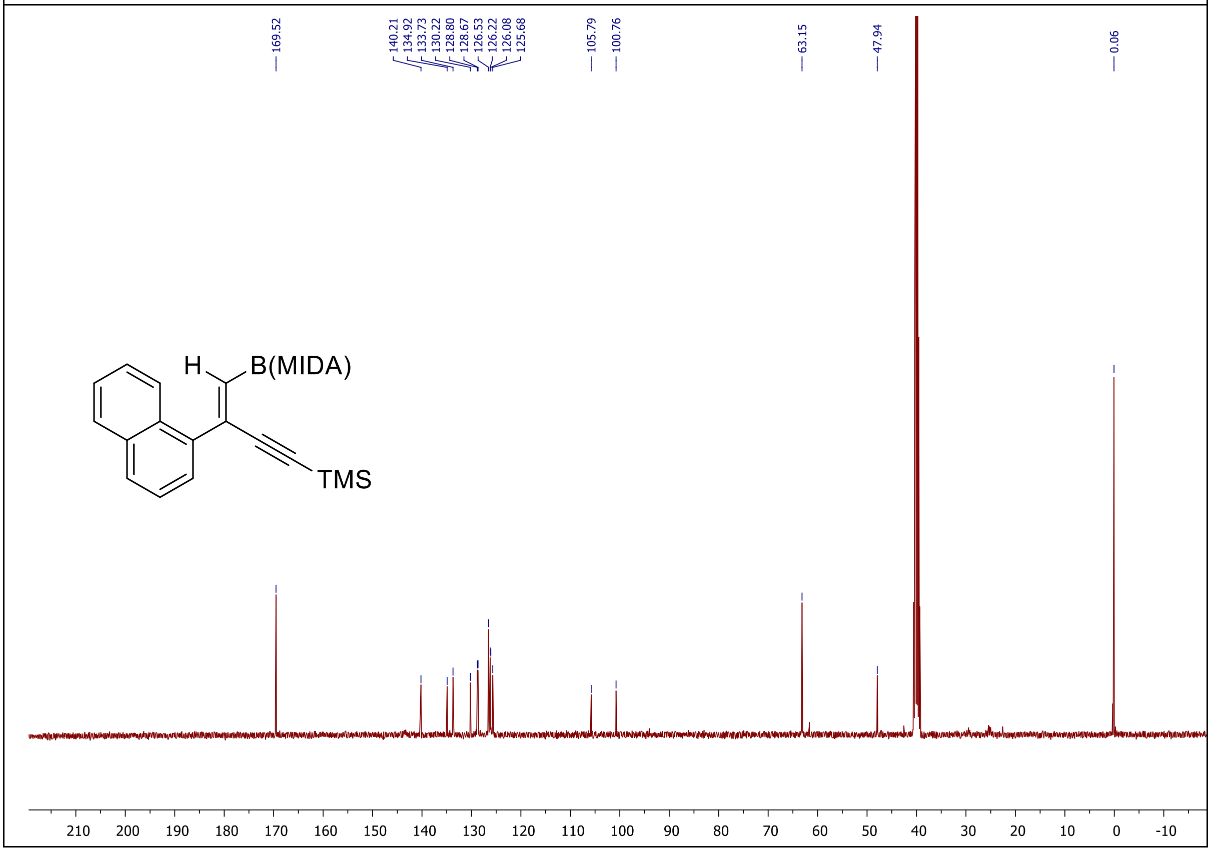
169.52
140.21
134.92
133.73
130.22
128.80
128.67
126.53
126.22
126.08
125.68
105.79
100.76
63.15
47.94
0.06
H
B(MIDA)
TMS
210 200 190 180 170 160 150 140 130 120 110 100 90 80 70 60 50 40 30 20 10 0 -10

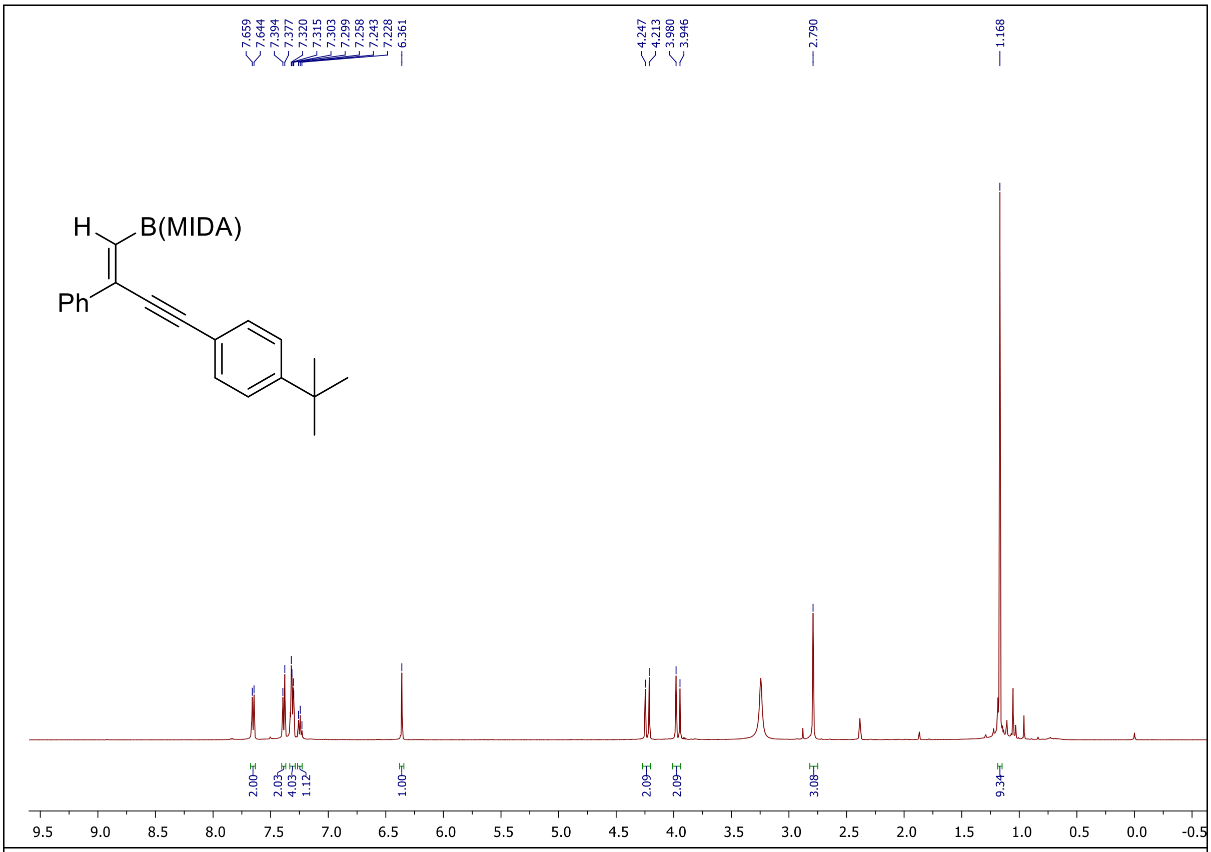

7.659
7.644
7.394
7.377
7.320
7.315
7.303
7.299
7.258
7.243
7.228
6.361
4.247
4.213
3.980
3.946
2.790
1.168
H
B(MIDA)
Ph
2.00
2.03
4.03
1.12
1.00
2.09
2.09
3.08
9.34
9.5 9.0 8.5 8.0 7.5 7.0 6.5 6.0 5.5 5.0 4.5 4.0 3.5 3.0 2.5 2.0 1.5 1.0 0.5 0.0 -0.5

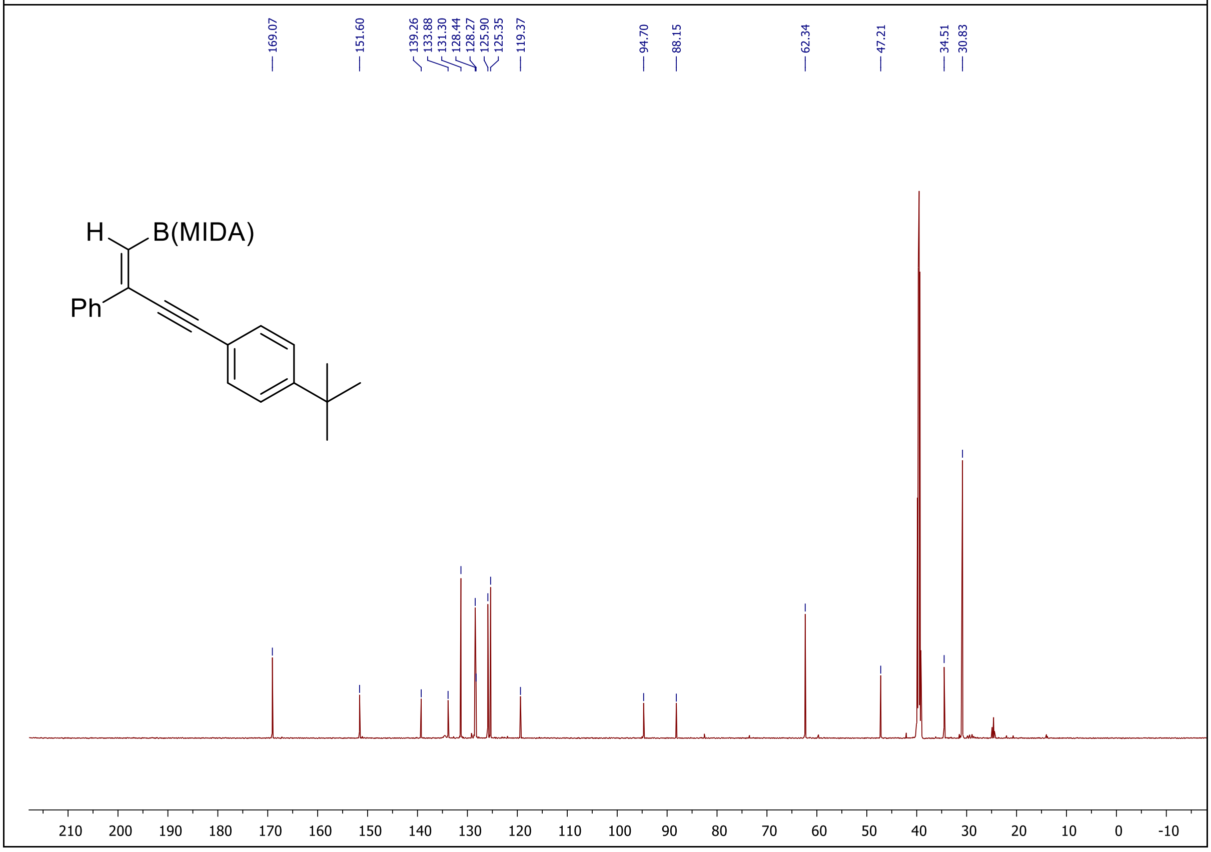

169.07
151.60
139.26
133.88
131.30
128.44
128.27
125.90
125.35
119.37
94.70
88.15
62.34
47.21
34.51
30.83
H
B(MIDA)
Ph
210 200 190 180 170 160 150 140 130 120 110 100 90 80 70 60 50 40 30 20 10 0 -10

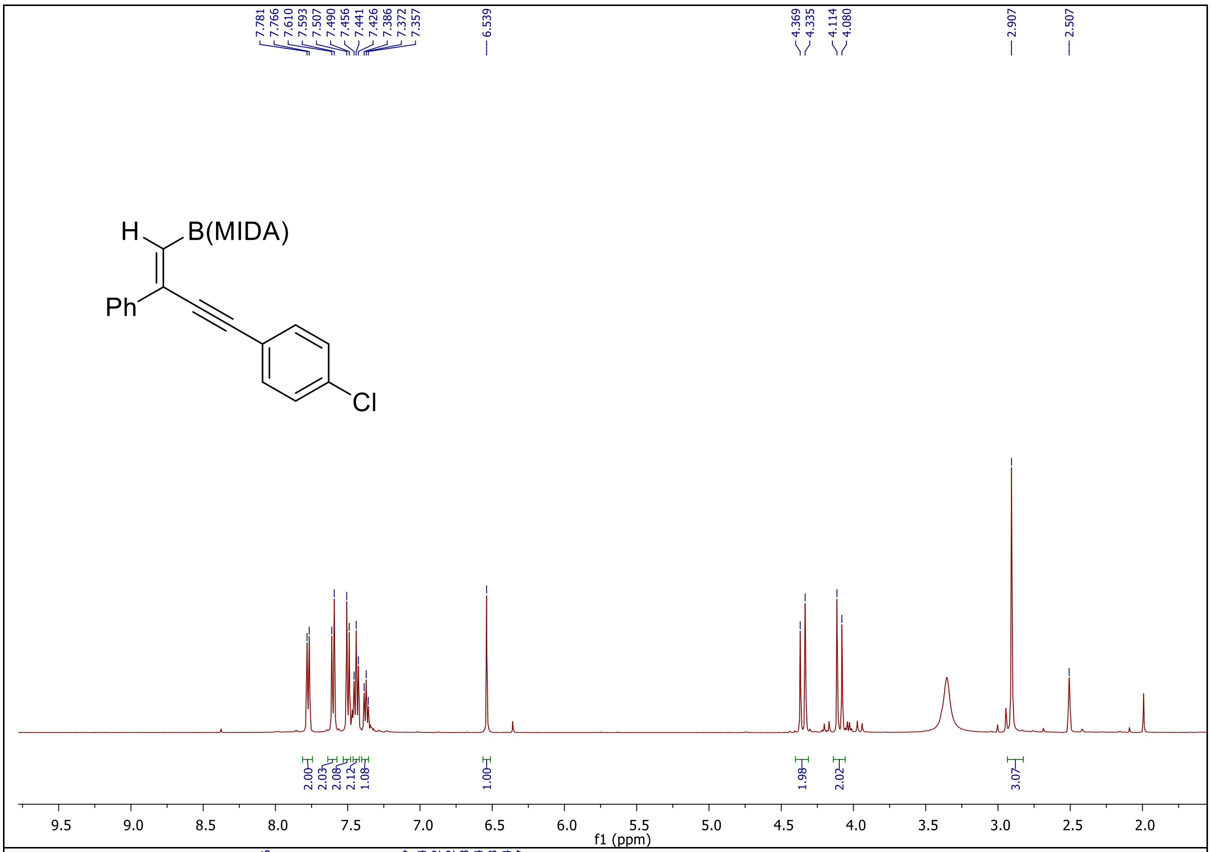
7.781
7.766
7.610
7.593
7.507
7.490
7.456
7.441
7.426
7.386
7.372
7.357
6.539
4.369
4.335
4.114
4.080
2.907
2.507
H
B(MIDA)
Ph
Cl
2.00
2.03
2.08
2.12
1.08
1.00
1.98
2.02
3.07
9.5
9.0
8.5
8.0
7.5
7.0
6.5
6.0
5.5
5.0
4.5
4.0
3.5
3.0
2.5
2.0
f1 (ppm)

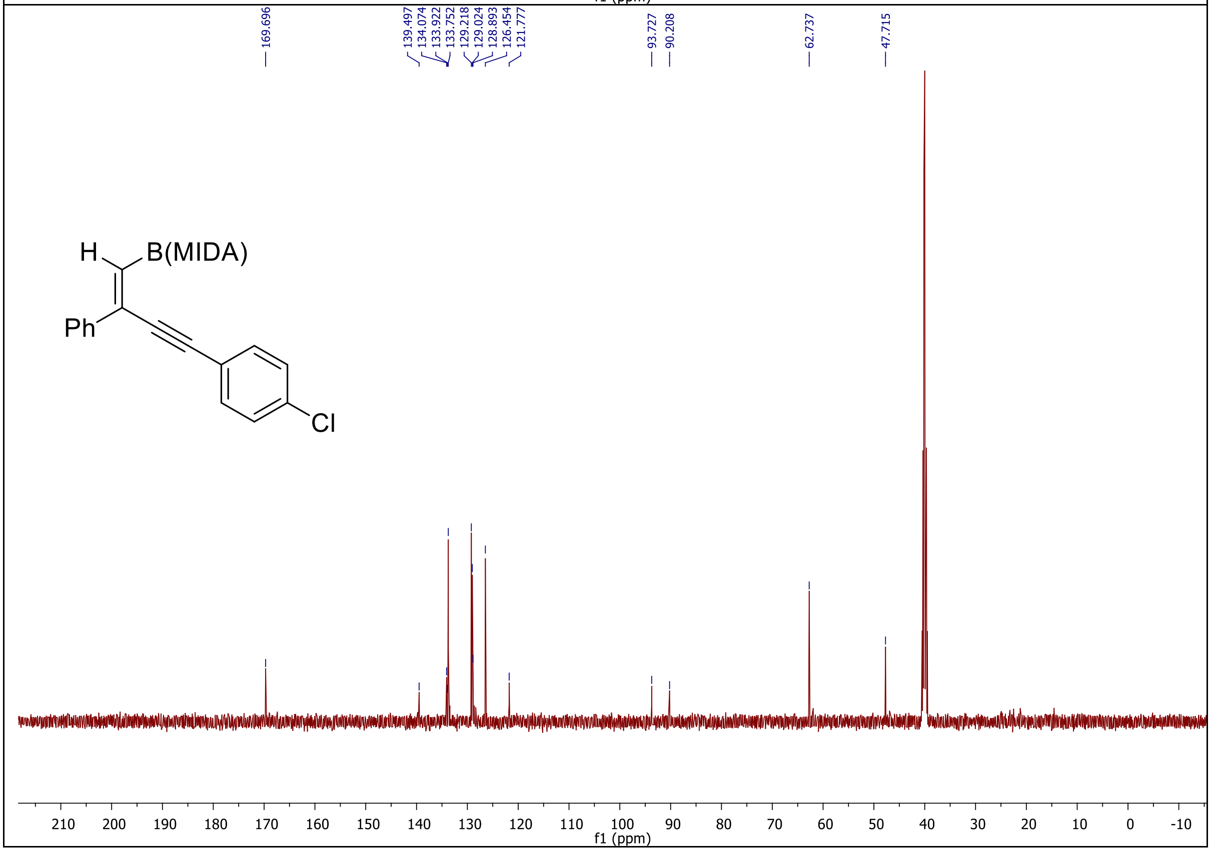
169.696
139.497
134.074
133.922
133.752
129.218
129.024
128.893
126.454
121.777
93.727
90.208
62.737
47.715
H
B(MIDA)
Ph
Cl
210
200
190
180
170
160
150
140
130
120
110
100
90
80
70
60
50
40
30
20
10
0
-10
f1 (ppm)

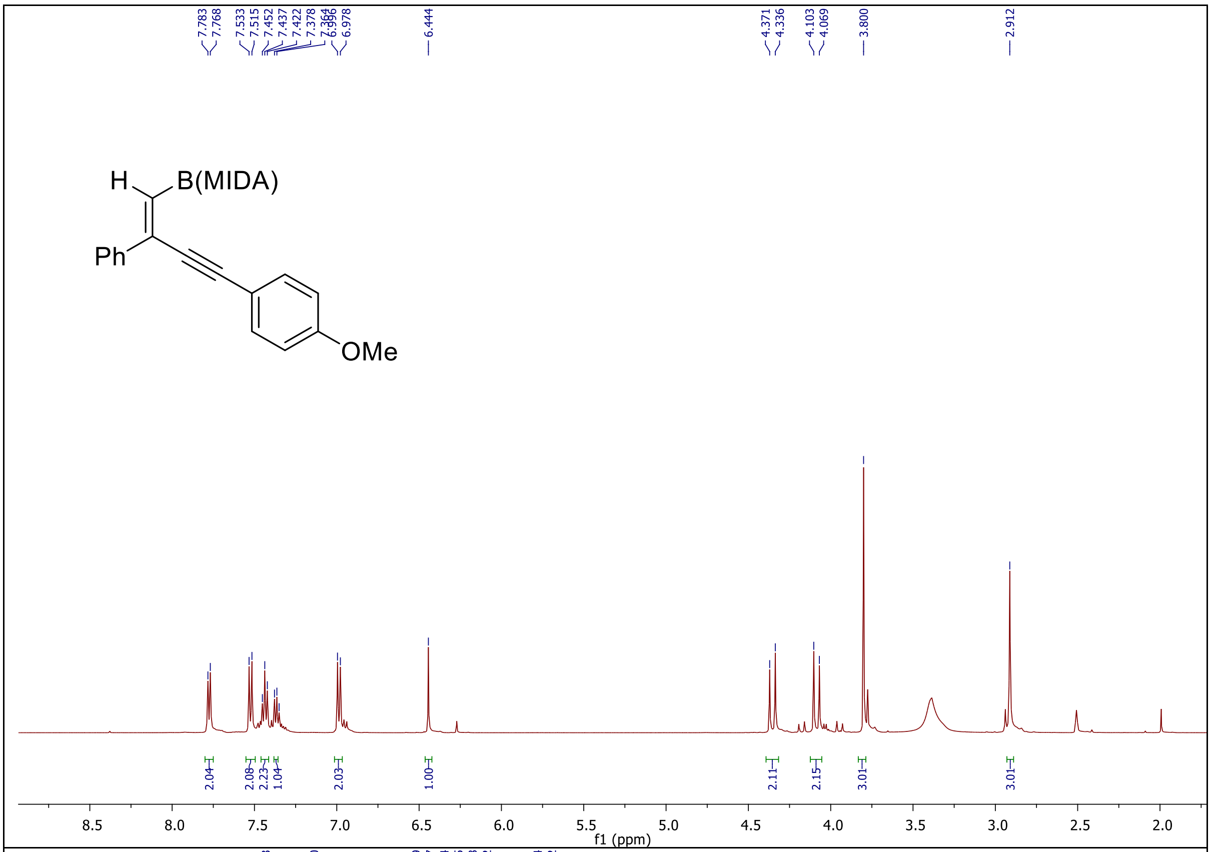
7.783
7.768
7.533
7.515
7.452
7.437
7.422
7.378
6.996
6.978
6.444
4.371
4.336
4.103
4.069
3.800
2.912
H
B(MIDA)
Ph
OMe
2.04
2.08
2.23
1.04
2.03
1.00
2.11
2.15
3.01
3.01
f1 (ppm)

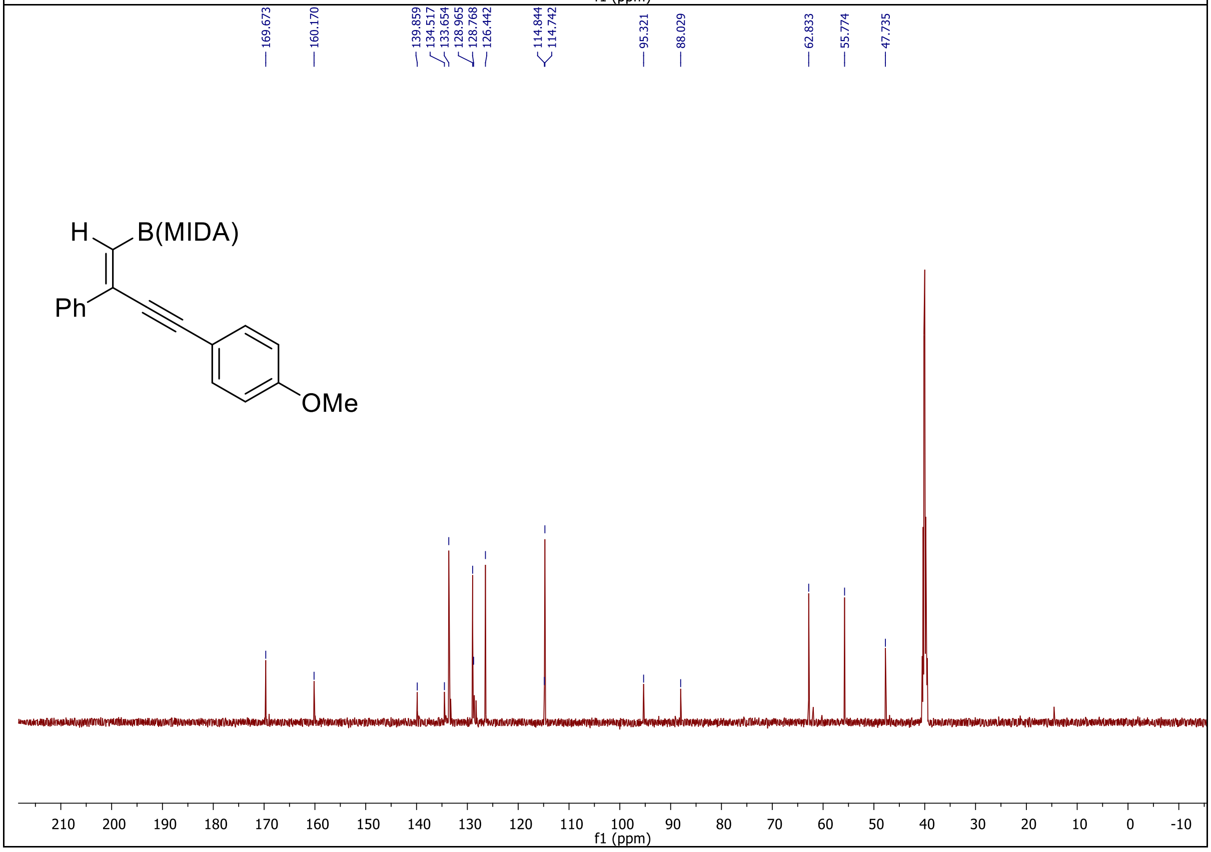
169.673
160.170
139.859
134.517
133.654
128.965
128.768
126.442
114.844
114.742
95.321
88.029
62.833
55.774
47.735
H
B(MIDA)
Ph
OMe
f1 (ppm)

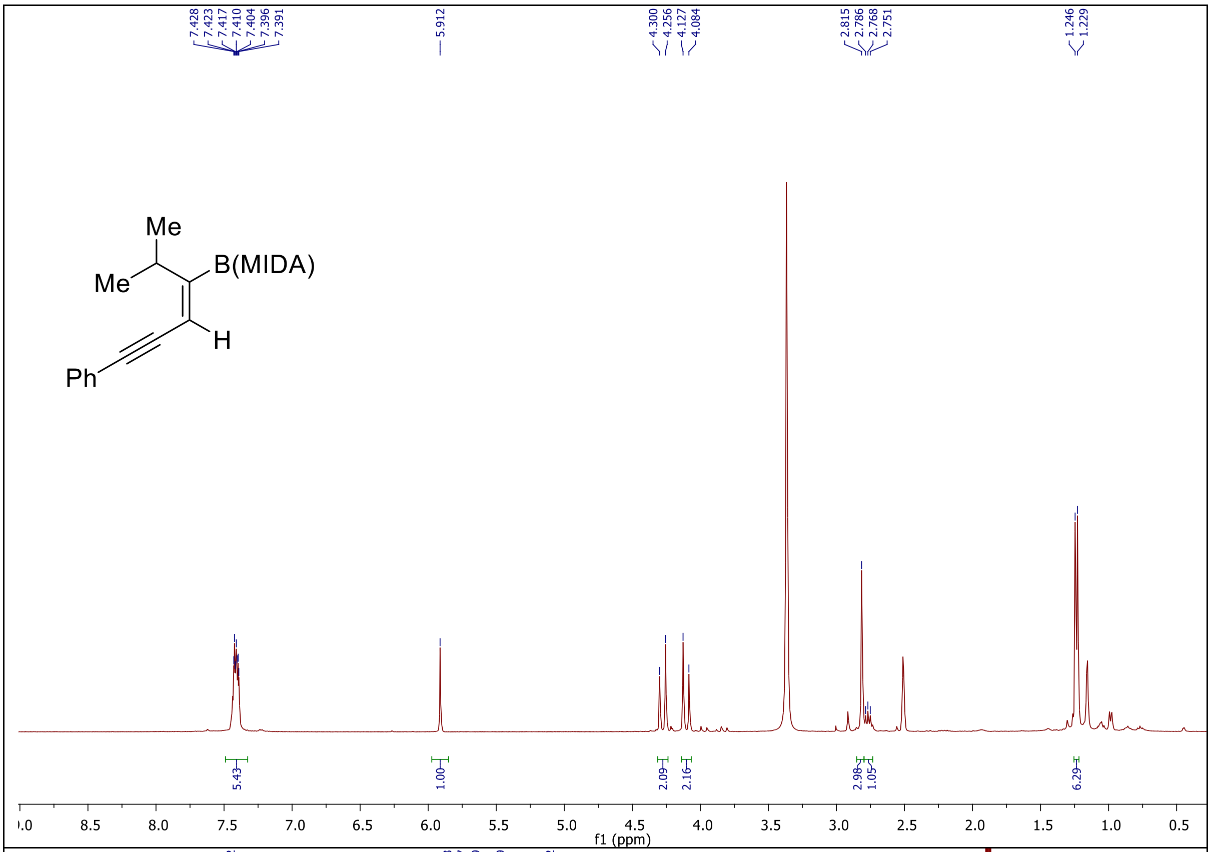

Me
Me
B(MIDA)
H
Ph
7.428
7.423
7.417
7.410
7.404
7.396
7.391
5.912
4.300
4.256
4.127
4.084
2.815
2.786
2.768
2.751
1.246
1.229
5.43
1.00
2.09
2.16
2.98
1.05
6.29
f1 (ppm)

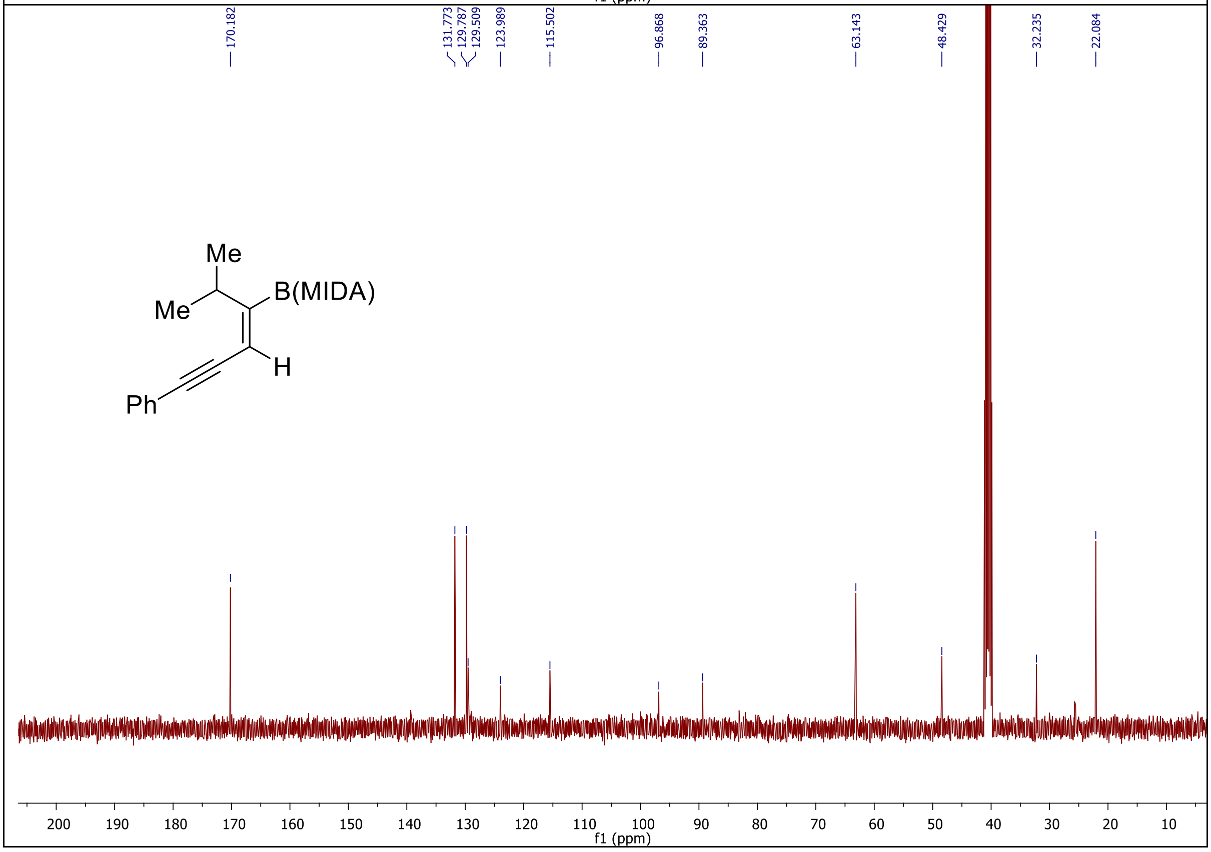

Me
Me
B(MIDA)
H
Ph
170.182
131.773
129.787
129.509
123.989
115.502
96.868
89.363
63.143
48.429
32.235
22.084
f1 (ppm)

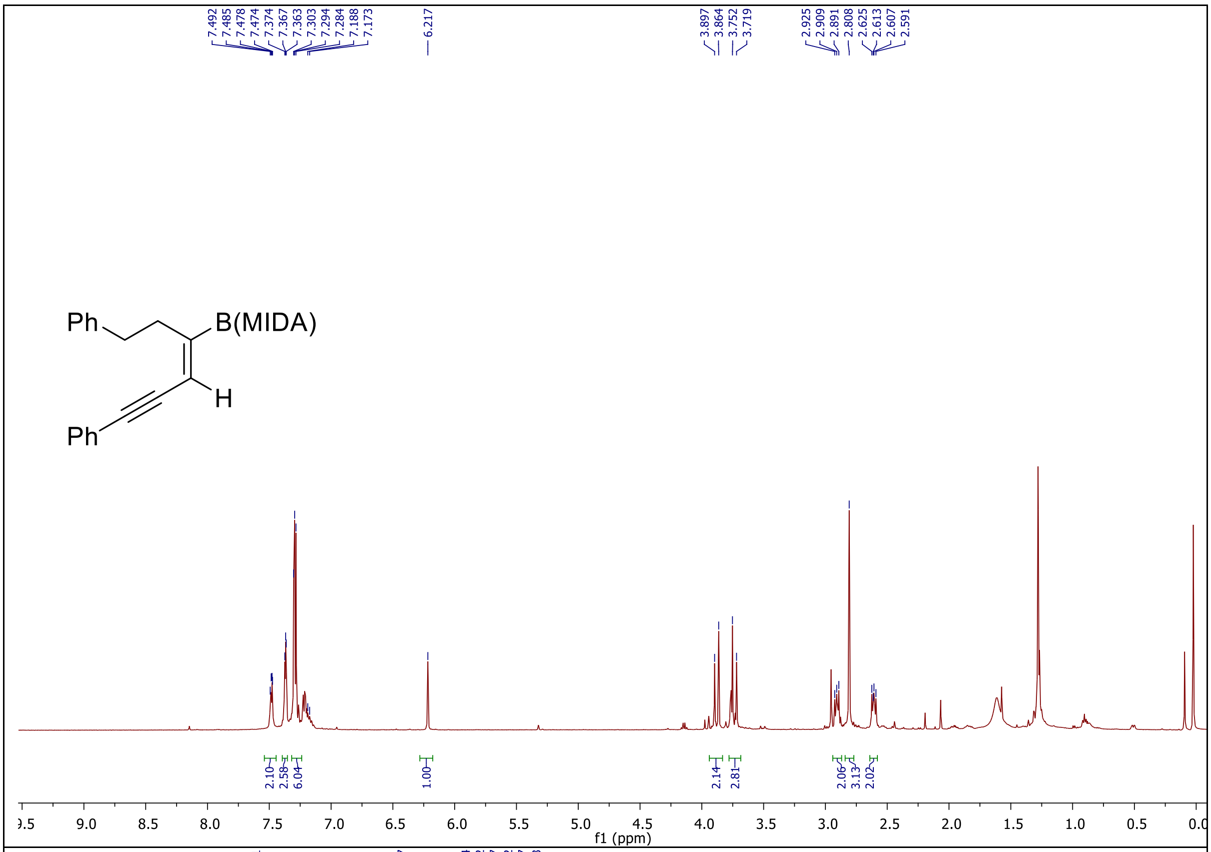

7.492
7.485
7.478
7.474
7.374
7.367
7.363
7.303
7.294
7.284
7.188
7.173
6.217
3.897
3.864
3.752
3.719
2.925
2.909
2.891
2.808
2.625
2.613
2.607
2.591
Ph
B(MIDA)
H
Ph
2.10
2.58
6.04
1.00
2.14
2.81
2.06
3.13
2.02
f1 (ppm)

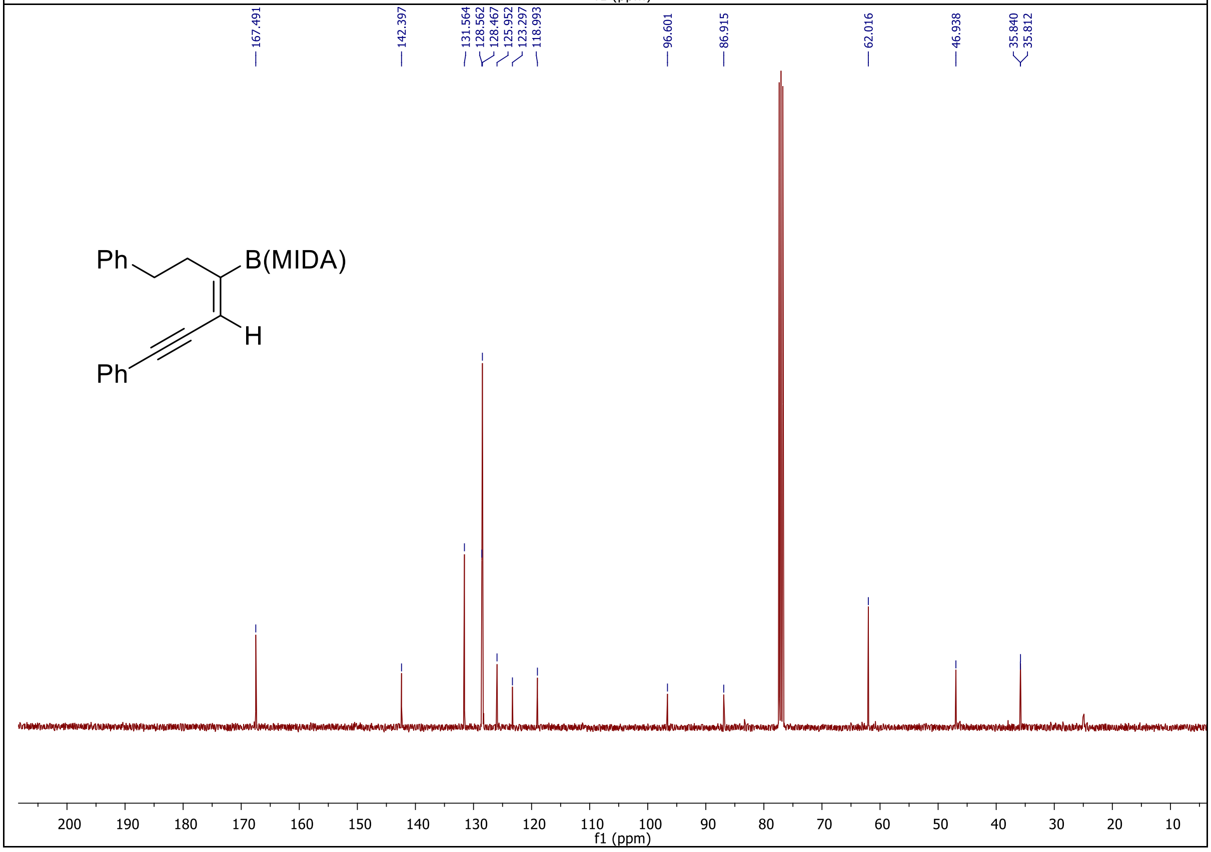

167.491
142.397
131.564
128.562
128.467
125.952
123.297
118.993
96.601
86.915
62.016
46.938
35.840
35.812
Ph
B(MIDA)
H
Ph
f1 (ppm)

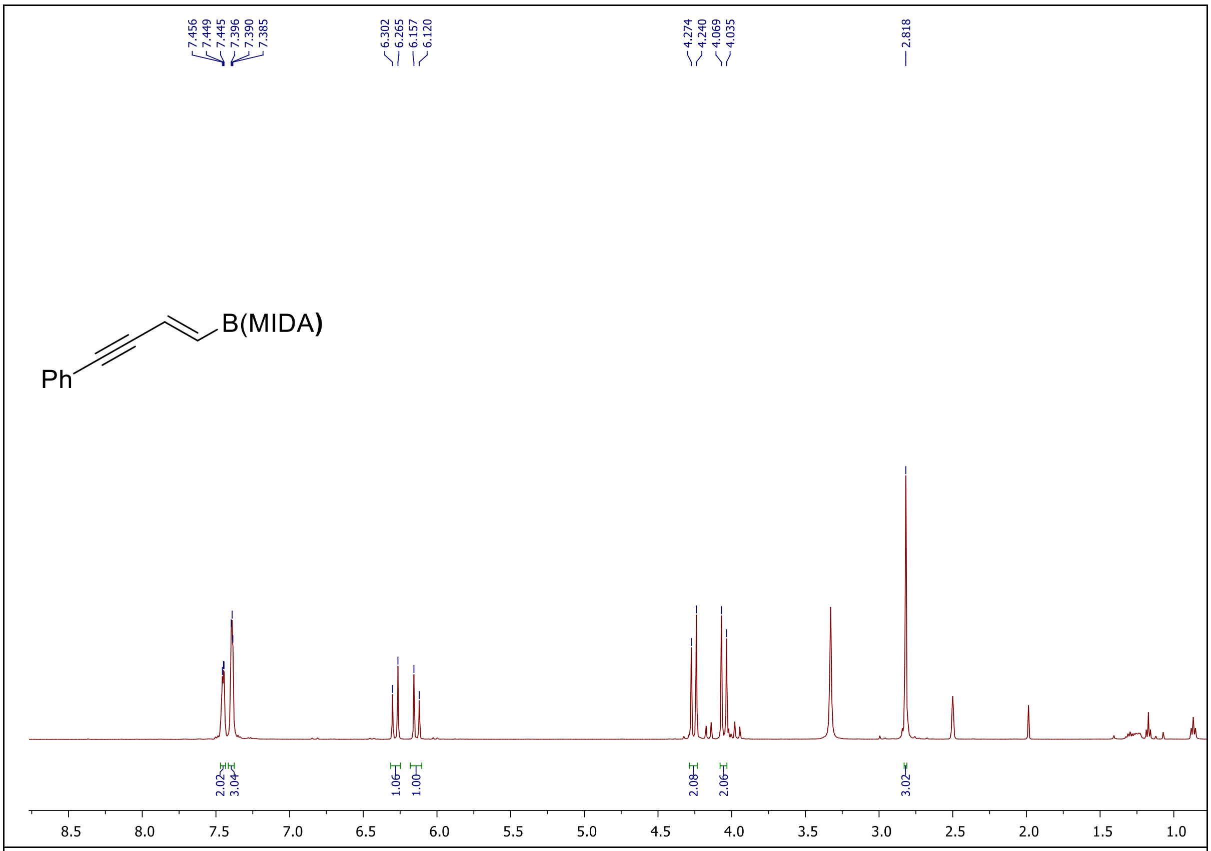
7.456
7.449
7.445
7.396
7.390
7.385
6.302
6.265
6.157
6.120
4.274
4.240
4.069
4.035
2.818
B(MIDA)
Ph
2.02
3.04
1.06
1.00
2.08
2.06
3.02
8.5
8.0
7.5
7.0
6.5
6.0
5.5
5.0
4.5
4.0
3.5
3.0
2.5
2.0
1.5
1.0

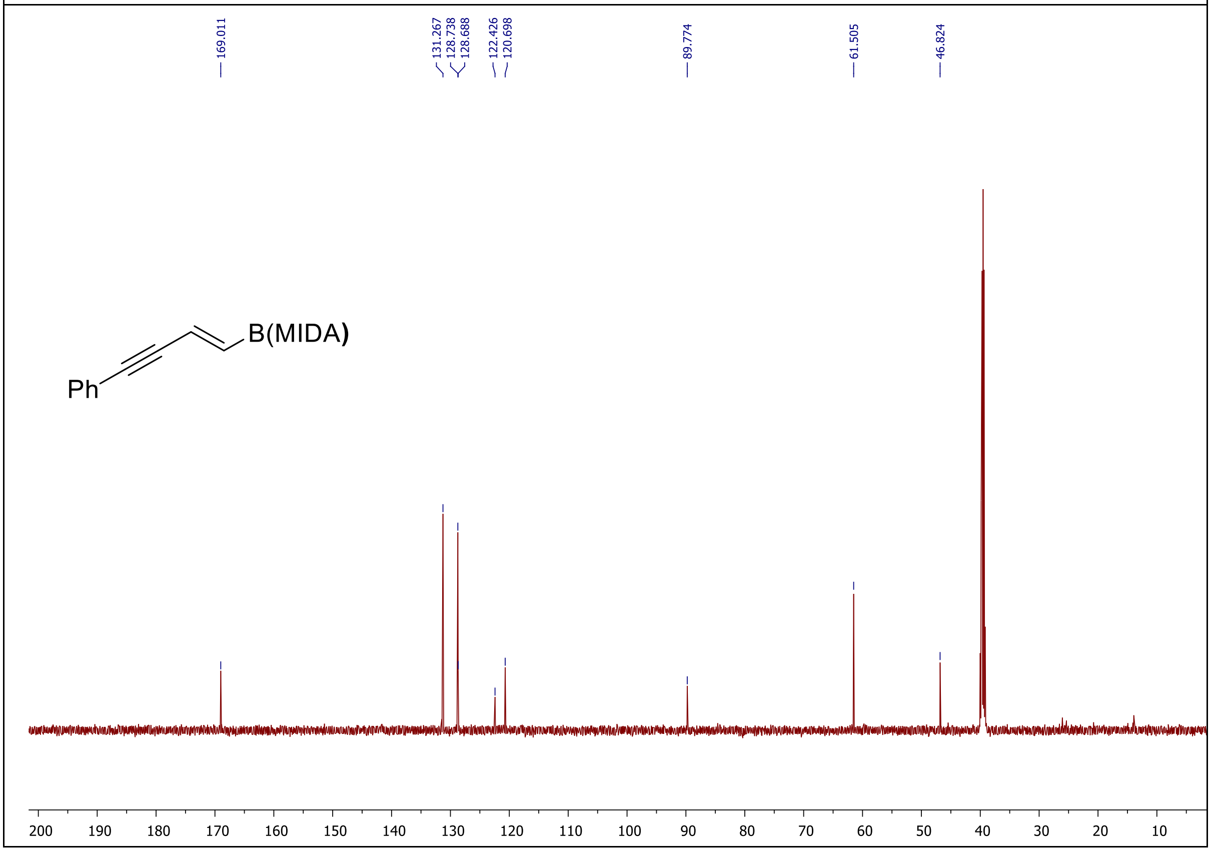
169.011
131.267
128.738
128.688
122.426
120.698
89.774
61.505
46.824
B(MIDA)
Ph
200
190
180
170
160
150
140
130
120
110
100
90
80
70
60
50
40
30
20
10

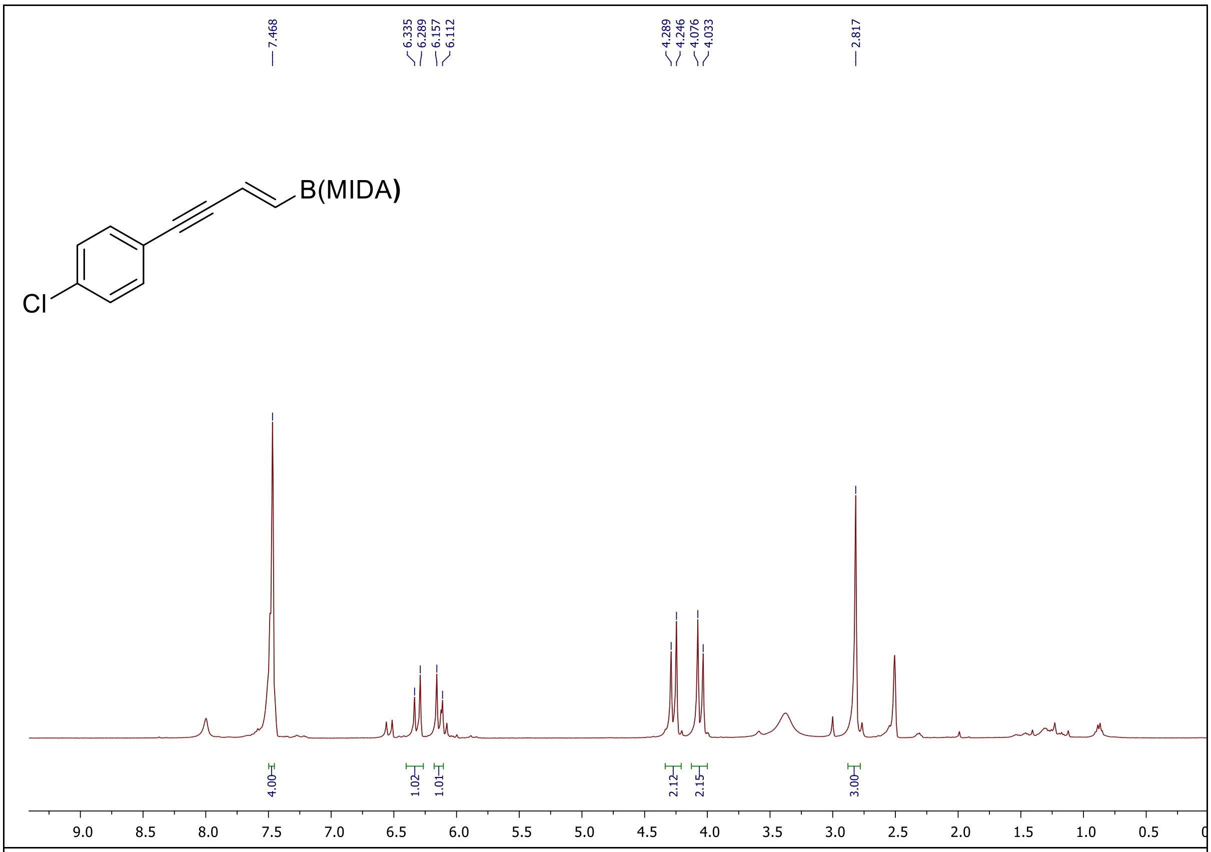
7.468
6.335
6.289
6.157
6.112
4.289
4.246
4.076
4.033
2.817
B(MIDA)
Cl
4.00
1.02
1.01
2.12
2.15
3.00
9.0 8.5 8.0 7.5 7.0 6.5 6.0 5.5 5.0 4.5 4.0 3.5 3.0 2.5 2.0 1.5 1.0 0.5

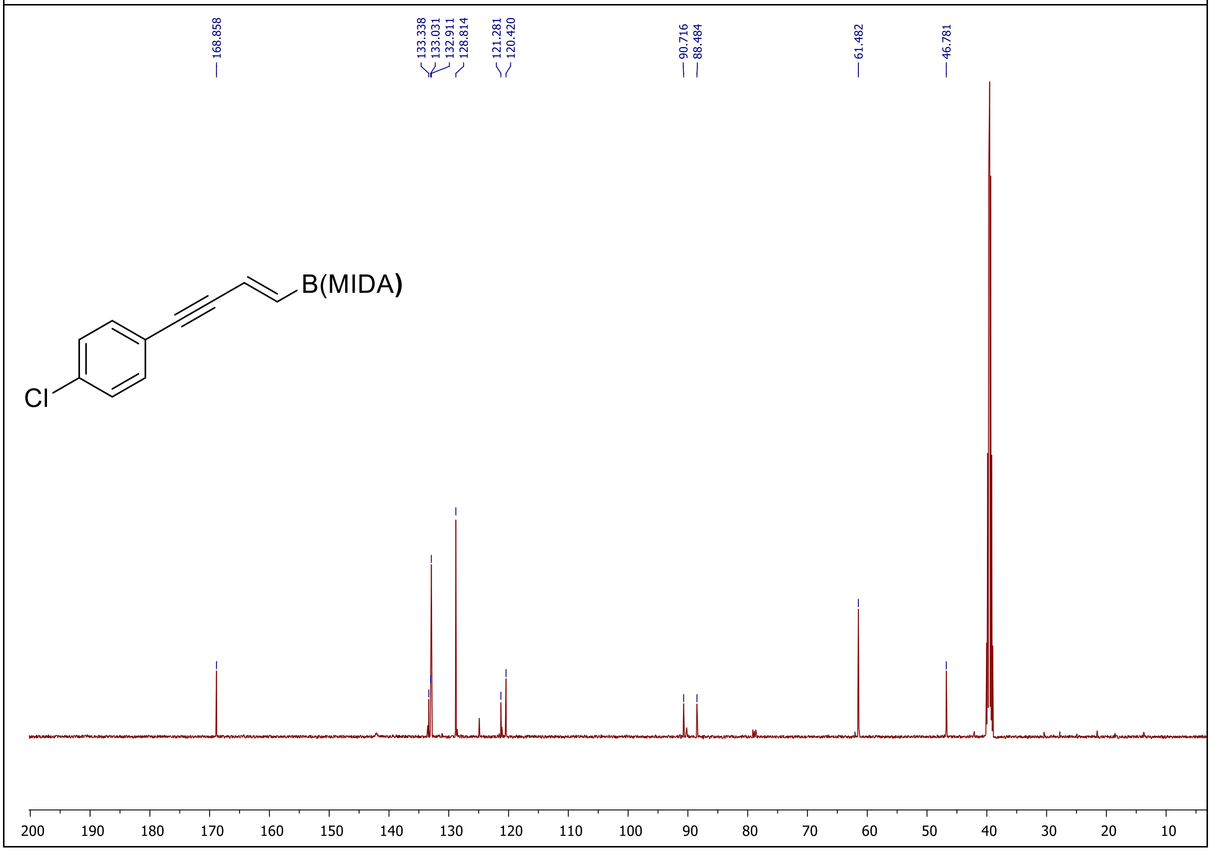
168.858
133.338
133.031
132.911
128.814
121.281
120.420
90.716
88.484
61.482
46.781
B(MIDA)
Cl
200 190 180 170 160 150 140 130 120 110 100 90 80 70 60 50 40 30 20 10

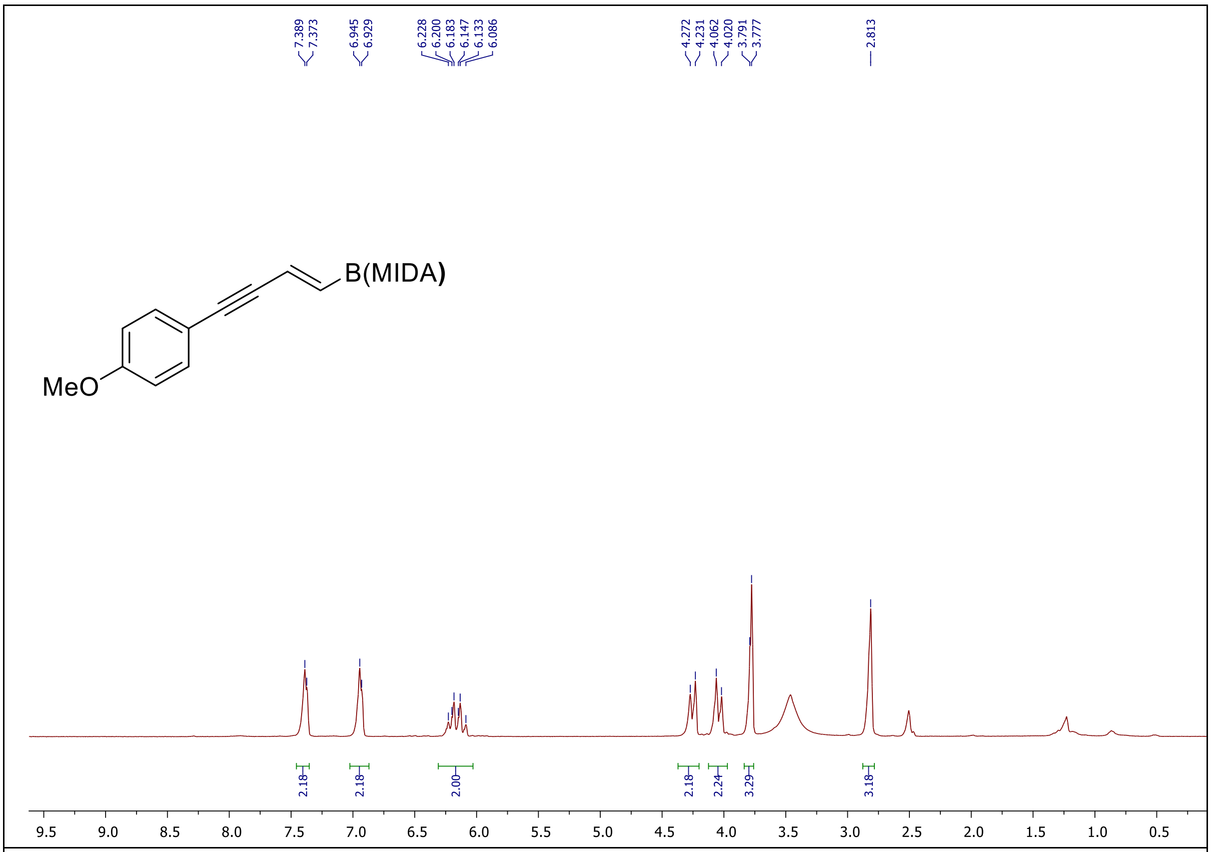

7.389
7.373
6.945
6.929
6.228
6.200
6.183
6.147
6.133
6.086
4.272
4.231
4.062
4.020
3.791
3.777
2.813
B(MIDA)
MeO
2.18
2.18
2.00
2.18
2.24
3.29
3.18
9.5
9.0
8.5
8.0
7.5
7.0
6.5
6.0
5.5
5.0
4.5
4.0
3.5
3.0
2.5
2.0
1.5
1.0
0.5

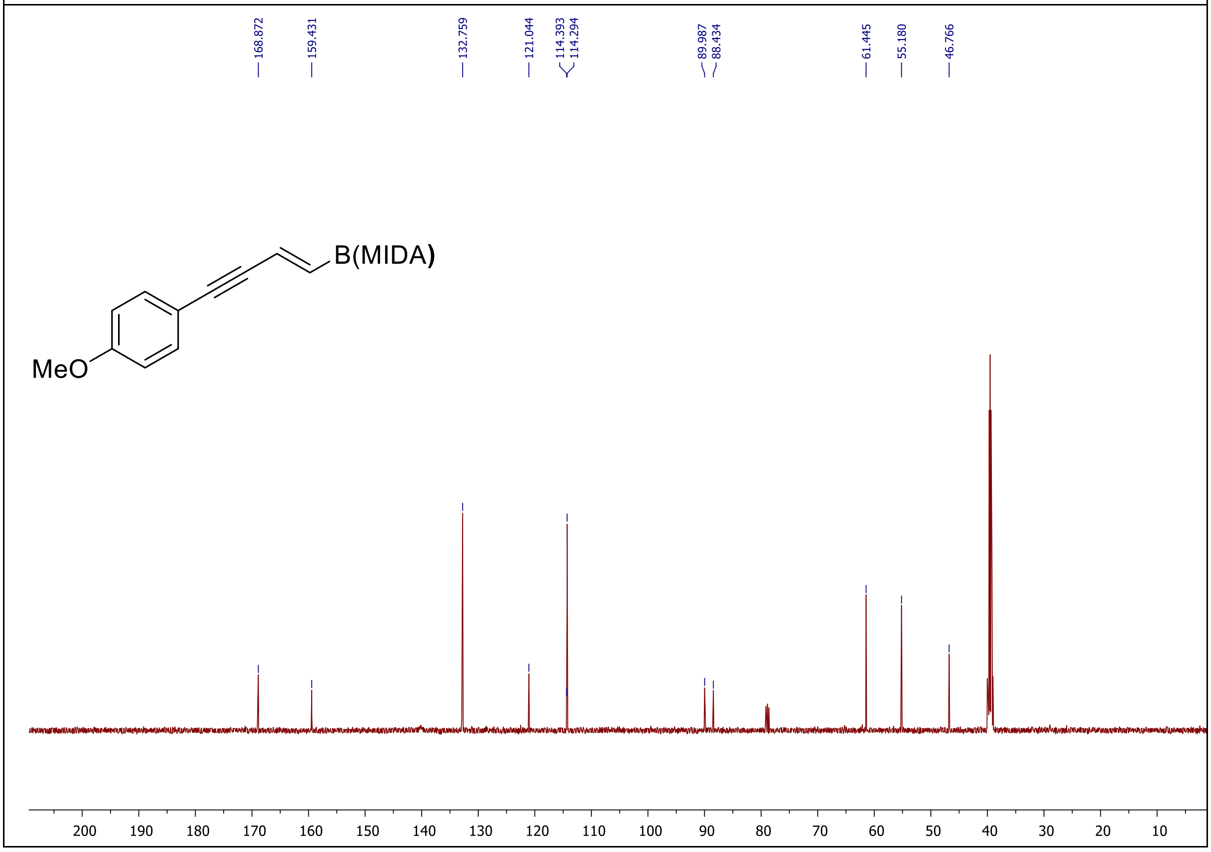

168.872
159.431
132.759
121.044
114.393
114.294
89.987
88.434
61.445
55.180
46.766
B(MIDA)
MeO
200
190
180
170
160
150
140
130
120
110
100
90
80
70
60
50
40
30
20
10

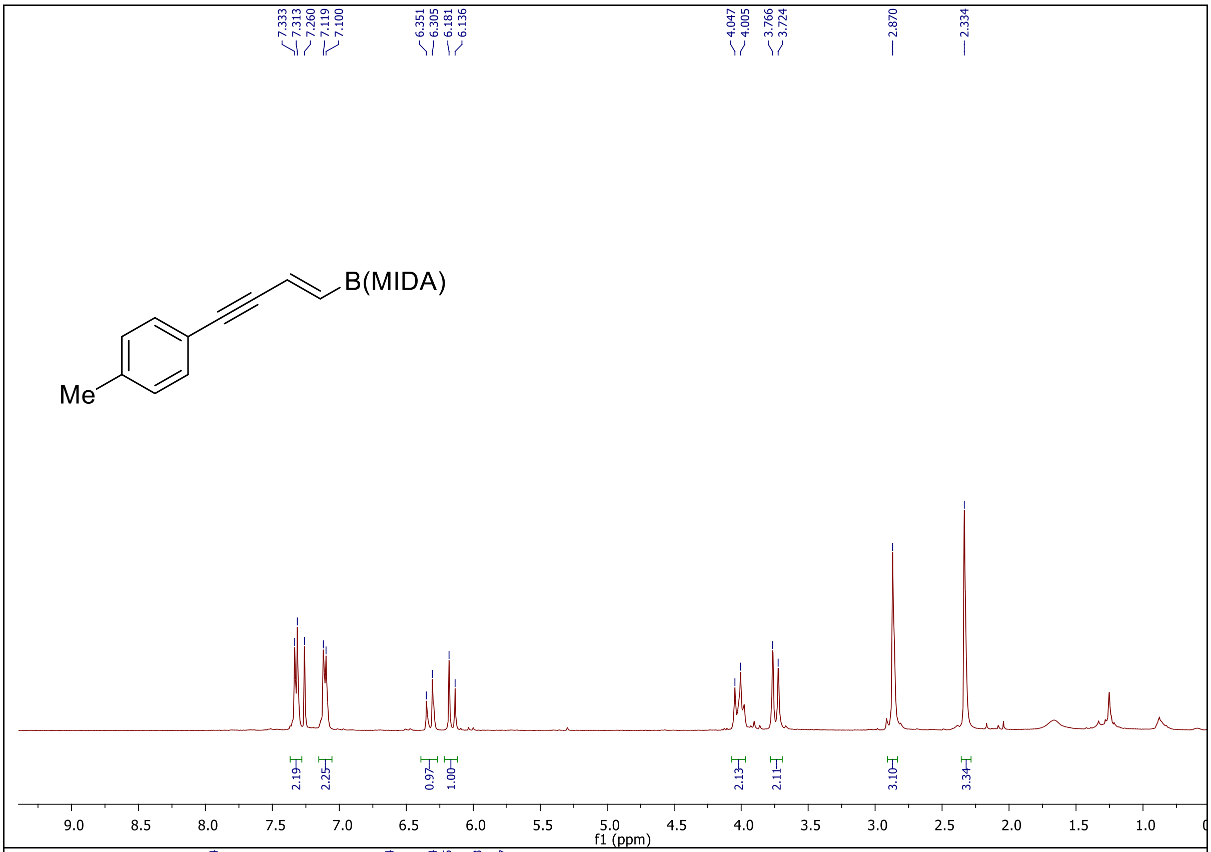

7.333
7.313
7.260
7.119
7.100
6.351
6.305
6.181
6.136
4.047
4.005
3.766
3.724
2.870
2.334
B(MIDA)
Me
2.19
2.25
0.97
1.00
2.13
2.11
3.10
3.34
9.0
8.5
8.0
7.5
7.0
6.5
6.0
5.5
5.0
4.5
4.0
3.5
3.0
2.5
2.0
1.5
1.0
f1 (ppm)

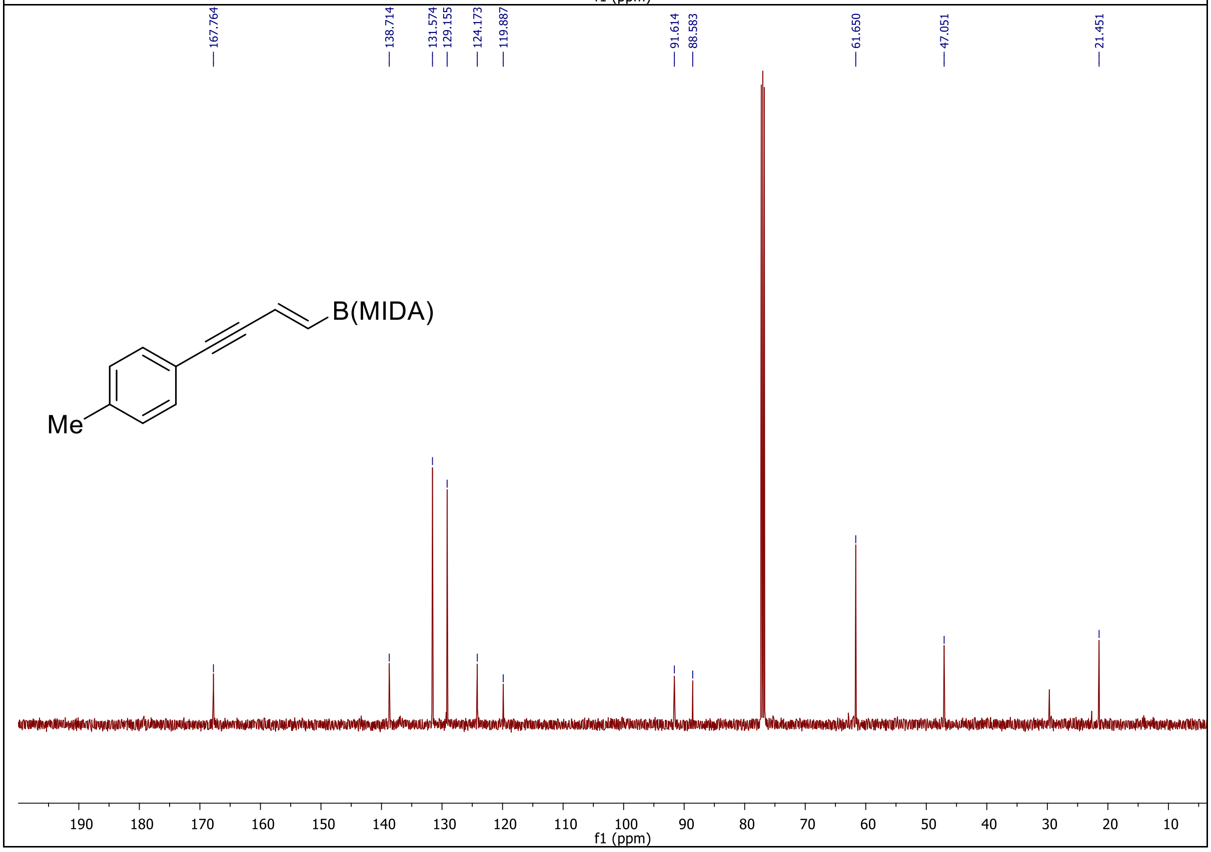

167.764
138.714
131.574
129.155
124.173
119.887
91.614
88.583
61.650
47.051
21.451
B(MIDA)
Me
190
180
170
160
150
140
130
120
110
100
90
80
70
60
50
40
30
20
10
f1 (ppm)

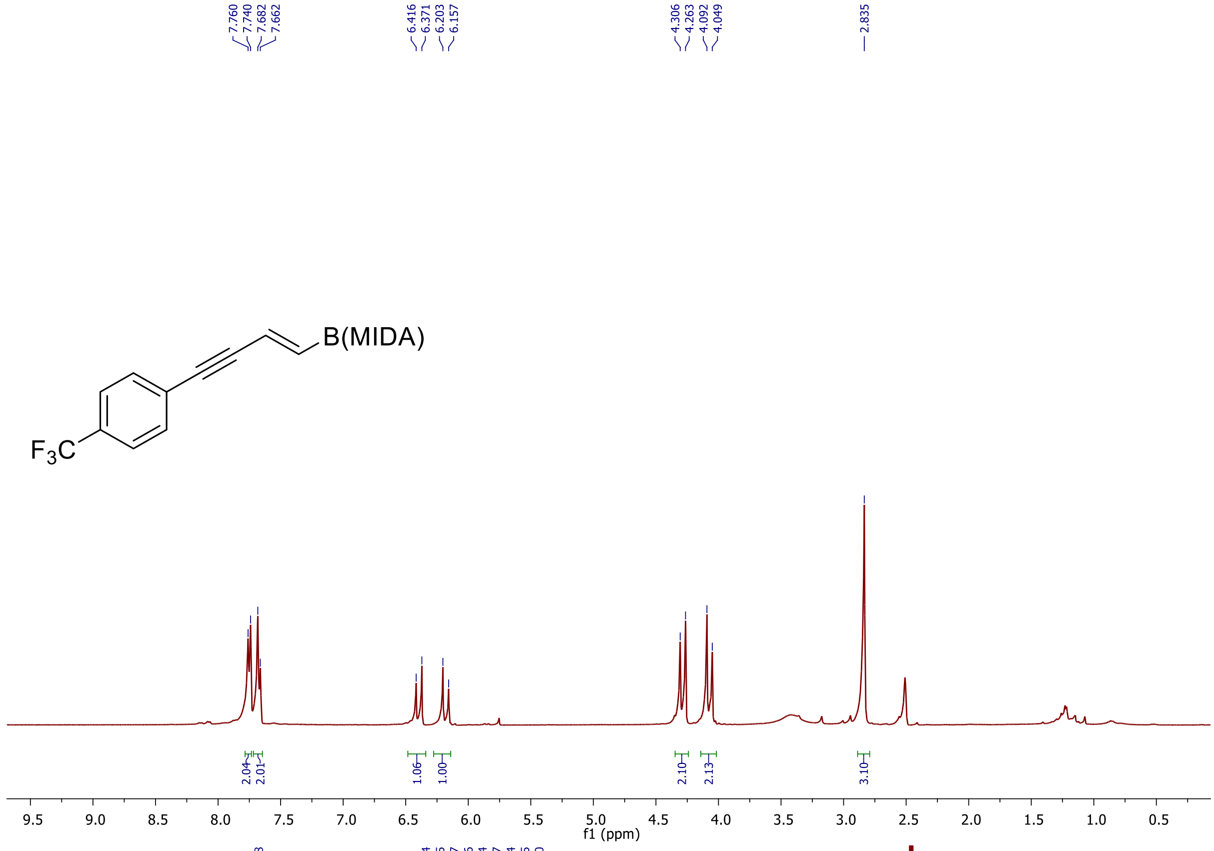

7.760
7.740
7.682
7.662
6.416
6.371
6.203
6.157
4.306
4.263
4.092
4.049
2.835
B(MIDA)
F3C
2.04
2.01
1.06
1.00
2.10
2.13
3.10
9.5 9.0 8.5 8.0 7.5 7.0 6.5 6.0 5.5 5.0 4.5 4.0 3.5 3.0 2.5 2.0 1.5 1.0 0.5
f1 (ppm)

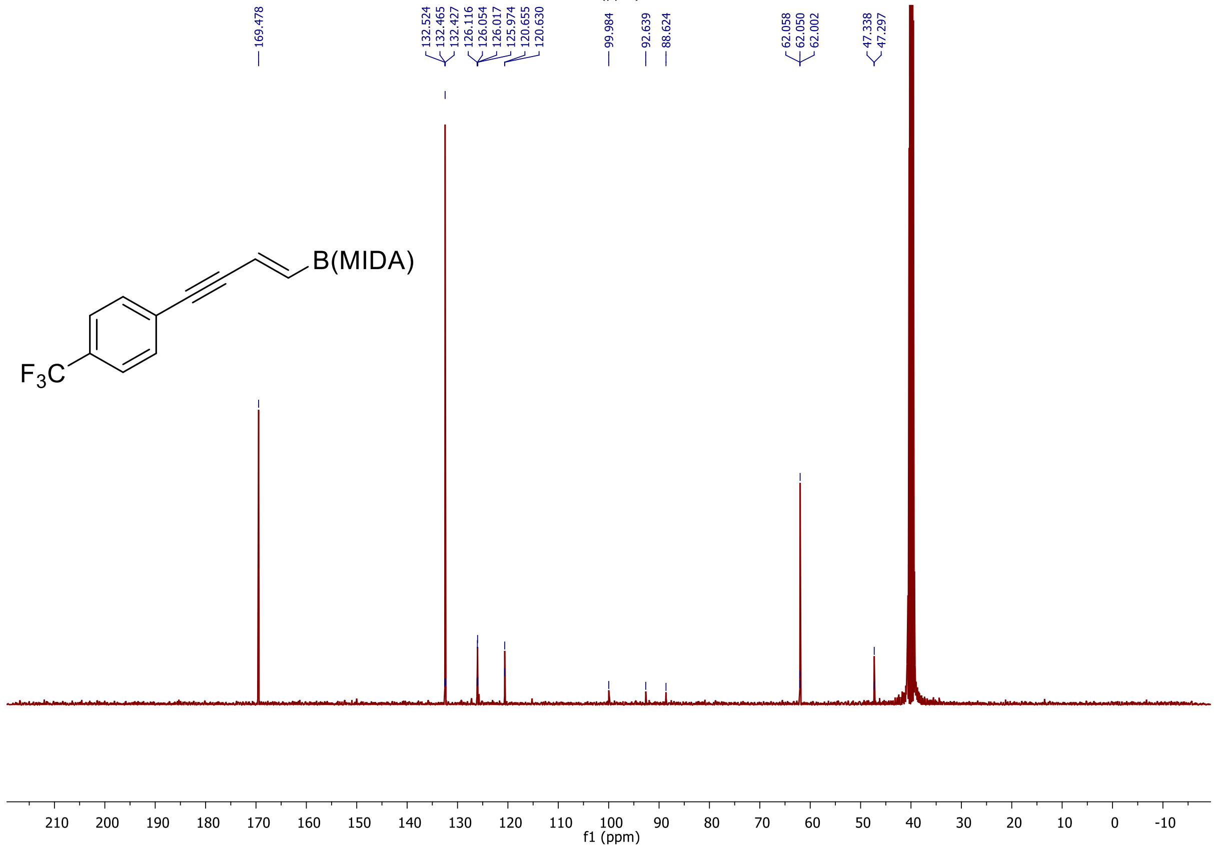

169.478
132.524
132.465
132.427
126.116
126.054
126.017
125.974
120.655
120.630
99.984
92.639
88.624
62.058
62.050
62.002
47.338
47.297
B(MIDA)
F3C
210 200 190 180 170 160 150 140 130 120 110 100 90 80 70 60 50 40 30 20 10 0 -10
f1 (ppm)

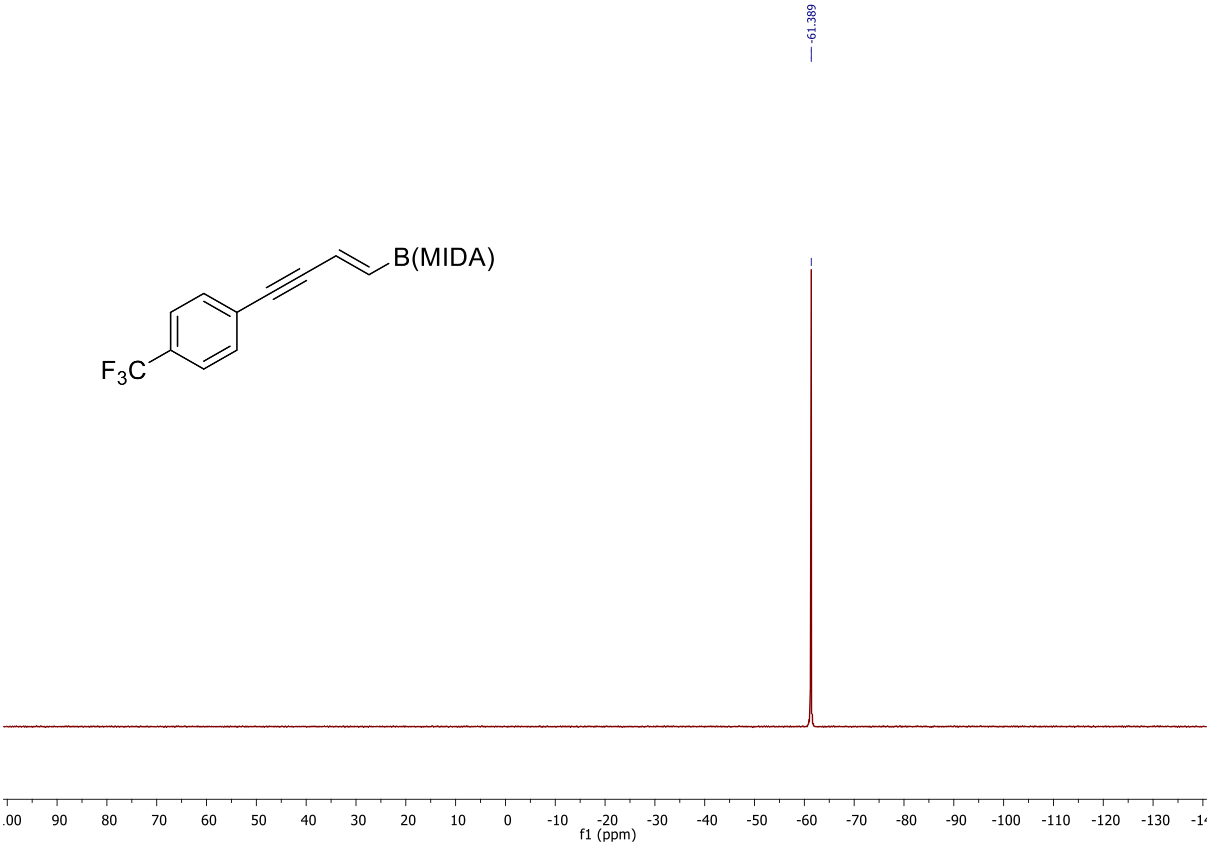
-61.389
B(MIDA)
F3C
f1 (ppm)

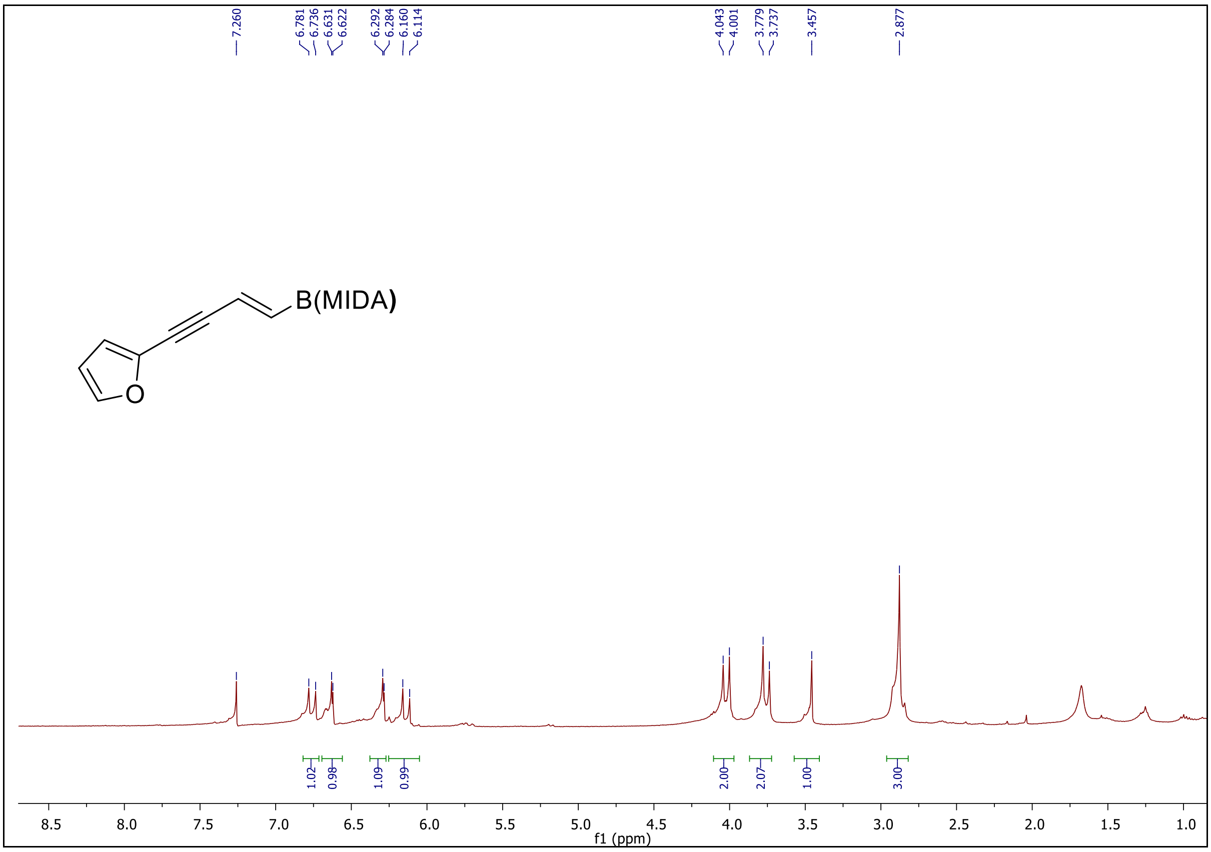
7.260
6.781
6.736
6.631
6.622
6.292
6.284
6.160
6.114
4.043
4.001
3.779
3.737
3.457
2.877
B(MIDA)
O
1.02
0.98
1.09
0.99
2.00
2.07
1.00
3.00
f1 (ppm)

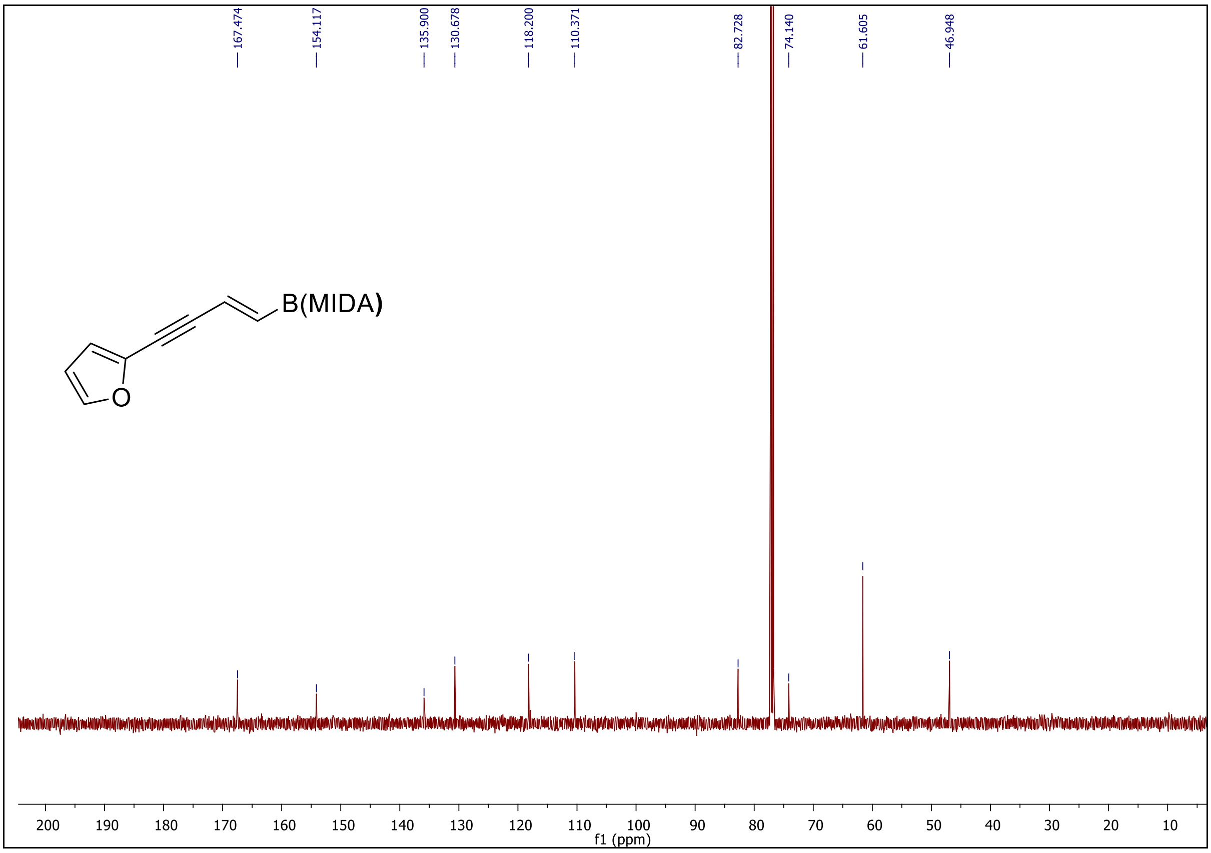
167.474
154.117
135.900
130.678
118.200
110.371
82.728
74.140
61.605
46.948
B(MIDA)
O
200
190
180
170
160
150
140
130
120
110
100
90
80
70
60
50
40
30
20
10
f1 (ppm)

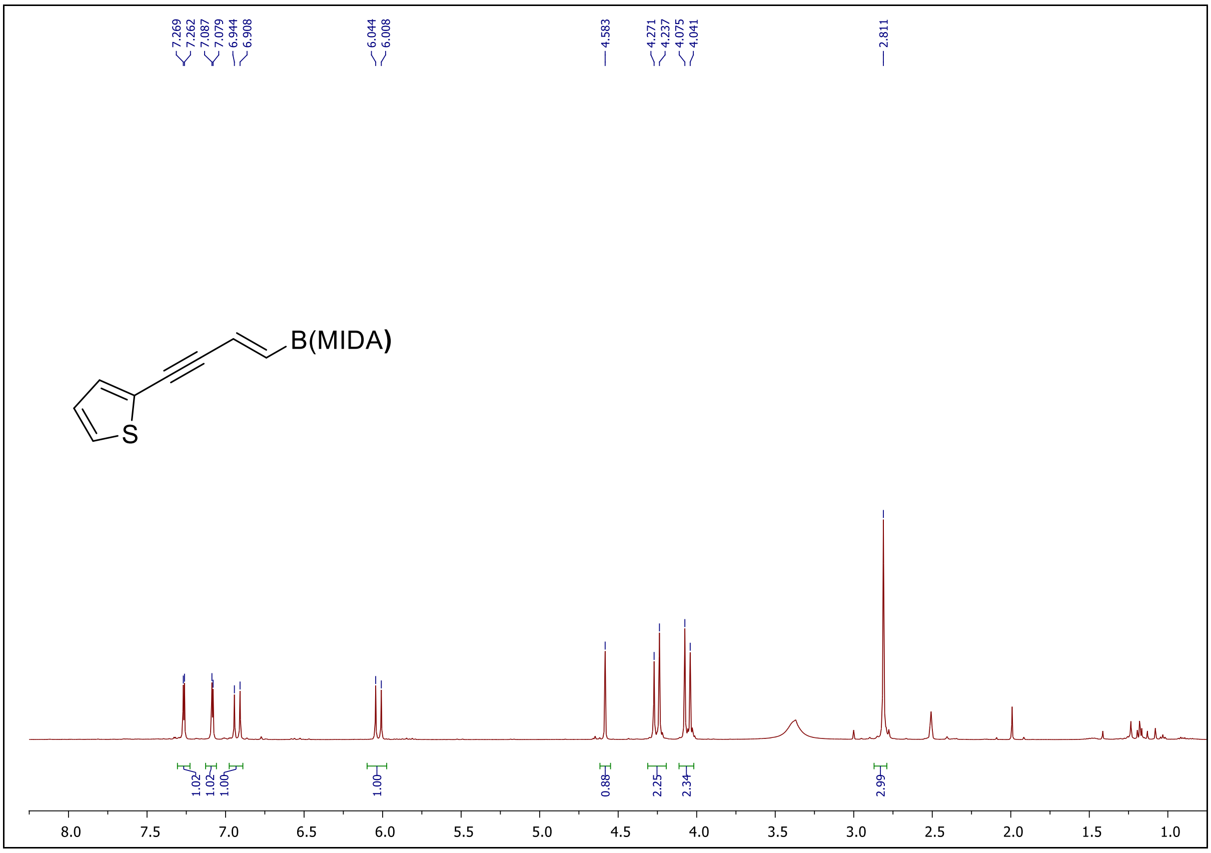
7.269
7.262
7.087
7.079
6.944
6.908
6.044
6.008
4.583
4.271
4.237
4.075
4.041
2.811
B(MIDA)
S
1.02
1.02
1.00
1.00
0.88
2.25
2.34
2.99
8.0
7.5
7.0
6.5
6.0
5.5
5.0
4.5
4.0
3.5
3.0
2.5
2.0
1.5
1.0

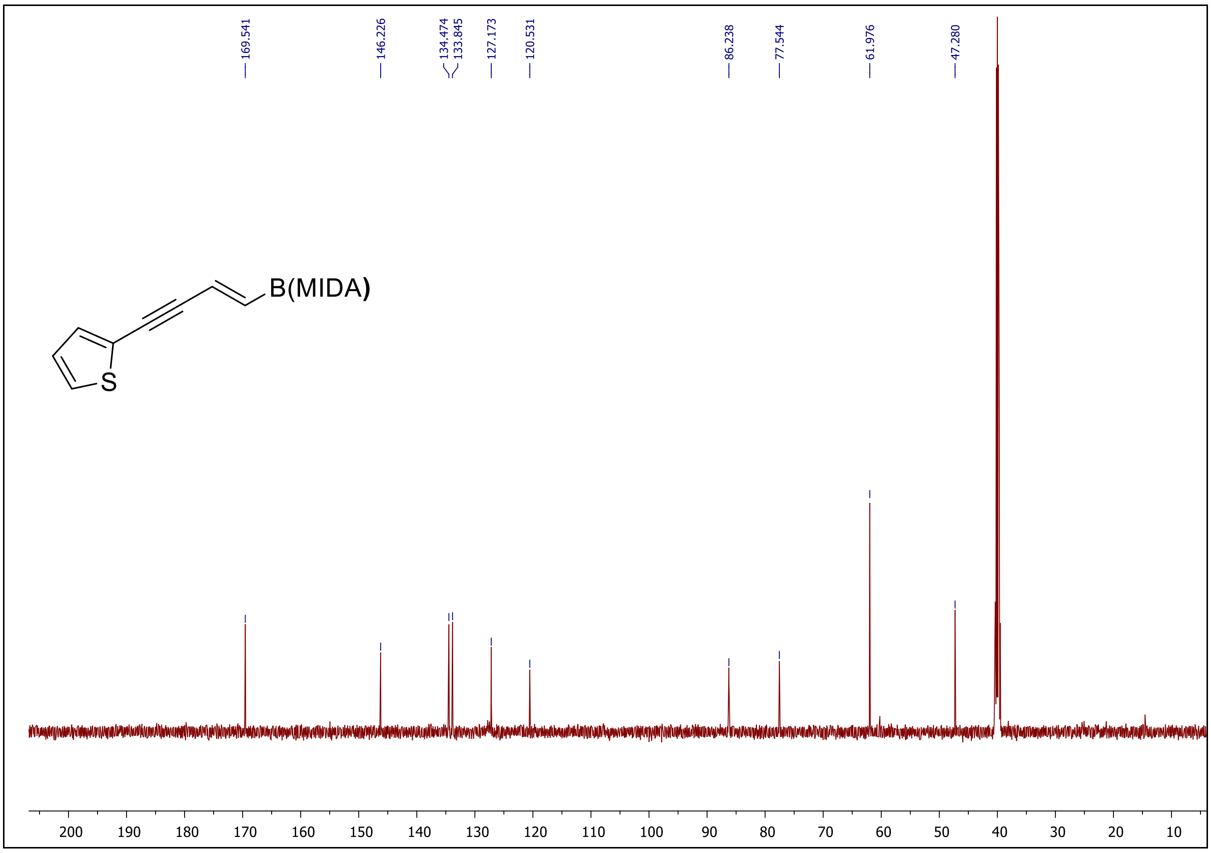
169.541
146.226
134.474
133.845
127.173
120.531
86.238
77.544
61.976
47.280
B(MIDA)
S
200 190 180 170 160 150 140 130 120 110 100 90 80 70 60 50 40 30 20 10

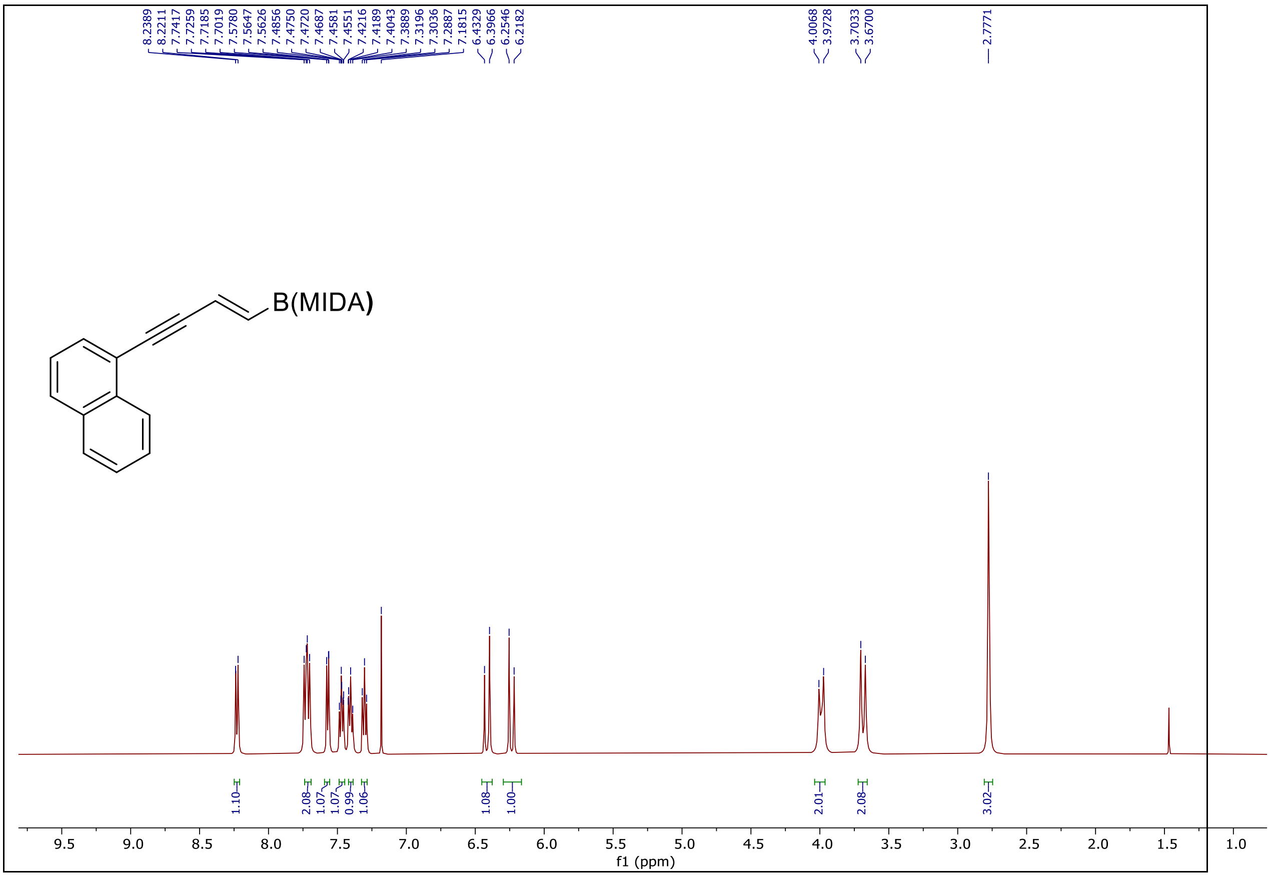
8.2389
8.2211
7.7417
7.7259
7.7185
7.7019
7.5780
7.5647
7.5626
7.4856
7.4750
7.4720
7.4687
7.4581
7.4551
7.4216
7.4189
7.4043
7.3889
7.3196
7.3036
7.2887
7.1815
6.4329
6.3966
6.2546
6.2182
4.0068
3.9728
3.7033
3.6700
2.7771
B(MIDA)
1.10
2.08
1.07
1.07
0.99
1.06
1.08
1.00
2.01
2.08
3.02
9.5 9.0 8.5 8.0 7.5 7.0 6.5 6.0 5.5 5.0 4.5 4.0 3.5 3.0 2.5 2.0 1.5 1.0
f1 (ppm)

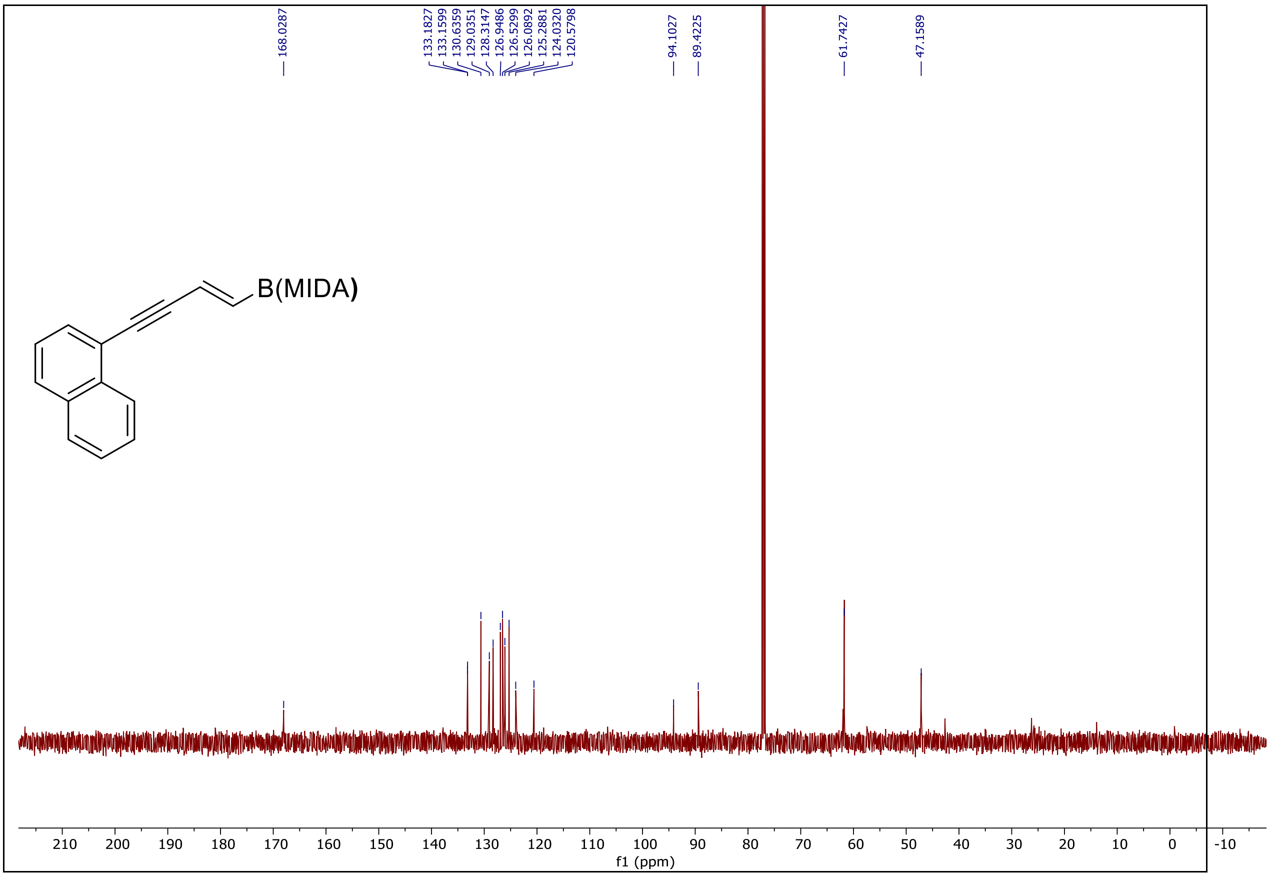
168.0287
133.1827
133.1599
130.6359
129.0351
128.3147
126.9486
126.5299
126.0892
125.2881
124.0320
120.5798
94.1027
89.4225
61.7427
47.1589
B(MIDA)
210 200 190 180 170 160 150 140 130 120 110 100 90 80 70 60 50 40 30 20 10 0 -10
f1 (ppm)

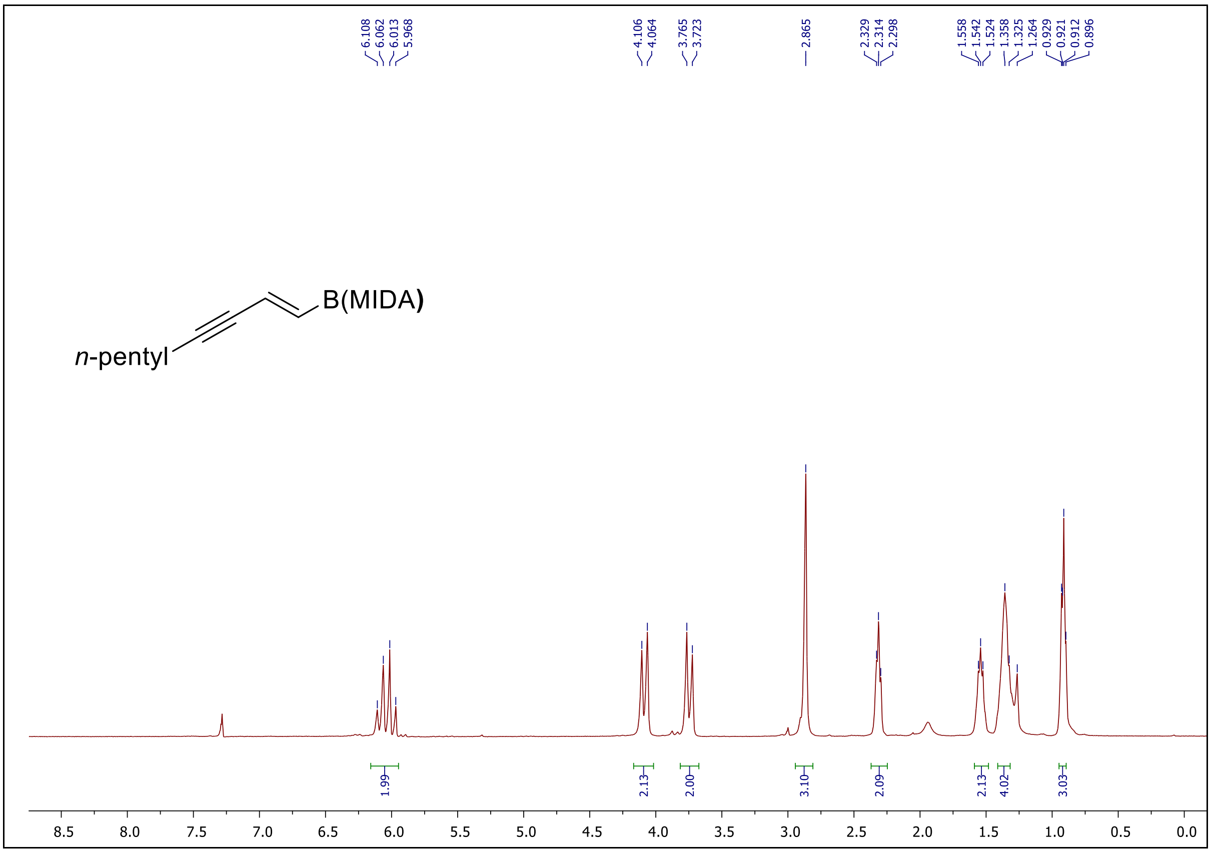
6.108
6.062
6.013
5.968
4.106
4.064
3.765
3.723
2.865
2.329
2.314
2.298
1.558
1.542
1.524
1.358
1.325
1.264
0.929
0.921
0.912
0.896
B(MIDA)
n-pentyl
1.99
2.13
2.00
3.10
2.09
2.13
4.02
3.03
8.5 8.0 7.5 7.0 6.5 6.0 5.5 5.0 4.5 4.0 3.5 3.0 2.5 2.0 1.5 1.0 0.5 0.0

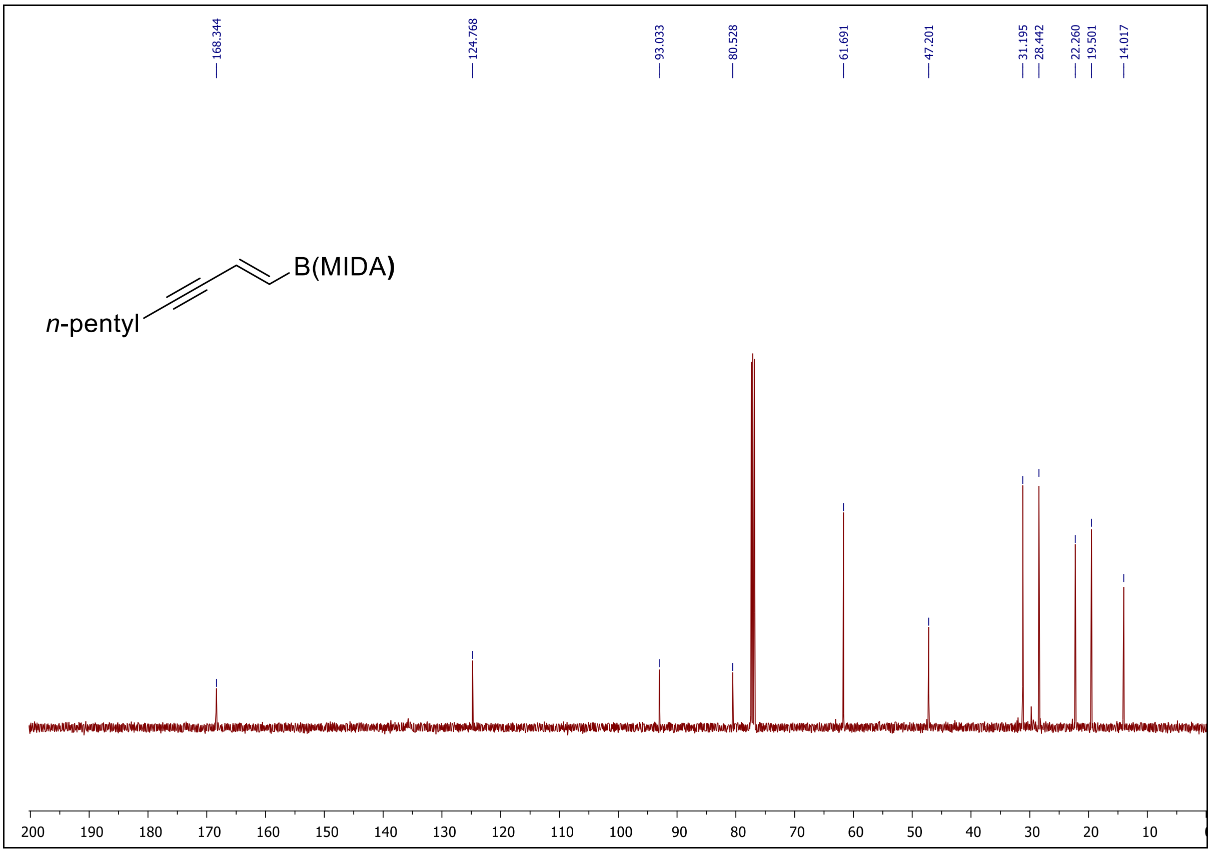
168.344
124.768
93.033
80.528
61.691
47.201
31.195
28.442
22.260
19.501
14.017
B(MIDA)
n-pentyl
200
190
180
170
160
150
140
130
120
110
100
90
80
70
60
50
40
30
20
10

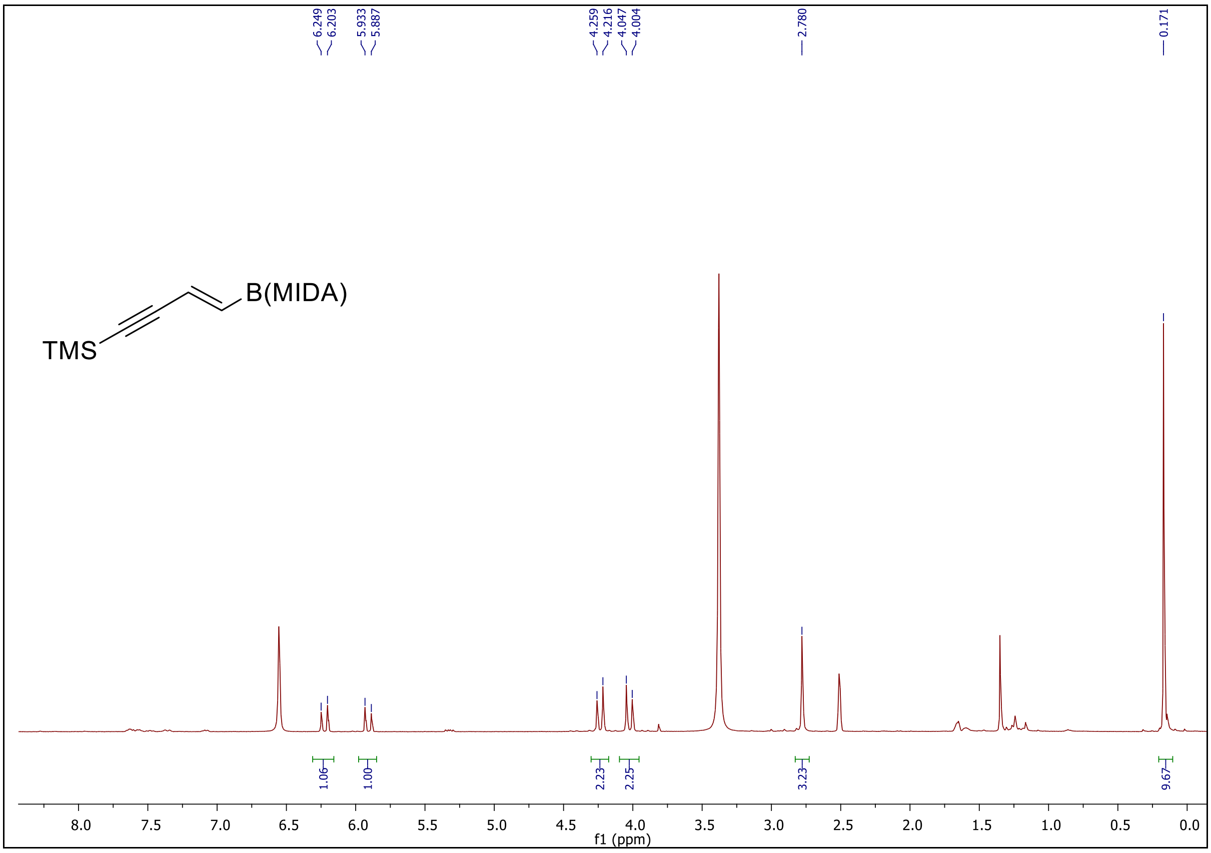
6.249
6.203
5.933
5.887
4.259
4.216
4.047
4.004
2.780
0.171
B(MIDA)
TMS
1.06
1.00
2.23
2.25
3.23
9.67
8.0
7.5
7.0
6.5
6.0
5.5
5.0
4.5
4.0
3.5
3.0
2.5
2.0
1.5
1.0
0.5
0.0
f1 (ppm)

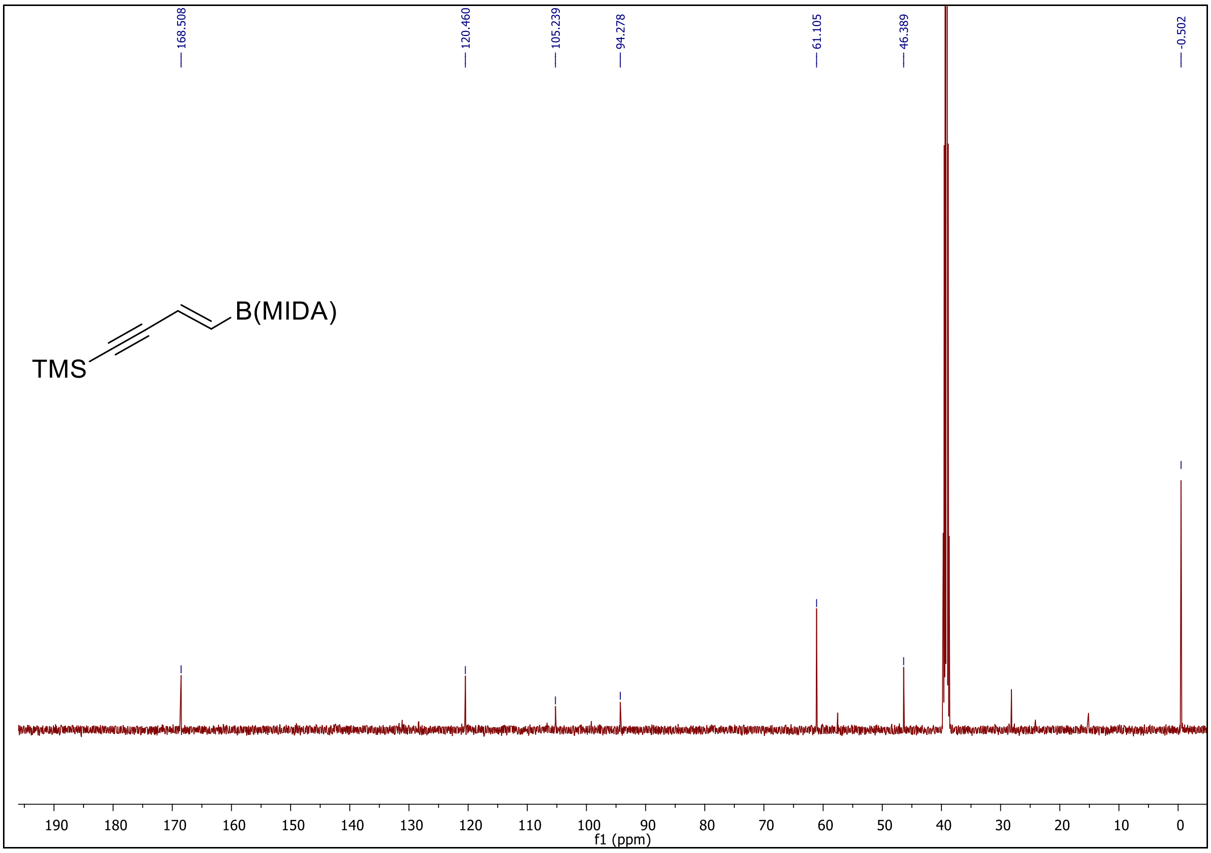
168.508
120.460
105.239
94.278
61.105
46.389
-0.502
B(MIDA)
TMS
190
180
170
160
150
140
130
120
110
100
90
80
70
60
50
40
30
20
10
0
f1 (ppm)

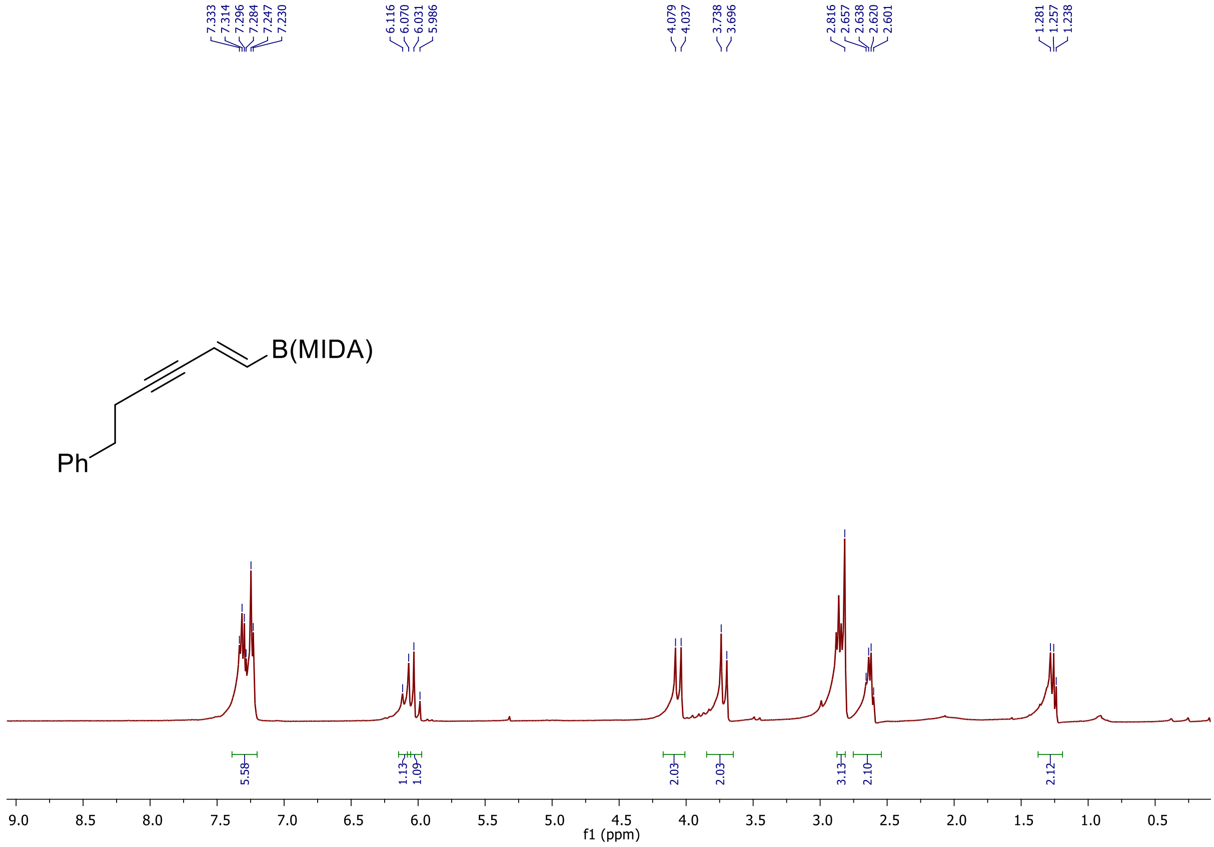
7.333
7.314
7.296
7.284
7.247
7.230
6.116
6.070
6.031
5.986
4.079
4.037
3.738
3.696
2.816
2.657
2.638
2.620
2.601
1.281
1.257
1.238
B(MIDA)
Ph
5.58
1.13
1.09
2.03
2.03
3.13
2.10
2.12
f1 (ppm)

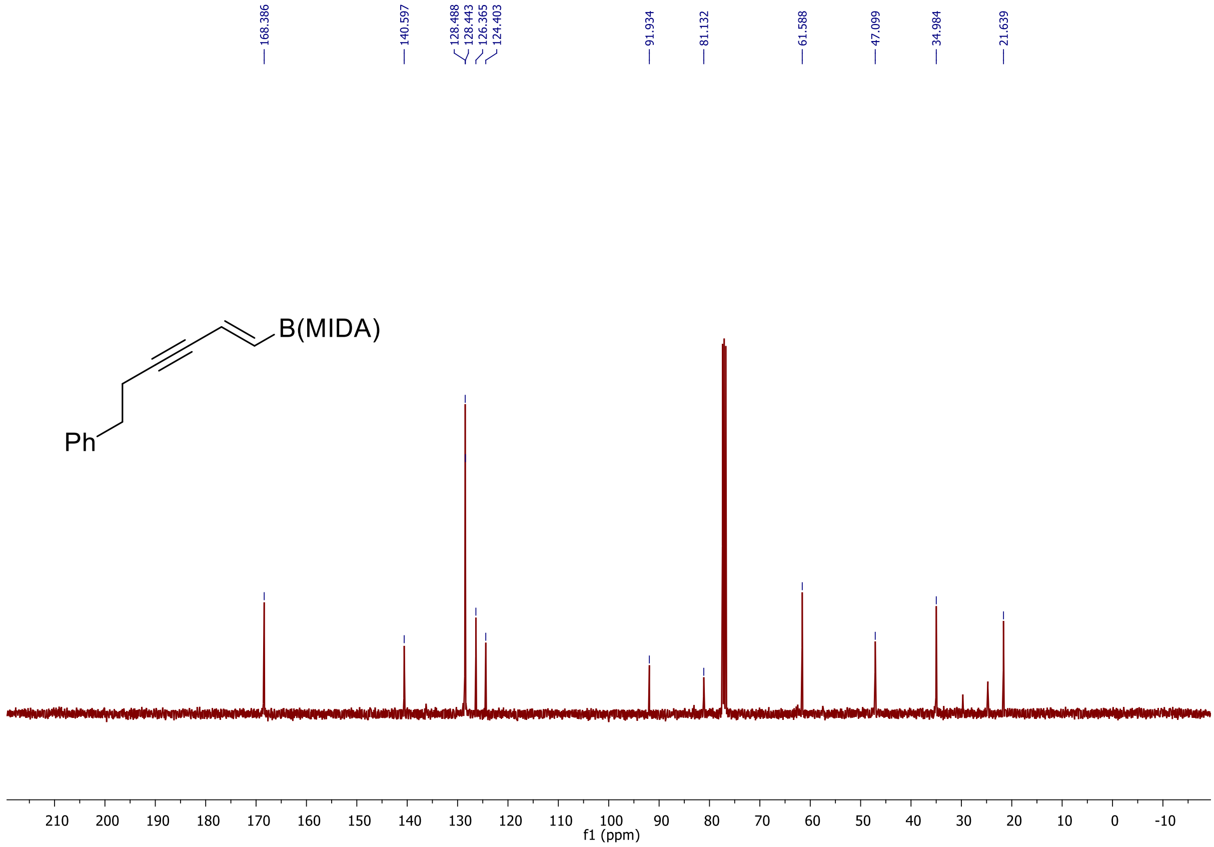
168.386
140.597
128.488
128.443
126.365
124.403
91.934
81.132
61.588
47.099
34.984
21.639
B(MIDA)
Ph
f1 (ppm)

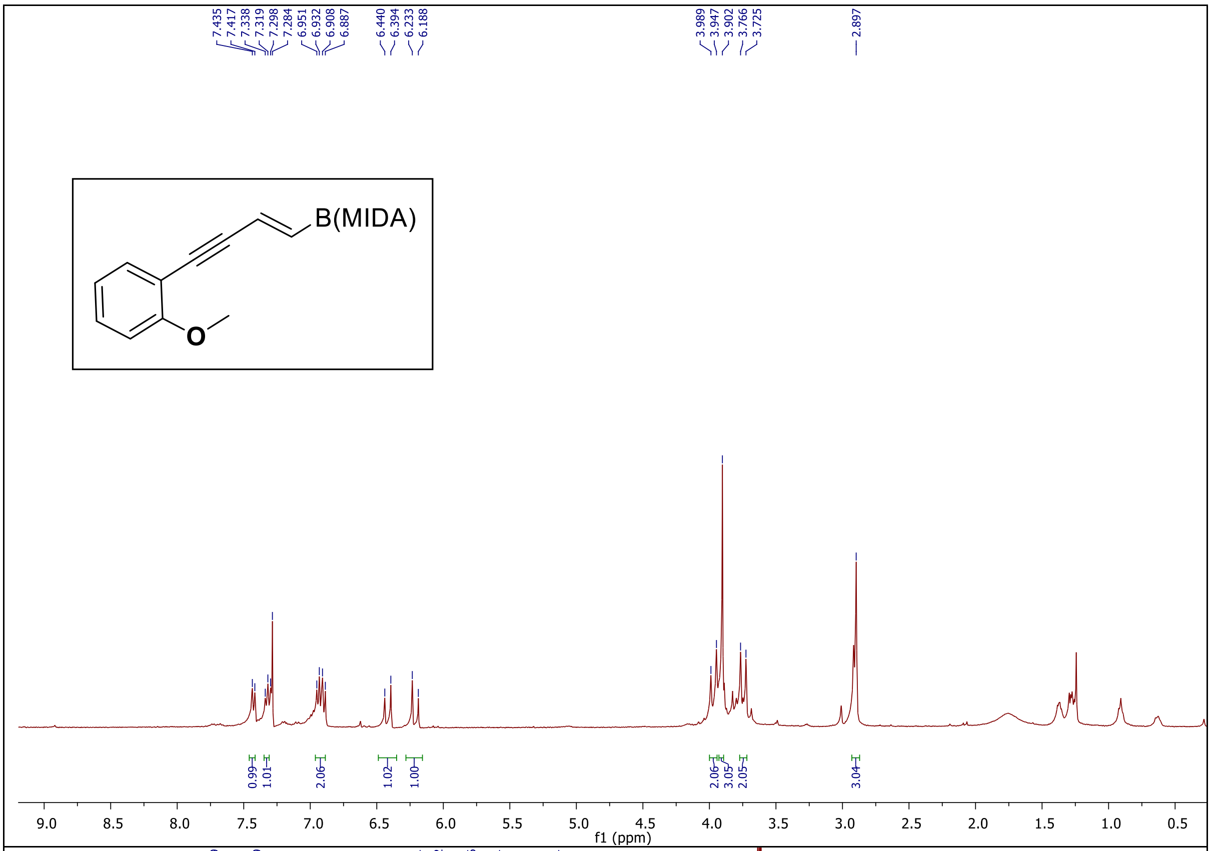

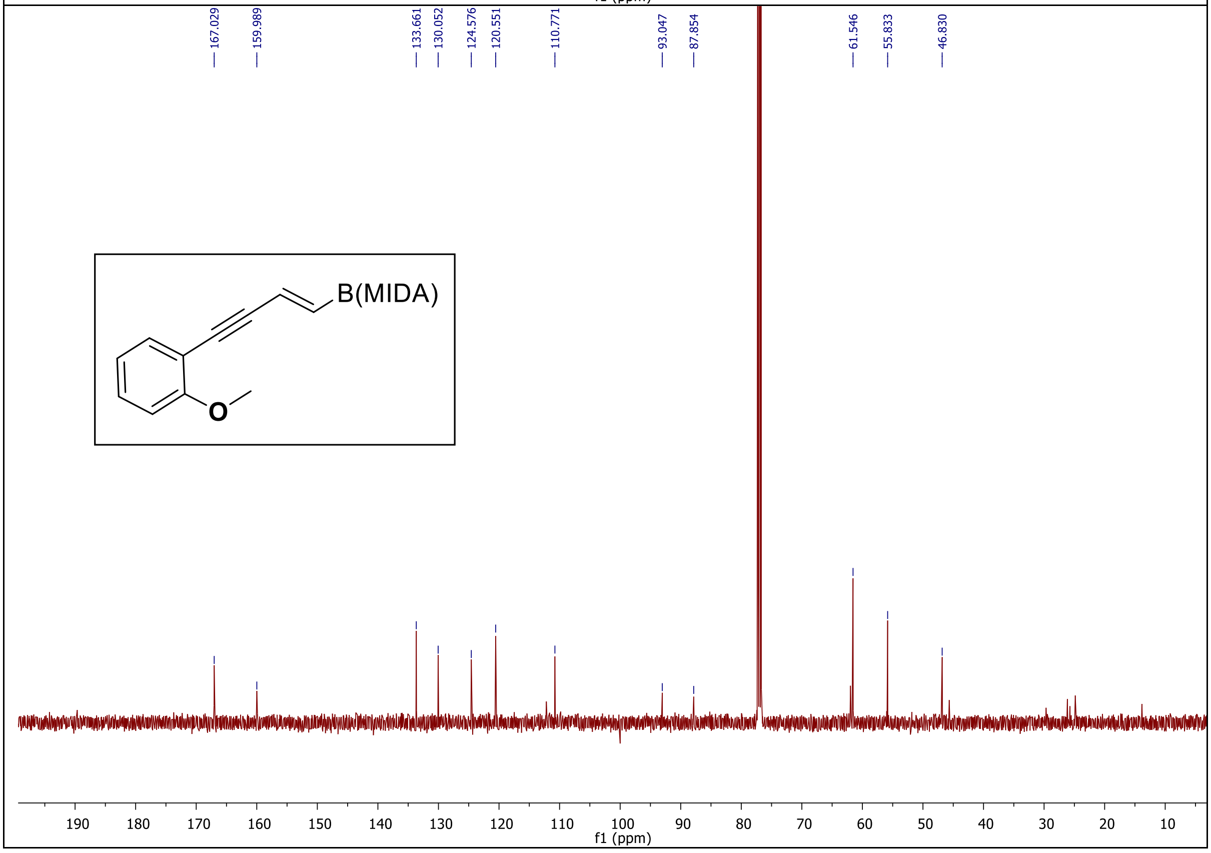

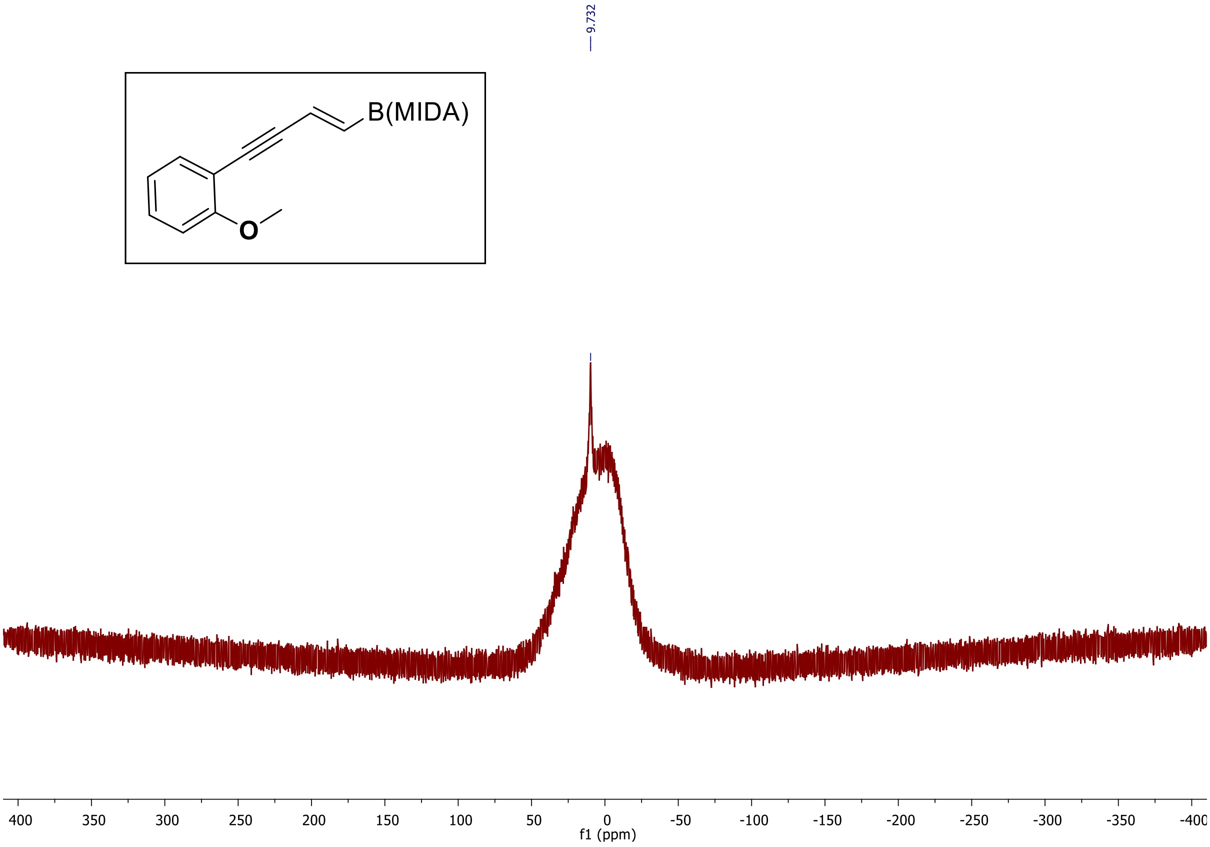

B(MIDA)
O
— 9.732
f1 (ppm)

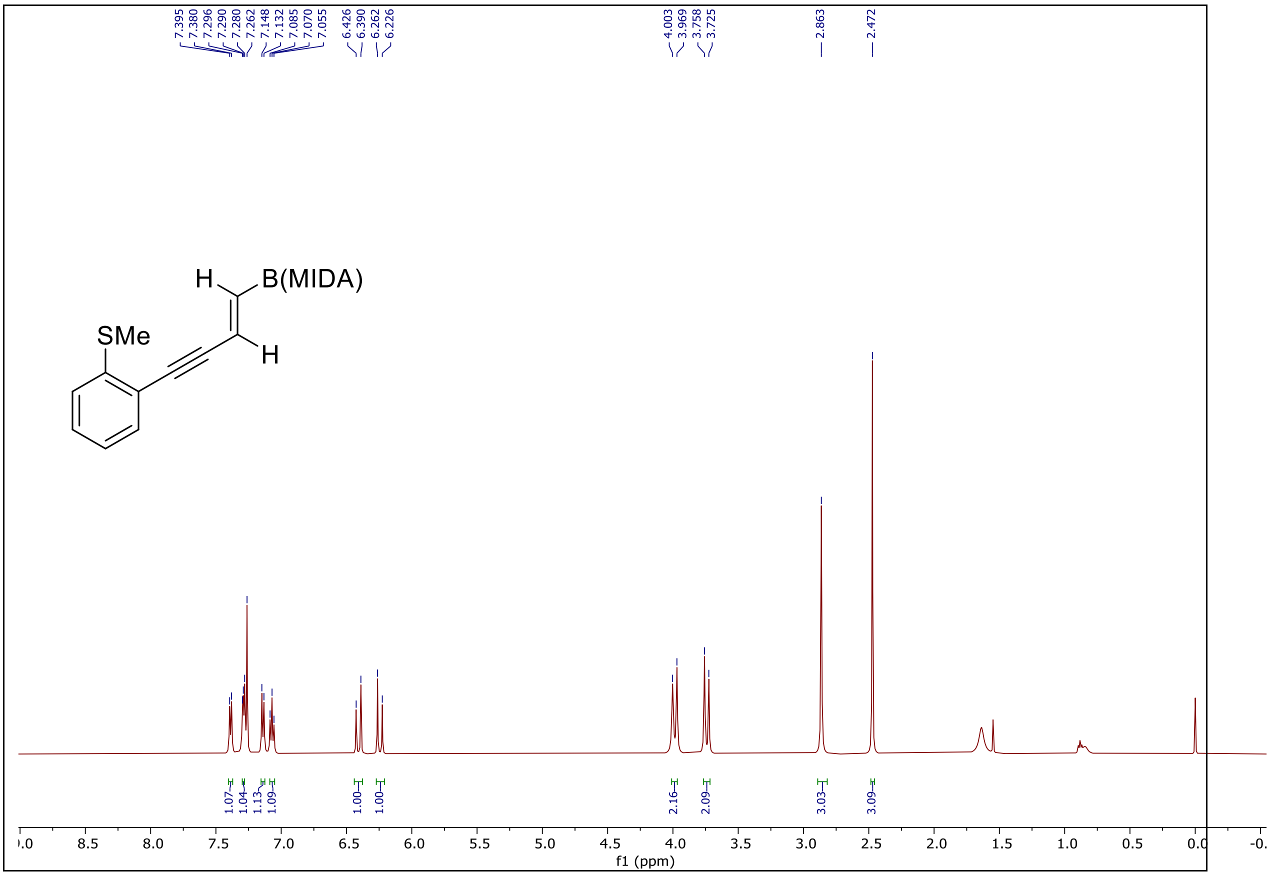

H
B(MIDA)
SMe
H
7.395
7.380
7.296
7.290
7.280
7.262
7.148
7.132
7.085
7.070
7.055
6.426
6.390
6.262
6.226
4.003
3.969
3.758
3.725
2.863
2.472
1.07
1.04
1.13
1.09
1.00
1.00
2.16
2.09
3.03
3.09
f1 (ppm)

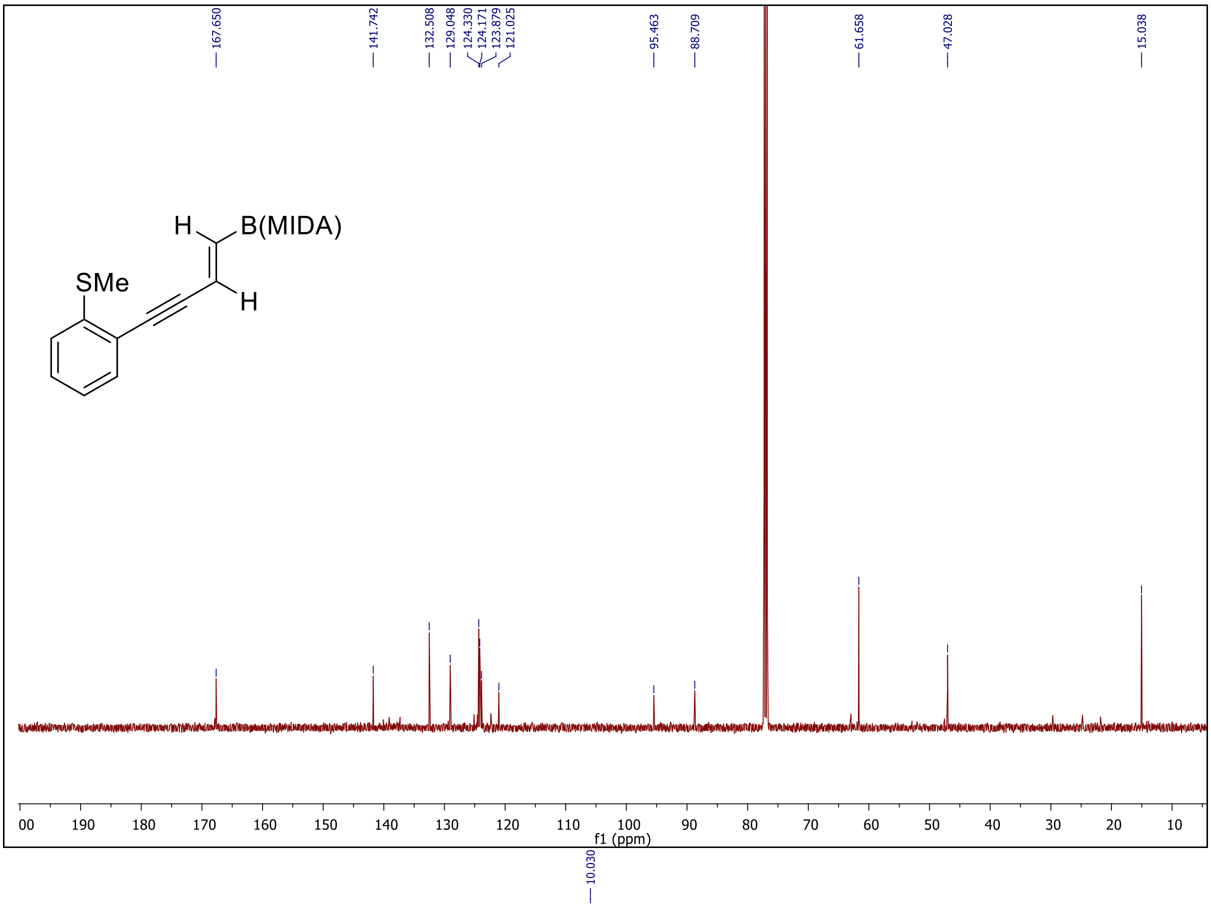
167.650
141.742
132.508
129.048
124.330
124.171
123.879
121.025
95.463
88.709
61.658
47.028
15.038
H
B(MIDA)
SMe
H
f1 (ppm)

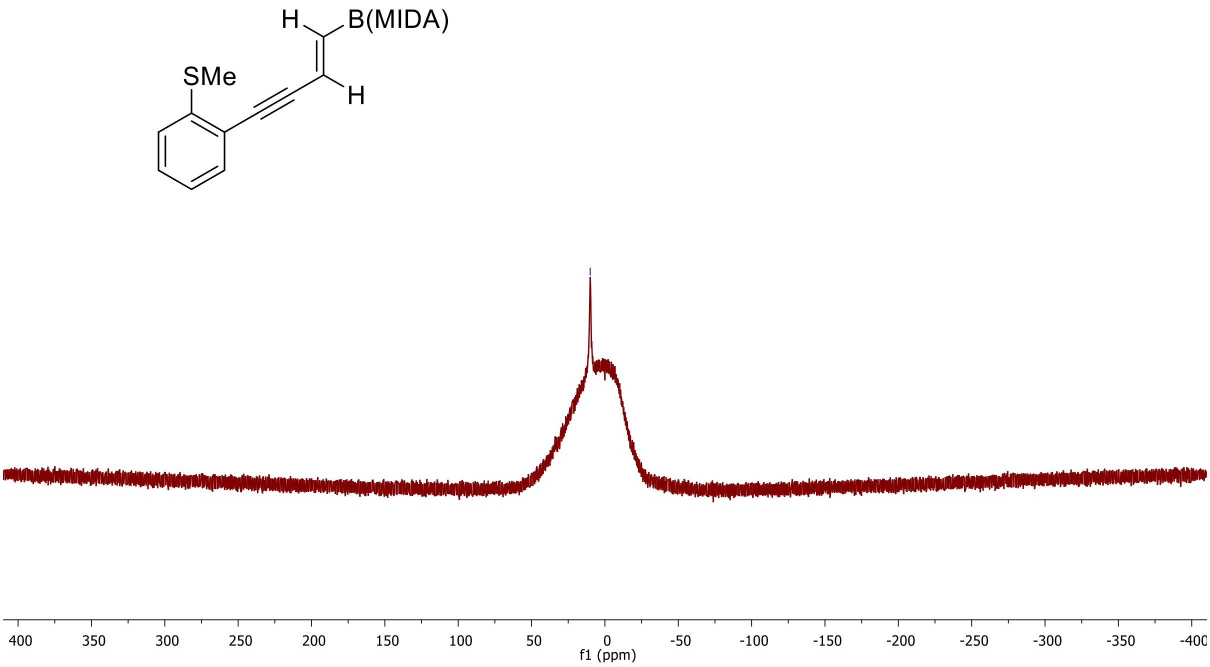
10.030
H
B(MIDA)
SMe
H
f1 (ppm)

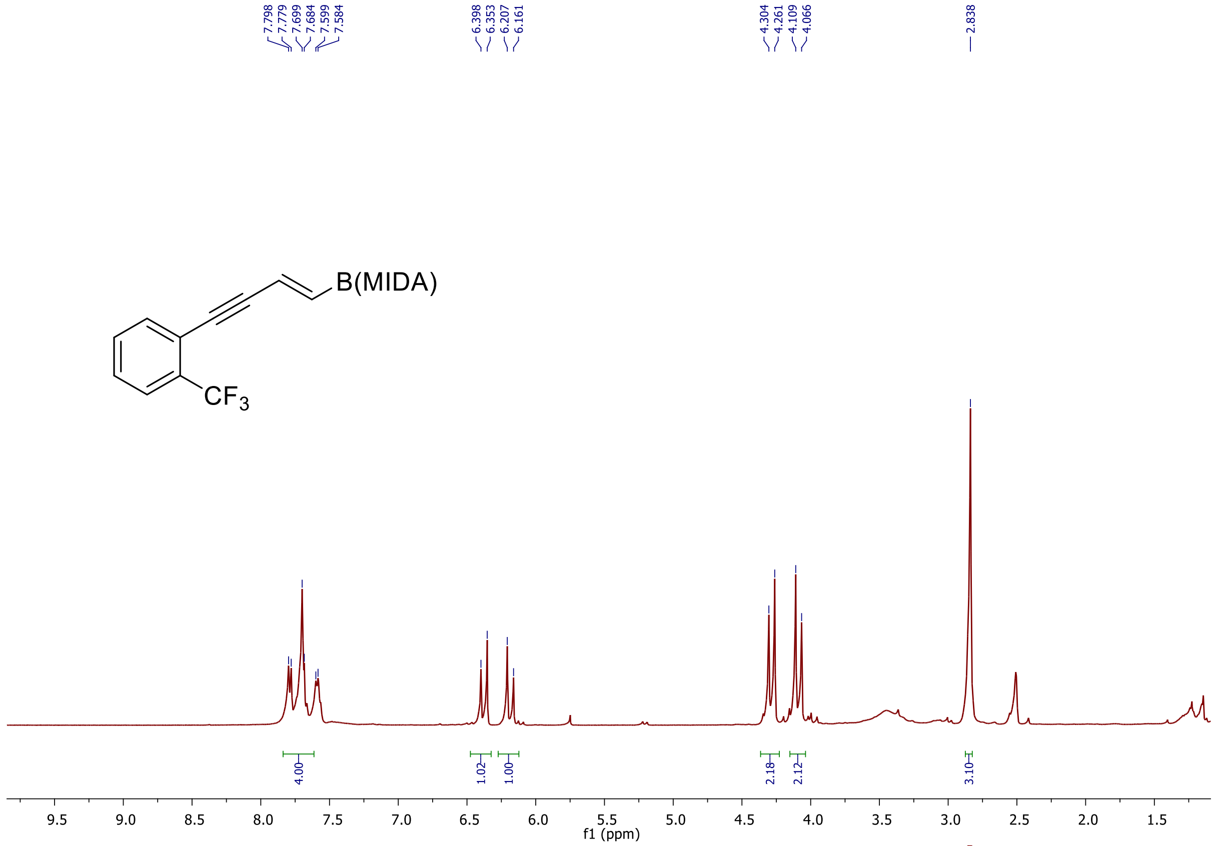
7.798
7.779
7.699
7.684
7.599
7.584
6.398
6.353
6.207
6.161
4.304
4.261
4.109
4.066
2.838
B(MIDA)
CF3
4.00
1.02
1.00
2.18
2.12
3.10
9.5 9.0 8.5 8.0 7.5 7.0 6.5 6.0 5.5 5.0 4.5 4.0 3.5 3.0 2.5 2.0 1.5
f1 (ppm)

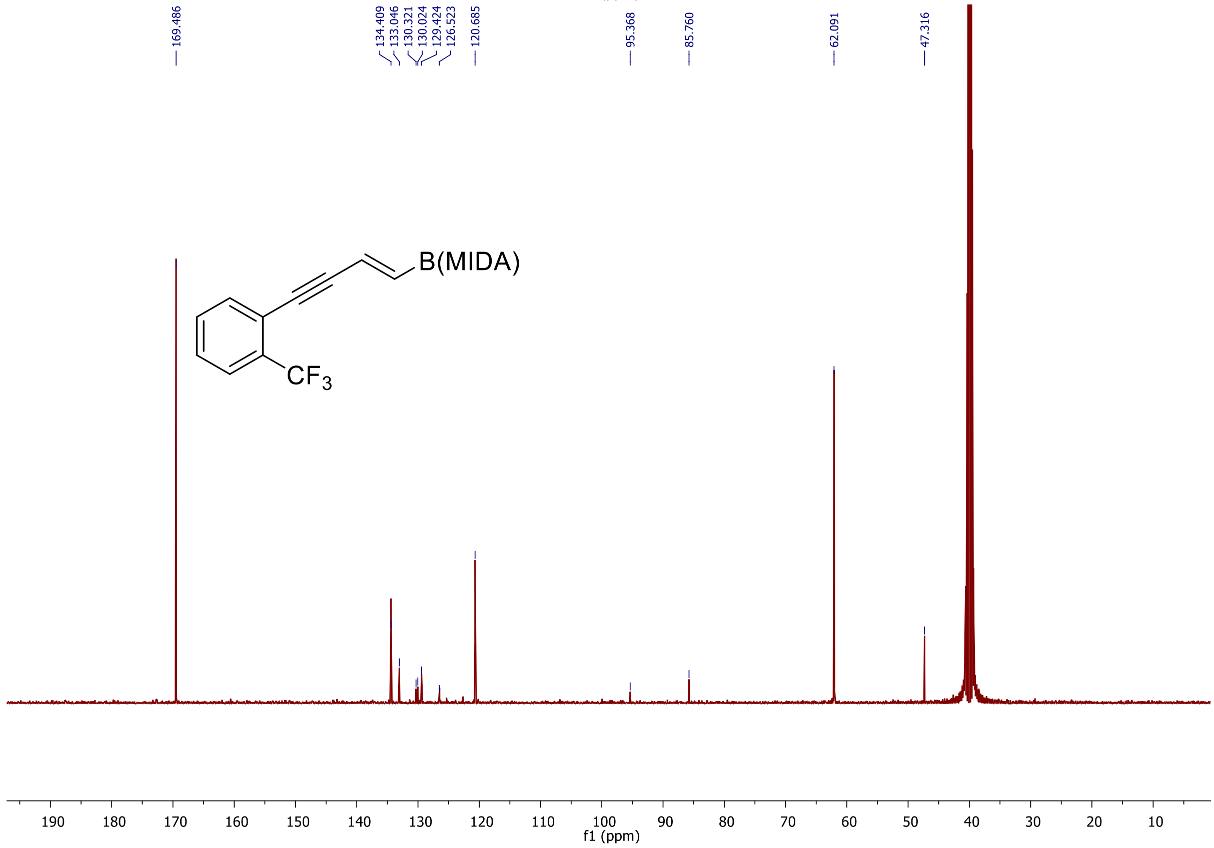
169.486
134.409
133.046
130.321
130.024
129.424
126.523
120.685
95.368
85.760
62.091
47.316
B(MIDA)
CF3
190 180 170 160 150 140 130 120 110 100 90 80 70 60 50 40 30 20 10
f1 (ppm)

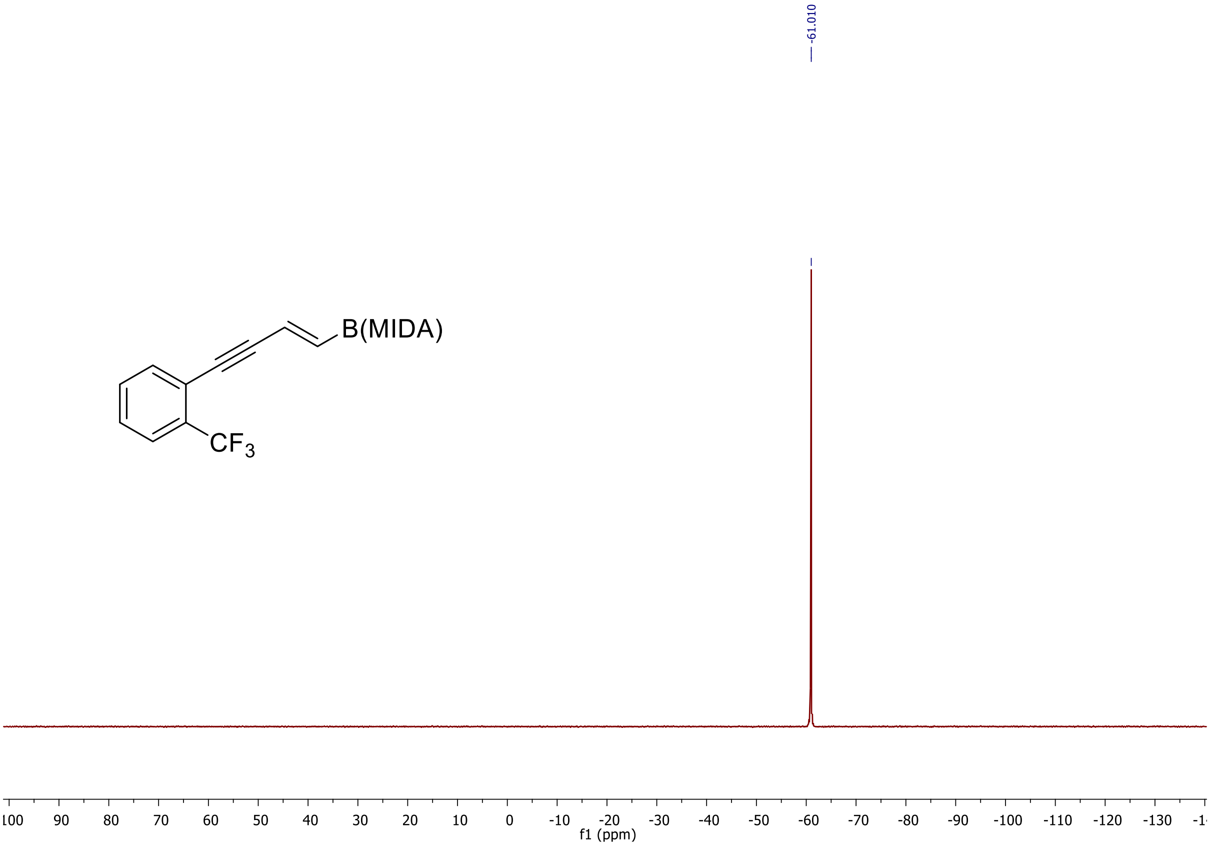
-61.010
B(MIDA)
CF3
100 90 80 70 60 50 40 30 20 10 0 -10 -20 -30 -40 -50 -60 -70 -80 -90 -100 -110 -120 -130
f1 (ppm)

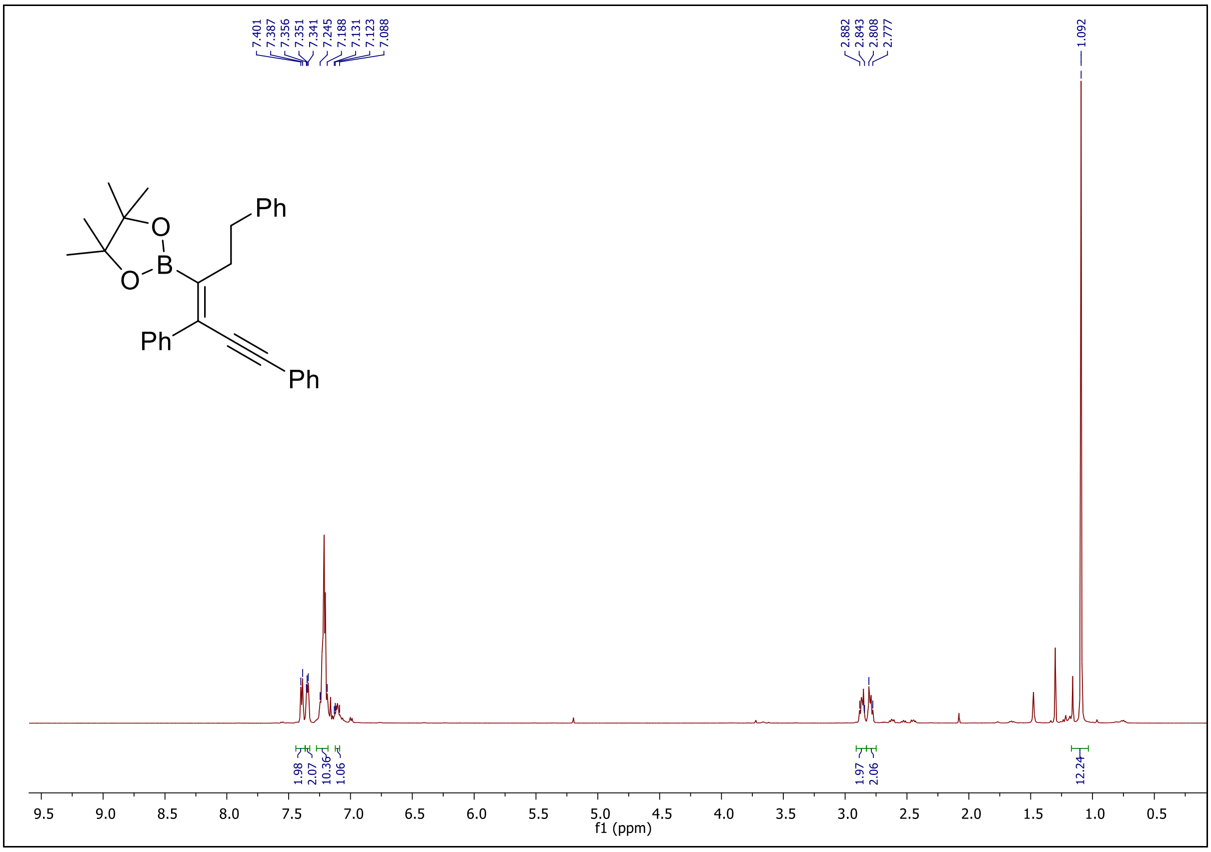
7.401 7.387 7.356 7.351 7.341 7.245 7.188 7.131 7.123 7.088
2.882 2.843 2.808 2.777
1.092
Ph
O
B
O
Ph
Ph
1.98 2.07 10.36 1.06
1.97 2.06
12.24
9.5 9.0 8.5 8.0 7.5 7.0 6.5 6.0 5.5 5.0 4.5 4.0 3.5 3.0 2.5 2.0 1.5 1.0 0.5
f1 (ppm)

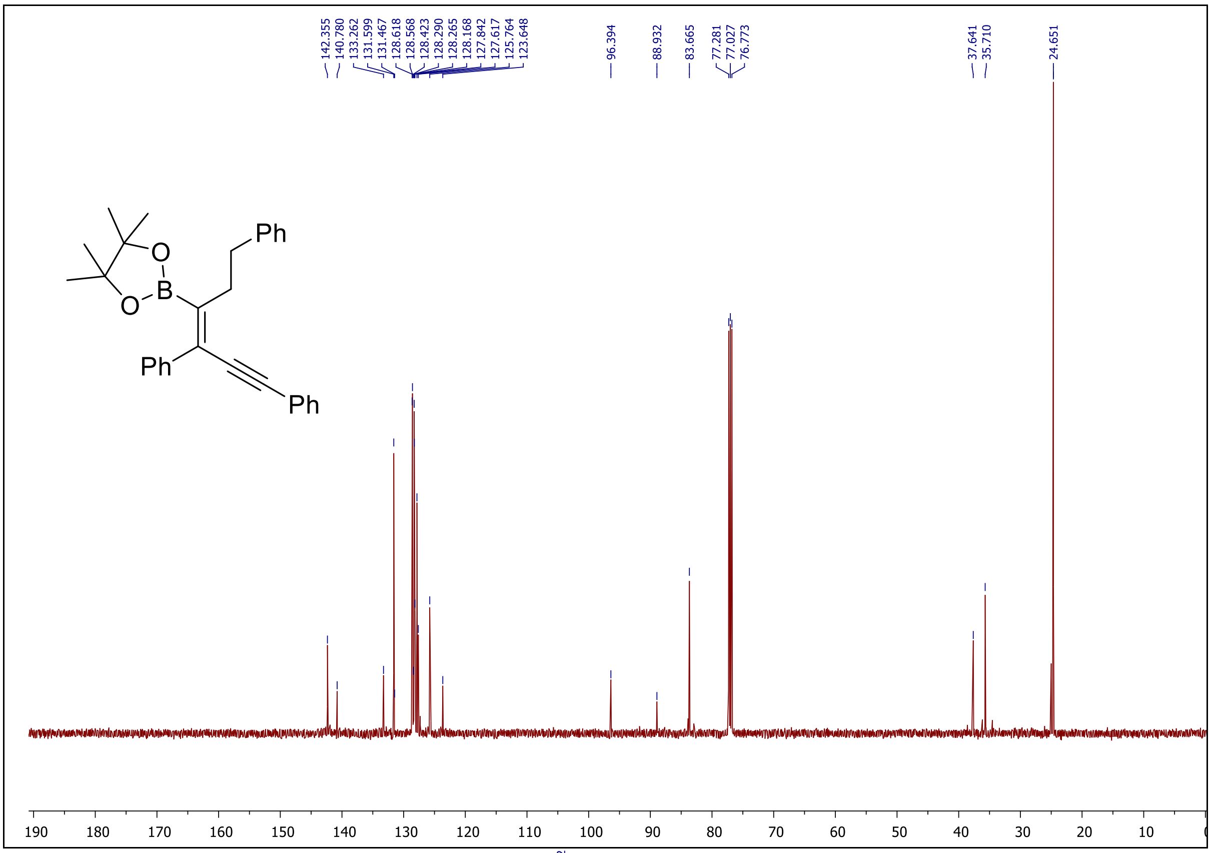
142.355
140.780
133.262
131.599
131.467
128.618
128.568
128.423
128.290
128.265
128.168
127.842
127.617
125.764
123.648
96.394
88.932
83.665
77.281
77.027
76.773
37.641
35.710
24.651
Ph
O
B
O
Ph
Ph
190
180
170
160
150
140
130
120
110
100
90
80
70
60
50
40
30
20
10

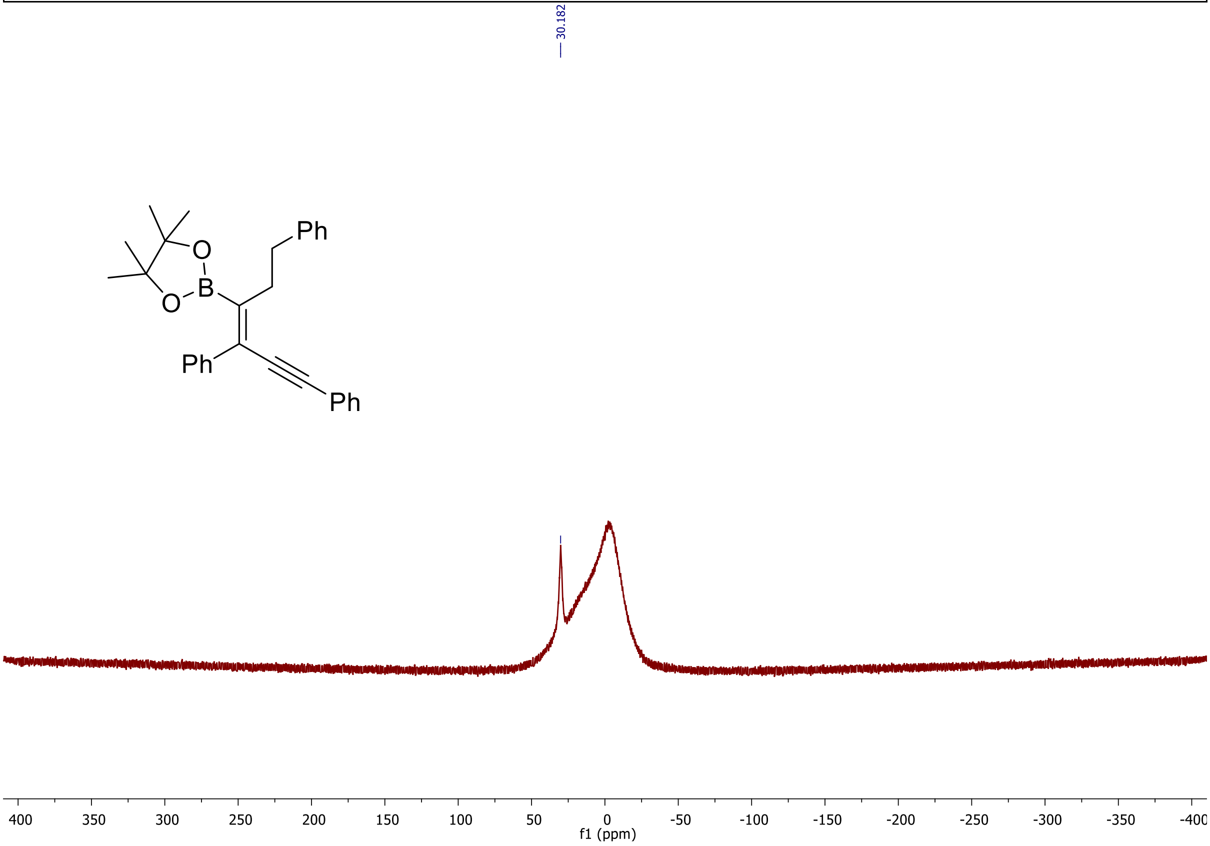
30.182
Ph
O
B
O
Ph
Ph
400
350
300
250
200
150
100
50
0
-50
-100
-150
-200
-250
-300
-350
-400
f1 (ppm)

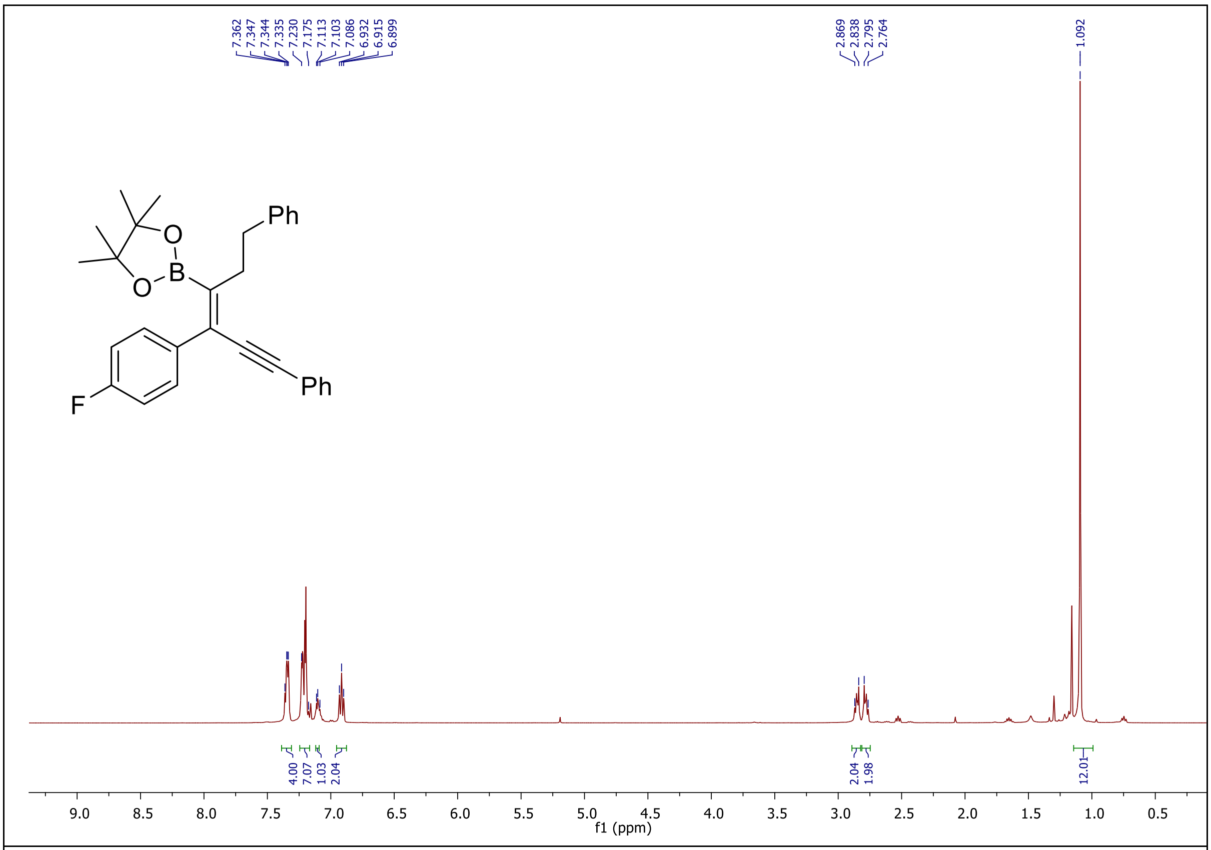

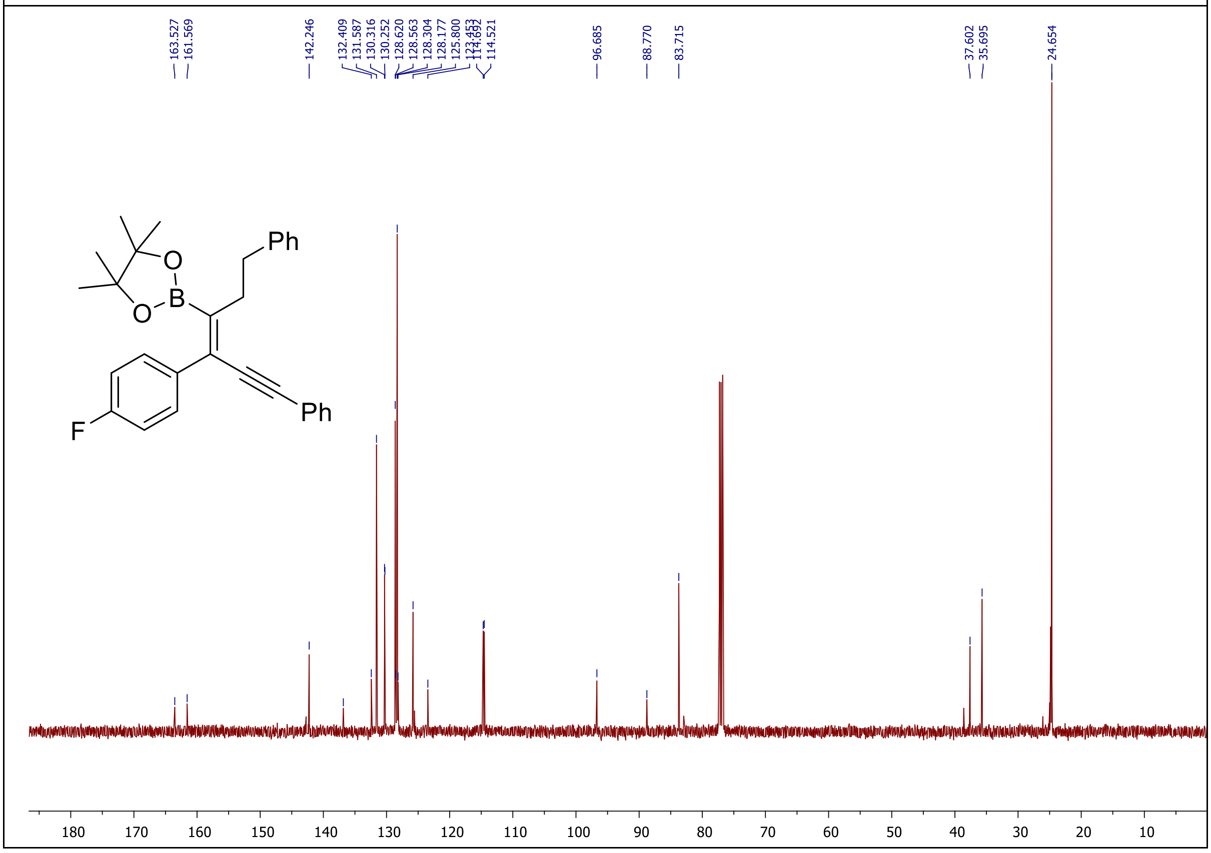

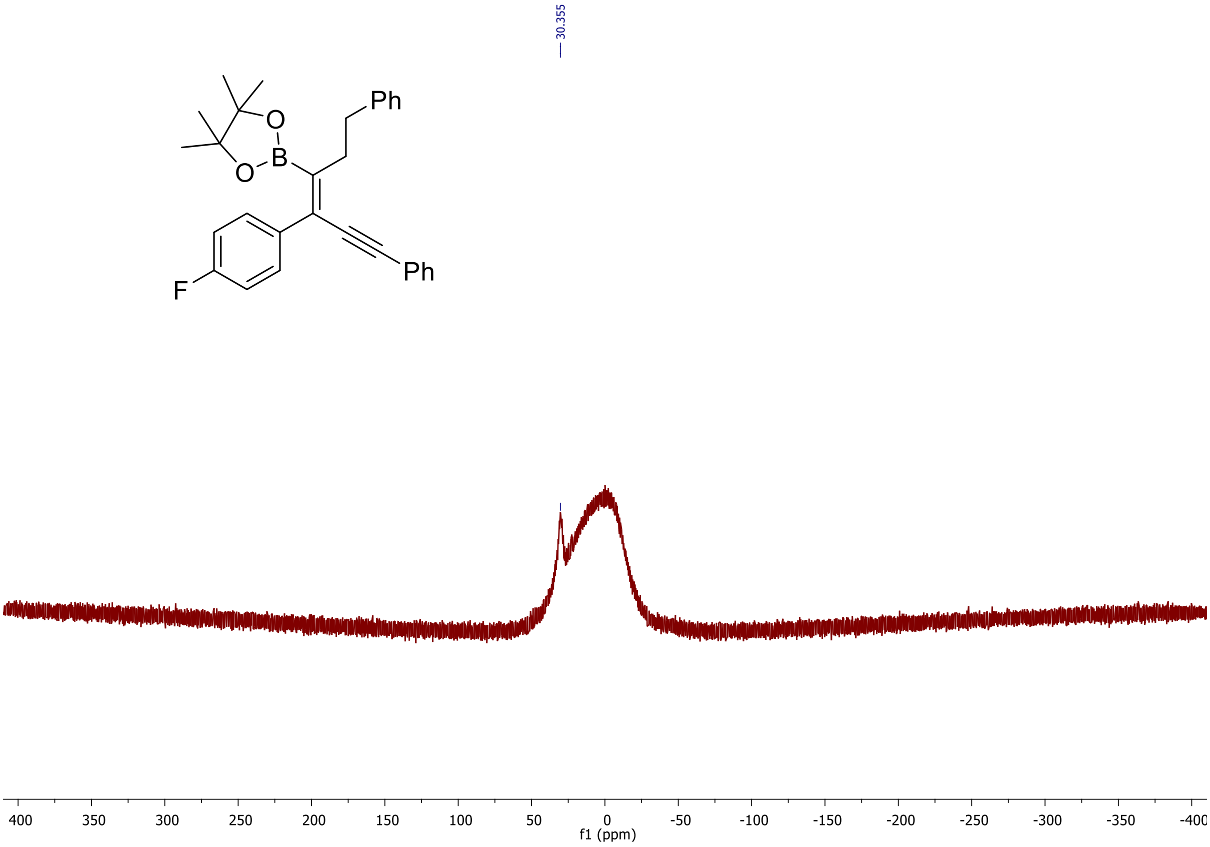
30.355
Ph
O
B
O
F
Ph
400 350 300 250 200 150 100 50 0 -50 -100 -150 -200 -250 -300 -350 -400
f1 (ppm)

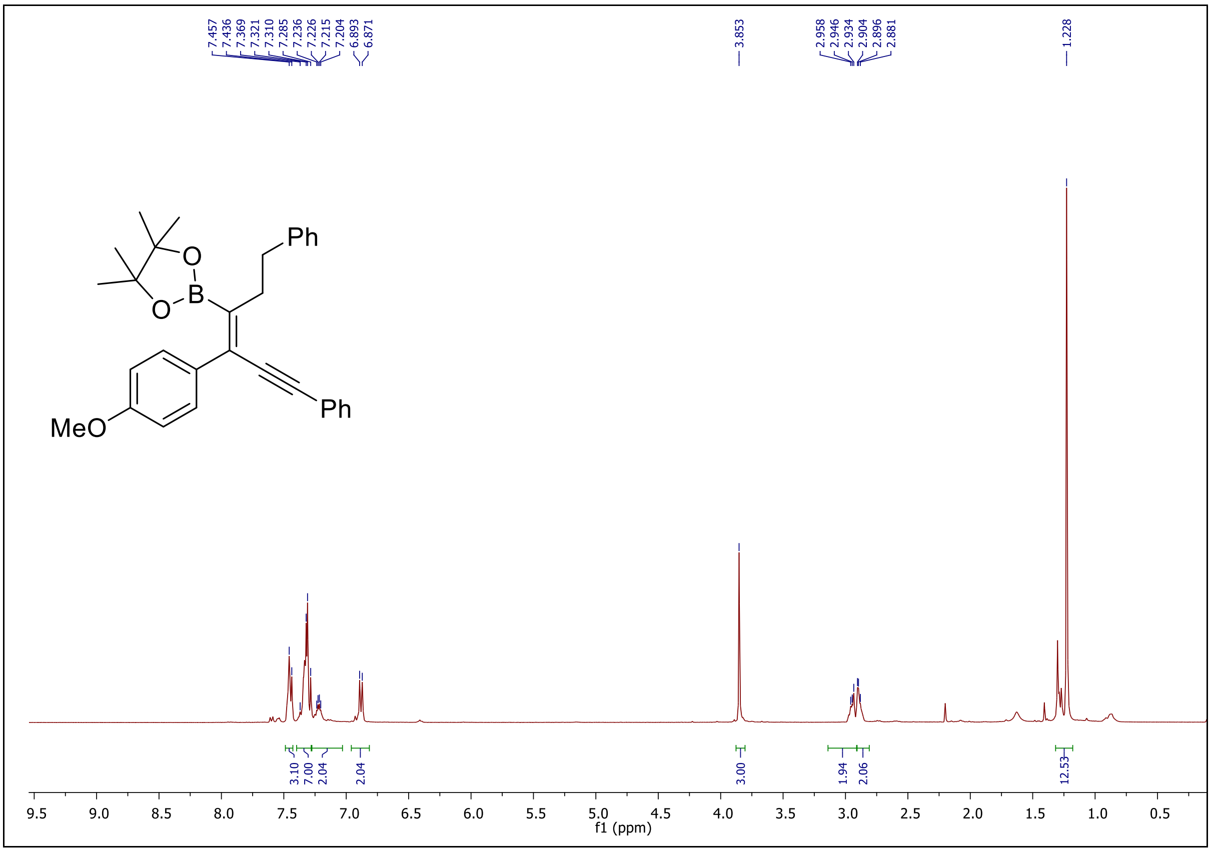
7.457
7.436
7.369
7.321
7.310
7.285
7.236
7.226
7.215
7.204
6.893
6.871
3.853
2.958
2.946
2.934
2.904
2.896
2.881
1.228
Ph
O
B
O
MeO
Ph
3.10
7.00
2.04
2.04
3.00
1.94
2.06
12.53
9.5 9.0 8.5 8.0 7.5 7.0 6.5 6.0 5.5 5.0 4.5 4.0 3.5 3.0 2.5 2.0 1.5 1.0 0.5
f1 (ppm)

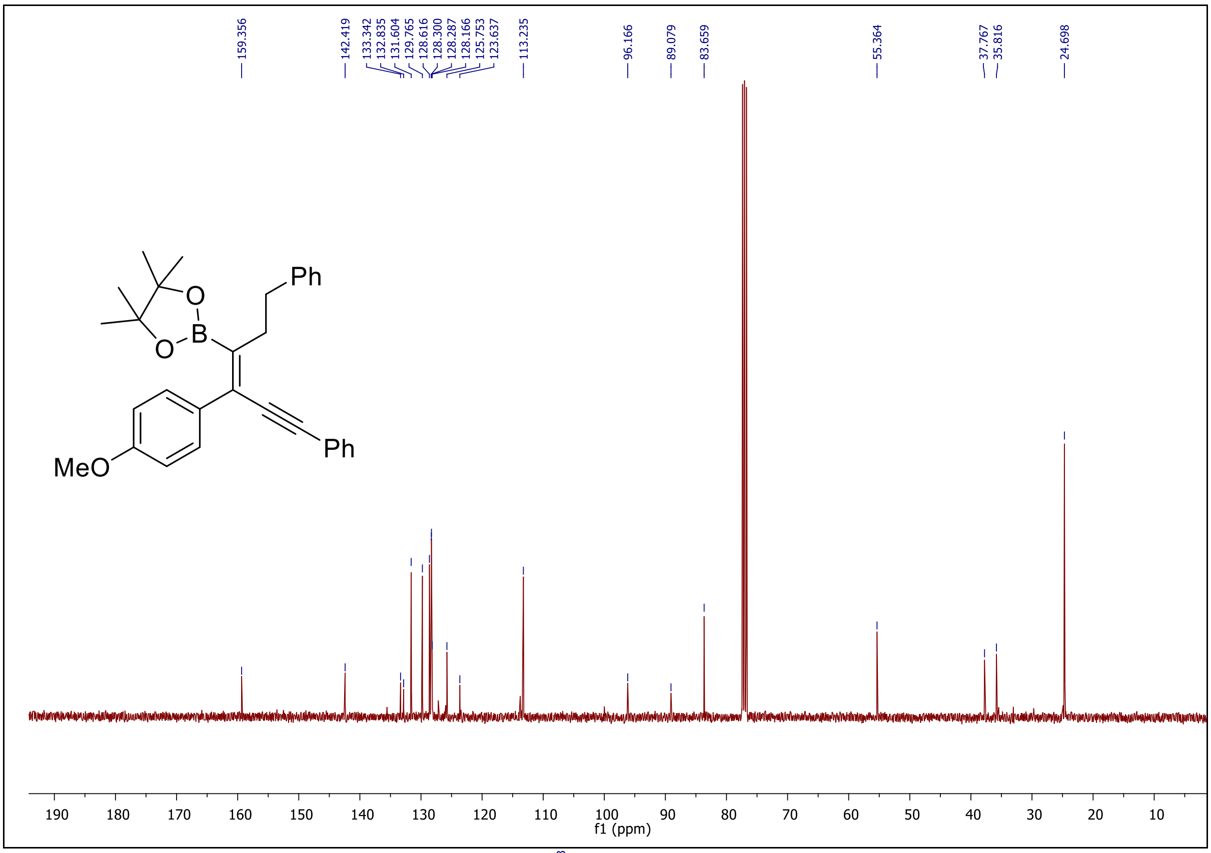
159.356
142.419
133.342
132.835
131.604
129.765
128.616
128.300
128.287
128.166
125.753
123.637
113.235
96.166
89.079
83.659
55.364
37.767
35.816
24.698
Ph
O
B
O
MeO
Ph
f1 (ppm)

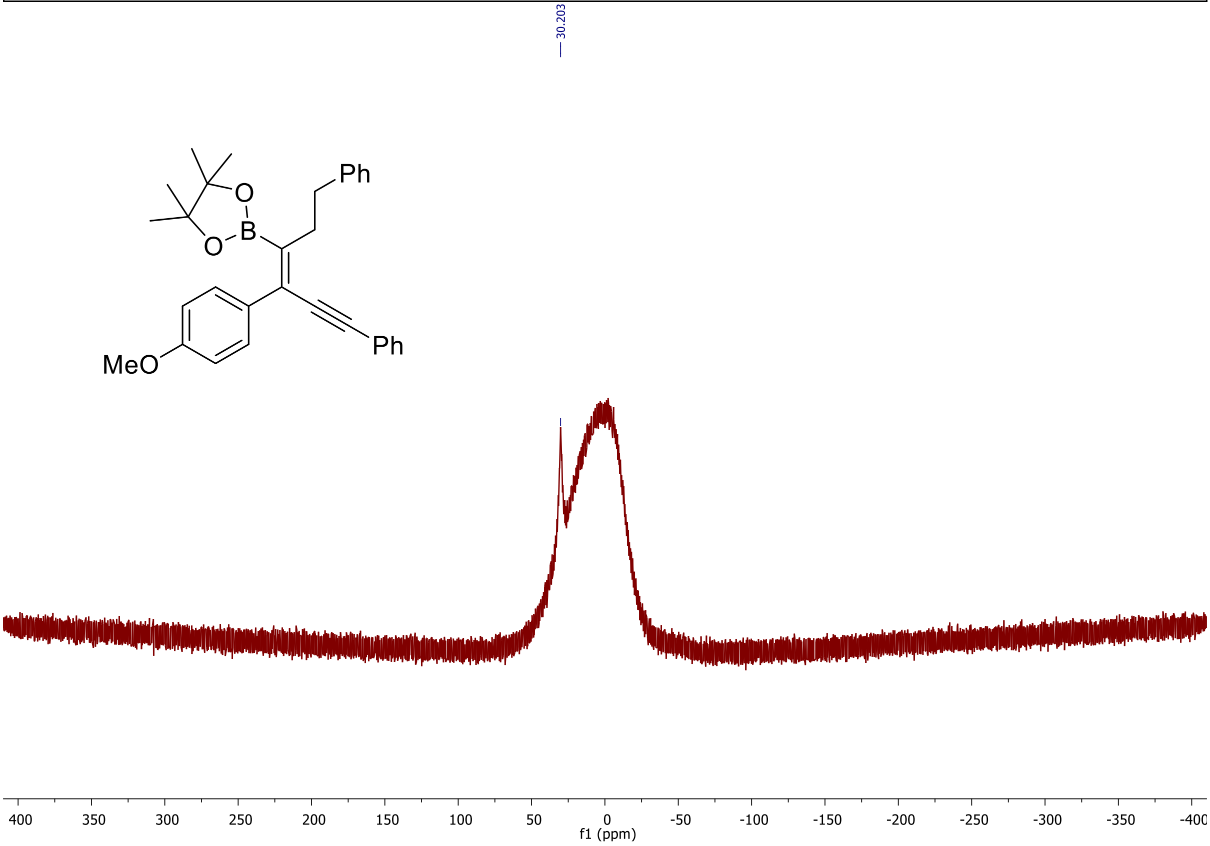
30.203
Ph
O
B
O
MeO
Ph
f1 (ppm)

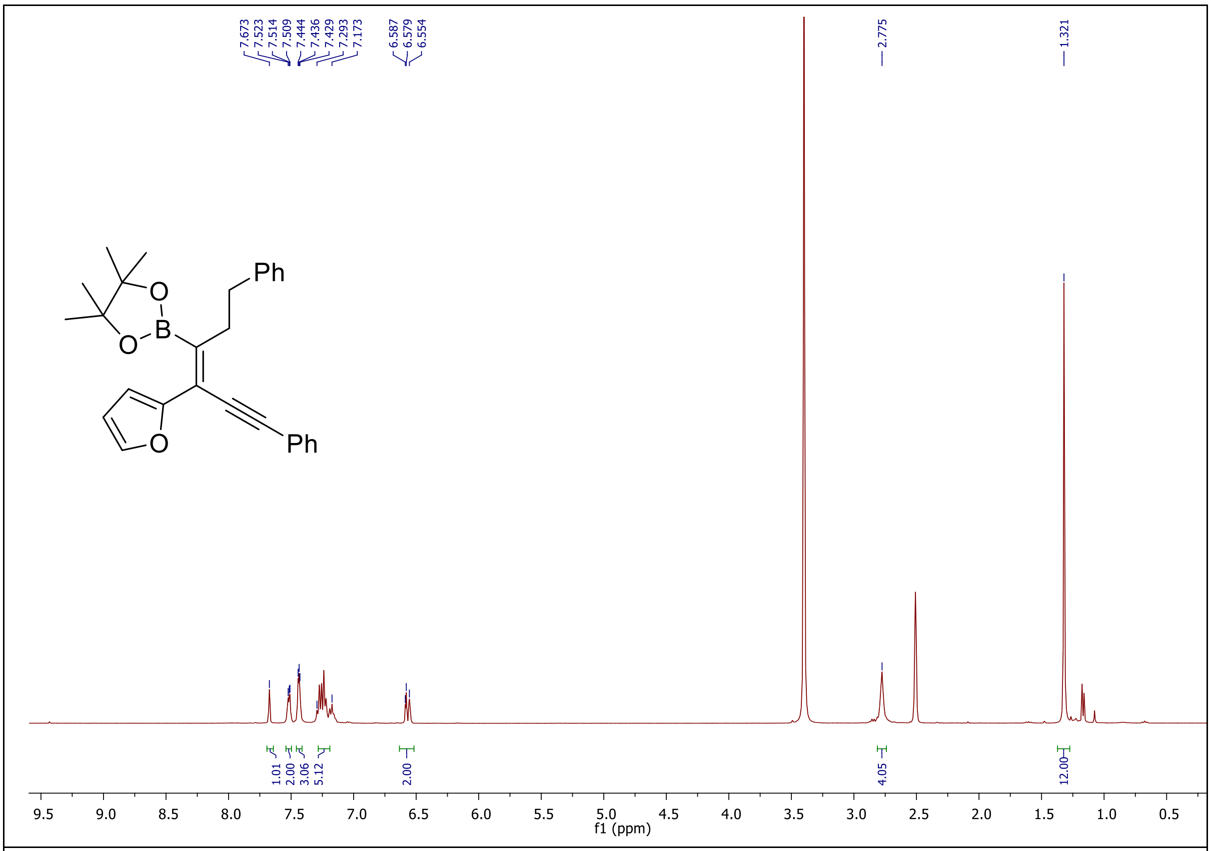
7.673
7.523
7.514
7.509
7.444
7.436
7.429
7.293
7.173
6.587
6.579
6.554
2.775
1.321
Ph
O
B
O
O
Ph
1.01
2.00
3.06
5.12
2.00
4.05
12.00
f1 (ppm)

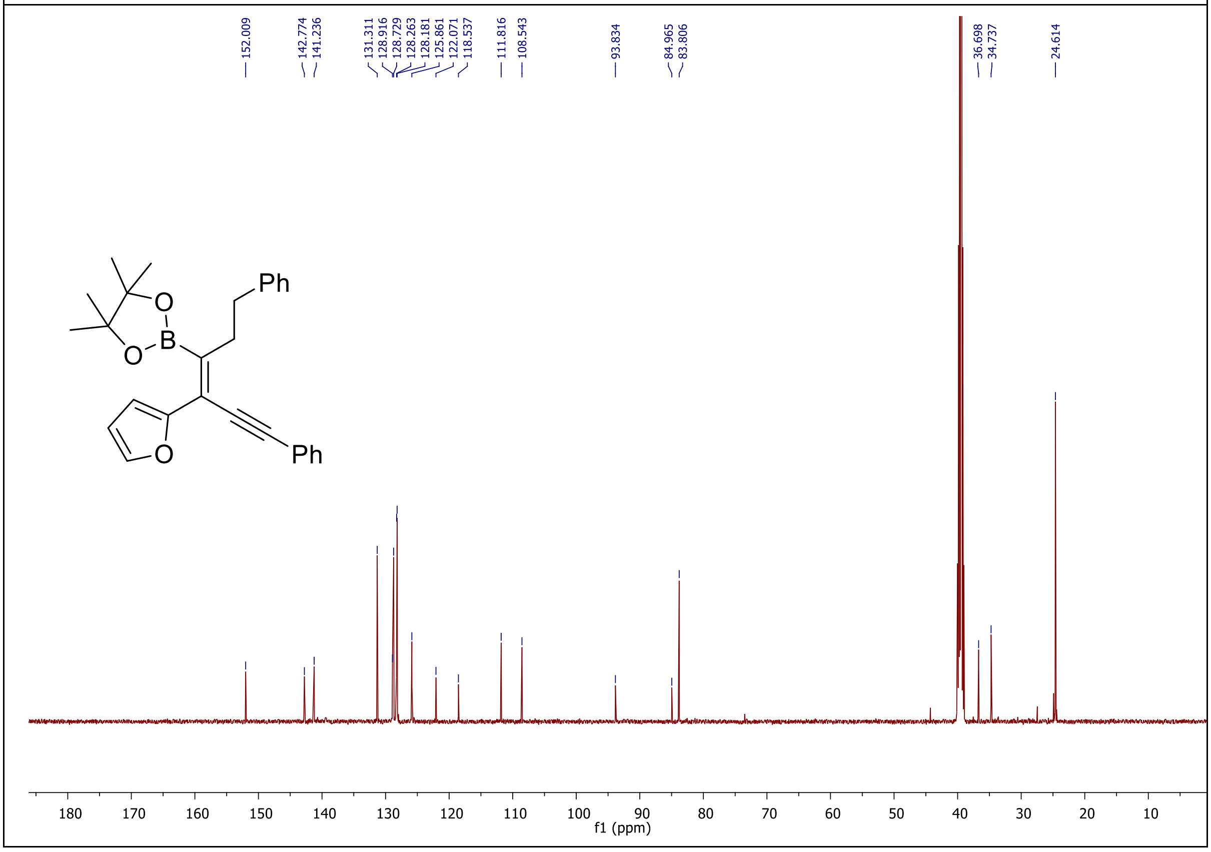
152.009
142.774
141.236
131.311
128.916
128.729
128.263
128.181
125.861
122.071
118.537
111.816
108.543
93.834
84.965
83.806
36.698
34.737
24.614
Ph
O
B
O
O
Ph
f1 (ppm)

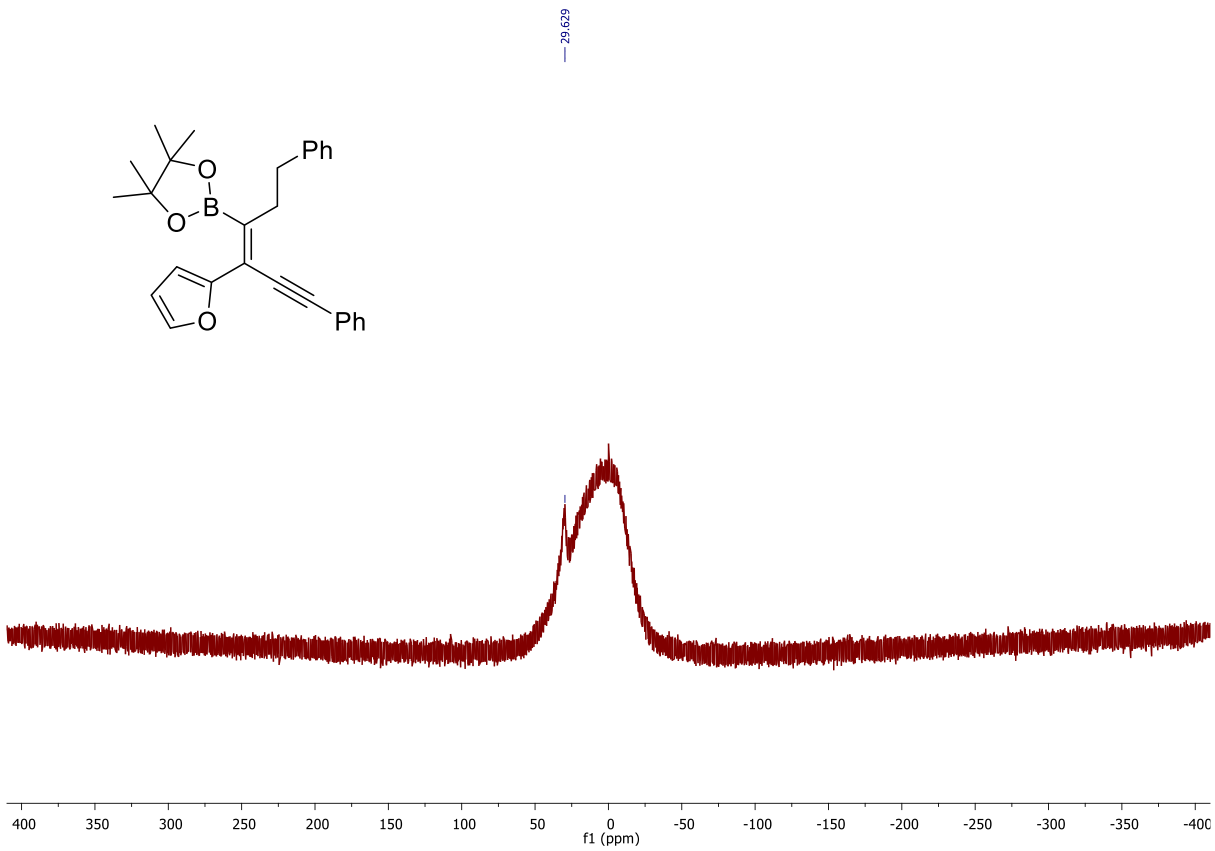

29.629
Ph
O
B
O
O
Ph
400
350
300
250
200
150
100
50
0
-50
-100
-150
-200
-250
-300
-350
-400
f1 (ppm)

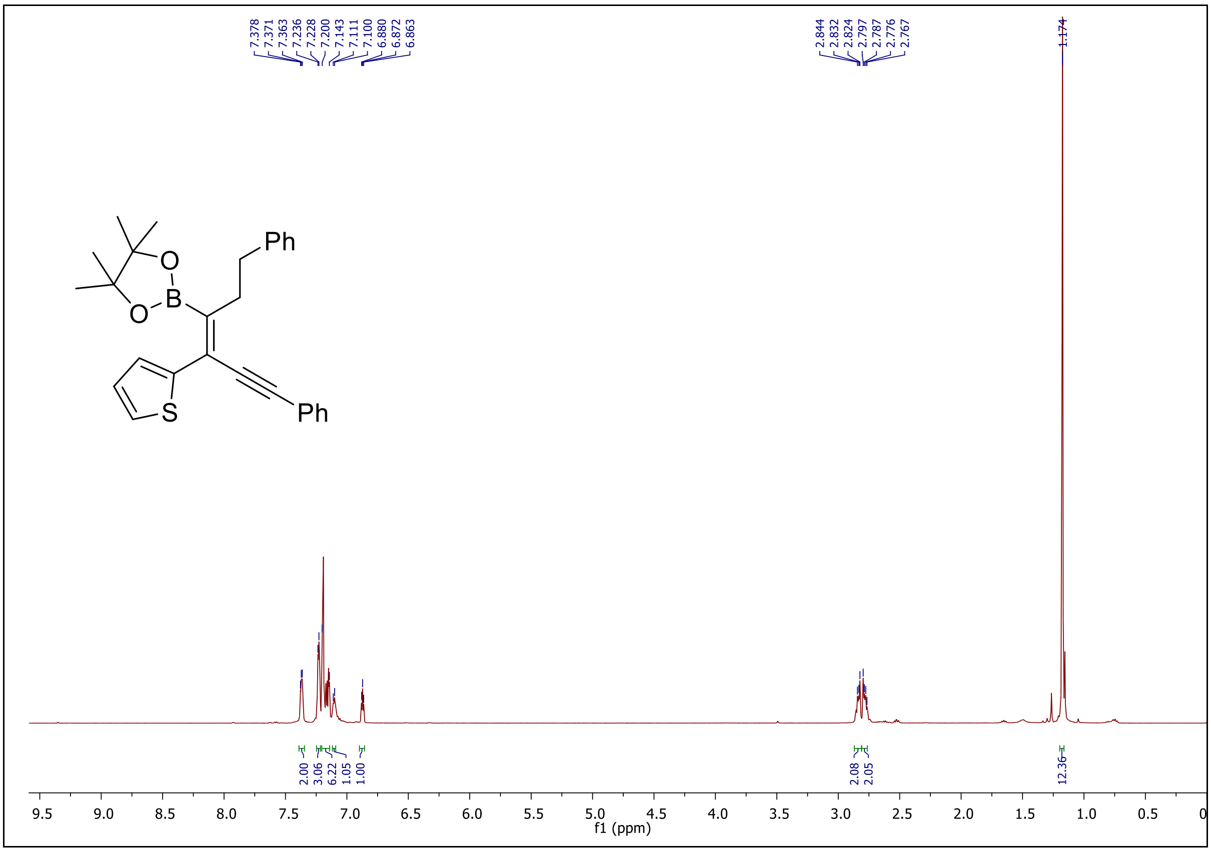

7.378
7.371
7.363
7.236
7.228
7.200
7.143
7.111
7.100
6.880
6.872
6.863
2.844
2.832
2.824
2.797
2.787
2.776
2.767
1.174
Ph
O
B
O
S
Ph
2.00
3.06
6.22
1.05
1.00
2.08
2.05
12.36
9.5
9.0
8.5
8.0
7.5
7.0
6.5
6.0
5.5
5.0
4.5
4.0
3.5
3.0
2.5
2.0
1.5
1.0
0.5
f1 (ppm)

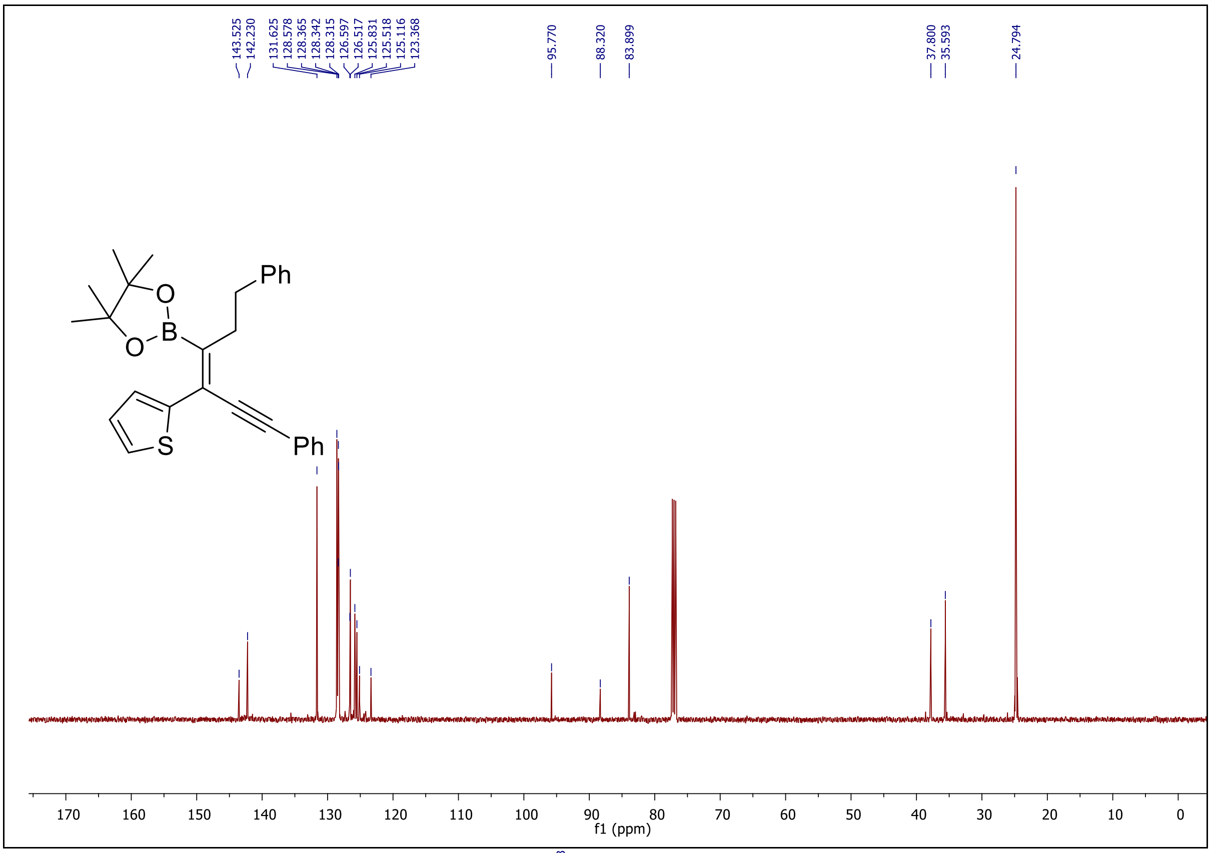
143.525
142.230
131.625
128.578
128.365
128.342
128.315
126.597
126.517
125.831
125.518
125.116
123.368
95.770
88.320
83.899
37.800
35.593
24.794
O
B
O
Ph
S
Ph
170 160 150 140 130 120 110 100 90 80 70 60 50 40 30 20 10 0
f1 (ppm)

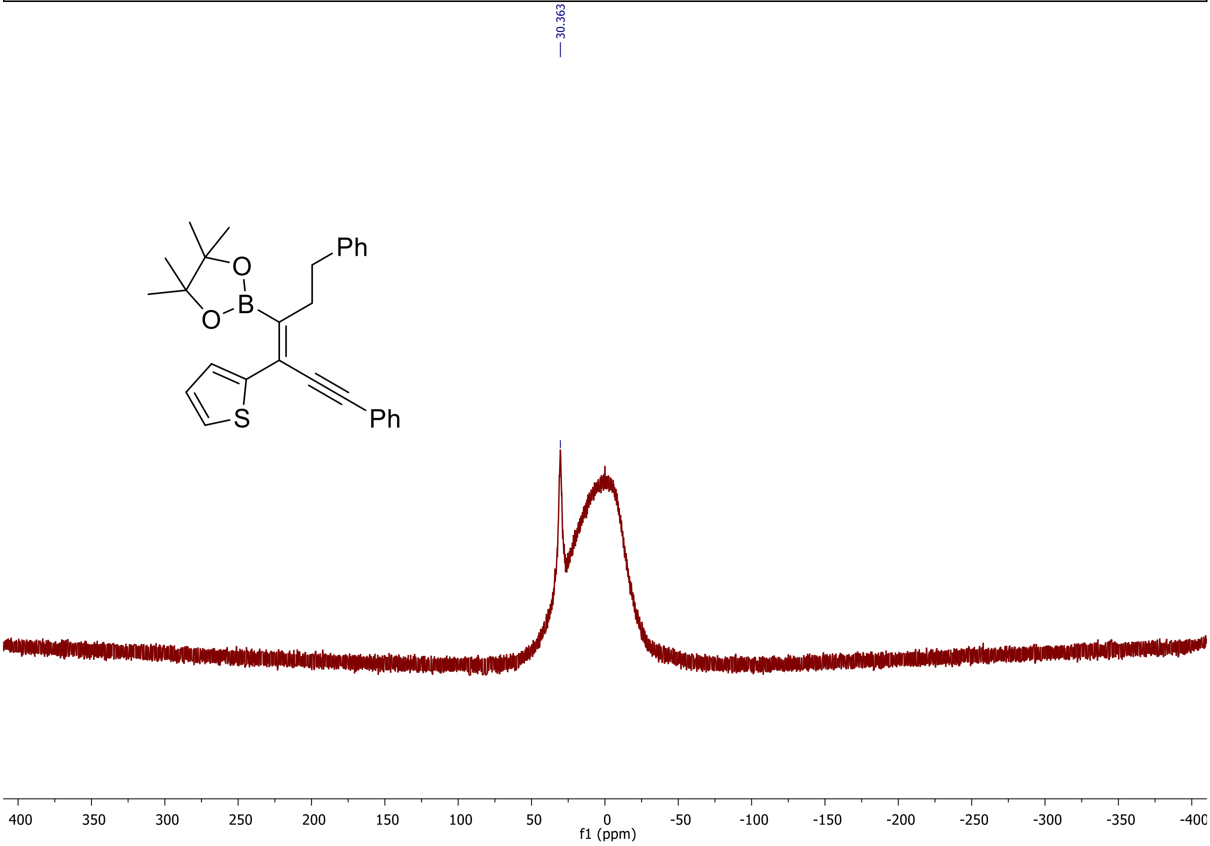
30.363
O
B
O
Ph
S
Ph
400 350 300 250 200 150 100 50 0 -50 -100 -150 -200 -250 -300 -350 -400
f1 (ppm)

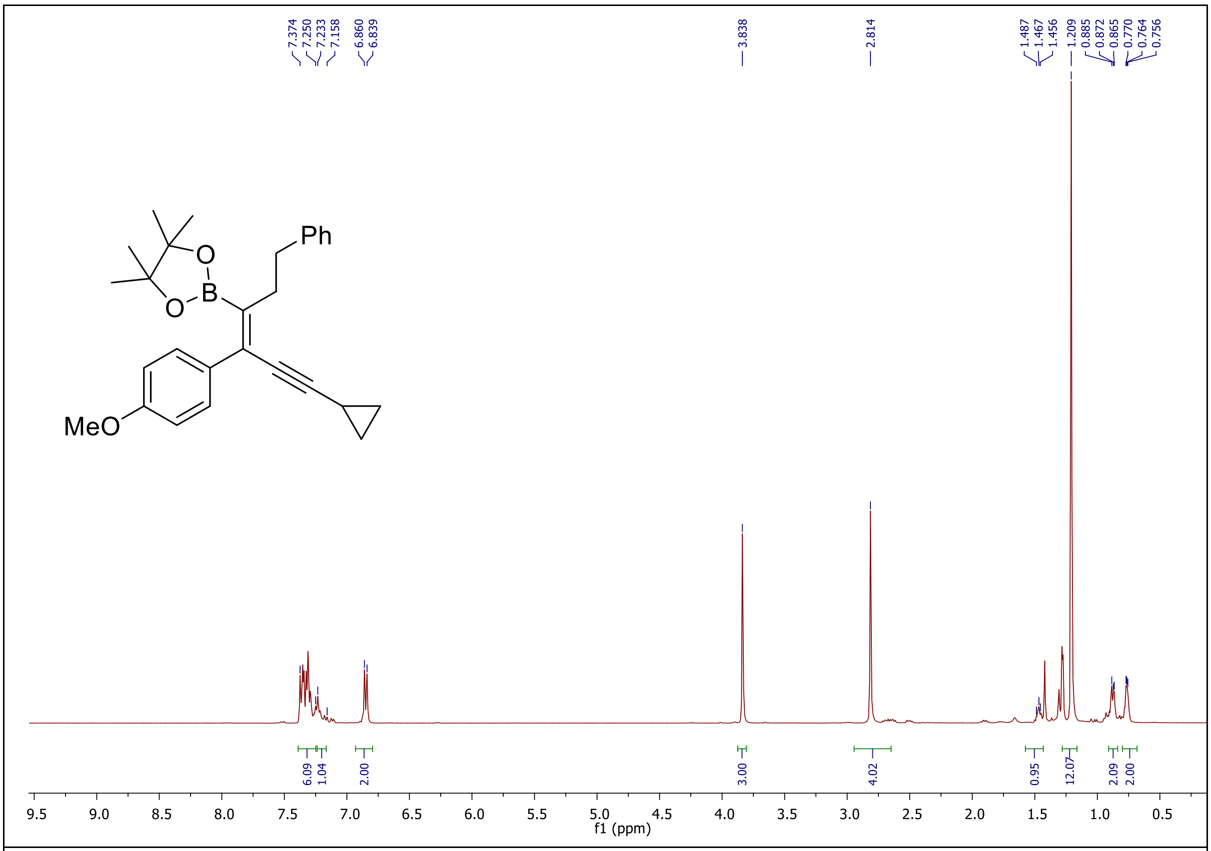
7.374
7.250
7.233
7.158
6.860
6.839
3.838
2.814
1.487
1.467
1.456
1.209
0.885
0.872
0.865
0.770
0.764
0.756
Ph
O
B
O
MeO
6.09
1.04
2.00
3.00
4.02
0.95
12.07
2.09
2.00
9.5
9.0
8.5
8.0
7.5
7.0
6.5
6.0
5.5
5.0
4.5
4.0
3.5
3.0
2.5
2.0
1.5
1.0
0.5
f1 (ppm)

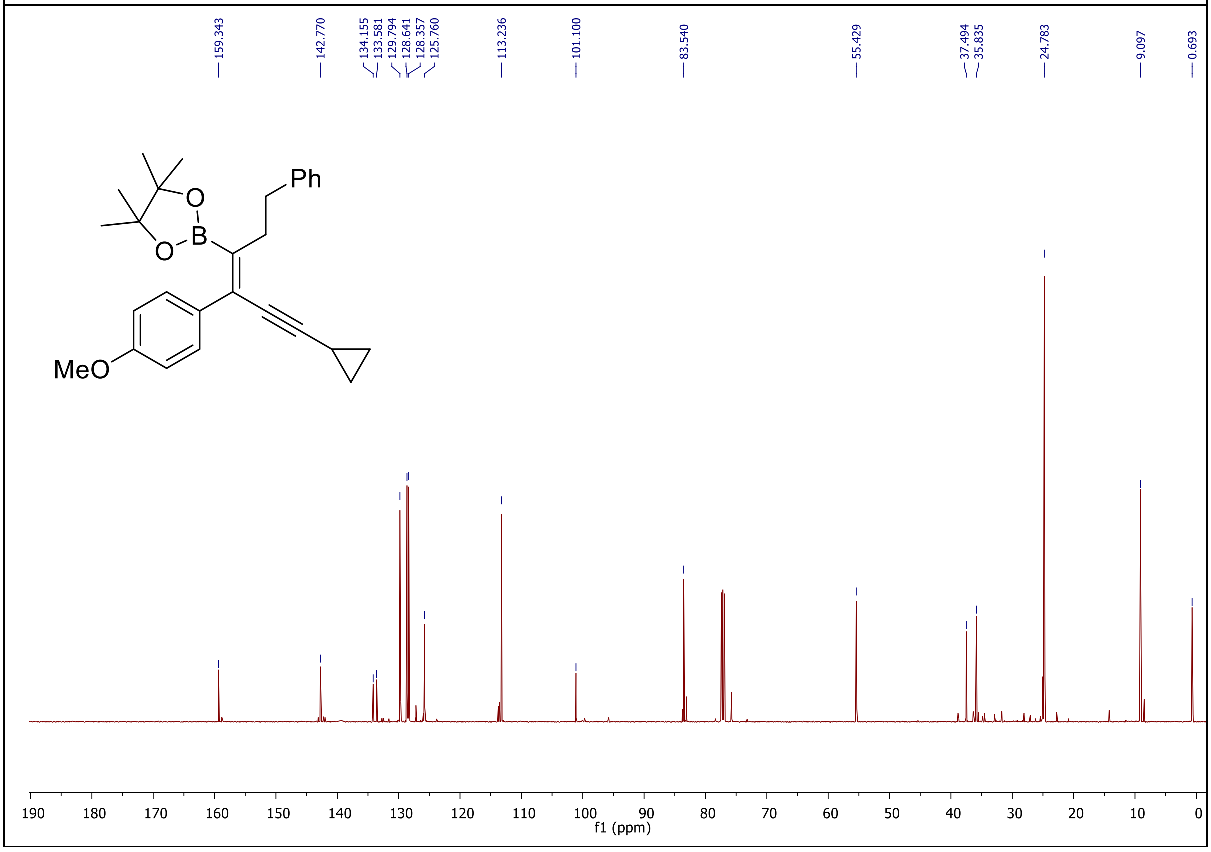
159.343
142.770
134.155
133.581
129.794
128.641
128.357
125.760
113.236
101.100
83.540
55.429
37.494
35.835
24.783
9.097
0.693
Ph
O
B
O
MeO
190
180
170
160
150
140
130
120
110
100
90
80
70
60
50
40
30
20
10
0
f1 (ppm)

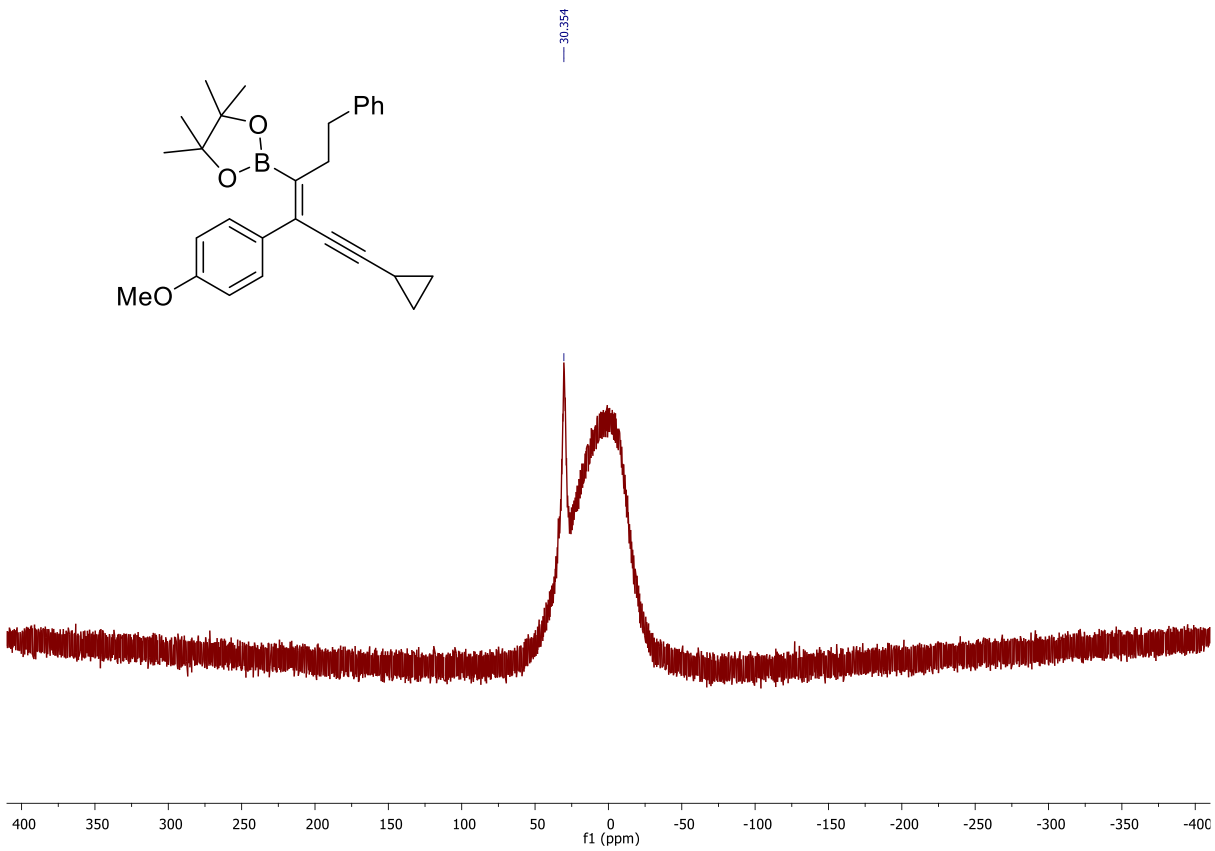

30.354
Ph
O
B
O
MeO
400 350 300 250 200 150 100 50 0 -50 -100 -150 -200 -250 -300 -350 -400
f1 (ppm)

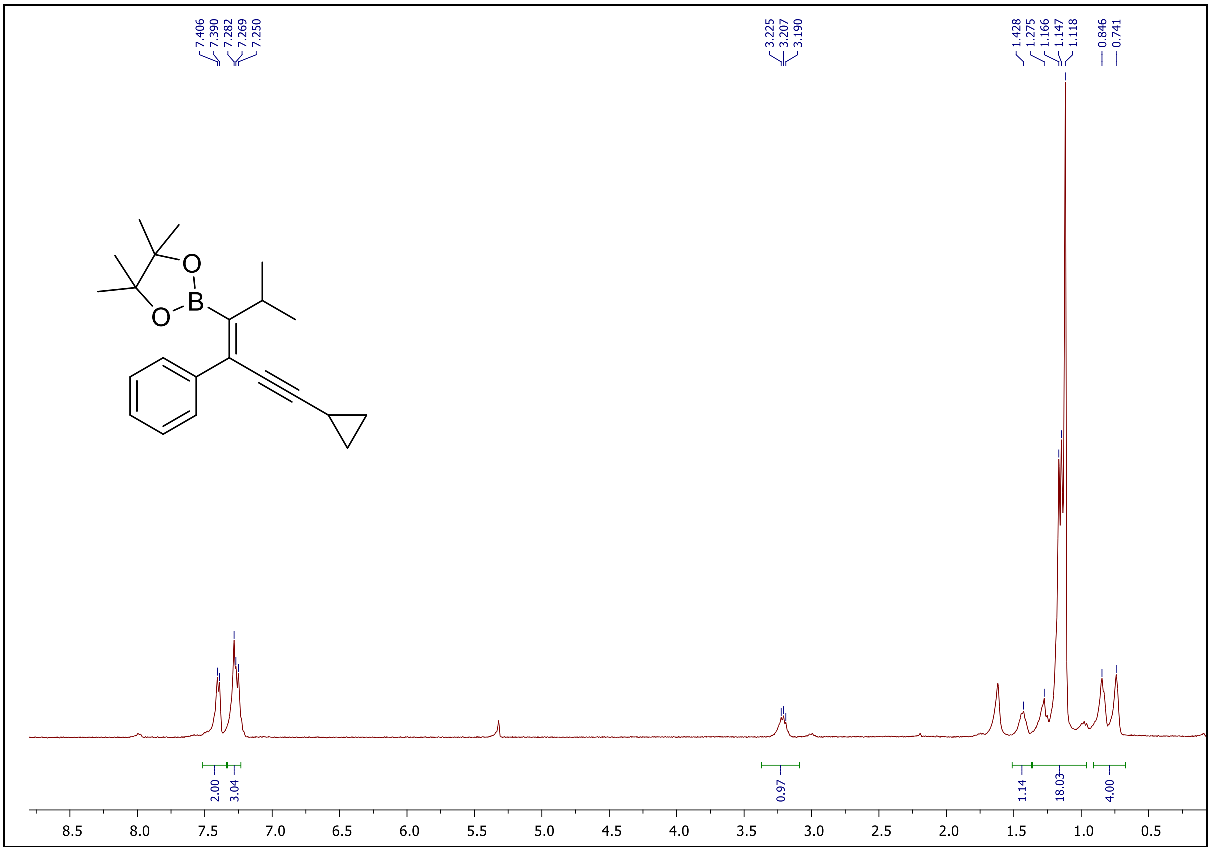

7.406
7.390
7.282
7.269
7.250
3.225
3.207
3.190
1.428
1.275
1.166
1.147
1.118
0.846
0.741
O
B
O
2.00
3.04
0.97
1.14
18.03
4.00
8.5 8.0 7.5 7.0 6.5 6.0 5.5 5.0 4.5 4.0 3.5 3.0 2.5 2.0 1.5 1.0 0.5

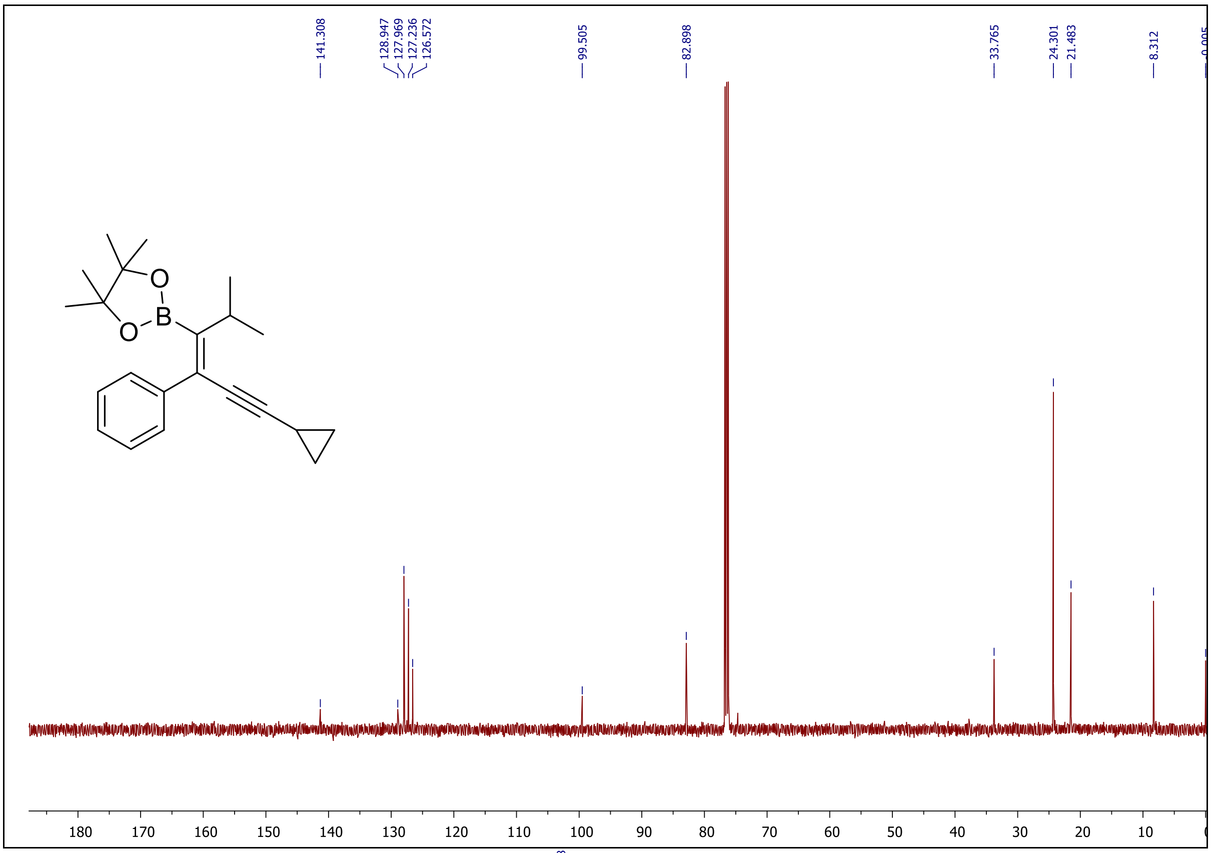

141.308
128.947
127.969
127.236
126.572
99.505
82.898
33.765
24.301
21.483
8.312
180
170
160
150
140
130
120
110
100
90
80
70
60
50
40
30
20
10
O
B
O

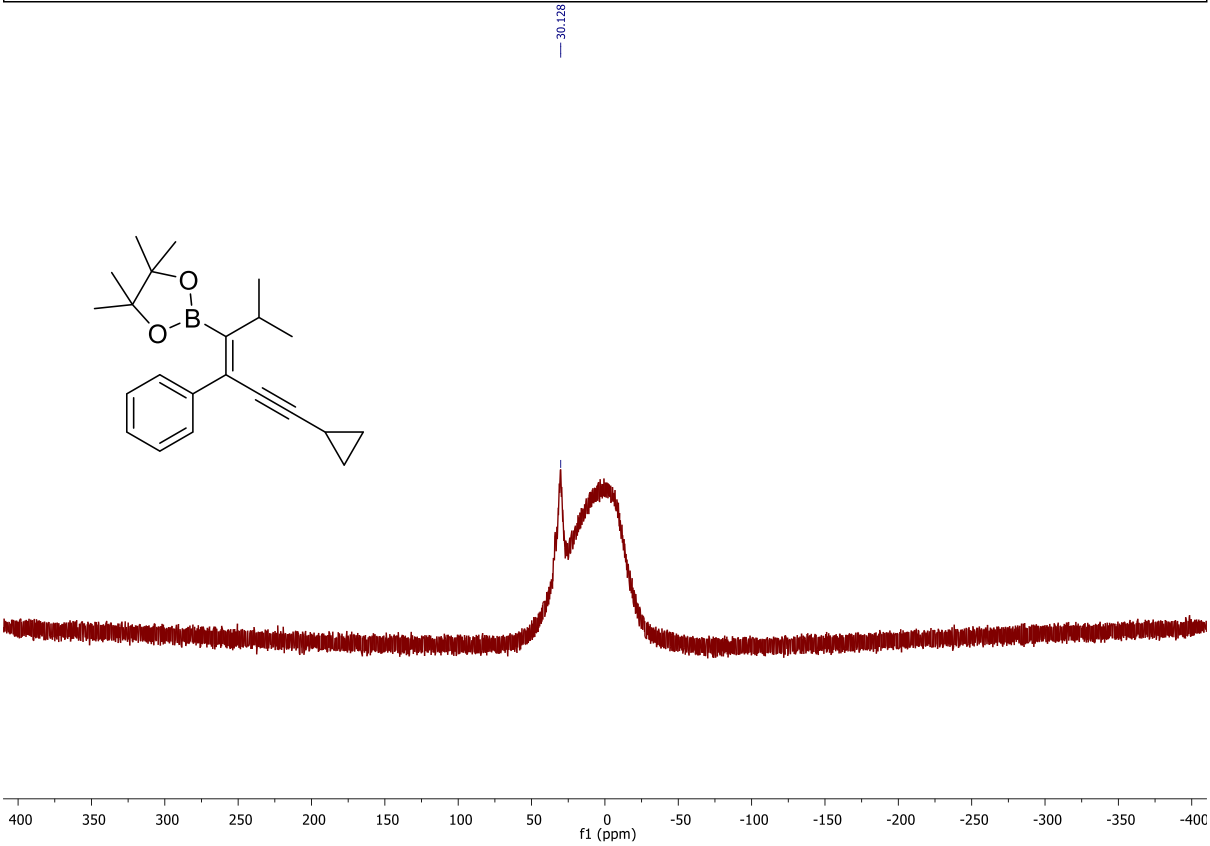

30.128
O
B
O
400
350
300
250
200
150
100
50
0
-50
-100
-150
-200
-250
-300
-350
f1 (ppm)

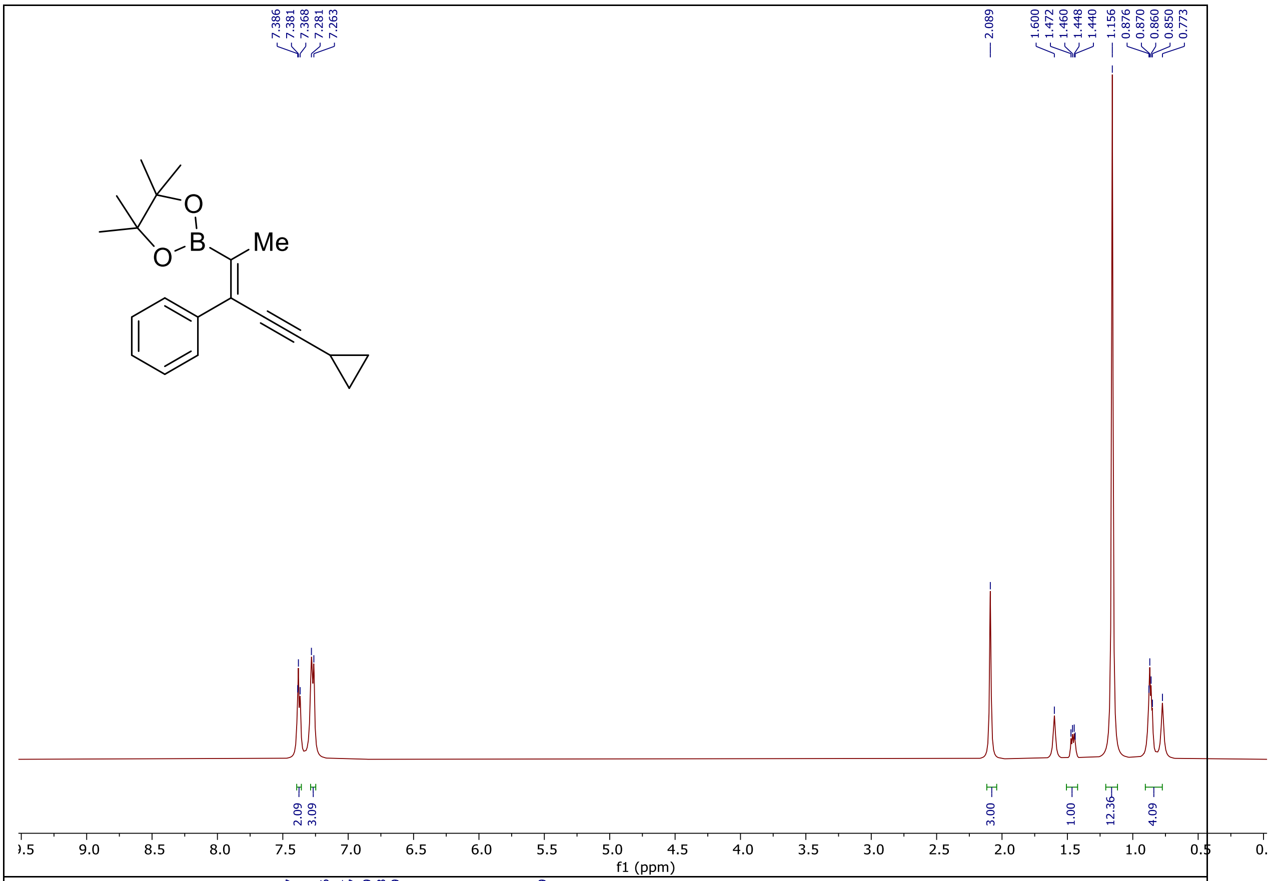
7.386
7.381
7.368
7.281
7.263
2.089
1.600
1.472
1.460
1.448
1.440
1.156
0.876
0.870
0.860
0.850
0.773
O
B
O
Me
2.09
3.09
3.00
1.00
12.36
4.09
f1 (ppm)

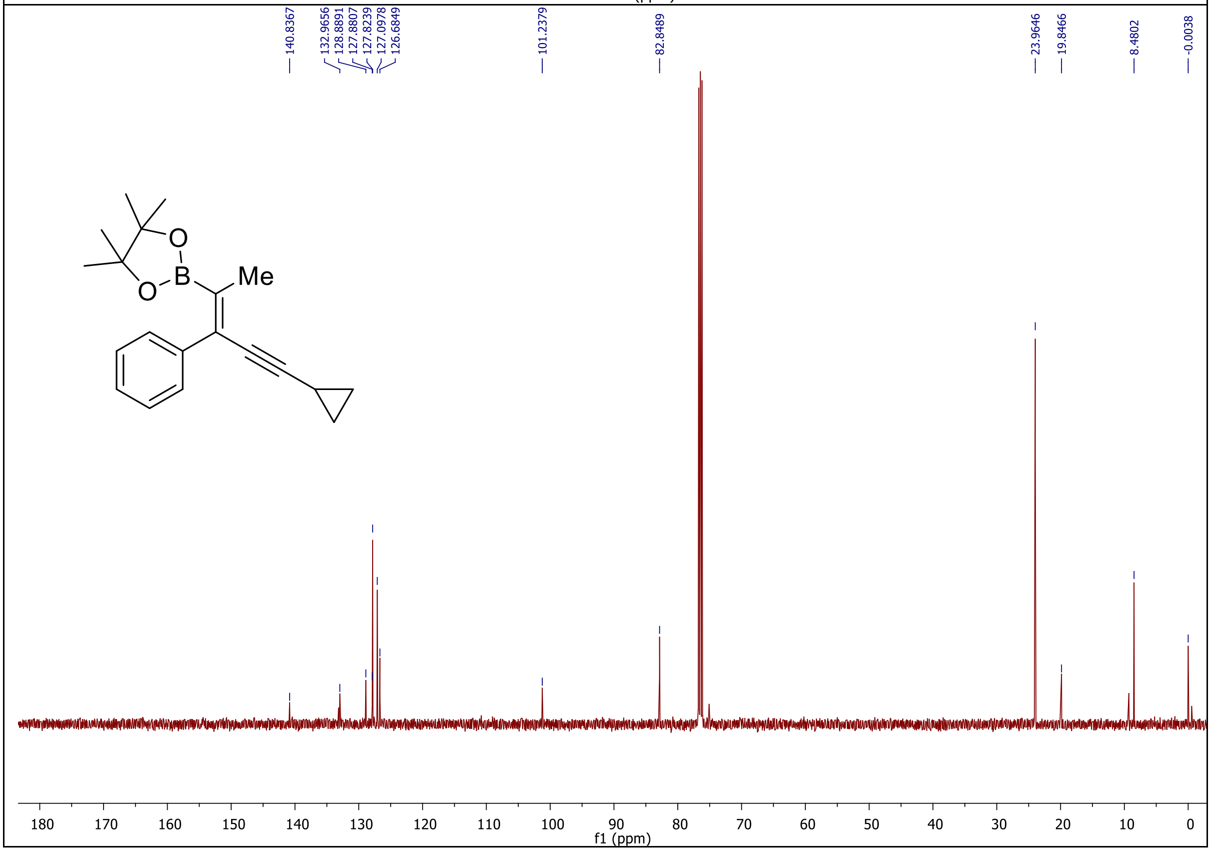
140.8367
132.9656
128.8891
127.8807
127.8239
127.0978
126.6849
101.2379
82.8489
23.9646
19.8466
8.4802
-0.0038
O
B
O
Me
f1 (ppm)

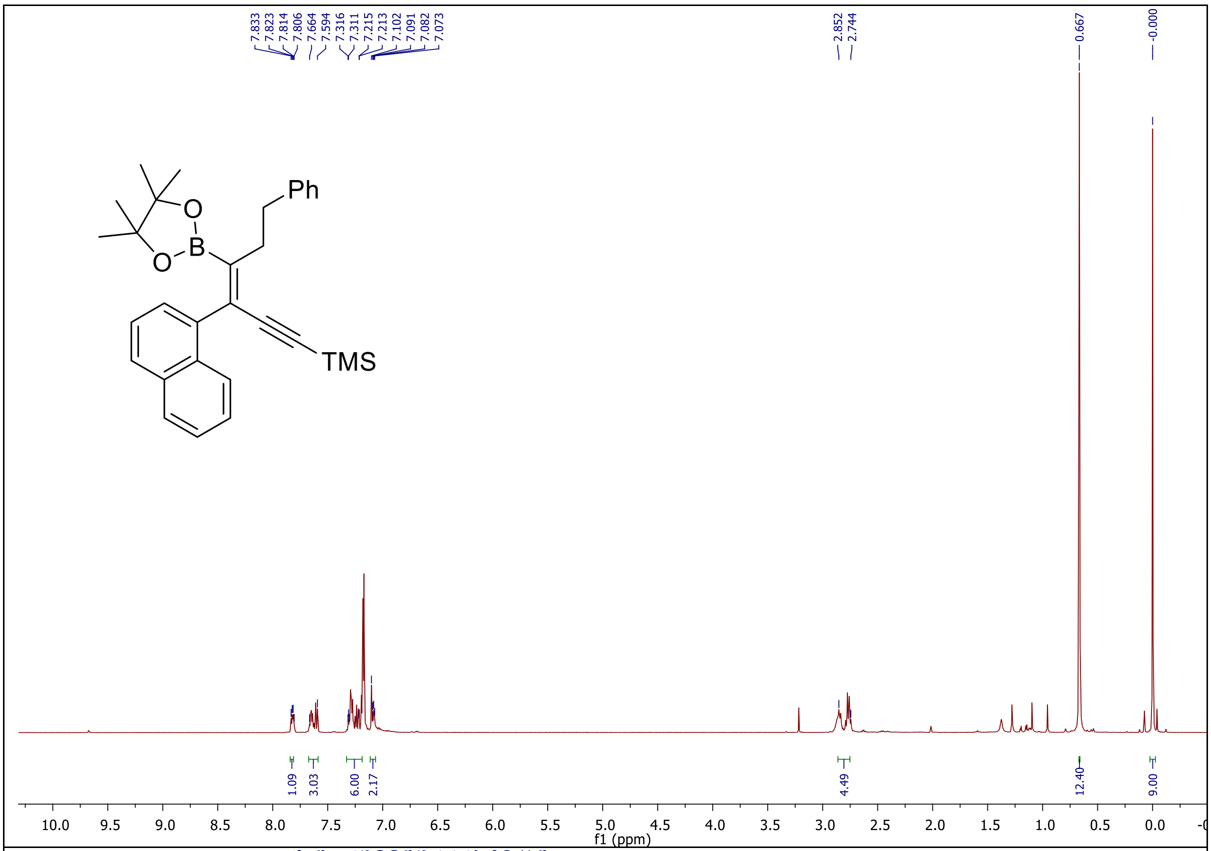

7.833
7.823
7.814
7.806
7.664
7.594
7.316
7.311
7.215
7.213
7.102
7.091
7.082
7.073
2.852
2.744
0.667
-0.000
Ph
O
B
O
TMS
1.09
3.03
6.00
2.17
4.49
12.40
9.00
f1 (ppm)

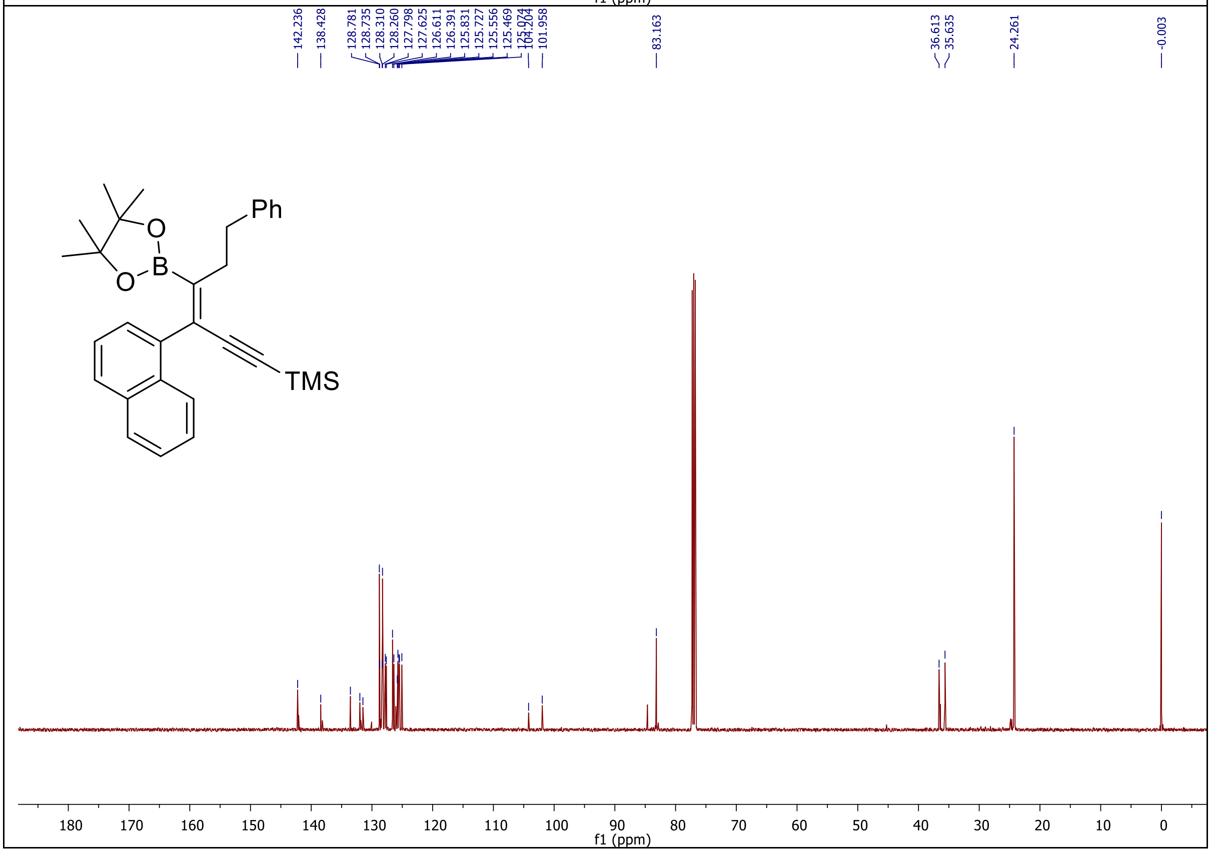

142.236
138.428
128.781
128.735
128.310
128.260
127.798
127.625
126.611
126.391
125.831
125.727
125.556
125.469
104.204
101.958
83.163
36.613
35.635
24.261
-0.003
Ph
O
B
O
TMS
f1 (ppm)

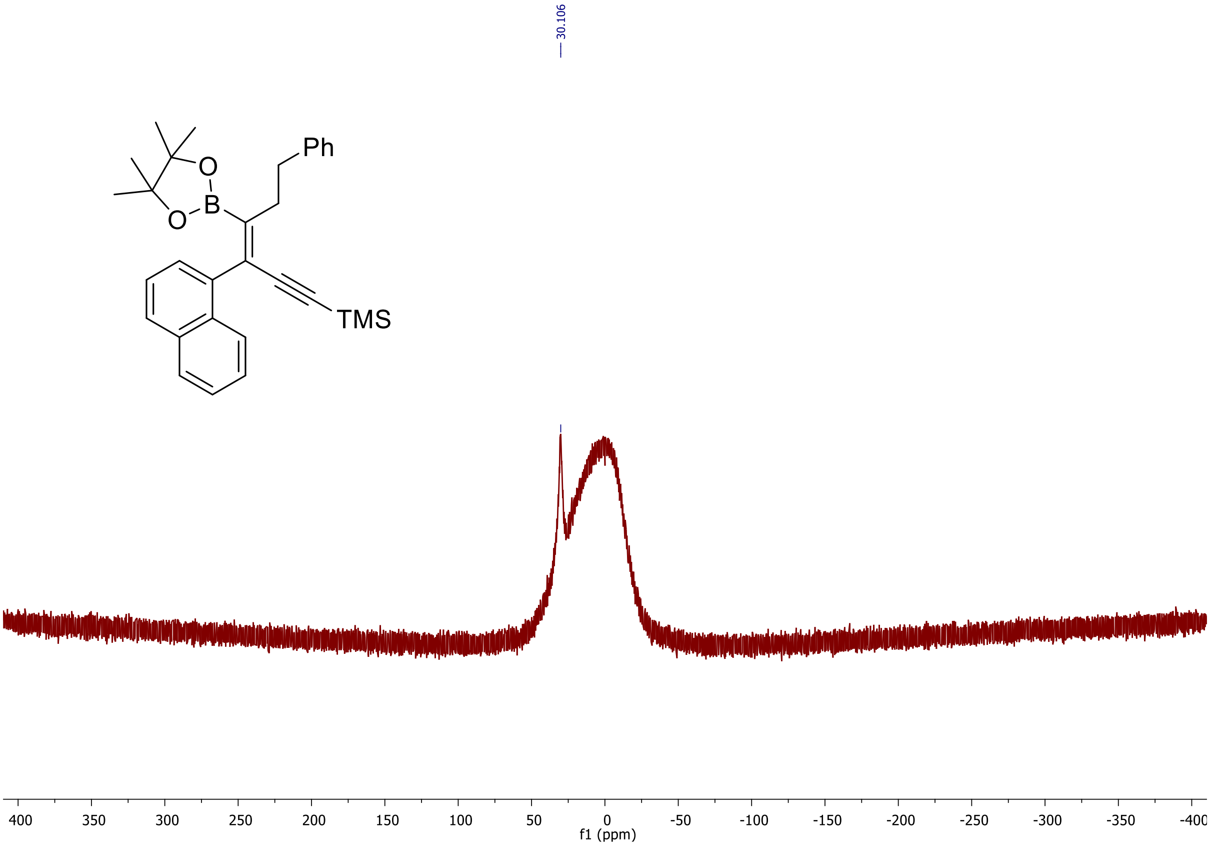

30.106
O
B
O
Ph
TMS
400
350
300
250
200
150
100
50
0
-50
-100
-150
-200
-250
-300
-350
-400
f1 (ppm)

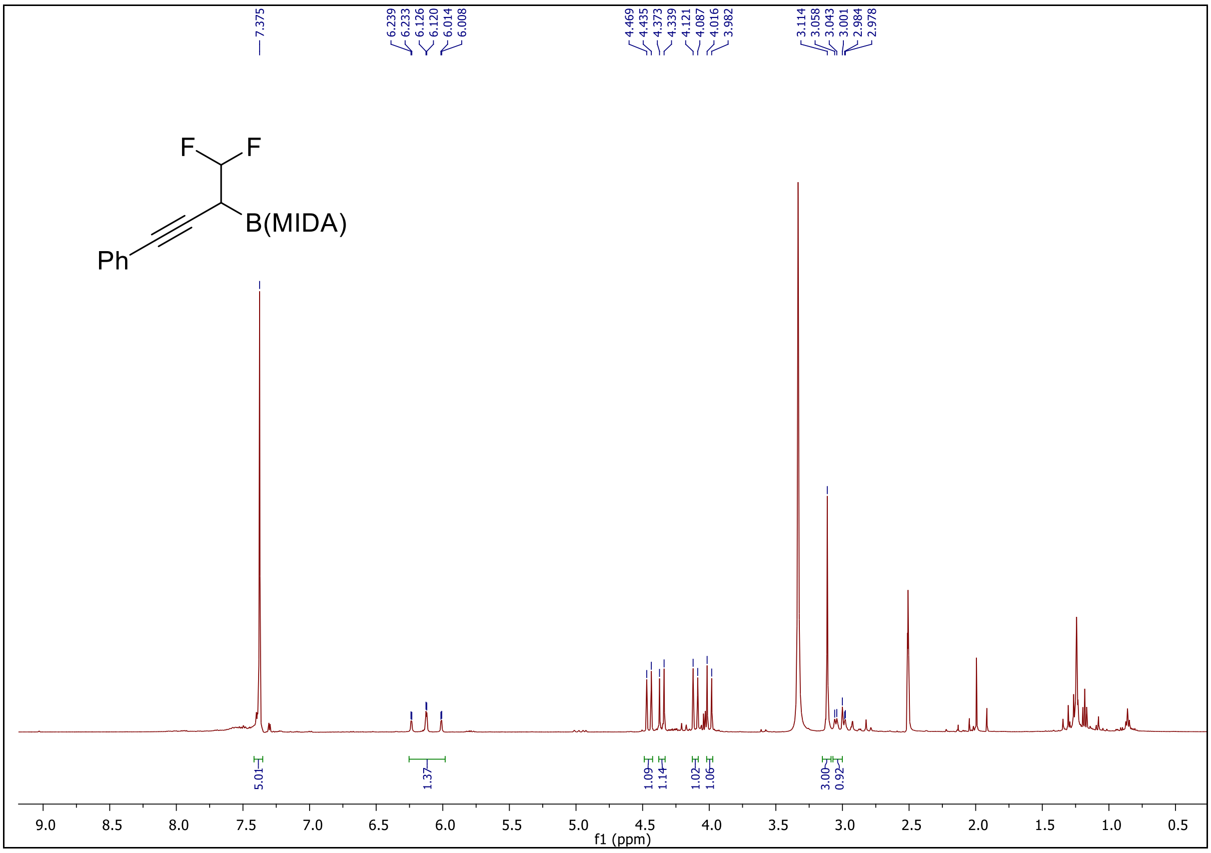

7.375
6.239
6.233
6.126
6.120
6.014
6.008
4.469
4.435
4.373
4.339
4.121
4.087
4.016
3.982
3.114
3.058
3.043
3.001
2.984
2.978
F
F
B(MIDA)
Ph
5.01
1.37
1.09
1.14
1.02
1.06
3.00
0.92
9.0
8.5
8.0
7.5
7.0
6.5
6.0
5.5
5.0
4.5
4.0
3.5
3.0
2.5
2.0
1.5
1.0
0.5
f1 (ppm)

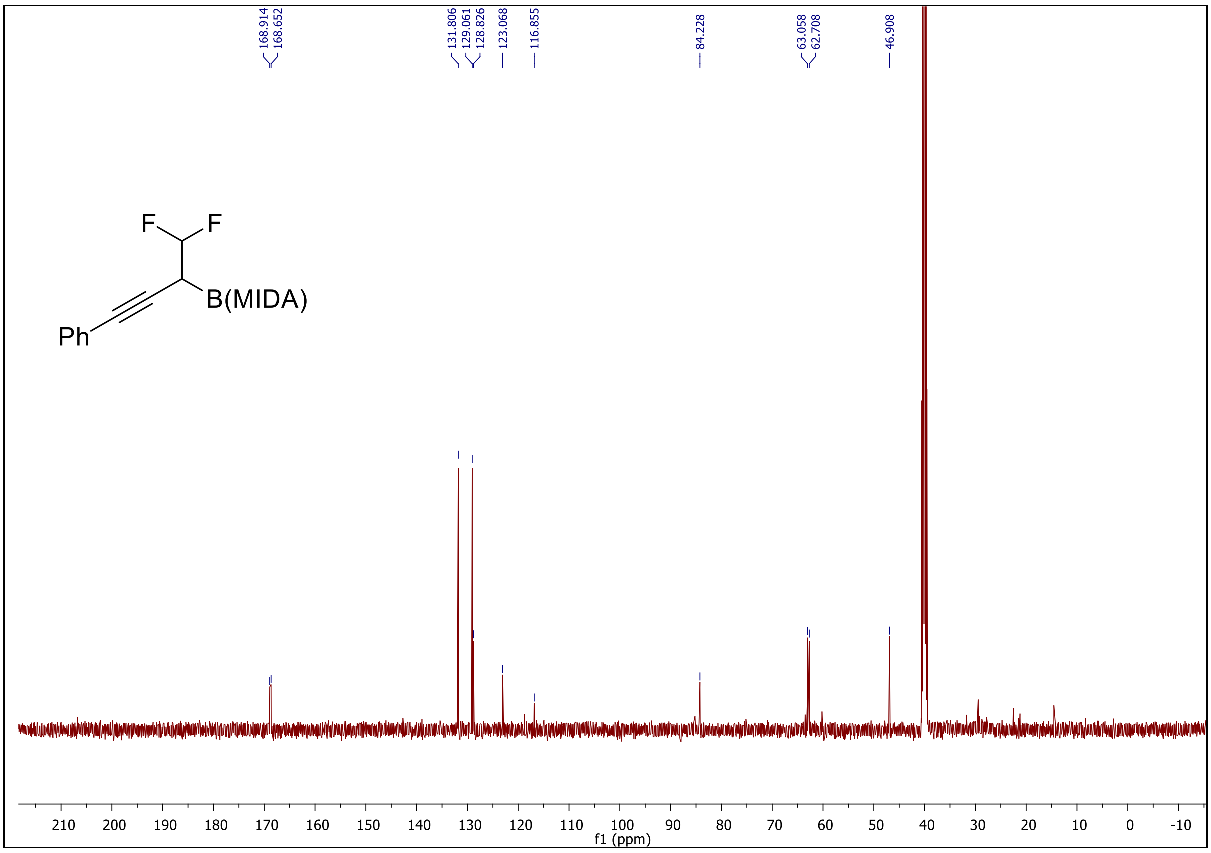

168.914
168.652
131.806
129.061
128.826
123.068
116.855
84.228
63.058
62.708
46.908
F
F
B(MIDA)
Ph
210 200 190 180 170 160 150 140 130 120 110 100 90 80 70 60 50 40 30 20 10 0 -10
f1 (ppm)

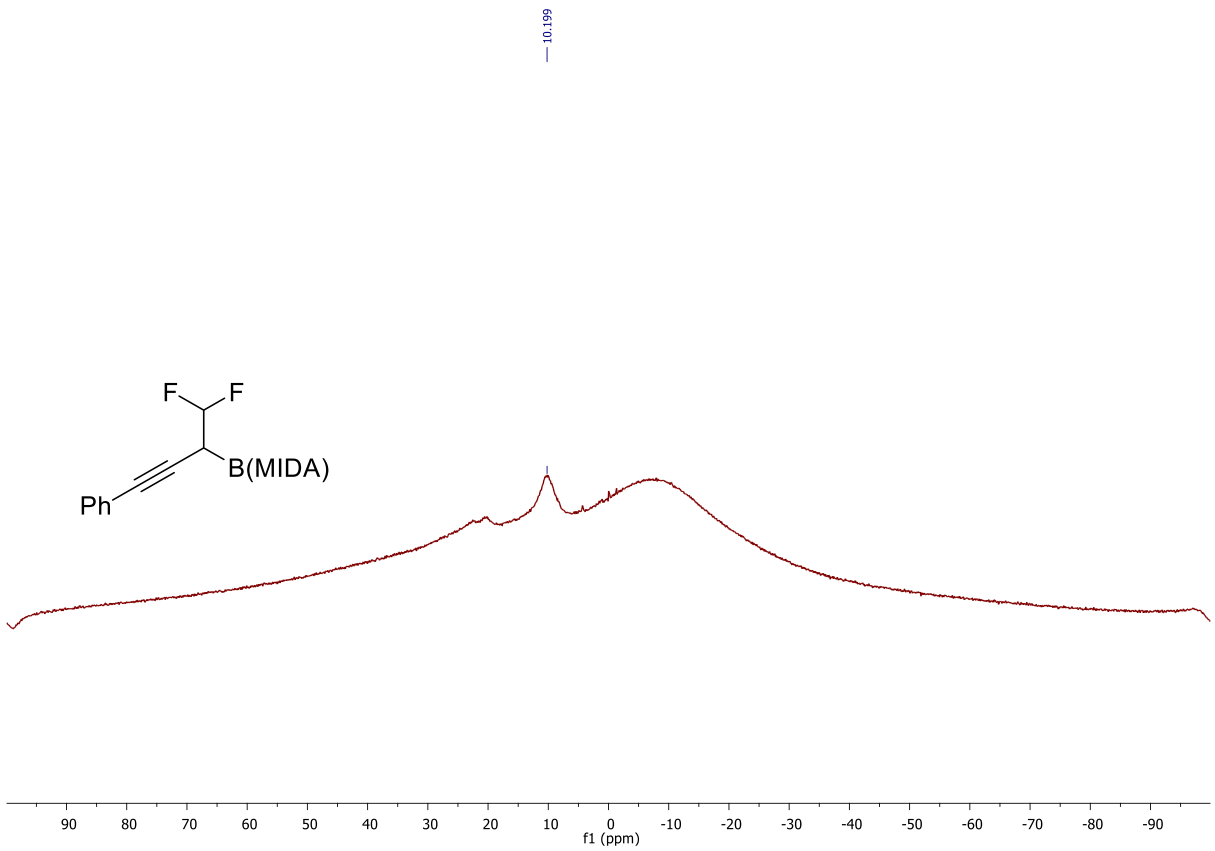

10.199
F
F
B(MIDA)
Ph
90
80
70
60
50
40
30
20
10
0
-10
-20
-30
-40
-50
-60
-70
-80
-90
f1 (ppm)

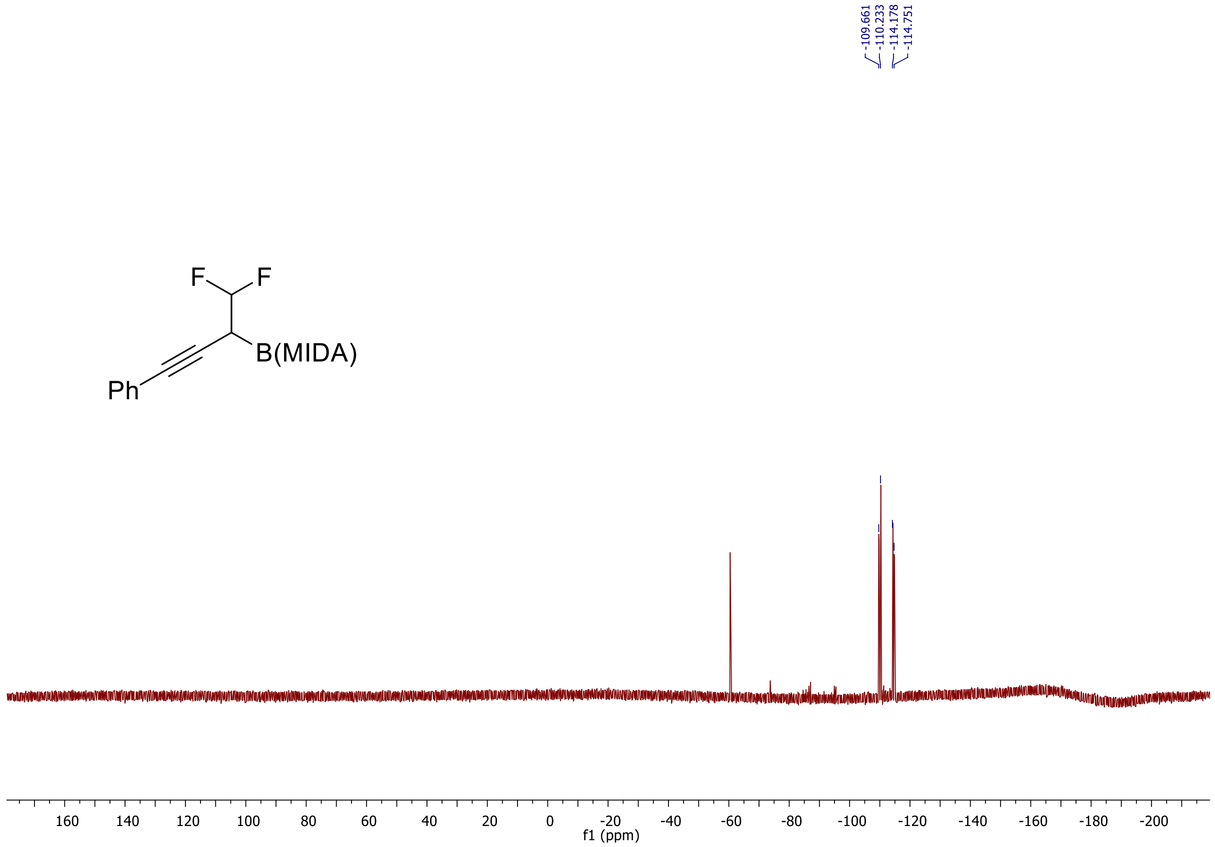

-109.661
-110.233
-114.178
-114.751
F
F
B(MIDA)
Ph
160
140
120
100
80
60
40
20
0
-20
-40
-60
-80
-100
-120
-140
-160
-180
-200
f1 (ppm)

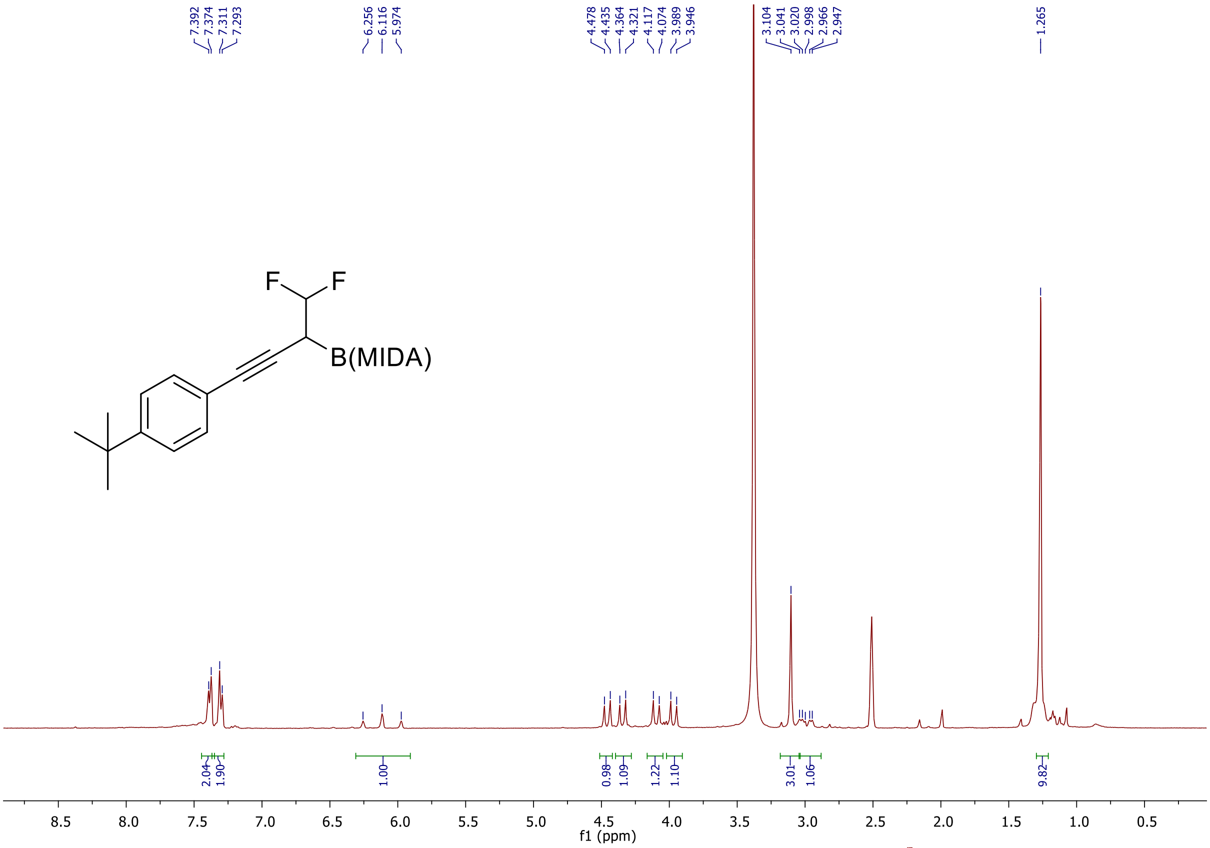
7.392
7.374
7.311
7.293
6.256
6.116
5.974
4.478
4.435
4.364
4.321
4.117
4.074
3.989
3.946
3.104
3.041
3.020
2.998
2.966
2.947
1.265
F
F
B(MIDA)
2.04
1.90
1.00
0.98
1.09
1.22
1.10
3.01
1.06
9.82
8.5 8.0 7.5 7.0 6.5 6.0 5.5 5.0 4.5 4.0 3.5 3.0 2.5 2.0 1.5 1.0 0.5
f1 (ppm)

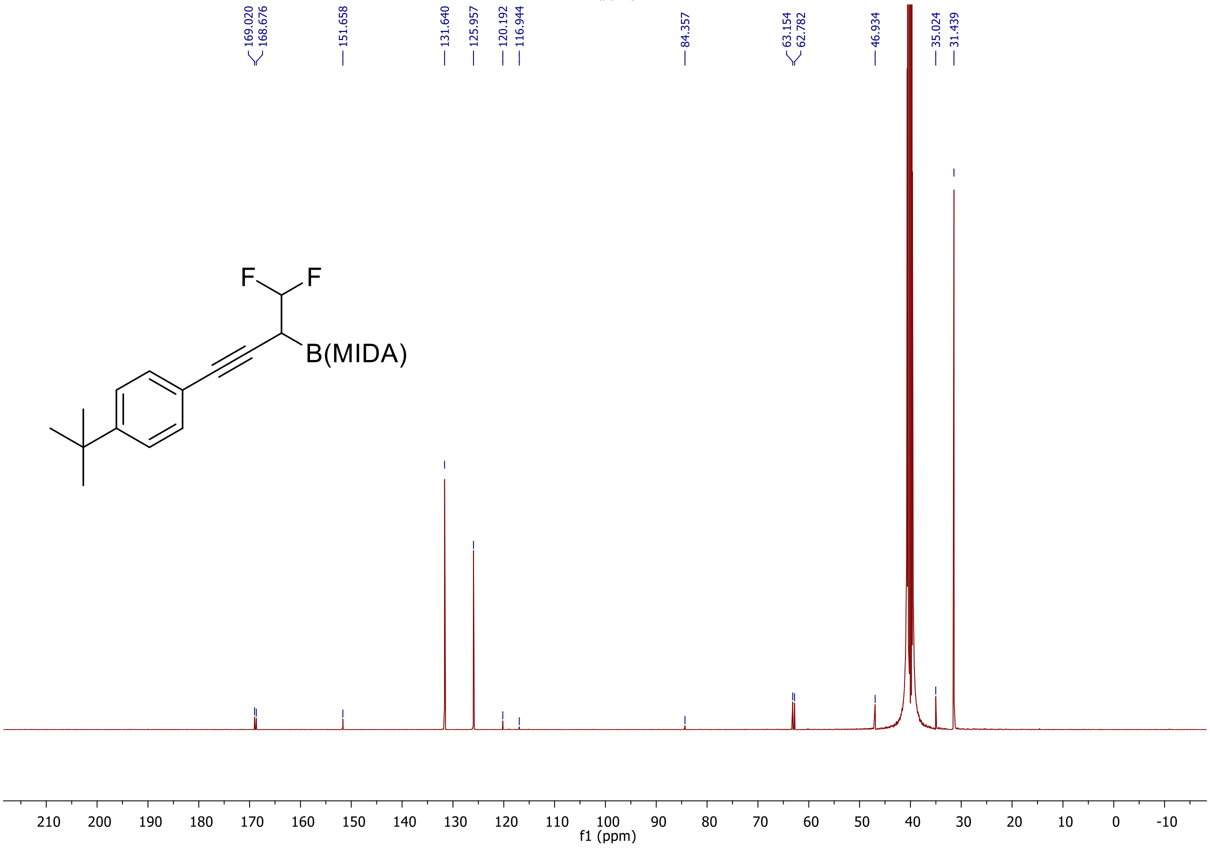
169.020
168.676
151.658
131.640
125.957
120.192
116.944
84.357
63.154
62.782
46.934
35.024
31.439
F
F
B(MIDA)
210 200 190 180 170 160 150 140 130 120 110 100 90 80 70 60 50 40 30 20 10 0 -10
f1 (ppm)

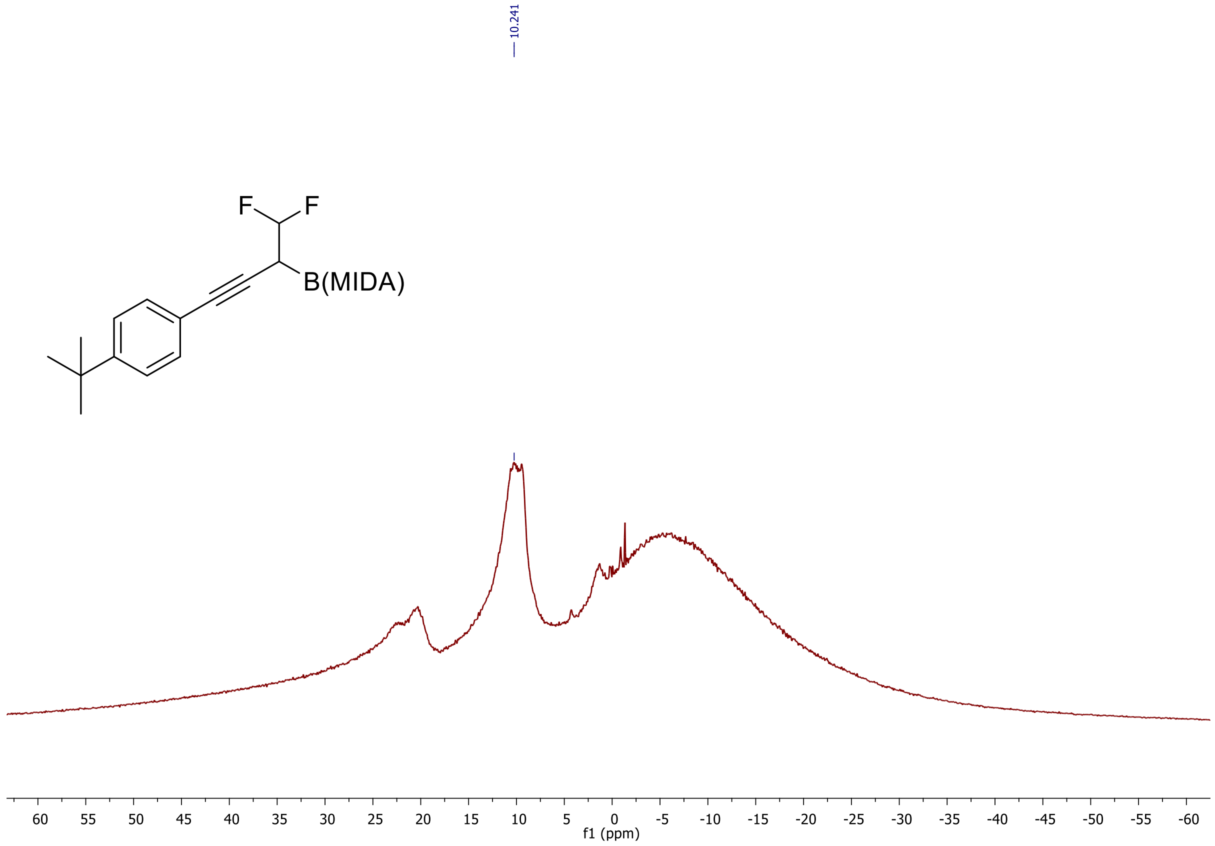
10.241
F
F
B(MIDA)
60 55 50 45 40 35 30 25 20 15 10 5 0 -5 -10 -15 -20 -25 -30 -35 -40 -45 -50 -55 -60
f1 (ppm)

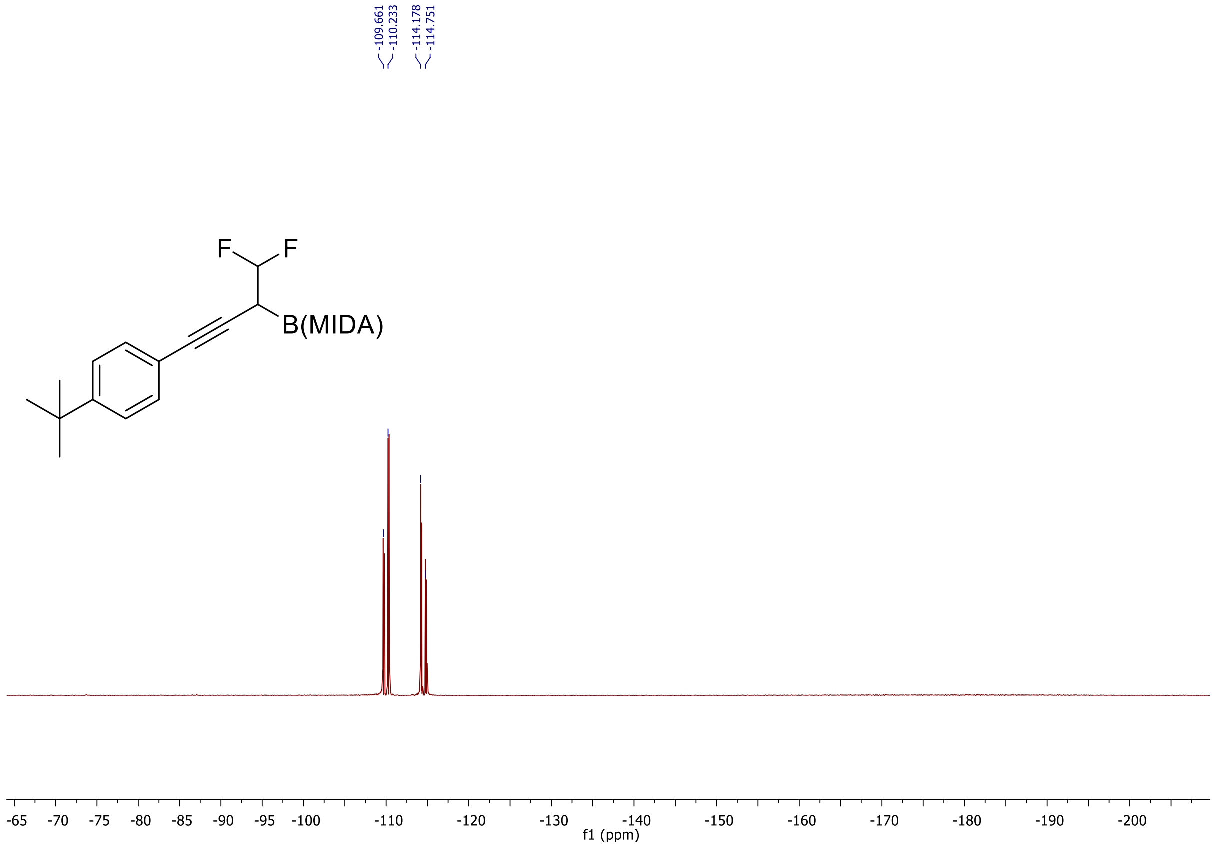
-109.661
-110.233
-114.178
-114.751
F
F
B(MIDA)
-65 -70 -75 -80 -85 -90 -95 -100 -110 -120 -130 -140 -150 -160 -170 -180 -190 -200
f1 (ppm)

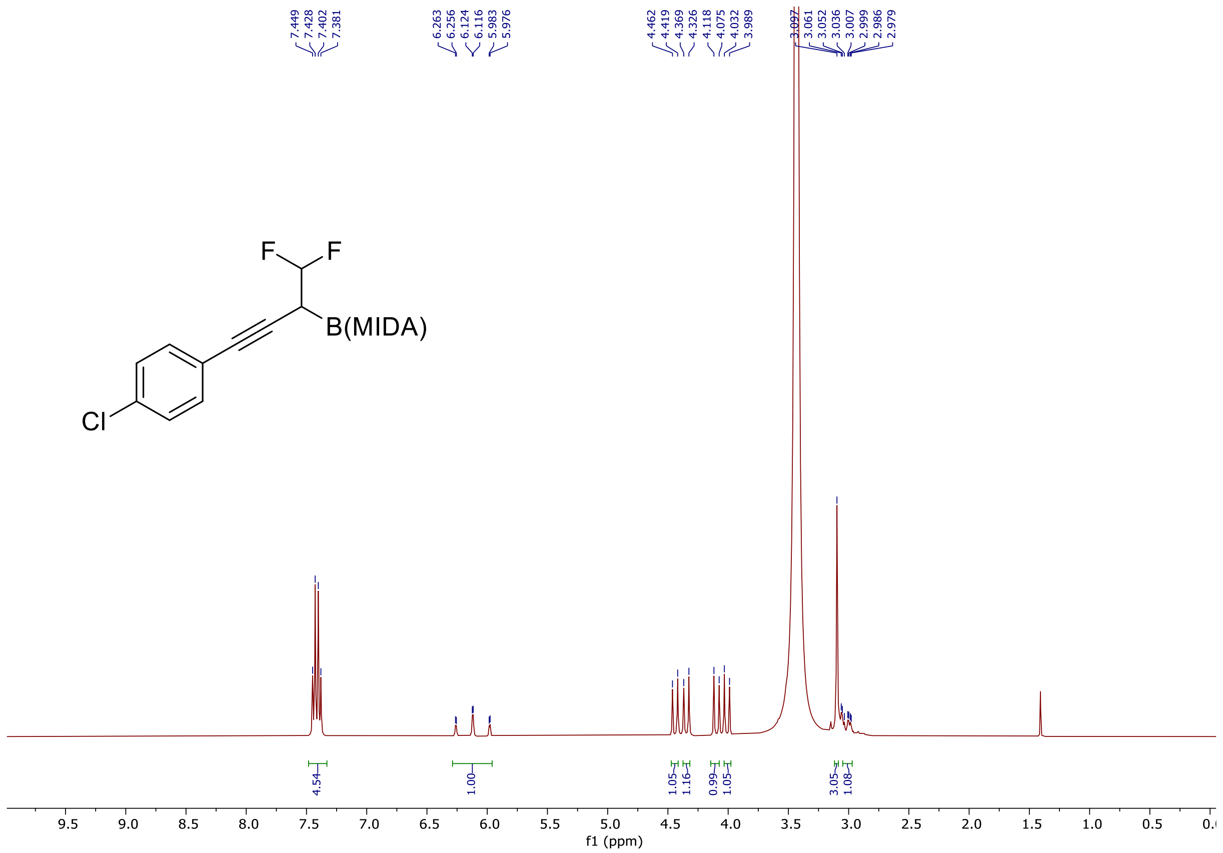
7.449
7.428
7.402
7.381
6.263
6.256
6.124
6.116
5.983
5.976
4.462
4.419
4.369
4.326
4.118
4.075
4.032
3.989
3.097
3.061
3.052
3.036
3.007
2.999
2.986
2.979
F
F
B(MIDA)
Cl
4.54
1.00
1.05
1.16
0.99
1.05
3.05
1.08
f1 (ppm)

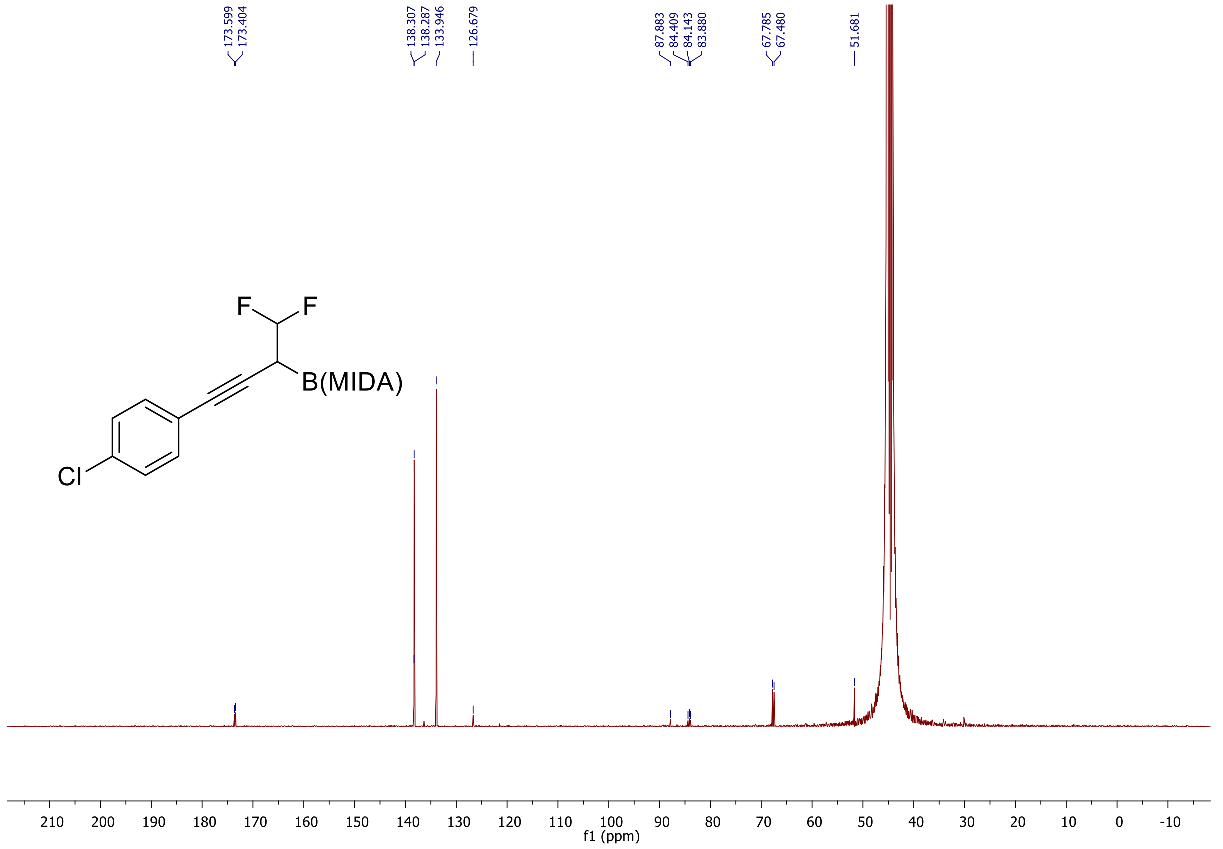
173.599
173.404
138.307
138.287
133.946
126.679
87.883
84.409
84.143
83.880
67.785
67.480
51.681
F
F
B(MIDA)
Cl
f1 (ppm)

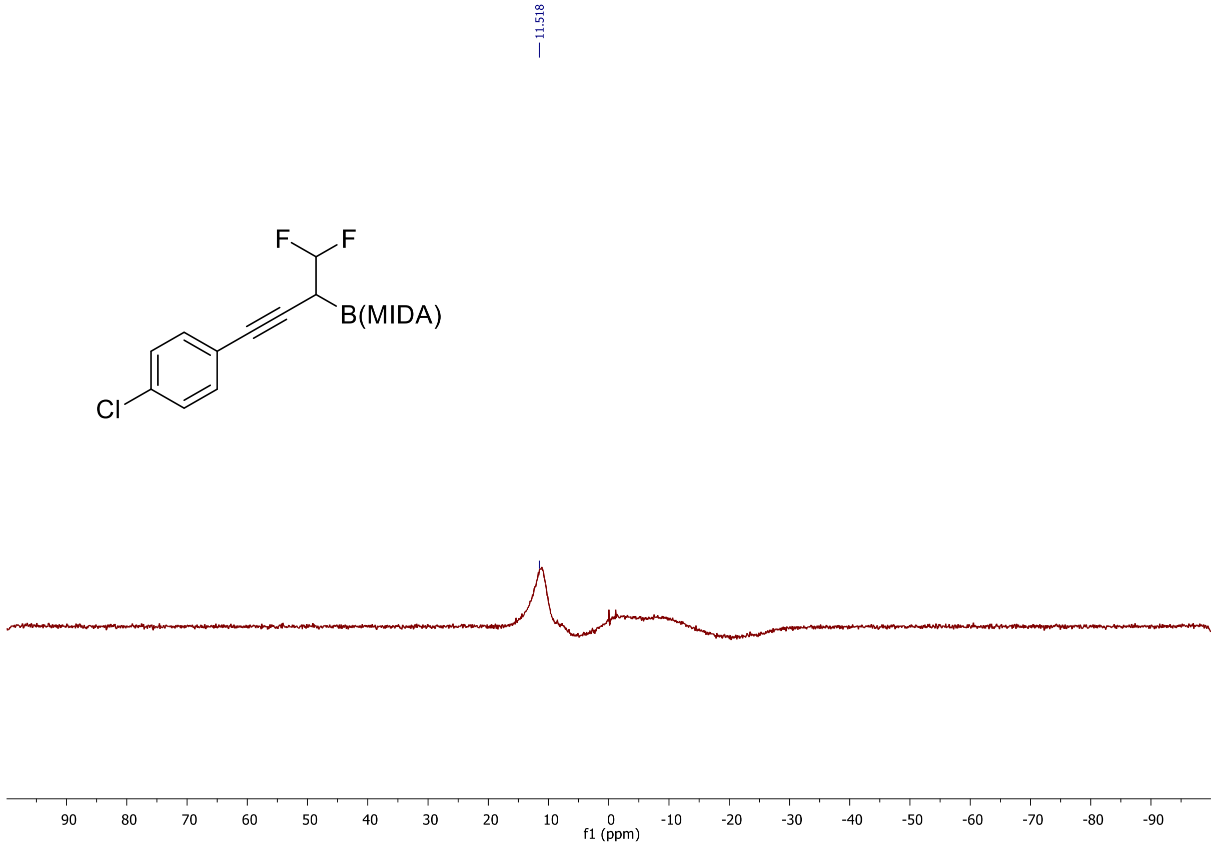
11.518
F
F
B(MIDA)
Cl
90 80 70 60 50 40 30 20 10 0 -10 -20 -30 -40 -50 -60 -70 -80 -90
f1 (ppm)

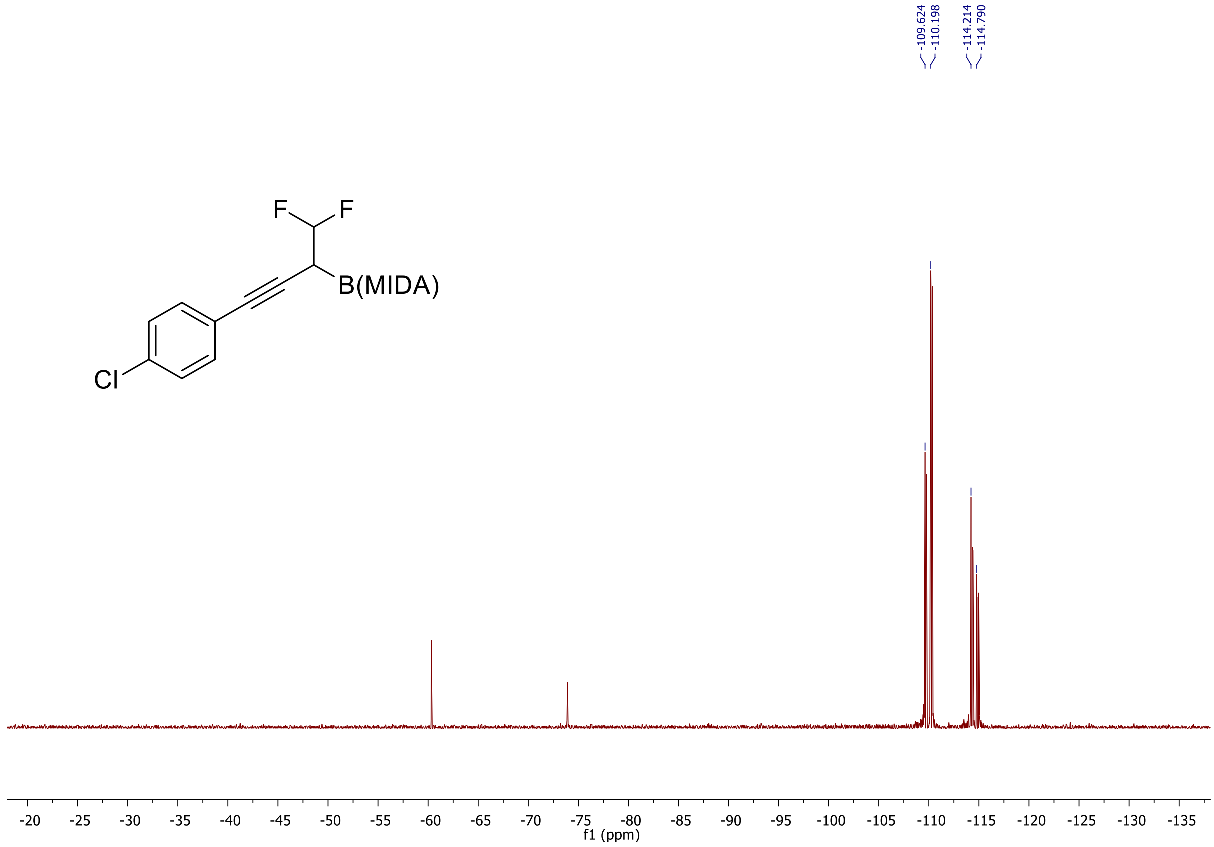
-109.624
-110.198
-114.214
-114.790
F
F
B(MIDA)
Cl
-20 -25 -30 -35 -40 -45 -50 -55 -60 -65 -70 -75 -80 -85 -90 -95 -100 -105 -110 -115 -120 -125 -130 -135
f1 (ppm)

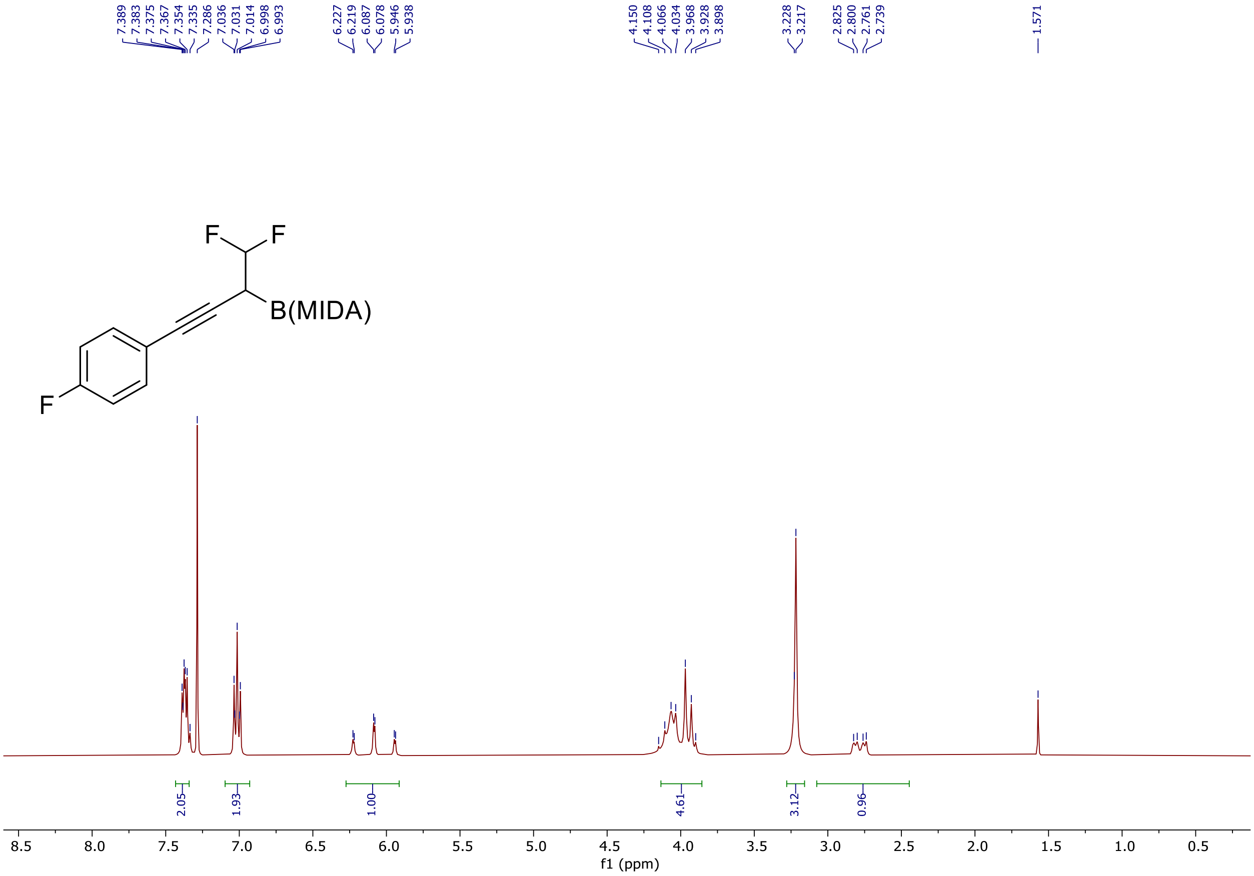
7.389
7.383
7.375
7.367
7.354
7.335
7.286
7.036
7.031
7.014
6.998
6.993
6.227
6.219
6.087
6.078
5.946
5.938
4.150
4.108
4.066
4.034
3.968
3.928
3.898
3.228
3.217
2.825
2.800
2.761
2.739
1.571
F
F
B(MIDA)
F
2.05
1.93
1.00
4.61
3.12
0.96
f1 (ppm)

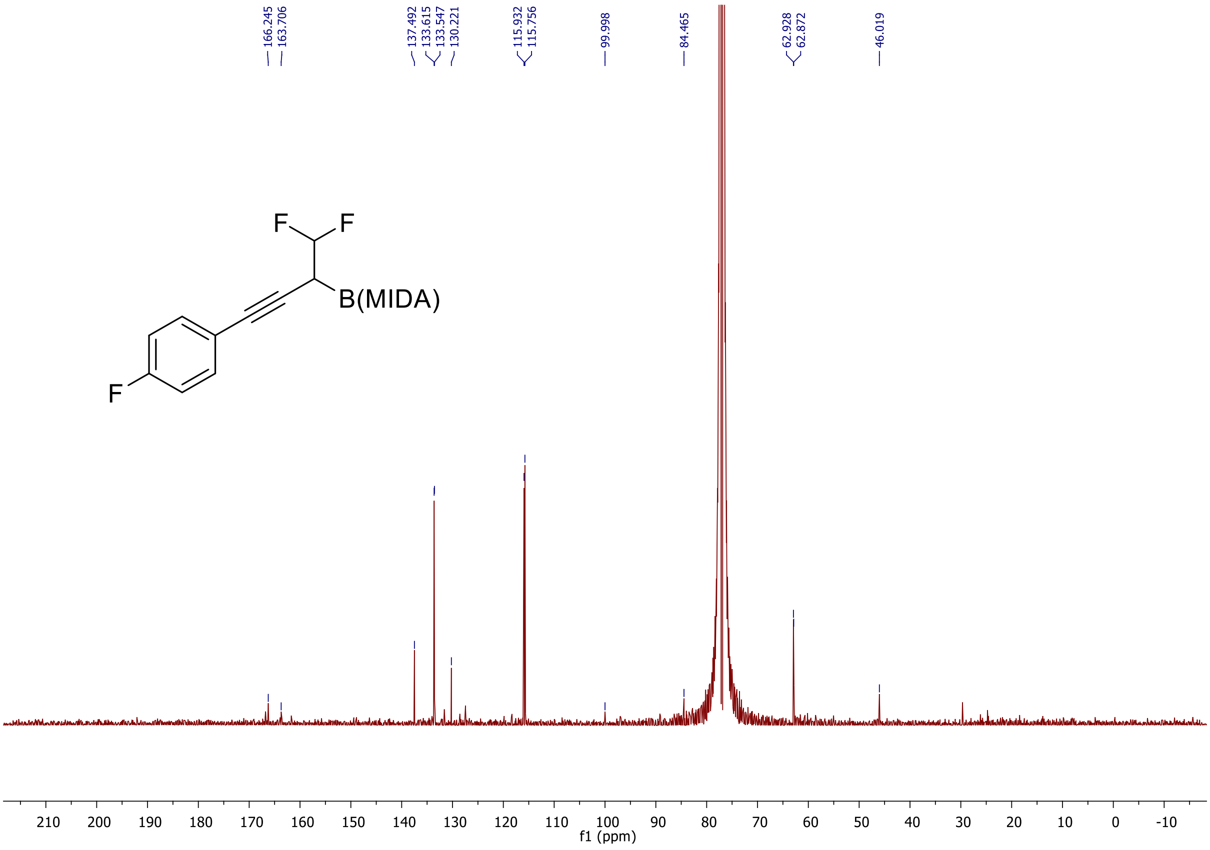
166.245
163.706
137.492
133.615
133.547
130.221
115.932
115.756
99.998
84.465
62.928
62.872
46.019
F
F
B(MIDA)
F
f1 (ppm)

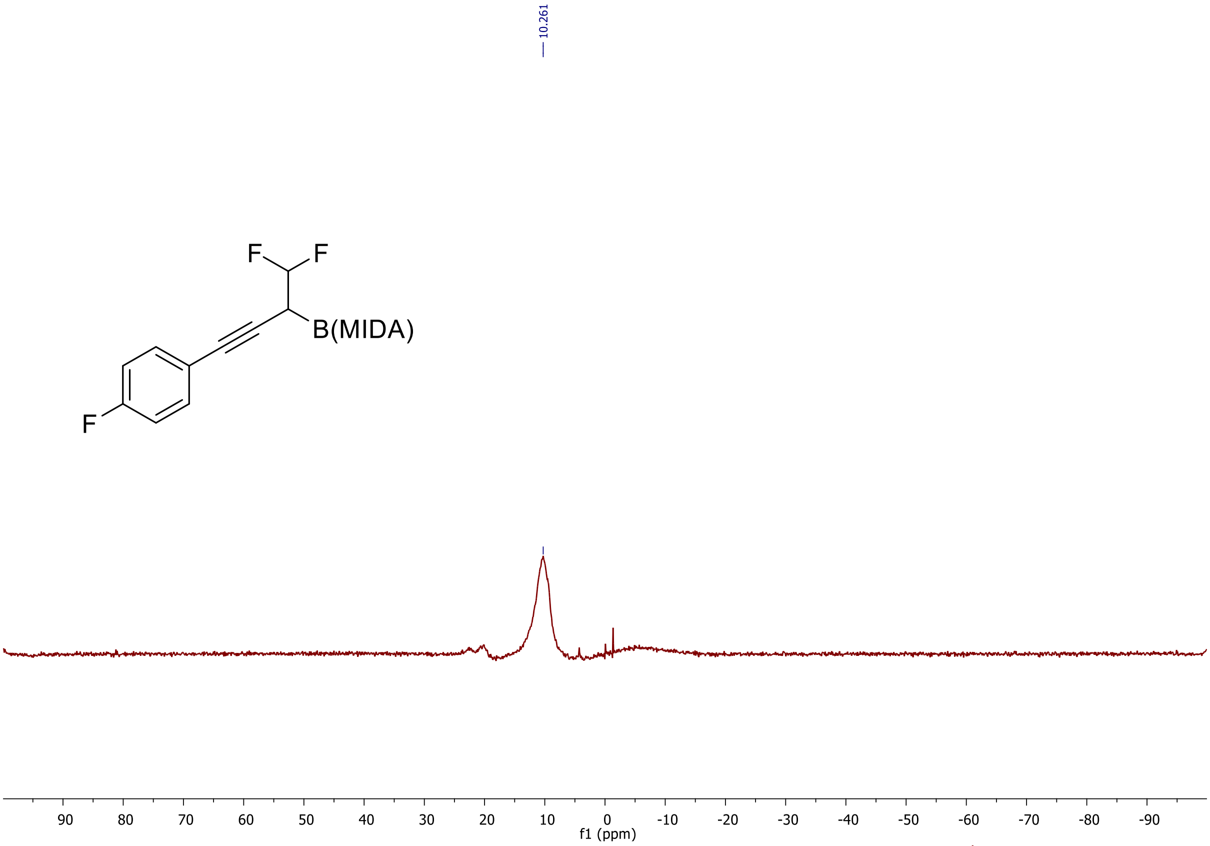
10.261
F
F
B(MIDA)
F
90 80 70 60 50 40 30 20 10 0 -10 -20 -30 -40 -50 -60 -70 -80 -90
f1 (ppm)

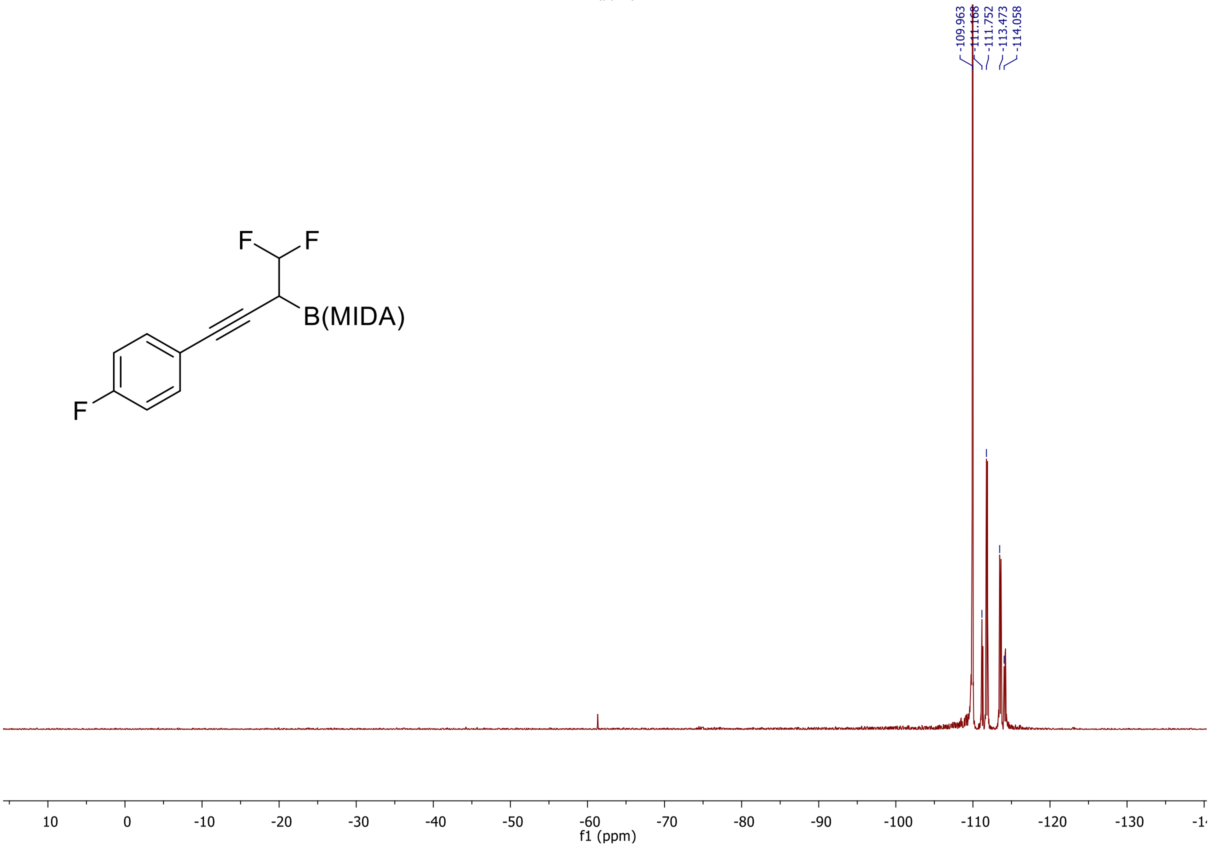
-109.963
-111.168
-111.752
-113.473
-114.058
F
F
B(MIDA)
F
10 0 -10 -20 -30 -40 -50 -60 -70 -80 -90 -100 -110 -120 -130
f1 (ppm)

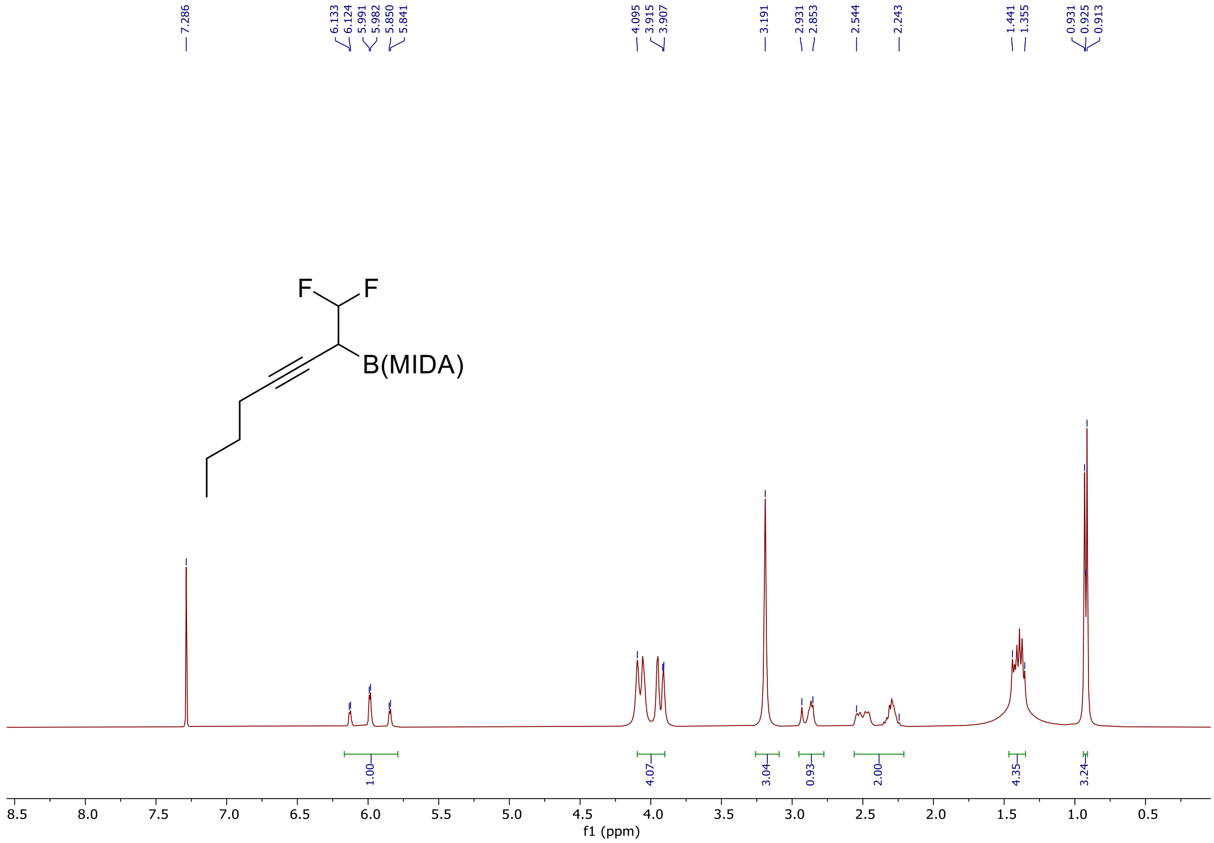
7.286
6.133
6.124
5.991
5.982
5.850
5.841
4.095
3.915
3.907
3.191
2.931
2.853
2.544
2.243
1.441
1.355
0.931
0.925
0.913
F
F
B(MIDA)
1.00
4.07
3.04
0.93
2.00
4.35
3.24
8.5 8.0 7.5 7.0 6.5 6.0 5.5 5.0 4.5 4.0 3.5 3.0 2.5 2.0 1.5 1.0 0.5
f1 (ppm)

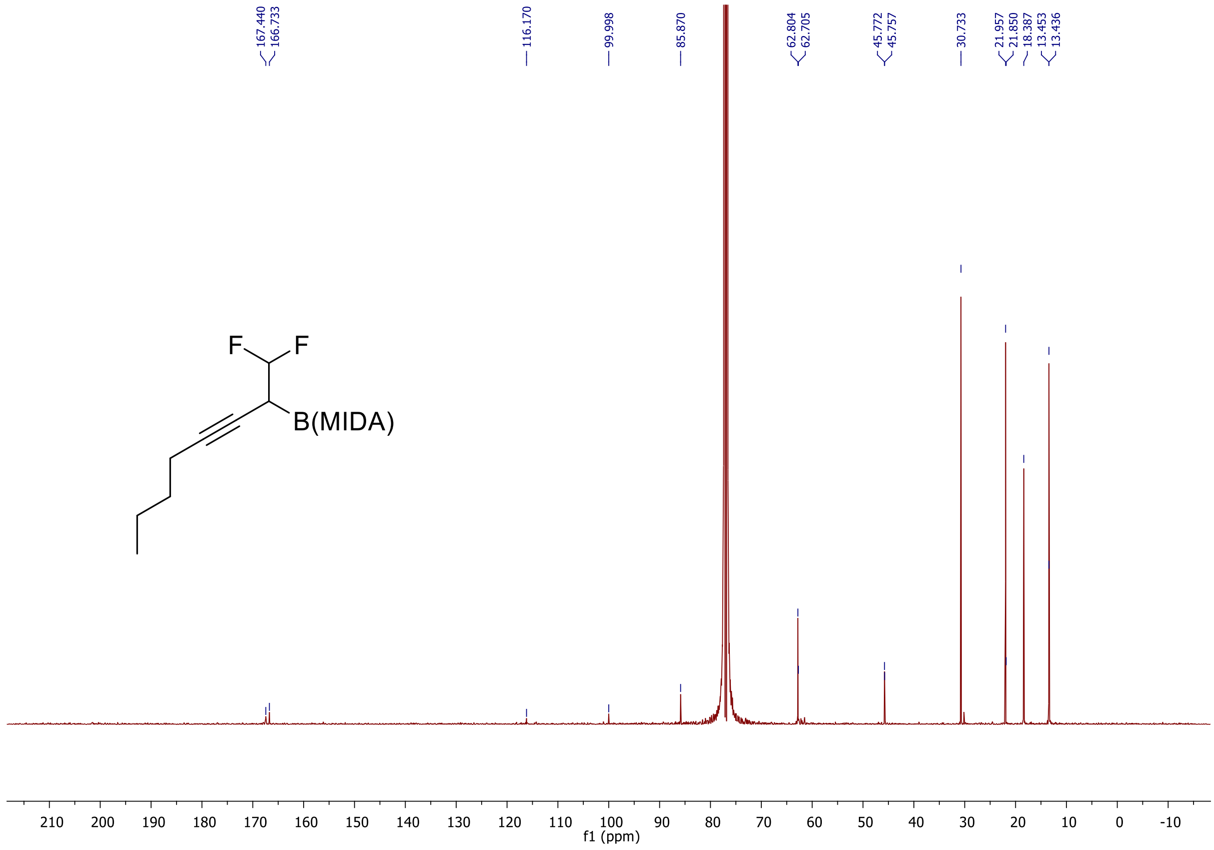
167.440
166.733
116.170
99.998
85.870
62.804
62.705
45.772
45.757
30.733
21.957
21.850
18.387
13.453
13.436
F
F
B(MIDA)
210 200 190 180 170 160 150 140 130 120 110 100 90 80 70 60 50 40 30 20 10 0 -10
f1 (ppm)

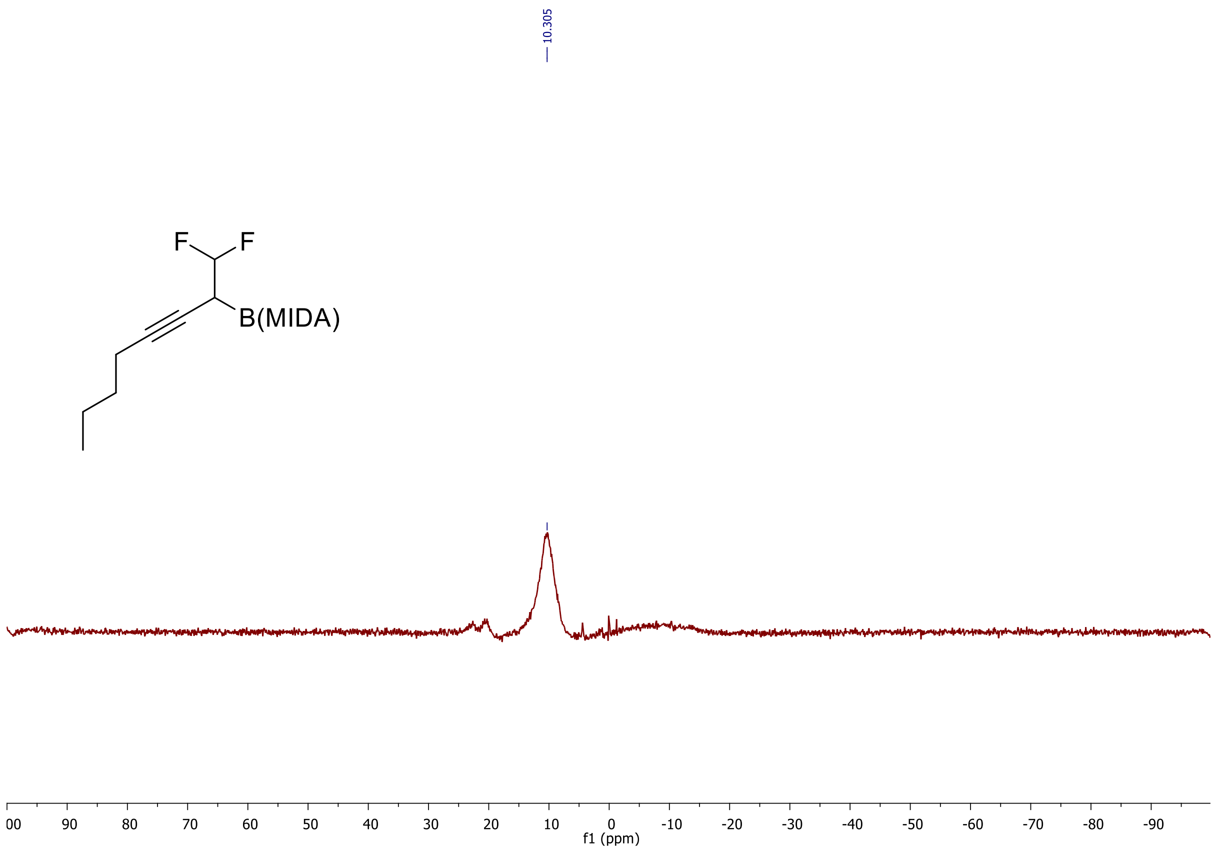
10.305
F
F
B(MIDA)
00
90
80
70
60
50
40
30
20
10
0
-10
-20
-30
-40
-50
-60
-70
-80
-90
f1 (ppm)

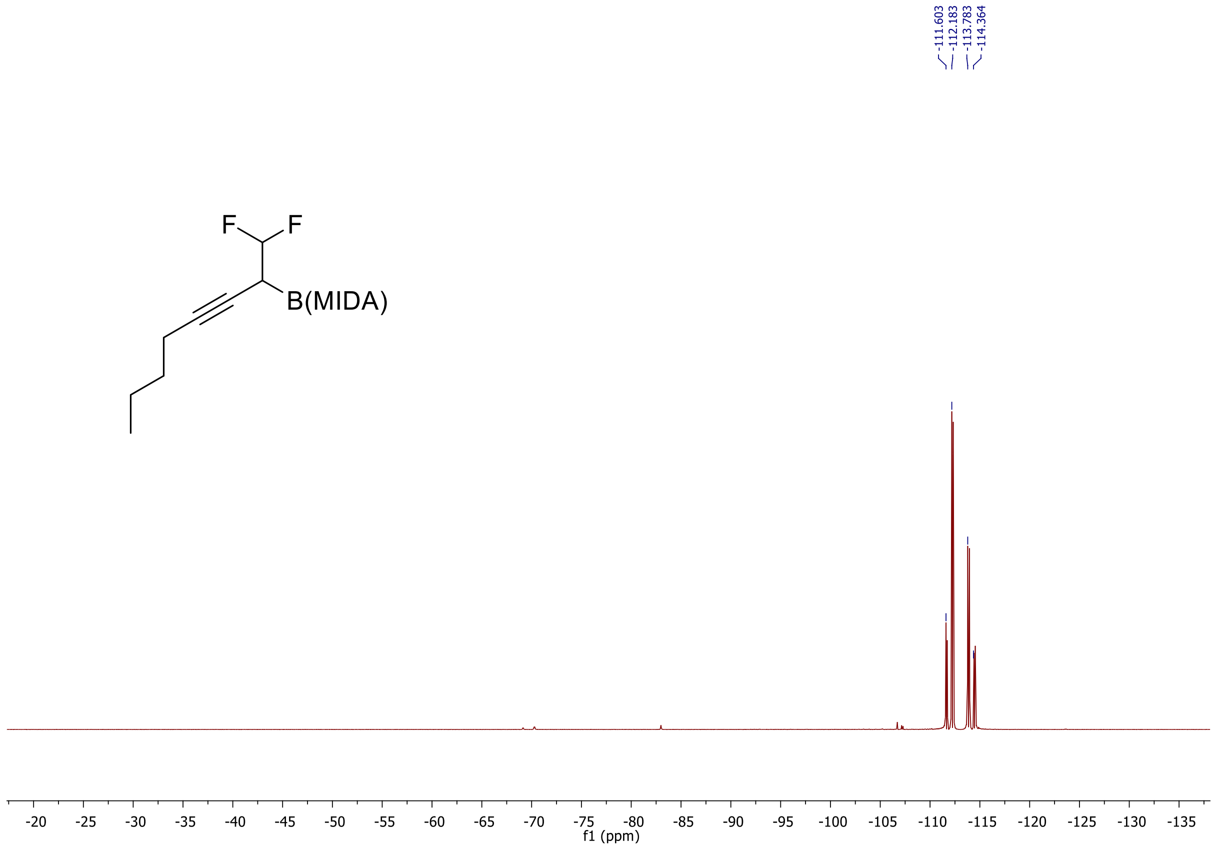
-111.603
-112.183
-113.783
-114.364
F
F
B(MIDA)
-20
-25
-30
-35
-40
-45
-50
-55
-60
-65
-70
-75
-80
-85
-90
-95
-100
-105
-110
-115
-120
-125
-130
-135
f1 (ppm)

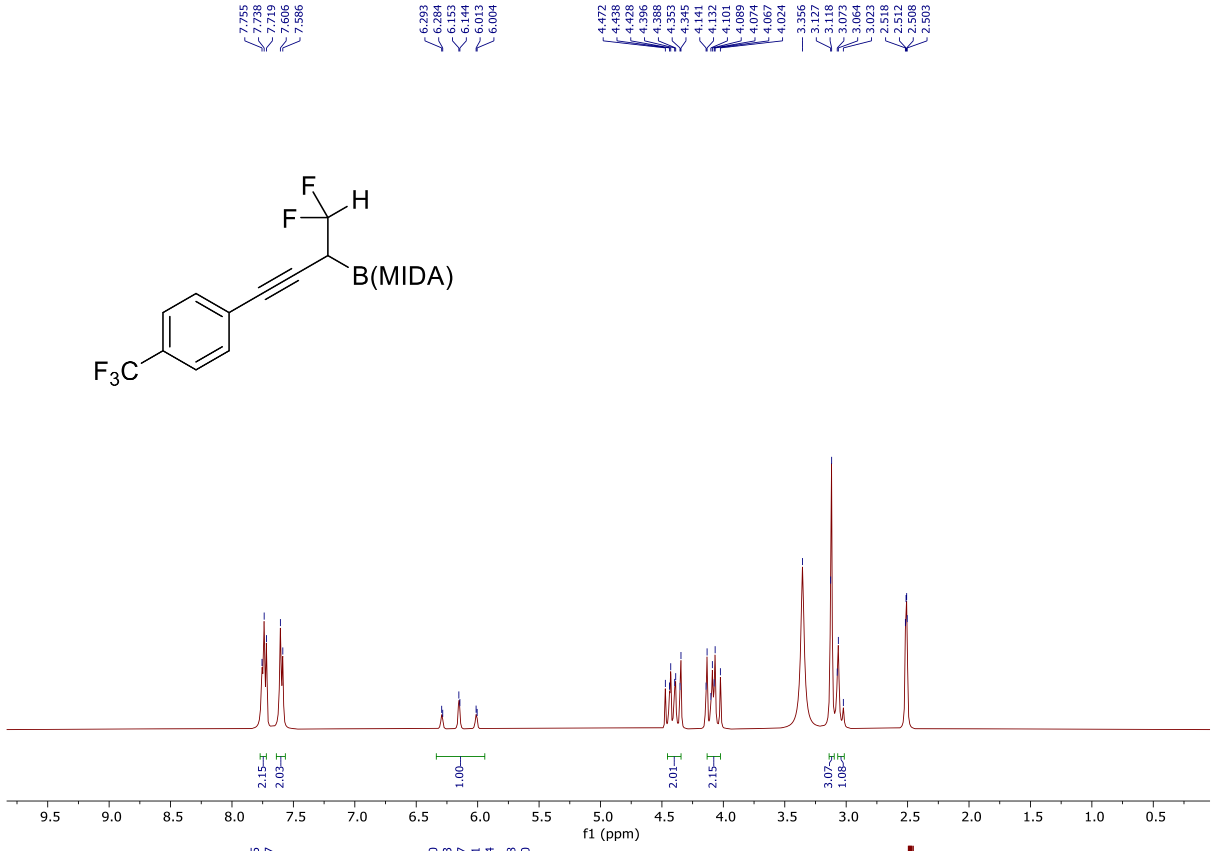
7.755
7.738
7.719
7.606
7.586
6.293
6.284
6.153
6.144
6.013
6.004
4.472
4.438
4.428
4.396
4.388
4.353
4.345
4.141
4.132
4.101
4.089
4.074
4.067
4.024
3.356
3.127
3.118
3.073
3.064
3.023
2.518
2.512
2.508
2.503
F
F
H
B(MIDA)
F3C
2.15
2.03
1.00
2.01
2.15
3.07
1.08
9.5 9.0 8.5 8.0 7.5 7.0 6.5 6.0 5.5 5.0 4.5 4.0 3.5 3.0 2.5 2.0 1.5 1.0 0.5
f1 (ppm)

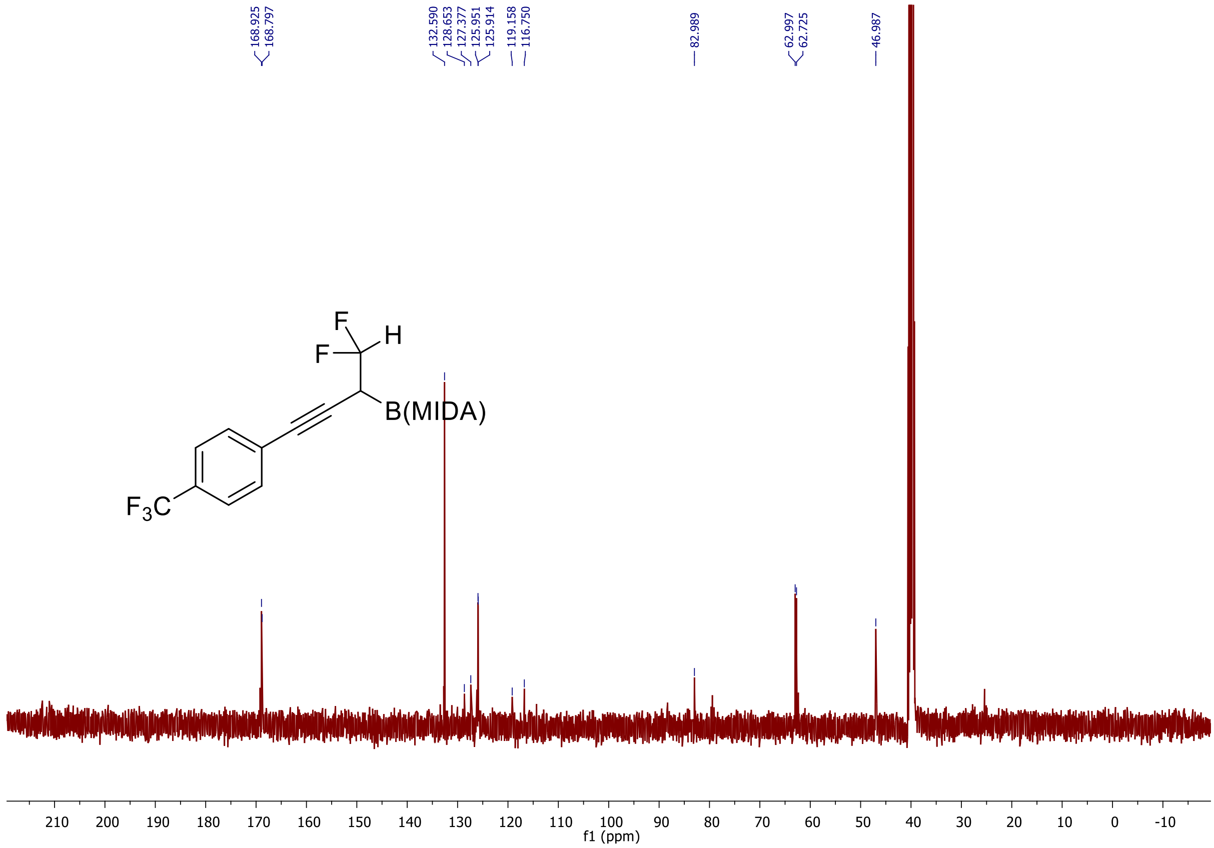
168.925
168.797
132.590
128.653
127.377
125.951
125.914
119.158
116.750
82.989
62.997
62.725
46.987
F
F
H
B(MIDA)
F3C
210 200 190 180 170 160 150 140 130 120 110 100 90 80 70 60 50 40 30 20 10 0 -10
f1 (ppm)

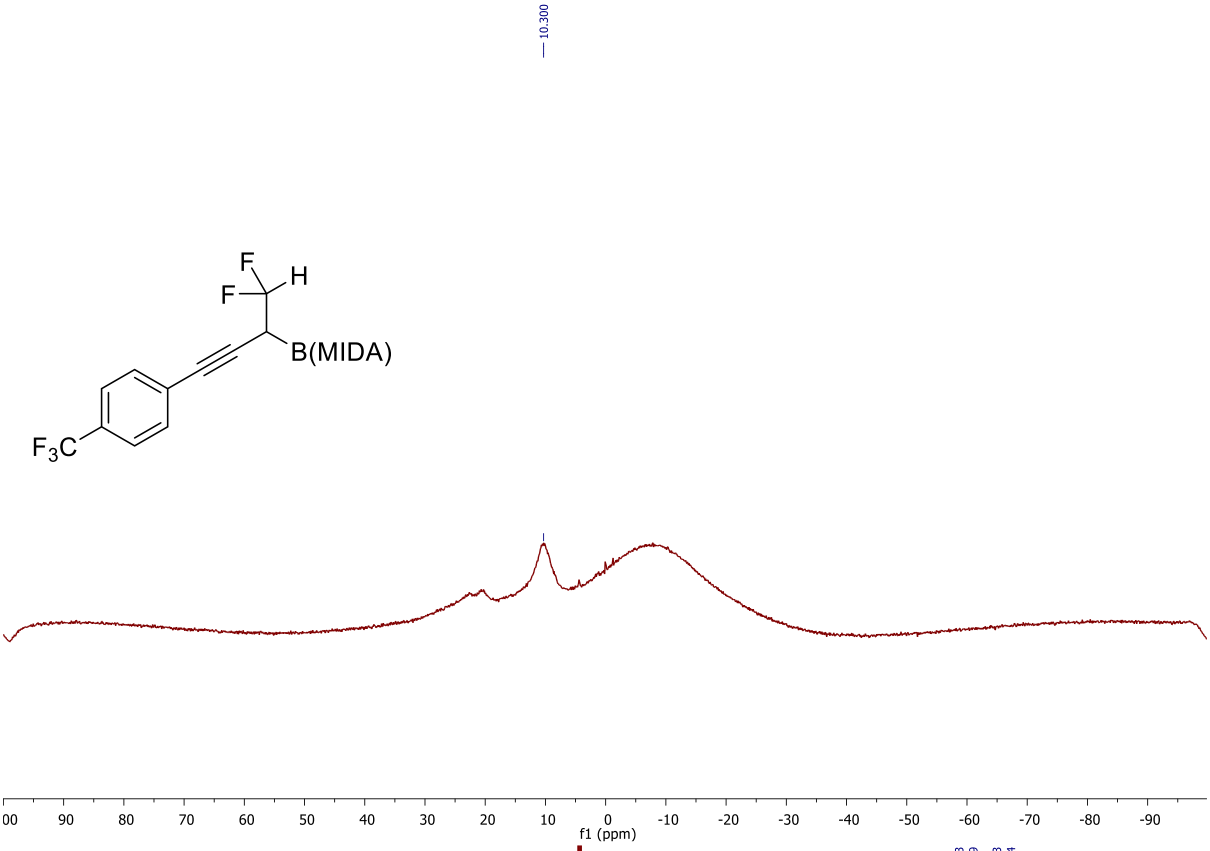
10.300
B(MIDA)
00 90 80 70 60 50 40 30 20 10 0 -10 -20 -30 -40 -50 -60 -70 -80 -90
f1 (ppm)

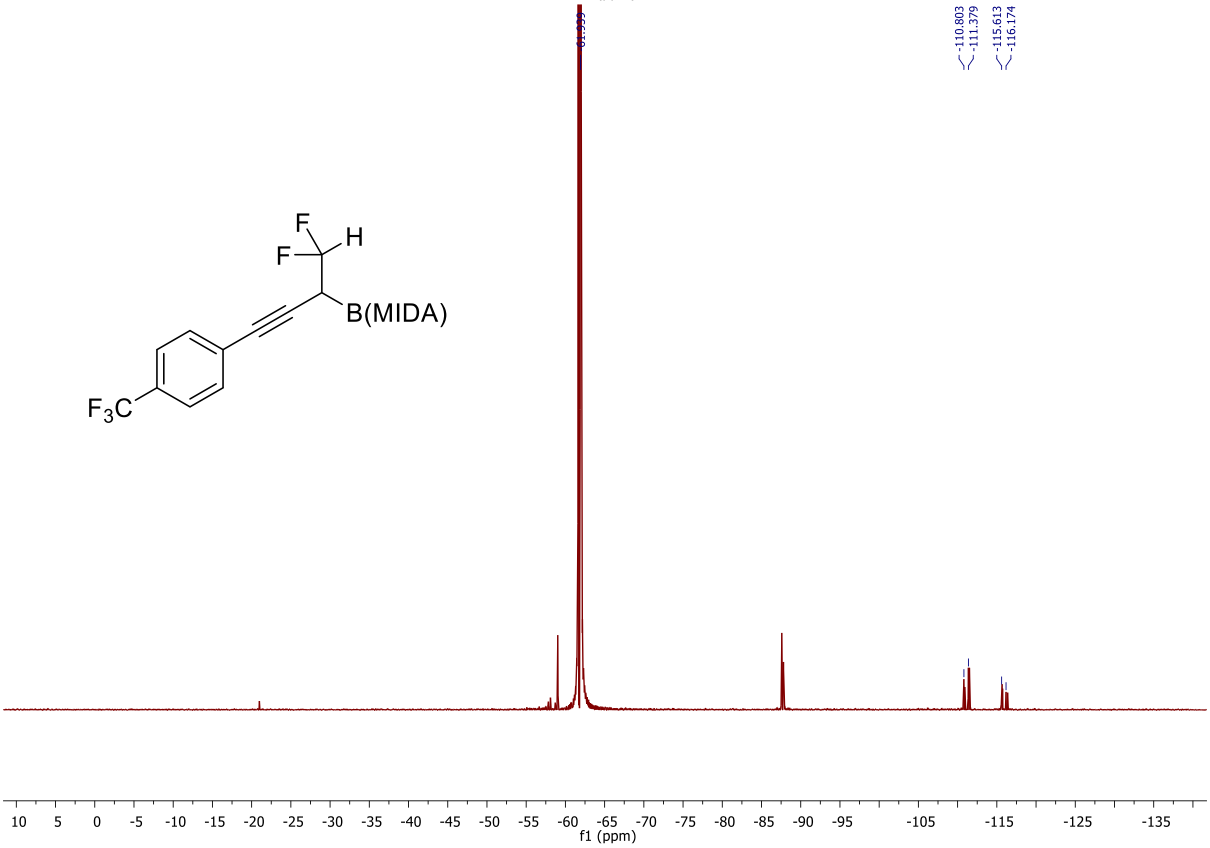
-110.803
-111.379
-115.613
-116.174
B(MIDA)
10 5 0 -5 -10 -15 -20 -25 -30 -35 -40 -45 -50 -55 -60 -65 -70 -75 -80 -85 -90 -95 -105 -115 -125 -135
f1 (ppm)

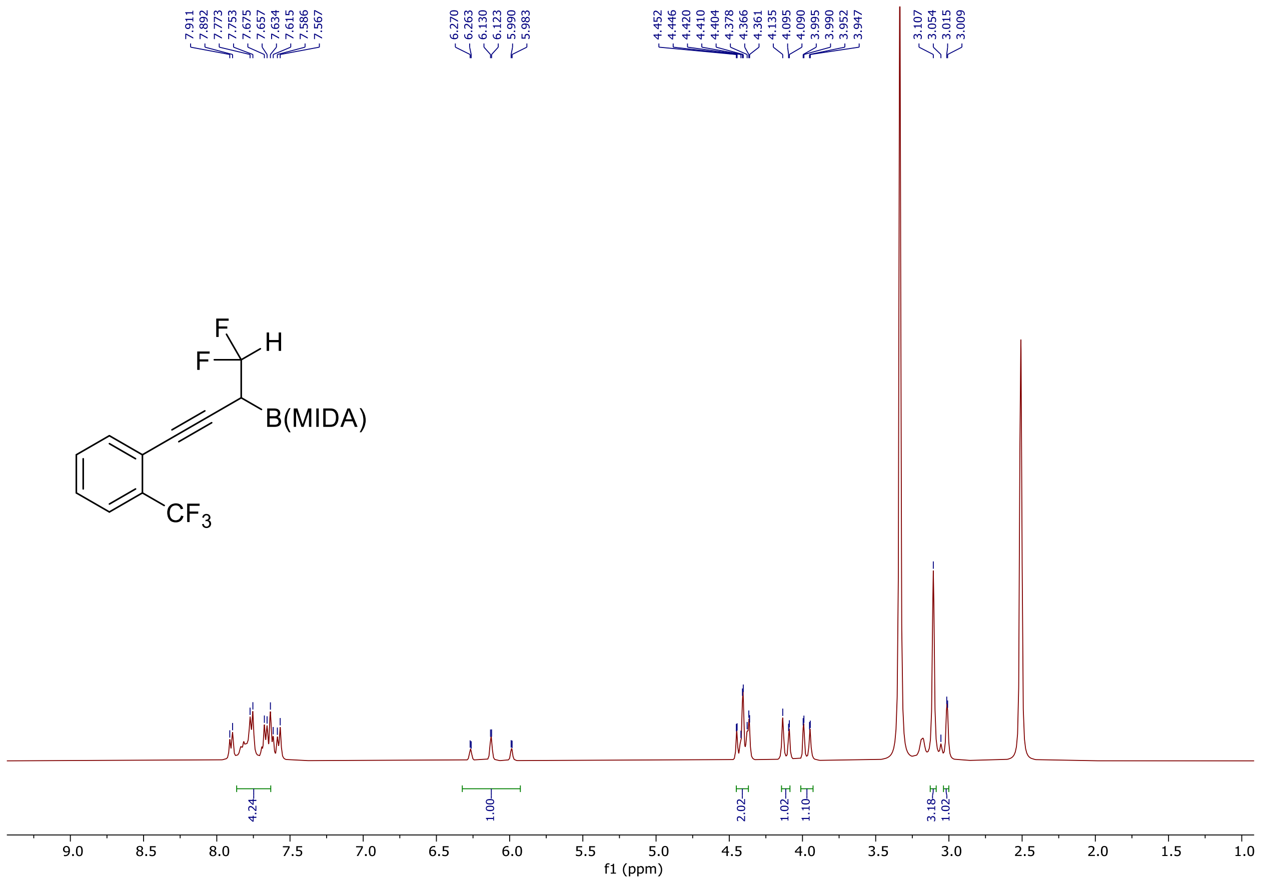
7.911
7.892
7.773
7.753
7.675
7.657
7.634
7.615
7.586
7.567
6.270
6.263
6.130
6.123
5.990
5.983
4.452
4.446
4.420
4.410
4.404
4.378
4.366
4.361
4.135
4.095
4.090
3.995
3.990
3.952
3.947
3.107
3.054
3.015
3.009
F
H
F
B(MIDA)
CF3
4.24
1.00
2.02
1.02
1.10
3.18
1.02
f1 (ppm)

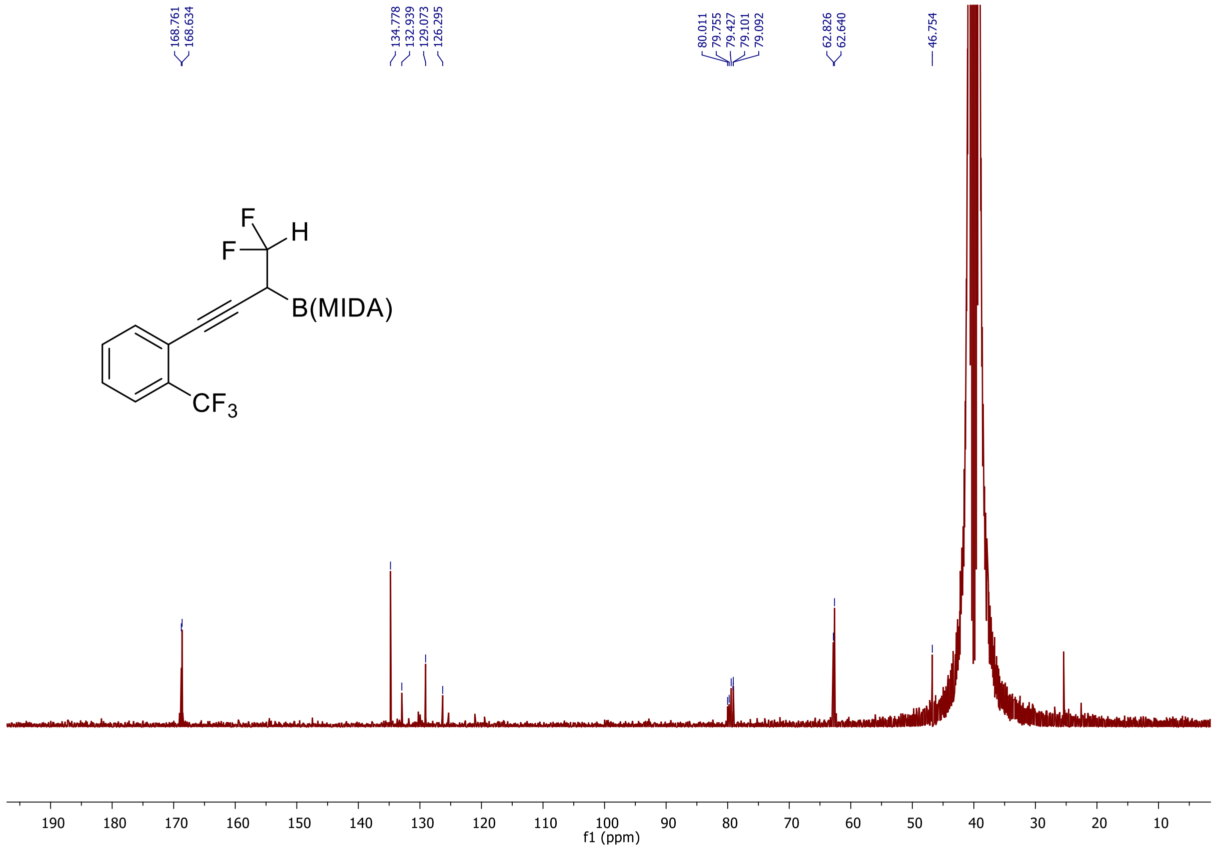
168.761
168.634
134.778
132.939
129.073
126.295
80.011
79.755
79.427
79.101
79.092
62.826
62.640
46.754
F
H
F
B(MIDA)
CF3
f1 (ppm)

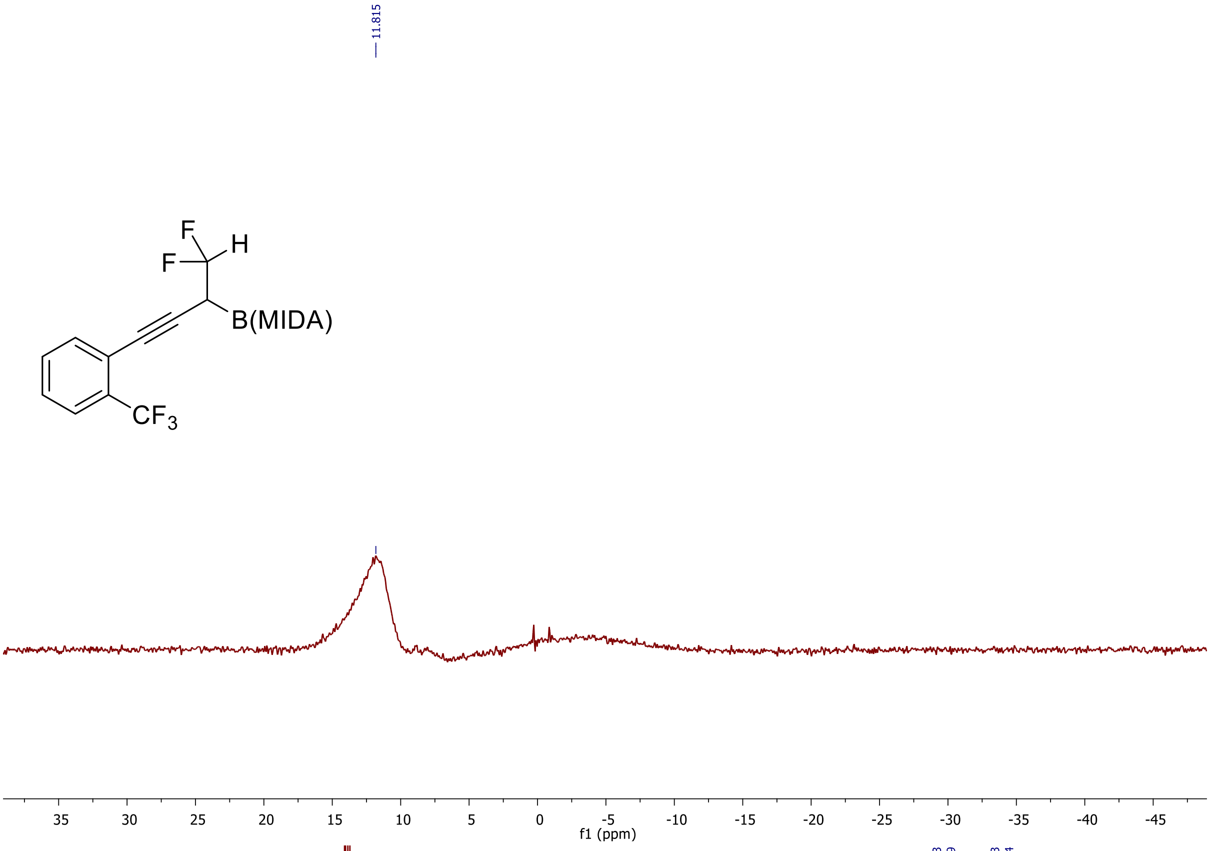

11.815
F
F
H
B(MIDA)
CF3
35 30 25 20 15 10 5 0 -5 -10 -15 -20 -25 -30 -35 -40 -45
f1 (ppm)

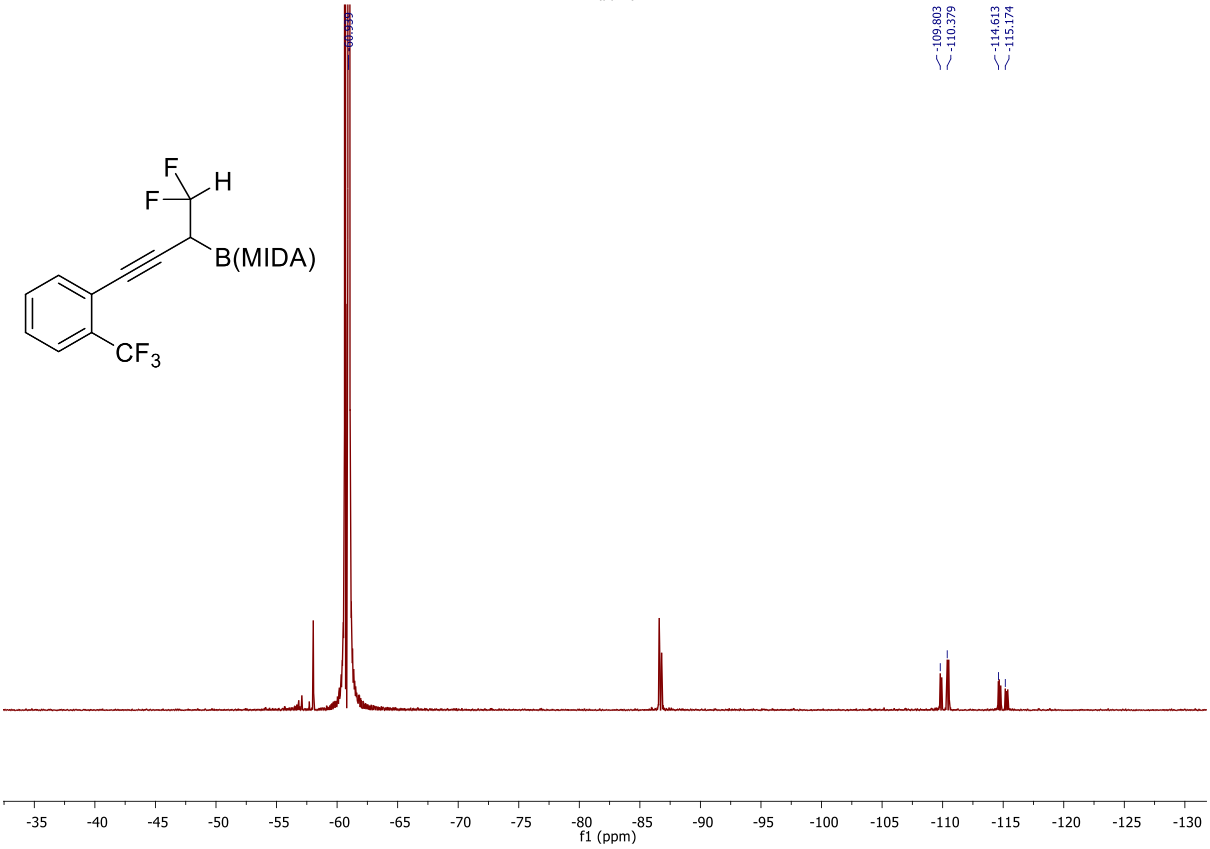

-60.939
-109.803
-110.379
-114.613
-115.174
F
F
H
B(MIDA)
CF3
-35 -40 -45 -50 -55 -60 -65 -70 -75 -80 -85 -90 -95 -100 -105 -110 -115 -120 -125 -130
f1 (ppm)

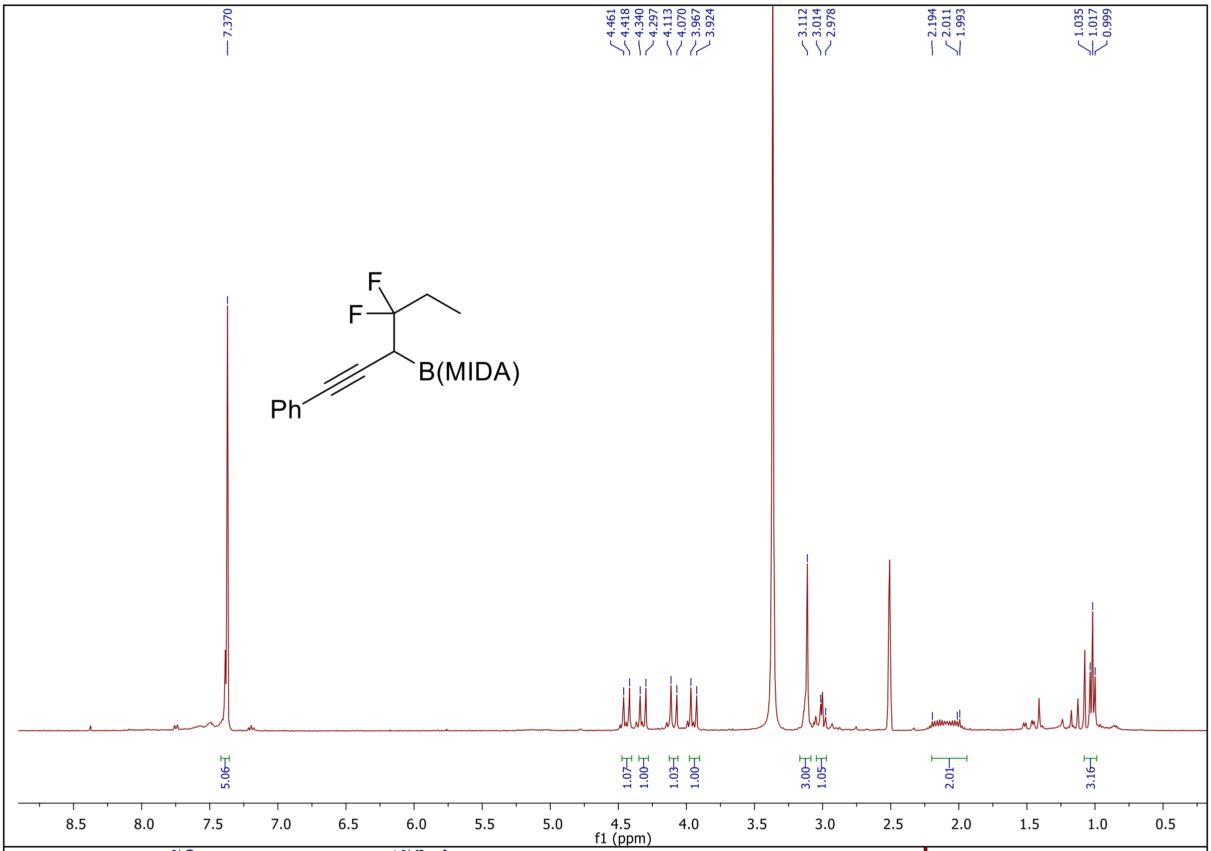
7.370
4.461
4.418
4.340
4.297
4.113
4.070
3.967
3.924
3.112
3.014
2.978
2.194
2.011
1.993
1.035
1.017
0.999
F
F
B(MIDA)
Ph
5.06
1.07
1.00
1.03
1.00
3.00
1.05
2.01
3.16
8.5
8.0
7.5
7.0
6.5
6.0
5.5
5.0
4.5
4.0
3.5
3.0
2.5
2.0
1.5
1.0
0.5
f1 (ppm)

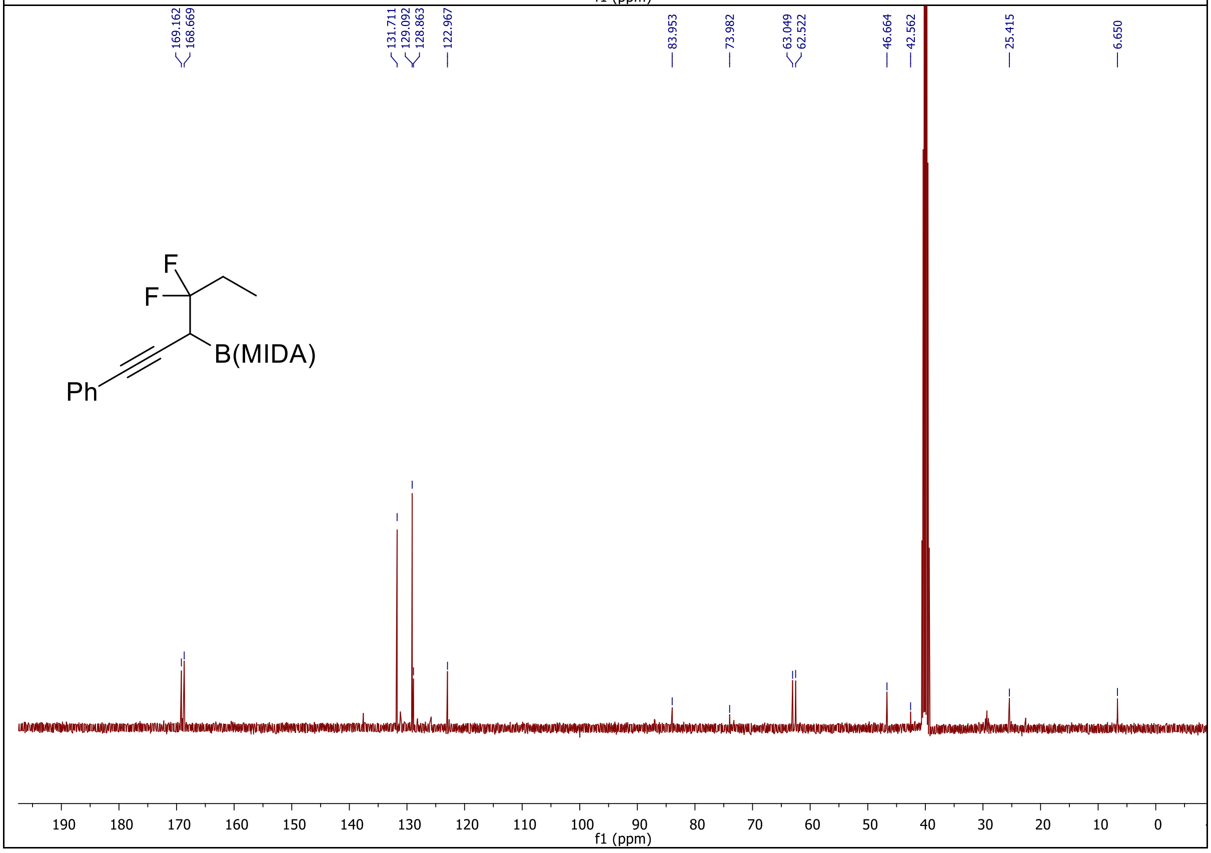
169.162
168.669
131.711
129.092
128.863
122.967
83.953
73.982
63.049
62.522
46.664
42.562
25.415
6.650
F
F
B(MIDA)
Ph
190
180
170
160
150
140
130
120
110
100
90
80
70
60
50
40
30
20
10
0
f1 (ppm)

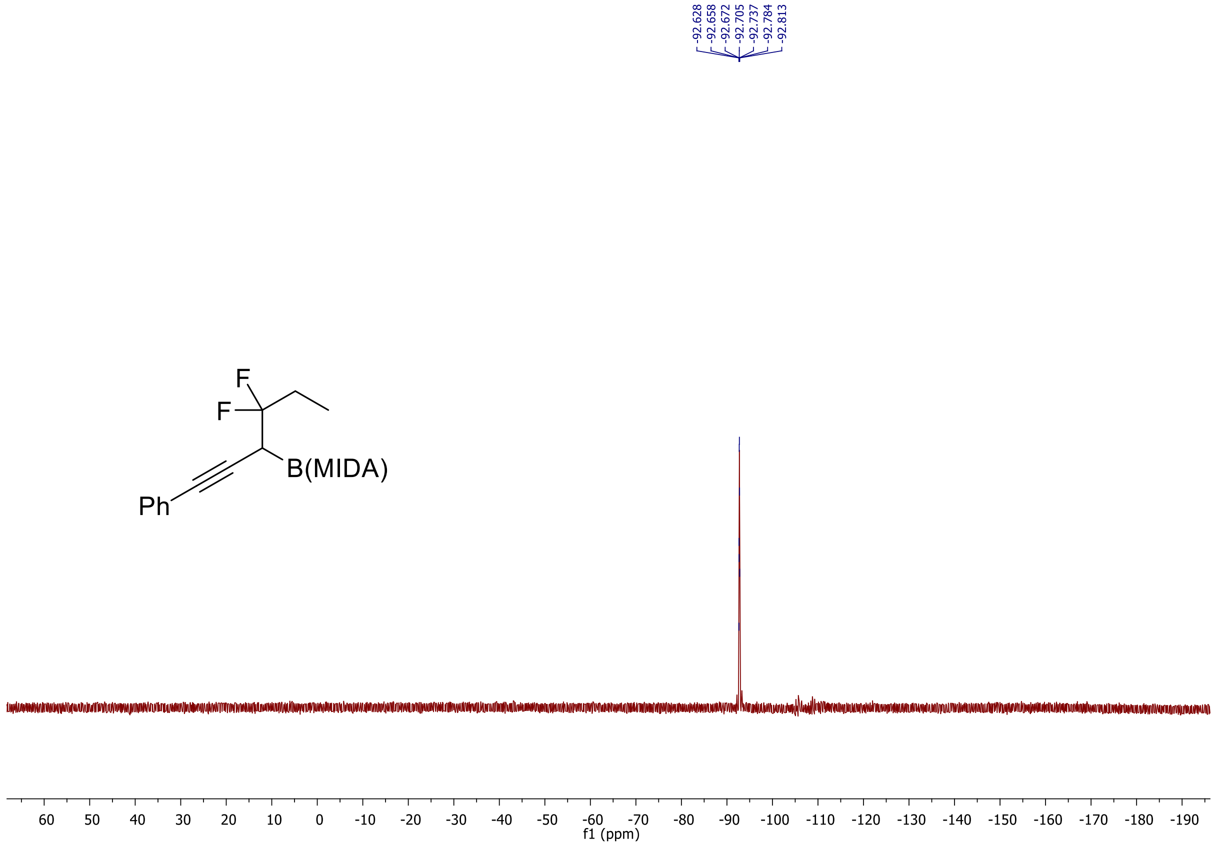
-92.628
-92.658
-92.672
-92.705
-92.737
-92.784
-92.813
F
F
B(MIDA)
Ph
f1 (ppm)

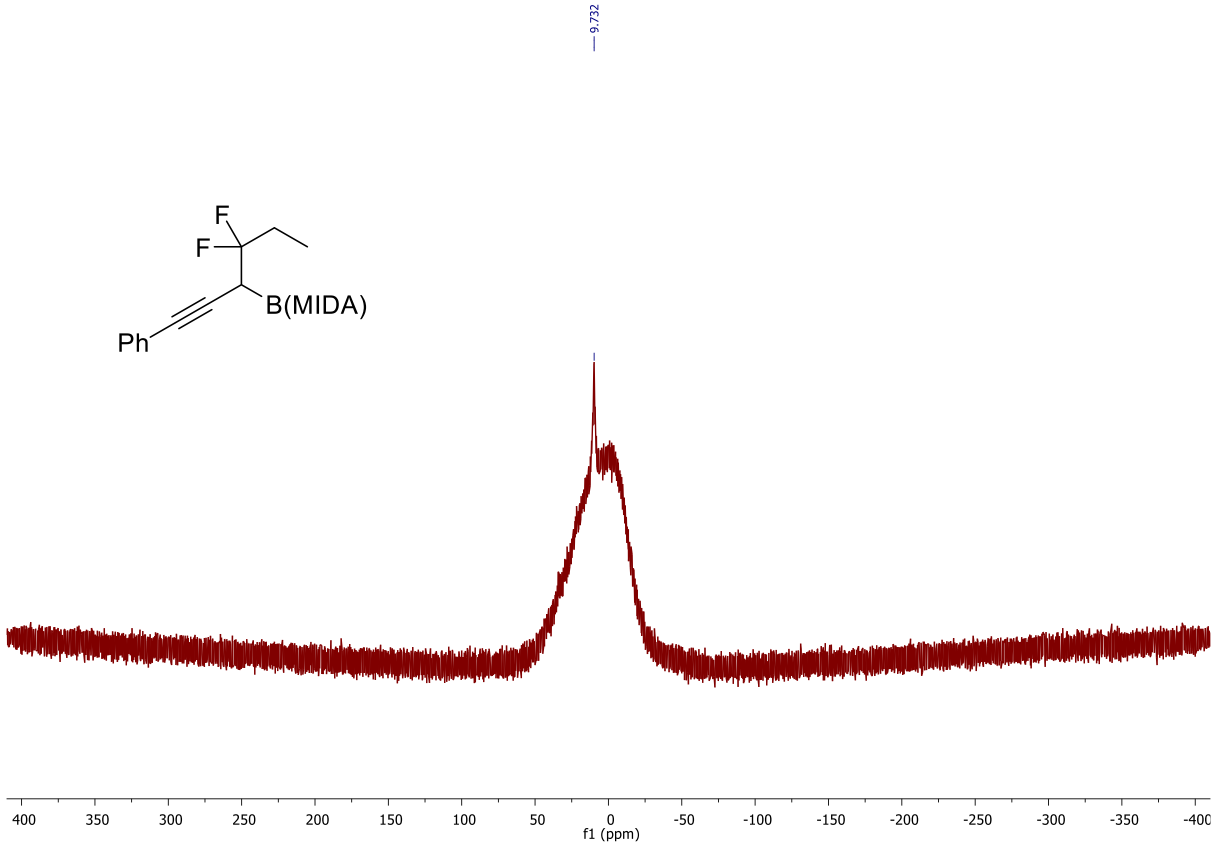
9.732
F
F
B(MIDA)
Ph
f1 (ppm)

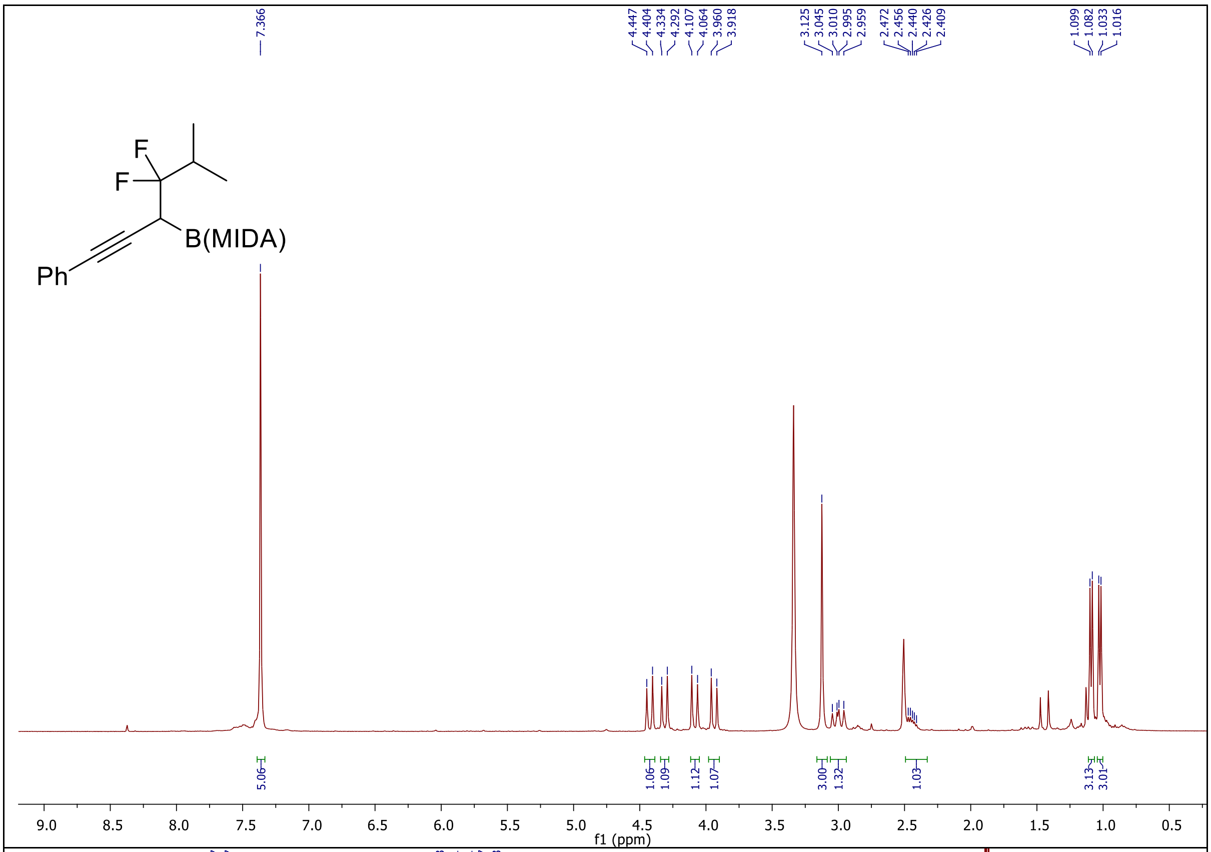
7.366
4.447
4.404
4.334
4.292
4.107
4.064
3.960
3.918
3.125
3.045
3.010
2.995
2.959
2.472
2.456
2.440
2.426
2.409
1.099
1.082
1.033
1.016
F
F
B(MIDA)
Ph
5.06
1.06
1.09
1.12
1.07
3.00
1.32
1.03
3.13
3.01
9.0 8.5 8.0 7.5 7.0 6.5 6.0 5.5 5.0 4.5 4.0 3.5 3.0 2.5 2.0 1.5 1.0 0.5
f1 (ppm)

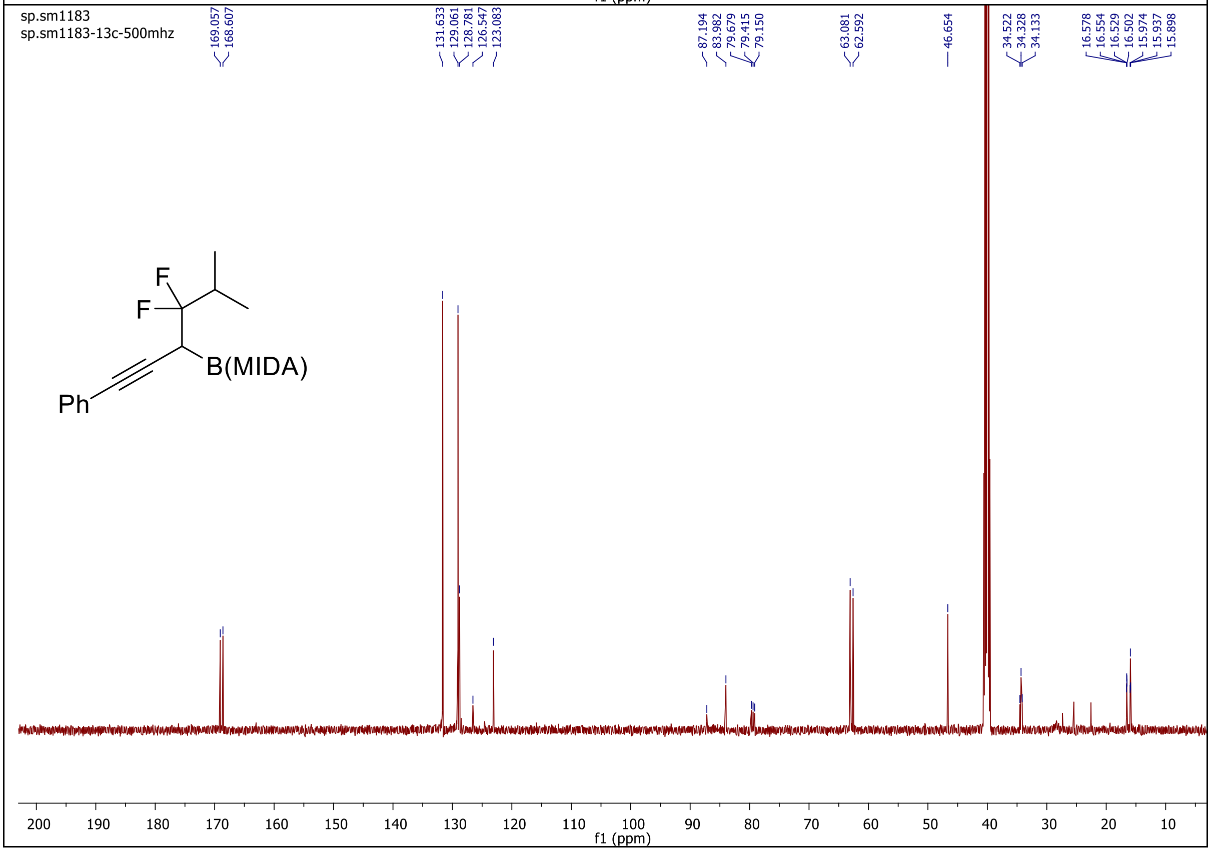
sp.sm1183
sp.sm1183-13c-500mhz
169.057
168.607
131.633
129.061
128.781
126.547
123.083
87.194
83.982
79.679
79.415
79.150
63.081
62.592
46.654
34.522
34.328
34.133
16.578
16.554
16.529
16.502
15.974
15.937
15.898
F
F
B(MIDA)
Ph
200 190 180 170 160 150 140 130 120 110 100 90 80 70 60 50 40 30 20 10
f1 (ppm)

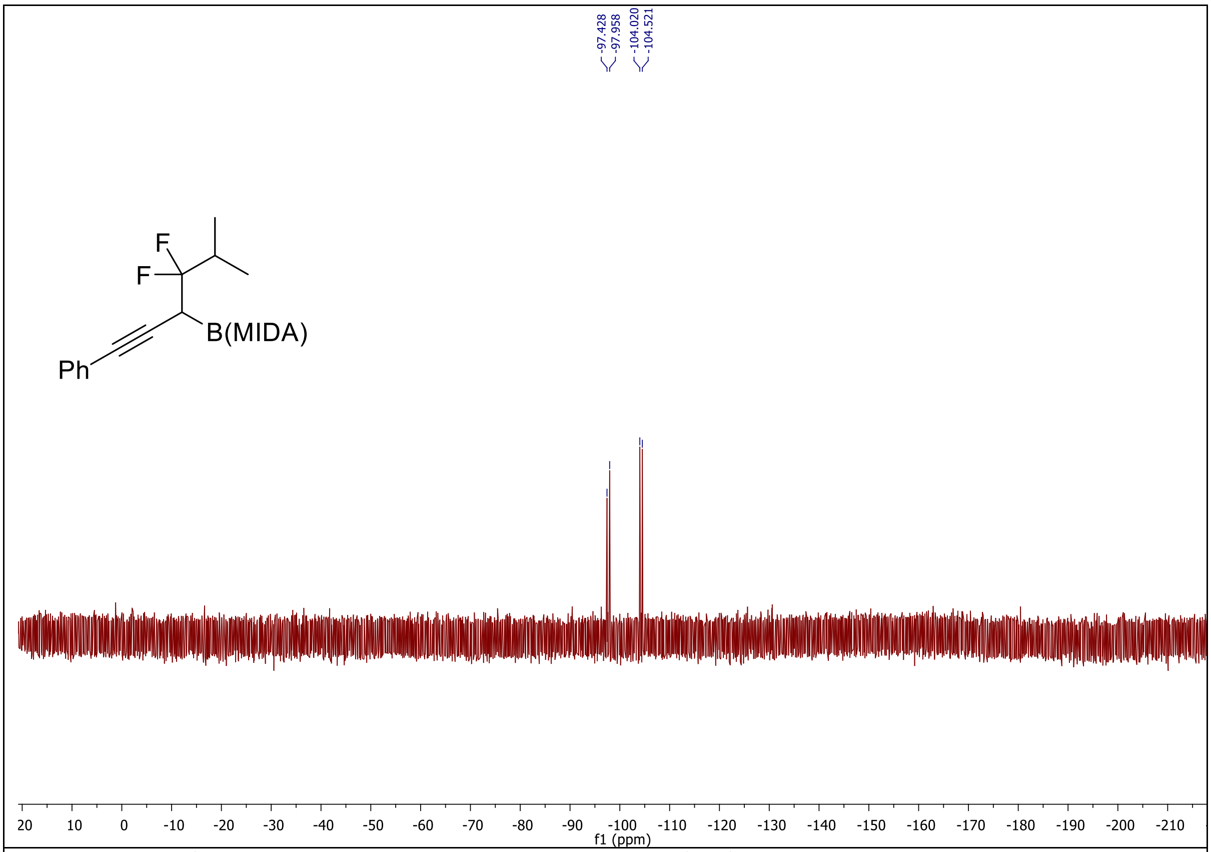
-97.428
-97.958
-104.020
-104.521
F
F
B(MIDA)
Ph
f1 (ppm)

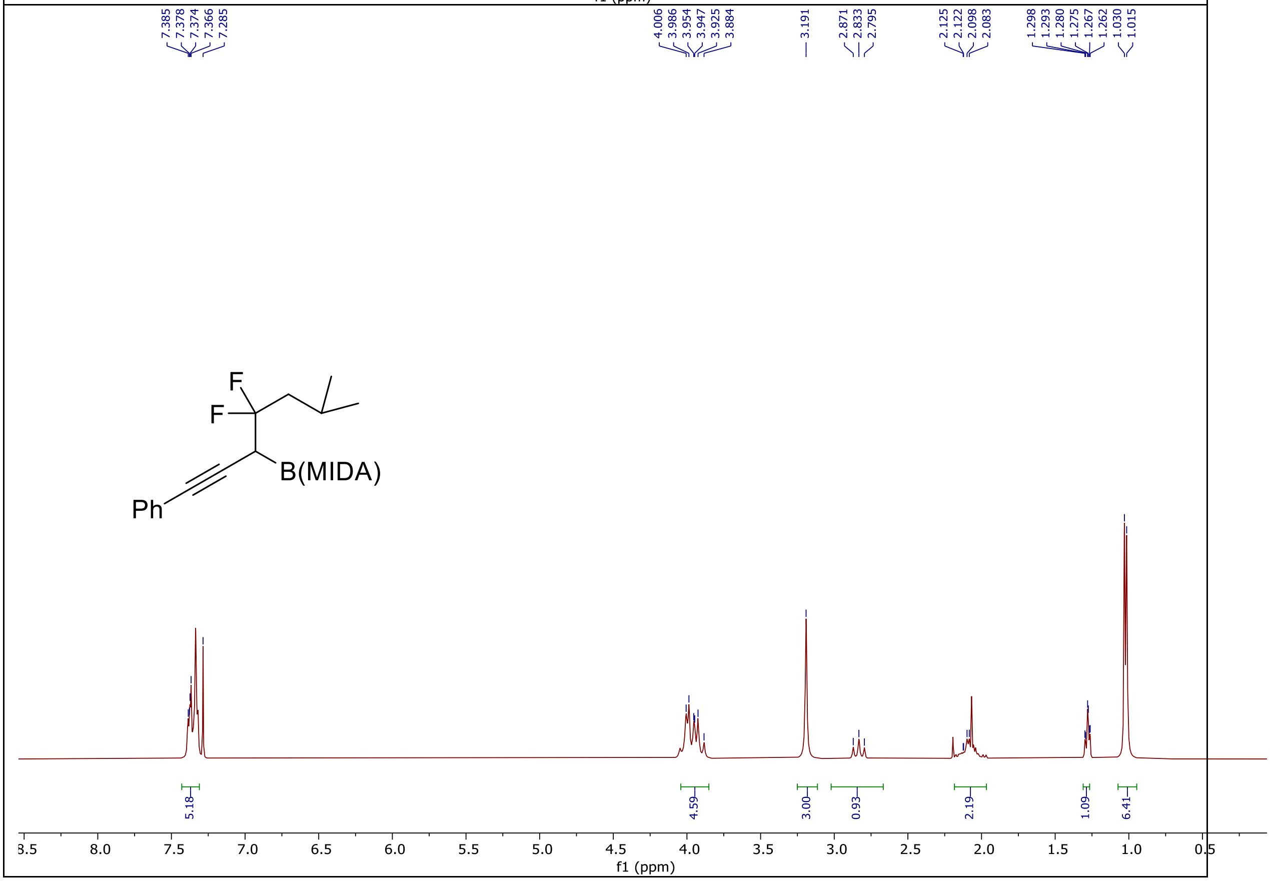
7.385
7.378
7.374
7.366
7.285
4.006
3.986
3.954
3.947
3.925
3.884
3.191
2.871
2.833
2.795
2.125
2.122
2.098
2.083
1.298
1.293
1.280
1.275
1.267
1.262
1.030
1.015
F
F
B(MIDA)
Ph
5.18
4.59
3.00
0.93
2.19
1.09
6.41
f1 (ppm)

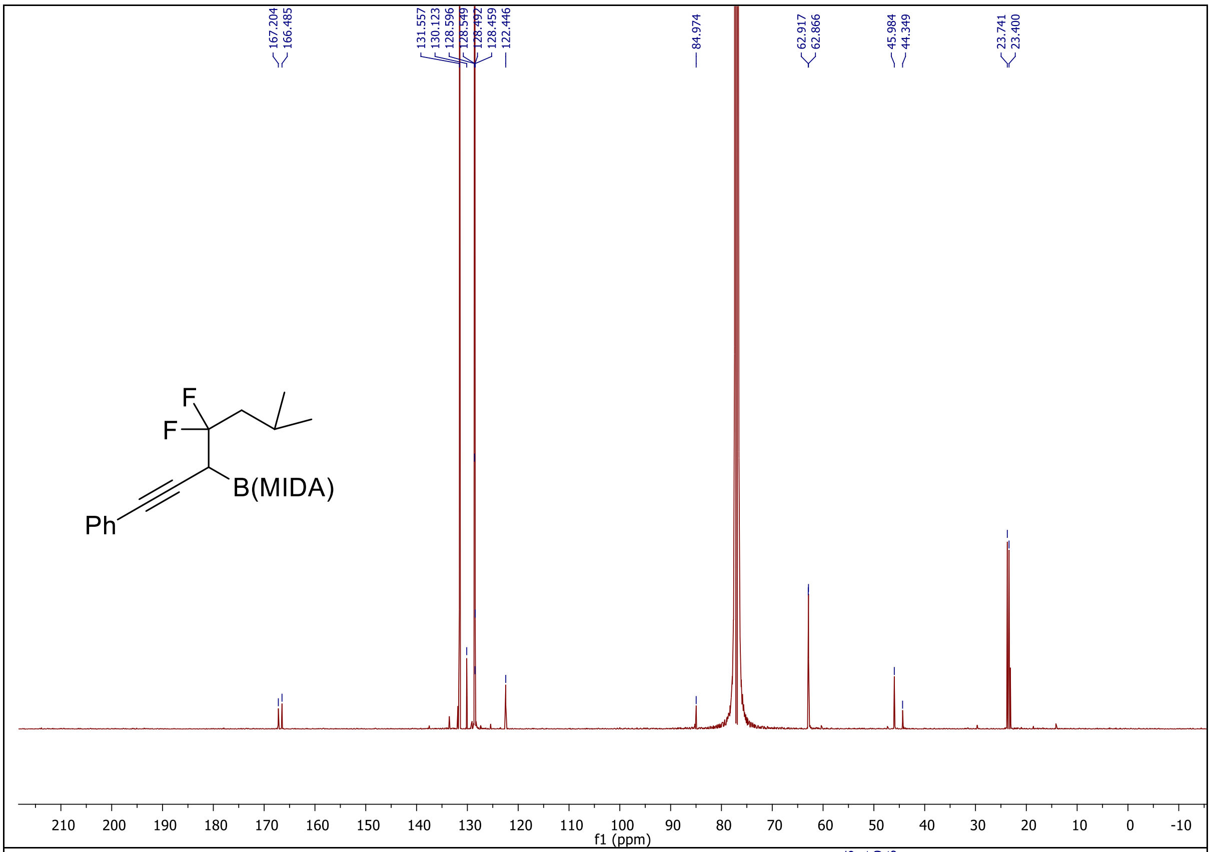
167.204
166.485
131.557
130.123
128.596
128.549
128.492
128.459
122.446
84.974
62.917
62.866
45.984
44.349
23.741
23.400
F
F
B(MIDA)
Ph
210 200 190 180 170 160 150 140 130 120 110 100 90 80 70 60 50 40 30 20 10 0 -10
f1 (ppm)

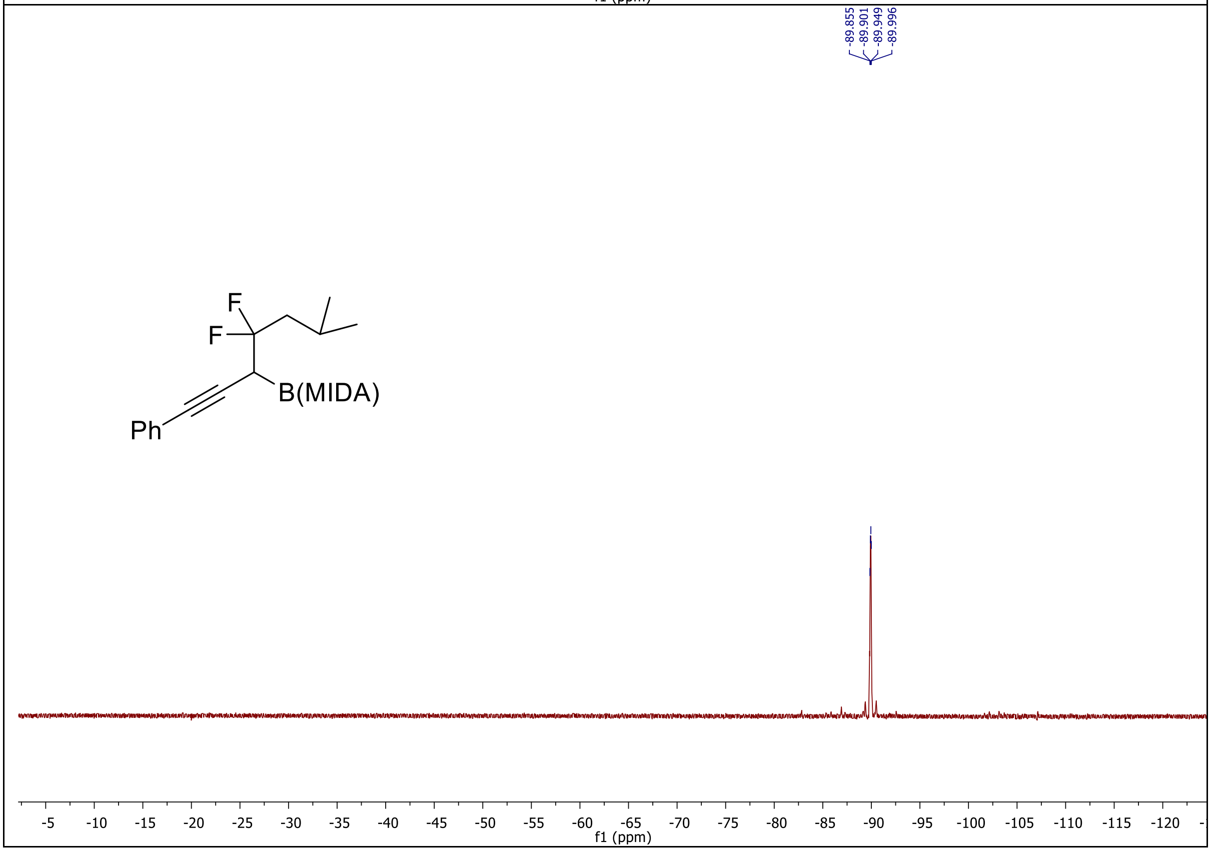
-89.855
-89.901
-89.949
-89.996
F
F
B(MIDA)
Ph
-5 -10 -15 -20 -25 -30 -35 -40 -45 -50 -55 -60 -65 -70 -75 -80 -85 -90 -95 -100 -105 -110 -115 -120
f1 (ppm)

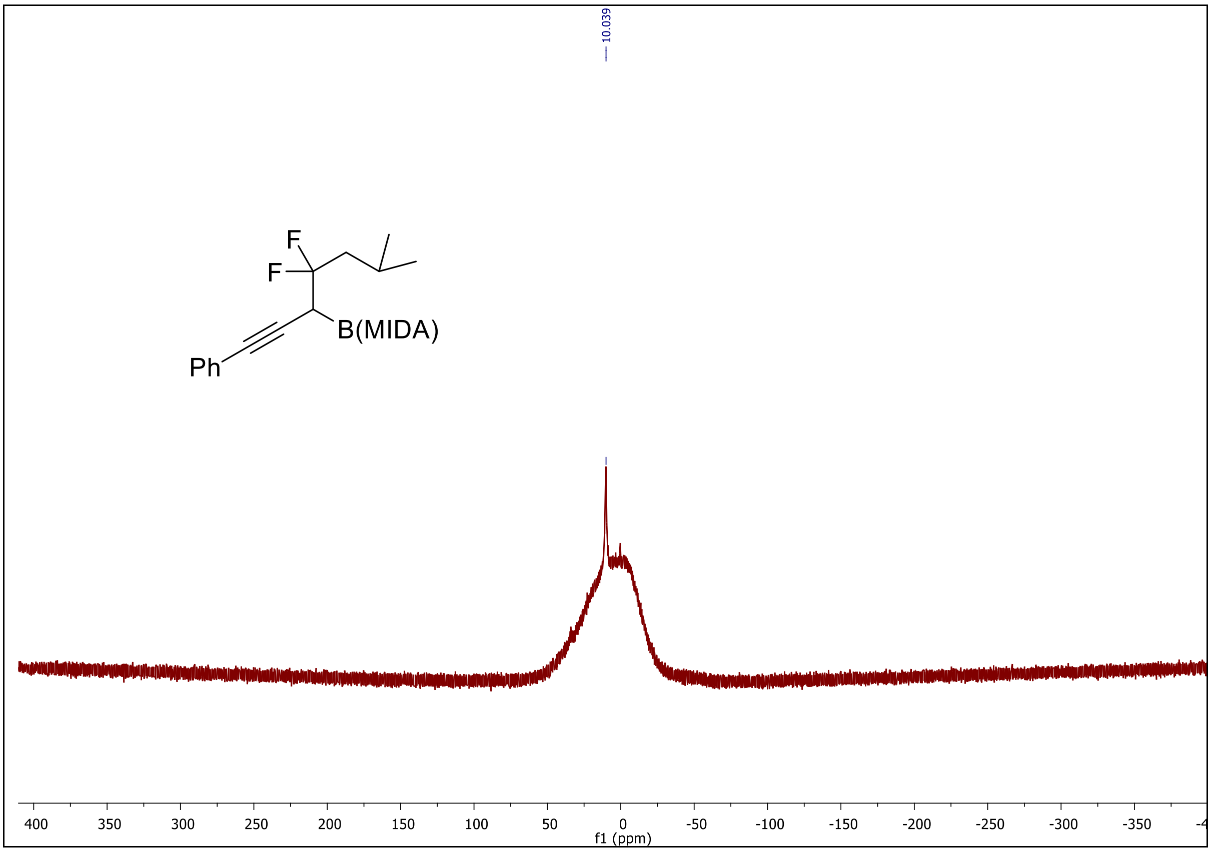

10.039
F
F
B(MIDA)
Ph
400
350
300
250
200
150
100
50
0
-50
-100
-150
-200
-250
-300
-350
f1 (ppm)

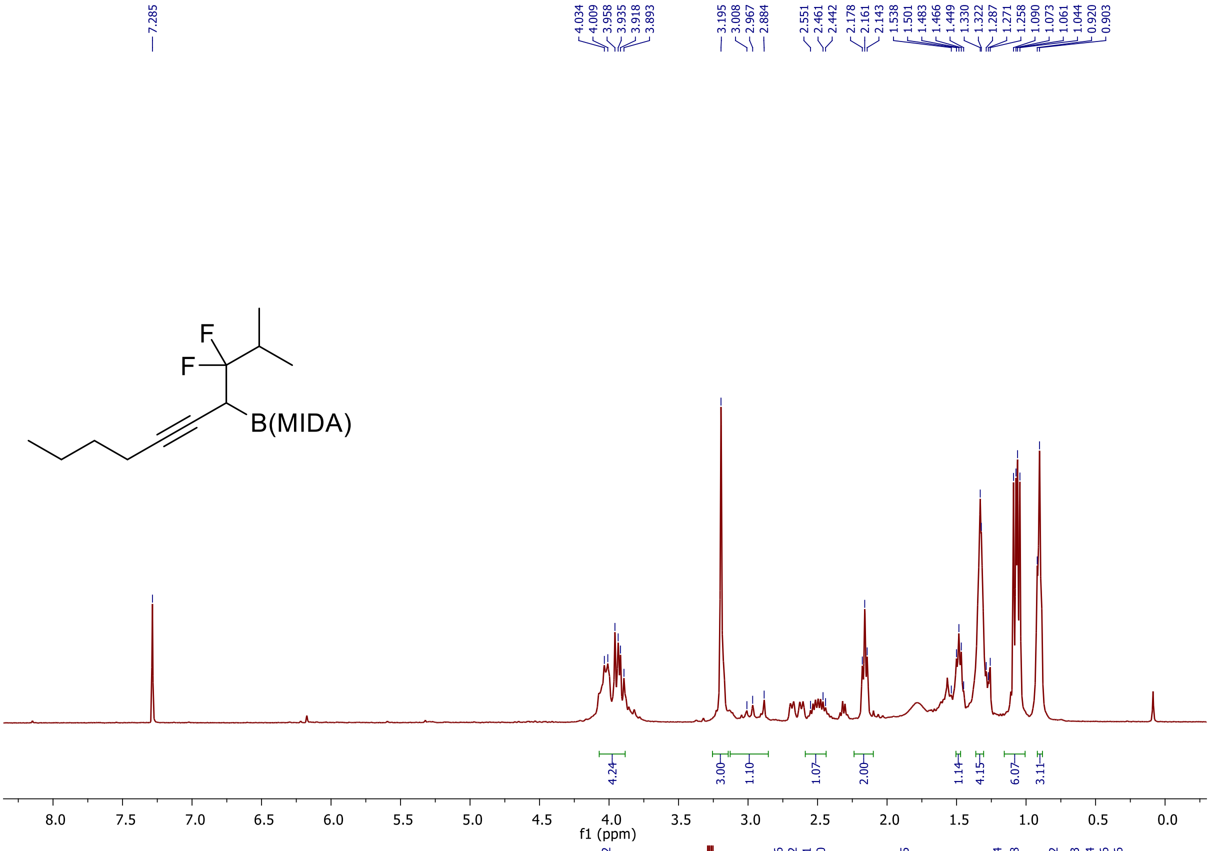
7.285
4.034 4.009 3.958 3.935 3.918 3.893
3.195 3.008 2.967 2.884
2.551 2.461 2.442 2.178 2.161 2.143 1.538 1.501 1.483 1.466 1.449 1.330 1.322 1.287 1.271 1.258 1.090 1.073 1.061 1.044 0.920 0.903
F
F
B(MIDA)
4.24 3.00 1.10 1.07 2.00 1.14 4.15 6.07 3.11
8.0 7.5 7.0 6.5 6.0 5.5 5.0 4.5 4.0 3.5 3.0 2.5 2.0 1.5 1.0 0.5 0.0
f1 (ppm)

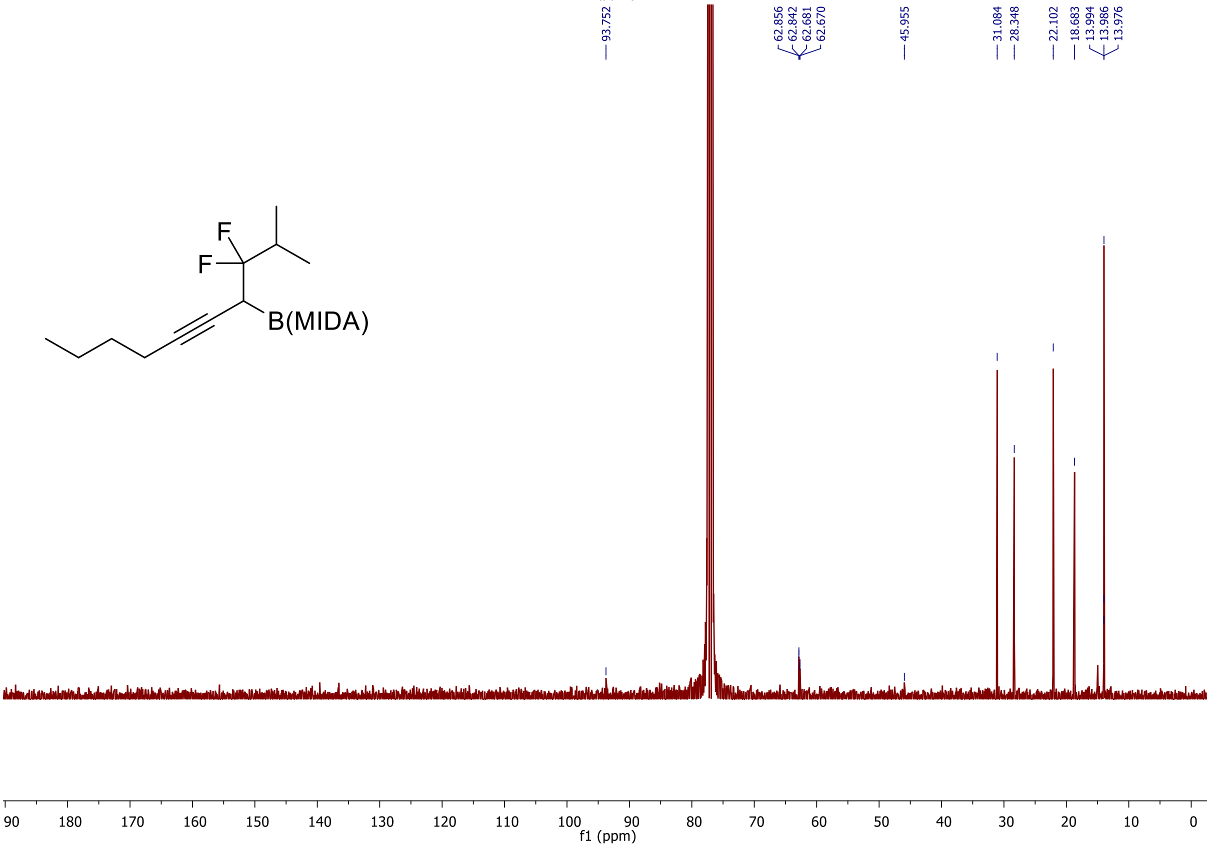
93.752
62.856 62.842 62.681 62.670
45.955
31.084 28.348
22.102 18.683 13.994 13.986 13.976
F
F
B(MIDA)
90 180 170 160 150 140 130 120 110 100 90 80 70 60 50 40 30 20 10 0
f1 (ppm)

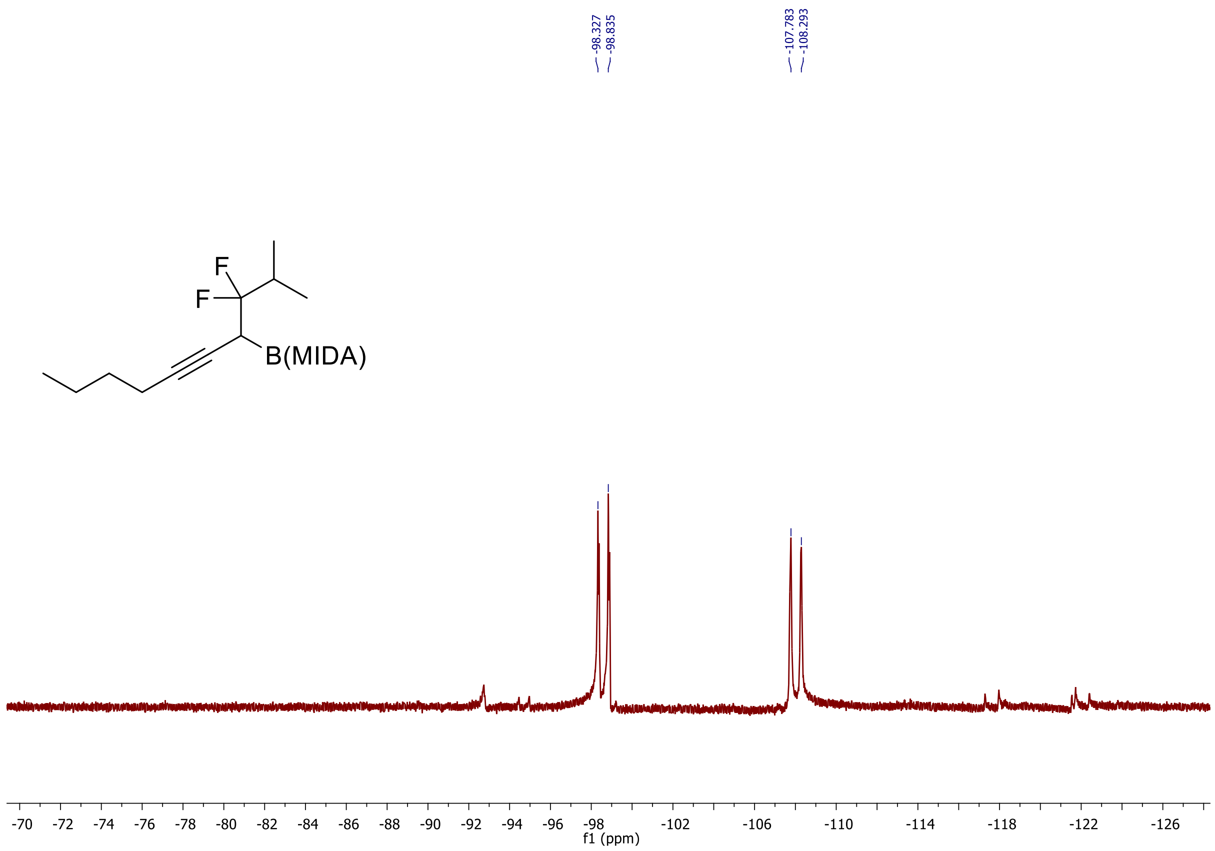

-98.327
-98.835
-107.783
-108.293
F
F
B(MIDA)
-70 -72 -74 -76 -78 -80 -82 -84 -86 -88 -90 -92 -94 -96 -98 -102 -106 -110 -114 -118 -122 -126
f1 (ppm)

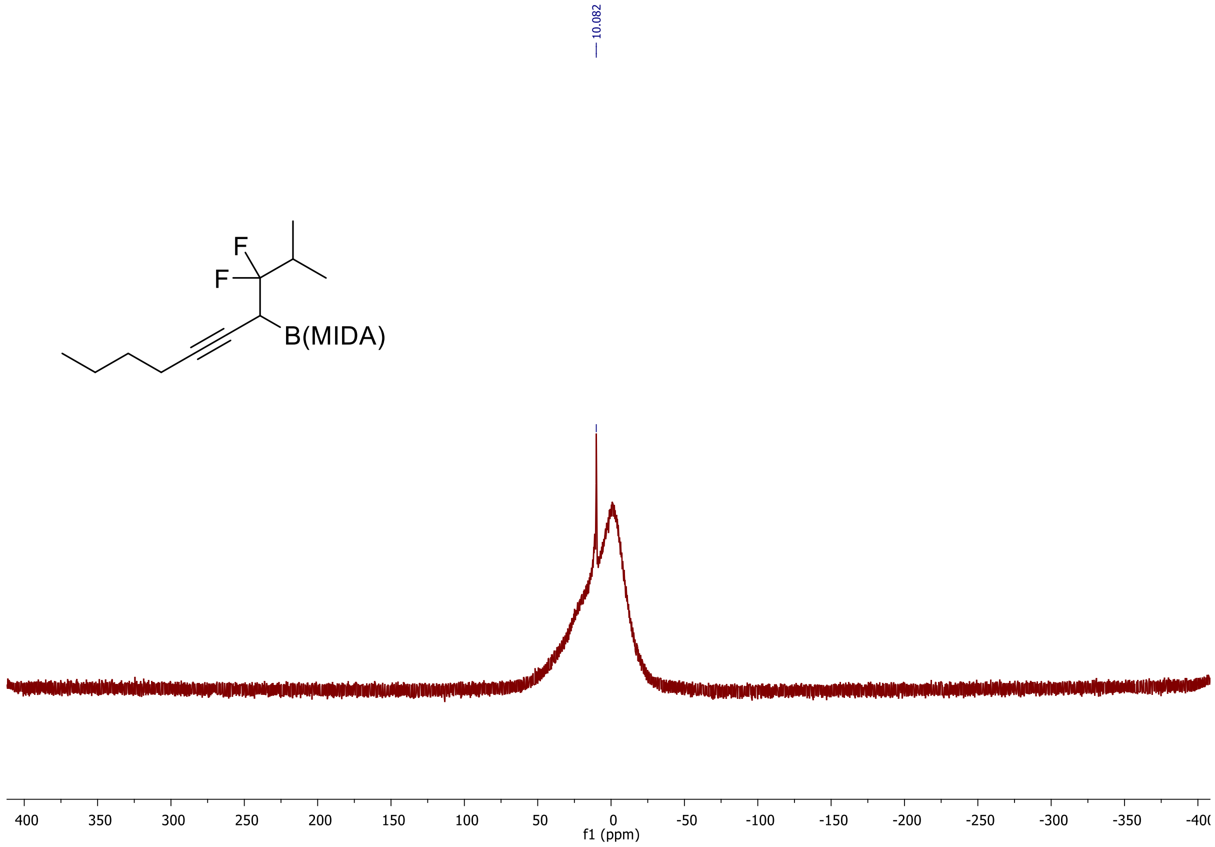

10.082
F
F
B(MIDA)
400 350 300 250 200 150 100 50 0 -50 -100 -150 -200 -250 -300 -350
f1 (ppm)

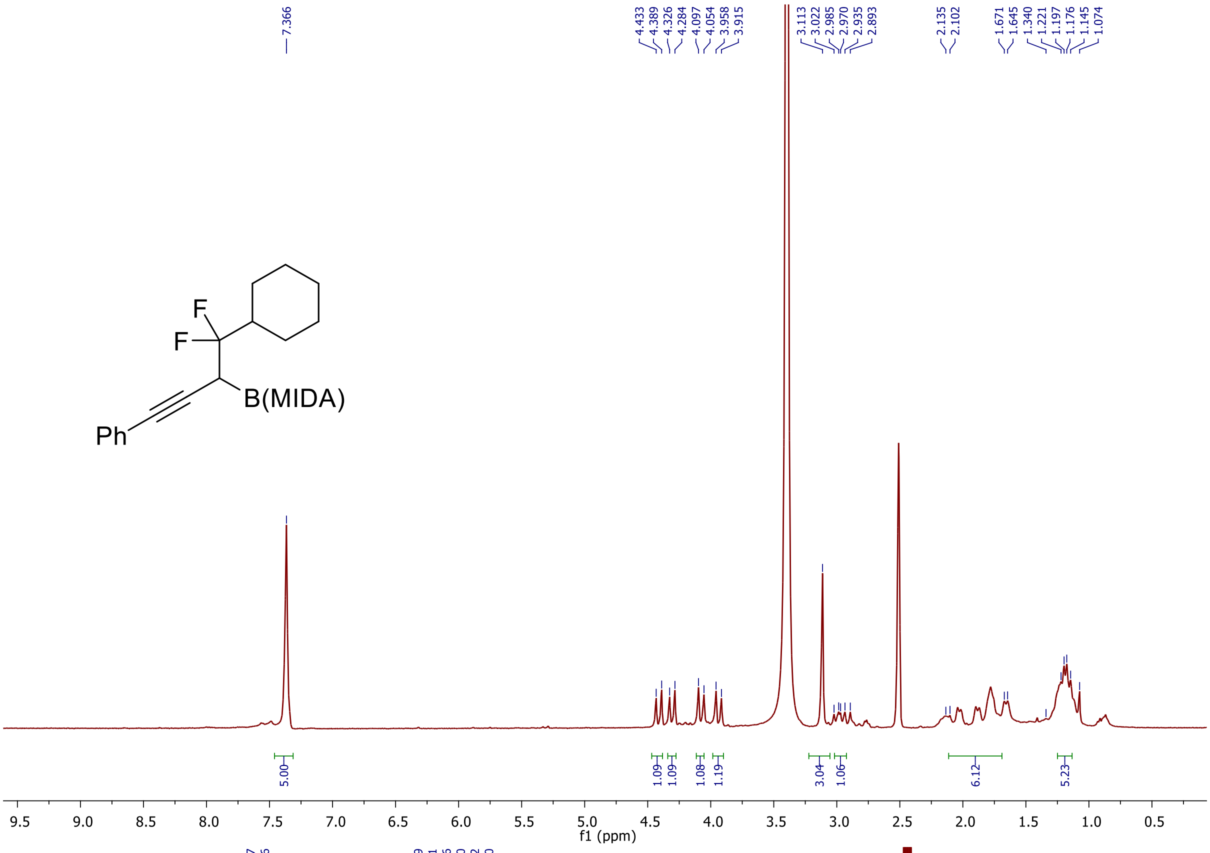
7.366
4.433
4.389
4.326
4.284
4.097
4.054
3.958
3.915
3.113
3.022
2.985
2.970
2.935
2.893
2.135
2.102
1.671
1.645
1.340
1.221
1.197
1.176
1.145
1.074
F
F
B(MIDA)
Ph
5.00
1.09
1.09
1.08
1.19
3.04
1.06
6.12
5.23
f1 (ppm)

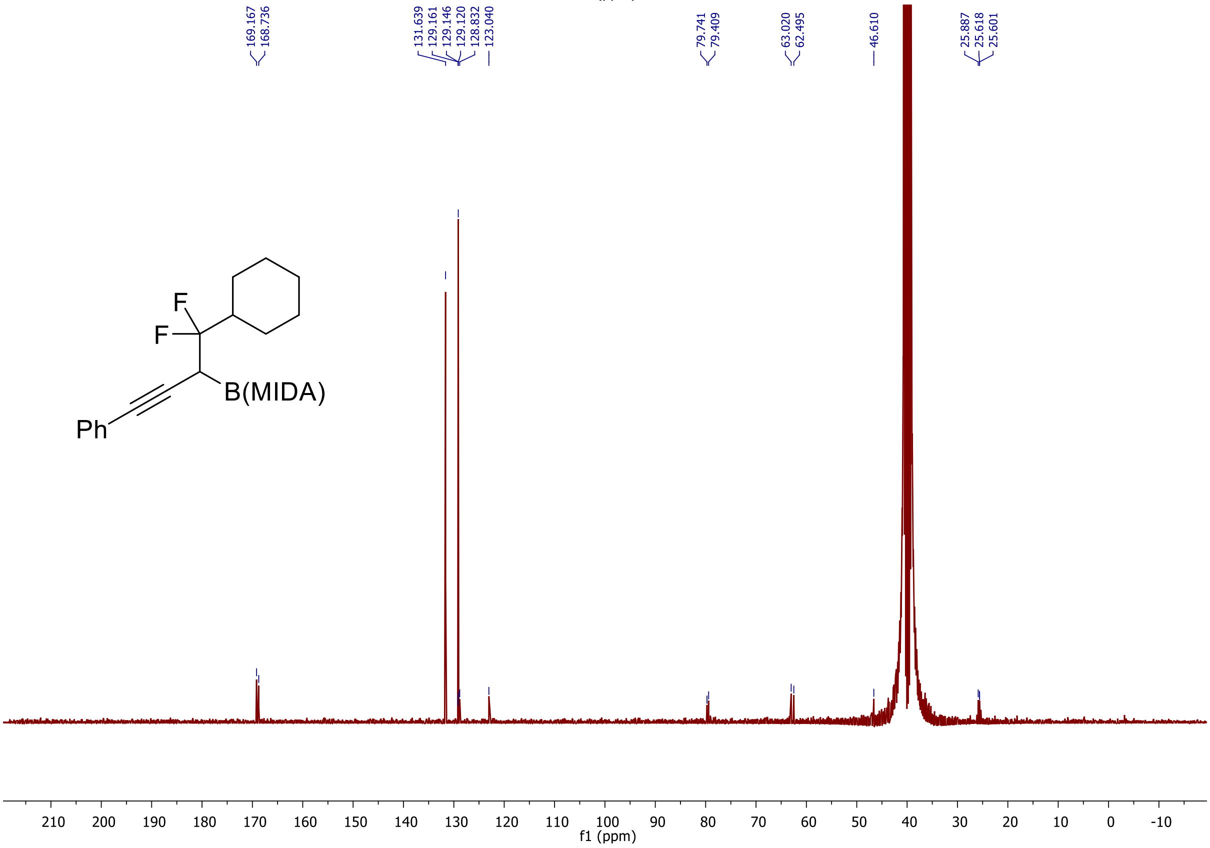
169.167
168.736
131.639
129.161
129.146
129.120
128.832
123.040
79.741
79.409
63.020
62.495
46.610
25.887
25.618
25.601
F
F
B(MIDA)
Ph
f1 (ppm)

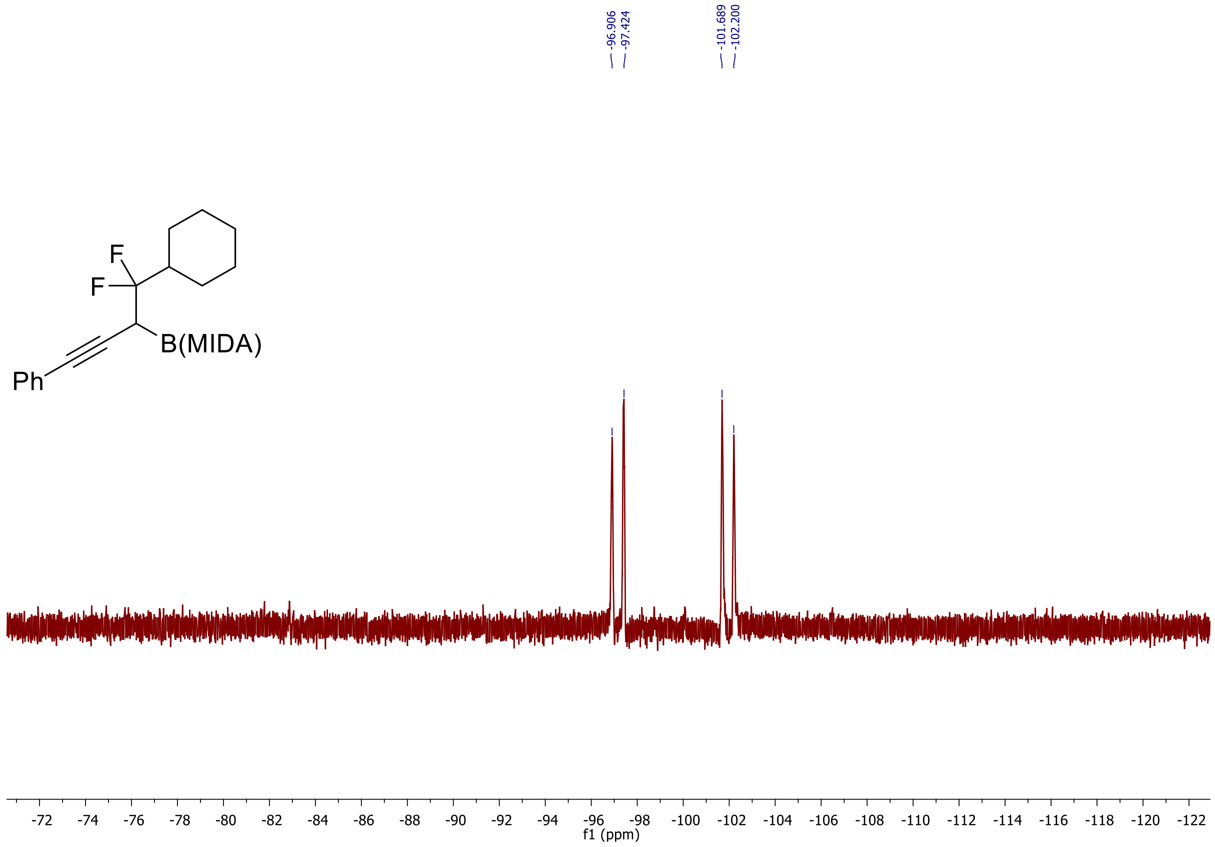

-96.906
-97.424
-101.689
-102.200
F
F
B(MIDA)
Ph
-72
-74
-76
-78
-80
-82
-84
-86
-88
-90
-92
-94
-96
-98
-100
-102
-104
-106
-108
-110
-112
-114
-116
-118
-120
-122
f1 (ppm)

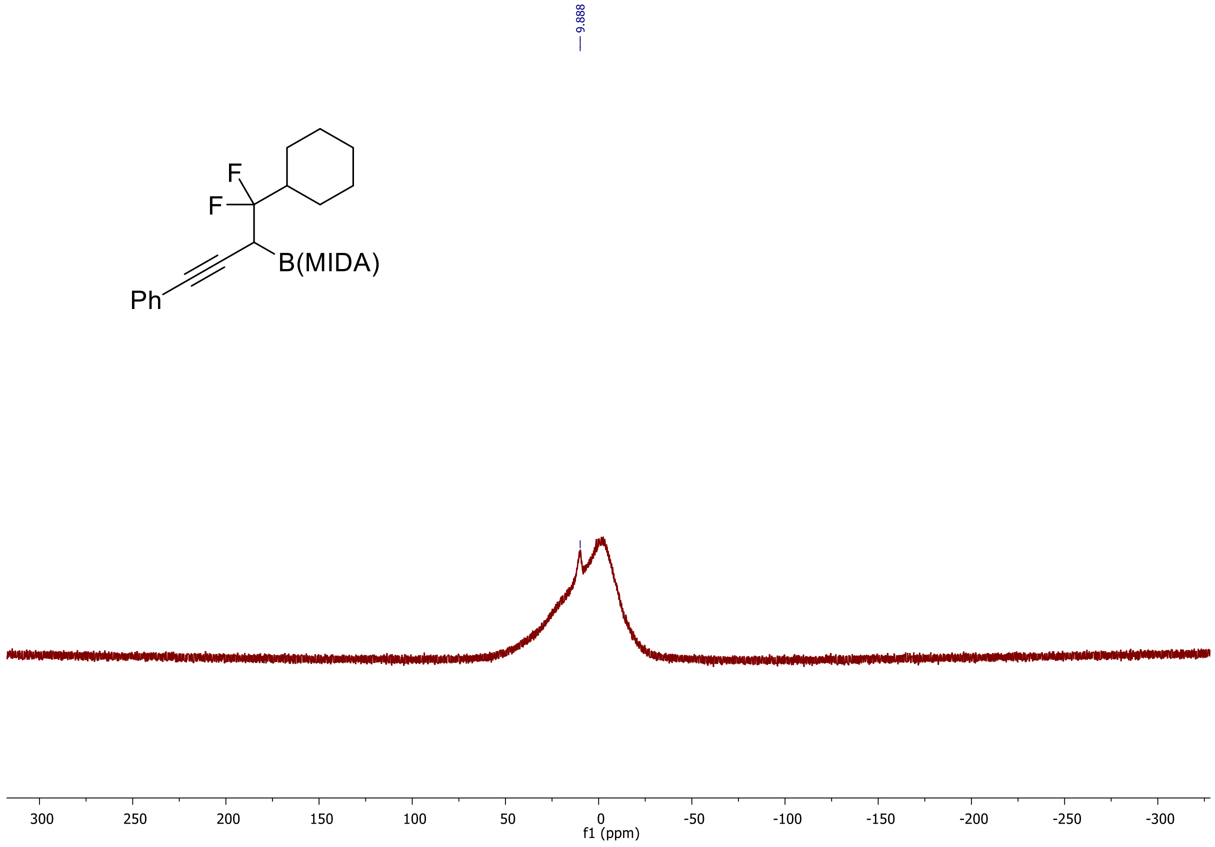

9.888
F
F
B(MIDA)
Ph
300
250
200
150
100
50
0
-50
-100
-150
-200
-250
-300
f1 (ppm)

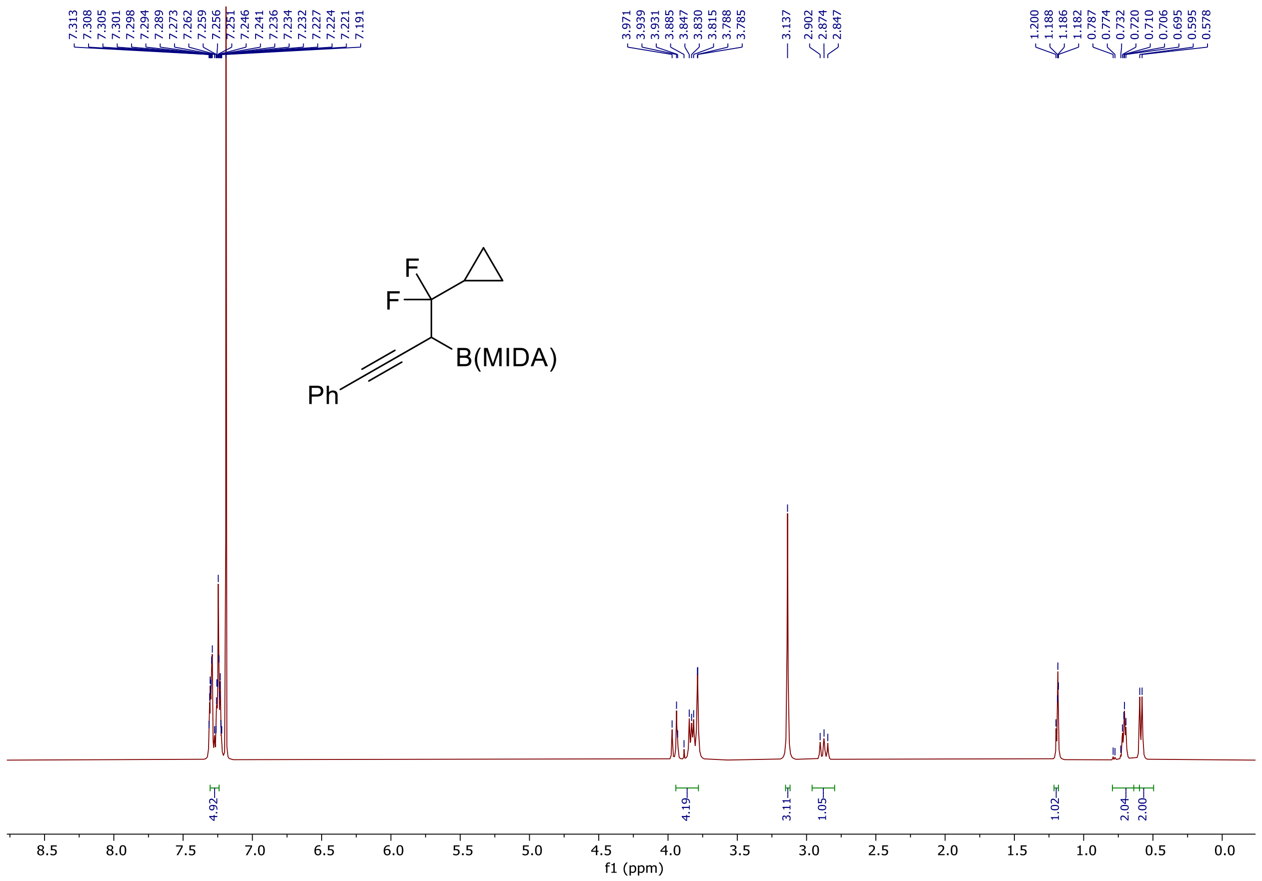

7.313
7.308
7.305
7.301
7.298
7.294
7.289
7.273
7.262
7.259
7.256
7.251
7.246
7.241
7.236
7.234
7.232
7.227
7.224
7.221
7.191
3.971
3.939
3.931
3.885
3.847
3.830
3.815
3.788
3.785
3.137
2.902
2.874
2.847
1.200
1.188
1.186
1.182
0.787
0.774
0.732
0.720
0.710
0.706
0.695
0.595
0.578
F
F
B(MIDA)
Ph
4.92
4.19
3.11
1.05
1.02
2.04
2.00
f1 (ppm)

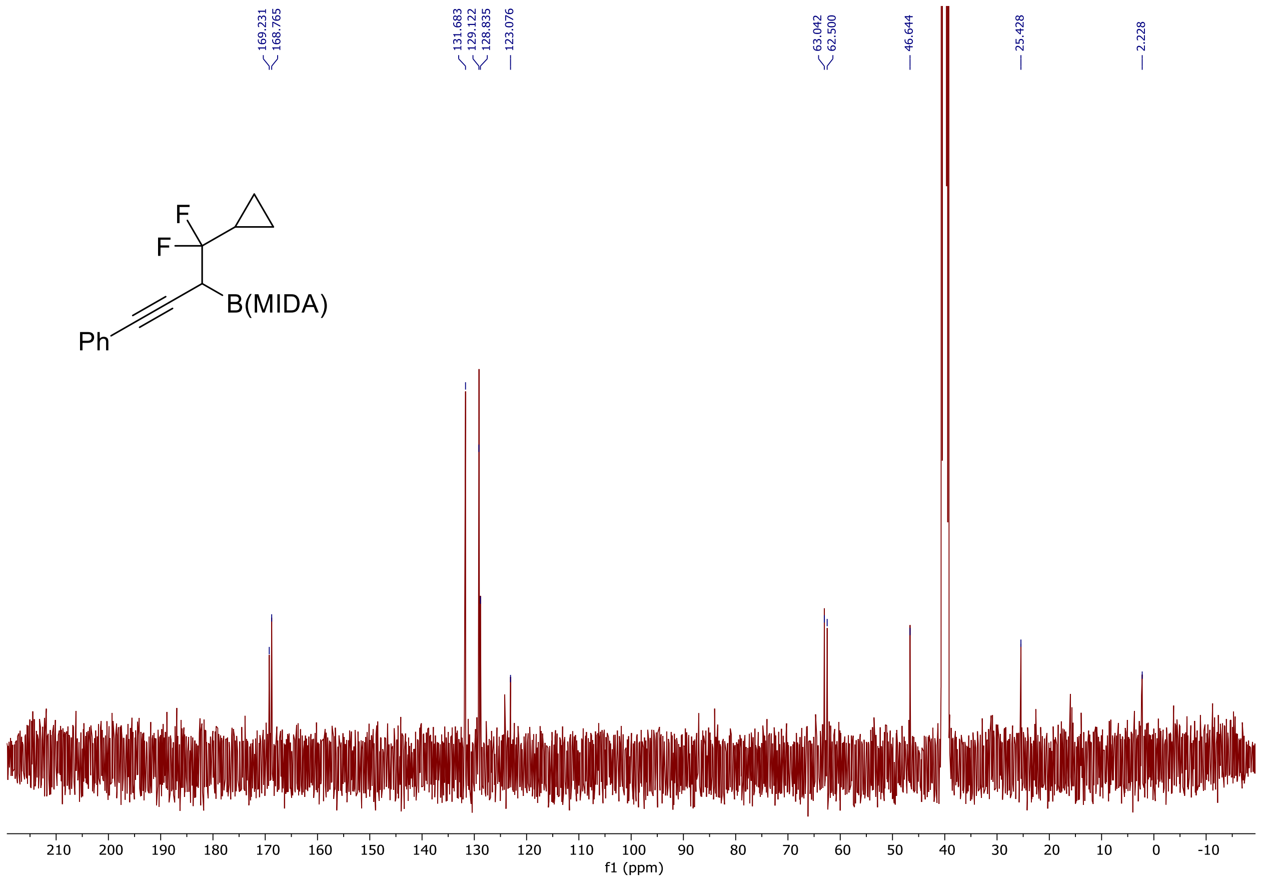

169.231
168.765
131.683
129.122
128.835
123.076
63.042
62.500
46.644
25.428
2.228
F
F
B(MIDA)
Ph
f1 (ppm)

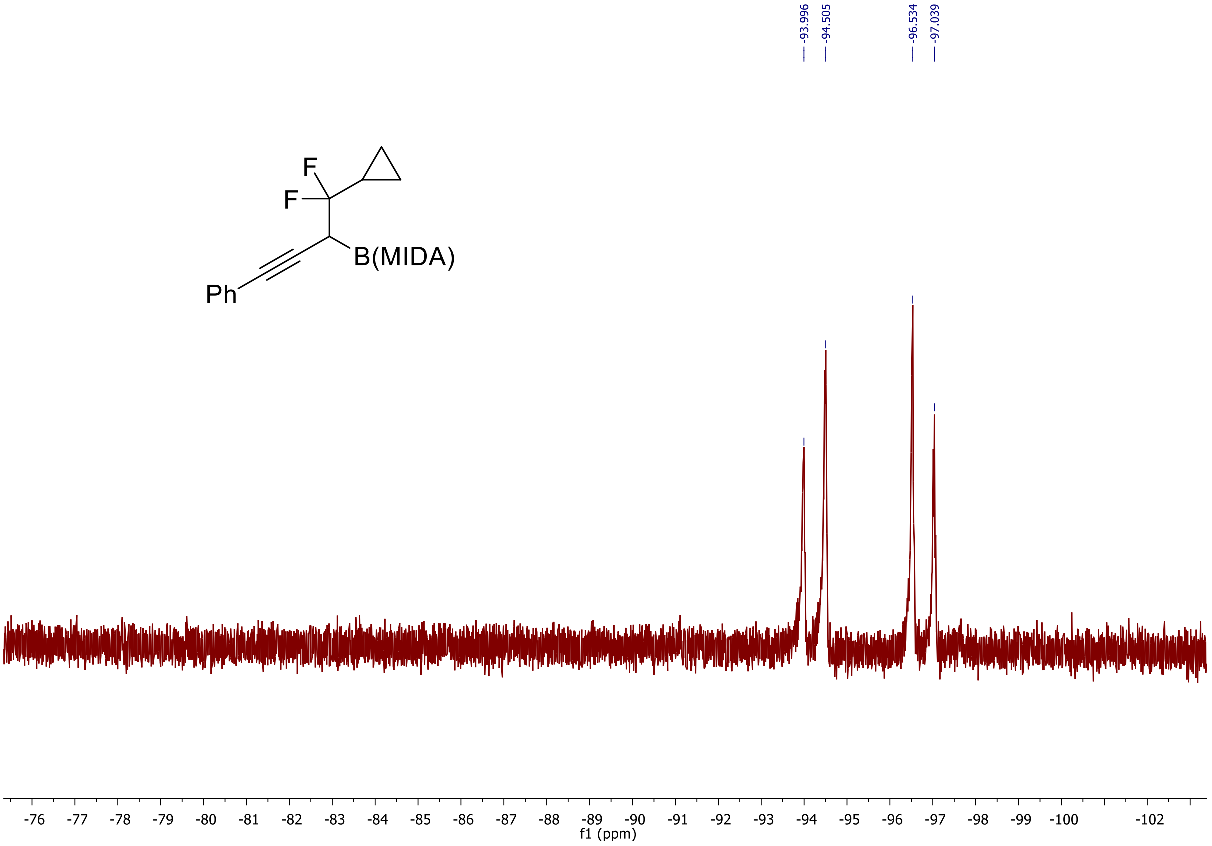
F
F
B(MIDA)
Ph
-93.996
-94.505
-96.534
-97.039
-76 -77 -78 -79 -80 -81 -82 -83 -84 -85 -86 -87 -88 -89 -90 -91 -92 -93 -94 -95 -96 -97 -98 -99 -100 -102
f1 (ppm)

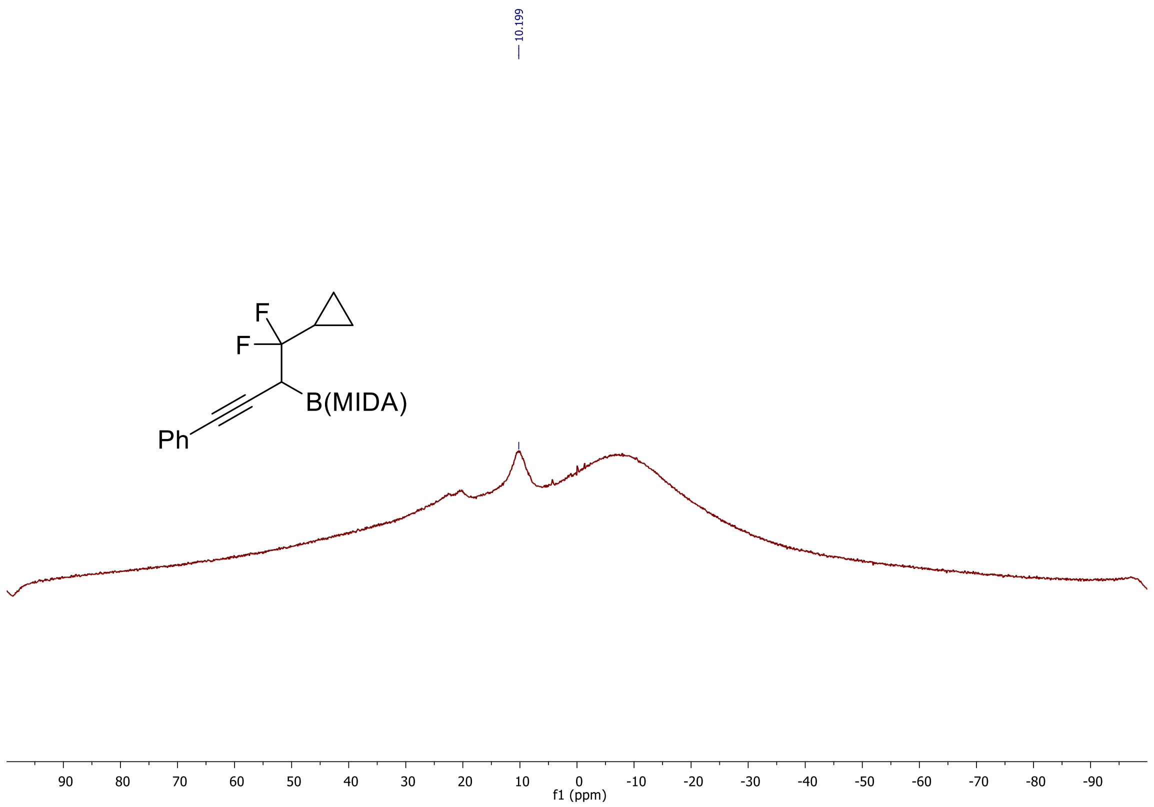
10.199
F
F
B(MIDA)
Ph
90 80 70 60 50 40 30 20 10 0 -10 -20 -30 -40 -50 -60 -70 -80 -90
f1 (ppm)

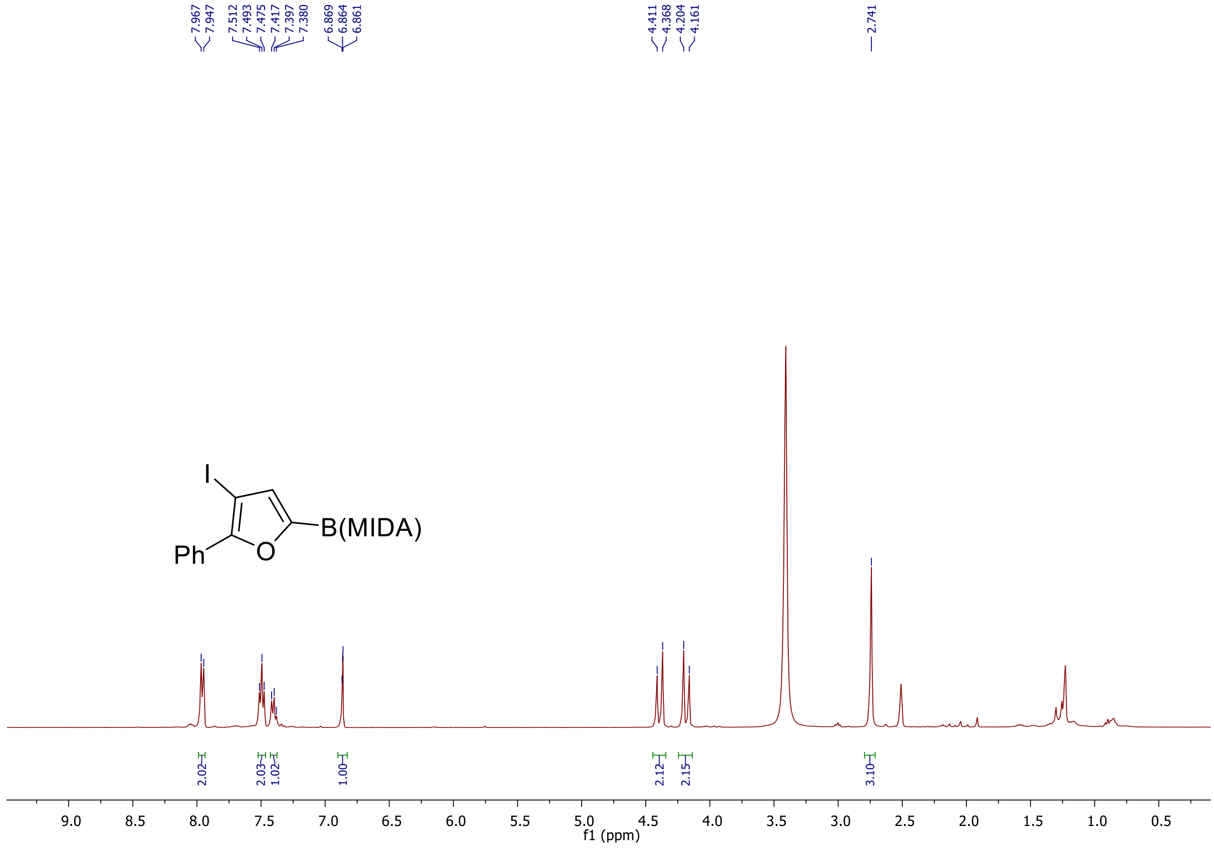
7.967
7.947
7.512
7.493
7.475
7.417
7.397
7.380
6.869
6.864
6.861
4.411
4.368
4.204
4.161
2.741
I
B(MIDA)
Ph
O
2.02
2.03
1.02
1.00
2.12
2.15
3.10
9.0 8.5 8.0 7.5 7.0 6.5 6.0 5.5 5.0 4.5 4.0 3.5 3.0 2.5 2.0 1.5 1.0 0.5
f1 (ppm)

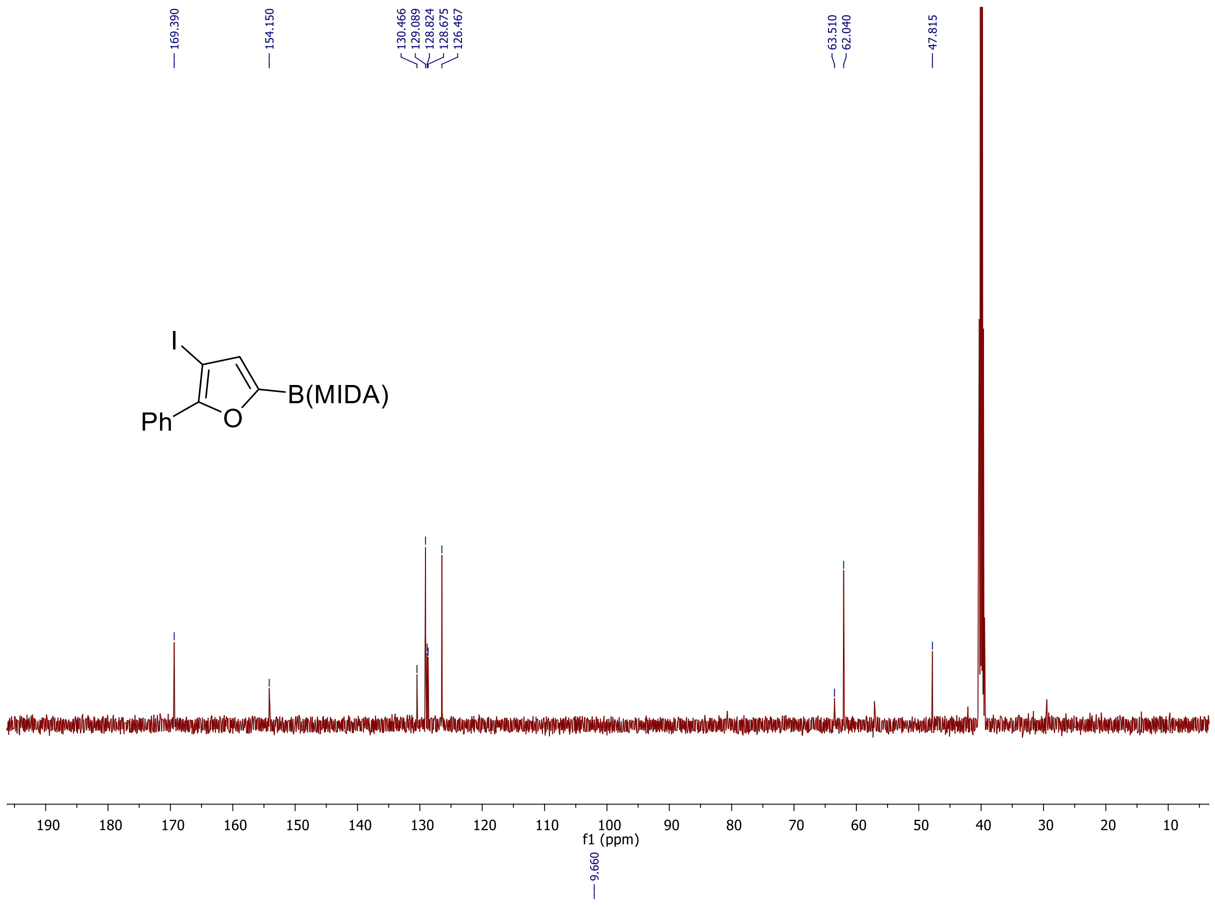
169.390
154.150
130.466
129.089
128.824
128.675
126.467
63.510
62.040
47.815
I
Ph
O
B(MIDA)
f1 (ppm)

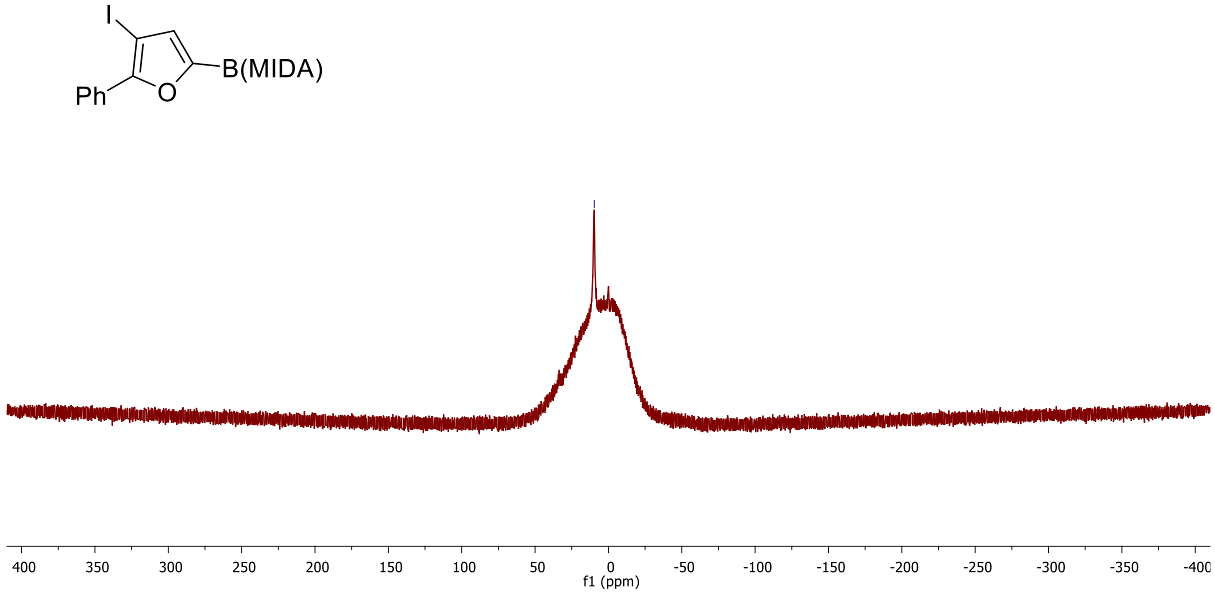
9.660
I
Ph
O
B(MIDA)
f1 (ppm)

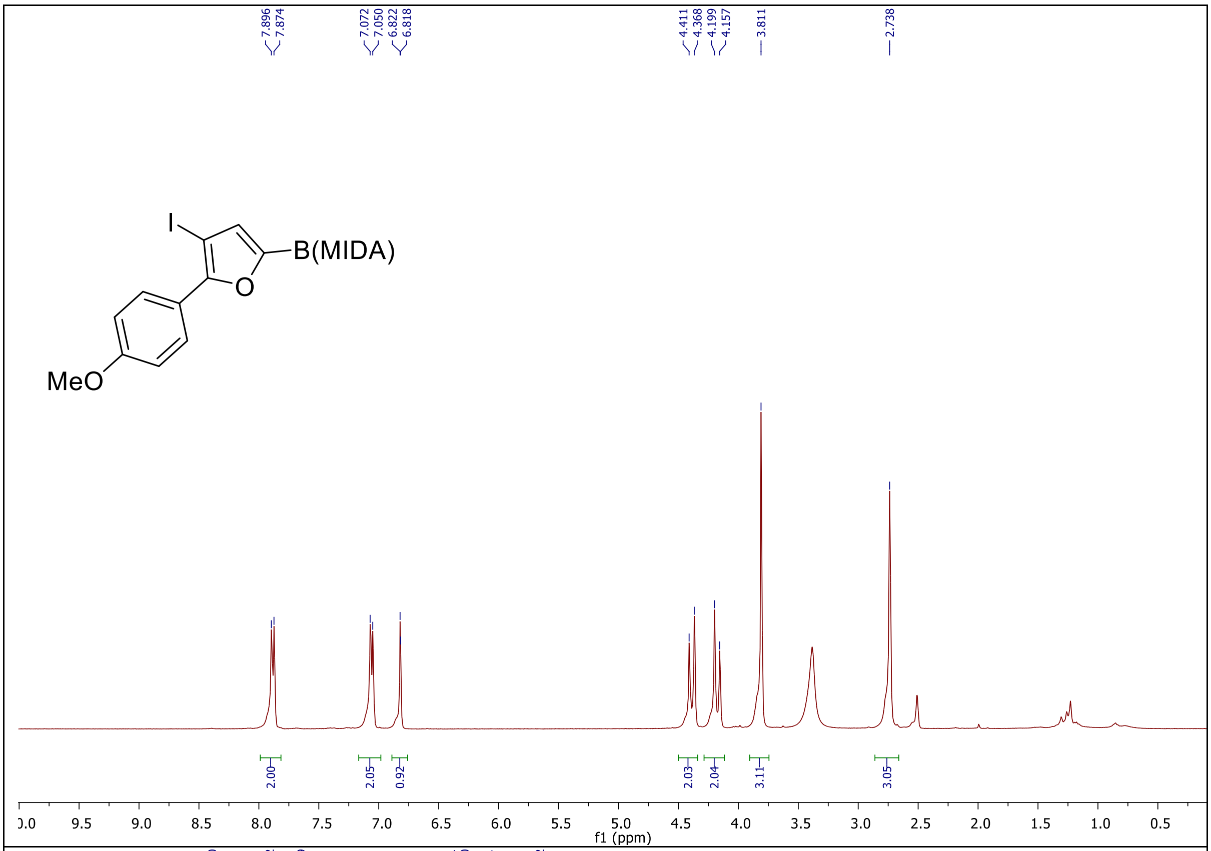

7.896
7.874
7.072
7.050
6.822
6.818
4.411
4.368
4.199
4.157
3.811
2.738
B(MIDA)
MeO
2.00
2.05
0.92
2.03
2.04
3.11
3.05
f1 (ppm)

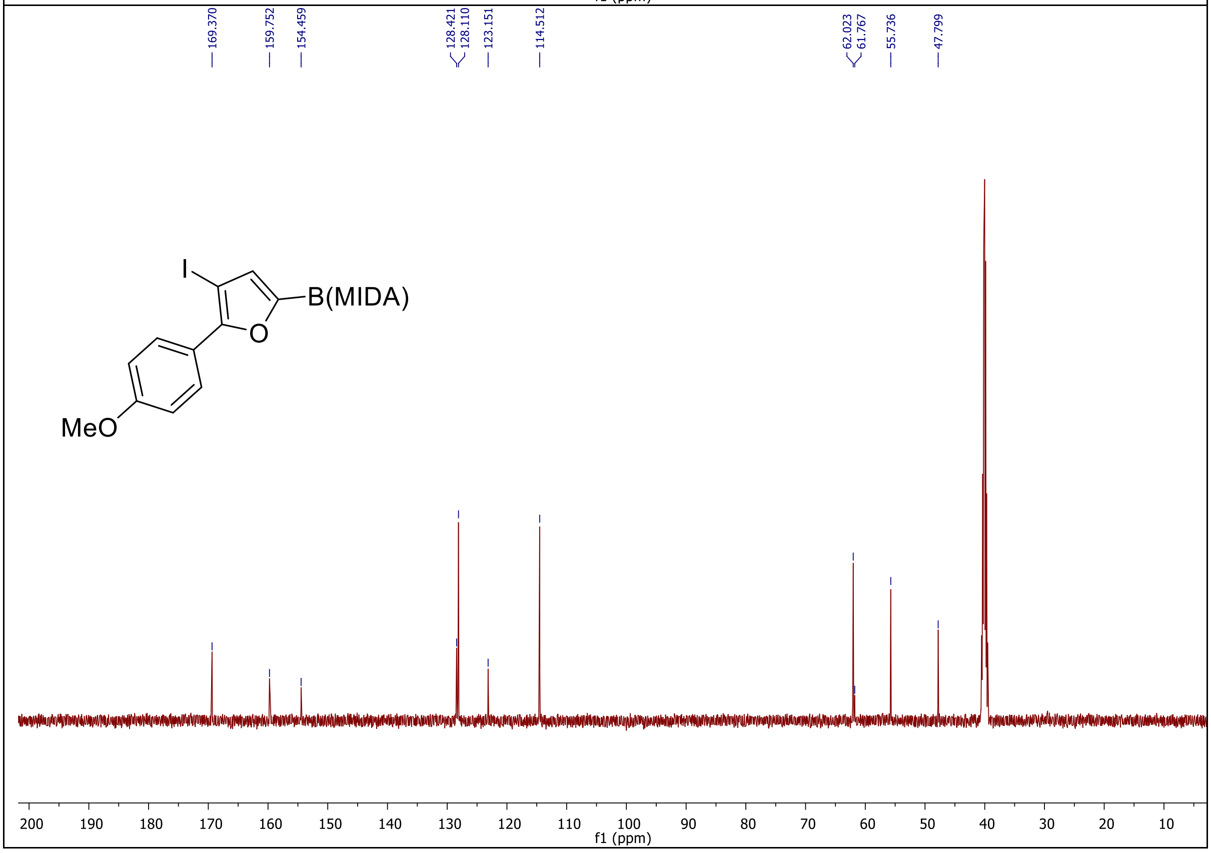

169.370
159.752
154.459
128.421
128.110
123.151
114.512
62.023
61.767
55.736
47.799
B(MIDA)
MeO
f1 (ppm)

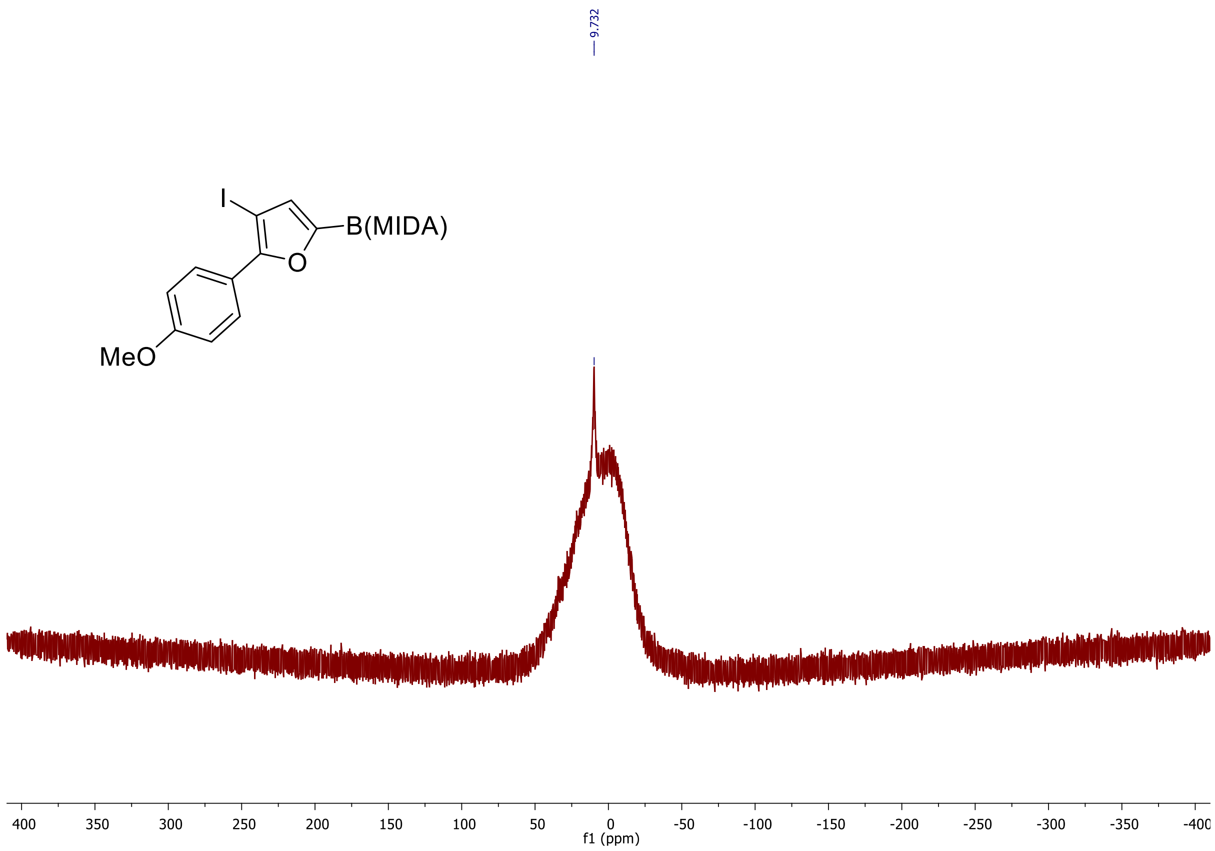

9.732
I
B(MIDA)
O
MeO
400
350
300
250
200
150
100
50
0
-50
-100
-150
-200
-250
-300
-350
-400
f1 (ppm)

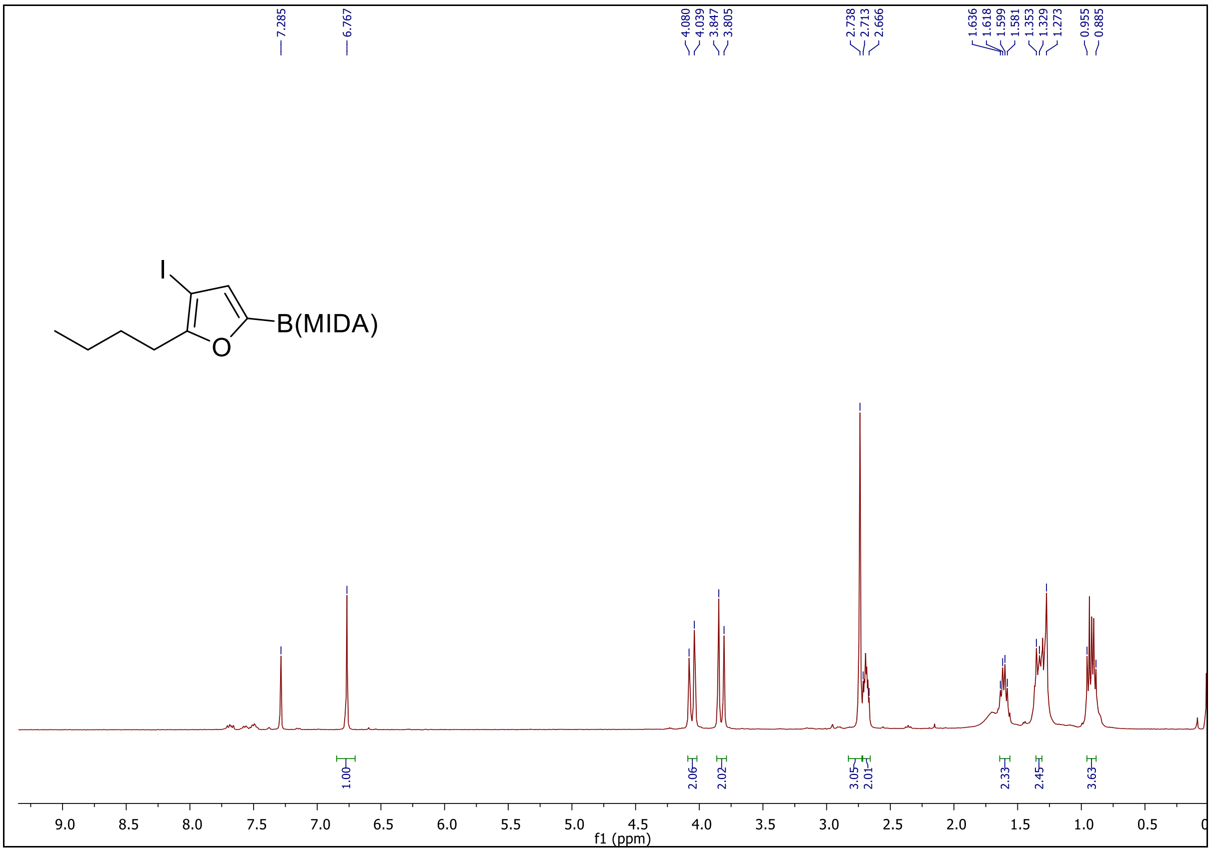

7.285
6.767
4.080
4.039
3.847
3.805
2.738
2.713
2.666
1.636
1.618
1.599
1.581
1.353
1.329
1.273
0.955
0.885
I
B(MIDA)
O
1.00
2.06
2.02
3.05
2.01
2.33
2.45
3.63
9.0
8.5
8.0
7.5
7.0
6.5
6.0
5.5
5.0
4.5
4.0
3.5
3.0
2.5
2.0
1.5
1.0
0.5
f1 (ppm)

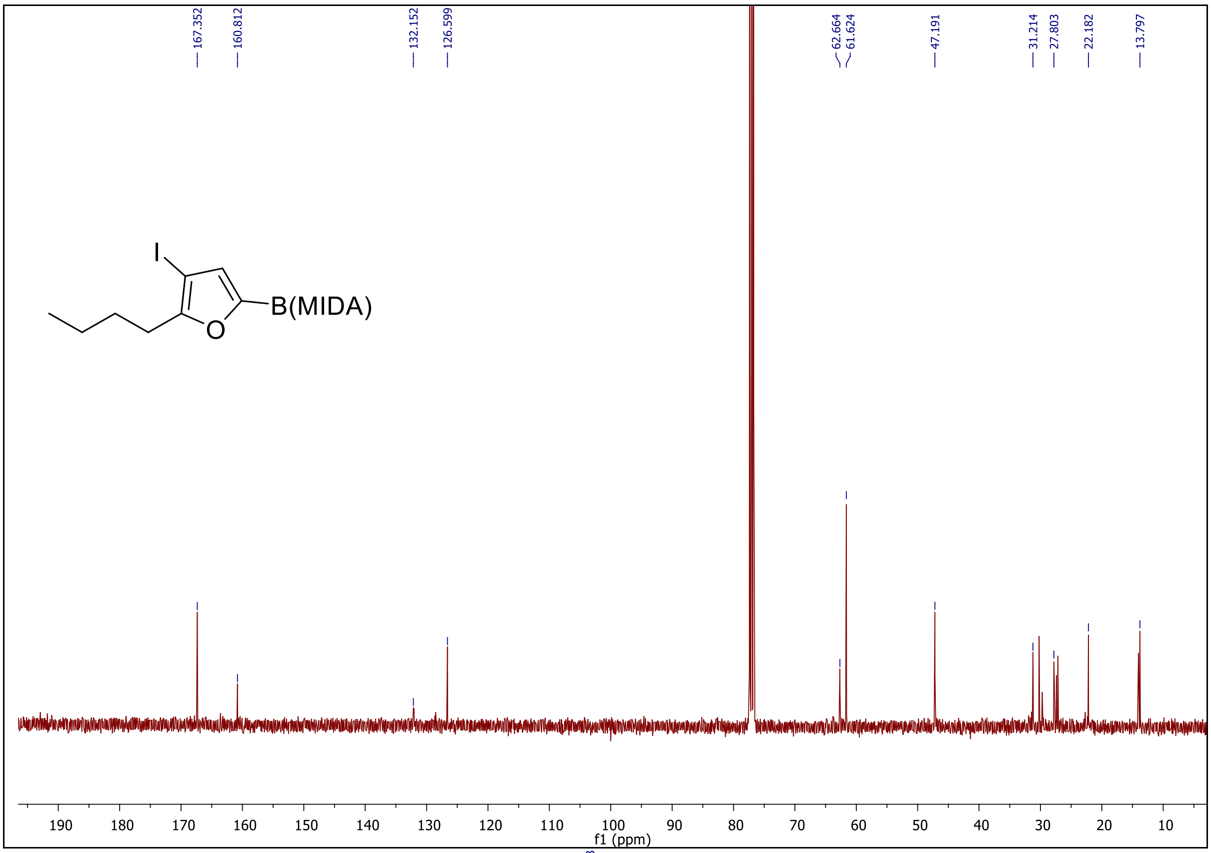
167.352
160.812
132.152
126.599
62.664
61.624
47.191
31.214
27.803
22.182
13.797
I
B(MIDA)
O
190
180
170
160
150
140
130
120
110
100
90
80
70
60
50
40
30
20
10
f1 (ppm)

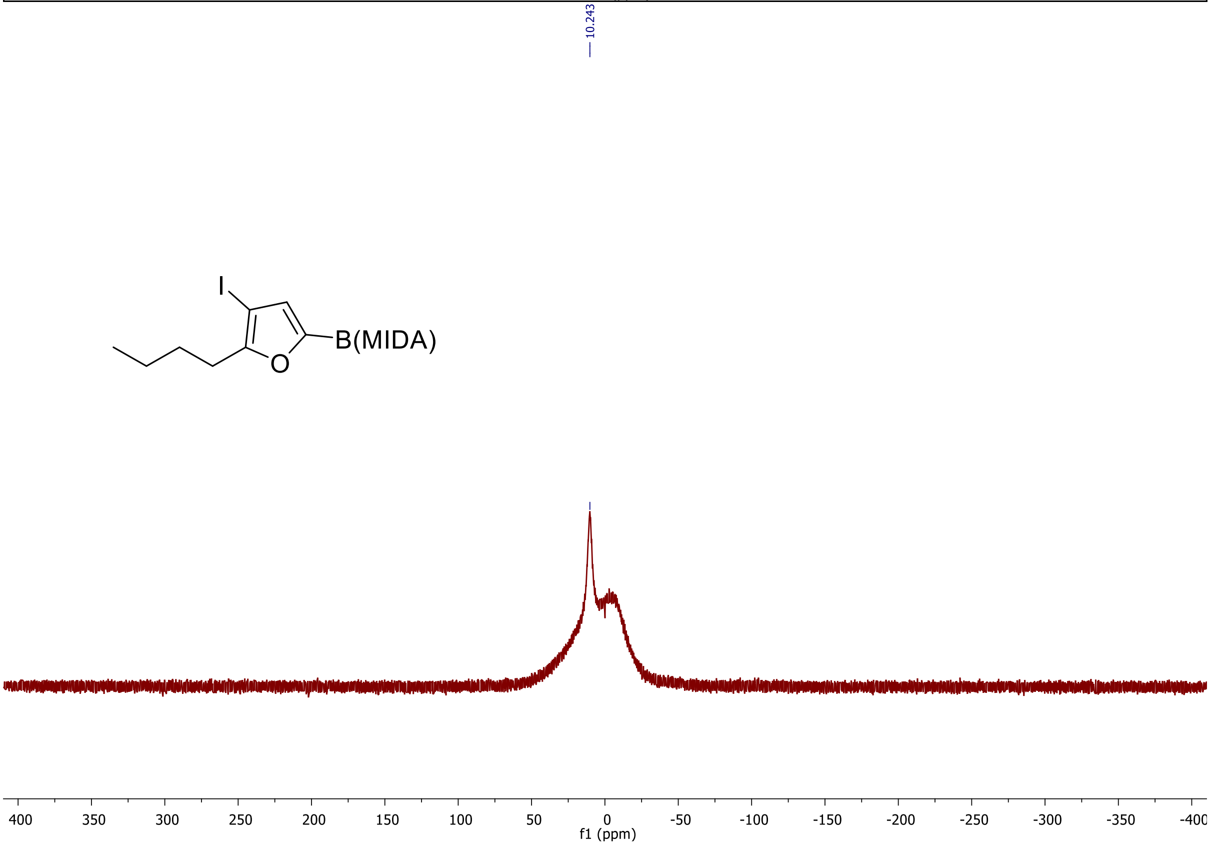
10.243
I
B(MIDA)
O
400
350
300
250
200
150
100
50
0
-50
-100
-150
-200
-250
-300
-350
-400
f1 (ppm)

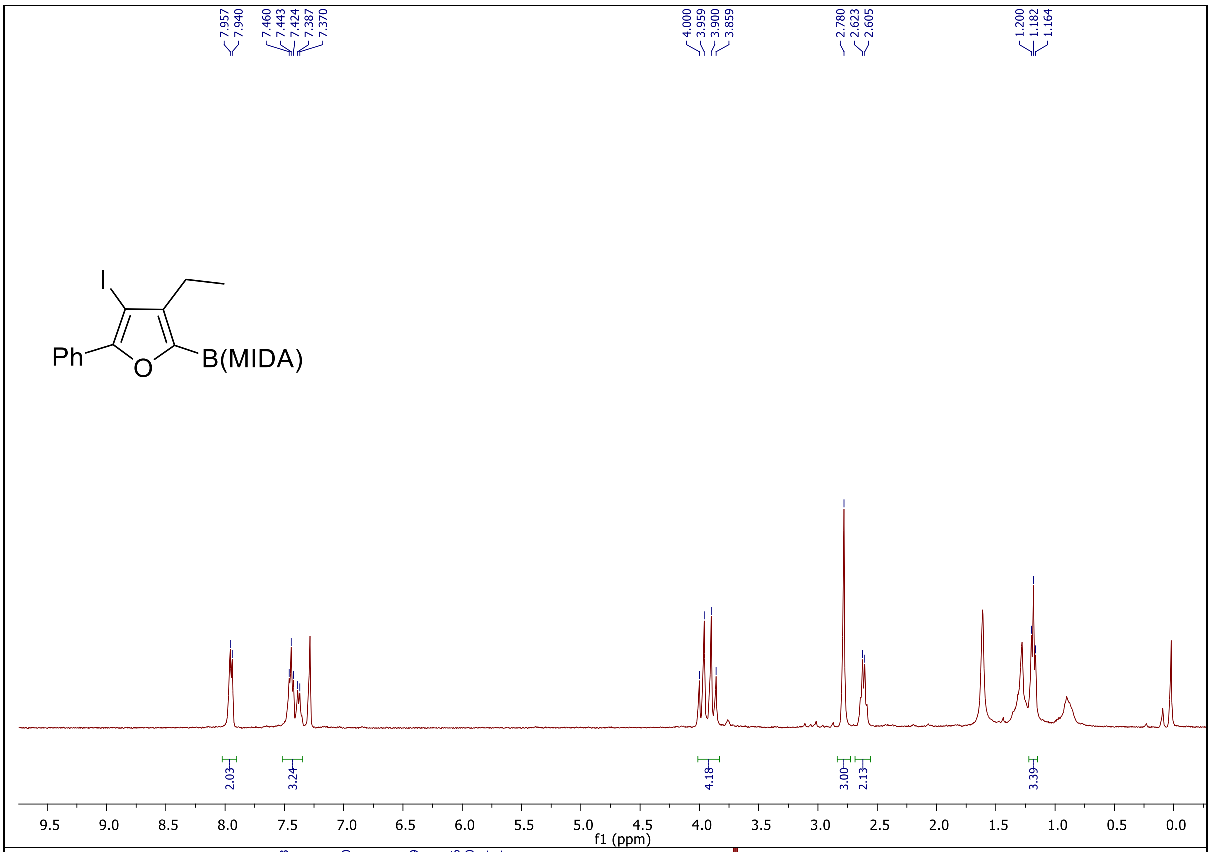

7.957
7.940
7.460
7.443
7.424
7.387
7.370
4.000
3.959
3.900
3.859
2.780
2.623
2.605
1.200
1.182
1.164
I
Ph
O
B(MIDA)
2.03
3.24
4.18
3.00
2.13
3.39
9.5 9.0 8.5 8.0 7.5 7.0 6.5 6.0 5.5 5.0 4.5 4.0 3.5 3.0 2.5 2.0 1.5 1.0 0.5 0.0
f1 (ppm)

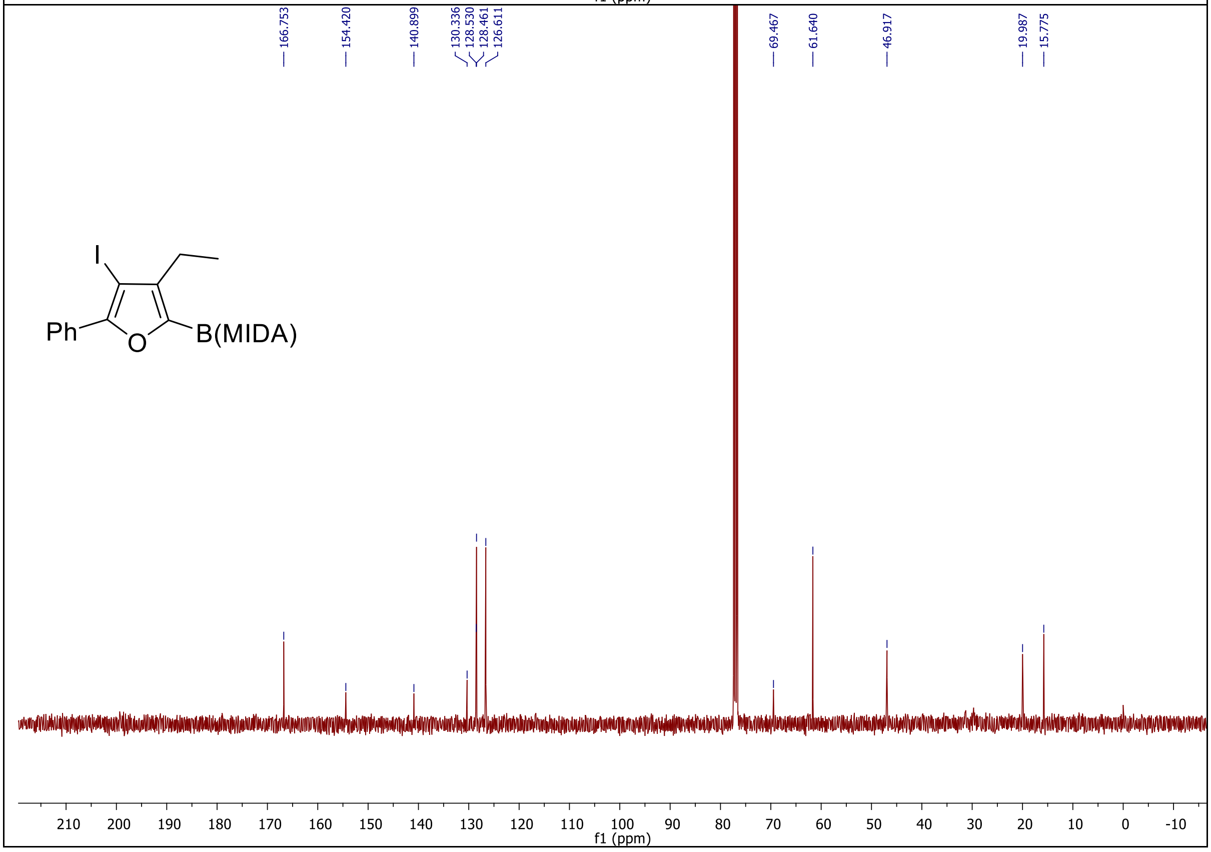

166.753
154.420
140.899
130.336
128.530
128.461
126.611
69.467
61.640
46.917
19.987
15.775
I
Ph
O
B(MIDA)
210 200 190 180 170 160 150 140 130 120 110 100 90 80 70 60 50 40 30 20 10 0 -10
f1 (ppm)

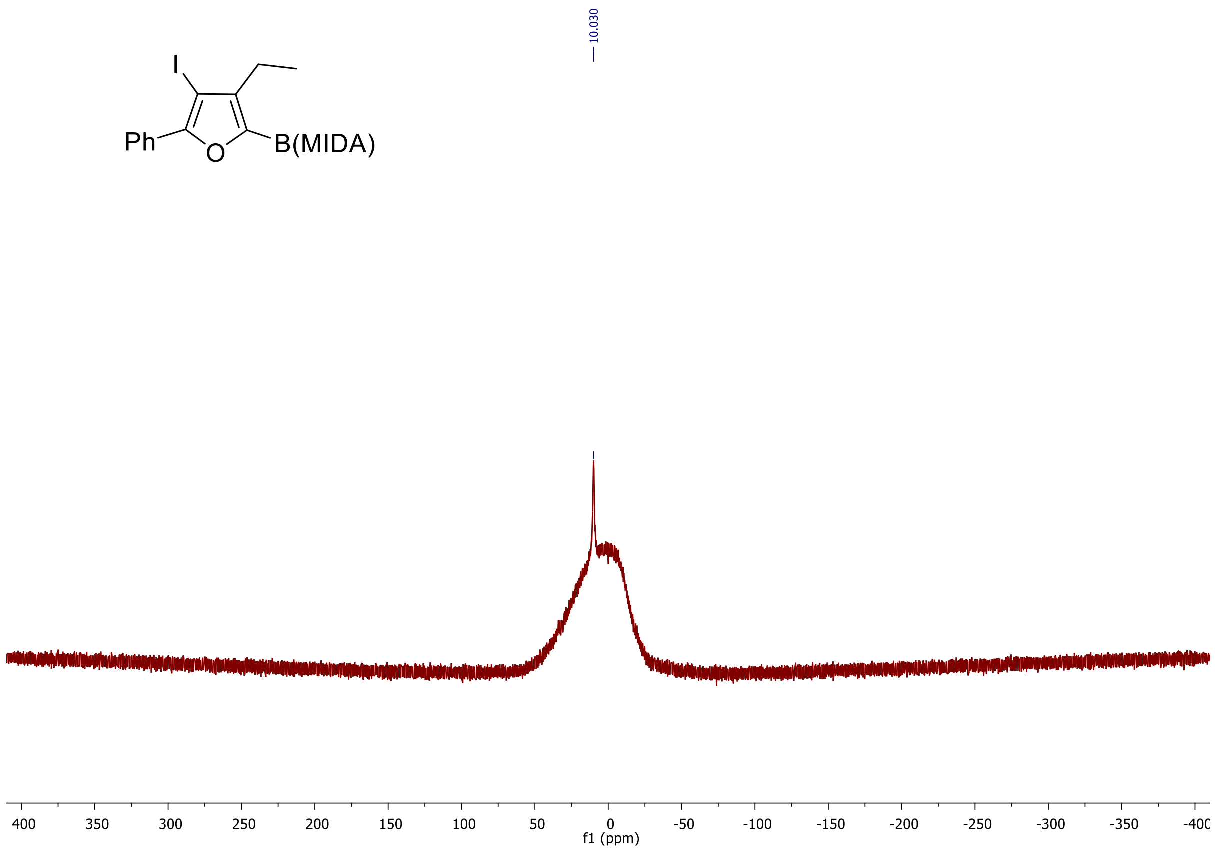

I
Ph
O
B(MIDA)
10.030
400
350
300
250
200
150
100
50
0
-50
-100
-150
-200
-250
-300
-350
-400
f1 (ppm)

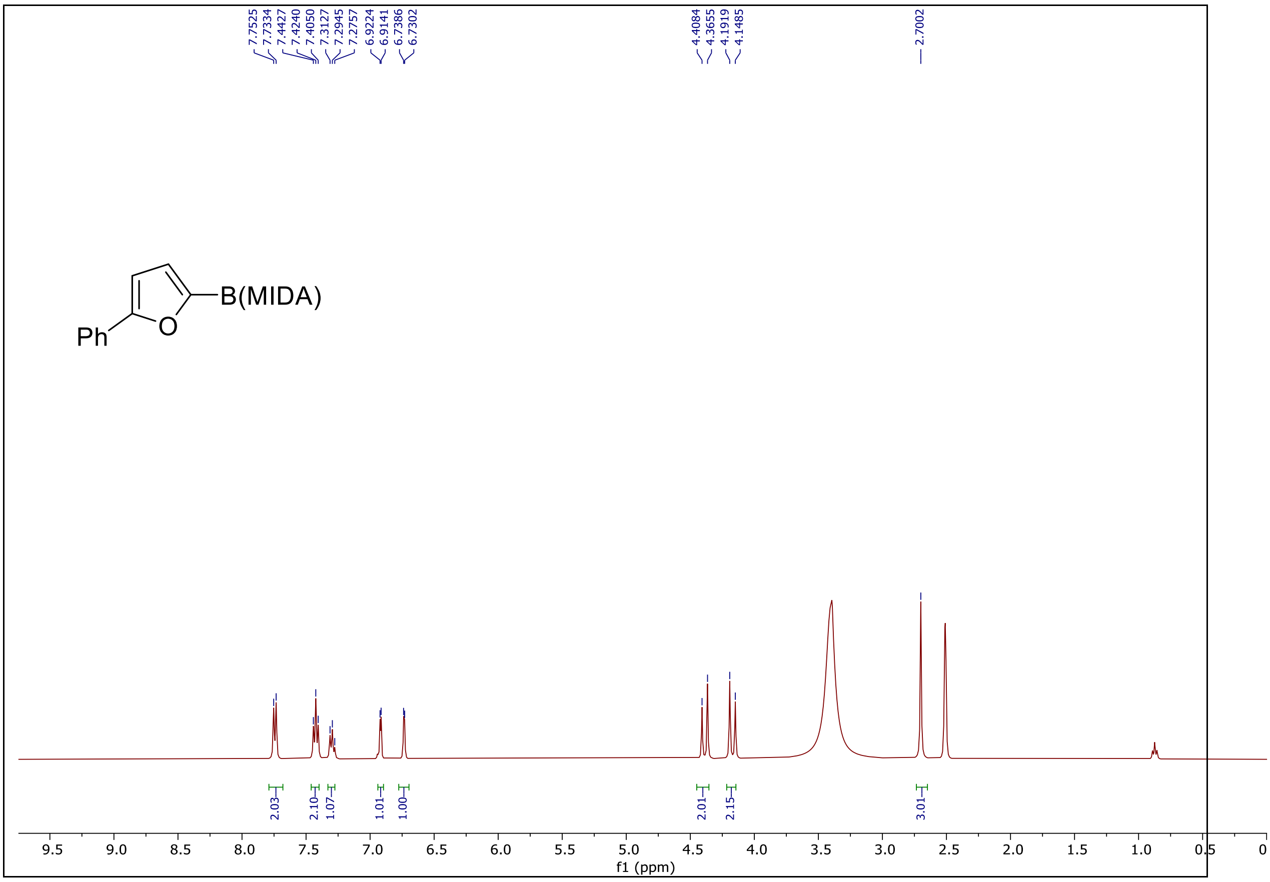

7.7525
7.7334
7.4427
7.4240
7.4050
7.3127
7.2945
7.2757
6.9224
6.9141
6.7386
6.7302
4.4084
4.3655
4.1919
4.1485
2.7002
Ph
O
B(MIDA)
2.03
2.10
1.07
1.01
1.00
2.01
2.15
3.01
9.5
9.0
8.5
8.0
7.5
7.0
6.5
6.0
5.5
5.0
4.5
4.0
3.5
3.0
2.5
2.0
1.5
1.0
0.5
0
f1 (ppm)

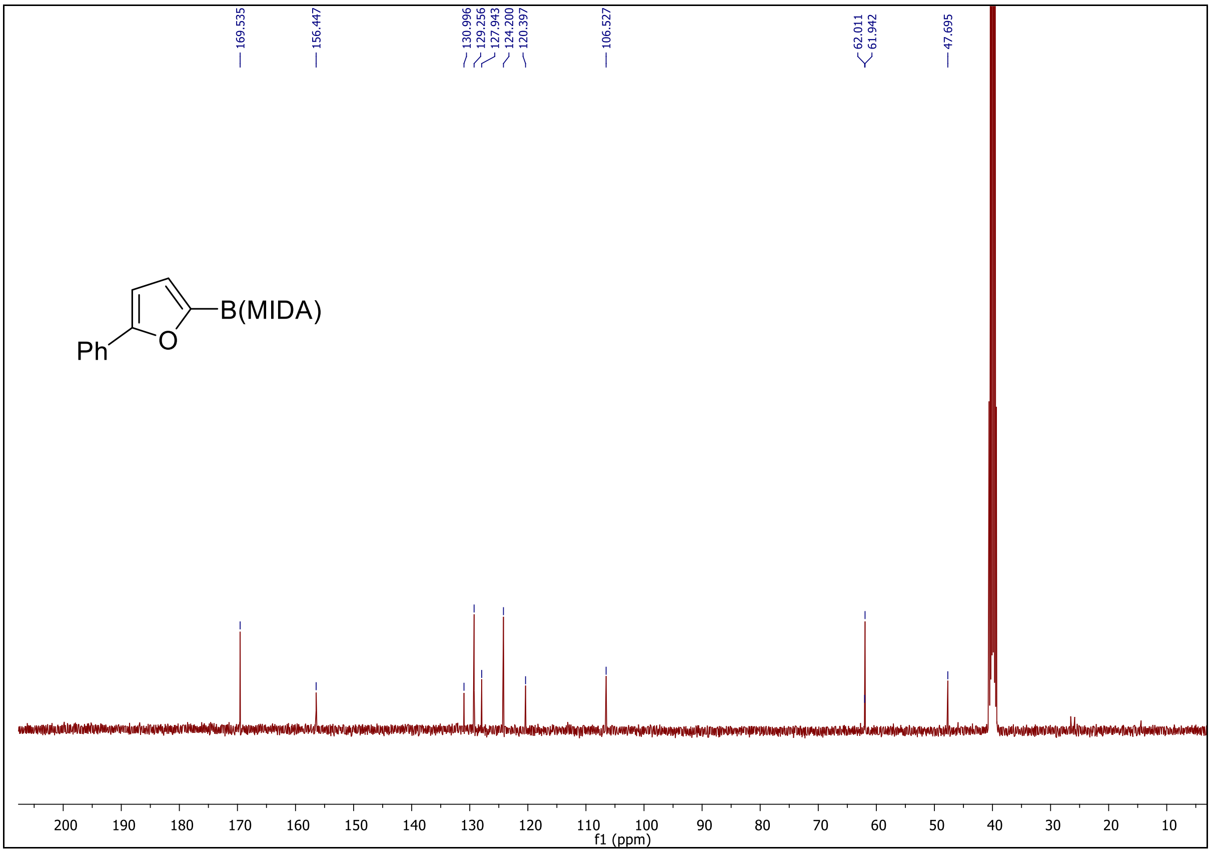
169.535
156.447
130.996
129.256
127.943
124.200
120.397
106.527
62.011
61.942
47.695
B(MIDA)
Ph
O
f1 (ppm)

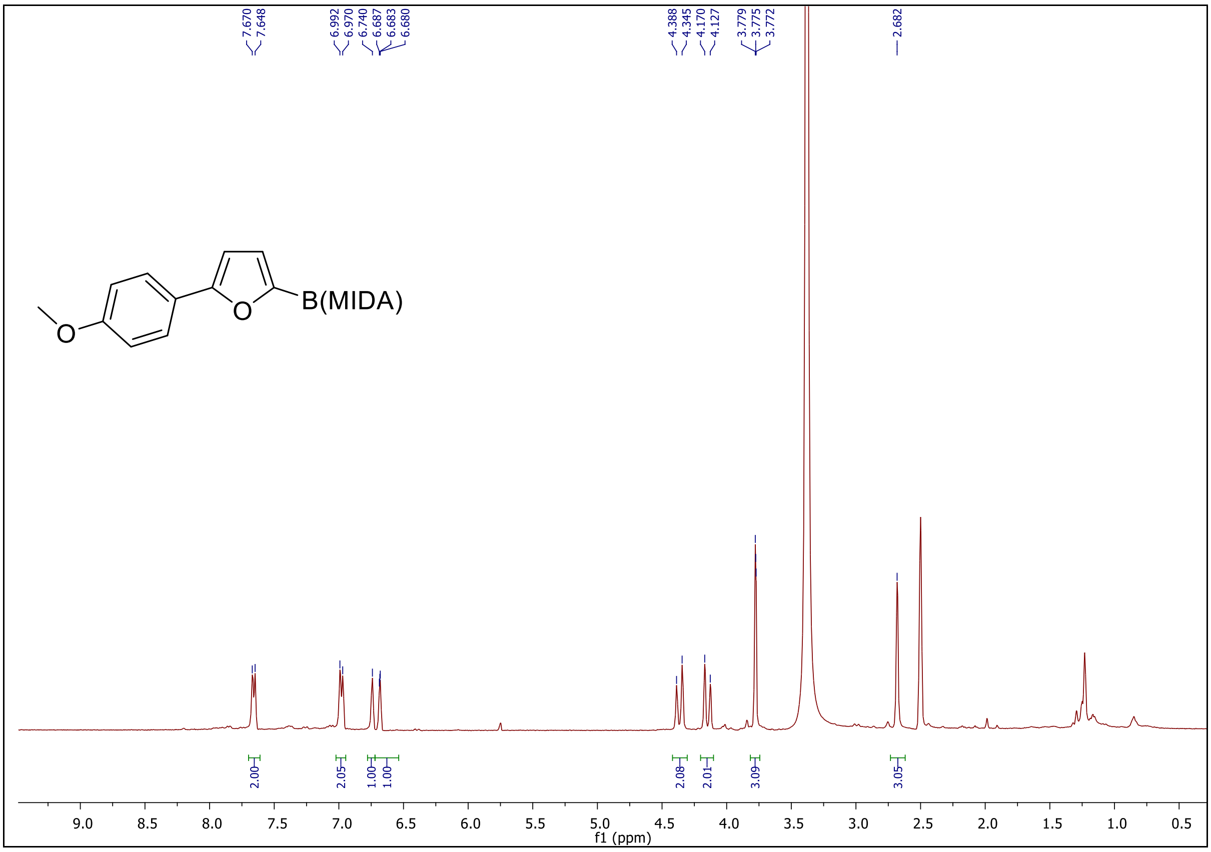
7.670
7.648
6.992
6.970
6.740
6.687
6.683
6.680
4.388
4.345
4.170
4.127
3.779
3.775
3.772
2.682
B(MIDA)
O
O
2.00
2.05
1.00
1.00
2.08
2.01
3.09
3.05
f1 (ppm)

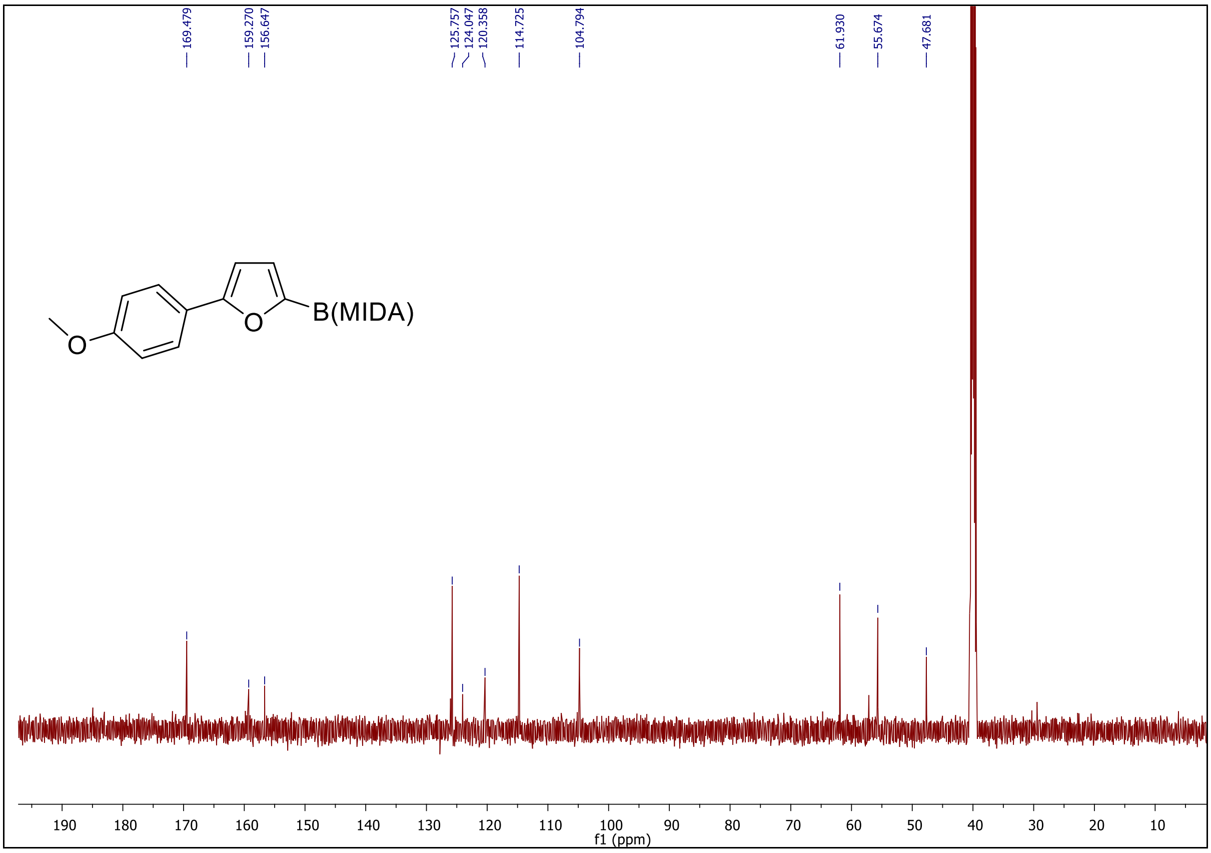

169.479
159.270
156.647
125.757
124.047
120.358
114.725
104.794
61.930
55.674
47.681
B(MIDA)
O
O
190
180
170
160
150
140
130
120
110
100
90
80
70
60
50
40
30
20
10
f1 (ppm)

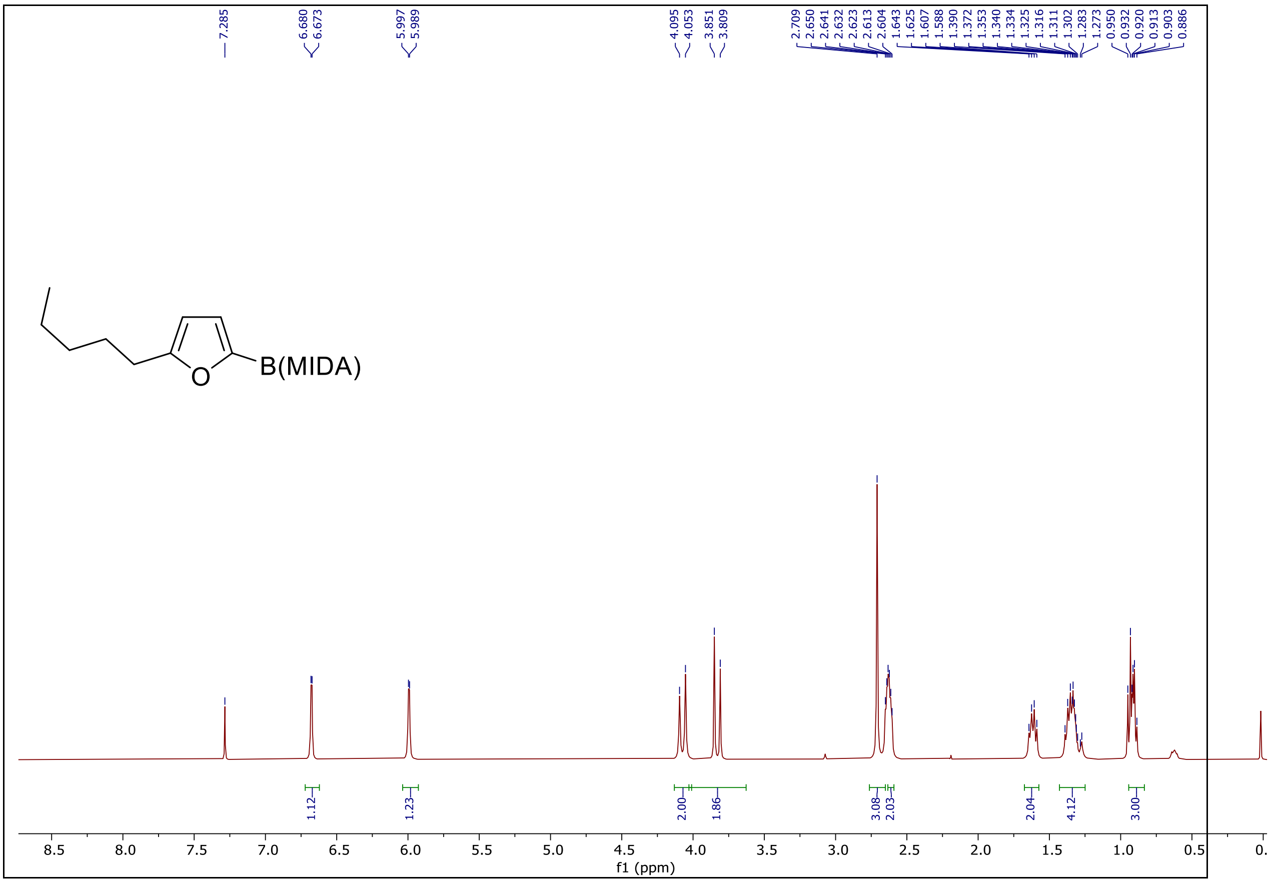

7.285
6.680
6.673
5.997
5.989
4.095
4.053
3.851
3.809
2.709
2.650
2.641
2.632
2.623
2.613
2.604
1.643
1.625
1.607
1.588
1.390
1.372
1.353
1.340
1.334
1.325
1.316
1.311
1.302
1.283
1.273
0.950
0.932
0.920
0.913
0.903
0.886
B(MIDA)
O
1.12
1.23
2.00
1.86
3.08
2.03
2.04
4.12
3.00
8.5
8.0
7.5
7.0
6.5
6.0
5.5
5.0
4.5
4.0
3.5
3.0
2.5
2.0
1.5
1.0
0.5
f1 (ppm)

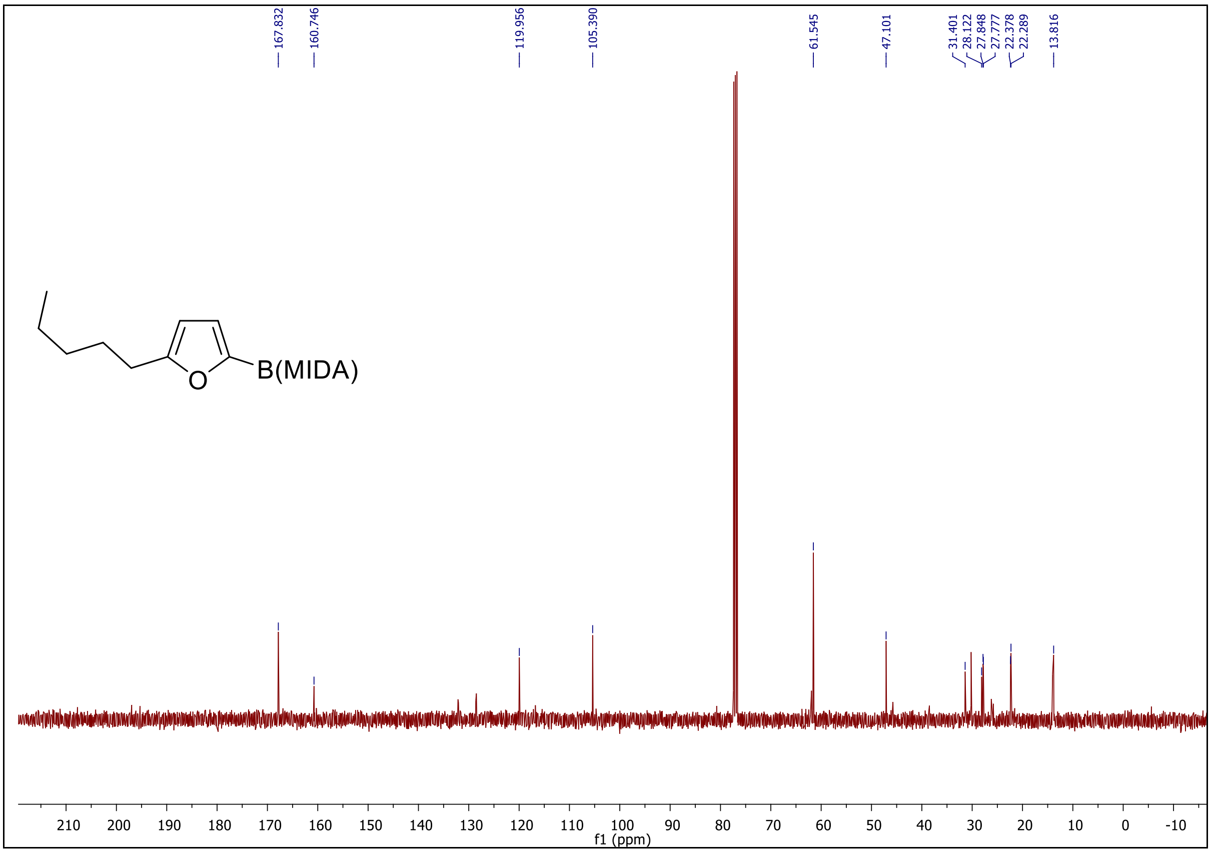
167.832
160.746
119.956
105.390
61.545
47.101
31.401
28.122
27.848
27.777
22.378
22.289
13.816
B(MIDA)
O
f1 (ppm)

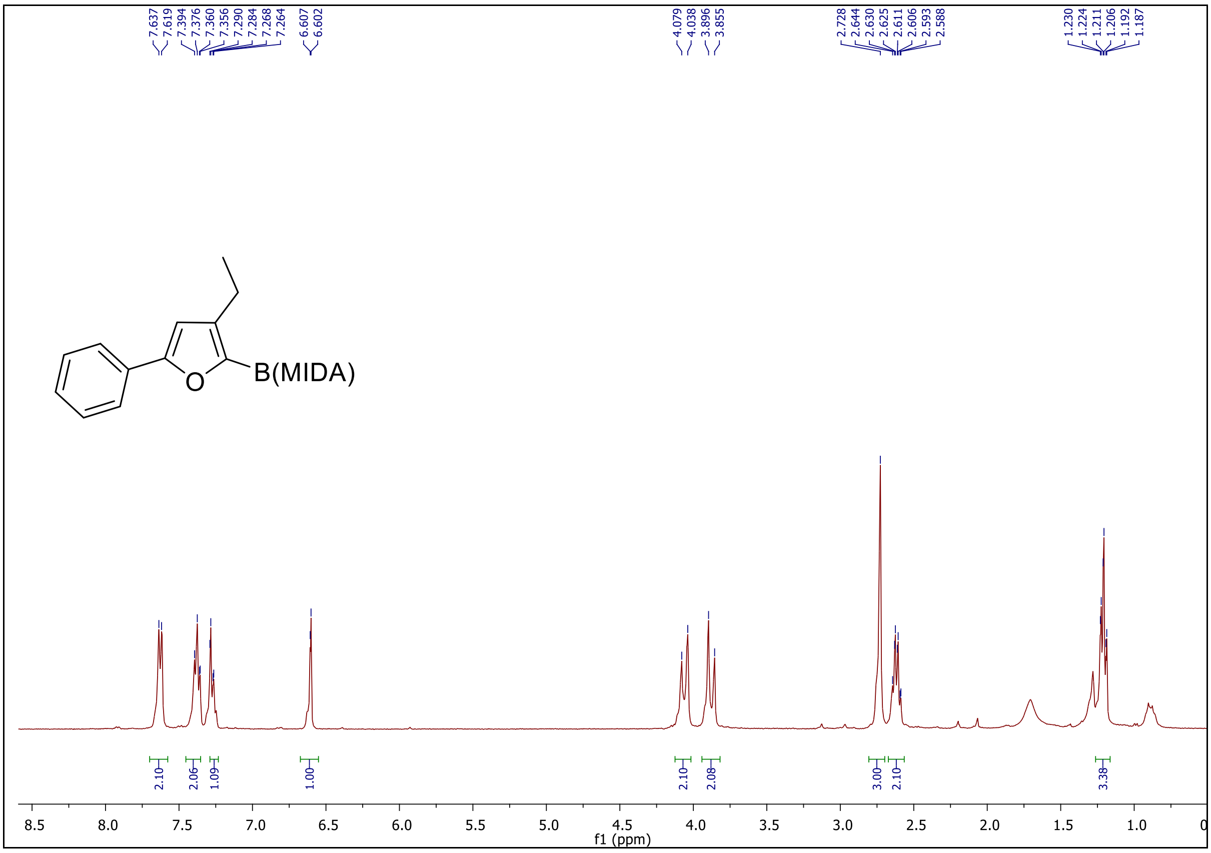
7.637
7.619
7.394
7.376
7.360
7.356
7.290
7.284
7.268
7.264
6.607
6.602
4.079
4.038
3.896
3.855
2.728
2.644
2.630
2.625
2.611
2.606
2.593
2.588
1.230
1.224
1.211
1.206
1.192
1.187
B(MIDA)
O
2.10
2.06
1.09
1.00
2.10
2.08
3.00
2.10
3.38
f1 (ppm)

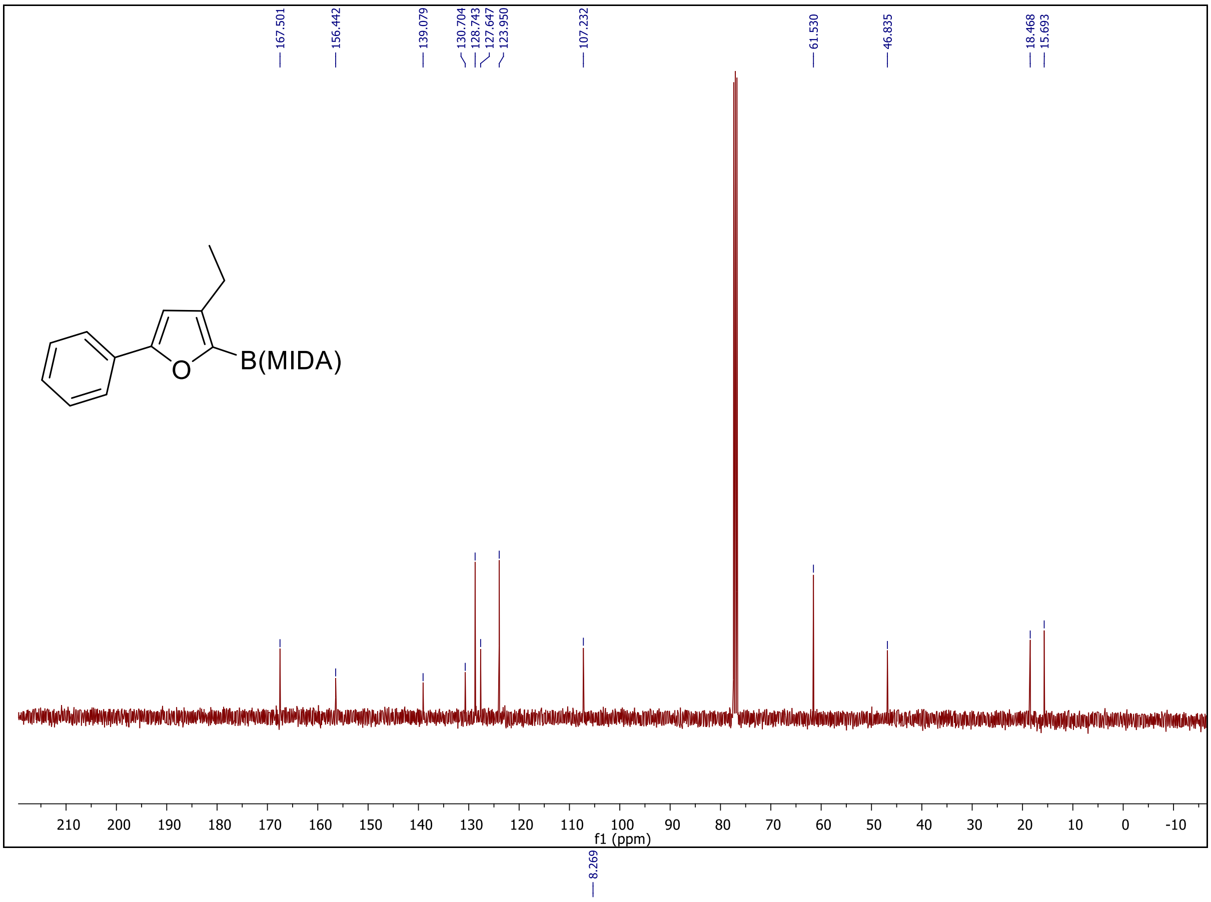
167.501
156.442
139.079
130.704
128.743
127.647
123.950
107.232
61.530
46.835
18.468
15.693
B(MIDA)
O
f1 (ppm)
8.269

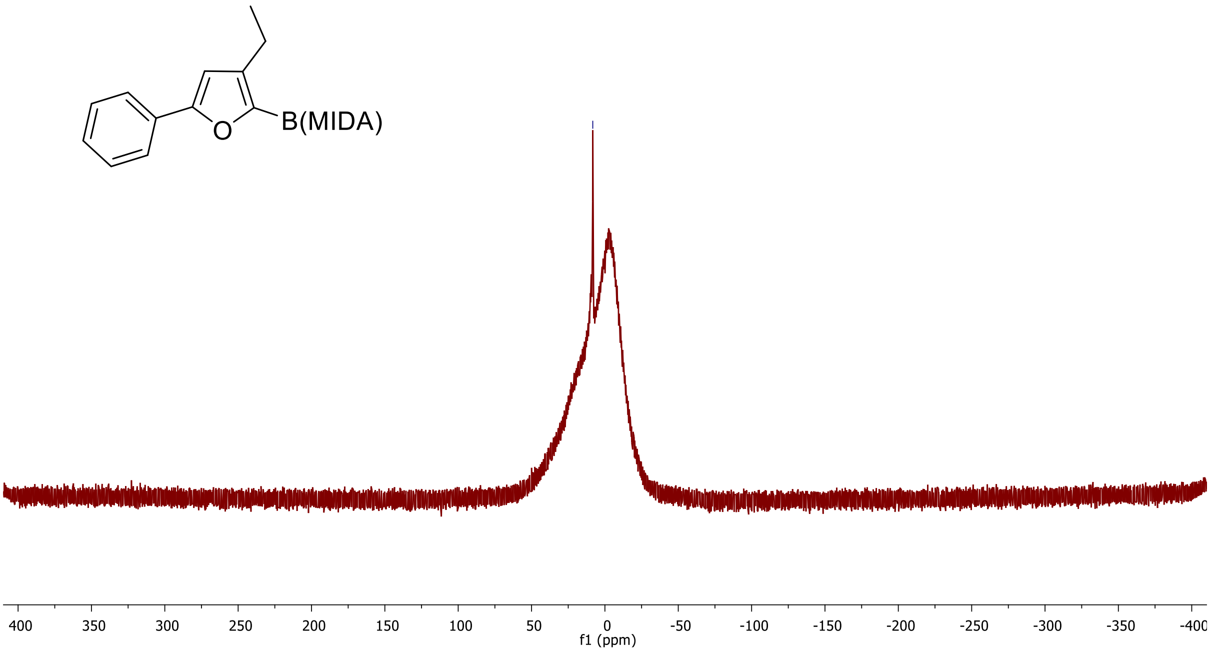
B(MIDA)
O
f1 (ppm)

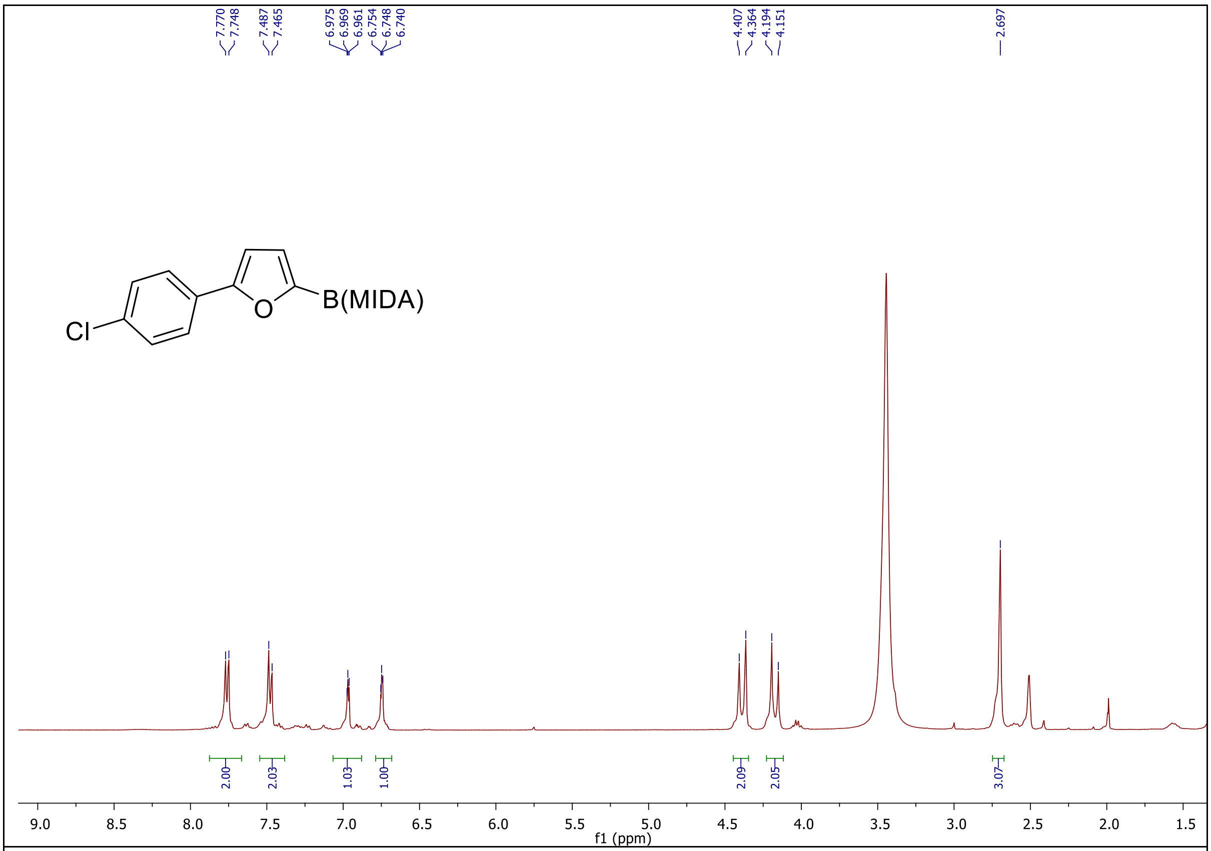
7.770
7.748
7.487
7.465
6.975
6.969
6.961
6.754
6.748
6.740
4.407
4.364
4.194
4.151
2.697
Cl
O
B(MIDA)
2.00
2.03
1.03
1.00
2.09
2.05
3.07
9.0
8.5
8.0
7.5
7.0
6.5
6.0
5.5
5.0
4.5
4.0
3.5
3.0
2.5
2.0
1.5
f1 (ppm)

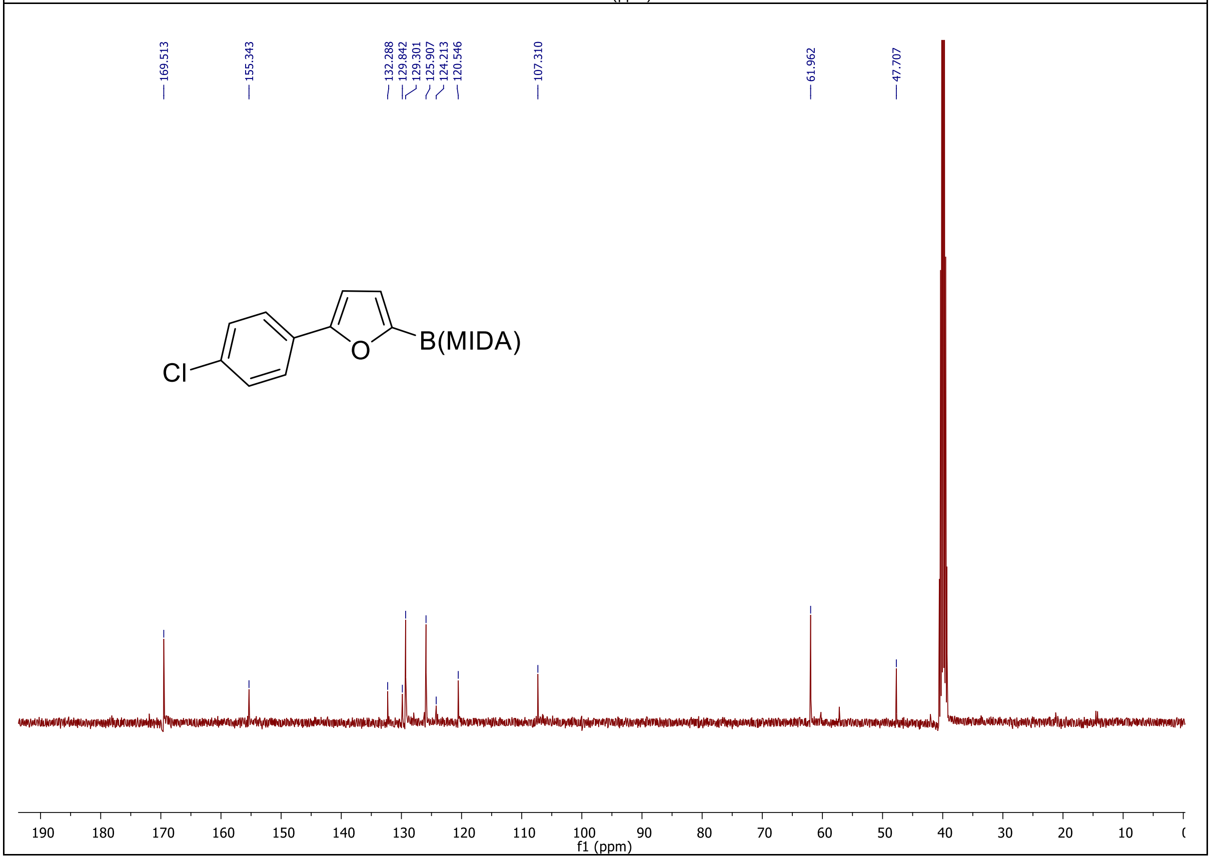
169.513
155.343
132.288
129.842
129.301
125.907
124.213
120.546
107.310
61.962
47.707
Cl
O
B(MIDA)
190
180
170
160
150
140
130
120
110
100
90
80
70
60
50
40
30
20
10
f1 (ppm)

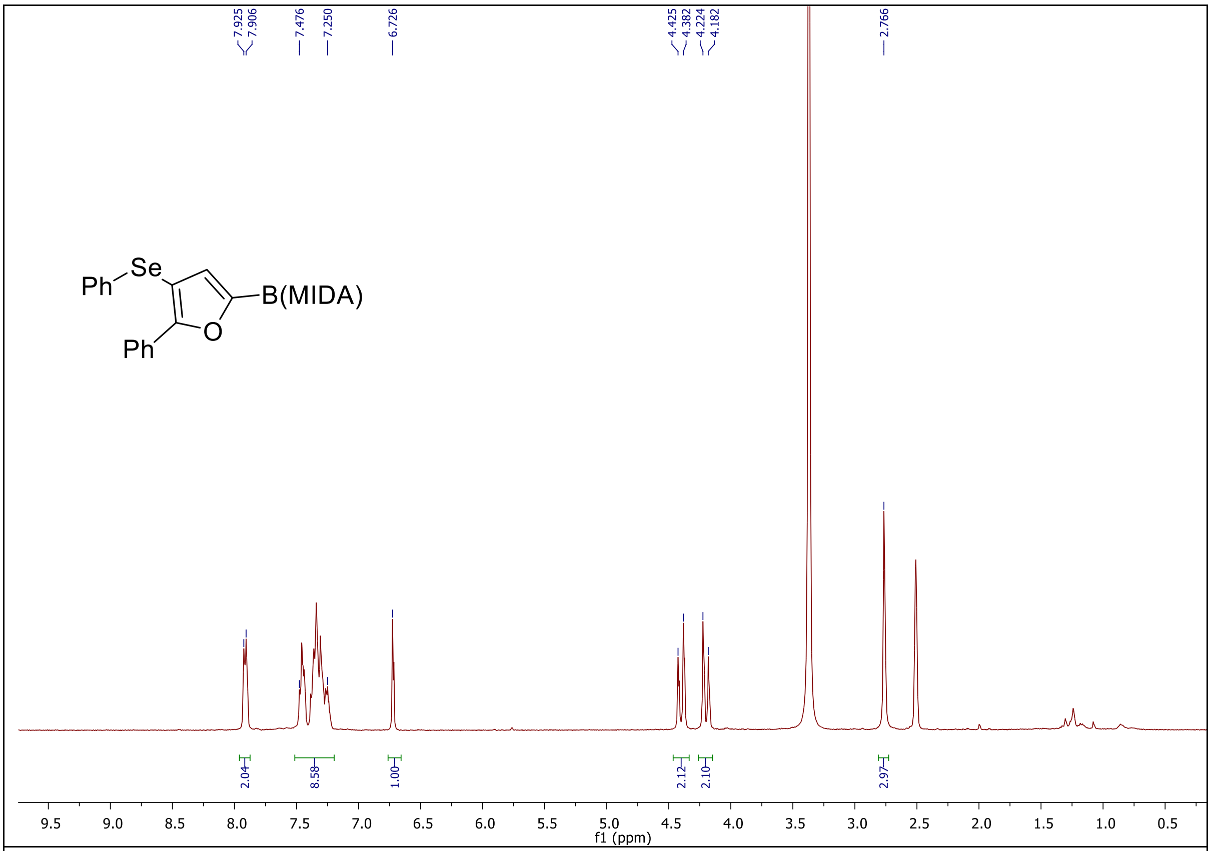
7.925
7.906
7.476
7.250
6.726
4.425
4.382
4.224
4.182
2.766
Ph
Se
B(MIDA)
Ph
O
2.04
8.58
1.00
2.12
2.10
2.97
9.5
9.0
8.5
8.0
7.5
7.0
6.5
6.0
5.5
5.0
4.5
4.0
3.5
3.0
2.5
2.0
1.5
1.0
0.5
f1 (ppm)

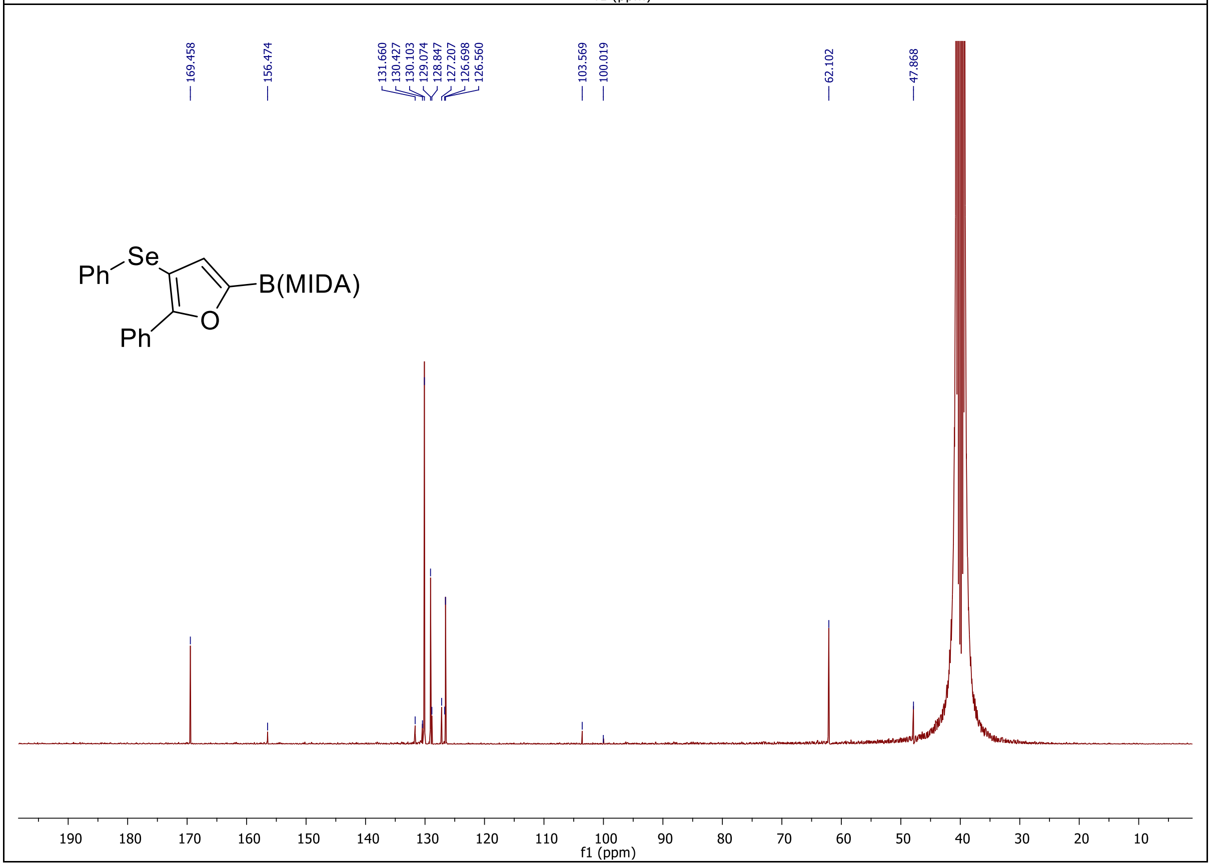
169.458
156.474
131.660
130.427
130.103
129.074
128.847
127.207
126.698
126.560
103.569
100.019
62.102
47.868
Ph
Se
B(MIDA)
Ph
O
190
180
170
160
150
140
130
120
110
100
90
80
70
60
50
40
30
20
10
f1 (ppm)

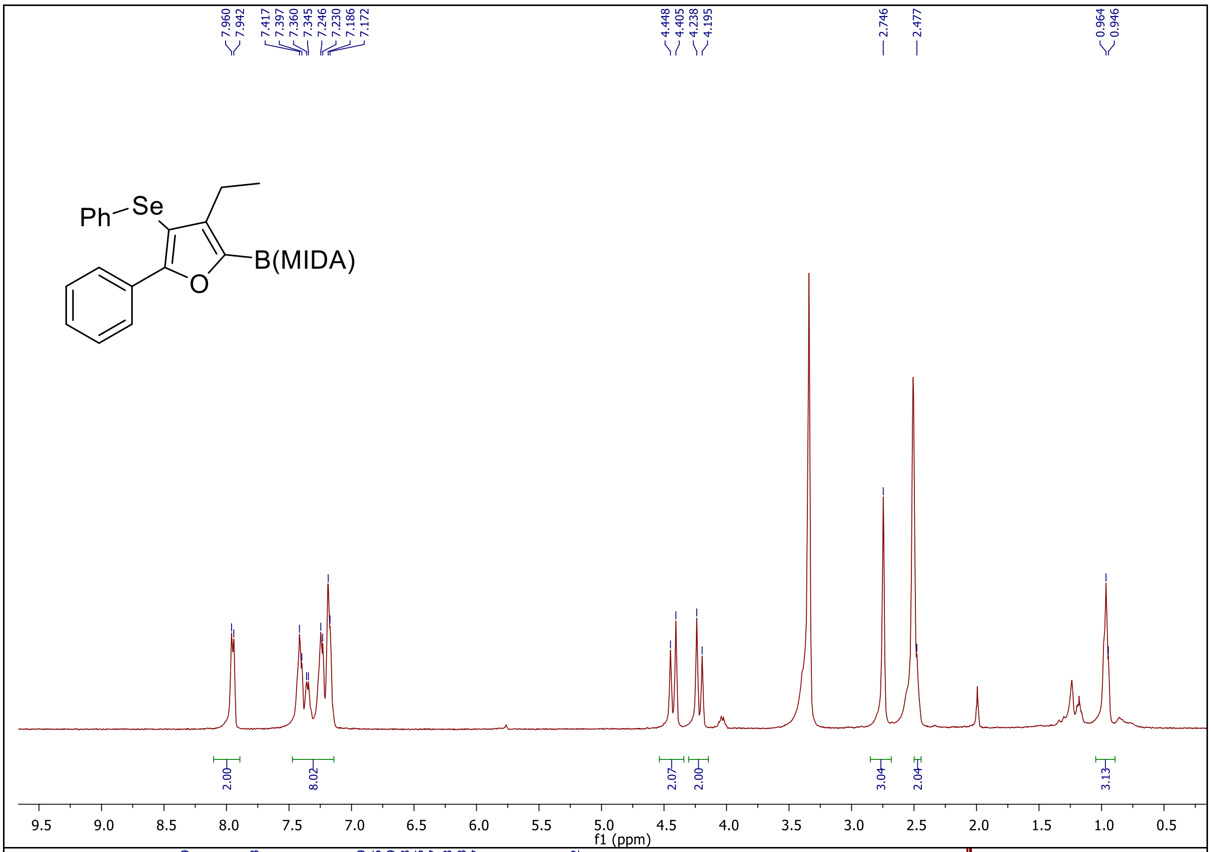

Ph
Se
B(MIDA)
O
7.960
7.942
7.417
7.397
7.360
7.345
7.246
7.230
7.186
7.172
4.448
4.405
4.238
4.195
2.746
2.477
0.964
0.946
2.00
8.02
2.07
2.00
3.04
2.04
3.13
f1 (ppm)

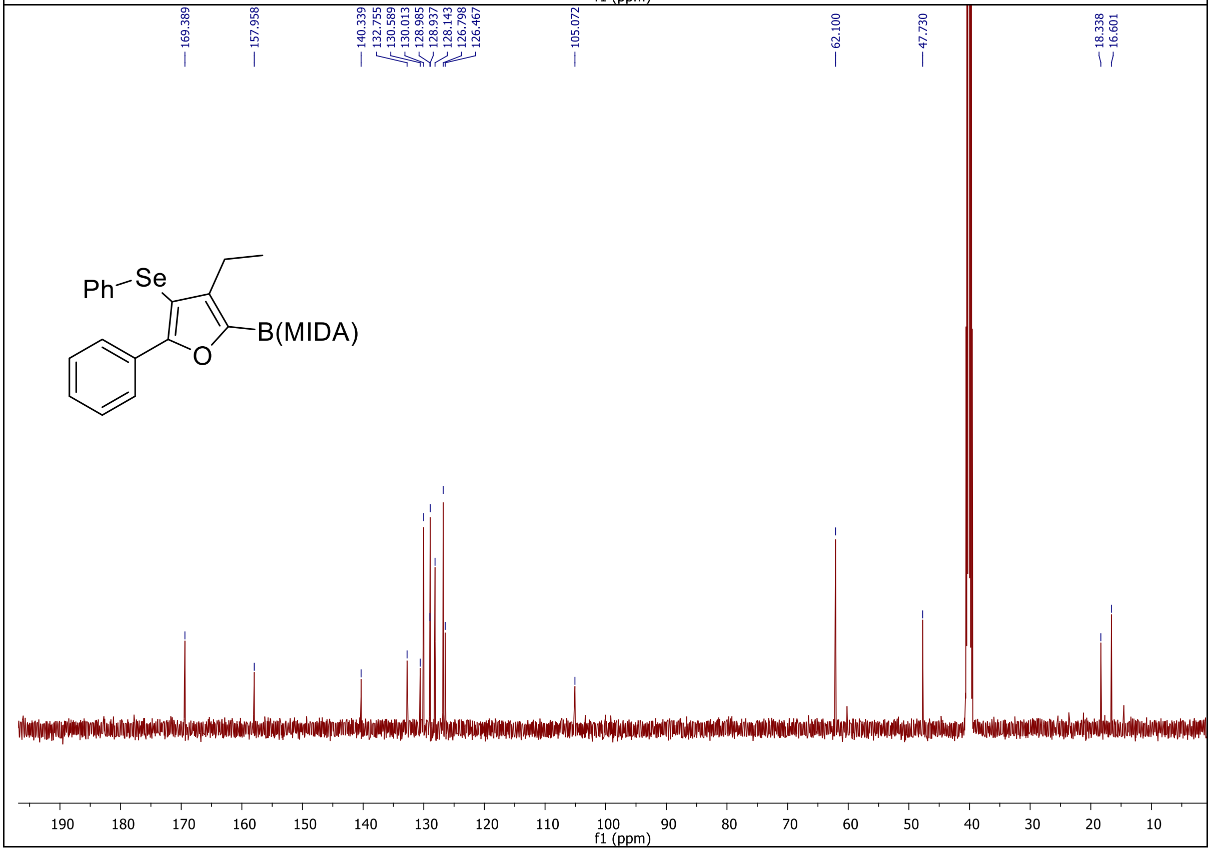

Ph
Se
B(MIDA)
O
169.389
157.958
140.339
132.755
130.589
130.013
128.985
128.937
128.143
126.798
126.467
105.072
62.100
47.730
18.338
16.601
f1 (ppm)

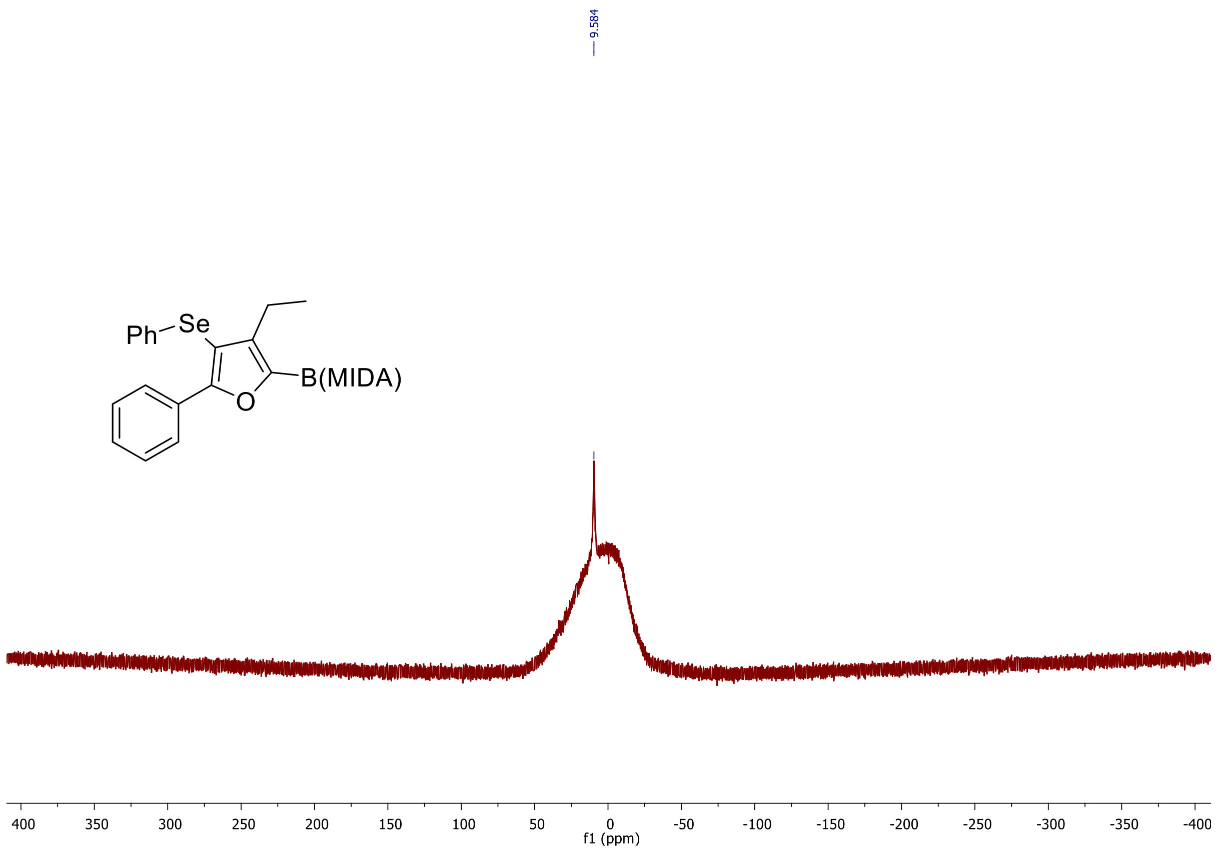
9.584
Ph
Se
B(MIDA)
O
f1 (ppm)

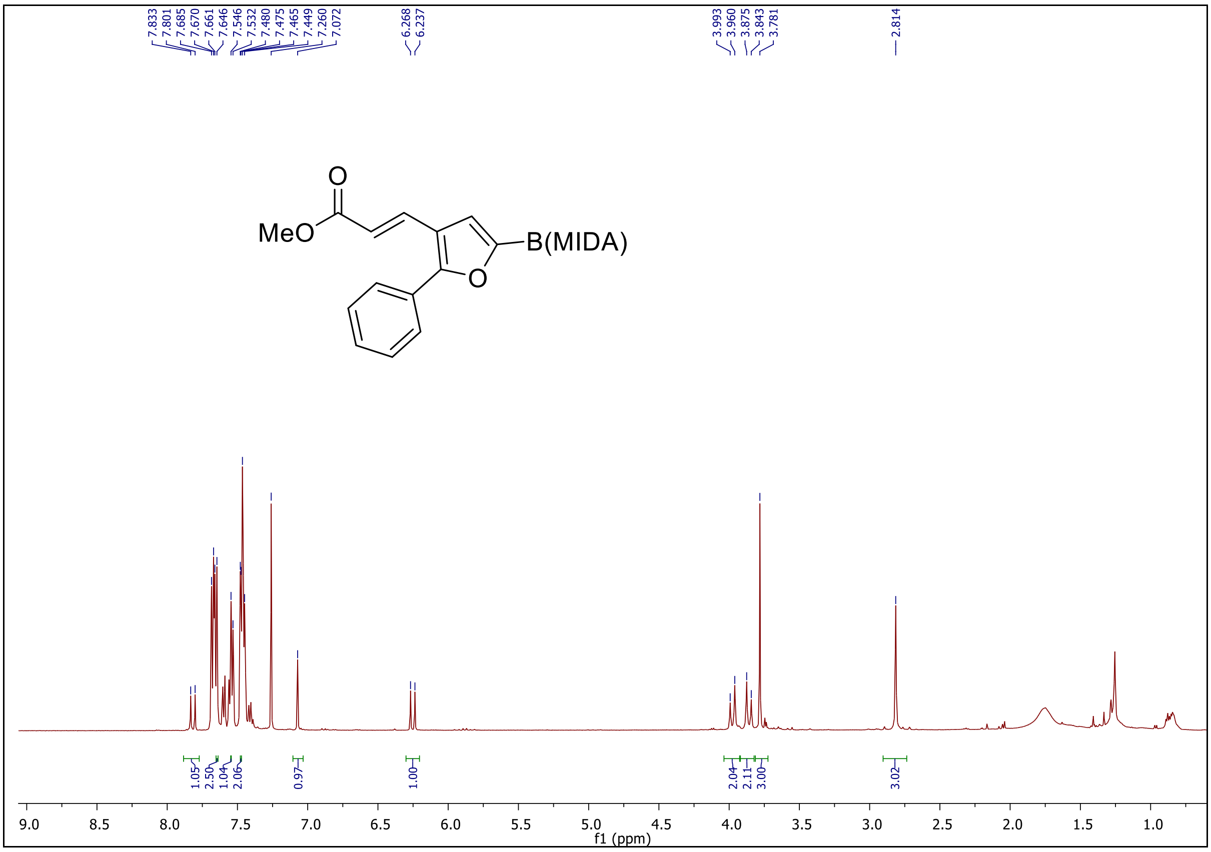
7.833
7.801
7.685
7.670
7.661
7.646
7.546
7.532
7.480
7.475
7.465
7.449
7.260
7.072
6.268
6.237
3.993
3.960
3.875
3.843
3.781
2.814
MeO
O
B(MIDA)
1.05
2.50
1.04
2.06
0.97
1.00
2.04
2.11
3.00
3.02
f1 (ppm)

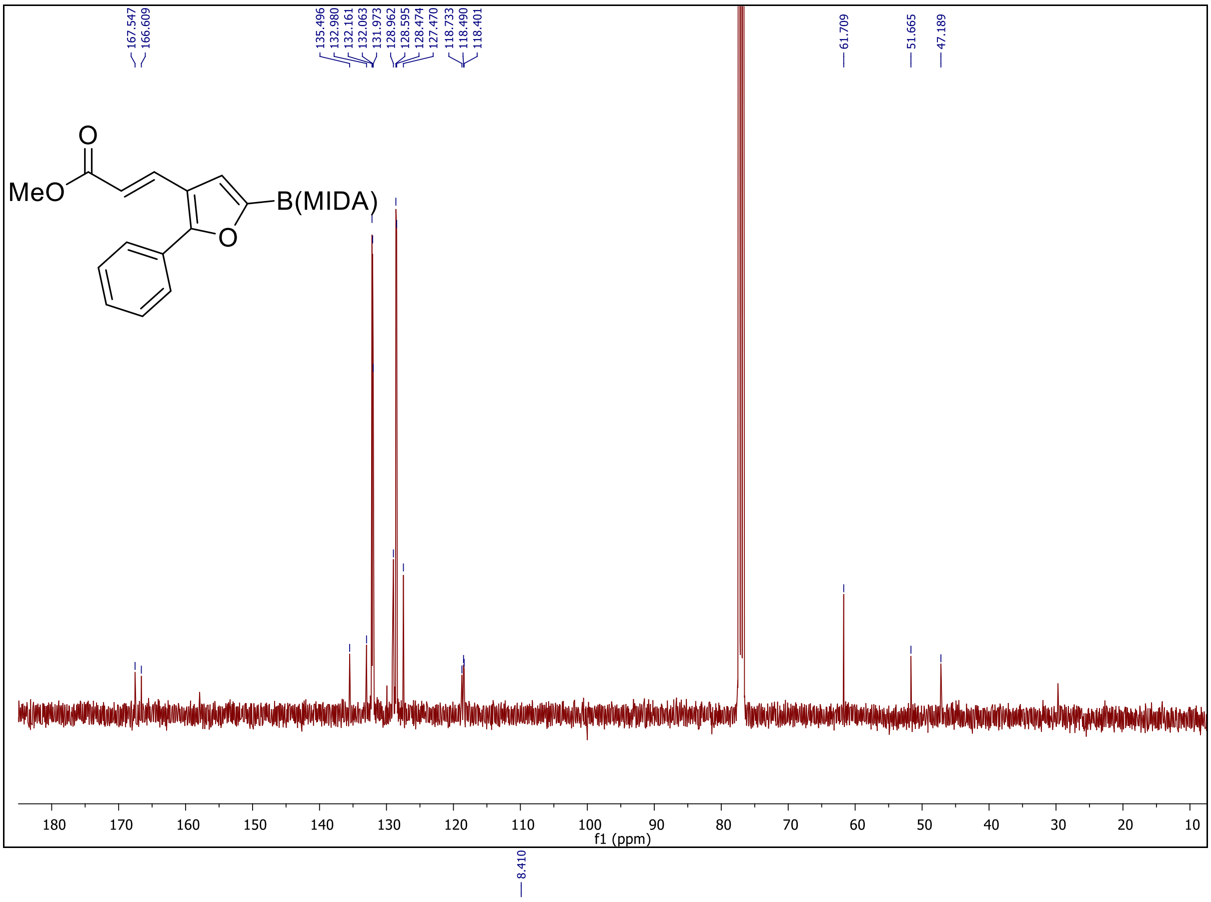

167.547
166.609
135.496
132.980
132.161
132.063
131.973
128.962
128.595
128.474
127.470
118.733
118.490
118.401
61.709
51.665
47.189
MeO
O
B(MIDA)
O
f1 (ppm)
8.410

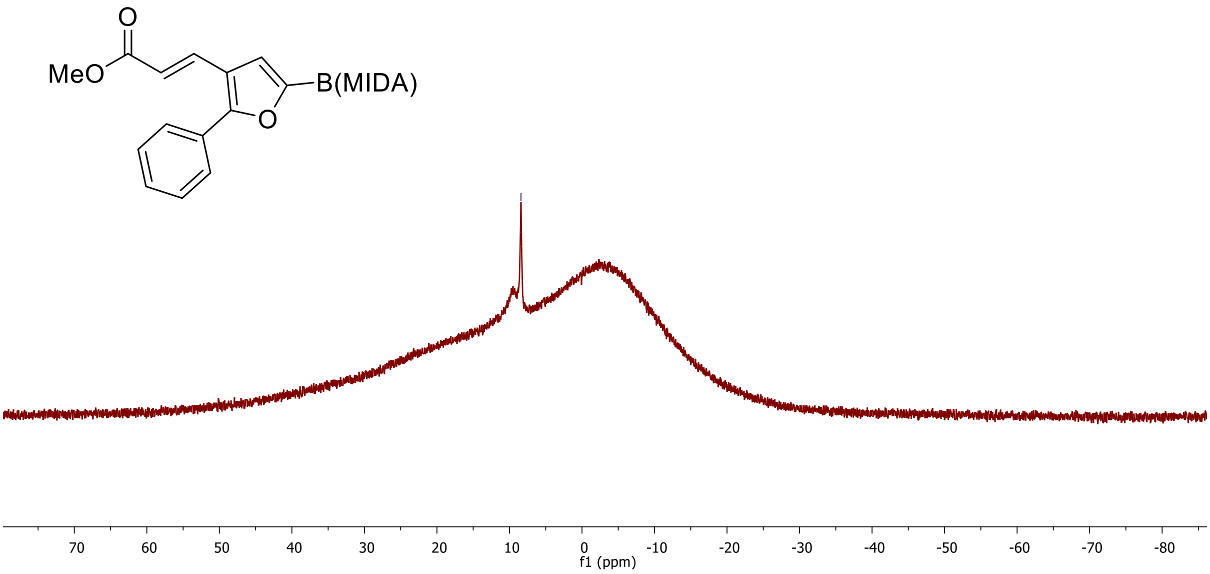

MeO
O
B(MIDA)
O
f1 (ppm)

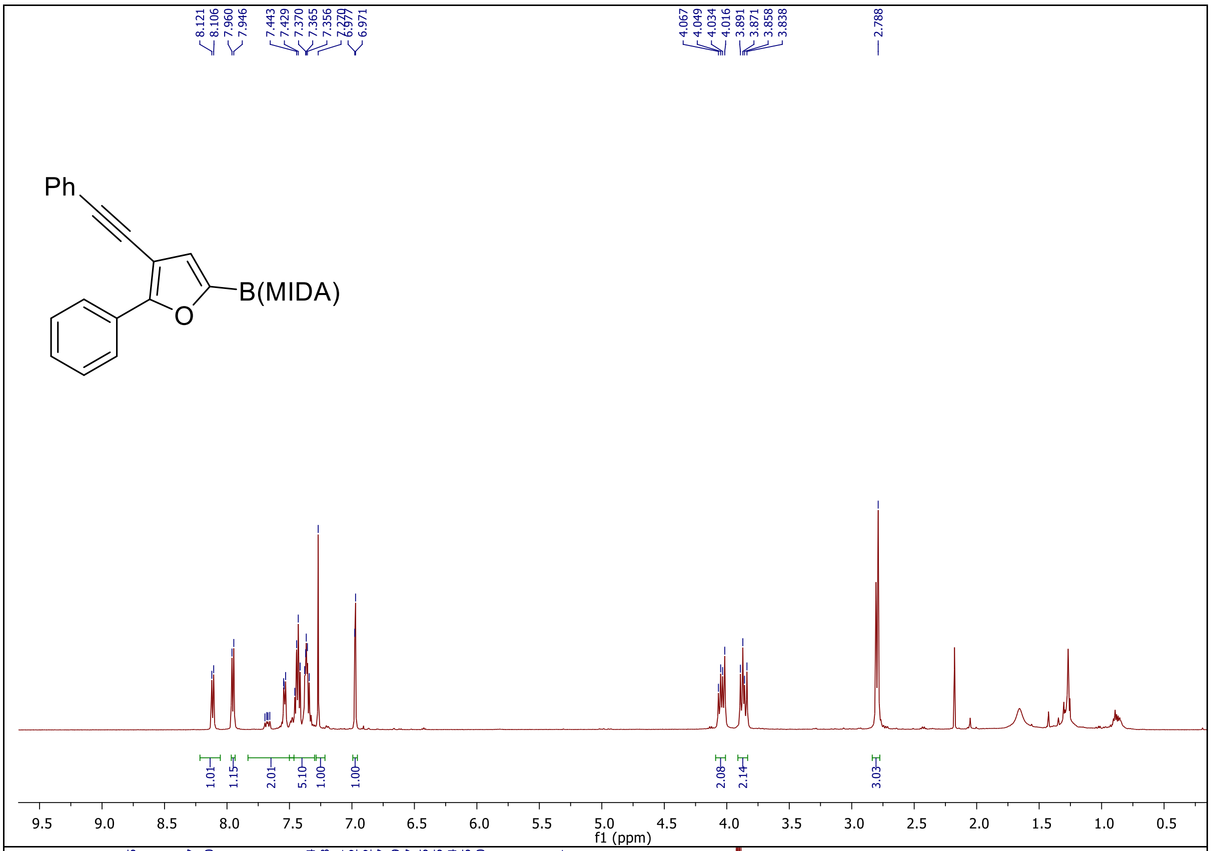

8.121
8.106
7.960
7.946
7.443
7.429
7.370
7.365
7.356
7.270
6.977
6.971
4.067
4.049
4.034
4.016
3.891
3.871
3.858
3.838
2.788
Ph
B(MIDA)
O
1.01
1.15
2.01
5.10
1.00
1.00
2.08
2.14
3.03
f1 (ppm)

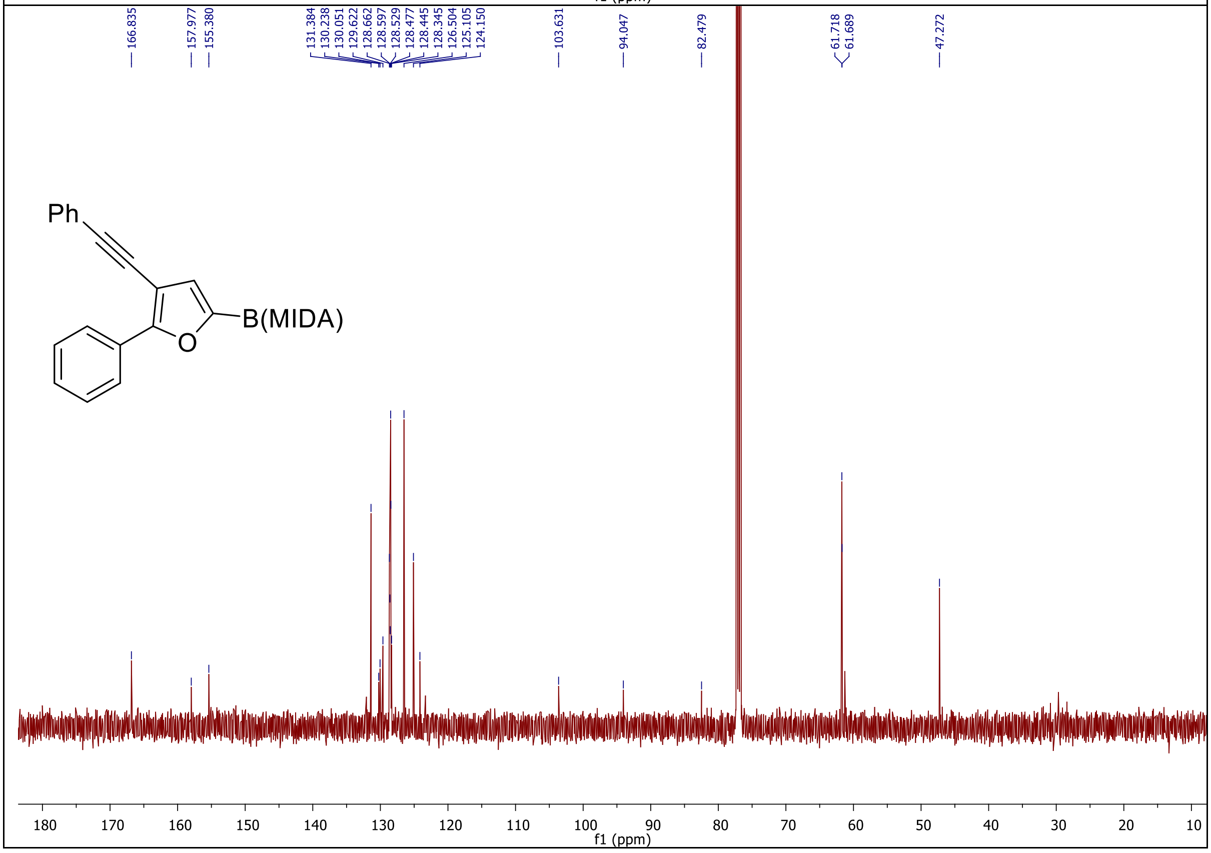

166.835
157.977
155.380
131.384
130.238
130.051
129.622
128.662
128.597
128.529
128.477
128.445
128.345
126.504
125.105
124.150
103.631
94.047
82.479
61.718
61.689
47.272
Ph
B(MIDA)
O
f1 (ppm)

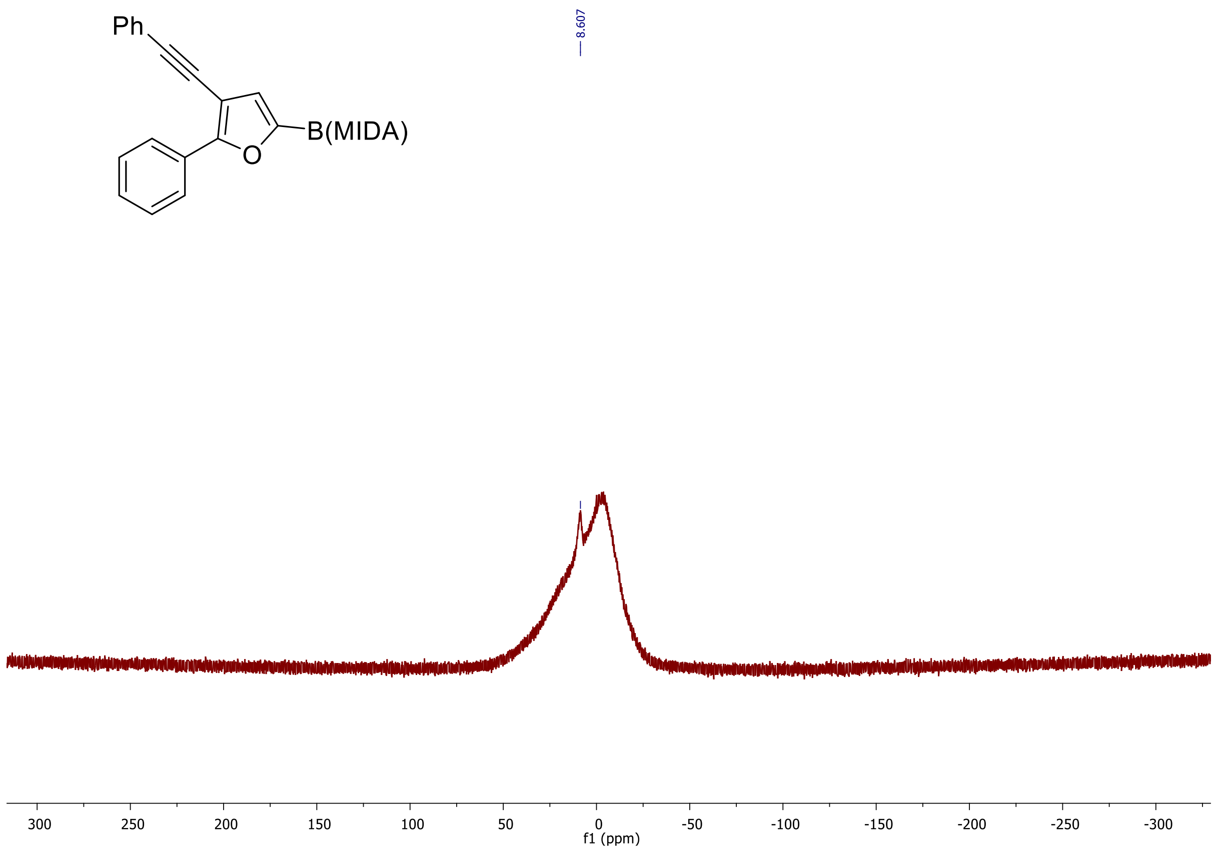
Ph
B(MIDA)
O
8.607
300
250
200
150
100
50
0
-50
-100
-150
-200
-250
-300
f1 (ppm)

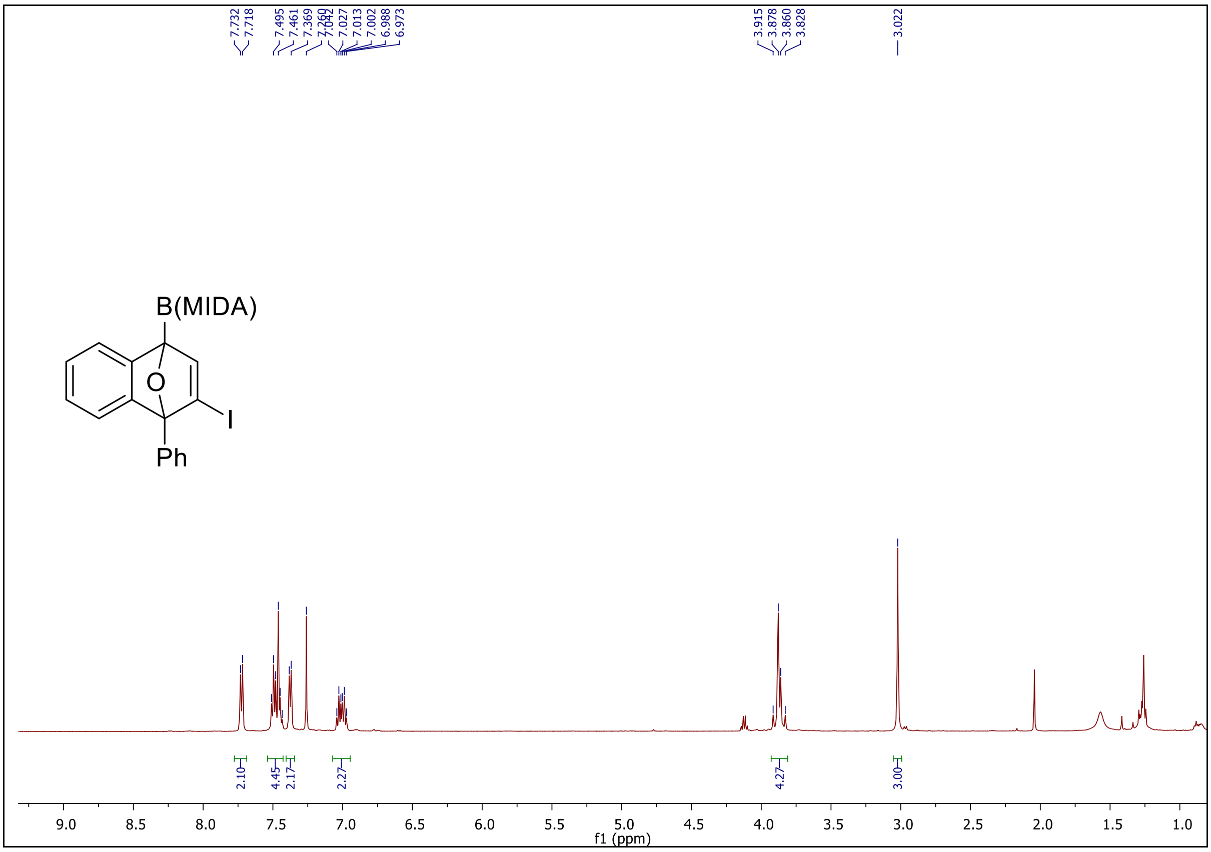
7.732
7.718
7.495
7.461
7.369
7.260
7.042
7.027
7.013
7.002
6.988
6.973
3.915
3.878
3.860
3.828
3.022
B(MIDA)
O
I
Ph
2.10
4.45
2.17
2.27
4.27
3.00
9.0
8.5
8.0
7.5
7.0
6.5
6.0
5.5
5.0
4.5
4.0
3.5
3.0
2.5
2.0
1.5
1.0
f1 (ppm)

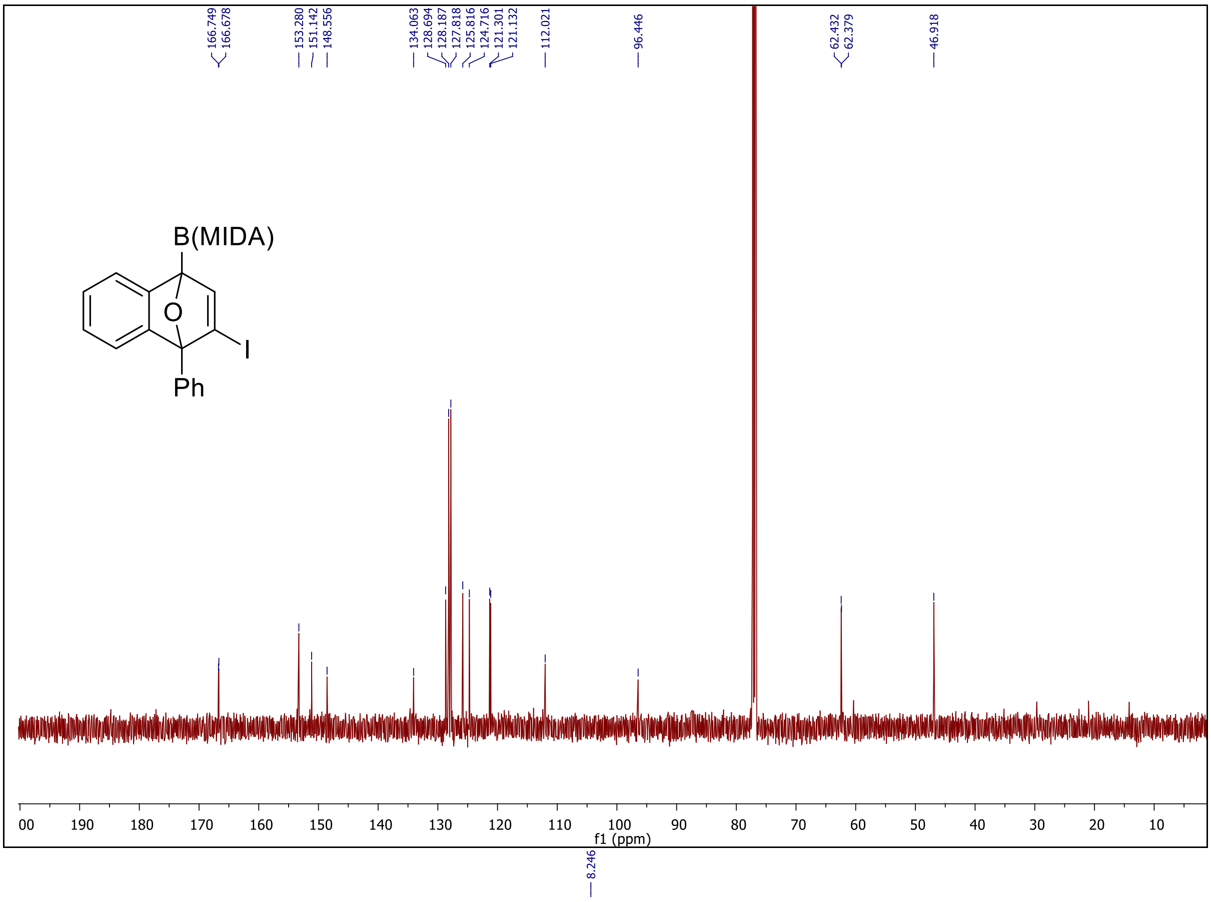
166.749
166.678
153.280
151.142
148.556
134.063
128.694
128.187
127.818
125.816
124.716
121.301
121.132
112.021
96.446
62.432
62.379
46.918
B(MIDA)
O
I
Ph
f1 (ppm)

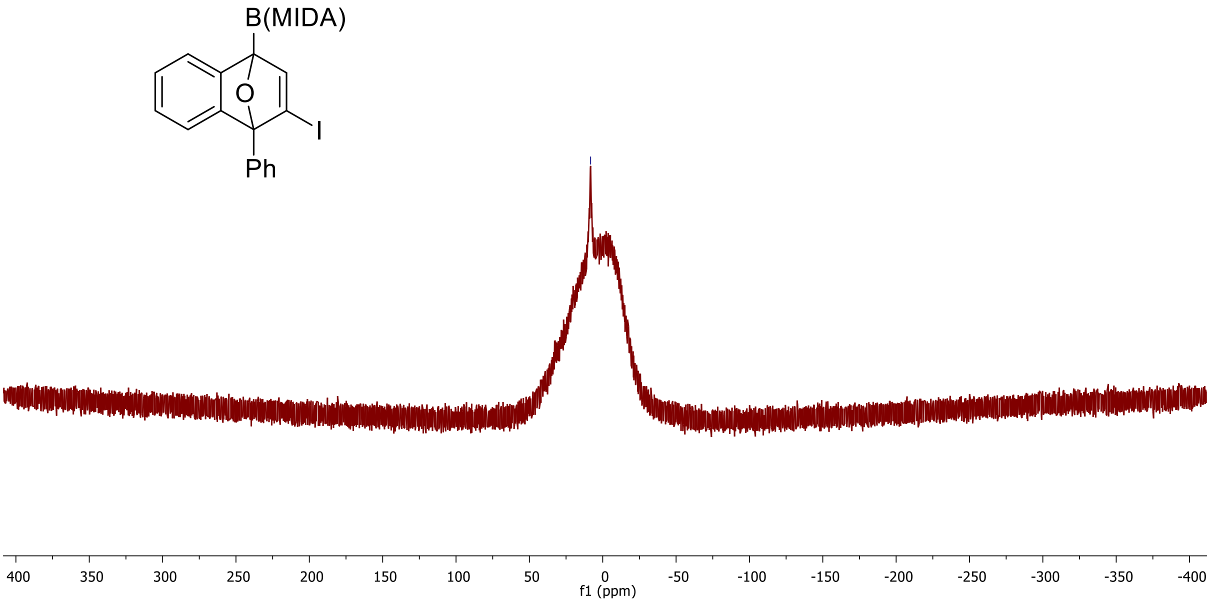
8.246
B(MIDA)
O
I
Ph
f1 (ppm)

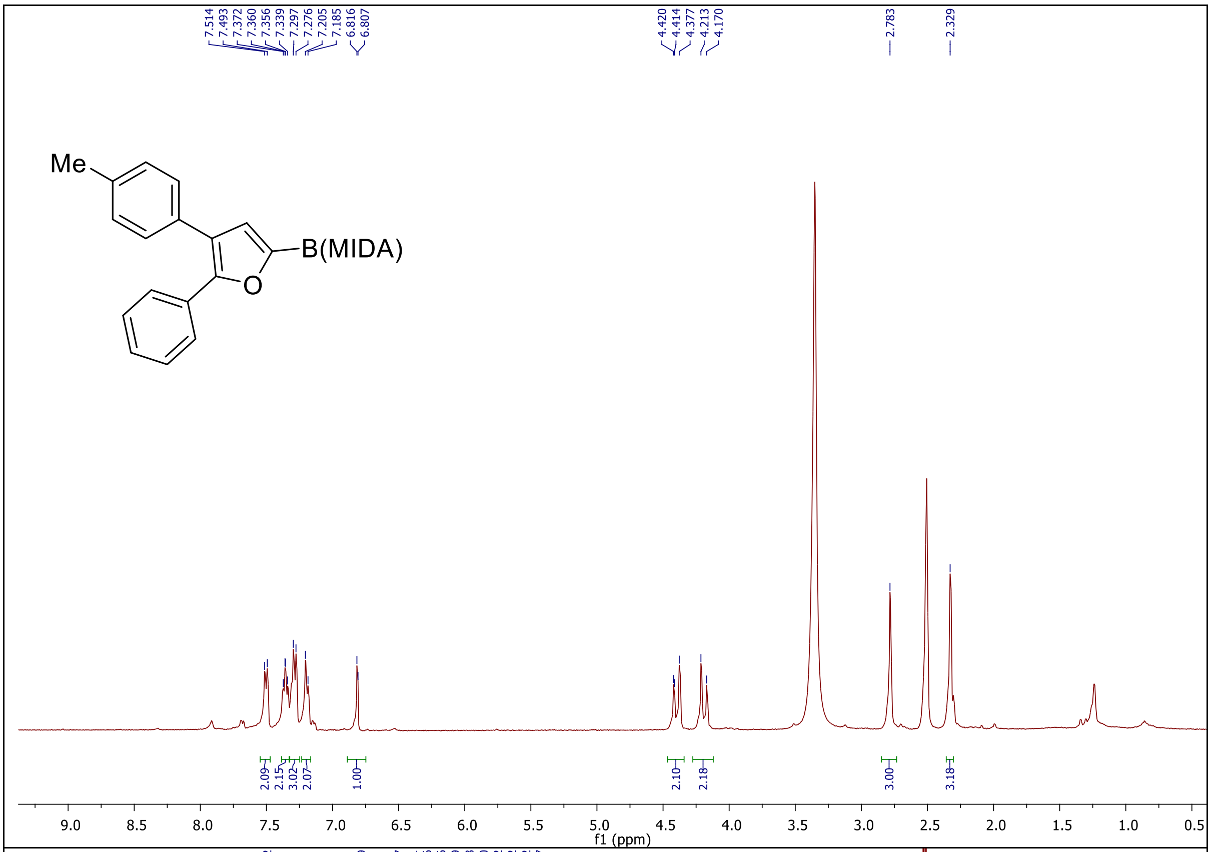
7.514
7.493
7.372
7.360
7.356
7.339
7.297
7.276
7.205
7.185
6.816
6.807
4.420
4.414
4.377
4.213
4.170
2.783
2.329
Me
B(MIDA)
O
2.09
2.15
3.02
2.07
1.00
2.10
2.18
3.00
3.18
f1 (ppm)

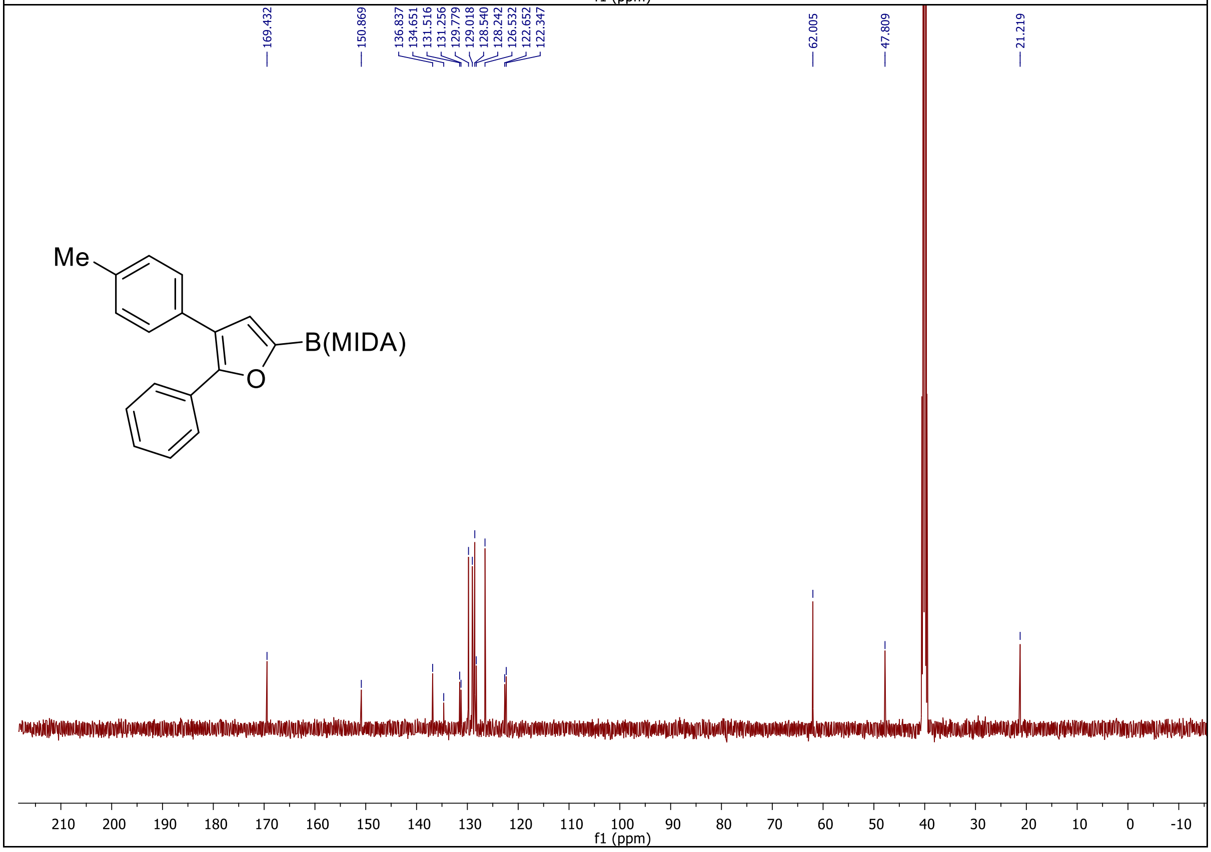
169.432
150.869
136.837
134.651
131.516
131.256
129.779
129.018
128.540
128.242
126.532
122.652
122.347
62.005
47.809
21.219
Me
B(MIDA)
O
f1 (ppm)

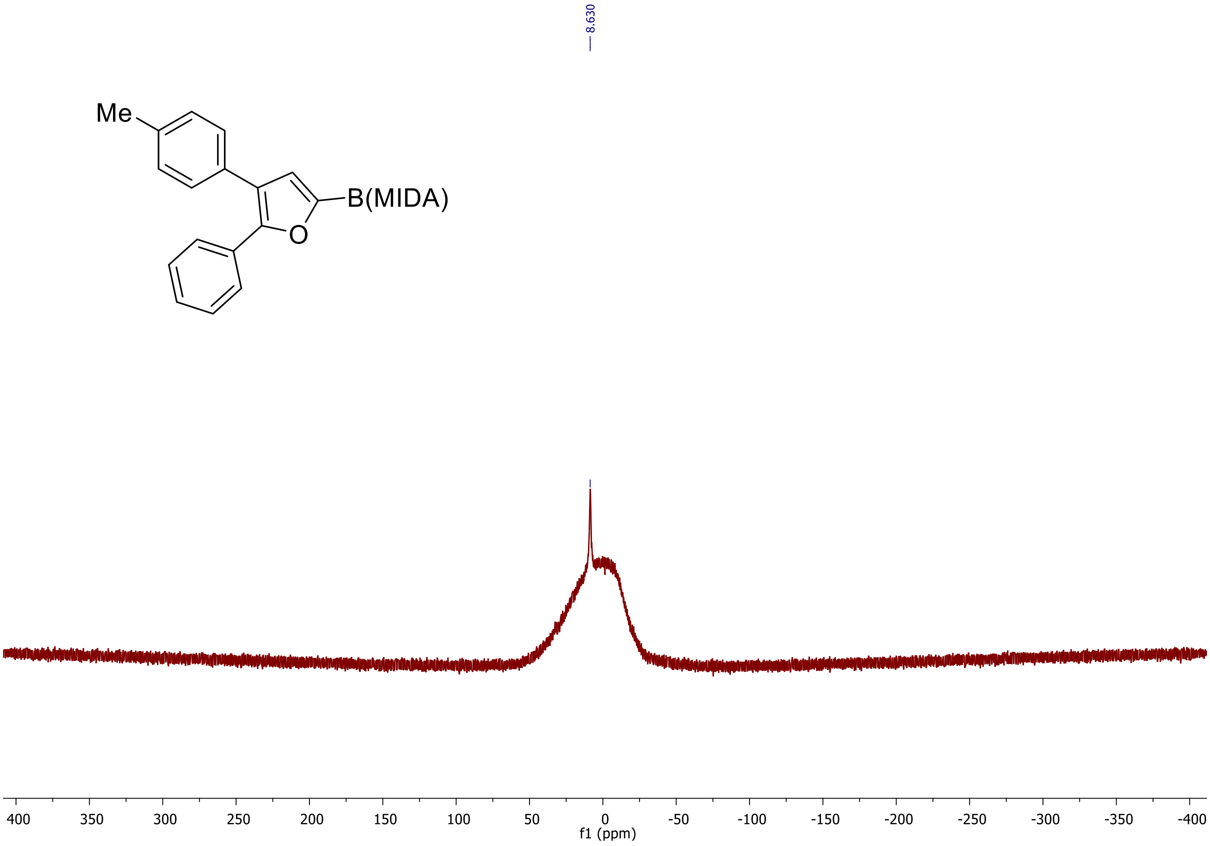
8.630
Me
B(MIDA)
O
400 350 300 250 200 150 100 50 0 -50 -100 -150 -200 -250 -300 -350 -400
f1 (ppm)

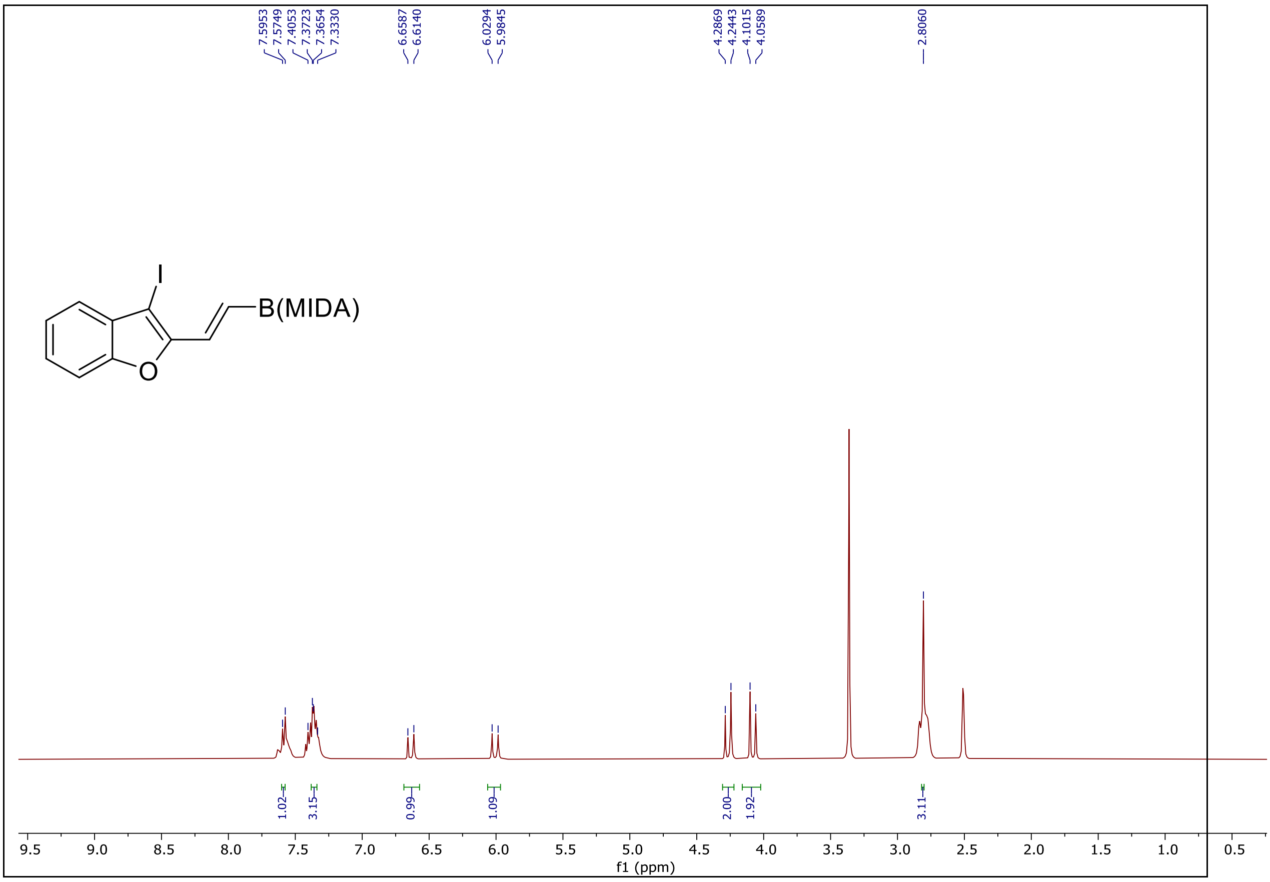
7.5953
7.5749
7.4053
7.3723
7.3654
7.3330
6.6587
6.6140
6.0294
5.9845
4.2869
4.2443
4.1015
4.0589
2.8060
I
B(MIDA)
O
1.02
3.15
0.99
1.09
2.00
1.92
3.11
9.5 9.0 8.5 8.0 7.5 7.0 6.5 6.0 5.5 5.0 4.5 4.0 3.5 3.0 2.5 2.0 1.5 1.0 0.5
f1 (ppm)

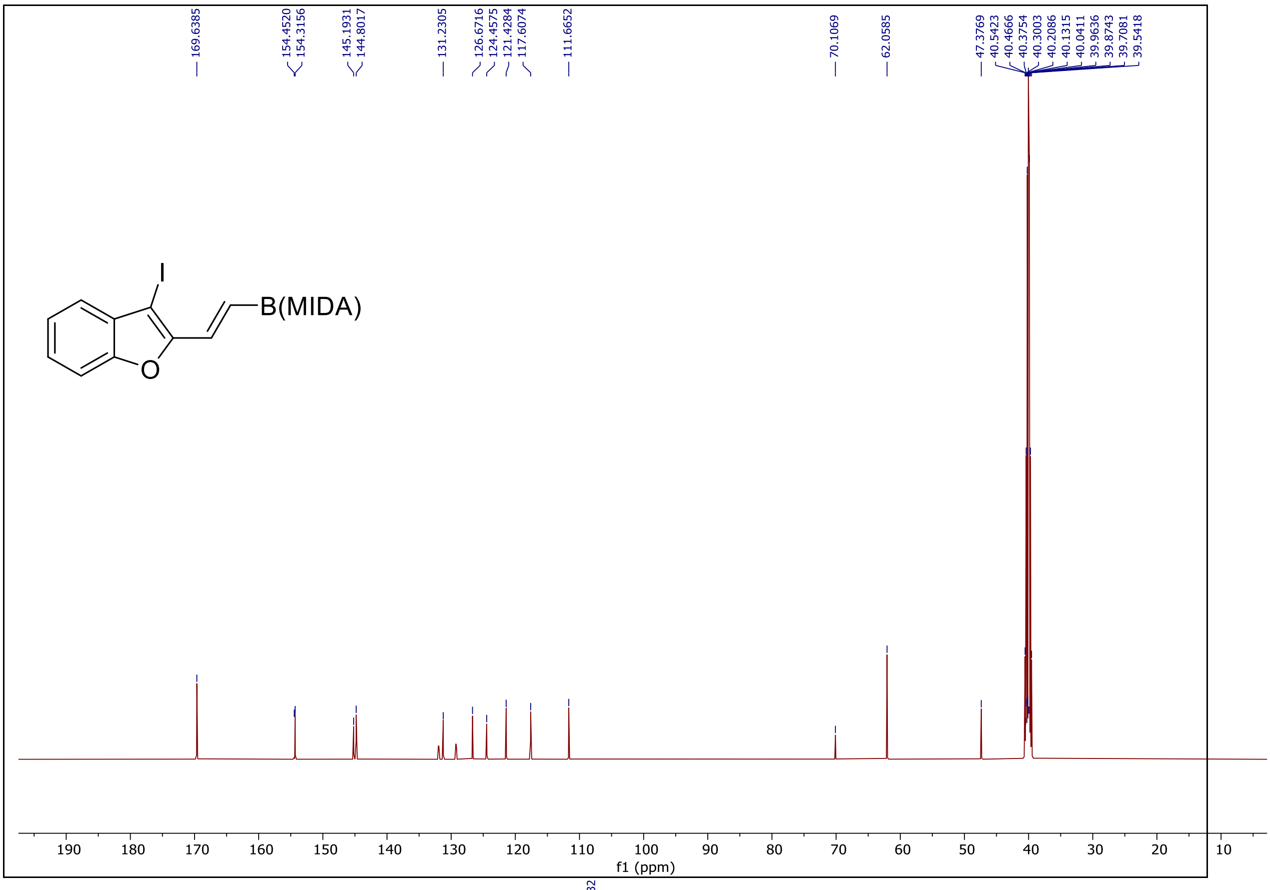
B(MIDA)
I
O
169.6385
154.4520
154.3156
145.1931
144.8017
131.2305
126.6716
124.4575
121.4284
117.6074
111.6652
70.1069
62.0585
47.3769
40.5423
40.4666
40.3754
40.3003
40.2086
40.1315
40.0411
39.9636
39.8743
39.7081
39.5418
f1 (ppm)

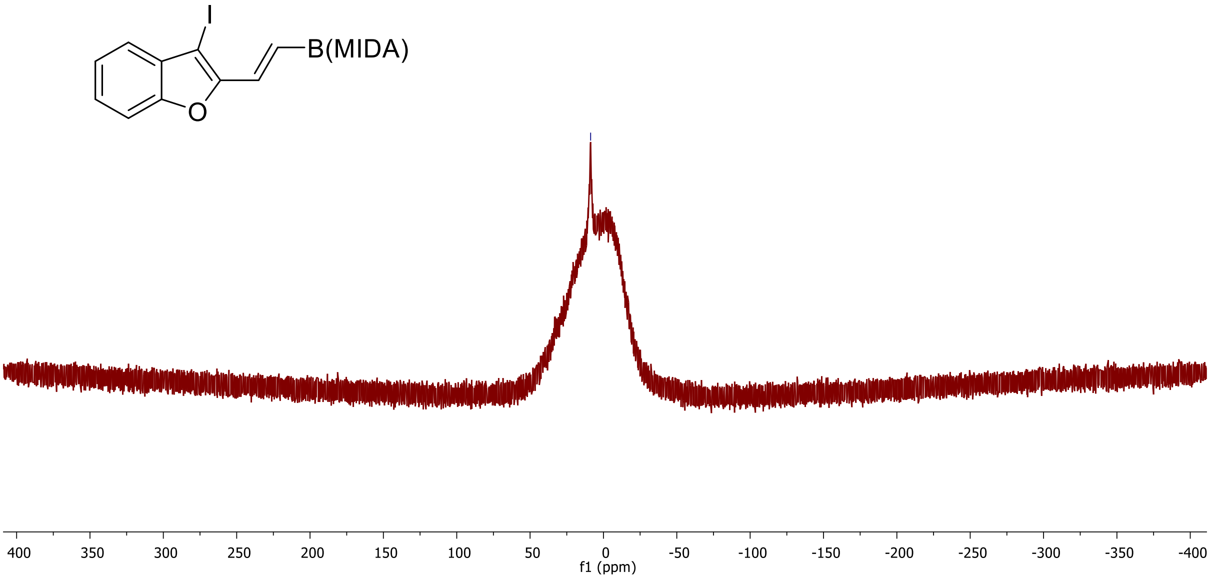
8.732
B(MIDA)
I
O
f1 (ppm)

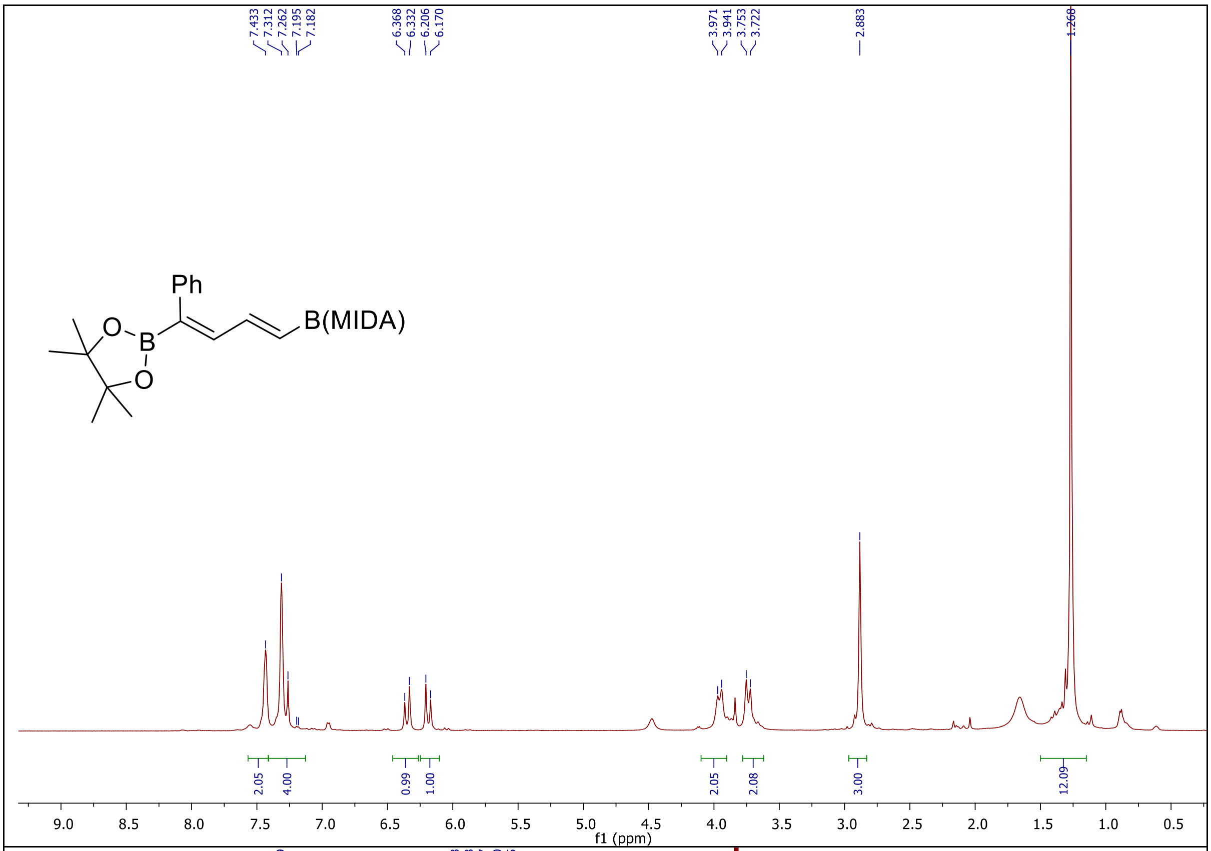
7.433
7.312
7.262
7.195
7.182
6.368
6.332
6.206
6.170
3.971
3.941
3.753
3.722
2.883
1.268
Ph
O
B
O
B(MIDA)
2.05
4.00
0.99
1.00
2.05
2.08
3.00
12.09
9.0 8.5 8.0 7.5 7.0 6.5 6.0 5.5 5.0 4.5 4.0 3.5 3.0 2.5 2.0 1.5 1.0 0.5
f1 (ppm)

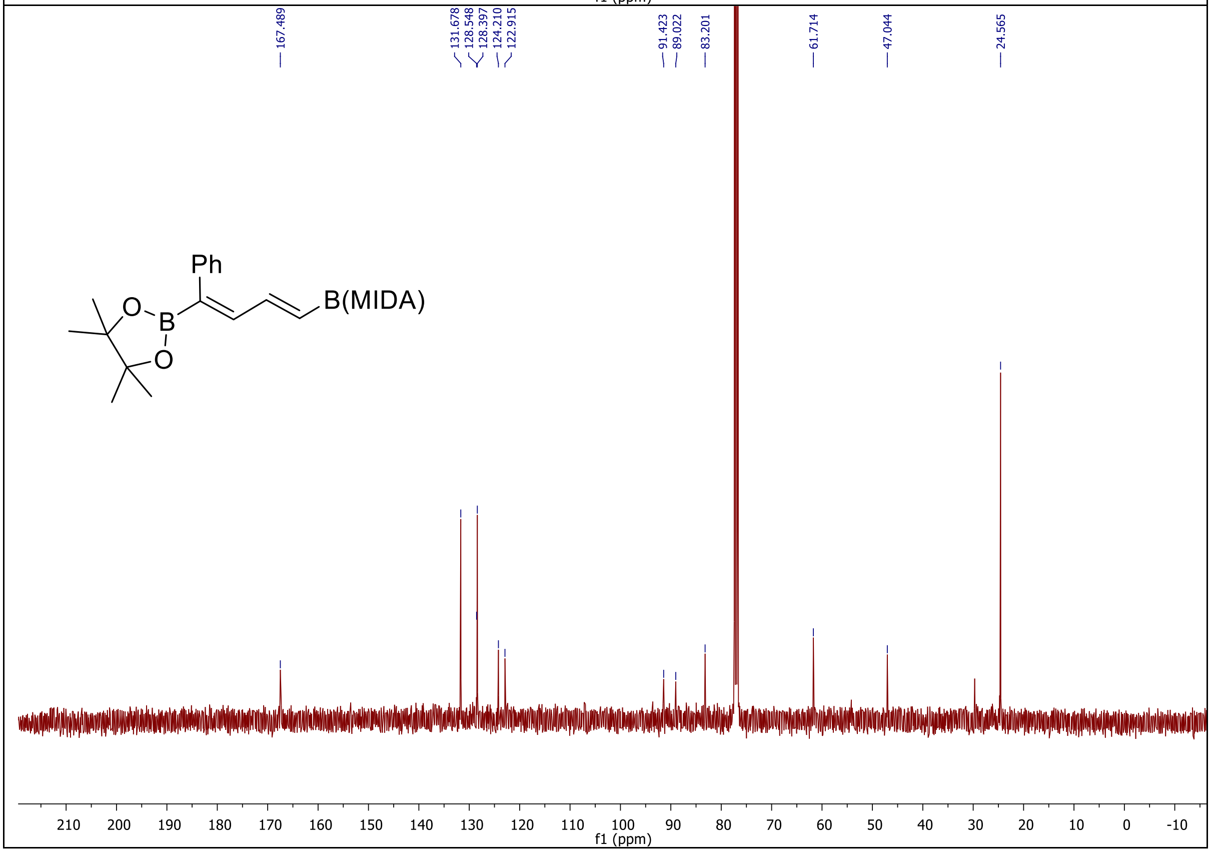
167.489
131.678
128.548
128.397
124.210
122.915
91.423
89.022
83.201
61.714
47.044
24.565
Ph
O
B
O
B(MIDA)
210 200 190 180 170 160 150 140 130 120 110 100 90 80 70 60 50 40 30 20 10 0 -10
f1 (ppm)

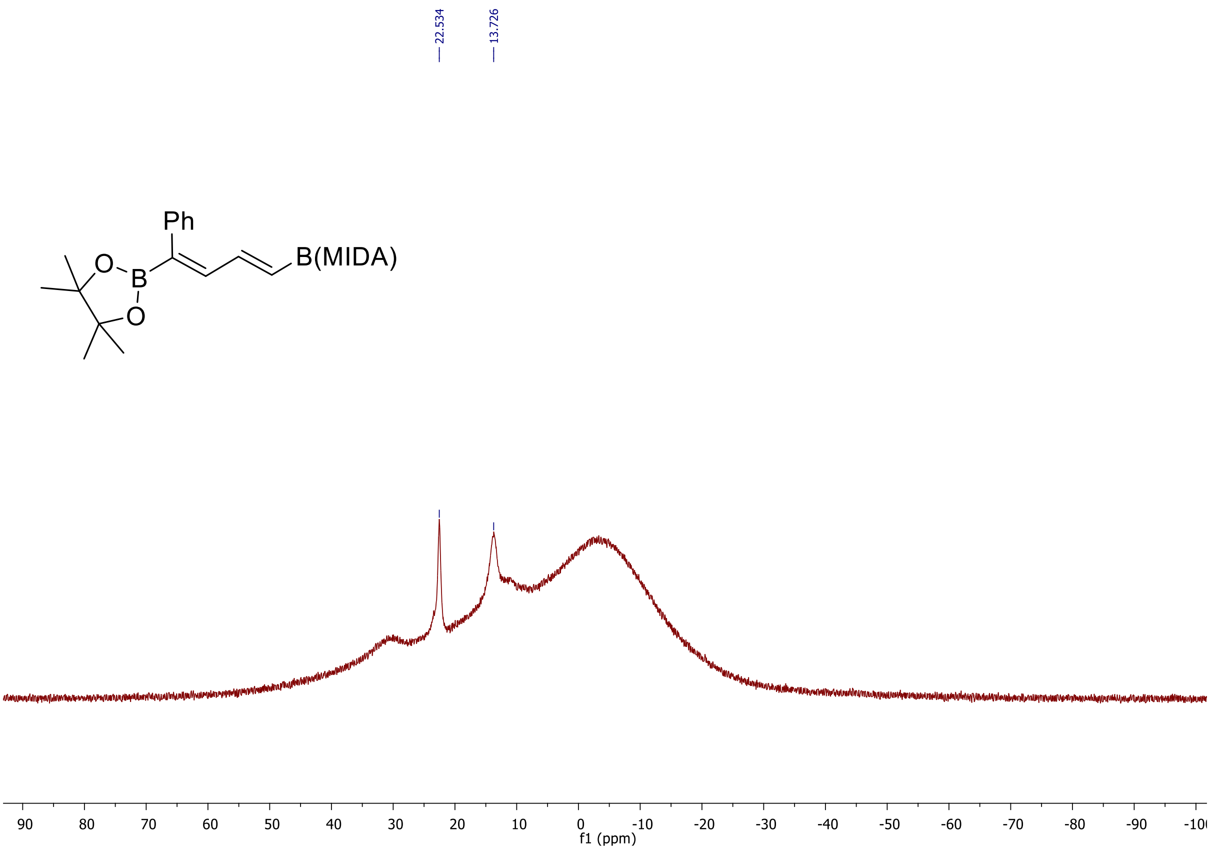
22.534
13.726
Ph
B(MIDA)
O
B
O
90
80
70
60
50
40
30
20
10
0
-10
-20
-30
-40
-50
-60
-70
-80
-90
f1 (ppm)

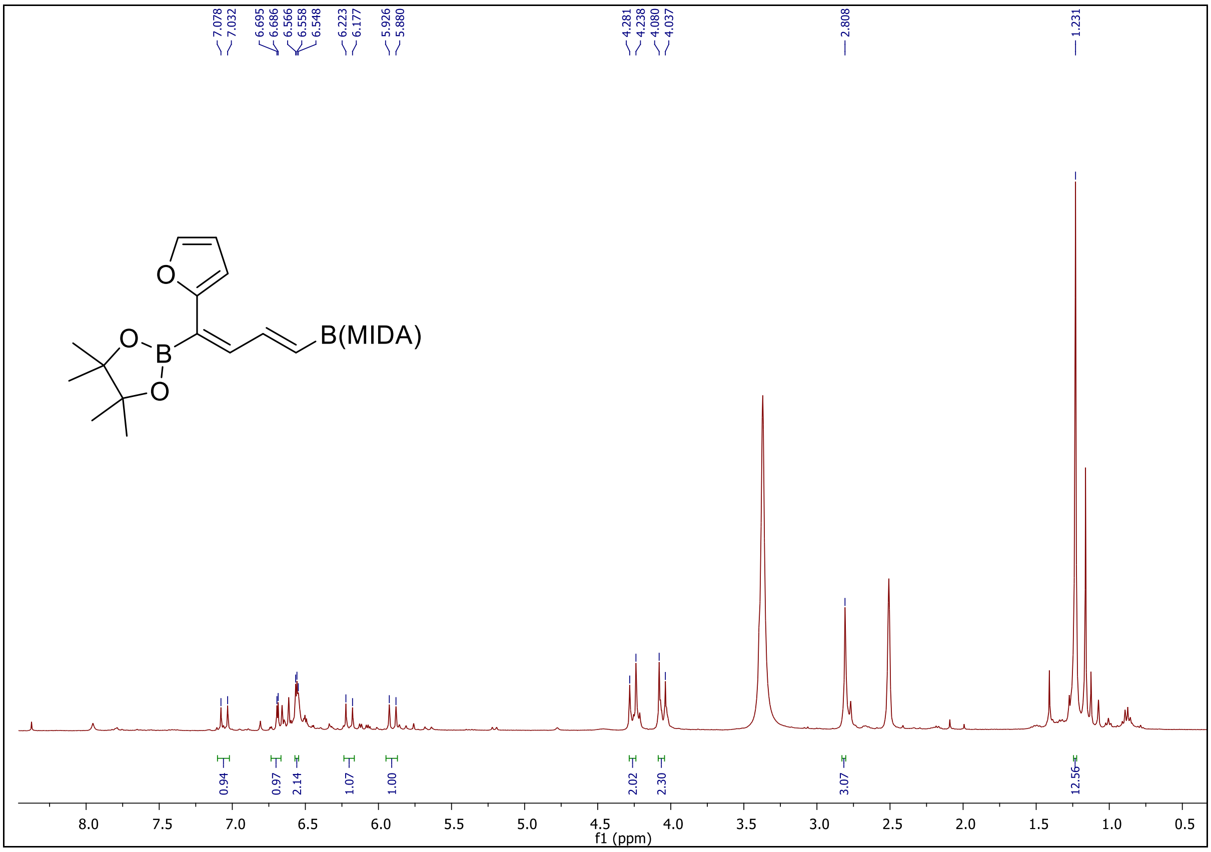
7.078
7.032
6.695
6.686
6.566
6.558
6.548
6.223
6.177
5.926
5.880
4.281
4.238
4.080
4.037
2.808
1.231
O
B(MIDA)
O
B
O
0.94
0.97
2.14
1.07
1.00
2.02
2.30
3.07
12.56
8.0
7.5
7.0
6.5
6.0
5.5
5.0
4.5
4.0
3.5
3.0
2.5
2.0
1.5
1.0
0.5
f1 (ppm)

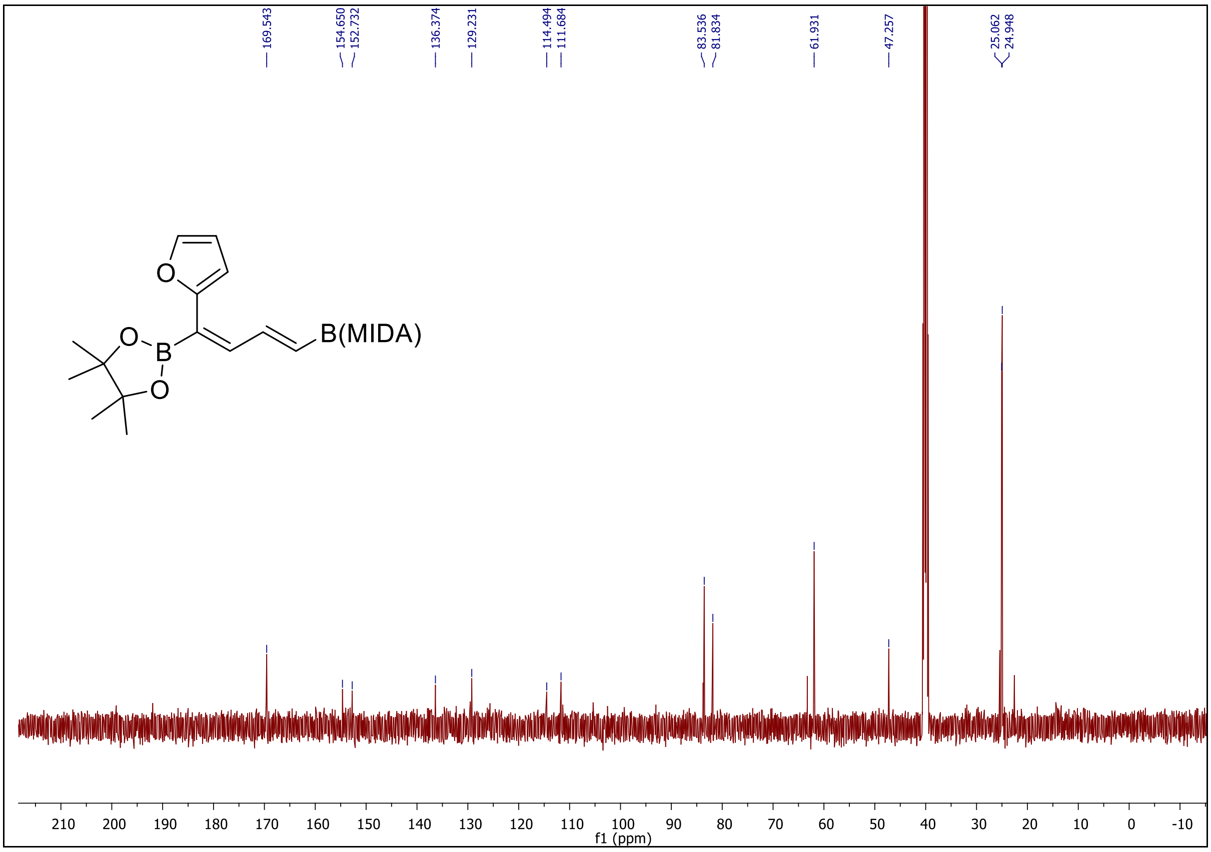
169.543
154.650
152.732
136.374
129.231
114.494
111.684
83.536
81.834
61.931
47.257
25.062
24.948
B(MIDA)
f1 (ppm)

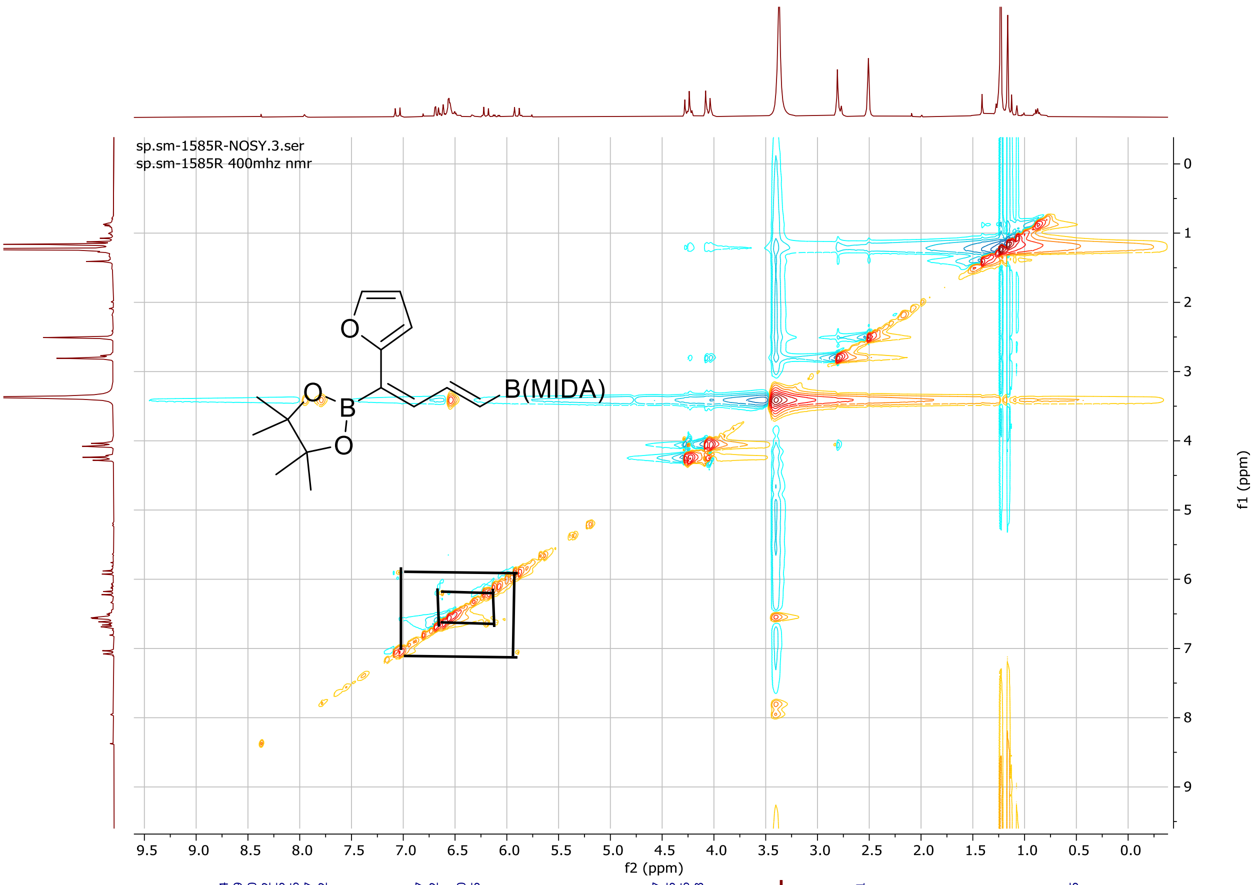
sp.sm-1585R-NOSY.3.ser
sp.sm-1585R 400mhz nmr
B(MIDA)
f2 (ppm)
f1 (ppm)

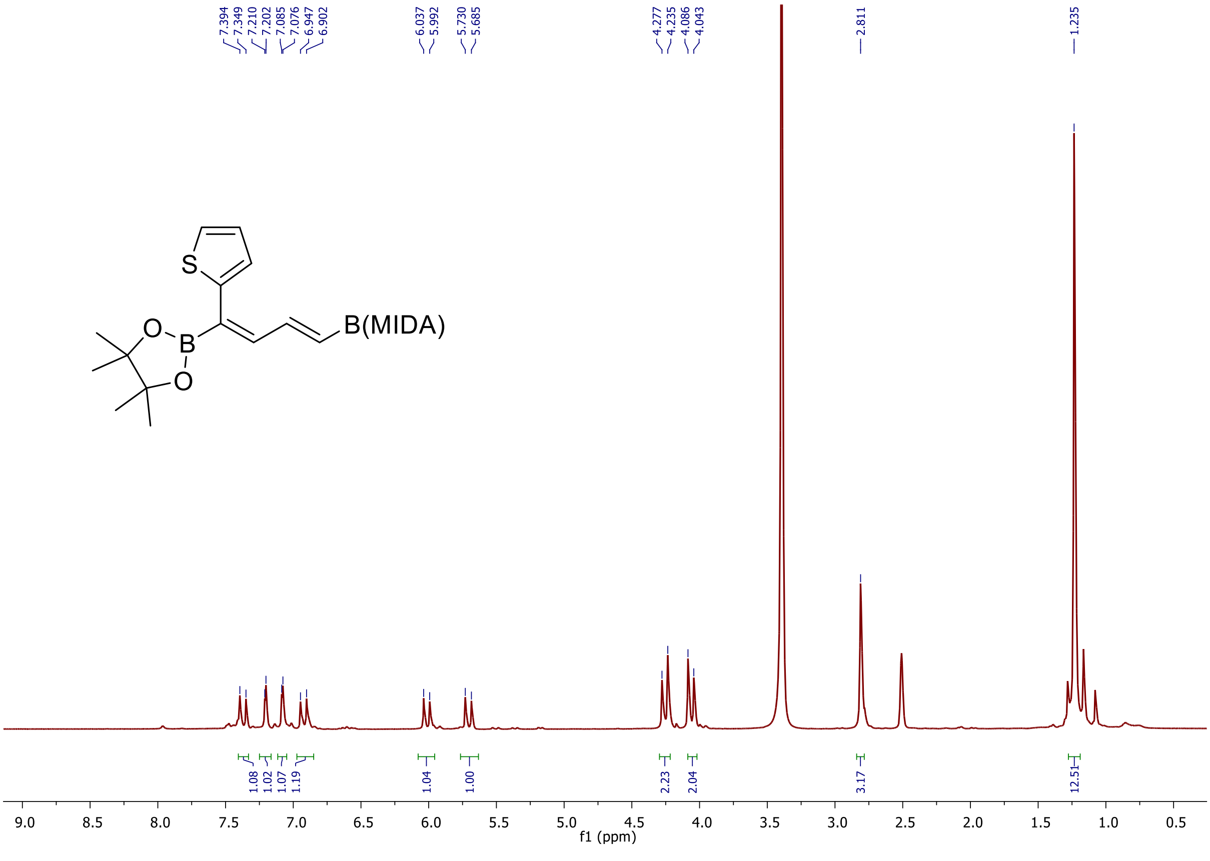
7.394
7.349
7.210
7.202
7.085
7.076
6.947
6.902
6.037
5.992
5.730
5.685
4.277
4.235
4.086
4.043
2.811
1.235
B(MIDA)
1.08
1.02
1.07
1.19
1.04
1.00
2.23
2.04
3.17
12.51
f1 (ppm)

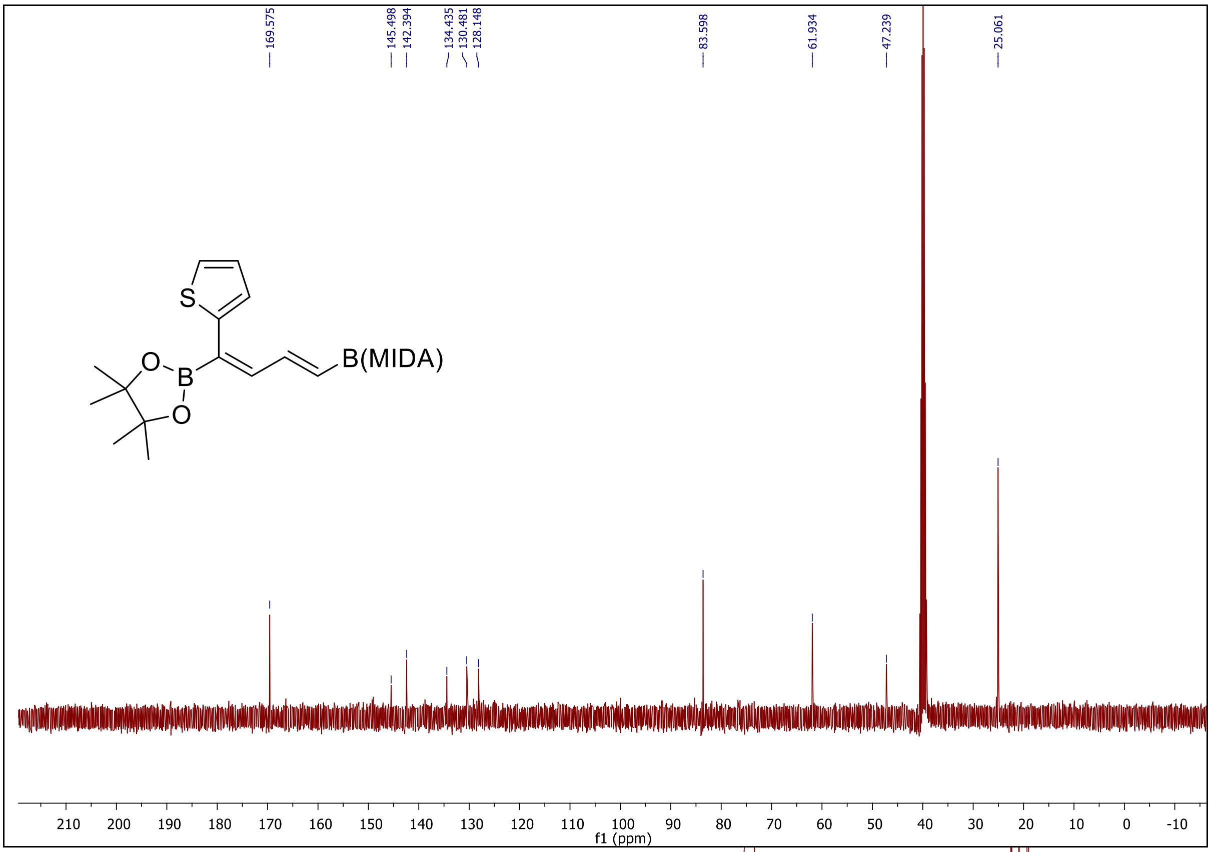
169.575
145.498
142.394
134.435
130.481
128.148
83.598
61.934
47.239
25.061
B(MIDA)
f1 (ppm)

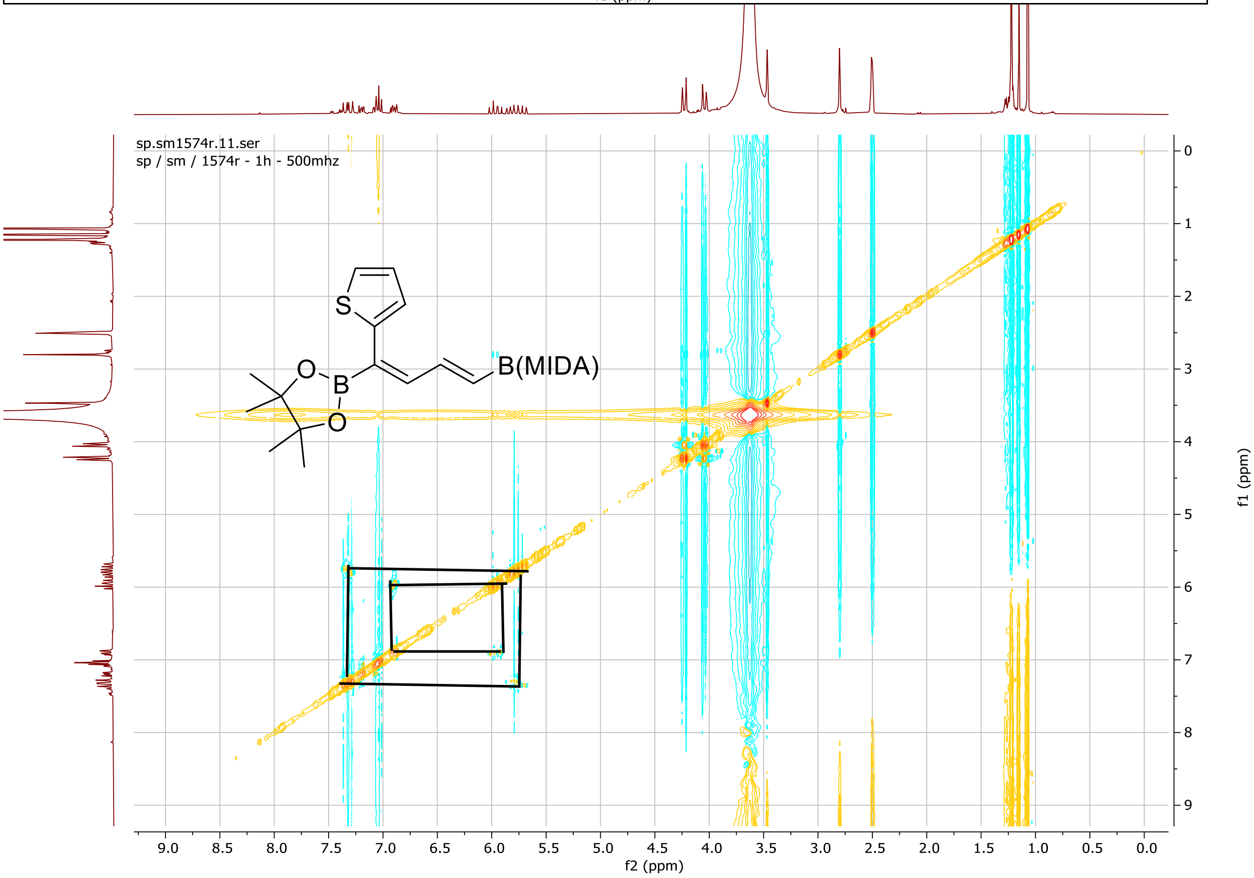
sp.sm1574r.11.ser
sp / sm / 1574r - 1h - 500mhz
B(MIDA)
f2 (ppm)
f1 (ppm)

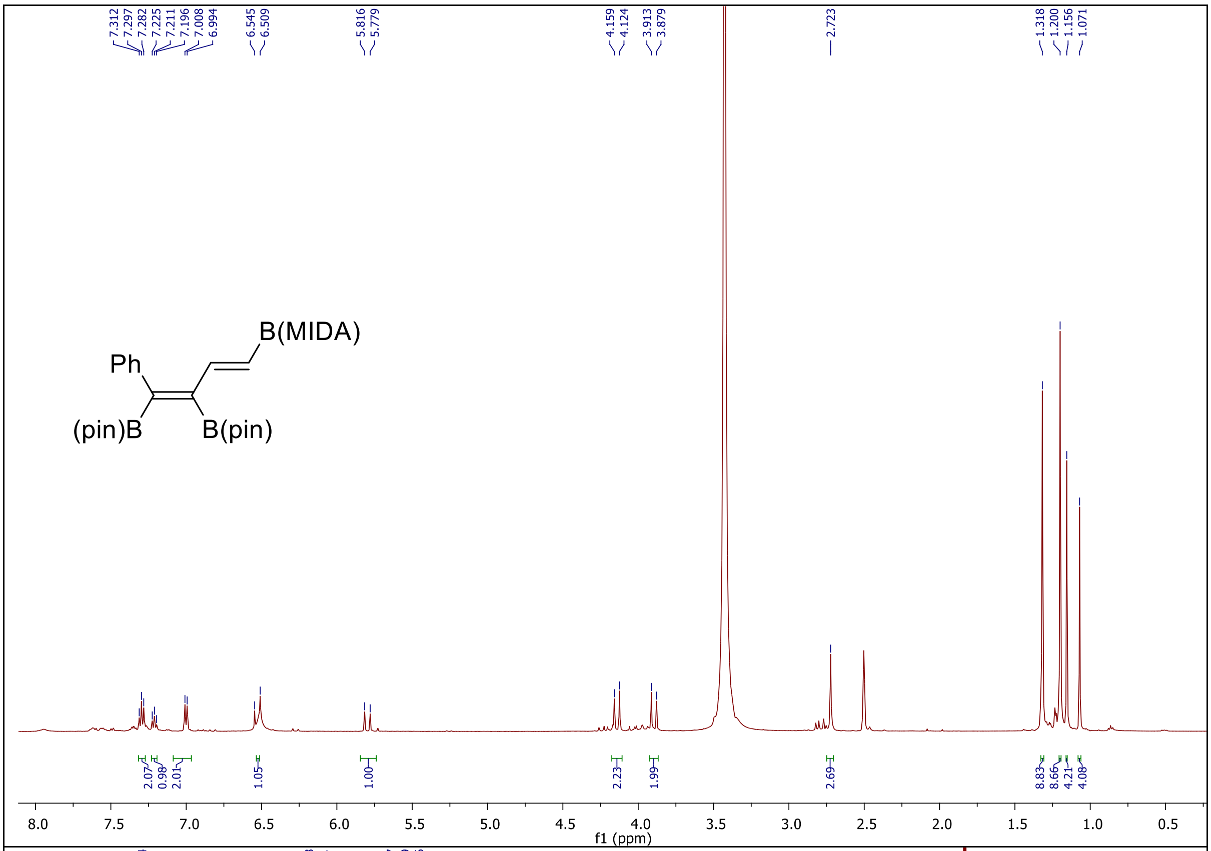
7.312
7.297
7.282
7.225
7.211
7.196
7.008
6.994
6.545
6.509
5.816
5.779
4.159
4.124
3.913
3.879
2.723
1.318
1.200
1.156
1.071
B(MIDA)
Ph
(pin)B
B(pin)
2.07
0.98
2.01
1.05
1.00
2.23
1.99
2.69
8.83
8.66
4.21
4.08
f1 (ppm)

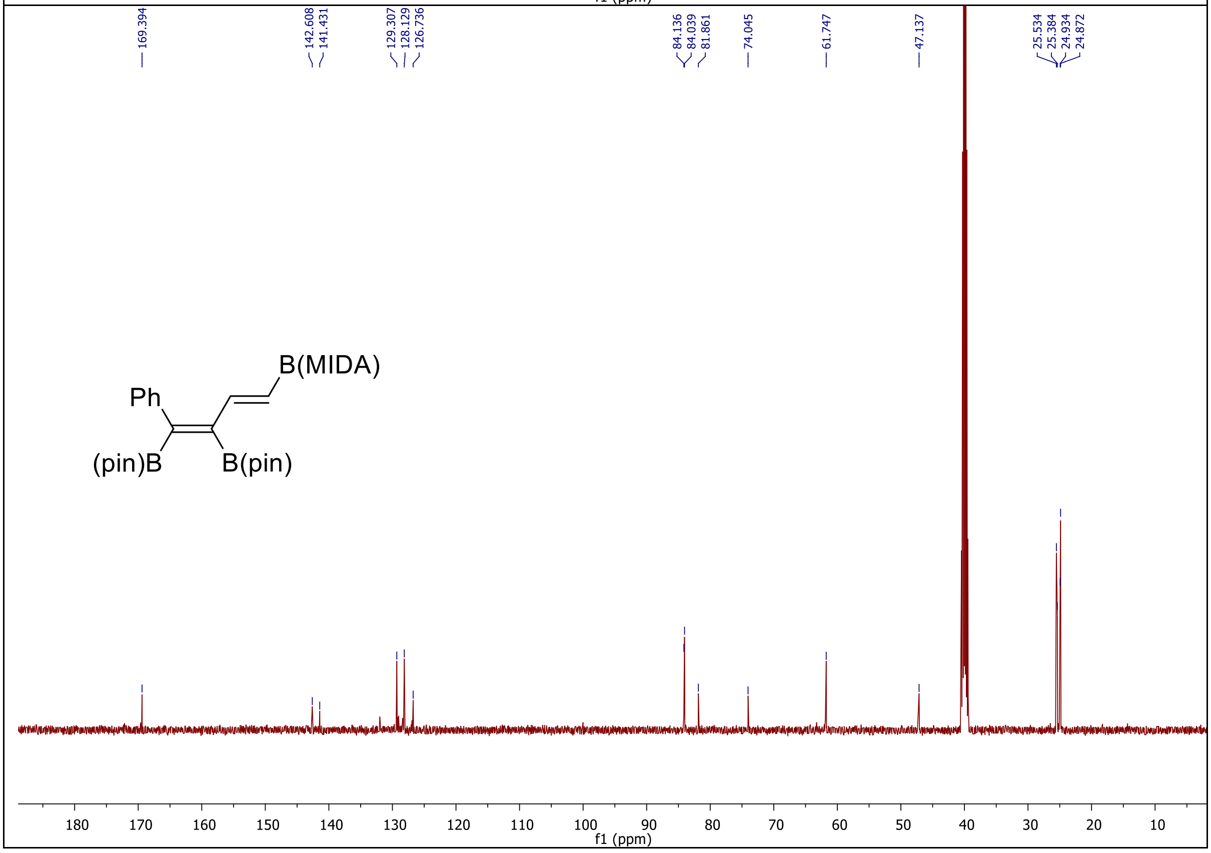
169.394
142.608
141.431
129.307
128.129
126.736
84.136
84.039
81.861
74.045
61.747
47.137
25.534
25.384
24.934
24.872
B(MIDA)
Ph
(pin)B
B(pin)
f1 (ppm)

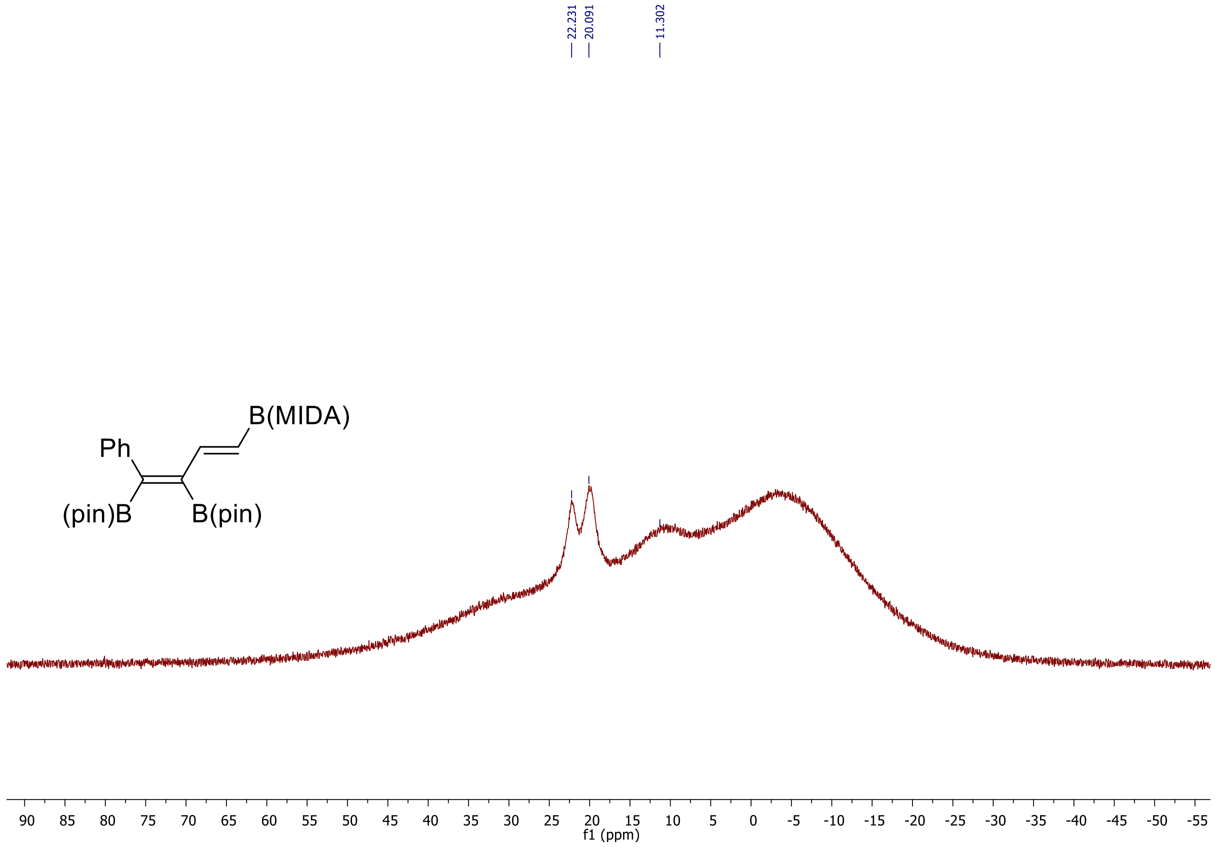
22.231
20.091
11.302
B(MIDA)
Ph
(pin)B
B(pin)
90 85 80 75 70 65 60 55 50 45 40 35 30 25 20 15 10 5 0 -5 -10 -15 -20 -25 -30 -35 -40 -45 -50 -55
f1 (ppm)

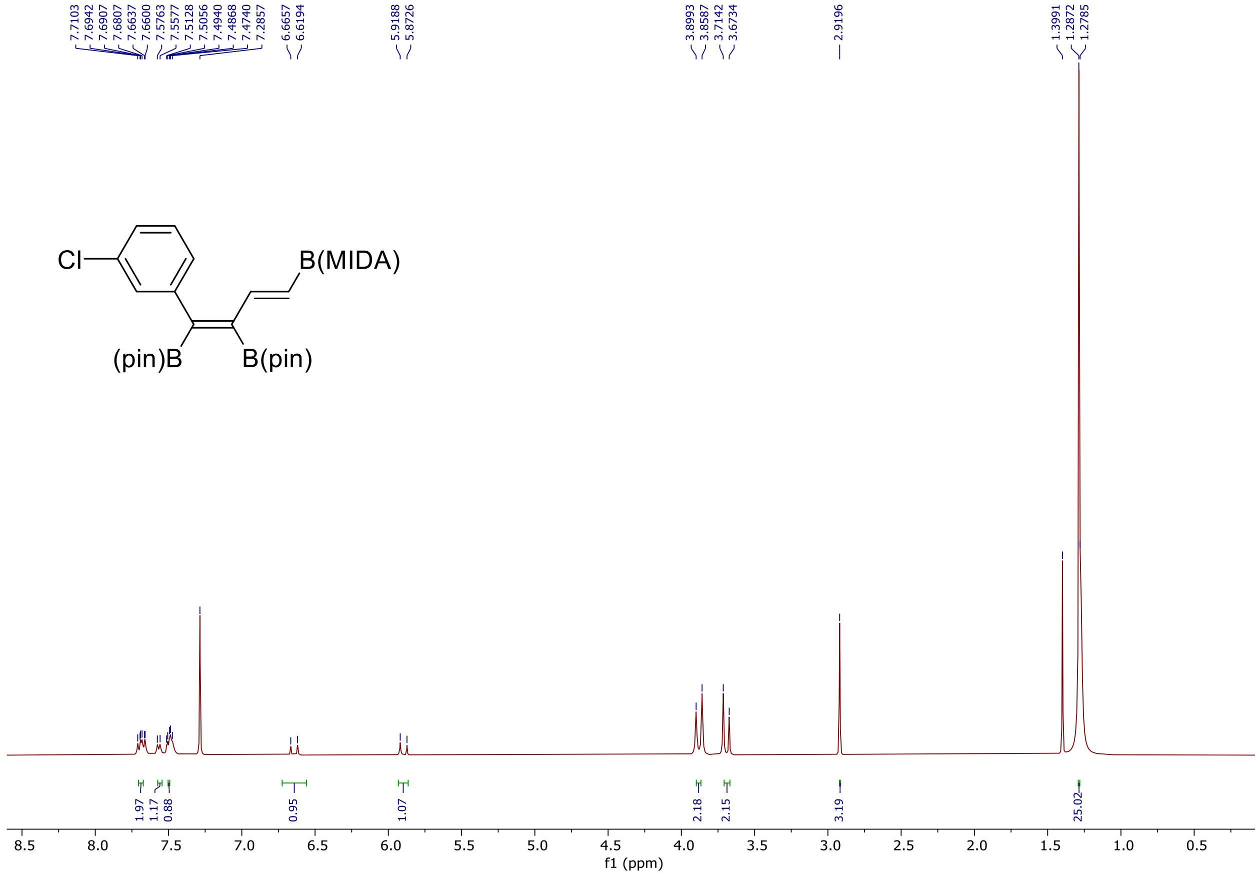
7.7103
7.6942
7.6907
7.6807
7.6637
7.6600
7.5763
7.5577
7.5128
7.5056
7.4940
7.4868
7.4740
7.2857
6.6657
6.6194
5.9188
5.8726
3.8993
3.8587
3.7142
3.6734
2.9196
1.3991
1.2872
1.2785
Cl
B(MIDA)
(pin)B
B(pin)
1.97
1.17
0.88
0.95
1.07
2.18
2.15
3.19
25.02
8.5 8.0 7.5 7.0 6.5 6.0 5.5 5.0 4.5 4.0 3.5 3.0 2.5 2.0 1.5 1.0 0.5
f1 (ppm)

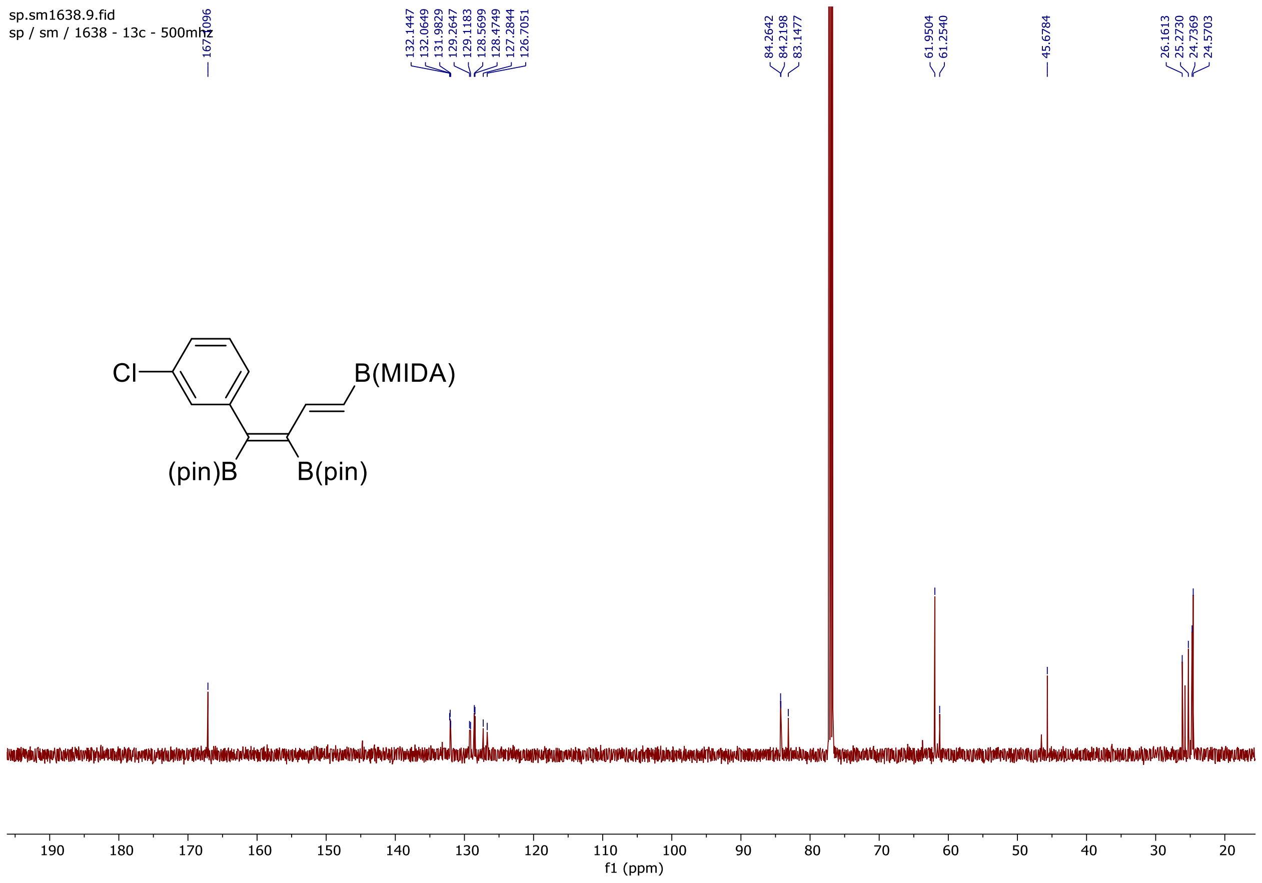
sp.sm1638.9.fid
sp / sm / 1638 - 13c - 500mhz
132.1447
132.0649
131.9829
129.2647
129.1183
128.5699
128.4749
127.2844
126.7051
84.2642
84.2198
83.1477
61.9504
61.2540
45.6784
26.1613
25.2730
24.7369
24.5703
Cl
B(MIDA)
(pin)B
B(pin)
190
180
170
160
150
140
130
120
110
100
90
80
70
60
50
40
30
20
f1 (ppm)

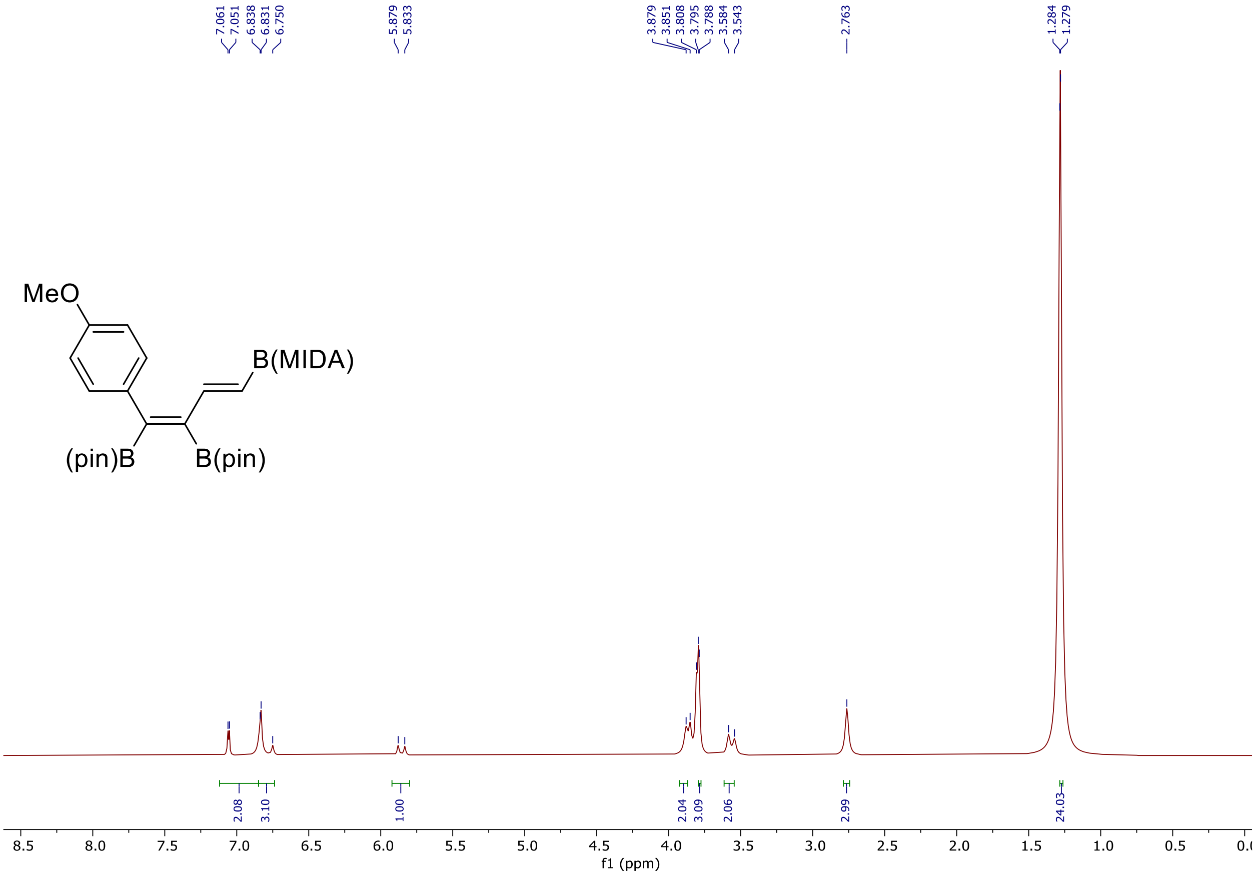
7.061
7.051
6.838
6.831
6.750
5.879
5.833
3.879
3.851
3.808
3.795
3.788
3.584
3.543
2.763
1.284
1.279
MeO
B(MIDA)
(pin)B
B(pin)
2.08
3.10
1.00
2.04
3.09
2.06
2.99
24.03
f1 (ppm)

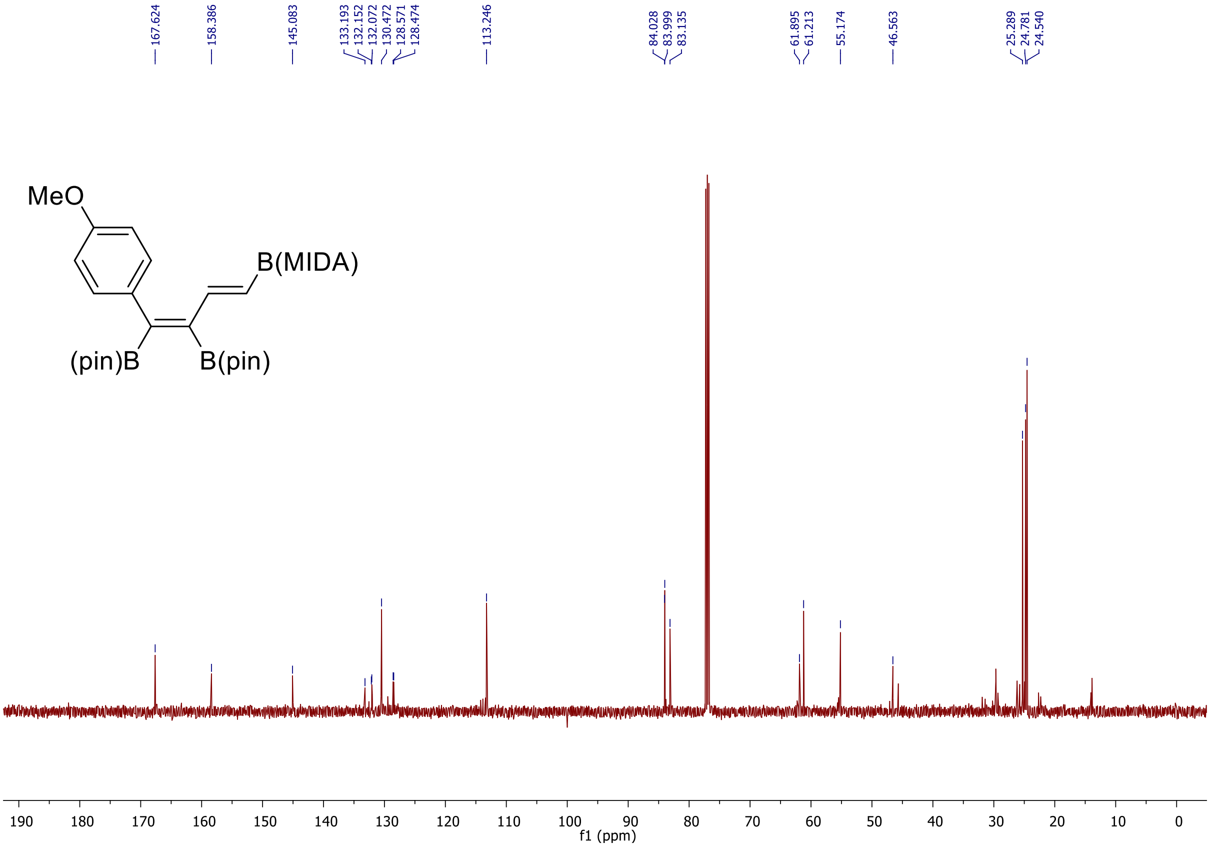
167.624
158.386
145.083
133.193
132.152
132.072
130.472
128.571
128.474
113.246
84.028
83.999
83.135
61.895
61.213
55.174
46.563
25.289
24.781
24.540
MeO
B(MIDA)
(pin)B
B(pin)
f1 (ppm)

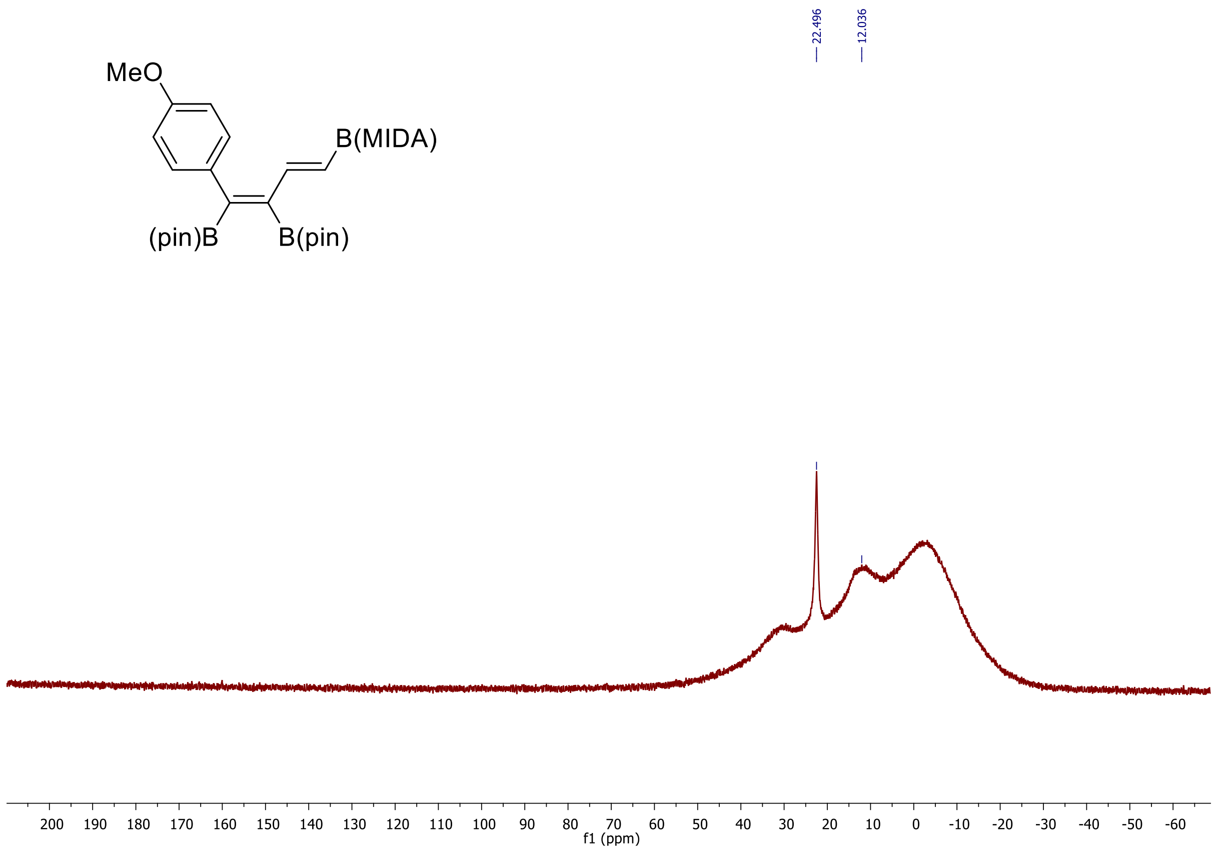
MeO
B(MIDA)
(pin)B
B(pin)
22.496
12.036
f1 (ppm)

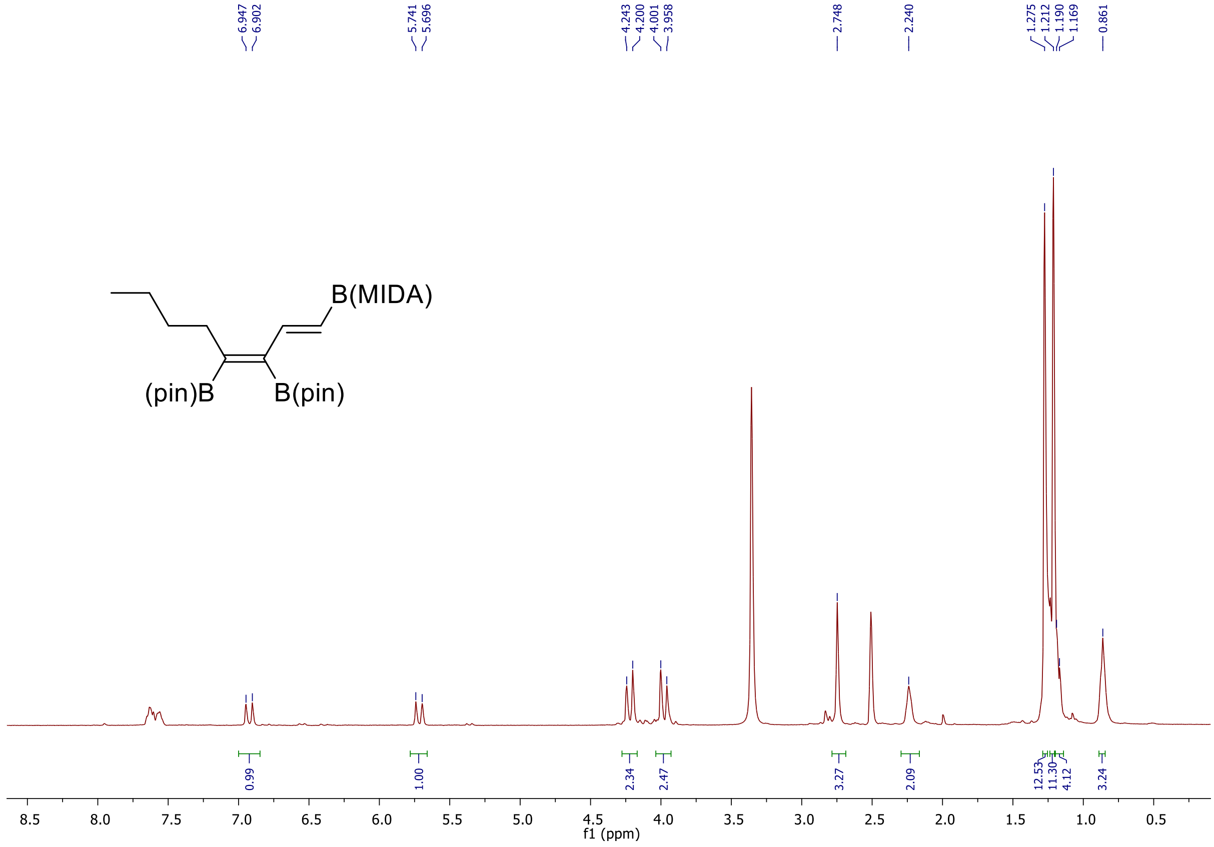
B(MIDA)
(pin)B
B(pin)
6.947
6.902
5.741
5.696
4.243
4.200
4.001
3.958
2.748
2.240
1.275
1.212
1.190
1.169
0.861
0.99
1.00
2.34
2.47
3.27
2.09
12.53
11.30
4.12
3.24
f1 (ppm)

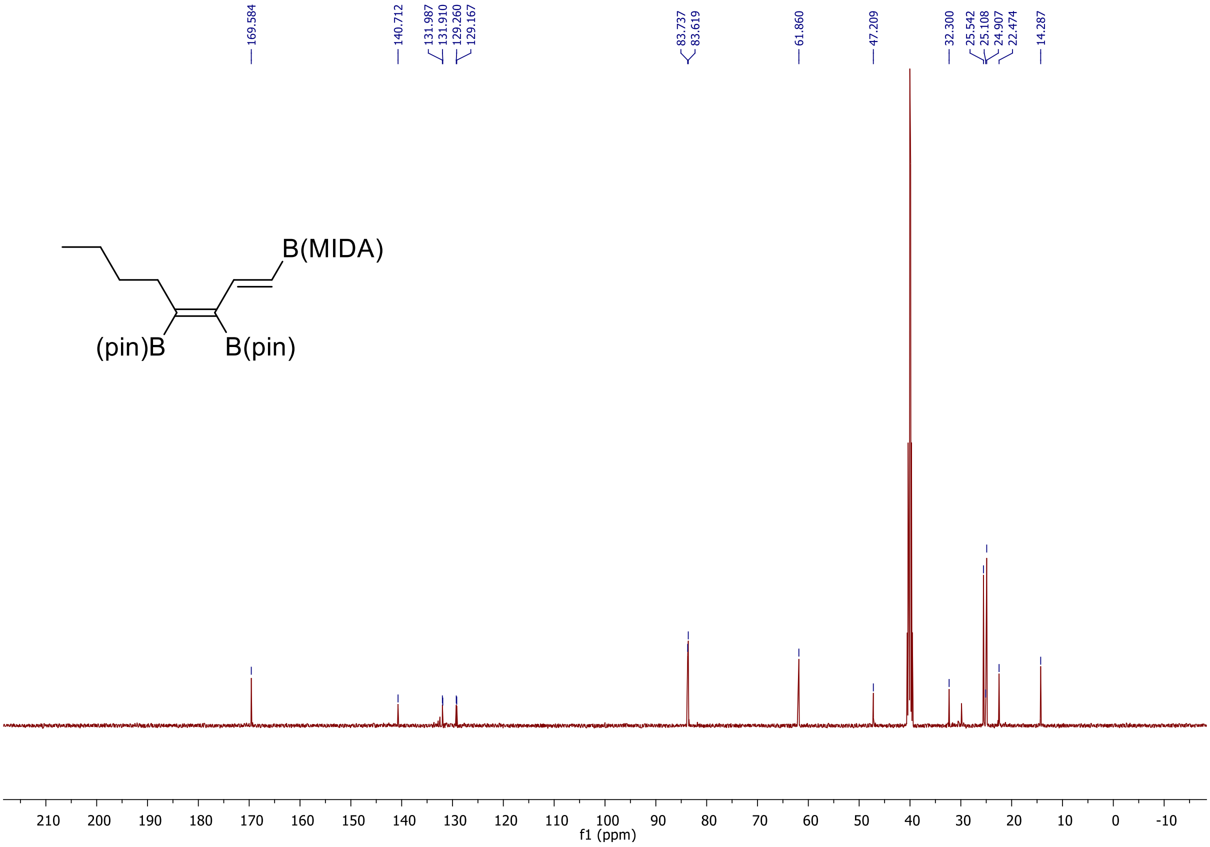

B(MIDA)
(pin)B
B(pin)
169.584
140.712
131.987
131.910
129.260
129.167
83.737
83.619
61.860
47.209
32.300
25.542
25.108
24.907
22.474
14.287
210
200
190
180
170
160
150
140
130
120
110
100
90
80
70
60
50
40
30
20
10
0
-10
f1 (ppm)

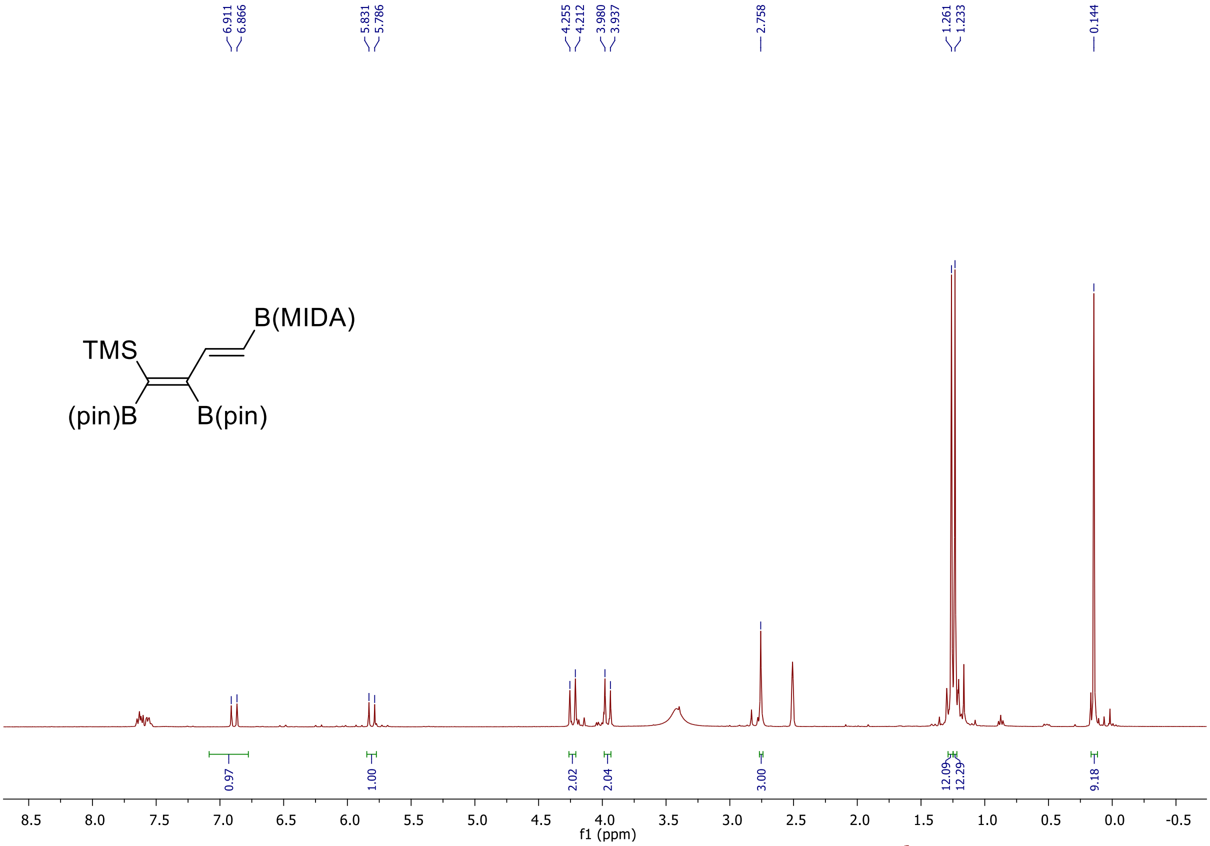
6.911
6.866
5.831
5.786
4.255
4.212
3.980
3.937
2.758
1.261
1.233
0.144
B(MIDA)
TMS
(pin)B
B(pin)
0.97
1.00
2.02
2.04
3.00
12.09
12.29
9.18
f1 (ppm)

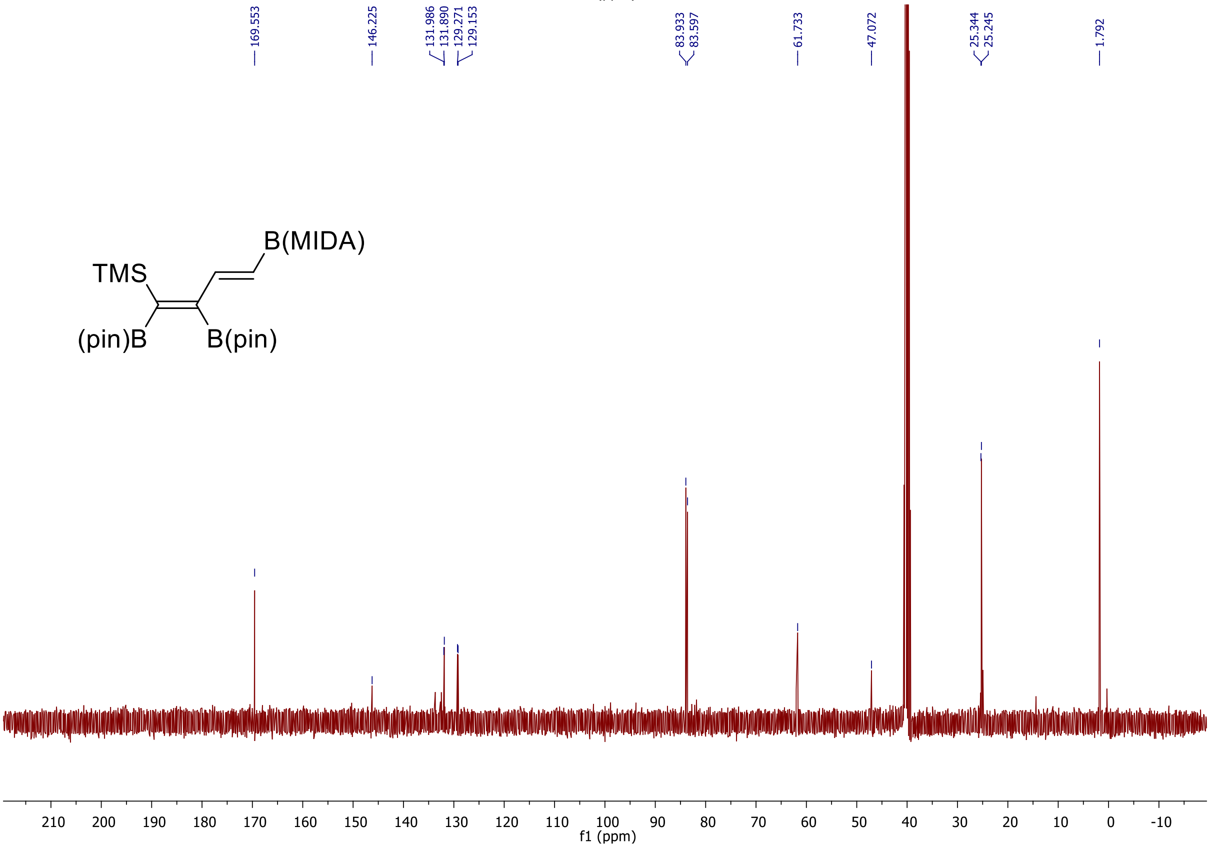
169.553
146.225
131.986
131.890
129.271
129.153
83.933
83.597
61.733
47.072
25.344
25.245
1.792
B(MIDA)
TMS
(pin)B
B(pin)
f1 (ppm)

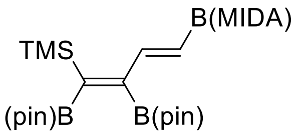

TMS
B(MIDA)
(pin)B
B(pin)

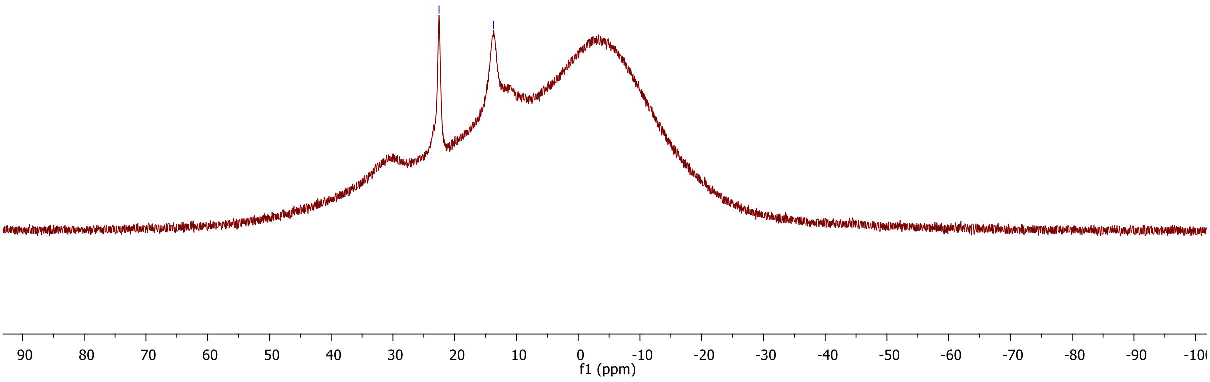

22.534
13.726
90
80
70
60
50
40
30
20
10
0
-10
-20
-30
-40
-50
-60
-70
-80
-90
f1 (ppm)

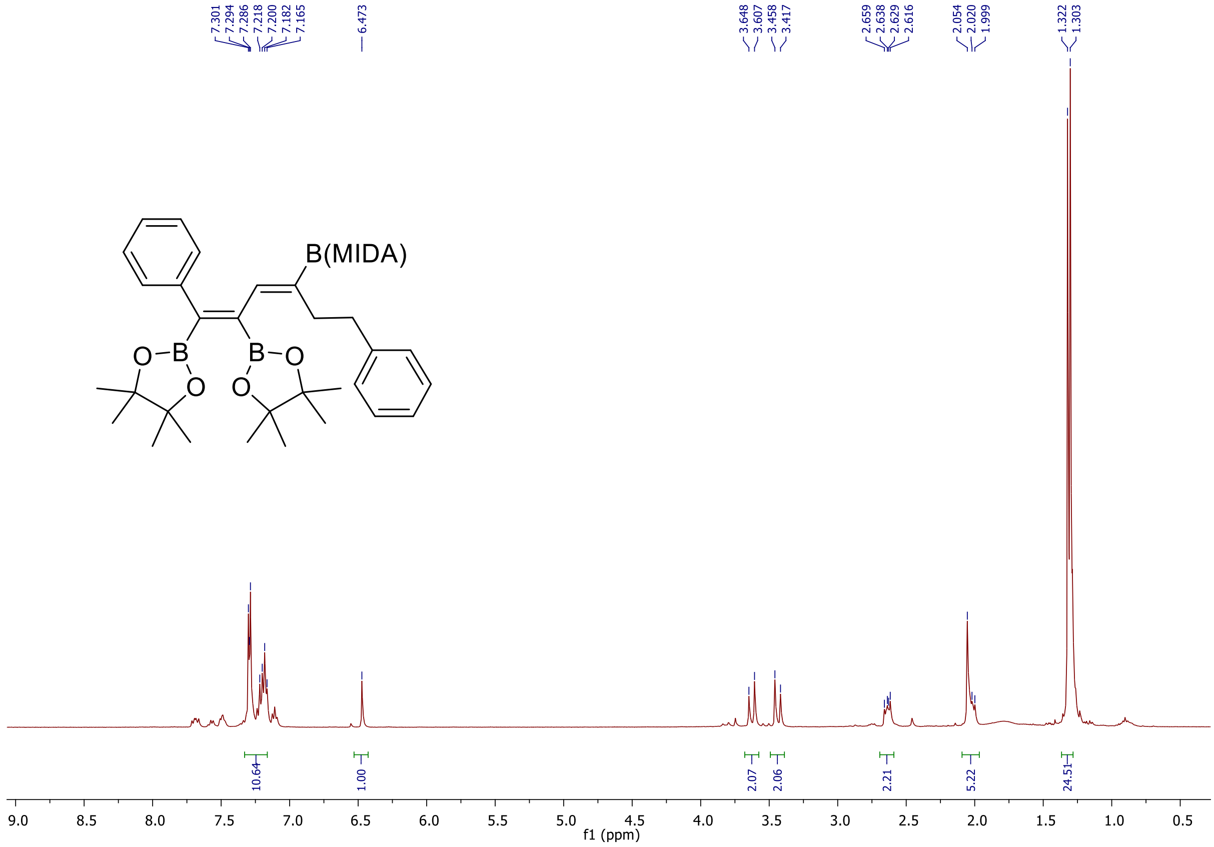
7.301
7.294
7.286
7.218
7.200
7.182
7.165
6.473
3.648
3.607
3.458
3.417
2.659
2.638
2.629
2.616
2.054
2.020
1.999
1.322
1.303
B(MIDA)
O
B
O
B
O
O
10.64
1.00
2.07
2.06
2.21
5.22
24.51
9.0
8.5
8.0
7.5
7.0
6.5
6.0
5.5
5.0
4.5
4.0
3.5
3.0
2.5
2.0
1.5
1.0
0.5
f1 (ppm)

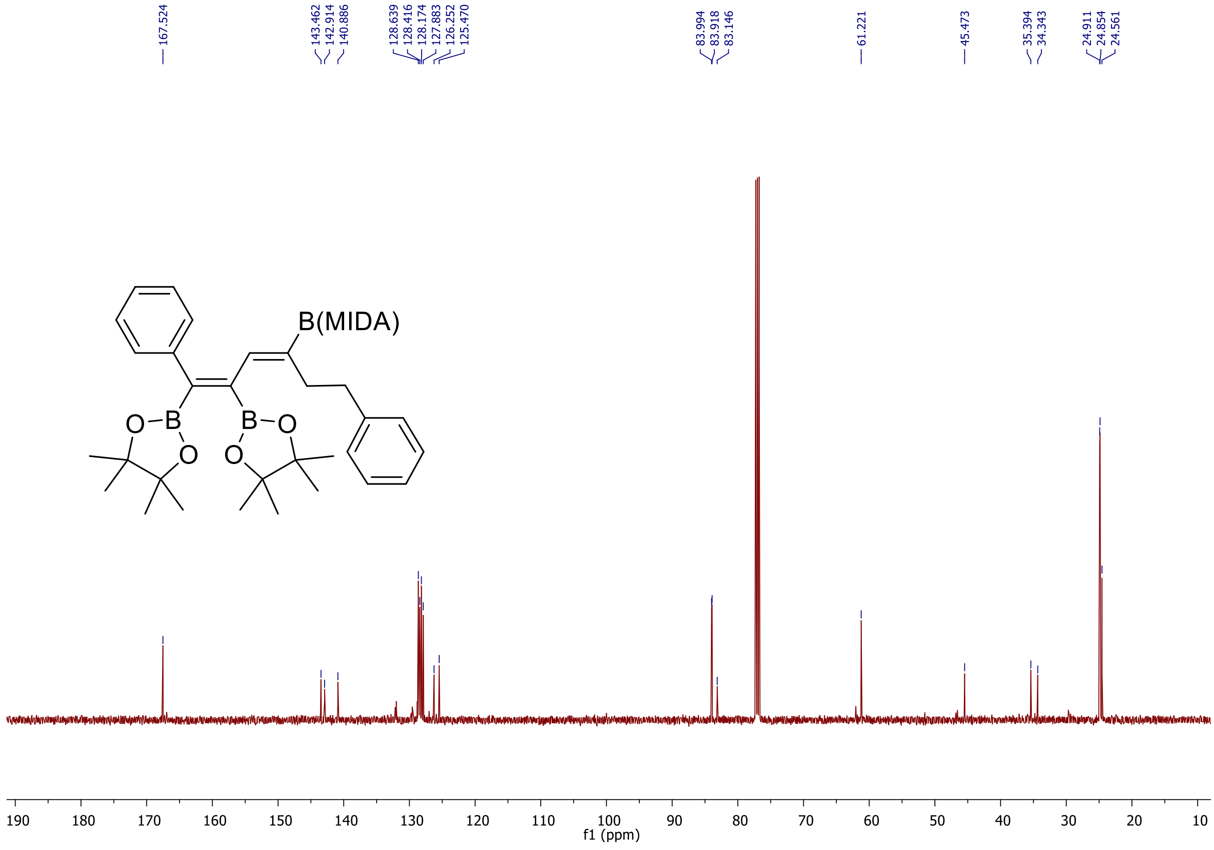
167.524
143.462
142.914
140.886
128.639
128.416
128.174
127.883
126.252
125.470
83.994
83.918
83.146
61.221
45.473
35.394
34.343
24.911
24.854
24.561
B(MIDA)
O
B
O
B
O
O
190
180
170
160
150
140
130
120
110
100
90
80
70
60
50
40
30
20
10
f1 (ppm)

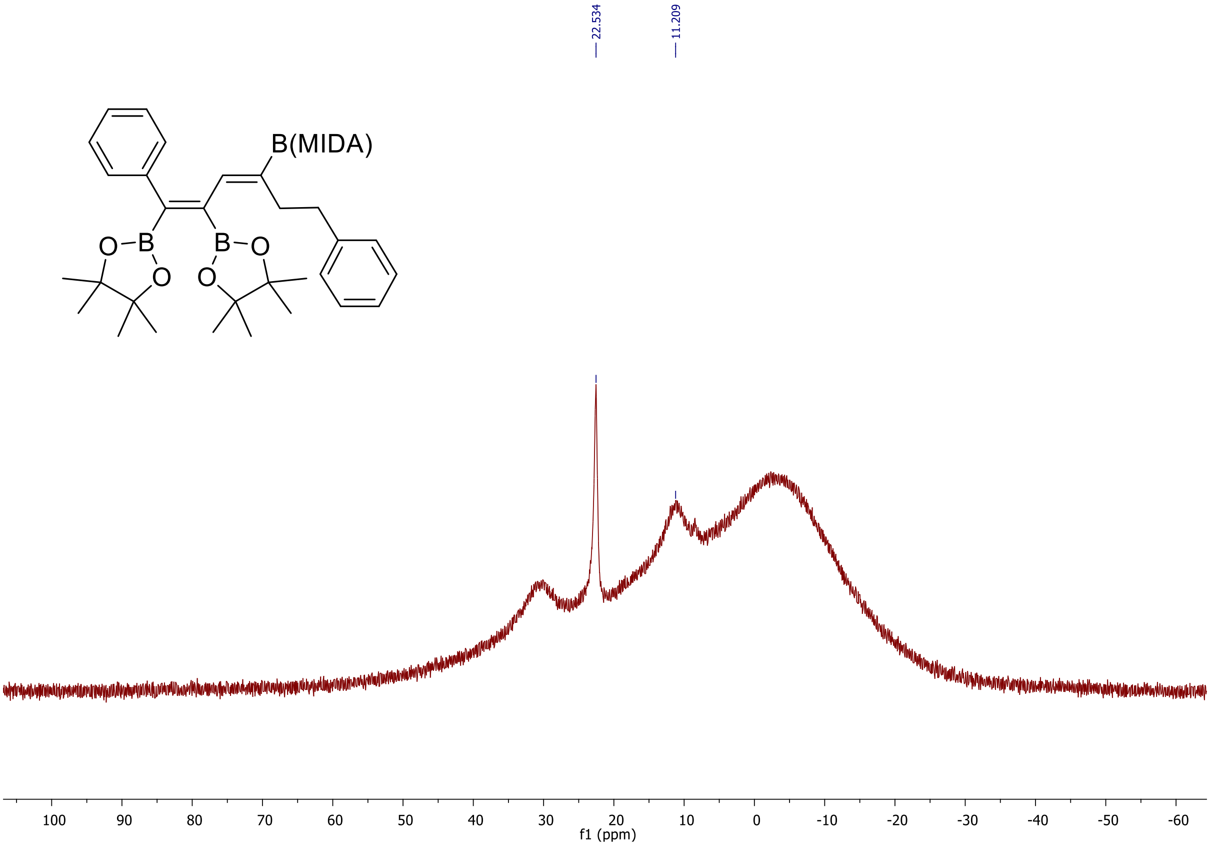

22.534
11.209
B(MIDA)
O
B
O
B
O
O
100 90 80 70 60 50 40 30 20 10 0 -10 -20 -30 -40 -50 -60
f1 (ppm)

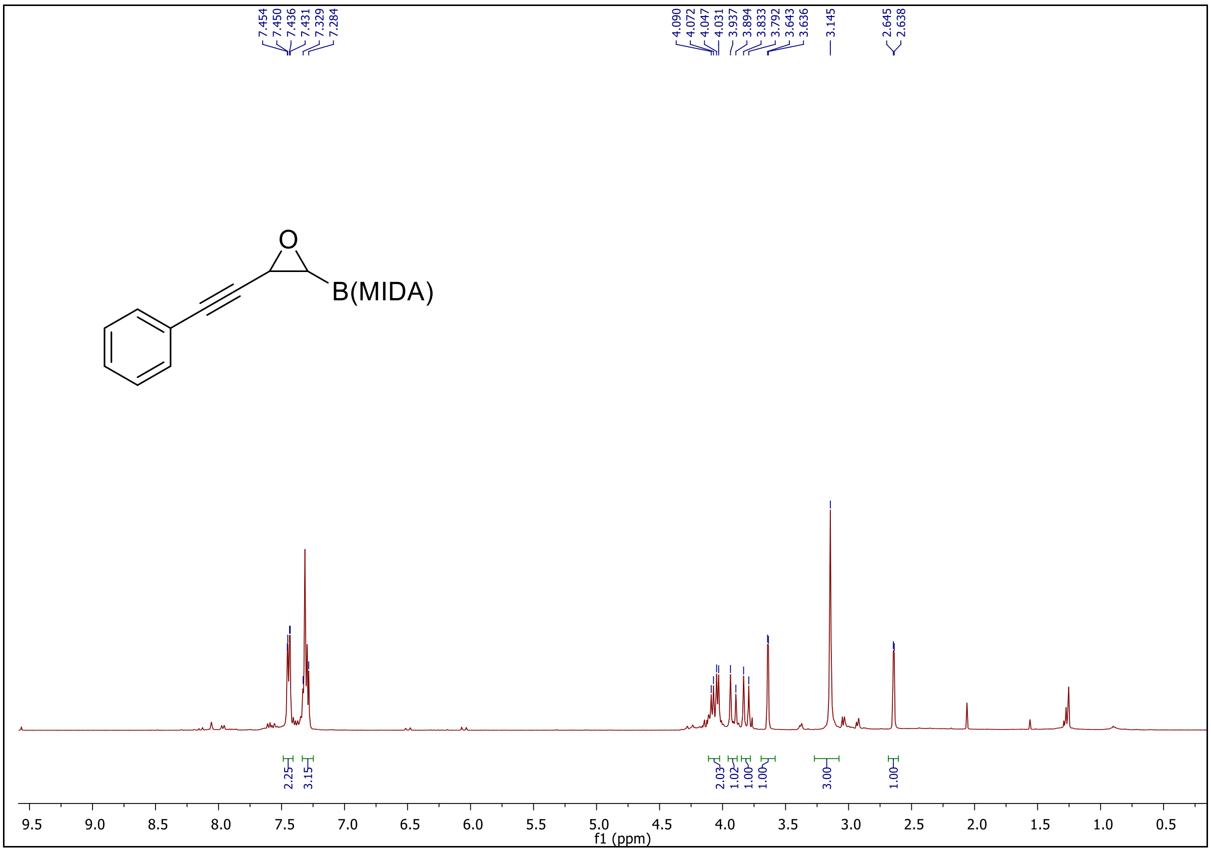

7.454
7.450
7.436
7.431
7.329
7.284
4.090
4.072
4.047
4.031
3.937
3.894
3.833
3.792
3.643
3.636
3.145
2.645
2.638
O
B(MIDA)
2.25
3.15
2.03
1.02
1.00
1.00
3.00
1.00
9.5 9.0 8.5 8.0 7.5 7.0 6.5 6.0 5.5 5.0 4.5 4.0 3.5 3.0 2.5 2.0 1.5 1.0 0.5
f1 (ppm)

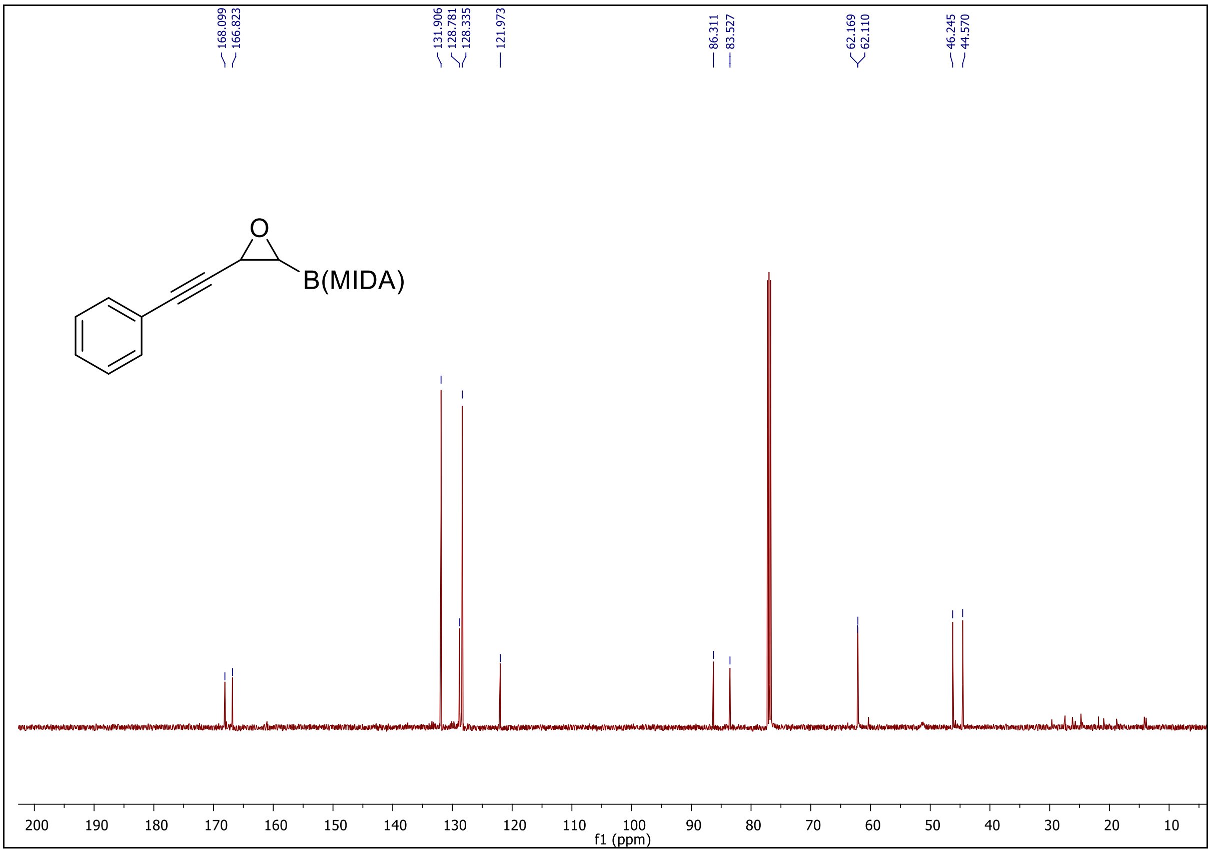
168.099
166.823
131.906
128.781
128.335
121.973
86.311
83.527
62.169
62.110
46.245
44.570
O
B(MIDA)
200
190
180
170
160
150
140
130
120
110
100
90
80
70
60
50
40
30
20
10
f1 (ppm)

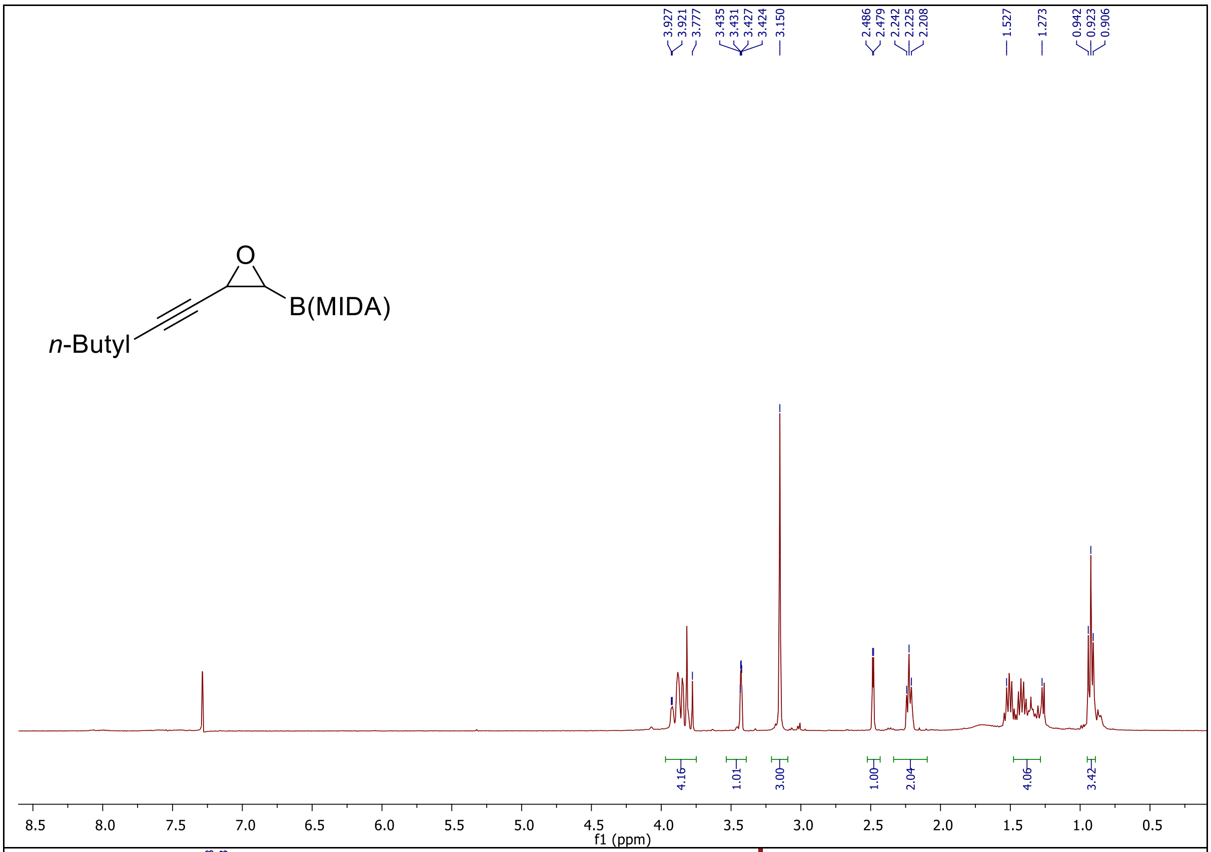
3.927
3.921
3.777
3.435
3.431
3.427
3.424
3.150
2.486
2.479
2.242
2.225
2.208
1.527
1.273
0.942
0.923
0.906
n-Butyl
B(MIDA)
O
4.16
1.01
3.00
1.00
2.04
4.06
3.42
f1 (ppm)

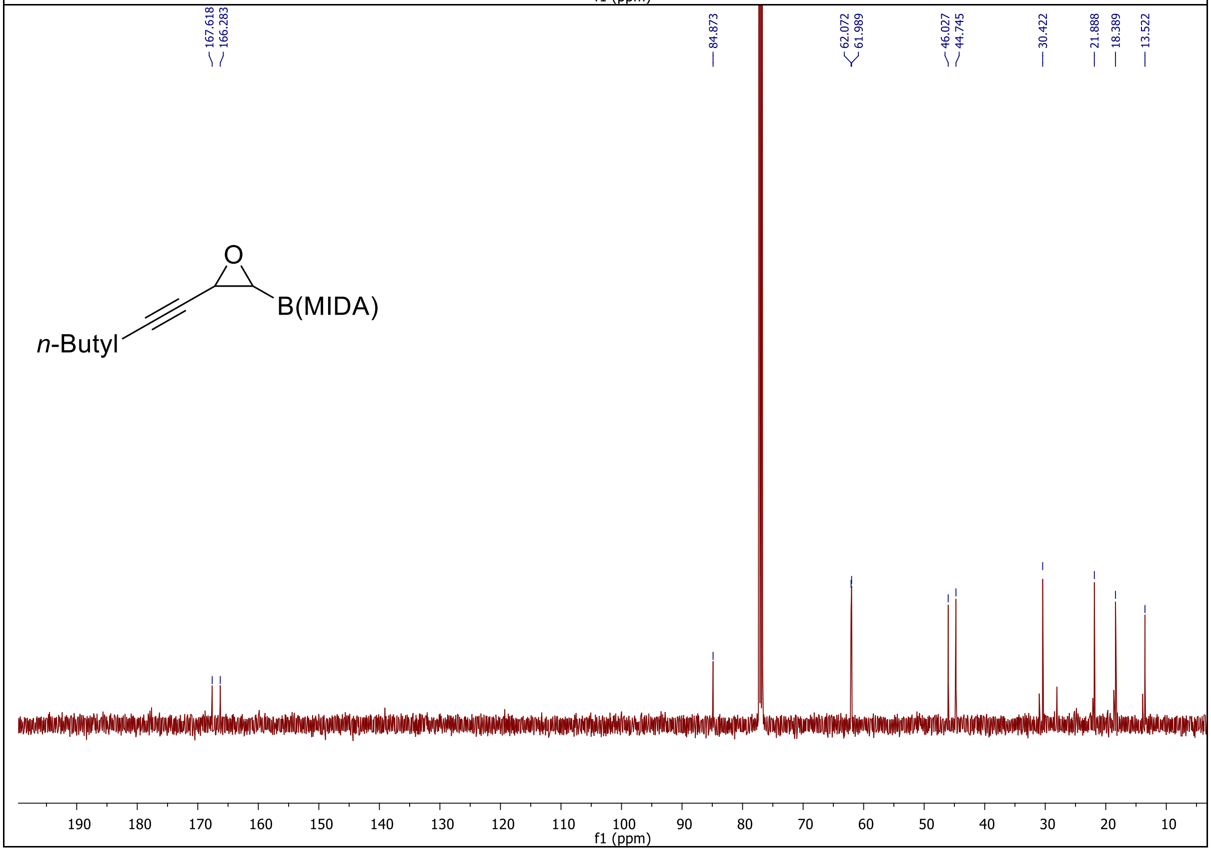
167.618
166.283
84.873
62.072
61.989
46.027
44.745
30.422
21.888
18.389
13.522
n-Butyl
B(MIDA)
O
f1 (ppm)

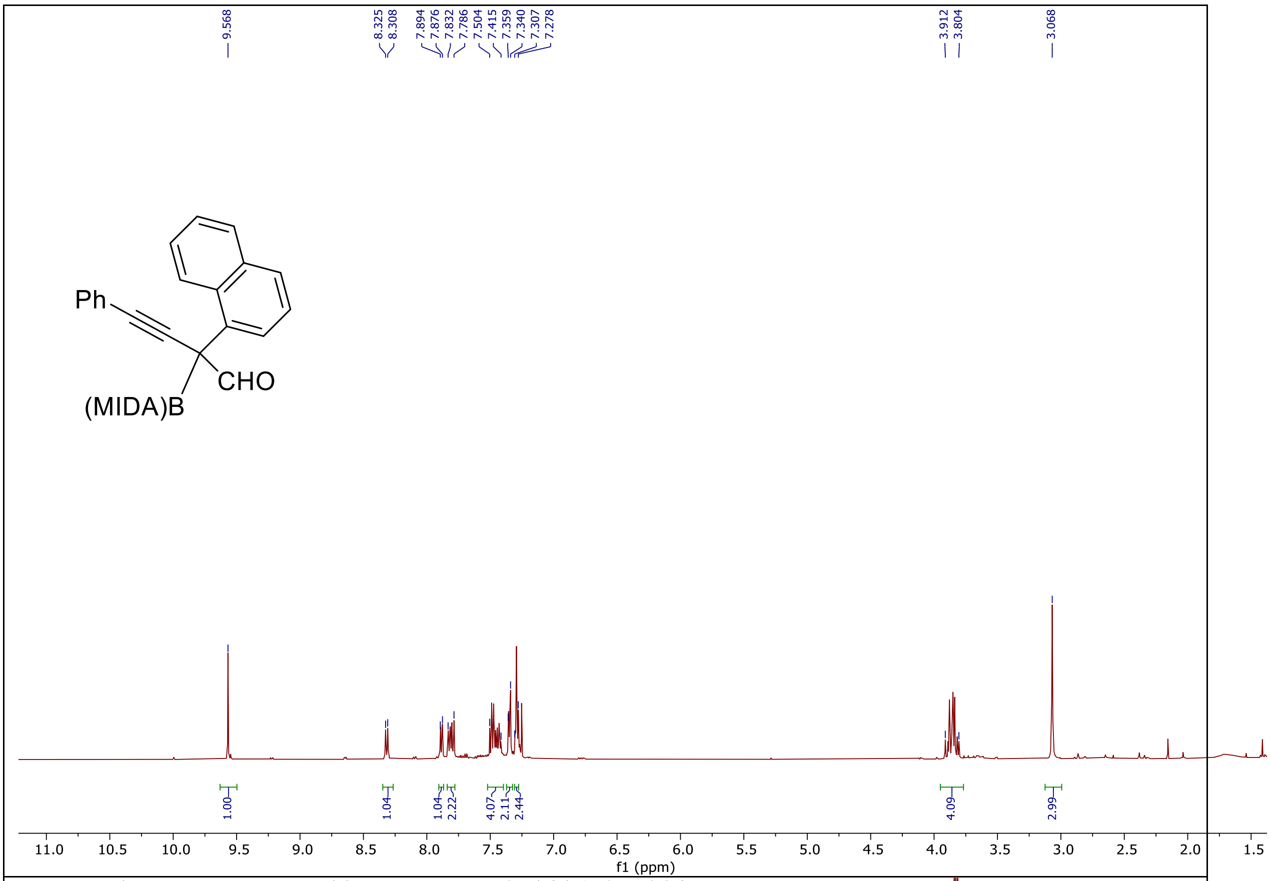
9.568
8.325
8.308
7.894
7.876
7.832
7.786
7.504
7.415
7.359
7.340
7.307
7.278
3.912
3.804
3.068
Ph
CHO
(MIDA)B
1.00
1.04
1.04
2.22
4.07
2.11
2.44
4.09
2.99
f1 (ppm)

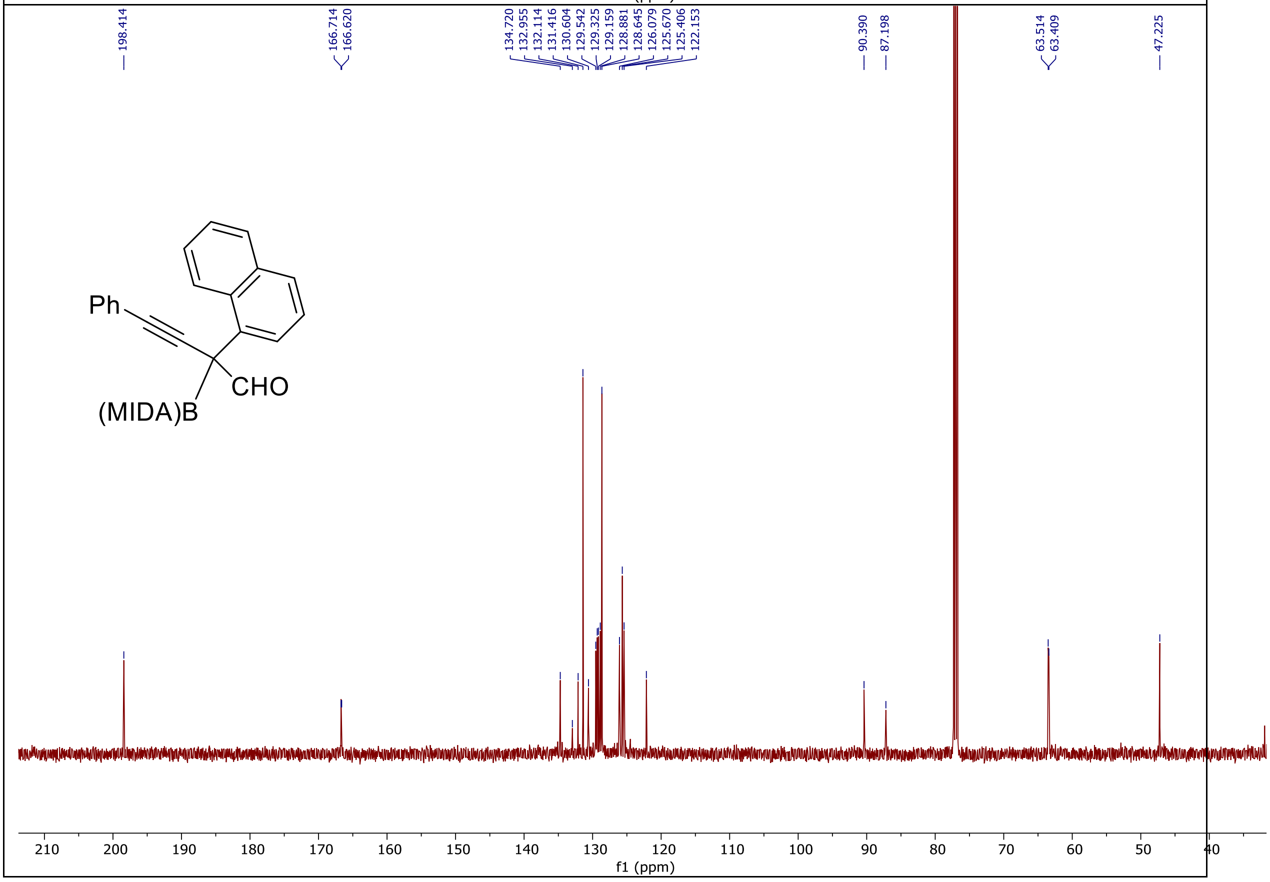
198.414
166.714
166.620
134.720
132.955
132.114
131.416
130.604
129.542
129.325
129.159
128.881
128.645
126.079
125.670
125.406
122.153
90.390
87.198
63.514
63.409
47.225
Ph
CHO
(MIDA)B
f1 (ppm)

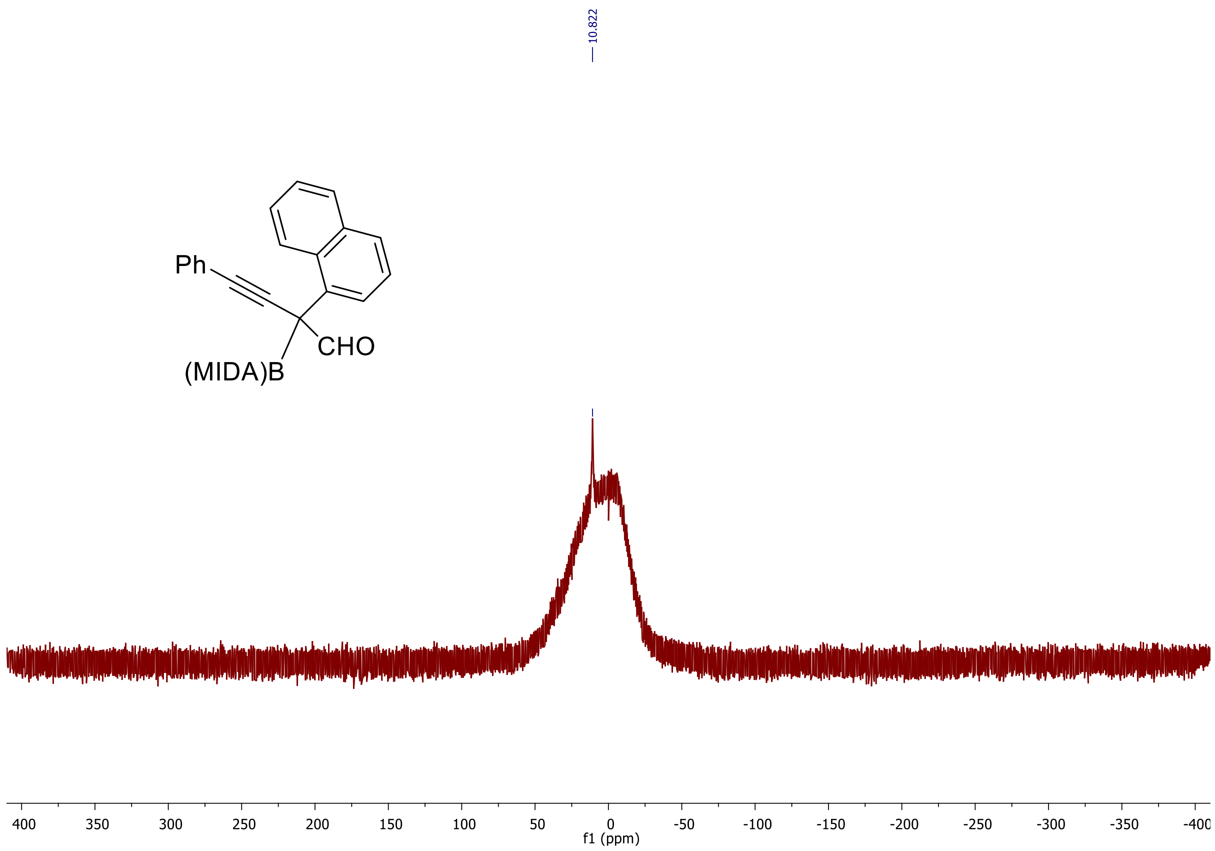

10.822
Ph
CHO
(MIDA)B
400
350
300
250
200
150
100
50
0
-50
-100
-150
-200
-250
-300
-350
-400
f1 (ppm)

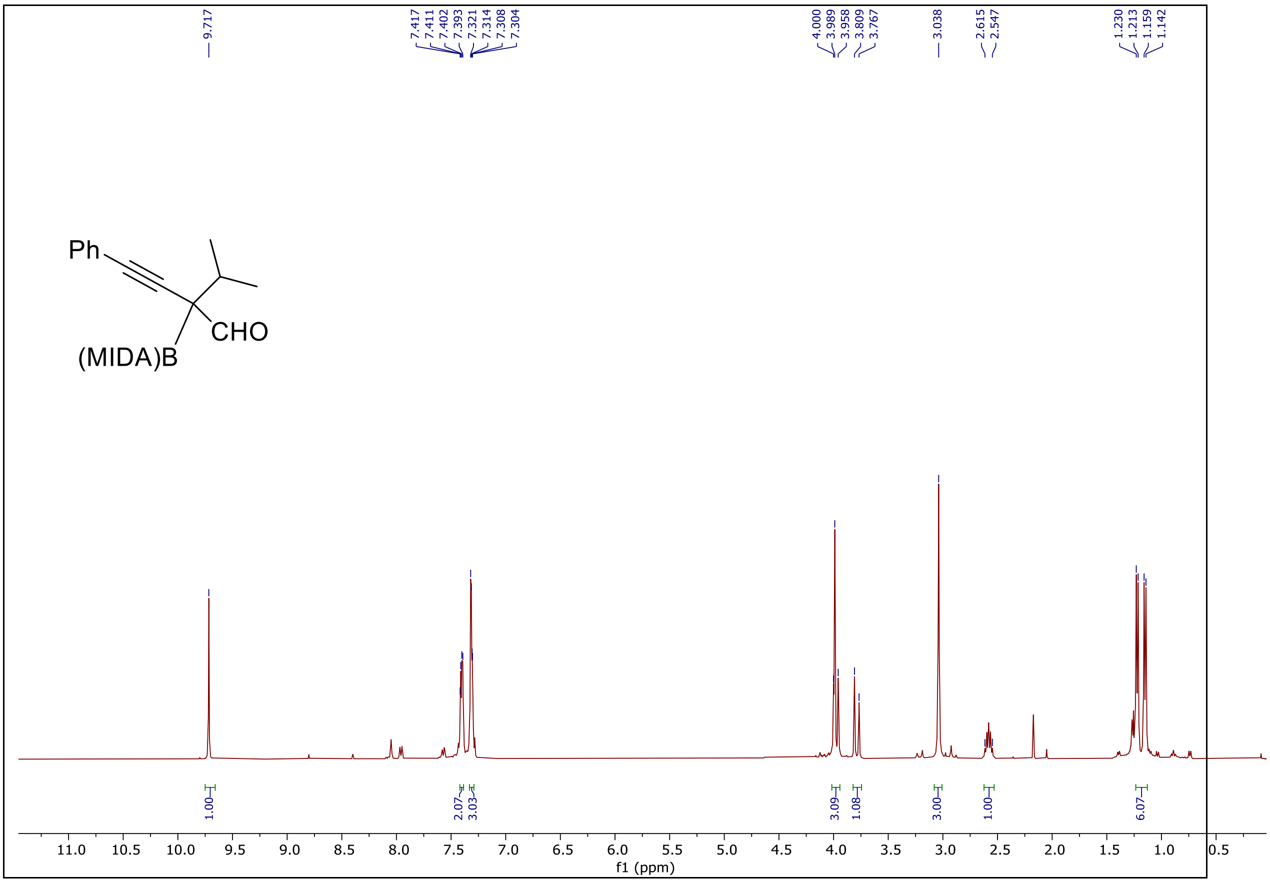

9.717
7.417
7.411
7.402
7.393
7.321
7.314
7.308
7.304
4.000
3.989
3.958
3.809
3.767
3.038
2.615
2.547
1.230
1.213
1.159
1.142
Ph
CHO
(MIDA)B
1.00
2.07
3.03
3.09
1.08
3.00
1.00
6.07
11.0
10.5
10.0
9.5
9.0
8.5
8.0
7.5
7.0
6.5
6.0
5.5
5.0
4.5
4.0
3.5
3.0
2.5
2.0
1.5
1.0
0.5
f1 (ppm)

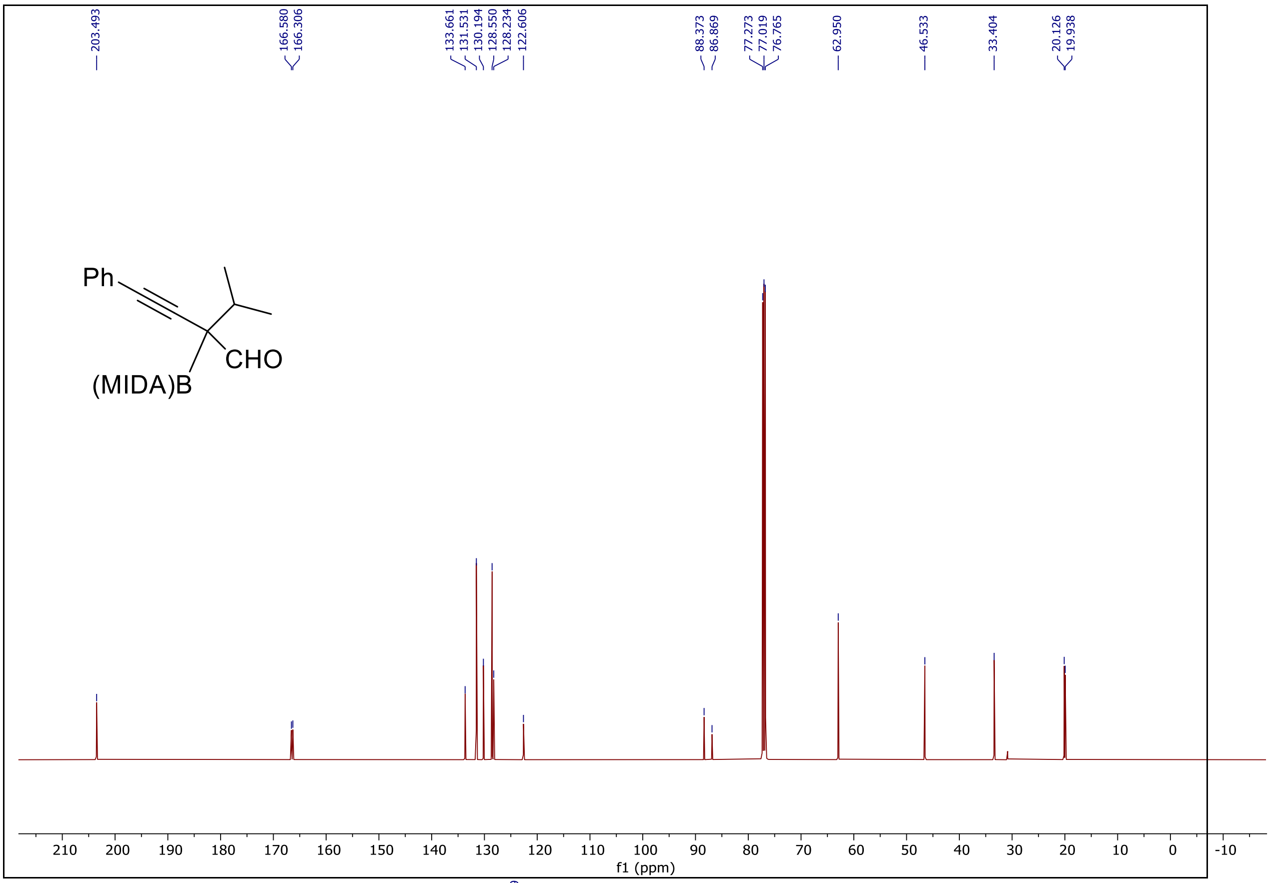
203.493
166.580
166.306
133.661
131.531
130.194
128.550
128.234
122.606
88.373
86.869
77.273
77.019
76.765
62.950
46.533
33.404
20.126
19.938
Ph
CHO
(MIDA)B
210
200
190
180
170
160
150
140
130
120
110
100
90
80
70
60
50
40
30
20
10
0
-10
f1 (ppm)

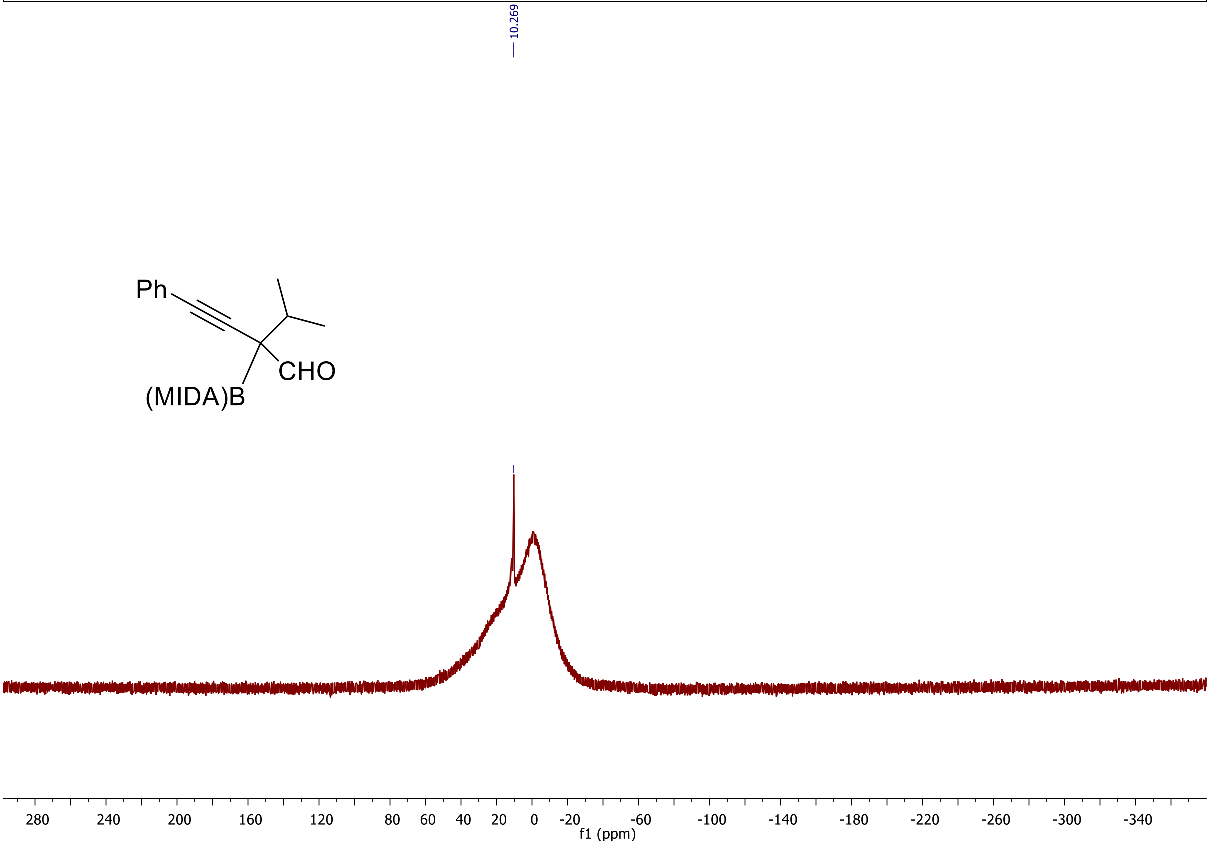
10.269
Ph
CHO
(MIDA)B
280
240
200
160
120
80
60
40
20
0
-20
-60
-100
-140
-180
-220
-260
-300
-340
f1 (ppm)

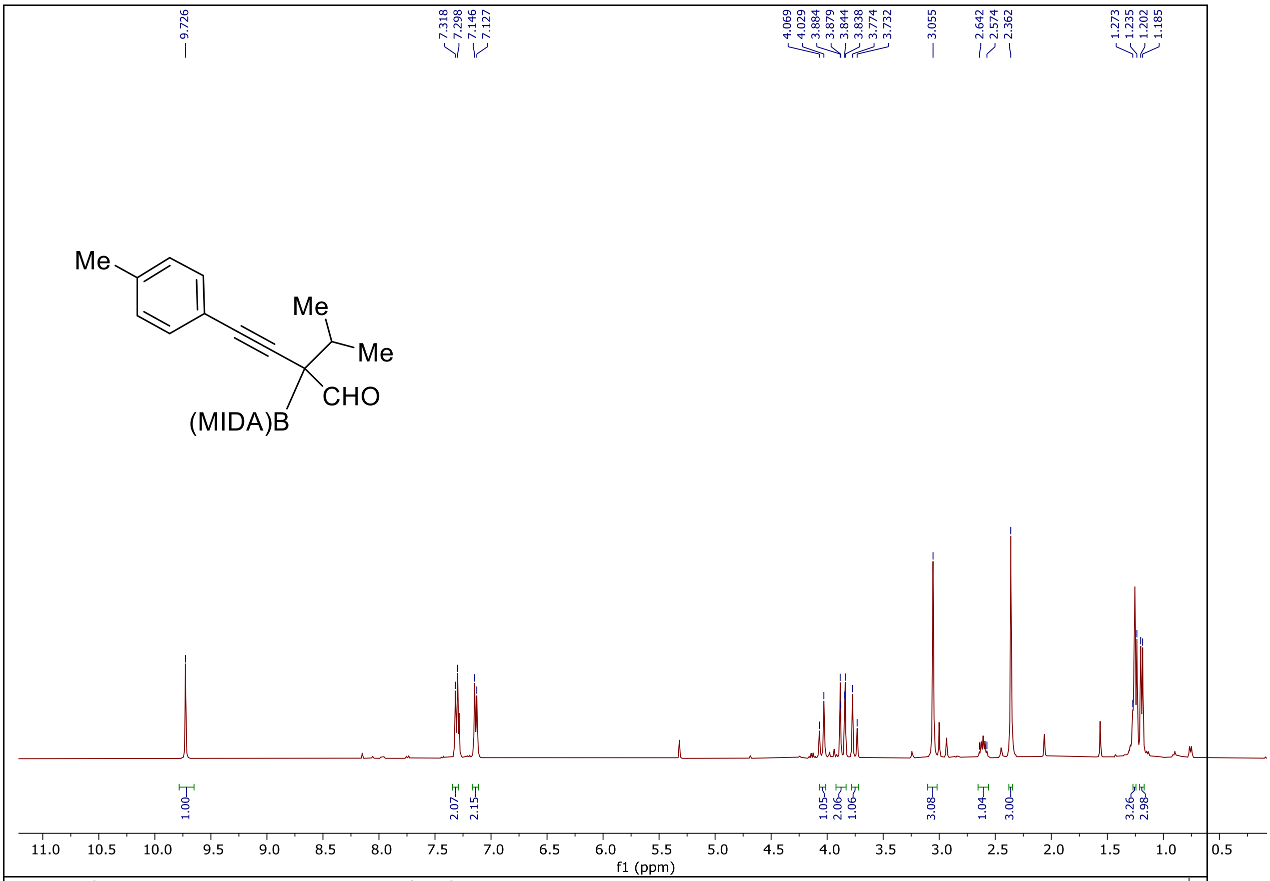

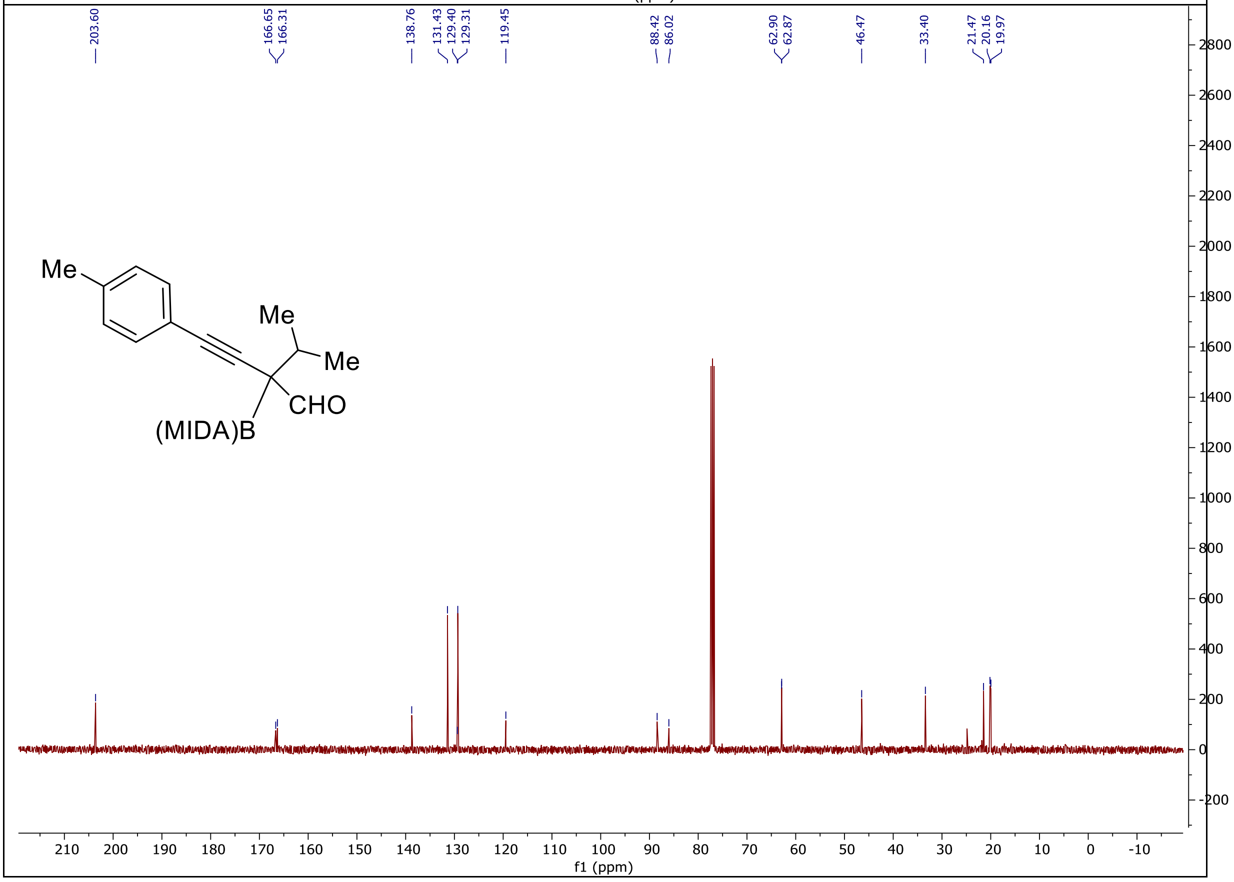

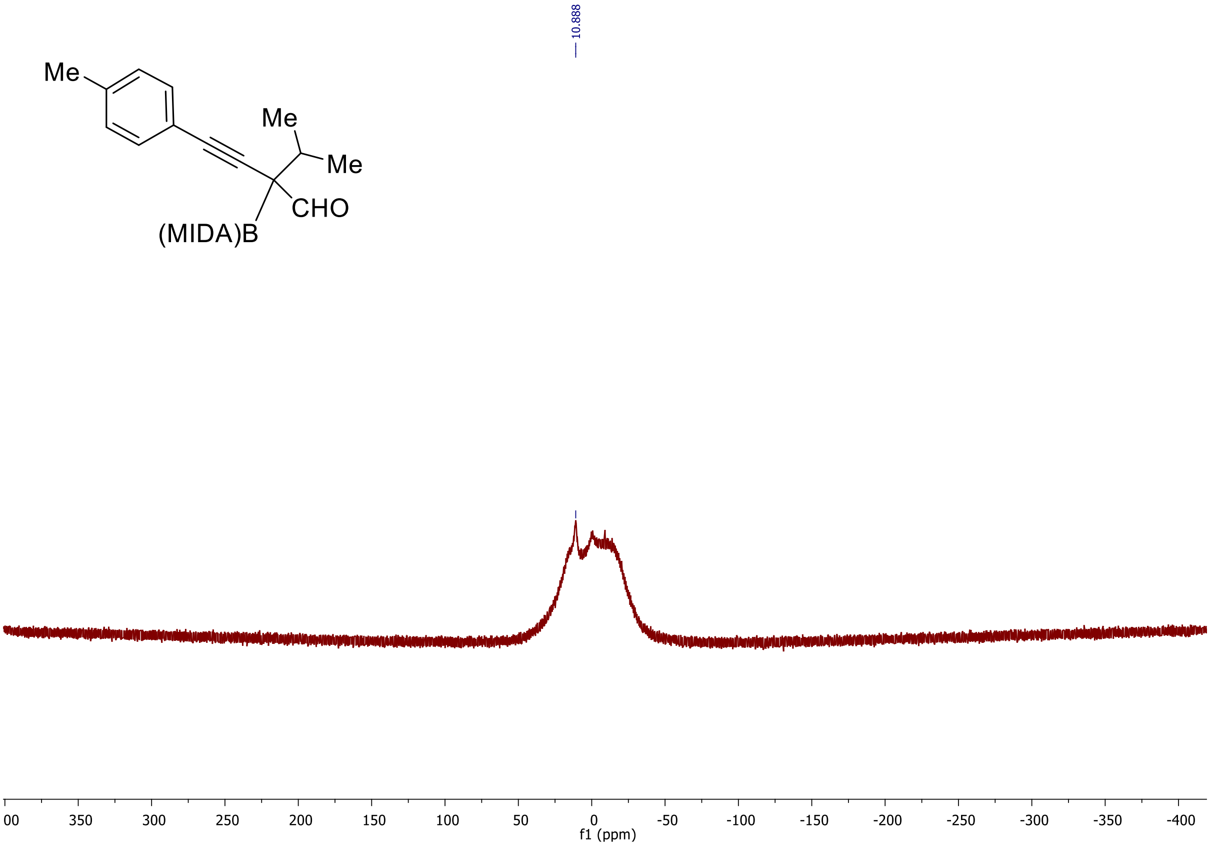
Me
Me
Me
CHO
(MIDA)B
10.888
f1 (ppm)

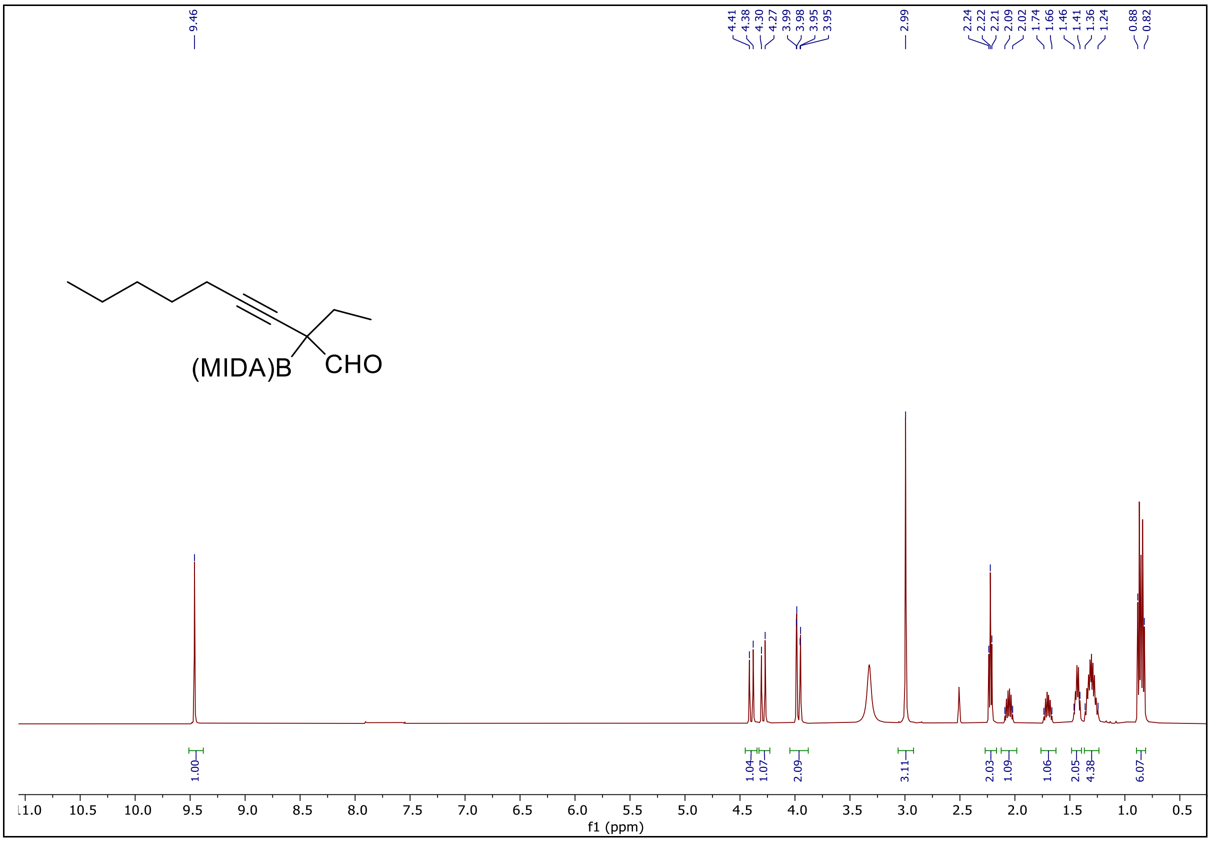
(MIDA)B
CHO
f1 (ppm)

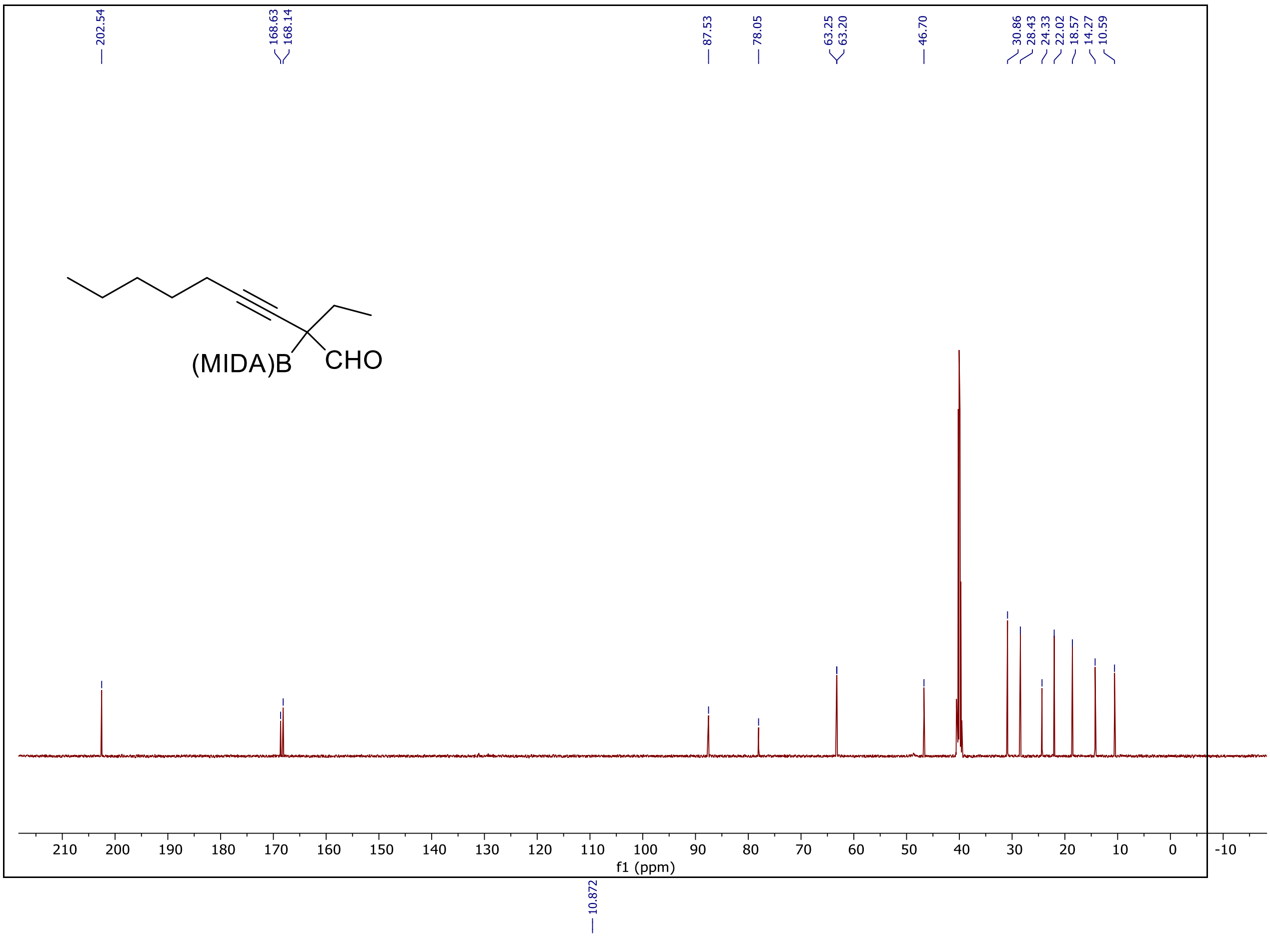

202.54
168.63
168.14
87.53
78.05
63.25
63.20
46.70
30.86
28.43
24.33
22.02
18.57
14.27
10.59
(MIDA)B
CHO
f1 (ppm)

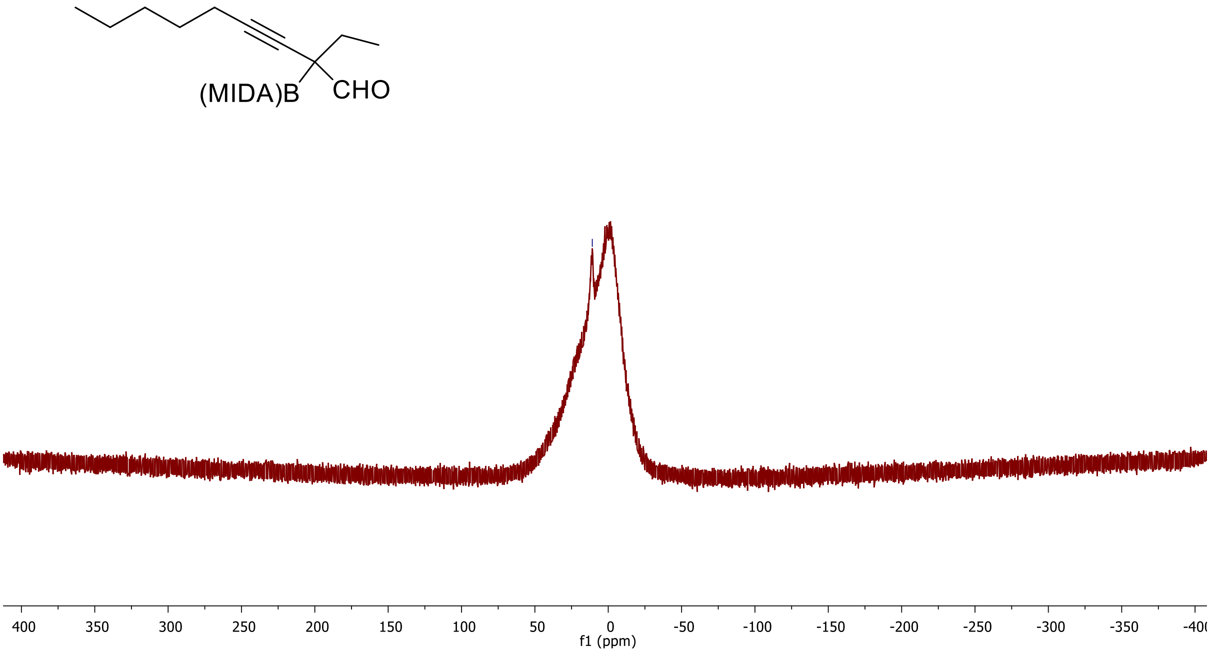

10.872
(MIDA)B
CHO
f1 (ppm)
